# Report on the sixth blind test of organic crystal structure prediction methods

**DOI:** 10.1107/S2052520616007447

**Published:** 2016-08-01

**Authors:** Anthony M. Reilly, Richard I. Cooper, Claire S. Adjiman, Saswata Bhattacharya, A. Daniel Boese, Jan Gerit Brandenburg, Peter J. Bygrave, Rita Bylsma, Josh E. Campbell, Roberto Car, David H. Case, Renu Chadha, Jason C. Cole, Katherine Cosburn, Herma M. Cuppen, Farren Curtis, Graeme M. Day, Robert A. DiStasio Jr, Alexander Dzyabchenko, Bouke P. van Eijck, Dennis M. Elking, Joost A. van den Ende, Julio C. Facelli, Marta B. Ferraro, Laszlo Fusti-Molnar, Christina-Anna Gatsiou, Thomas S. Gee, René de Gelder, Luca M. Ghiringhelli, Hitoshi Goto, Stefan Grimme, Rui Guo, Detlef W. M. Hofmann, Johannes Hoja, Rebecca K. Hylton, Luca Iuzzolino, Wojciech Jankiewicz, Daniël T. de Jong, John Kendrick, Niek J. J. de Klerk, Hsin-Yu Ko, Liudmila N. Kuleshova, Xiayue Li, Sanjaya Lohani, Frank J. J. Leusen, Albert M. Lund, Jian Lv, Yanming Ma, Noa Marom, Artëm E. Masunov, Patrick McCabe, David P. McMahon, Hugo Meekes, Michael P. Metz, Alston J. Misquitta, Sharmarke Mohamed, Bartomeu Monserrat, Richard J. Needs, Marcus A. Neumann, Jonas Nyman, Shigeaki Obata, Harald Oberhofer, Artem R. Oganov, Anita M. Orendt, Gabriel I. Pagola, Constantinos C. Pantelides, Chris J. Pickard, Rafal Podeszwa, Louise S. Price, Sarah L. Price, Angeles Pulido, Murray G. Read, Karsten Reuter, Elia Schneider, Christoph Schober, Gregory P. Shields, Pawanpreet Singh, Isaac J. Sugden, Krzysztof Szalewicz, Christopher R. Taylor, Alexandre Tkatchenko, Mark E. Tuckerman, Francesca Vacarro, Manolis Vasileiadis, Alvaro Vazquez-Mayagoitia, Leslie Vogt, Yanchao Wang, Rona E. Watson, Gilles A. de Wijs, Jack Yang, Qiang Zhu, Colin R. Groom

**Affiliations:** a The Cambridge Crystallographic Data Centre, 12 Union Road, Cambridge CB2 1EZ, England; b Chemical Crystallography, Chemistry Research Laboratory, Mansfield Road, Oxford OX1 3TA, England; c Department of Chemical Engineering, Centre for Process Systems Engineering, Imperial College London, London SW7 2AZ, England; d Fritz-Haber-Institut der Max-Planck-Gesellschaft, Faradayweg 4-6, 14195, Berlin, Germany; e Department of Chemistry, Institute of Physical and Theoretical Chemistry, University of Graz, Heinrichstraße 28/IV, 8010 Graz, Austria; f Mulliken Center for Theoretical Chemistry, Institut für Physikalische und Theoretische Chemie, Rheinische Friedrich-Wilhelms Universität Bonn, Beringstraße 4, 53115 Bonn, Germany; g School of Chemistry, University of Southampton, Southampton SO17 1BJ, England; h Radboud University, Institute for Molecules and Materials, Heyendaalseweg 135, 6525 AJ Nijmegen, The Netherlands; i Department of Chemistry, Princeton University, Princeton, NJ 08544, USA; j University Institute of Pharmaceutical Sciences, Panjab University, Chandigarh, India; k Department of Physics and Engineering Physics, Tulane University, New Orleans, LA 70118, USA; l Department of Physics, University of Toronto, Toronto, Canada M5S 1A7; m Department of Physics, Carnegie Mellon University, Pittsburgh, PA 15213, USA; n Department of Chemistry and Chemical Biology, Cornell University, Ithaca, NY 14853, USA; o Karpov Institute of Physical Chemistry, Moscow, Russia; p Utrecht University, The Netherlands; q OpenEye Scientific Software, 9 Bisbee Court, Suite D, Santa Fe, NM 87508, USA; r Center for High Performance Computing, University of Utah, 155 South 1452 East Room 405, Salt Lake City, UT 84112-0190, USA; s Department of Biomedical Informatics, University of Utah, 155 South 1452 East Room 405, Salt Lake City, UT 84112-0190, USA; t Departamento de Física and Ifiba (CONICET) Facultad de Ciencias Exactas y Naturales, Universidad de Buenos Aires, Ciudad Universitaria, Pab. I (1428), Buenos Aires, Argentina; u Educational Programs on Advanced Simulation Engineering, Toyohashi University of Technology, 1-1 Hibarigaoka, Tempaku-cho, Toyohashi, Aichi 441-8580, Japan; v Department of Computer Science and Engineering, Graduate School of Engineering, Toyohashi University of Technology, 1-1 Hibarigaoka, Tempaku-cho, Toyohashi, Aichi 441-8580, Japan; w Department of Chemistry, University College London, 20 Gordon Street, London WC1H 0AJ, England; x CRS4, Parco Scientifico e Tecnologico, POLARIS, Edificio 1, 09010 PULA, Italy; y FlexCryst, Schleifweg 23, 91080 Uttenreuth, Germany; z Institute of Chemistry, University of Silesia, Szkolna 9, 40-006 Katowice, Poland; aa Faculty of Life Sciences, University of Bradford, Richmond Road, Bradford BD7 1DP, England; bb Argonne Leadership Computing Facility, Argonne National Laboratory, Lemont, IL 60439, USA; cc Department of Chemistry, University of Utah, 155 South 1452 East Room 405, Salt Lake City, UT 84112-0190, USA; dd State Key Laboratory of Superhard Materials, Jilin University, Changchun 130012, People’s Republic of China; ee Department of Materials Science and Engineering and Department of Physics, Carnegie Mellon University, Pittsburgh, PA 15213, USA; ff NanoScience Technology Center, University of Central Florida, 12424 Research Parkway, PAV400, Orlando, FL 32826, USA; gg Department of Chemistry, University of Central Florida, 4111 Libra Drive PSB225, Orlando, FL 32816, USA; hh Department of Physics, University of Central Florida, 4111 Libra Drive PSB430, Orlando, FL 32816, USA; ii Department of Condensed Matter Physics, National Research Nuclear University MEPhI, Kashirskoye shosse 31, Moscow 115409, Russia; jj Department of Physics and Astronomy, University of Delaware, Newark, DE 19716, USA; kk School of Physics and Astronomy, Queen Mary University of London, London E1 4NS, England; ll Khalifa University, PO Box 127788, Abu Dhabi, United Arab Emirates; mm Cavendish Laboratory, 19, J. J. Thomson Avenue, Cambridge CB3 0HE, England; nn Department of Physics and Astronomy, Rutgers University, Piscataway, NJ 08854-8019, USA; oo Avant-garde Materials Simulation, Germany; pp Chair for Theoretical Chemistry and Catalysis Research Center, Technische Universität München, Lichtenbergstr. 4, D-85747 Garching, Germany; qq Department of Geosciences, Center for Materials by Design, and Institute for Advanced Computational Science, SUNY Stony Brook, NY 11794-2100, USA; rr Skolkovo Institute of Science and Technology, Skolkovo Innovation Centers, Bldg. 3, Moscow Region, 143026, Russia; ss Moscow Institute of Physics and Technology, 9 Institutskiy Lane, Dolgoprudny City, Moscow Region 141700, Russia; tt International Center for Materials Discovery, School of Materials Science and Engineering, Northwestern Polytechnical University, Xi’an 710072, China; uu Department of Materials Science and Metallurgy, University of Cambridge, 27 Charles Babbage Road, Cambridge CB3 0FS, England; vv Department of Physics and Astronomy, University College London, Gower St., London WC1E 6BT, England; ww Department of Chemistry, New York University, New York, NY 10003, USA; xx Physics and Materials Science Research Unit, University of Luxembourg, L-1511 Luxembourg; yy Courant Institute of Mathematical Sciences, New York University, New York, NY 10012, USA; zz NYU-ECNU Center for Computational Chemistry at NYU Shanghai, 3663 Zhongshan Road North, Shanghai 200062, China; aaa Department of Chemistry, Loyola University, New Orleans, LA 70118, USA

**Keywords:** crystal structure prediction, polymorphism, lattice energies, Cambridge Structural Database

## Abstract

The sixth blind test of organic crystal structure prediction (CSP) methods has been held, with five target systems: a small nearly rigid molecule, a polymorphic former drug candidate, a chloride salt hydrate, a co-crystal and a bulky flexible molecule. This blind test has seen substantial growth in the number of participants, with the broad range of prediction methods giving a unique insight into the state of the art in the field. Significant progress has been seen in treating flexible molecules, usage of hierarchical approaches to ranking structures, the application of density-functional approximations, and the establishment of new workflows and ‘best practices’ for performing CSP calculations. All of the targets, apart from a single potentially disordered *Z*′ = 2 polymorph of the drug candidate, were predicted by at least one submission. Despite many remaining challenges, it is clear that CSP methods are becoming more applicable to a wider range of real systems, including salts, hydrates and larger flexible molecules. The results also highlight the potential for CSP calculations to complement and augment experimental studies of organic solid forms.

## Abstract 2

The results of the sixth blind test of organic crystal structure prediction methods are presented and discussed, highlighting progress for salts, hydrates and bulky flexible molecules, as well as on-going challenges.

## 1. Introduction   

The ability to predict or explore the solid-state properties of molecules has long been a central aim of computational chemistry and materials science. The ultimate goal of crystal structure prediction (CSP) methods is to be able to explore the possible polymorphs, co-crystals, salts, hydrates *etc.* of a molecule based solely on minimal information such as its two-dimensional chemical diagram. This information could be used to predict or design novel solid forms, or determine the chance of undesirable polymorphs or solid forms occurring. The latter application of CSP methods is of particular importance for active pharmaceutical ingredients, due to the time and material cost of experimental solid-form screening and the serious consequences of unforeseen polymorphism or alternative solid forms.

Progress in the development of organic CSP methods over the past 15 years has been charted in a series of blind tests, hosted by the Cambridge Crystallographic Data Centre (CCDC). Five blind tests have been held to date, in 1999 (Lommerse *et al.*, 2000 ▸), 2001 (Motherwell *et al.*, 2002 ▸), 2004 (Day *et al.*, 2005 ▸), 2007 (Day *et al.*, 2009 ▸) and 2010 (Bardwell *et al.*, 2011 ▸). Participants were provided with the two-dimensional chemical diagram and crystallization conditions of a set of target systems where the experimental structure had been determined but not yet reported.

These tests have shown many advances, with the range and size of the target systems expanding from three relatively ‘simple’ molecules (Lommerse *et al.*, 2000 ▸), to tackling ‘drug-like’ molecules, co-crystals and polymorphic systems in the most recent fifth blind test (Bardwell *et al.*, 2011 ▸). In the fourth and fifth blind tests, all systems were predicted by at least one method (Neumann *et al.*, 2008 ▸; Day *et al.*, 2009 ▸; Bardwell *et al.*, 2011 ▸). However, the tests have highlighted many challenges, including accuracy of ranking methods, their computational cost and the applicability of methods for the full range of solid-form types, with salts, hydrates and larger molecules proving challenging in previous blind tests.

For many years, the focus of CSP research and the blind tests was often on predicting ‘the’ crystal structure of a molecule, with participants in previous blind tests submitting only three official predictions for each target. Recently, CSP methods have moved towards understanding the solid-form landscape of the putative structures they generate, with various factors influencing which structures are likely to be found experimentally (Price, 2013 ▸). At the same time, there has been considerable interest in using CSP methods to augment and understand experimental solid-form screening of pharmaceuticals (see, for example: Bhardwaj *et al.*, 2013 ▸; Ismail *et al.*, 2013 ▸; Kuleshova *et al.*, 2013 ▸; Neumann *et al.*, 2015 ▸), organic semiconductors (Valle *et al.*, 2008 ▸) and microporous materials (Pyzer-Knapp *et al.*, 2014 ▸). Density-functional approximations (DFAs), which have been some of the most promising tools for ranking the stability of possible crystal structures have also developed considerably, with many new van der Waals (vdW)-inclusive methods (Klimeš & Michaelides, 2012 ▸) particularly suited to modelling molecular materials (Reilly & Tkatchenko, 2015 ▸; Kronik & Tkatchenko, 2014 ▸; Brandenburg & Grimme, 2014 ▸). New developments in CSP codes and algorithms have also been reported (Habgood *et al.*, 2015 ▸; Wang *et al.*, 2012 ▸; Lund *et al.*, 2015 ▸; Zhu *et al.*, 2012 ▸; Obata & Goto, 2015 ▸), while there have been a number of new insights into conformational polymorphism (Cruz-Cabeza & Bernstein, 2014 ▸; Thompson & Day, 2014 ▸).

On the basis of this shift in the focus of CSP and new methodological developments and insights, a sixth blind test of organic CSP methods was launched in 2014. The aims of this test were to provide a fair benchmark of the state-of-the-art in CSP methodology, to spur on the continued development of CSP methods, and to provide a platform to communicate progress and challenges for CSP research with the wider scientific community (Groom & Reilly, 2014 ▸). To this end, this blind test has seen more challenging and ‘realistic’ target systems and changes in the nature of submissions to ensure as much information and as many insights as possible can be gained from the blind test.

This paper reports the overall results of the blind test, and its structure is as follows: the blind-test procedure and selection of targets is outlined in §2, a brief report of the methods and approaches employed is given in §3 and a summary and discussion of the results is presented in §4, including a discussion of current challenges in §4.8. With 25 submissions, the volume of data and information precludes a detailed discussion of every result. However, the supporting-information documents of each submission (part of the supporting information of this paper) provide important context for the trends and general results presented in the main paper, and the interested reader is encouraged to consult these.

## 2. Organization and approach   

Previous blind tests largely followed the same format with the number and complexity of the target systems increasing over the years. Following dialogue with the CSP community in early 2014, a number of changes were made to the organization of the sixth blind test, which are outlined in the following subsections.

### 2.1. Target categories and selection   

In the previous blind test (Bardwell *et al.*, 2011 ▸), six target categories were employed, covering simple and more complex rigid molecules, partially flexible molecules, salts and co-crystals, flexible molecules and polymorphic systems. Finding unpublished crystal structures of small rigid molecules containing only CHNO atoms proved very difficult in the fifth blind test, as did finding a polymorphic system (Bardwell *et al.*, 2011 ▸). Therefore, the target categories for the sixth blind test were adjusted to remove the small rigid CHNO molecule target and the separate polymorphic system. In addition, co-crystals and salts, which had been a single category previously, were split into two separate categories, resulting in five target categories:

(1) Rigid molecules, with functional groups restricted to CHNO, halogens, S, P, B; one molecule in the asymmetric unit; up to about 30 atoms.

(2) Partially flexible molecules with two to four internal degrees of freedom; one molecule in the asymmetric unit; up to about 40 atoms.

(3) Partially flexible molecule with one or two internal degrees of freedom as a salt; two charged components in the asymmetric unit, in any space group; up to about 40 atoms.

(4) Multiple partially flexible (one or two degrees of freedom) independent molecules as a co-crystal or solvate in any space group; up to about 40 atoms.

(5) Molecules with four to eight internal degrees of freedom; no more than two molecules in the asymmetric unit, in any space group; 50–60 atoms.

One of the most challenging aspects of organizing the blind tests has been finding suitable unpublished crystal structures that fit these categories. In addition to being unpublished, the structures must be of high quality and have all atoms located. As in previous blind tests, the structures were also required to be free of disorder. The collection of potential experimental structures for these categories took place in summer 2014. A number of crystallographers were contacted and asked to send information on any suitable targets directly to an external referee, Professor Richard Cooper (University of Oxford). A general request for structures was also included in the announcement of the blind test (Groom & Reilly, 2014 ▸). The full experimental structures were known only to the external referee, who also made the final selection of candidates, enabling the CCDC itself to participate in the blind test.

#### 2.1.1. Selection of suitable targets   

Following the initial requests, 20 unpublished structures were submitted for consideration. Of these, ten were considered candidates for category 2, four were considered for category 4, and two fell into each of the remaining categories. A further request yielded some additional possible category 1 and 2 structures. The final targets are given in Table 1 ▸ and are numbered (XXII)–(XXVI), following on from the 21 molecules and systems studied in previous blind tests.

All three potential category 1 molecules contained one or more ring systems with more than one possible conformation. Molecule (XXII) contains no rotatable bonds but the molecule is ‘hinged’ about the six-membered ring, introducing some flexibility, with the flat molecule representing a saddle point *in vacuo*. However, the hinged conformation and flexibility was deemed to be predictable, although participants were not provided with the conformation.

Molecule (XXIII) was disclosed along with five known crystal structures (*A*–*E*) and experimental determination of the most stable polymorphs at 257 and 293 K through slurrying experiments. The molecule formally has five rotatable bonds but an intramolecular hydrogen bond between the amine and carboxylic acid group constrains two of these to be almost planar in the observed crystal structures, although a complete CSP calculation would need to explore the possibility of the molecule not forming such a hydrogen bond. The presence of two *Z*′ = 2 polymorphs (*C* and *E*) also stretches the requirements of category 2, but given there were three other *Z*′ = 1 crystal structures as potential structure prediction targets, it was decided that this would not make the target too difficult. One of the two molecules in the asymmetric unit of form *E* has significantly larger anisotropic displacement parameters than the other, particularly for the ethyl linker between the two phenyl rings (see Fig. S1 of the supporting information). While this suggests that there is potentially disorder in the structure, it was still deemed a valid target.

Structure (XXIV) was chosen from two candidates and satisfied the criteria of category 3. Although containing only 11 non-H atoms, it did contain an additional solvent of crystallization, which increases the difficulty of the structure prediction problem.

Structure (XXV) was chosen from four candidates as the best example of a co-crystal that satisfied the category 4 criteria. Both molecules in the structure appeared to be quite rigid, but the two possible hydrogen-bonding interactions between the molecules retained some of the complexity. The original experimental data for molecule (XXV) were collected at room temperature. They were remeasured after the blind test at 100 K, which revealed that there is a significant amount of proton transfer from the carboxylic acid group to the amine. A competitive refinement determined proton occupancies of 0.58 (3) on the carboxylic acid oxygen and 0.42 (3) on the nitrogen.

Molecule (XXVI) was one of two possibilities for category 5 and contains five rotatable bonds, with each half of the topologically symmetric molecule adopting different conformations in the solid state. Molecule (XXVI) was screened for additional polymorphs by Johnson Matthey (Pharmorphix). The study found one high-temperature polymorph and several solvates.

### 2.2. Structure of the blind test   

The primary aims of the sixth blind test were to enable the CSP community to perform a fair benchmark of their methodologies, provide a platform to communicate progress and state-of-the art in the field and to spur new development in the methodologies. To further these aims, the format and structure of this latest blind test differs from the previous one in a number of areas.

In previous blind tests, participants were allowed to submit three predicted crystal structures for each target as their principal predictions, although they were encouraged to submit extended lists of structures resulting from their predictions for further analysis. This is not in keeping with the more recent focus of CSP methods on solid form landscapes and the insight they can provide on the multiple likely solid forms of a molecule. The restriction of submitting only three structures as principal predictions also created an arbitrary cut-off point for what was considered a successful prediction. In choosing their three structures, some participants combined different analysis or ranking approaches, highlighting that various information and calculations can be complementary.

Reflecting all these points, each submission in the sixth blind test could contain up to 100 predicted structures ranked in order of their likelihood using some form of fitness function. Participants were also allowed to submit a second list of 100 structures, which could be generated or re-ranked using alternative methods. The purpose of these changes was to maximise the information and insight gained from the blind test. For this reason, re-ranking submissions, where a submission solely re-ranked structures provided by other participants, were also permitted for this blind test. This allowed a number of research groups developing ranking approaches [*e.g.* bespoke potentials, density-functional theory (DFT) and quantum-chemical methods] to apply their methods under blind-test conditions.

Participants were required to submit a supporting-information document that would provide a clear summary of their methodology at the time of submission, as opposed to optionally providing one afterwards. These changes in procedure were agreed through dialogue with potential participants in spring and summer 2014. Previous participants in blind tests and anyone who had expressed interest in any new blind tests were invited *via* email to take part in the sixth blind test, while an open invitation was published on the CCDC and IUCr websites and in *Acta Cryst. B* (Groom & Reilly, 2014 ▸).

The two-dimensional chemical diagrams and crystallization conditions (Table 1 ▸) were sent to researchers interested in participating on 12 September 2014 by the referee, with a deadline for submissions of 31 August 2015. As in previous blind tests, participants were not required to attempt all five target systems. A number of researchers expressed interest after the start date and were also allowed to participate. In the week following the submission deadline the predicted structures were compared with the experimentally known ones by the CCDC and the referee. Participants were then sent the experimental structures on 7 September 2015, and the results confirmed by mid-September 2015. A workshop was held to discuss the results in October 2015 in Cambridge, UK.

### 2.3. Assessment of predictions   

The predicted crystal structures submitted by participants were compared with the experimentally known crystal structures using the Crystal Packing Similarity Tool (Chisholm & Motherwell, 2005 ▸), as available through the CSD Python API (Groom *et al.*, 2016 ▸) and *Mercury* 3.6 (Macrae *et al.*, 2008 ▸). The tool represents a crystal structure using a cluster of *N* molecules comprised of a central reference molecule and (*N* − 1) nearest-neighbour molecules. The distances and a subset of the triangles that define the reference cluster are then used as a three-dimensional substructure-search query within the comparison structure. For this search, two molecules within the packing shells are considered to match if these distances agree within 25% and the angles of the triangles agree within 25°. Those molecules that match are then overlaid and a root mean-squared deviation (RMSD) is calculated.

The result of the comparison is a number of molecules that match, *n*, between the two packing shells and a corresponding RMSD_*n*_ for those matching molecules. Where multiple clusters can be defined for an input crystal (*i.e.* *Z*′ > 1 or structures submitted in *P*1 symmetry) the best result is retained. The Crystal Packing Similarity Tool normally considers only heavy atoms when calculating distances and angles within clusters and for the final RMSD analysis, ignoring H-atom positions due to their limited accuracy in standard X-ray diffraction crystal structures. However, matching and overlay of the heavy atoms does require the number of H atoms bonded to them to be the same. Predicted structures were deemed to match an experimental structure when 20 out of 20 molecules matched. The largest RMSD_20_ value was approximately 0.8 Å. A single predicted structure of (XXV) approximately matched the experimental structure, but with an RMSD of more than 1.2 Å, which was deemed too far from the experimental geometry.

For (XXIII), some of the predicted crystal structures have the same heavy-atom positions as the experimental structure but place the carboxylic acid H atom on the oxygen closest to the NH group. The analysis for these systems was therefore performed twice, once requiring the H atom to be located as in the experimental structure and a second time where the H-atom location and connectivity was not considered.

In the case of (XXIV), each of the three components in the asymmetric unit counts towards *N*, therefore a cluster of 20 components does not amount to the same physical extent as for the other systems. In addition, H-atom positions are particularly important for this system. Therefore, initial analysis was performed ignoring H-atom positions and with *N* = 20. If a match was found, the analysis for that structure was re-run considering H-atom positions and with *N* = 60 to confirm the match.

Finally, after the blind test had concluded it was discovered that the hydrogen-bonding proton in (XXV) is disordered, making the structure a mixture of a molecular salt and a co-crystal. Therefore, the analysis of (XXV) was performed twice to find both co-crystal and salt matches to the experimental heavy-atom coordinates.

## 3. Methodologies   

There are a wide variety of approaches to predicting organic crystal structures. The larger number of submissions in this blind test has seen a number of new approaches being applied in a blind test for the first time. Broadly speaking, the CSP process can be broken down into a series of steps:

(i) Exploration of the conformational preferences of the target molecules.

(ii) Generating plausible crystal-packing arrangements of the target molecules.

(iii) Ranking the likelihood of resulting crystal structures forming using some form of scoring or fitness function.

There are, however, many variations on these steps. In this section we summarize some of the approaches used in the current blind test. Brief details of the approach used in each submission are given in Table 2 ▸, while full details are provided in the supporting information document that accompanied each submission.

### 3.1. Molecular structure generation and conformational analysis   

For many approaches to predicting crystal structures, the first stage is to explore the conformational flexibility of the target molecules. This can help to define a set of rigid conformations that some methods use for structure generation, while in other methods this information is used to define and limit the flexible degrees of freedom explored in tandem with the unit-cell degrees of freedom. Not all approaches require this information though, with some exploring molecular degrees of freedom in the search stage in an unbiased way or with implicit limits imposed by the search strategy.

In several approaches, the initial starting conformations for molecules were determined using *ab initio* calculations of isolated molecules in the gas phase, including ‘scans’ of specific degrees of freedom (such as torsions), which have been used to understand the extent of flexibility of a molecule and define conformations. Information on conformational preferences from the Cambridge Structural Database (Bruno *et al.*, 2004 ▸) has been combined with *ab initio* data in some methods, and also used to directly generate conformations in one approach.

In some cases, force fields have been used for the initial stages of exploring flexibility, which allows one to apply more exhaustive methods for exploring conformational flexibility, such as low-mode conformational searches (Kolossváry & Guida, 1996 ▸), systematic grid searches and perturbations of initial conformations, including CONFLEX conformational searches (Goto & Osawa, 1989 ▸; Goto & Osawa, 1993 ▸). In many cases, the resulting conformations were then optimized using *ab initio* methods.

### 3.2. Crystal structure generation   

There are a plethora of methods for generating possible organic crystal structures, which requires exploring the degrees of freedom of the unit cell (up to six lattice parameters), the position and orientation of molecules in the unit cell and, in some cases, internal molecular degrees of freedom. As in the previous blind test, the majority of methods employ some variation on random or quasi-random searches to generate trial crystal structures (Submissions 3, 5–7, 10, 11, 15, 16 and 18–20), with four submissions (3, 15, 18, 19) using low-discrepancy Sobol’ sequences (Sobol’, 1967 ▸). Monte Carlo simulated annealing (Submissions 1 and 13) and parallel tempering (Submission 14) have also been used, as have systematic grid searches (Submissions 4, 9, 17) and evolutionary and genetic algorithms (Submissions 8, 12 and 21). Shape matching of the target systems to known experimental structures in the CSD has been employed in one submission to generate analogue crystal structures (Submission 2).

An important choice in the structure-generation process is the consideration of the set of space groups or *Z* values to consider in the search. The majority of submissions imposed crystallographic symmetry, explicitly exploring a set of space groups, typically chosen on the basis of frequencies of occurrence in the CSD. For some submissions, parts of the ranking or generation process, including some DFT codes and MD simulations, do not fully conserve the crystallographic symmetry. Software and utilities including *PLATON* (Spek, 2009 ▸), *PyMatGen* (Ong *et al.*, 2013 ▸), *FINDSYM* (Stokes & Hatch, 2005 ▸) and *Spglib* (Spglib, 2015 ▸) have been used to detect and enforce such symmetry in the final submitted structures.

As noted above, some methods explore the molecular degrees of freedom as part of the search for putative crystal structures. This can be important, as conformers that appear unstable for the molecule *in vacuo* can be found in the stable crystal structure of the molecule (Thompson & Day, 2014 ▸), while in some cases the solid-state conformation may not even correspond to a conformer on the isolated molecule’s potential-energy surface. More than half of the search methods in the present blind test allowed for some molecular flexibility while exploring the search space and many of those that performed only a rigid-conformation search used a set of likely or low-energy conformations or were attempting only molecule (XXII), which contains no rotatable bonds.

### 3.3. Optimization and ranking   

The final stage of predicting crystal structures is to optimize or minimize the energy of the raw crystal structures generated and then rank them in order of stability or likelihood of occurrence. All of the submissions in this blind test used some form of energy-based metric to rank structures.

In a number of methods, a hierarchical approach has been adopted, in which a less intensive computational method or algorithm is used initially, for example, generic or tailor-made empirical potentials (Neumann, 2008 ▸) or ‘coarse’ evaluation of DFT energies, including the use of a modified Harris approximation to calculate solid-state charge densities from molecular charge densities (Submission 12). More computationally demanding methods and algorithms were then employed for the final set of structures closest to the global minimum. In a number of submissions the final ranking was performed using potentials based on distributed multipole electrostatics (Stone, 2005 ▸; Price *et al.*, 2010 ▸), *ab initio* intramolecular energies (Kazantsev *et al.*, 2011 ▸; Habgood *et al.*, 2015 ▸) and various dispersion–repulsion potentials. Other methods employed generic force fields, sometimes fitted to *ab initio* or experimental data or augmented with *ab initio* conformational energies (van Eijck *et al.*, 2001*a* ▸), while three submissions shared potentials derived from symmetry-adapted perturbation theory based on DFT [SAPT(DFT)] calculations (Misquitta *et al.*, 2005 ▸) of (XXII) (Submissions 17, 19 and 20).

DFT has seen extensive use with a range of vdW-inclusive density-functional approximations (DFAs) (Klimeš & Michaelides, 2012 ▸) being applied. These include the Neumann–Perrin (Neumann & Perrin, 2005 ▸), D2 (Grimme, 2006 ▸), TS (Tkatchenko & Scheffler, 2009 ▸), XDM (Becke & Johnson, 2007 ▸), D3 (Grimme *et al.*, 2010 ▸) and MBD (Tkatchenko *et al.*, 2012 ▸; Ambrosetti *et al.*, 2014 ▸) methods, as well as two vdW density functionals, vdW-DF (Dion *et al.*, 2004 ▸) and optB86b-vdW (Klimeš *et al.*, 2011 ▸). These treatments differ in the way the dispersion interaction is modelled. Many of the methods are based on 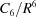 terms, and differ in the origin of the 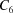 coefficients and whether higher-order terms (*i.e.* 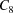 and/or 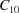 term, as in D3 and XDM) are included. Many-body vdW effects, which have been shown to be increasingly important for molecular materials (Reilly & Tkatchenko, 2015 ▸) including for polymorphism (Marom *et al.*, 2013 ▸), are also modelled by some methods, either using three-body Axilrod–Teller–Muto (Axilrod & Teller, 1943 ▸) contributions (D3), or a full many-body treatment using coupled atomic response functions (MBD). Most of these have been combined with the Perdew, Burke and Ernzerhof (PBE) semi-local density functional (Perdew *et al.*, 1996 ▸), with the TPSS (Tao *et al.*, 2003 ▸) and BLYP (Lee *et al.*, 1988 ▸; Becke, 1988 ▸) functionals also used. The two vdW density functionals feature an additional density-dependent term in the functional to approximate long-range or non-local correlation. See Table 2 ▸ and the supporting-information documents for details of the methods used by each submission.

The ranking methods mentioned above are normally used to estimate a lattice-energy difference between polymorphs. In reality, the relative thermodynamic stability of polymorphs is governed by free-energy differences, which include the contributions of zero-point and thermal motion to the enthalpy and entropy of the lattice, with configurational entropy also important in cases of disorder. Such contributions can affect the rank ordering of polymorphs (van Eijck *et al.*, 2001*b* ▸; Reilly & Tkatchenko, 2014 ▸; Nyman & Day, 2015 ▸). A number of methods have involved the use of lattice dynamics (Born & Huang, 1954 ▸; Dove, 1993 ▸) to estimate harmonic Helmholtz free energies. The effects of anharmonicity of the free energy have been captured using an extension of lattice dynamics (vibrational self-consistent field theory; Monserrat *et al.*, 2013 ▸), while molecular-dynamics (MD) simulations have been used to generate time- and ensemble-averaged structures and lattice energies at experimental temperatures and pressures. Finally, one submission considered kinetic aspects by ranking the structures generated based on the smallest critical-nucleus size determined from kinetic Monte Carlo simulations (Boerrigter *et al.*, 2004 ▸; Deij *et al.*, 2007 ▸). However, although crystallization conditions (*e.g.* solvent of crystallization) were provided as part of the blind test, none of the methods used this information as part of the CSP process.

### 3.4. Analysis and post-processing   

Many CSP methods involve analysis and post-processing of the structures generated. The nature of search algorithms frequently leads to the same structure being generated multiple times. In some approaches this is used as a measure or indication of the search completeness (Case *et al.*, 2016 ▸), but in all cases further calculations on duplicate structures waste computational resources. Many different approaches are used to detect and remove duplicates, ranging from packing-similarity analysis (discussed in §2.3), powder-pattern similarity (de Gelder *et al.*, 2001 ▸; Hofmann & Kuleshova, 2005 ▸), fingerprint functions (Oganov & Valle, 2009 ▸) and radial distribution functions (Verwer & Leusen, 1998 ▸). In some cases, structures that were very similar (*e.g.* structures with closely related hydrogen-bonding patterns or similar gross packings) were also removed, on the basis that such structures are unlikely to exist as distinct points or minima on the free-energy solid-form landscape. Filtering of results based on CSD informatics has also been used.

Post-processing of structures has been used to investigate the sensitivity of the results to the method used to rank them, *e.g.* to different repulsion–dispersion parameters, different quality wavefunctions or a polarizable continuum model for distributed multipoles and intramolecular energy contributions. As noted above, MD simulations and lattice-dynamics calculations can be used to provide finite-temperature estimates of relative stability of different structures. Such methods also provide an indication of the inherent finite-temperature and mechanical stability of the crystal structures generated. The crystal-adiabatic free-energy dynamics method (Yu & Tuckerman, 2011 ▸) was used to explore the stability and relations of structures in one submission.

### 3.5. Changes in the methodologies   

Comparing the present blind test with previous ones, we can see a number of changes in the approaches and methods employed. Firstly, there has been a change in the aims of some methods, which are not targeting an accurate prediction of the experimental crystal structure, but rather explicitly aiming to generate the experimental lattice somewhere within their low-energy structures. These results might then feed into other re-ranking approaches or analysis.

The protocols and workflows used by the different methods have also been developed and refined. Many approaches are now employing more exhaustive searches, considering more space groups, as well as larger regions of conformational space or a greater number of rigid conformations. In many instances, these expanded searches are guided by analysis of the results to inform on their completeness or sensitivity to levels of theory. This already feeds directly into the search process for some methods, while in others it is used to refine future searches (see individual supporting-information documents for more details).

One of the most significant changes is in the ranking methods employed. Solid-state DFT calculations have been used by 12 submissions, a significant increase compared with the fifth blind test, where only two submissions employed DFT. Many other submissions used more computationally demanding or bespoke potentials than in the past, with the use of generic empirical potentials and simple point-charge electrostatics as a final ranking method further declining to only a few submissions. In addition to focusing on better lattice energies, more methods are calculating free energies to rank the experimental structures at finite temperatures.

## 4. Results and discussion   

The sixth blind test has been the biggest to date: 25 distinct submissions were received, of which seven were full submissions, 14 attempted some of the targets, and four involved re-ranking structures generated using another method (by another team). This compares to 15 submissions in total in the previous blind test. Table 2 ▸ lists those who contributed to each submission along with a very brief summary of the methods employed, while Tables S10 and S11 in the supporting information provide a more detailed summary of the methods employed. The supporting-information document also contains details on access to computational data resulting from the blind test.

The overall results of the blind test are presented in Table 1 ▸, which lists for each system the number of attempts at prediction, the number of times the experimental structure was generated and the best ranking of that structure within the submitted lists. Table 3 ▸ provides the full results of each submission, broken down by target and the two lists. Tables showing the relative deviation between the lattice parameters of the predicted and experimental structures, as well as crystal and conformational RMSD values, are provided in the supporting information.

Given the number of submissions and large volume of data produced, an exhaustive account of the results is beyond the scope of this publication. Instead, we now focus on describing the experimental structures of the target systems and the trends and challenges in predicting and modelling them. A broad discussion of the results is then presented in §4.7.

### 4.1. Target (XXII)   

Tricyano-1,4-dithiino[*c*]-isothiazole (C_8_N_4_S_3_) was crystallized from an acetone:water mixture with X-ray diffraction data collected at 150 K (Horton & Gossel, 2016 ▸). The molecule crystallizes in the monoclinic 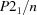 space group. In the experimental crystal structure the molecules form rows of molecules clasped together but offset from one another.

**Figure 1:**
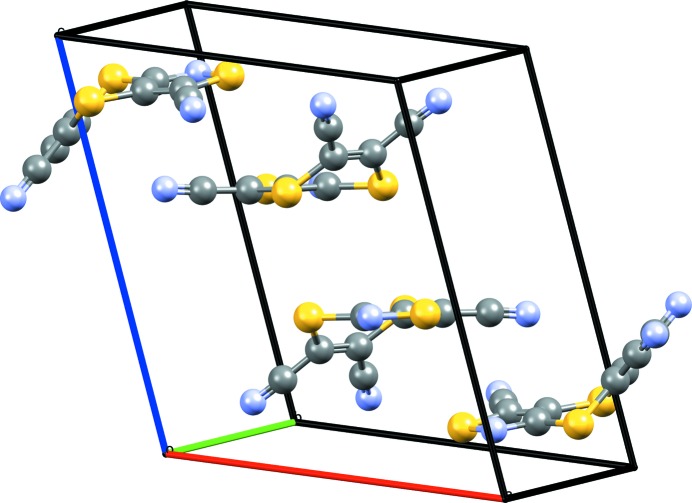
Experimental crystal structure of (XXII); C atoms are in grey, N in blue and S in yellow.

As Fig. 1 ▸ shows, the six-membered ring containing two S atoms is hinged, with an angle between the two C=C—S planes of 44.4°. This makes the molecule chiral, although calculations suggest the barrier to interconversion may be small. As communicated to participants, no chiral precursors were used during synthesis and therefore crystallization in a centrosymmetric space group is not unexpected. A search of the CSD (Version 5.37; Groom *et al.*, 2016 ▸; *R*-factor < 0.075; no errors, disorder or polymeric systems; organics only) for the six-membered dithiino ring, finds 77 structures that contain it, the majority of which feature the molecule in the hinged conformation with an angle between the two C=C—S planes of > 40°. Around 15 molecules have angles close to or at 0°, but many sit on a symmetry element such as an inversion centre, which can result in conformational bias (Cruz-Cabeza *et al.*, 2012 ▸).

Some force fields fail to adequately represent the hinge of this molecule, instead predicting that the molecule should be completely flat. Such a flat molecule is, as noted by a number of groups, a saddle point between the S atoms being above or below the mean plane of the molecule. Even some DFT methods have difficulty with the conformation of the molecule, which can be traced back to issues with the treatment of the S atoms in some vdW approaches. As a result, a number of submissions, even fully *ab initio* ones, featured crystal structures with flat or nearly flat molecules, although intermolecular interactions will also stabilize the planar conformation in some crystal structures.

Overall though, the experimental crystal structure was successfully generated and ranked by 12 out of 21 submissions, with all but one of those ranking the known experimental structure within the top eight most likely or stable structures and four ranking it as number one. A comparison of the predicted structures with the experimental one is given in Table S1. There is no definite trend in performance, with a range of treatments from generic potentials, point and multipole electrostatics, and DFAs ranking the experimental structure as being one of the most stable. Some of the other predicted structures are similar to the experimental one (for example, featuring a shift of the inversion centre), while others have more layered structures. Interestingly, many low-energy putative structures were found by multiple submissions. Solid-form screening of (XXII) may shed light on whether these predicted crystal structures could be isolated experimentally.

A number of second lists of predicted structures were submitted for (XXII) and three submissions re-ranked other structures, which gives an insight into the sensitivity of the ranking to the method employed. Three submissions (Podeszwa *et al.*, Szalewicz *et al.*, and Tuckerman, Szalewicz *et al.*) shared a set of potentials fitted to SAPT(DFT) calculations. Different functional forms for the potential, necessitated by the different software employed by the different methods, led to significantly different rankings for the experimental structure, while the ranking was sensitive to errors in the fitting procedure. Tkatchenko *et al.* re-ranked structures provided by Price *et al.* using the PBE+MBD functional, which improved the ranking compared with that with the FIT potential and multipole electrostatics. The second lists of Day *et al.*, Price *et al.* and Tkatchenko *et al.* all employed Helmholtz free energies, which changed the rank order of the putative structures and, in all three cases, improved the ranking of the experimentally known structure. In addition to free energies, two methods (Tuckerman, Szalewicz *et al.* and Podeszwa *et al.*) used MD simulations to obtain thermally averaged structures and potential energies at 300 K. The actual temperature of the diffraction experiment (150 K) was not disclosed to participants. These simulations confirm the stability of the experimental form on the potential-energy surface of the SAPT(DFT)-fitted potential. In post-test analysis, Marom *et al.* have also explored the rank ordering of low-energy structures of (XXII) using the PBE0 hybrid functional (Adamo & Barone, 1999 ▸) alongside different dispersion contributions.

### 4.2. Target (XXIII)   

2-((4-(3,4-Dichlorophenethyl)phenyl)amino)benzoic acid (C_21_H_17_Cl_2_N_1_O_2_) is a former drug candidate. (XXIII) targeted β-amyloid aggregation (Simons *et al.*, 2009 ▸; Augelli-Szafran *et al.*, 2002 ▸), which is believed to play an important role in Alzheimer’s disease. Five polymorphs of (XXIII) are known, three 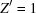 structures [forms *A* (Samas, 2016*a* ▸), *B* (Samas, 2016*b* ▸) and *D* (Samas, 2016*d* ▸)] and two 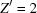 structures [forms *C* (Samas, 2016*c* ▸) and *E* (Samas, 2016*e* ▸)]. Forms *A* and *D* crystallize in the monoclinic 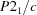 space group, while forms *B*, *C* and *E* crystallize as triclinic 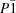 structures. Slurrying experiments have identified form *A* as being the most stable polymorph at 257 K, while at 293 K form D is the most stable polymorph (Samas, 2015 ▸).

**Figure 2:**
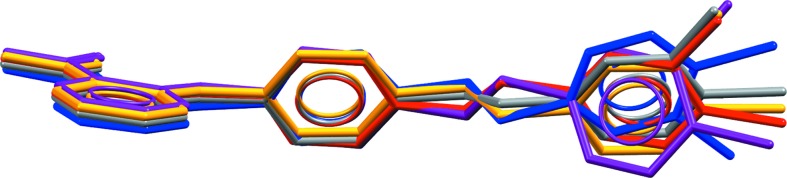
Molecular conformations found in forms *A*–*D* of (XXIII), overlaid onto the fenemate group of the molecule; form *A* is in blue, form *B* in grey, form *C* molecule 1 is in red, form C molecule 2 in purple and form *D* in orange. H atoms are omitted for clarity.

**Figure 3:**
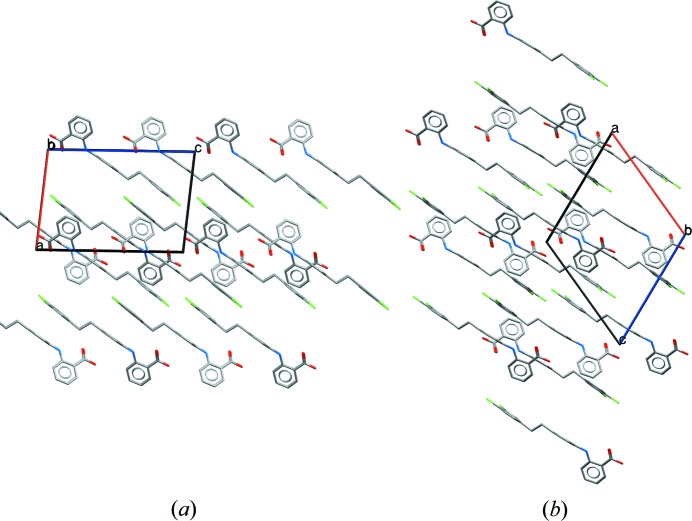
Crystal structures of (*a*) form *A* and (*b*) form *D* of (XXIII), showing the similar layers found in the two structures. H atoms are omitted for clarity.

All five polymorphs feature 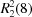 carboxylic acid hydrogen-bond dimers and intramolecular hydrogen bonds between the NH group and the carbonyl oxygen of the carboxylic acid, which is common in many fenamate structures. Fig. 2 ▸ shows the overlay of the conformations of (XXIII) in forms *A*–*D*. Forms *B* and *D* have a similar conformation, while form *A* has the chloro-phenyl ring flipped approximately 180° compared with *B* and *D*. The two molecules in the asymmetric unit of form *C* are similar, adopting the same torsions about the ethyl but differing in the twist of the phenyl group. The two molecules in form *E* (see Fig. S1) have distinct conformations from those found in forms *A*–*D*, with one molecule having the central phenyl ring rotated by approximately 120° compared with all of the other experimental conformations. Forms *B* and *C* have a similar gross packing, but deviate due to the two different conformations of the molecules in the asymmetric unit of form *C*. Forms *A* and *D* are also related in terms of their packing, featuring similar layers or sheets of molecules as seen in Fig. 3 ▸, again, differing only due to the different conformations of the end phenyl group. Given their close resemblance, interconversion of forms *A* and *D*, and forms *B* and *C*, respectively, might be expected to be facile but conversion of *A* or *D* to *B* or *C* might be much slower. Disorder might also be expected, with small energy barriers between some of the conformations.

The three 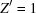 forms of (XXIII) were the main targets for this molecule, with 14 attempted predictions and three submissions re-ranking structures. Form *A* was generated four times in the top 100 structures, form *B* ten times and form *D* three times, with two methods (Day *et al.*; Neumann, Leusen, Kendrick) generating all three structures. In some cases the heavy-atom positions of the polymorphs were predicted, but not the correct ordering of the protons of the carboxylic acid dimer. These predictions are not counted in the totals above, as the proton environments are likely to be very different and distinguishable, but are denoted in parenthesis in Table 3 ▸.

The ranking of the experimental structures is more varied than for (XXII), with only a few of the predictions ranking the experimental structures as being one of the ten most stable structures, with form *A* having the best rank of 23 (Day *et al.*). A number of submissions predicted form *B* to be the most stable of the three 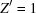 polymorphs, with a highest rank of 1 (Price *et al.*). In all of the experimentally observed conformations the molecule is extended. However, some of the low-energy predicted crystal structures have more compact conformations, with the terminal phenyl ring bending back towards the other end of the molecule. Such conformations could be favoured *in vacuo*, but not necessarily in solution or the solid state (Thompson & Day, 2014 ▸). Conformation and packing are the main differences between many of the predicted structures of (XXIII), as the CO_2_H dimer motif is found in the majority of low-energy structures.

As for (XXII), second lists and re-ranking submissions shed some light on the sensitivity of the results and methods. Price *et al.* predicted form *D* to be ranked 85th based on lattice energies from distributed multipoles and the FIT intermolecular potential. Re-ranking by Tkatchenko *et al.* placed the experimental structure as 14th in terms of lattice energy. Both submissions employed Helmholtz free energies (calculated at 300 K) in their second lists, which also significantly changed the polymorph rankings, and in the case of Tkatchenko *et al.* changed the relative ordering of the *B* and *D* polymorphs, improving the rank of *D* to second. Shifting through different levels of theory, from minimal basis-set Hartree–Fock theory to DFT (Brandenburg & Grimme, 2014 ▸), also altered Brandenburg & Grimme’s ranking of form *B* from number 11 to number 1.

Four attempts were made at predicting the 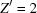 polymorphs. Form *C* was predicted by one method (Neumann, Kendrick and Leusen), ranking at number six in a list of both 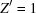 and 2 structures. The second 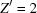 polymorph, form *E*, was not predicted by any submission. The potential disorder in the experimental structure might point to this being difficult to predict, but post-test analysis results suggest that most ranking methods have a valid local minimum corresponding to the experimental structure of form *E*, which means the structure should have been predictable with these methods.

Following the disclosure of the structures after the submission deadline, the experimental structures have been optimized and ranked using a number of different methods. The resulting calculated relative stabilities of the five polymorphs are presented in Table S12. Of the experimental structures, forms *B* and *C* are most often found to be the lowest-energy polymorph, although they are not generally found as the global minimum. This contrasts with the experimental stabilities from the slurrying data, where form *A* is most stable at 257 K and form *D* at 293 K. Directly comparing their rank or position on the energy landscape of each submission is difficult, as some methods may generate more or fewer local minima than others. This is demonstrated by the combined 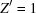 and 2 list of Neumann, Kendrick and Leusen, where some of the additional 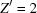 structures are lower in energy than some of the 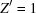 structures, making the ranks of the latter worse. However, post-test analysis does suggest that some of the more recent vdW-inclusive DFT methods (*e.g.* TPSS-D3 and PBE+MBD) would have ranked the experimental structures better, perhaps within the top 10–15 putative structures, if applied to a larger set of initial crystal structures or combined with different search methods.

### 4.3. Target (XXIV)   

**Figure 4:**
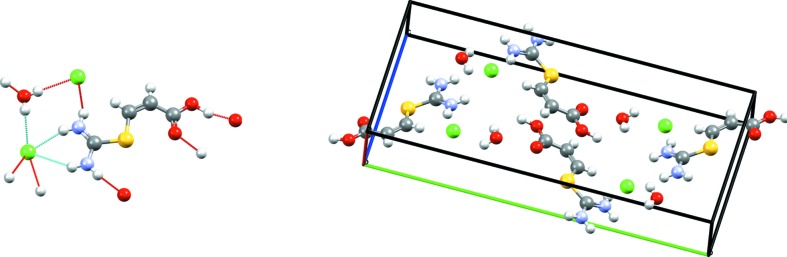
Experimental crystal structure of (XXIV) showing both the hydrogen bonds of the asymmetric unit and the unit cell; C atoms are in grey, H in white, O in red, N in blue, S in yellow and Cl in green.

Target (XXIV) is a chloride salt hydrate of (*Z*)-3-((diaminomethyl)thio)acrylic acid [(C_4_H_8_N_2_O^2^)^+^Cl^−^·H_2_O], which was crystallized in the monoclinic 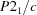 space group from a 1 *M* HCl solution, with the structure determined at 240 K (Foxman, 2016 ▸). The experimental crystal structure is shown in Fig. 4 ▸. Graph-set analysis (Etter *et al.*, 1990 ▸) yields over 25 distinct hydrogen-bond types. The Cl^−^ ions are six coordinate, with four short contacts and two longer ones, forming separate 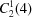 hydrogen-bond chains with thiouronium groups of the acid and water molecules. An 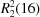 ring motif is also formed between carbonyl O atoms and the thiouronium groups of the acid molecules. As the molecule has a relatively flat conformation, the combination of the two motif types is to form interlocking layers or strands of acid molecules.

Of the eight full submissions for this target system, only the method of Neumann, Kendrick and Leusen generated the known experimental structure, ranking it as the second most stable structure with the PBE functional plus the Neumann–Perrin dispersion correction. Other structures in this and other submissions contain a large variety of different hydrogen-bonding patterns. The experimental hydrogen-bonding set is found in a few predictions, while some of the individual motifs (in particular, the 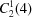 Cl^−^⋯water⋯Cl^−^ chains) are found in a number of structures generated by other methods.

As there are three components in the asymmetric unit, this is one of the most challenging target systems in the series of blind tests to date. This is both in terms of generating the complex hydrogen-bond patterns of the crystal structure and the demands of correctly ranking the strength of such interactions. Dealing with charged species, modelling charge penetration (Stone, 2013 ▸), capturing the coordination preferences of the Cl^−^ ion, and modelling polarization within the crystal are all serious challenges for empirical potentials. A number of submissions reported significant re-ordering of their predicted structures based on the type of Cl potential employed, and the dielectric constant used to model the effect of polarization on the electrostatic interactions in the crystal structures. Post-test analysis has borne this out, with some methods ranking the experimental structure more than 20 kJ mol^−1^ above the global minimum. Standard density-functional approximations can also struggle to deal with charged systems and charge transfer adequately due to self-interaction errors (Cohen *et al.*, 2008 ▸, 2012 ▸), but in the case of (XXIV), DFT provides a good basis for fitting a bespoke potential and ranking the predicted structures.

### 4.4. Target (XXV)   

**Figure 5:**
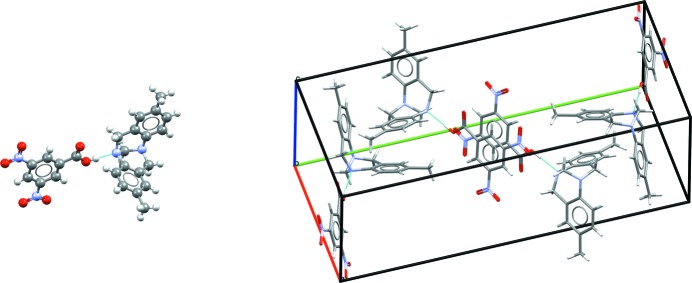
Experimental crystal structure of (XXV) at 300 K, showing the asymmetric unit and the unit cell; C atoms are in grey, H in white, O in red and N in blue. The proton is shown as originally refined at 300 K, attached to the carboxylic acid. Close analysis of the data and further data collected at 100 K suggest that a disordered structure with the H atom occupying two sites is more representative.

(XXV) is a multi-component system consisting of 3,5-dinitrobenzoic acid (C_7_H_4_N_2_O_6_) and 2,8-dimethyl-6*H*,12*H*-5,11-methanodibenzo[*b*,*f*][1,5]diazocine (C_17_H_18_N_2_), also known as Tröger’s base. The N atoms of Tröger’s base are unable to invert and therefore the molecule is chiral, but the structure was crystallized from a methanol solution that contained both enantiomers. X-ray diffraction data were initially collected at 300 K (Wheeler & Breen, 2016*a* ▸). The two components crystallize in the monoclinic 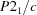 space group, with the asymmetric unit and unit cell shown in Fig. 5 ▸. Both molecules in the structure adopt their expected conformation, with only a slight tilting of the NO_2_ groups of the acid. The position of the H atom between the two co-formers was determined from a Fourier difference map, which shows that the proton is mostly located on the O atom, forming a co-crystal. Experimental data collected at 100 K after the blind test had concluded, show more clearly that the system is disordered with a two-site refinement suggesting the proton occupancy on the O atom is 0.58 (3) and that on the N atom is 0.42 (3) (Wheeler & Breen, 2016*b* ▸). More variable-temperature studies and neutron diffraction may resolve whether the proton disorder is a dynamic, temperature-related effect. In a few experimental structures of Tröger’s base derivatives, the N atoms appear to be clearly protonated, forming salts rather than co-crystals (see, for example, CSD refcodes: LEMBEL, CUNQAE), while neutral hydrogen bonds are observed in other structures such as PECDIM and PIPXAP.

In total, 14 attempted predictions were made for (XXV), with five groups generating the experimental structure and two re-ranking submissions also ranking the experimental structure within their list of 100 structures. All of these predicted a co-crystal, with no isostructural salt being found in any submissions. Once generated, (XXV) has generally been ranked as one of the most stable structures in the predicted landscape, with three predictions (van Eijck; Pantelides, Adjiman *et al.*; Price *et al.*) ranking it as being the most stable structure, and the worst rank being sixth. The re-ranking submissions of Brandenburg & Grimme, and Tkatchenko *et al.* ranked it as being the second-most or most stable structure, respectively.

The proton position in (XXV) is a significant challenge both for theory and experiment. As (XXV) was stated to be a co-crystal in the blind-test announcement, it is expected and understandable that no method explored the proton position explicitly, and for a number of methods the protonation state is fixed on the basis of the information given and cannot vary during the CSP calculation. Had the disorder been known in advance, it is likely that many methods would have been adapted as well, perhaps employing multiple searches with both neutral and charged co-formers and the potential parameters or ‘typing’ used for the N and O atoms would have been varied or explored, all of which could affect the results of the prediction (Mohamed *et al.*, 2011 ▸). Three methods (Facelli *et al.*; Neumann, Kendrick and Leusen; Zhu, Oganov, Masunov) did predict a non-isostructural salt form as being the most stable form for (XXV), although the latter two submissions do rank the experimental form as being one of the most stable structures. The prediction of a salt form for (XXV) is possible due to their use of DFT in the final ranking stage, which allows for proton migration and transfer to occur, although only if there is no barrier for this with the DFA used. Many of the other methods that use DFAs also predicted salt structures somewhere in their submitted lists.

While the disorder in (XXV) was an unexpected complication, it highlights the ongoing challenges of modelling proton positions and disorder. Salts and co-crystals are often considered distinct types of solid forms, but (XXV) also demonstrates the fine line between the two and the challenges of predicting or even characterizing them.

### 4.5. Target (XXVI)   

*N*,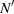-([1,1′-Binaphthalene]-2,2′-diyl)bis(2-chlorobenzamide) (C_34_H_22_C_12_N_2_O_2_) was crystallized from a 1:1 mixture of hexane and dichloromethane in the triclinic 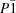 space group, with data collected at room temperature (Wheeler & Hopkins, 2016 ▸). This crystal structure was the original target for this molecule and is referred to as form 1. Polymorph screening (Sharp *et al.*, 2016 ▸) found that form 1 undergoes a phase transition to another polymorph at around 428 K. This high-temperature polymorph is known as form 11 and has been characterized using high-resolution powder diffraction, with structure solution on-going (Sharp *et al.*, 2016 ▸). The polymorph screen also found nine solvates of (XXVI) (known as forms 2–10).

**Figure 6:**
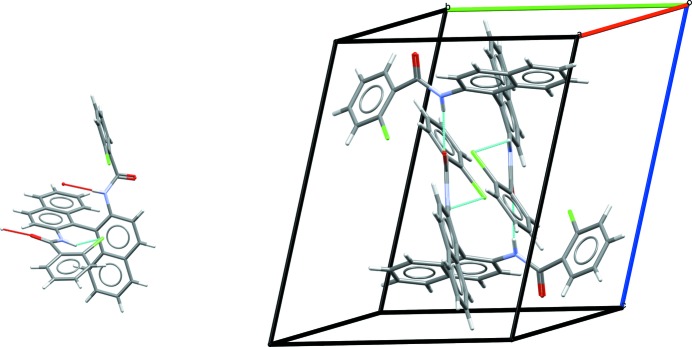
Experimental crystal structure of (XXVI), showing the molecular conformation and the unit cell, with hydrogen bonds shown by blue lines; C atoms are in grey, H in white, O in red, Cl in green and N in blue.

Compounds containing the 1,1′-binaphthalene fragment can feature axial chirality, however, no chiral precursors were used in the synthesis of (XXVI). While the category for this target stated that the experimental crystal structure was 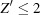, the experimental structure for form 1 is 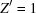, with one molecule in the asymmetric unit. In the crystal structure, shown in Fig. 6 ▸, the two molecules in the unit cell form an 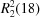 dimer. There is also a close contact within the molecule between the Cl and an amide hydrogen on one of the two amide groups in the molecule. One of the two amide O atoms in the molecule is unsatisfied in terms of hydrogen bonds. As noted by a number of groups, the bulky binaphthalene and phenyl groups may well cause frustration in the molecular conformation, leading to difficulty in forming a more extensive intermolecular hydrogen-bond network, although intramolecular NH⋯O hydrogen bonds might be observed. Comparing the experimental intramolecular geometry to CSD-derived angle and torsion distributions (using *Mogul*; Bruno *et al.*, 2004 ▸) suggests that the angle and torsions between the amide group and phenyl ring that are involved in both hydrogen bonds are unusual compared with expected CSD values.

There were 12 attempted predictions for molecule (XXVI), five of which explicitly considered the possibility of the experimental structure being 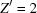. Three methods (Elking & Fusti-Molnar, Neumann, Kendrick and Leusen, and Price *et al.*) generated the experimental structure of form 1. All three submissions ranked form 1 as being the most stable polymorph in at least one of their two lists. For one submission (Elking & Fusti-Molnar), form 1 was ranked as number eight by an empirical potential, with DFT (PBE+XDM) improving the ranking to be number one in the second list. A comparison of the experimental structure of form 1 with the correction predictions is given in Table S8.

In many of the submissions, high-ranking structures (*e.g.* within the ten highest ranked predictions) do not feature intermolecular hydrogen bonds and conversely in some cases low packing coefficients are reported. This reflects the difficulty the molecule has in forming stable close-packed structures and intermolecular hydrogen bonds simultaneously and perhaps tallies with the preponderance of solvates in the experimental solid-form screen. For a number of methods, the failure to generate the form 1 structure can be attributed to difficulties in generating the experimental conformation due to its distorted nature. This posed a significant difficulty for searches employing rigid conformations, but even with flexibility permitted some methods would have needed more exhaustive searches to generate the correct conformation.

### 4.6. Computational resources   

As in previous blind tests, participants were asked to include a brief summary of the computational resources and hardware used to carry out their predictions. Directly comparing these data is difficult not only due to the different CPUs used but also the wide range of architectures employed, ranging from standard desktop PCs to massively parallel machines at national supercomputing facilities. As a result the data have not been normalized. A summary of each submission’s usage is provided in Table S9, with more details available in each submission’s supporting-information document.

In general, the resources employed for predictions have increased significantly since the last blind test, with 13 submissions employing more than 100 000 CPU hours, compared to four in the fifth blind test. This is partly due to the increased use of more sophisticated ranking and refinement methods (such as DFT, tailor-made force fields and flexible multipoles) and partly due to more detailed and demanding searches of the conformational and structural landscapes of the targets, increasing the number of putative structures. A number of the full submissions that targeted all five systems employed over 500 000 CPU hours. For a single target, 100 000 CPU hours would amount to approximately 16 d elapsed time on a 256-core machine, representing a substantial investment of computational resources and time. Nevertheless, the increased importance and potential of computational modelling in general means that such computational resources are more widely available in both academia and industry, and further advancements and optimization in algorithms and software might well yield significant reductions in computational costs.

However, as in previous blind tests, there is a significant disparity in the amount of computational resources employed in obtaining a successful prediction. For (XXII), a number of successful predictions employed 10 000–30 000 CPU hours, while a few submissions predicted the known experimental structure with less than 200 CPU hours, using comparatively simple empirical potentials and, at most, rigid multipole electrostatics. Conversely, a number of full DFT/*ab initio* submissions for (XXII) failed to predict the experimental structure, despite using orders of magnitude more computational resources. A few methods generated some of the experimental structures of (XXIII) and (XXV) with a fraction of the CPU resources of other approaches and in some cases comparable ranking. This disparity suggests that there remains considerable scope to improve our understanding of where simple potentials are sufficient for some or all of the CSP calculation, where instead bespoke potentials and *ab initio* information and calculations must be used, and where optimizations and improvements in algorithms are possible.

As a final point, it is worth noting that as computational resources become more widely available and cheaper, the personnel cost of the methods becomes more important. This too likely varies significantly between the different methods and approaches to the problem. Whereas ranking is the most time-consuming process from a computational perspective, conformational analysis and interpretation of the CSP results are likely the most demanding parts of the calculation in terms of human resources.

### 4.7. Performance and progress of crystal structure prediction methods   

The performance and ‘success’ of a CSP calculation is naturally first assessed in terms of whether experimental structures are generated by the calculation and where they are placed on the putative crystal-structure landscape. Generation relies on the experimental structure corresponding to a local minimum of the fitness function (or potential-energy surface) used. All the experimental structures in the sixth blind test, apart from the potentially disordered form *E* of (XXIII), were generated by one or more methods and submissions, with one method (Neumann, Kendrick and Leusen) generating all of them [apart from (XXIII) *E*].

While all of the structures have been generated, their ranking and placement on the predicted landscapes is more variable. (XXII), form *B* of (XXIII), (XXV) and (XXVI) were ranked as the lowest-energy, most-stable putative structure by a few methods but not consistently by a single method. This inconsistency may be explained, in part, by the possibility that some higher-ranked predicted structures might correspond to undiscovered experimental forms of (XXII), (XXIV) and (XXV), which have not been subject to extensive solid-form screening.

**Figure 7:**
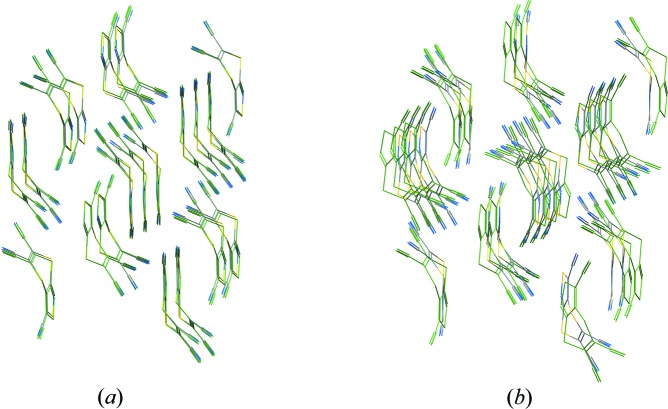
Two example overlays of the experimental crystal structure of (XXII) with predicted structures of (*a*) Tkatchenko *et al.* with an RMSD of 0.166 Å, and (*b*) Obata & Goto with an RMSD of 0.808 Å. The predicted structures are shown in green for clarity. With the smaller RMSD in (*a*) the two structures are difficult to distinguish visually, while for the larger RMSD in (*b*) the predicted and experimental molecules are clearly offset.

The extent to which experimental structures have been reproduced in terms of the crystal structure is also variable. One measure of this is the RMSD between clusters from the experimental and predicted crystal structures, with example structure overlays for (XXII) shown in Fig. 7 ▸ (see Tables S1–S8 and §2.3 for more values and details, respectively). The values for this blind test are comparable to those in the previous one, although some are relatively large at ∼ 0.8 Å. The RMSD value is often a combination of deviations in the gross packing and conformation, and therefore expected values may vary depending on the conformational flexibility of a molecule and the degree to which flexibility was permitted in the CSP calculation. In general, the smallest RMSD values are found for methods using DFAs for the final optimization and ranking step. However, it is worth remembering that experimental structures feature thermal-expansion effects, whereas the majority of the CSP methods are predicting 0 K ‘equilibrium’ geometries. MD simulations, which have been used by two submissions (Podeszwa *et al.* and Tuckerman, Szalewicz *et al.*), should capture these effects and provide better comparison with experiment. Such simulations require the temperature of the diffraction experiment as input though, which was not disclosed to participants. For (XXII), MD simulations at 300 K gave an RMSD_20_ of 0.187 Å (Tuckerman, Szalewicz *et al.*), but a post-test MD simulation at the experimental temperature of 150 K, gives a value of 0.140 Å, which is smaller than any of the RMSD values for the submitted structures. This demonstrates the significant contribution of thermal and zero-point motion to RMSDs. Although zero-point motion would not be expected to influence ranking and RMSD values in molecules such as target (XXII), which contains all heavy atoms, in general, this is a factor that needs to be carefully considered.

To understand how the field has progressed and developed we can compare the sixth blind test with the previous fifth one. In that test the targets were generated and ranked within the top 100 structures between three and five times with typically 10–15 submissions (Bardwell *et al.*, 2011 ▸), leading to around 24 out of 68 predictions generating the experimental structure, although it should be noted that the criteria in the fifth blind test considered only the top-three predicted structures as a success and not all submissions provided extended lists of structures. In the present blind test, 36 predictions out of 70 (for 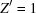 structures) generated the experimental structure. Some systems have been generated by a number of methods, *e.g.* 10 of 14 submissions generating or ranking (XXIII) form *B*, while only one method predicted the experimental structure of (XXIV) and none predicted (XXIII) *E*.

However, a key difference and development is the nature of the target molecules, which represent a significantly increased challenge. (XXIV) is the first three-component and salt–hydrate system, with both salts and hydrates having proven difficult individually in the previous blind test (Bardwell *et al.*, 2011 ▸). (XXVI) is the largest molecule attempted in a blind test to date, while the polymorphic nature of (XXIII), its intramolecular flexibility and two 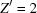 forms makes it a serious challenge and test for methods as well, and (XXII) cannot be considered a strictly rigid molecule.

In this sense, the current blind test shows the advancement in the capabilities of CSP methods in the five years since the last test, and the broadening of their applicability to new types of solid forms and more complex molecules. While many challenges remain, as will be discussed below, the wide range of methods, many of them applied for the first time in this blind test, does bode well for the CSP in the future. There is a wealth of information in the submissions that points to new and continuing developments, as post-test analysis has already begun to show. Another important aspect of the development of CSP methods is the establishment of more well defined protocols and ‘best practice’ guidelines for performing the calculations, which will be further developed in light of the results of this blind test.

### 4.8. Challenges in CSP methods   

The sixth blind test highlights the continuing development of CSP methods but also the challenges they face. The first of these is in the initial generation of the experimental crystal structure. In many cases where methods failed this can be traced back to issues in generating the experimental conformation, either due to the search using rigid conformations significantly different from those in the experimentally observed forms or not considering a wide enough search space in flexible CSP calculations, which was seen in particular for (XXVI). In other cases, assumptions or limits placed on the search space or possible intermolecular interactions prevented the search from finding the observed crystal structure, or the search was simply not exhaustive enough. Experimental structures were initially generated by some search algorithms but not ranked highly by the intermediate optimization and ranking methods, and therefore not brought forward to the final stages where these missing structures could have ranked highly. Encouragingly, post-test analysis has suggested a number of adjustments and refinements to different methods that should limit or prevent these issues in future.

The final, definitive ranking of the predicted structures remains a long-standing issue for CSP methods. The majority of methods based their final rankings on differences in static, 0 K lattice energies. DFT is emerging as a leading method for calculating these differences, being used by 12 CSP methods in this blind test. However, a number of benchmark studies of density-functional approximations and models of vdW interactions (Otero-de-la-Roza & Johnson, 2012 ▸; Reilly & Tkatchenko, 2013 ▸; Moellmann & Grimme, 2014 ▸) suggest accuracies of 3–4 kJ mol^−1^ for absolute lattice energies, while one of the most sophisticated quantum-chemical calculations of the lattice energy of benzene is accurate to only 1 kJ mol^−1^ (Yang *et al.*, 2014 ▸). Given the small energy differences needed to resolve some polymorphs (Price, 2009 ▸), accuracies of lattice-energy differences therefore may still involve fortuitous cancellation of errors, which is not assured with so many different types of interactions, conformations and packing arrangements possible. Post-test analysis of the (XXIII) polymorphs (Table S12) highlights the differences between ranking methods with a range of different relative orderings and absolute differences.

Many of the benchmark DFT studies have pointed towards ways of improving accuracy and transferability, including many-body vdW interactions (Risthaus & Grimme, 2013 ▸; Tkatchenko *et al.*, 2012 ▸) and the use of hybrid and *meta*-GGA density functionals (Reilly & Tkatchenko, 2013 ▸; Moellmann & Grimme, 2014 ▸). Affordable periodic quantum-chemical calculations are also emerging (Wen & Beran, 2011 ▸; Bygrave *et al.*, 2012 ▸; Del Ben *et al.*, 2012 ▸), and are already providing insights into polymorphism (Wen & Beran, 2012*a* ▸,*b* ▸; Bygrave *et al.*, 2012 ▸). The cost of *ab initio* calculations is a related issue for ranking, with less-intensive intermediate ranking methods still important for making CSP calculations tractable. The decline in the use of generic empirical potentials points to the need for better potentials to be developed or wider use of bespoke potentials based on first-principles methods, such as DFT (*e.g.* Neumann *et al.*, 2008 ▸; Grimme, 2014 ▸) or SAPT(DFT) (Misquitta *et al.*, 2005 ▸). Such intermediate methods may lead to more confidence in selecting the final set of structures for optimization and ranking with more expensive methods.

After considering static lattice energies, it is important to remember the contributions of vibrations, disorder and, if it is an experimental variable, pressure to the free-energy differences of crystal structures. Vibrational contributions can be readily estimated in the harmonic limit using lattice dynamics (Born & Huang, 1954 ▸; Dove, 1993 ▸), and have been used as part of a number of methods in this blind test and shown to affect rankings of a number of systems and the ordering of the polymorphs of (XXIII). However, such calculations neglect the contributions of anharmonic vibrations and thermal expansion, the role of which in polymorph free-energy differences is not well understood. Wider use of anharmonic lattice dynamics (Monserrat *et al.*, 2013 ▸) and MD simulations may shed more light on this. Configurational disorder can also be modelled, for example using ensemble approaches (Habgood *et al.*, 2011 ▸) or approaches based on Monte Carlo and substitution methods (Neumann *et al.*, 2015 ▸). However, the cost of all of these calculations is substantial, often more than an order of magnitude more than the initial geometry optimization (see the supporting-information documents of a number of submissions), making a fully consistent estimate of thermodynamic ordering very computationally demanding and challenging. Given the small energy differences observed between some low-energy structures in this blind test, it may become more important to include these contributions in future.

Beyond thermodynamics, there remains the fundamental role of kinetics in determining the experimentally observed or accessible solid forms (Threlfall, 2003 ▸; Blagden & Davey, 2003 ▸; Price, 2013 ▸). Some thermodynamically stable solid forms may be slow to nucleate, for example, due to the required molecular conformation being unstable in the crystallization solution, while metastable solid forms favoured by the fastest pathway to crystallization may be slow to revert to other forms. The similarities between some of the forms of (XXIII) and significant differences between others suggests that the balance between kinetics and thermodynamics might well be important for (XXIII). Only one method in the present blind test explicitly considered kinetics (using kinetic Monte Carlo simulations to determine critical-nucleus sizes), and no submission took account of the crystallization conditions supplied. There have been many advances in the modelling of nucleation (Anwar & Zahn, 2011 ▸) and crystal growth (Piana *et al.*, 2005 ▸; Salvalaglio *et al.*, 2012 ▸), but again these are involved and computationally demanding simulations, mostly limited, to date, to considering model systems, with relatively generic empirical potentials.

While direct modelling of kinetics is not routine, some CSP methods do involve considering differences between predicted structures, with the aim of rationalizing whether they would amount to distinct solid forms that would be expected to crystallize separately (Price, 2013 ▸, 2014 ▸). Structural informatics based on experimental crystal structures, such as hydrogen-bond propensities (Galek *et al.*, 2007 ▸, 2009 ▸), could also be used to assess the experimental likelihood of features in predicted structures. Approaches such as these may provide a bridge between the thermodynamic ranking produced by CSP calculations and the more demanding investigations of how kinetics affect the final solid form(s) of a molecule.

### 4.9. Beyond predicting ‘the’ crystal structure   

While significant challenges remain for routine and definitive prediction of the stable solid forms of organic molecules, this is not always the true aim of performing CSP calculations, which are emerging as a general tool to complement experimental studies of organic solid forms. On a fundamental level, CSP calculations represent one of the most demanding challenges of the reliability of empirical potentials and first-principles methods. Their role in providing information for solving or confirming crystal structures from powder X-ray diffraction data is now well established, and they can also aid alternative structure-characterization methods, such as NMR or electron diffraction (Baias *et al.*, 2013 ▸; Eddleston *et al.*, 2013 ▸).

CSP calculations also have a significant role in understanding the potential solid forms of a molecule. This has been demonstrated by a number of studies combining CSP calculations with experimental solid-form screening, as has already been noted in §1, and the sixth blind test further illustrates this. For (XXV), some of the submitted lists show large gaps in terms of energy between the lowest-energy structure and other putative structures. For other systems, the results show a range of structures close to the global minimum, which is more indicative of potential polymorphism. The experimental form of (XXII) was predicted by 12 out of 21 submissions, but a number of the other structures predicted as being low in energy were found in multiple submissions. While the exact predictions of the experimental structures are not always correct, these observations might help guide where more experiments, *e.g.* solid-form screening, are more likely to be needed. Indeed, it is worth remembering that the practical use of CSP calculations is unlikely to be ‘blind’, with either structures known experimentally or the difficulty of crystallization having been established. A CSP calculation that predicts many possible putative structures competitive with an experimentally observed form, as seen for all of the submissions for (XXIII), would suggest more experimental studies as being advisable.

Beyond guiding experiment directly, the landscapes or sets of putative crystal structures can inform on the general behaviour of a target molecule. For a number of submissions, low-energy predictions that do not match the experimental structures are nevertheless closely related to them, with a number of the unsuccessful submissions for (XXII) predicting structures that matched the experimental form with 14 out of 20 molecules. Such structures might well have similar properties to the observed solid form. In other cases, the submissions show how CSP enables one to explore the general ability of a molecule to pack with itself. A number of submissions for (XXVI) show the distorted nature of the molecular conformation and the difficulty the molecule has in forming extended hydrogen-bonding networks. Low packing coefficients are also reported, correlating well with the experimental observation of nine solvates in solid-form screening.

In the context of these wider applications of CSP methods, the ‘success’ of a CSP calculation can only be measured in terms of its specific goals and aims, which will rarely mean a completely blind prediction. These types of applications of CSP methods will require not only developments in the methods themselves but clear protocols for analysing the putative structures generated, as well as a greater understanding of how to turn information on possible or putative structures into new experiments and ultimately new solid forms. This will no doubt be one focus of ongoing research in CSP methods and future blind tests might well reflect this in the choice of target systems and goals.

## 5. Conclusion   

The sixth blind test of organic CSP methods has been the biggest to date, with 21 submissions attempting to predict one or more of the five target systems, and four submissions re-ranking other predictions with different methods. The range of methods and approaches show the development of the field, with progress in the treatment of conformational flexibility in molecules, wider use of *ab initio* or *ab initio*-based methods for optimizing and ranking the final structures, as well as more well defined and systematic protocols for performing CSP calculations.

Apart from the potentially disordered form *E* of (XXIII), all of the experimental crystal structures of the five targets were predicted by one or more submissions, with one method based on Monte Carlo parallel tempering for structure generation and final ranking with DFT (Neumann, Kendrick and Leusen) generating all of them. While the rate of success is comparable to the previous blind test, the target systems are significantly more challenging, and include a polymorphic former drug candidate, a three-component chloride salt hydrate and a bulky flexible molecule that is the largest attempted in a blind test to date. In this context, we conclude that state-of-the-art CSP calculations are now applicable to a wider range of solid forms, such as salts and hydrates, as well as larger more flexible molecules.

However, significant challenges remain for routine and reliable CSP calculations. One source of difficulties in generating structures was the conformational flexibility and preferences of the targets. For (XXII), force fields and even some density-functional approximations had difficulty with the hinged nature of the molecule, while searches with rigid conformations had difficulties for (XXIII) and (XXVI). Encouragingly, post-test analysis of the results has already suggested a number of refinements to the CSP workflows used in the submissions.

The definitive ranking of the predicted crystal structures remains difficult and computationally expensive. While the experimental structures of many of the targets were ranked as being the most stable or one of the most stable predicted crystal structures, no method consistently ranked all of the experimental structures, as (XXIII) highlights. Post-test analysis again suggests that state-of-the-art density-functional approximations could improve upon the submitted results and ongoing developments in *ab initio* and DFT methods, algorithms and the use of bespoke force fields bode well. As ranking based on lattice energies improves, considering additional contributions such as entropy will be more important, with this blind test also seeing an increase in the number of submissions ranking structures based on Helmholtz free energies.

Overall, the results of this blind test have demonstrated the increased maturity of CSP methods. They have also illustrated the role for CSP calculations to guide and complement our understanding and experimental studies of organic solid forms. This is likely to be an important focus for the application and development of CSP methods moving forward.

## Supplementary Material

Supporting tables and figures. DOI: 10.1107/S2052520616007447/gp5080sup1.pdf

Prediction of crystal structures (and related data) submitted by participants in the blind test.. DOI: 10.1107/S2052520616007447/gp5080sup2.zip

Supporting information files for each submission in the blind test. DOI: 10.1107/S2052520616007447/gp5080sup3.zip

## Figures and Tables

**Table 1:** Two-dimensional chemical diagrams, crystallization conditions for the five target systems in the sixth blind test, including information disclosed to participants initially and following queries, as well as a summary of the full predictions for each target system Separate lists and re-ranking submissions are not counted in these totals, but the best rank given does include re-ranking attempts. See §2.1 for more details of the categories.

Target	Chemical diagram	Crystallization conditions, remarks and clarifications	Attempted predictions	Times generated	Best rank (incl. re-ranking)
(XXII)	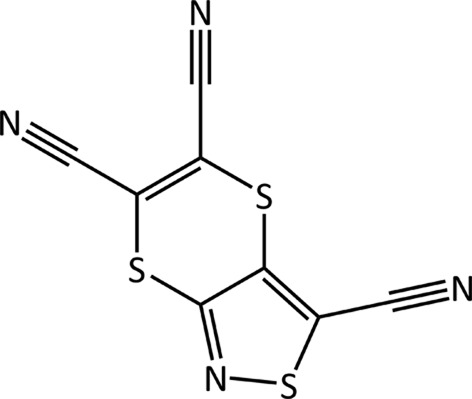	Crystallized from an acetone/water mixture; chiral-like character due to potential flexibility of the six-membered ring, but no chiral precursors used in synthesis.	21	12	1
(XXIII)	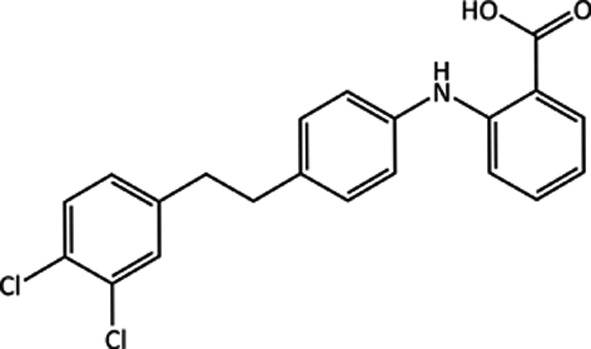	Five known polymorphs (*A*–*E*); three 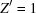 (*A*, *B*, *D*), two 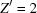 (*C* and *E*). The most stable polymorphs at 257 and 293 K are both 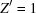 . Crystallization conditions include slow evaporation of acetone solution and of ethyl acetate:water mixture.	*A*, *B* and *D*: 14; *C* and *E*: 3	*A*: 4, *B*: 8, *C*: 1, *D*: 3, *E*: 0	*A*: 23, *B*: 1, *C*: 6, *D*: 2, *E*: –
(XXIV)	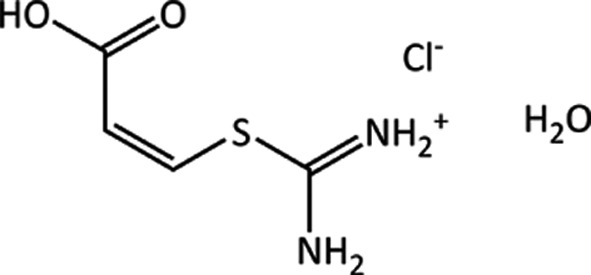	Crystallized from 1 *M* HCl solution. The substituents of the C=C double bond are in the *cis* configuration.	8	1	2
(XXV)	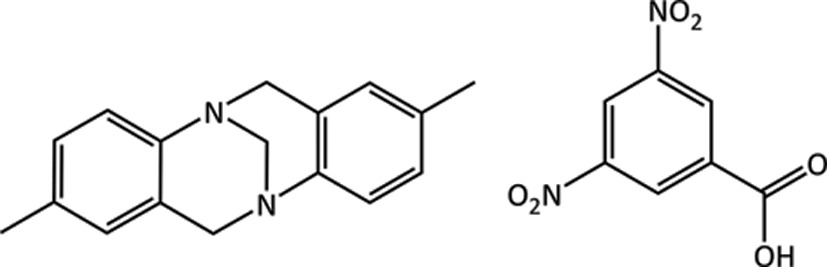	Slow evaporation of a methanol solution, which contained a racemic mixture of the enantiomers of Tröger’s base.	14	5	1
(XXVI)	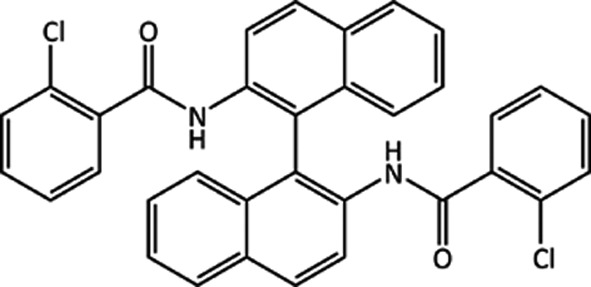	Slow evaporation from 1:1 mixture of hexane and dichloromethane. No chiral precursors used in synthesis.	12	3	1

**Table 2:** List of members of each team/submission (* denotes corresponding author), as well as a *brief* summary of the generation and ranking methods used Please refer to §3 for an overview of the methods, Tables S10 and S11 of the supporting information, and each submission’s supporting-information document for more details. Helmholtz free-energy contributions are denoted by 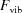, polarizable continuum model is abbreviated PCM, while Monte Carlo is abbreviated MC.

			Final ranking method(s)	
Team	Members	Generation method	List One (L1)	List Two (L2)
1	Chadha,* Singh	MC simulated annealing	COMPASS (2.8) force field	–
2	Cole,* McCabe, Read, Reilly, Shields	CSD analogues	Fitted exp-6 potential	–
3	Day*, Bygrave, Campbell, Case, Gee, McMahon, Nyman, Pulido, Taylor, Yang	Quasi-random search (Sobol’)	Atomic multipoles and exp-6	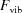 contributions [(XXII) and (XXV)], PCM 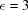 [(XXIV) and (XXVI)]
4	Dzyabchenko	Grid search	Empirical potential	–
5	van Eijck	Random search	Atomic charges, intramolecular 6-31G** energies and exp-6	–
6	Elking, Fusti-Molnar	Random generation	Empirical potential	PBE+XDM
7	de Jong, van den Ende,* de Gelder, de Klerk, Bylsma, de Wijs, Meekes, Cuppen	Random search	*q*-GRID method	Smallest critical nucleus size from kinetic MC simulations
8	Lund, Pagola, Orendt, Ferraro, Facelli*	Genetic algorithm	PBE-D2	PBE-D2 for all stages of GA search
9	Obata, Goto*	Grid search	PBE+TS	–
10	Hofmann,* Kuleshova	Random search	Fitted potential	–
11	Lv, Wang, Ma*	Random search	optB86b-vdW	–
12	Curtis, Li, Schober, Cosburn, Lohani, Vacarro, Oberhofer, Reuter, Bhattacharya, Vázquez-Mayagoitia, Ghiringhelli, Marom*	Genetic algorithm	PBE+TS	PBE+MBD
13	Mohamed	MC simulated annealing	Atomic multipoles and exp-6	–
14	Neumann, Kendrick, Leusen	MC parallel tempering	PBE+Neumann–Perrin	Includes 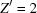 structures for (XXIII) and (XXVI)
15	Sugden, Gatsiou, Vasileiadis, Adjiman,* Pantelides*	Quasi-random search (Sobol’)	Atomic multipoles and exp-6	–
16	Pickard,* Monserrat, Misquitta, Needs	Random search	PBE+MBD	–
17	Jankiewicz, Metz, Podeszwa,* Szalewicz	Grid search	SAPT(DFT) fitted potential	Alternative SAPT(DFT) fitted potential
18	S. L. Price,* Hylton, L. S. Price, Guo, Watson, Iuzzolino	Quasi-random search (Sobol’)	Atomic multipoles and exp-6	Different PCM treatments (all); 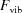 for all but (XXIV)
19	Metz, Hylton, S. L. Price, Szalewicz*	Quasi-random search (Sobol’)	SAPT(DFT) fitted potential	–
20	Vogt, Schneider, Metz, Tuckerman,* Szalewicz*	Random search	SAPT(DFT) fitted potential	–
21	Zhu,* Oganov, Masunov	Evolutionary algorithm	vdW-DF	–
				
22	Boese	Re-ranking 10	PBE+TS and BLYP-D3	–
23	Brandenburg, Grimme	Re-ranking 18	HF-3c^atm^	TPSS-D3^atm^
24	Metz, Guo, Szalewicz	Re-ranking 18	SAPT(DFT) fitted potential	–
25	Hoja, Ko, Car, DiStasio Jr, Tkatchenko*	Re-ranking 18	PBE+MBD	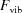 contributions

**Table 3:** Results of each submission in the sixth blind test, broken down by target system and the two lists (L1 and L2; *cf.* Table 2 ▸) that could be submitted Numbers indicate the position in the submitted list at which an experimental structure was found, a dash (–) indicates that the experimental structure was not found in the submitted predicted structures, and a blank entry indicates no prediction was attempted. For re-ranking submissions, an asterisk (*) indicates that the experimental structure was not present in the set of re-ranked structures. For (XXIII) *C* and *E*, only submissions that explicitly considered 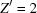 searches are noted in the table. Numbers in parentheses for (XXIII) indicate that the heavy-atom positions were predicted, but not the correct position of the H atom of the carboxylic acid.

		(XXII)		(XXIII)										(XXIV)		(XXV)		(XXVI)	
				A		B		C		D		E							
Team	Members	L1	L2	L1	L2	L1	L2	L1	L2	L1	L2	L1	L2	L1	L2	L1	L2	L1	L2
1	Chadha & Singh	–		–		–				–								–	
2	Cole *et al.*	–		–		–		–		–		–				–		–	
3	Day *et al.*	3	1	23	–	–	75			75	–			–	–	–	–	–	–
4	Dzyabchenko	1														–		–	
5	van Eijck	4		83		20				–				–		1		–	
6	Elking & Fusti-Molnar	–	–	–	–	78	–			(73)	–			–	–	–	–	8	1
7	van den Ende, Cuppen *et al.*	9	90	–	–	–	–			–	–					–	–		
8	Facelli *et al.*	–	–	–		–				–				–		–			
9	Obata & Goto	2		–		13				(66)						–			
10	Hofmann & Kuleshova	–		–		–		–		–		–		–		–		–	
11	Lv, Wang, Ma	–	–																
12	Marom *et al.*	–	–																
13	Mohamed	1		–		88				–						–		–	
14	Neumann, Kendrick, Leusen	2		26	85	2	4	–	6	11	39	–	–	2		6		1	1
15	Pantelides, Adjiman *et al.*	6		70		13				–						1		–	
16	Pickard *et al.*	–																	
17	Podeszwa *et al.*	8	3																
18	Price *et al.*	6	2	–	–	1	2			85	44			–	–	1	1	2	1
19	Szalewicz *et al.*	–																	
20	Tuckerman, Szalewicz *et al.*	4																	
21	Zhu, Oganov, Masunov	3	–	–		–				–				–		2		–	
22	Boese	*		*		*		*		*		*		*		*		*	
23	Brandenburg & Grimme	–	–	–	–	11	1			–	–			*	*	2		–	–
24	Szalewicz *et al.*													*					
25	Tkatchenko *et al.*	3	1	–	–	2	5			14	2			*		1			
