# Supplementary material for: Report on the sixth blind test of organic crystal structure prediction methods
Source: Acta Crystallogr B Struct Sci Cryst Eng Mater. 2016 Aug 1;72(Pt 4):439–59. doi: 10.1107/S2052520616007447 (PMC4971545; doi:10.1107/S2052520616007447)
Supplement: Supplementary file 1 [file b-72-00439-sup1.pdf]

Supporting information: report on the sixth blind test of organic crystal-structure prediction methods

Anthony M. Reilly, *et al.* *

The Cambridge Crystallographic Data Centre, 12 Union Road, Cambridge CB2 1EZ, United Kingdom

April 27, 2016

Summary and Data Access Statements

The supporting information for this publication includes all of the final submitted predictions in the crystallographic information framework (.cif) format. The relative energies of the structures are provided as a field or comment in some of these cifs, while for some submissions separate data files are provided. Additional details, analysis and discussion of the methods applied by each submission are also included in portable document format (.pdf). Those intending to cite these individual supporting-information documents are suggested to use the format: "[Main Paper Citation]; Supporting Information for Submission X: [Submission Authors]", or similar.

Tables S1–S8 report comparisons between the experimental crystal structures of each target and successful predictions. Table S9 shows the computation resources used in each submission, while Tables S10 and S11 give further details of each submissions methodology. Table S12 gives relative energies of the polymorphs of XXIII, as calculated with a range of methods. Finally, Table S13 gives CCDC numbers and digital object identifiers for each list of structures submitted in the blind test, which can be used to access and cite each putative crystal structure.

General requests for additional information or data can be directed to the corresponding author or the CCDC, who will then assist in contacting the relevant submission. **Submission 18:** The structures generated by submission 18, and re-ranked in submissions 23, 24 and 25 are held in the Crystal Navigator Database at UCL, and are available on request. **Submission 20:** Generated structures, molecular-dynamics trajectories, and ranking information can be found at the NYU Faculty Digital Archive (`archive.nyu.edu`).

*E-mail: `reilly@ccdc.cam.ac.uk`

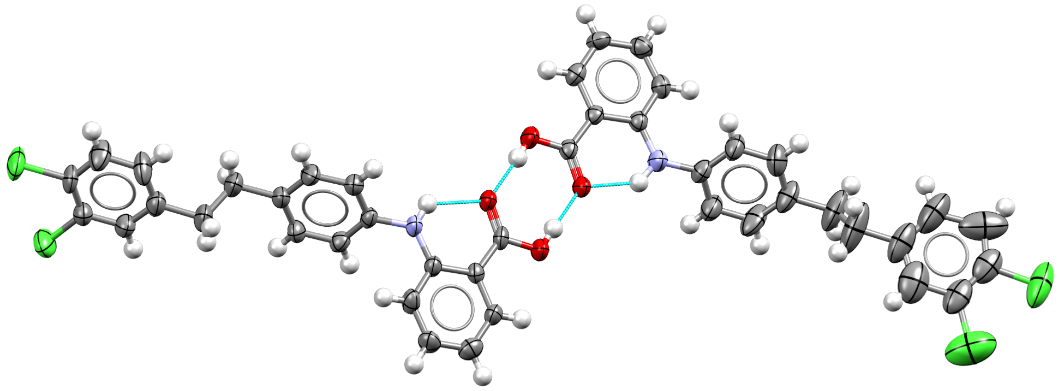

Figure S1: Asymmetric unit of XXIII form E, showing anisotropic displacement parameters (thermal ellipsoids) of the heavy atoms, plotted at the 50% probability level. H atoms are drawn as fixed-sized spheres for clarity.

Table S1: Comparison of the experimental structure and matching predictions of XXII, which crystallises as $P2_1/n$, in terms of the relative deviation in lattice parameters, volume and density: ((pred. − expt.)/expt.) × 100%. The root mean squared deviation for the overlay of matching clusters of 20 molecules ($RMSD_{20}$) and the overlay of the experimental and predicted conformations ($RMSD_1$) are also given in Å. Experimental values for lattice parameters, unit-cell volume and density are reported in Å and °, $Å^3$ and g/cm^3, respectively.

	Rank	List	a	b	c	β	Volume	Density	$RMSD_{20}$	$RMSD_1$
Experiment (T = 150 K)	–	–	11.947(2)	6.696(1)	12.598(3)	108.60(3)	955.164	1.727	–	–
Day *et al.*	1	2	−2.07	−0.68	−3.23	−3.13	−4.17	4.33	0.267	0.043
Dzyabchenko	1	1	0.44	1.61	−0.44	−1.84	2.73	−2.68	0.189	0.060
van Eijck	4	1	2.40	−1.57	−1.89	−1.19	−0.39	0.37	0.269	0.051
van den Ende, Cuppen *et al.*	9	1	0.91	−0.56	0.05	−0.07	0.44	−0.46	0.196	0.113
Obata & Goto	2	1	6.14	−1.49	8.22	−2.32	14.72	−12.85	0.808	0.049
Mohamed	1	1	0.70	−0.17	−0.47	−1.99	1.26	−1.26	0.234	0.081
Neumann, Kendrick, Leusen	2	1	1.78	0.80	2.61	1.26	4.40	−4.23	0.170	0.040
Pantelides, Adjiman *et al.*	6	1	1.27	−1.52	−3.14	−2.97	−1.72	1.73	0.306	0.067
Podeszwa *et al.*	3	2	3.00	−0.49	0.80	0.17	3.20	−3.12	0.257	0.111
Price *et al.*	6	1	1.94	−0.68	−1.32	−2.29	1.27	−1.27	0.260	0.041
Price *et al.*	2	2	1.85	1.38	1.29	−1.22	5.38	−5.12	0.204	0.048
Tuckerman, Szalewicz *et al.*	4	1	1.66	0.70	0.43	0.47	2.49	−2.45	0.187	0.102
Zhu, Oganov, Masunov	3	1	2.07	−0.93	−2.48	−3.26	0.48	−0.49	0.340	0.046
Tkatchenko *et al.* (Price)	1	2	1.53	0.82	0.20	−0.83	3.10	−3.02	0.166	0.026

Table S2: Comparison of the experimental structure and matching predictions of XXIII A, which crystallises as $P2_1/c$, in terms of the relative deviation in lattice parameters, volume and density: ((pred. − expt.)/expt.) × 100%. The root mean squared deviation for the overlay of matching clusters of 20 molecules ($RMSD_{20}$) and the overlay of the experimental and predicted conformations ($RMSD_1$) are also given in Å. Experimental values for lattice parameters, unit-cell volume and density are reported in Å and °, $Å^3$ and g/cm^3, respectively.

	Rank	a	b	c	β	Volume	Density	$RMSD_{20}$	$RMSD_1$
Experiment (T = 300 K)	–	11.1637(10)	10.5295(10)	16.2358(15)	95.749(2)	1898.9(3)	1.351	–	–
Day *et al.*	23	−2.57	3.05	1.76	1.03	1.98	−1.93	0.388	0.181
van Eijck	83	9.60	−2.54	−4.23	−3.62	2.73	−2.65	0.785	0.177
Neumann, Kendrick, Leusen	26	−2.37	0.48	−0.07	−0.77	−1.86	1.90	0.181	0.069
Pantelides, Adjiman *et al.*	70	8.68	−6.20	−3.96	0.87	−2.25	2.31	0.769	0.232

Table S3: Comparison of the experimental structure and matching predictions of XXIII B, which crystallises as $P\bar{1}$, in terms of the relative deviation in lattice parameters, volume and density: ((pred. − expt.)/expt.) × 100%. The root mean squared deviation for the overlay of overlay of matching clusters of 20 molecules ($RMSD_{20}$) and the overlay of the experimental and predicted conformations ($RMSD_1$) are also given in Å. Experimental values for lattice parameters, unit-cell volume and density are reported in Å and °, $Å^3$ and g/cm^3, respectively.

	Rank	List	a	b	c	α	β	γ	Volume	Density	$RMSD_{20}$	$RMSD_1$
Experiment ($T = 300$ K)	–	–	7.0061(13)	7.8047(15)	18.893(4)	85.277(4)	80.753(4)	65.769(3)	929.7(3)	1.380	–	–
Day *et al.*	75	2	2.40	5.71	−4.25	4.88	7.00	−0.68	4.44	−4.26	0.733	0.253
van Eijck	20	1	2.93	3.87	−3.82	4.81	3.05	−1.86	2.35	−2.30	0.548	0.132
Elking & Fusti-Molnar	78	1	2.61	2.47	−4.83	5.15	2.03	−0.60	0.04	−0.06	0.550	0.148
Obata & Goto	13	1	2.90	5.19	−1.21	2.51	4.96	−1.10	7.28	−6.79	0.512	0.144
Mohamed	88	1	4.55	2.49	2.37	1.73	8.26	−2.95	9.19	−8.43	0.827	0.339
Neumann, Kendrick, Leusen	2	1	1.18	−0.21	−3.25	2.69	−0.20	−2.11	−3.50	3.61	0.344	0.092
Pantelides, Adjiman *et al.*	13	1	2.92	1.97	−6.23	−2.46	3.27	0.06	−1.23	1.23	0.767	0.180
Price *et al.*	1	1	2.22	0.85	−4.36	4.57	1.08	−1.19	−1.91	1.94	0.476	0.133
Price *et al.*	2	1	2.21	0.75	−4.36	4.67	1.16	−1.24	−2.03	2.06	0.480	0.133
Brandenburg & Grimme (Price)	11	1	0.47	−3.39	−3.07	3.33	−1.09	−2.53	−7.53	8.12	0.524	0.223
Brandenburg & Grimme (Price)	1	2	2.74	−0.05	−6.10	4.86	0.18	−3.60	−5.62	5.94	0.608	0.125
Tkatchenko *et al.* (Price)	2	1	1.63	−0.62	−4.78	4.28	0.60	−1.33	−4.48	4.67	0.470	0.094

Table S4: Comparison of the experimental structure and matching prediction of XXIII C, which crystallises as $Z' = 2$ in $P2_1/c$, in terms of the relative deviation in lattice parameters, volume and density: ((pred. − expt.)/expt.) × 100%. The root mean squared deviation for the overlay of matching clusters of 20 molecules ($RMSD_{20}$) is also given in Å. Experimental values for lattice parameters, unit-cell volume and density are reported in Å and °, $Å^3$ and g/cm^3, respectively.

	Rank	a	b	c	α	β	γ	Volume	Density	$RMSD_{20}$
Experiment ($T = 300$ K)	–	7.6375(11)	12.0393(17)	20.443(3)	84.790(3)	85.379(3)	80.091(3)	1840.0(5)	1.394	–
Neumann, Kendrick, Leusen	6	−1.40	−1.78	−0.60	2.01	1.24	0.28	−3.38	3.53	0.228

Table S5: Comparison of the experimental structure and matching predictions of XXIII D, which crystallises as $P2_1/n$, in terms of the relative deviation in lattice parameters, volume and density: ((pred. − expt.)/expt.) × 100%. The root mean squared deviation for the overlay of matching clusters of 20 molecules ($RMSD_{20}$) and the overlay of the experimental and predicted conformations ($RMSD_1$) are also given in Å. Experimental values for lattice parameters, unit-cell volume and density are reported in Å and °, Å^3 and g/cm^3, respectively.

	Rank	List	a	b	c	β	Volume	Density	$RMSD_{20}$	$RMSD_1$
Experiment (T = 300 K)	–	–	13.886(4)	10.728(3)	14.078(4)	113.632(5)	1921.3(9)	1.335	–	–
Day *et al.*	75	1	2.18	1.86	2.33	2.59	3.98	−3.80	0.410	0.225
Neumann, Kendrick, Leusen	11	1	−2.32	1.14	0.90	2.58	−2.68	2.78	0.469	0.131
Price *et al.*	85	1	−2.10	1.29	1.55	1.80	−0.94	0.98	0.417	0.109
Price *et al.*	44	2	−2.29	1.16	1.48	1.83	−1.36	1.40	0.422	0.109
Tkatchenko *et al.* (Price)	2	2	−3.47	0.42	0.83	1.56	−3.62	3.79	0.437	0.113

Table S6: Comparison of the experimental structure and matching prediction of XXIV, which crystallises as $P2_1/c$, in terms of the relative deviation in lattice parameters, volume and density: ((pred. − expt.)/expt.) × 100%. The root mean squared deviation for the overlay of matching clusters of 60 components ($RMSD_{60}$, including H atoms) is also given in Å. Experimental values for lattice parameters, unit-cell volume and density are reported in Å and °, Å^3 and g/cm^3, respectively.

	Rank	a	b	c	β	Volume	Density	$RMSD_{60}$
Experiment (T = 240 K)	–	3.9906(1)	21.2366(6)	10.1014(3)	97.833(2)	848.07(4)	1.571	–
Neumann, Kendrick, Leusen	2	2.70	−1.47	−1.32	0.77	−0.34	0.34	0.169

Table S7: Comparison of the experimental structure and matching predictions of XXV, which crystallises as $P2_1/c$, in terms of the relative deviation in lattice parameters, volume and density: ((pred. − expt.)/expt.) × 100%. The root mean squared deviation for the overlay of matching clusters of 20 molecules (RMSD_{20}) is also given in Å. Experimental values for lattice parameters, unit-cell volume and density are reported in Å and °, $Å^3$ and g/cm^3, respectively.

	Rank	List	a	b	c	β	Volume	Density	RMSD_{20}
Experiment (T = 298 K)	–	–	10.4240(2)	27.5781(6)	8.1258(2)	109.564(1)	2201.10(8)	1.396	–
van Eijck	1	1	4.58	1.43	−4.60	0.73	0.68	−0.71	0.464
Neumann, Kendrick, Leusen	6	1	0.19	−1.12	−1.16	0.33	−2.30	2.32	0.124
Pantelides, Adjiman *et al.*	1	1	3.24	1.60	−3.53	1.42	0.18	−0.21	0.340
Price *et al.*	1	1	3.03	1.04	−3.69	1.15	−0.54	0.51	0.316
Price *et al.*	1	2	2.71	0.79	−3.66	1.15	−1.07	1.04	0.310
Zhu, Oganov, Masunov	2	1	0.97	−0.18	−5.24	1.10	−5.22	5.48	0.296
Brandenburg & Grimme (Price)	2	1	−0.54	−0.92	−4.68	0.31	−6.27	6.65	0.273
Tkatchenko *et al.* (Price)	1	1	2.02	0.75	−3.82	1.38	−2.11	2.12	0.295

Table S8: Comparison of experimental and matching predictions of form 1 of XXVI, which crystallises as $P\bar{1}$, in terms of the relative deviation in lattice parameters, volume and density: ((pred. − expt.)/expt.) × 100%. The root mean squared deviation for the overlay of overlay of matching clusters of 20 molecules (RMSD_{20}) and the overlay of the experimental and predicted conformations (RMSD_1) are also given in Å. Experimental values for lattice parameters, unit-cell volume and density are reported in Å and °, $Å^3$ and g/cm^3, respectively.

	Rank	List	a	b	c	α	β	γ	Volume	Density	RMSD_{20}	RMSD_1
Experiment (T = 298 K)	–	–	10.4022(8)	11.0302(14)	14.1789(10)	76.829(8)	73.331(7)	63.470(12)	1384.9(3)	1.346	–	–
Elking & Fusti-Molnar	8	1	0.47	−0.28	2.39	14.60	1.24	−1.14	2.52	−2.46	0.366	0.186
Elking & Fusti-Molnar	1	2	−1.32	−1.95	−2.73	2.65	1.66	1.99	−4.03	4.20	0.295	0.096
Neumann, Kendrick, Leusen	1	1	−1.22	0.33	−1.59	2.27	0.20	0.30	−1.90	1.94	0.227	0.080
Price *et al.*	2	1	−1.33	1.30	0.38	2.23	0.27	−0.96	0.25	−0.25	0.285	0.126
Price *et al.*	1	2	−1.37	1.30	0.18	2.29	0.09	−0.95	−0.04	0.04	0.293	0.126

Table S9: Summary of the computational resources used by each submission in terms of raw CPU hours. Due to the range of hardware and facilities used the numbers have not been normalised. In total, over 40 million CPU hours were used by the submissions combined.

Team	XXII	XXIII	XXIV	XXV	XXVI	Total	Notes
Chadha & Singh	350	450			600	1,400	Intel® Xeon® 3.2 GHz processors
Cole *et al.*	6	538		46	246	836	Intel Core™ i7 3.5 GHz processors
Day *et al.*	12,714	394,948	15,241	121,701	179,897	724,501	Range of processors/hardware, see SI document
Dzyabchenko	144	3,648	3,360			7,152	Intel Xeon 5450
van Eijck	130	2,810	1,400	8,060	7,630	20,030	Normalised to 2.66 GHz Intel Quad 9400 processors
Elking & Fusti-Molnar	418,540	242,000	235,400	135,000	190,000	1,220,940	Intel Xeon Processors
van den Ende, Cuppen *et al.*	9,741	7,777		6,388		23,906	Intel and AMD processors (2.2–2.6 GHz)
Facelli *et al.*	268,012	38,500	11,500	39,000		357,012	Intel Xeon E5-2670 processors (2.6 GHz), time for XXII includes alternative *ab initio* method
Obata & Goto	19,200	346,000		325,000		690,200	Normalised to Intel Xeon 2.7 GHz
Hofmann & Kuleshova	10	630	623	202	255	1,720	Intel E5440 2.8 GHz processors
Lv, Wang, Ma	325,000					325,000	Normalised to 3 GHz
Marom *et al.*	30,000,000					30,000,000	1.6 GHz PowerPCs (for majority) and Intel Xeon E5-2680 2.8 GHz processors
Mohamed	26	106		81	61	274	2.0 GHz and 2.2 GHz processors
Neumann, Kendrick, Leusen	32,160	146,120	103,700	84,680	356,844	723,504	Normalised to 2.6 GHz
Pantelides, Adjiman *et al.*	333	87,000		37,535	272,500	397,368	Typically Intel Xeon E5-2660 2.20 GHz processors
Pickard *et al.*	380,000					380,000	Intel Xeon E5-2680v2 2.8 GHz and Ivy Bridge E5-2697v2 2.7 Ghz
Podeszwa *et al.*	72,220					72,220	2.6 and 2.2 GHz AMD Opteron™ processors (counting potential generation by Szalewicz *et al.*)
Price *et al.*	26,000	84,000	63,000	169,000	327,000	669,000	Various (old) hardware, see SI document
Szalewicz *et al.*	66,000					66,000	Intel Ivy Bridge 2.5 GHz
Tuckerman, Szalewicz *et al.*	81,000					81,000	AMD Athlon™ X4 620 2.6 GHz and Intel Xeon 2695v3 2.3 GHz processors (counting potential generation by Szalewicz *et al.*)
Zhu, Oganov, Masunov	4,000	275,000	279,800	30,000	180,000	768,800	Intel Xeon E5-2630v2 2.6 GHz
Boese (Hofmann)	80,000	80,000	80,000	80,000	80,000	400,000	Intel Xeon E5-2650v2 2.6 GHz
Brandenburg & Grimme (Price)	13,665	8,661	3,509	34,824	10,135	70,794	Intel Xeon E5620
Szalewicz *et al.* (Price)			15,000			15,000	Intel Ivy Bridge 2.5 GHz
Tkatchenko *et al.* (Price)	100,000	2,100,000	500,000	500,000		3,200,000	1.6 GHz PowerPCs & 2.6 GHz Intel Sandy Bridge-EP

Table S10: Brief summary of the methods used by each group in the investigation and generation of conformations and initial crystal structures. See respective SI document for each team for full details.

Team	Conformational Searches	Molecular Model in Search	Structure Generation	Software	Space groups	References
1	Molecular dynamics	Rigid conformations in search	Simulated annealing	*Materials Studio* 8.0	All 230 space groups	Karfunkel and Gdanitz (1992)
2	Generated using Corina and CSD bond length, angle and rotamer distributions	Rigid analogue	Based on CSD analogues	In-house software; *CSD Conformer Generator*	No restrictions on analogue structure's space group	
3	Low-mode conformation search method with OPLS2005 followed by DFT calculations	Rigid searches for all, one flexible search for XXIII	Sobol' sequences	*Global Lattice Energy Explorer* (*GLEE*)	94 space groups for XXII, up to 25 most-common space groups for others; see SI	Case et al. (2016)
4	XXII: bent *vs.* planar conformations of the free molecule have been compared by their optimised Hartree-Fock energies. Not a separate step for other systems	XXII: rigid throughout the packing search; XXVI: flexible with respect to torsion rotations about the central, the naphthalene-amide and the amide-chlorobenzene bonds	Systematic scan of parameter space for starting models: up to 1080 sets of Euler angles, eight center-of-mass positions of molecule in the unit cell and seven unit-cell shapes	*PMC* (updated version with new procedure for automatic scans of trial models)	$P\bar{1}$, $P2_1$, Pc, $P2_1/c$, $C2$, Cc, $P2_12_12_1$, $Pca2_1$, $Pna2_1$, $Pbca$, $C2/c$ for XXII and XXVI; $P\bar{1}$ and $P2_1/c$ for XXV	Dzyabchenko (2008)
5	CSD search and *ab initio* (6-31G*) conformational scans	Fully flexible molecules	Random search	*UPACK* 10/11, *GAMESS-UK* 6.2.1, *MOLDEN*	$P2_1/c$, $P\bar{1}$, $P2_12_12_1$, $P2_1$, $Pbca$, $C2/c$, $Pna2_1$, Cc, $Pca2_1$, $C2$, $P1$, $Pbcn$, and Pc	van Eijck and Kroon (2000); van Eijck (2015)
6	Conformations generated in the gas phase using a force field (MMFF)		Randomly generated structures	–	32 most-likely space groups (from CSD)	
7	DFT optimisation of gas-phase molecules	Flexible throughout all targets, one specific conformation (XXII), multiple conformations three random assigned dihedrals (XXIII), XXV three differently oriented 1:1 pairs and two differently oriented 1:2 triples both with three randomly assigned dihedrals	Quasi-random search	*UPACK* 10	$C2$, $C2/c$, $C2/m$, Cc, $P\bar{1}$, $P1$, $P2_1$, $P2_12_12$, $P2_12_12_1$, $P2_1/c$, $P2_1m$, $P2/c$, $Pbca$, $Pbcn$, Pc, $Pca2_1$, $Pccn$, $Pna2_1$, and $Pnma$	van Eijck and Kroon (2000)

Continued on next page...

Table S10: Brief summary of the methods used by each group in the investigation and generation of conformations and initial crystal structures. See respective SI document for each team for full details.

Team	Conformational Searches	Molecular Model in Search	Structure Generation	Software	Space groups	References
8	Randomly selected dihedrals	Flexible (dihedrals)	Genetic algorithm	*MGAC*	$P1$, $P\bar{1}$, $P2_1$, $C2$, Pc, Cc, $P2_1/c$, $C2/c$, $P2_12_12_1$, $Pca2_1$, $Pna2_1$, $Pbcn$, $Pbca$, and $Pnma$	Kim et al. (2009)
9	Gas-phase searches using *CONFLEX*	Fully flexible molecules	systematic grid search	*CONFLEX*	$P1$, $P\bar{1}$, $P2_1$, $C2$, Pc, Cc, $P2_1/c$, $C2/c$, $P2_12_12_1$, $Pca2_1$, $Pna2_1$, $Pbcn$, $Pbca$, and $Pnma$	Goto and Osawa (1989, 1993)
10	Molecular structures were analysed using systematic grid searches for possible conformations	Rigid conformations in search	Quasi-random searching using estimated cell volume	*Materials Studio*, *FlexCryst* 2.03.05	Nine most-common space groups	Hofmann (2010)
11	DFT geometry optimisations	Rigid conformations in structure	Random search under constraints of space-group symmetry	*CALYPSO* 4.0	Searches in space groups with $Z \leq 4$	Wang et al. (2012)
12	*Ab initio* unconstrained optimisation (PBE+TS) of monomer geometry	Initial pool contained different conformations; the molecules were fully flexible in the *ab initio* GA search	Genetic algorithm	*GAtor*	$P2_1$, $P2$, $P\bar{1}$, Pc, Pm, $P2_12_12_1$, $P2_12_12$, $C2$, $P2_1/c$, $Pca2_1$, and $Pna2_1$	
13	Conformational search space initially mapped at the (semi-empirical) AM1 level but final conformations were calculated *ab initio* [MP2/6-31G(d,p) or B3LYP/6-31G(d,p)]	Rigid conformation used for all systems during crystal structure searches. Two separate rigid body searches were performed for XXIII	Monte Carlo simulated annealing	*Materials Studio* 7.0	$P1$, $P\bar{1}$, $P2_1$, $P2_1/c$, $P2_12_12_1$, $P2_12_12$, $Pbca$, $Pna2_1$, $Pca2_1$, $C2/c$, Cc, $C2$ for all attempts with additional space groups for XXII; see SI document	Karfunkel and Gdanitz (1992)
14	Isolated-molecule conformer analysis with tailor-made force field to characterise molecular flexibility	Fully flexible molecules	Monte Carlo parallel tempering	*GRACE* 2.4	All 230 space groups for XXII, 38 most-common space groups for all other $Z' = 1$ searches and 11 most-likely for $Z' = 2$ searches	
15	Scans of specific torsions and CSD analysis	Partially flexible; see SI for details	Sobol' sequences	*CrystalPredictor*	59 most-common space groups	Kazantsev et al. (2010); Habgood et al. (2015)
16	–	Fully flexible molecule	*Ab initio* random structure searching	*CASTEP*	All space groups with $Z = 1, 2$ random space groups for $Z > 2$	Clark et al. (2005)

Continued on next page...

Table S10: Brief summary of the methods used by each group in the investigation and generation of conformations and initial crystal structures. See respective SI document for each team for full details.

Team	Conformational Searches	Molecular Model in Search	Structure Generation	Software	Space groups	References
17	*Ab initio* unconstrained optimisation (PBE0-D3)	Rigid conformation	Systematic angular sweep for each of the coordination geometries used with SAPT(DFT)-based potential	*MOLPAK*, *PMIN* (March 2014)	$P1$, $P\bar{1}$, $P2_1$, $P2_1/c$, Cc, $C2$, $C2/c$, Pc, $P2/c$, $P2_1/m$, $P2/m$, $P2$, Pm, $P2/m$, $P2_12_12$, $P2_12_12_1$, $Pca2_1$, $Pna2_1$, $Pnn2$, $Pba2$, $Pnc2$, $P222_1$, $Pmn2_1$, $Pma2$, $Pbcn$, $Pbca$	Holden et al. (2014)
18	*Ab initio* torsion scans + CSD surveys	Rigid conformations for XXII and XXV, partially flexible for XXIII, XXIV and XXVI	Sobol' sequences	*CrystalPredictor* 1.6–2.1	59 most-common space groups	Kazantsev et al. (2010, 2011); Habgood et al. (2015)
19	*Ab initio* unconstrained optimisation (PBE0-D3) of monomer geometry	Rigid conformation	Sobol' sequences	*CrystalPredictor* 1.6	59 most-common space groups	Kazantsev et al. (2010); Misquitta et al. (2005)
20	*Ab initio* unconstrained optimisation [PBE0-D3, aug-cc-pVTZ] to obtain the monomer geometry	Rigid conformation	Random packing followed by structure optimisation followed by thermal averaging using molecular dynamics in an isothermal-isobaric ensemble with a fully flexible cell. The stability of structures on a free-energy surface was tested using the Crystal-AFED approach	*UPACK* and *PINY_MD* (modified for use with SAPT(DFT) potentials)	16 common space groups	van Eijck and Kroon (1999); Tuckerman et al. (2000); Misquitta et al. (2005)
21	Exhaustive conformational search with FF partly fitted to DFT scans of potential-energy surface	Rigid conformations	Evolutionary algorithm; dimers used as starting points in some calculations for XXIII, XXV and XXVI	*TINKER*, *USPEX*	All triclinic, monoclinic, orthorhombic and tetragonal space groups with $Z \leq 8$ for $Z' = 1$ searches. $Z' = 2$ searches used $P1$, $P\bar{1}$, $P2_1$, Cc, Pc, $P2_1/c$, $P2_12_12_1$, $Pna2_1$, $Pca2_1$	Zhu et al. (2012); Lyakhov et al. (2013)

Table S11: Brief summary of the methods used by each group in the optimisation and ranking of generated crystal structures. See respective SI document for each team for full details.

		Final Predictions			
Team	Fitness Function for Generation and Initial Optimisation	List 1	List 2	Software	References
1	COMPASS (2.8) force field	Force-field energy ranking	–	*Materials Studio* 8.0	
2	CSD-fitted 6-exp potential (no partial charges) with a CSD-derived torsion term	Force-field score ranking, with some final lists partially filtered by contacts, motifs *etc.*	–	In-house software	
3	exp-6 potential (trained in some cases) with atomic multipoles calculated in polarisable continuum model	Ranked by final lattice energy after flexible optimisations	Rigid-body Helmholtz free energies at 300 K (XXII, XXV); lattice energies different polarisation treatments (XXIV and XXVI); Lattice energy after a fully flexible search for XXIII	*DMACRYS*, *CrystalOptimizer*	Price et al. (2010); Kazantsev et al. (2011); Nyman and Day (2015)
4	Lattice energy calculated with empirical potentials as a function of all structural parameters consistent with postulated space group	Ranked by final lattice energy from optimisations	–	*PMC*	–
5	Generation: OPLS-type Lennard-Jones potential; Initial optimisation: Price-Williams-type Buckingham function. Both with fixed point charges	Price-Williams-type Buckingham force field, 6-31G** calculations for individual point changes and intramolecular energies	–	*UPACK* 10/11, *GAMESS-UK* 6.2.1, *MOLDEN*	See SI document
6	8-6 LJ potential, distributed multipoles	Final structures with optimised molecular geometries and multipoles	DFT optimisations and re-ranking of intermediate results using PBE+XDM functional	*Quantum ESPRESSO*	Giannozzi et al. (2009)
7	Simple LJ force field and adapted Generalized Amber Force Field (GAFF); flexible molecules	Lattice-energy estimation using *q-GRID* for top-25 structures (XXII), 10 out of top-25 structures (XXIII); the rest of top 100 comes from adapted GAFF	Growth-rate analysis from kinetic Monte Carlo simulations	*q-GRID*, *Monty*	de Klerk et al. (2016); Deij et al. (2007)
8	CHARMM force field	Top 110 structures re-ranked with PBE-D2 density functional	Full *ab initio* search with PBE-D2 density functional and updated *MGAC* code	*Quantum ESPRESSO*	Giannozzi et al. (2009)
9	MMFF94 force field	Low-energy structures re-ranked with PBE+TS functional	(Continuation of List 1)	*CONFLEX* 7, *Materials Studio* 8.0, *CASTEP*	See SI document

Continued on next page...

Table S11: Brief summary of the methods used by each group in the optimisation and ranking of generated crystal structures. See respective SI document for each team for full details.

		Final Predictions			
Team	Fitness Function for Generation and Initial Optimisation	List 1	List 2	Software	References
10	Force field obtained in multi-step procedure from experimental crystal structures by data mining. In a first step an approximate function is derived by singular value decomposition and the final force field is refined by classification	Final ranking with FF energies	–	*FlexCryst* 2.03.05	Apostolakis et al. (2001)
11	Lattice energy obtain through plane-wave density-functional theory *via* PBE+optB86b-vdW functional	Re-optimisation of top 100 structures with PBE+optB86b-vdW functional (tighter settings)	(Continuation of List 1)	*VASP* 5.3	Kresse and Furthmüller (1996)
12	Harris functional evaluation of single-point PBE+TS energies	PBE+TS density functional optimisations	PBE+MBD density functional single-point energies	*FHI-aims*	Blum et al. (2009)
13	Dreiding force field used for search with atomic charges fitted to electrostatic potential of *ab initio* wavefunction	Re-optimisation of the lattice energy for the 2000 lowest energy structures from the Polymorph Predictor search using distributed multipole model of *ab initio* wavefunction using DMACRYS	–	*Materials Studio* 7.0, *DMACRYS* 2.0.8	Price et al. (2010)
14	Step 1: Lattice energies calculated with tailor-made force field; Step 2: Course DFT-D lattice energies	DFT-D with PBE functional and dispersion correction according to Neumann-Perrin	List also contains $Z' = 2$ structures for XXIII and XXVI	*GRACE* 2.4, *VASP* 5.2	Neumann and Perrin (2005); Kendrick et al. (2012); Kresse and Furthmüller (1996)
15	FIT potential and atomic charges for search, atomic multipoles used for optimisation; Intramolecular conformational energy interpolated from DFT calculations	Final flexible optimisations with atomic multipoles, FIT potential using DFT intramolecular energies	–	*Crystal Predictor*, *DMACRYS*, *CrystalOptimizer*	Kazantsev et al. (2010); Price et al. (2010); Kazantsev et al. (2011)
16	PBE density functional augmented with various vdW terms	Ranked on PBE+MBD after optimisations; see SI for discussion of harmonic and anharmonic free-energy contributions	–	*CASTEP* 8.0	Clark et al. (2005)
17	Minimum volume followed by energy minimisation for 500 highest-density structures for each coordination geometry. See SI documents for details of potentials	298 K molecular dynamics simulation with a different SAPT(DFT)-based potential	Previous step repeated with a different SAPT(DFT)-based potential	*DL_POLY Classic* 1.9, *SAPT2012.2*	Todorov et al. (2006); Misquitta et al. (2005)

Continued on next page...

Table S11: Brief summary of the methods used by each group in the optimisation and ranking of generated crystal structures. See respective SI document for each team for full details.

Team	Fitness Function for Generation and Initial Optimisation	Final Predictions: List 1	Final Predictions: List 2	Software	References
18	Lattice energy calculated with atomic charges and empirical exp-6 intermolecular model, and interpolation of a grid of *ab initio* conformational energies	Optimisation of lattice energy from PBE0/6-31G(d,p) intramolecular energy and distributed multipoles and intermolecular energy from distributed multipoles and repulsion-dispersion FIT exp-6 potential	Re-ranking with second derivative entropy estimate and PCM ($\epsilon = 3$) polarization. Similar structures were removed	*DMACRYS* 2.2.0.1, *CrystalOptimizer* 2.4	Price et al. (2010); Kazantsev et al. (2011); Habgood et al. (2015)
19	Analytical fit to SAPT(DFT) surface with combining rules for exp-6-1 parameters.	Re-optimisation of lattice energy from analytical undamped atom-atom ex-6-1 function fitted to SAPT(DFT) dimer intermolecular energies	–	*CrystalPredictor* 1.6, *DMACRYS* 2.2.0.1	Kazantsev et al. (2010); Price et al. (2010)
20	*Ab initio* potential-energy surface with rigid monomers built as a sum of pair energies. *Ab initio* calculation on a grid of dimer's inter-monomer configurations performed using SAPT(DFT) and then fitted to an analytic function	Energies of structures determined by thermal averaging using molecular dynamics in an isothermal-isobaric ensemble with a fully flexible cell	–	*SAPT*, *PINY_MD*, *PLATON*	Tuckerman et al. (2000); Spek (2009); Misquitta et al. (2005)
21	Atomic multipoles and intermolecular force field (XXII, XXIII, XXV and XXVI), vdW-DF density functional for XXIV	Lowest-energy structures re-ranked with vdW-DF functional	–	*DMACRYS*, *VASP*	Price et al. (2010); Kresse and Furthmüller (1996)
22	Structures supplied by Hofmann (Submission 10)	Top 100 structures of the two last snapshots of submission 10 were optimised with the PBE+TS functional for compounds XXII, XXIII, XXV, and XXVI. For compound XXIV BLYP-D3 was used. Zero-point energies computed by finite differences of gradients, where then added to the final DFT energies	–	*VASP* 5.4.1	Becke (1988); Kresse and Furthmüller (1996); Lee et al. (1988); Perdew et al. (1996); Tkatchenko and Scheffler (2009); Grimme et al. (2010)
23	Structures supplied by Price (Submission 18) and then filtered on single-point energies with density-functional tight binding and minimal basis-set corrected Hartree-Fock theory	Lowest-energy structures fully optimised with HF-3c^{atm} method	Lowest-energy structures from List 1, optimised and ranked with TPSS-D3^{atm} density functional and combined with HF-3c^{atm} zero-point corrections	*CRYSTAL14*, *VASP*	Dovesi et al. (2014); Kresse and Furthmüller (1996); Grimme et al. (2010); Tao et al. (2003)

Continued on next page...

Table S11: Brief summary of the methods used by each group in the optimisation and ranking of generated crystal structures. See respective SI document for each team for full details.

Team	Fitness Function for Generation and Initial Optimisation	Final Predictions: List 1	Final Predictions: List 2	Software	References
24	Structures supplied by Price (Submission 18)	Evaluation of lattice energy from analytical undamped atom-atom exp-6-1 function fitted to SAPT(DFT) intermolecular energies of the 6 dimer types.	–	*DMACRYS* 2.0.8	Price et al. (2010); Misquitta et al. (2005)
25	Structures supplied by Price (Submission 18) and then optimised with PBE+TS density functional	Single-point PBE+MBD density functional total energy	PBE+MBD energies augmented with Helmholtz free energies at 300 K (PBE+TS)	*FHI-aims*	Blum et al. (2009); Tkatchenko and Scheffler (2009); Tkatchenko et al. (2012); Ambrosetti et al. (2014)

Table S12: The stabilities of the five experimentally known polymorphs of XXIII calculated by different teams after the blind test deadline, reported relative to the lowest-energy polymorph at that level of theory. All values are in kJ/mol (per molecule), apart from those of Team 10, which are dimensionless. All vibrational free-energy contributions (F_{vib}) have been calculated at 300 K. While the method column provides a brief summary of the methods employed, there are many underlying differences between the different approaches, *e.g.* density-functional theory basis set and self-consistent field convergence parameters, k- and q-point sampling, wavefunctions used for atomic charges and multipoles and intra-molecular energies, and geometries used for the calculations. Please consult the SI documents of each submission for full details.

Team	Method	Form A	Form B	Form C	Form D	Form E
3	Atomic multipoles and exp-6	1.3	5.5	0.0	2.5	0.5
5	Atomic charges and exp-6	4.2	0.0	5.6	5.6	4.6
10	Data-mining force field	23	18	5	48	0
14	PBE+Neumann-Perrin	3.9	0.0	0.1	2.7	2.0
18	Atomic multipoles and exp-6	9.4	0.0	3.3	9.2	5.3
18	As above with F_{vib}	7.4	0.0	1.8	7.1	–
R22	PBE+TS	4.5	0.0	2.8	7.0	5.8
R22	PBE+TS + F_{vib} (PBE+TS)	1.6	2.9	0.0	0.8	2.2
R22	PBE+MBD	3.8	0.8	0.0	4.5	2.1
R22	PBE+MBD + F_{vib} (PBE+TS)	3.7	6.5	0.0	1.2	1.4
R22	optB88-vdW	5.5	0.2	0.0	7.6	3.8
R22	optB88-vdW + F_{vib} (PBE+TS)	5.4	5.9	0.0	4.3	3.0
R22	RPBE-D3	0.8	0.4	0.0	1.2	1.3
R22	RPBE-D3 + F_{vib} (PBE+TS)	2.8	8.2	2.1	0.0	2.6
R23	HF-3c	11.2	2.9	0.0	10.4	5.4
R23	TPSS-D3	3.3	0.0	5.8	5.4	3.7
R23	TPSS-D3 + F_{vib}	4.1	0.0	3.7	2.9	1.7
R25	PBE+TS	4.4	0.0	2.3	6.4	4.7
R25	PBE+TS + F_{vib}	1.9	0.0	2.1	2.7	1.8
R25	PBE+MBD	4.0	1.9	0.0	4.7	1.9
R25	PBE+MBD + F_{vib} (PBE+TS)	2.5	2.9	0.9	2.0	0.0

Table S13: CCDC numbers and digital object identifier of each of the submissions in the blind test, broken down by submission, target and list. Although the CCDC numbers for a given list are not necessarily a complete block, they do match the order of the submitted structures. Note that at most two submitted lists were considered in the blind test, but a third list for Submission 12 is included for reference (see SI document for more details).

Submission	Target	List	CCDC numbers	DOI
1	XXII	1	1457235-67, 1457312-45, 1457277-309	10.5517/ccdc.6bt.s01.xxii.l1
1	XXIII	1	1457429-61, 1457464-96, 1457510-43	10.5517/ccdc.6bt.s01.xxiii.l1
1	XXVI	1	1458119-51, 1458164-96, 1458198-231	10.5517/ccdc.6bt.s01.xxvi.l1
2	XXII	1	1452500-32, 1452540-73, 1452575-607	10.5517/ccdc.6bt.s02.xxii.l1
2	XXIII	1	1452620-69, 1452689-738	10.5517/ccdc.6bt.s02.xxiii.l1
2	XXV	1	1453605-654, 1453811-60	10.5517/ccdc.6bt.s02.xxv.l1
2	XXVI	1	1461807-39, 1461840-72, 1461873-906	10.5517/ccdc.6bt.s02.xxvi.l1
3	XXII	1	1458237-70, 1458274-306, 1458308-40	10.5517/ccdc.6bt.s03.xxii.l1
3	XXII	2	1458341-73, 1458383-415, 1458416-49	10.5517/ccdc.6bt.s03.xxii.l2
3	XXIII	1	1458463-95, 1458496-528, 1458529-62	10.5517/ccdc.6bt.s03.xxiii.l1
3	XXIII	2	1458568-600, 1458601-34, 1458637-69	10.5517/ccdc.6bt.s03.xxiii.l2
3	XXIV	1	1458725-57, 1458758-90, 1458791-824	10.5517/ccdc.6bt.s03.xxiv.l1
3	XXIV	2	1459339-71, 1459372-404, 1459405-38	10.5517/ccdc.6bt.s03.xxiv.l2
3	XXV	1	1459440-72, 1459477-509, 1459510-43	10.5517/ccdc.6bt.s03.xxv.l1
3	XXV	2	1459544-77, 1459579-611, 1459613-45	10.5517/ccdc.6bt.s03.xxv.l2
3	XXVI	1	1459646-78, 1459679-711, 1459712-45	10.5517/ccdc.6bt.s03.xxvi.l1
3	XXVI	2	1459746-78, 1459779-812, 1459813-45	10.5517/ccdc.6bt.s03.xxvi.l2
4	XXII	1	1459846-78, 1459880-912, 1459913-46	10.5517/ccdc.6bt.s04.xxii.l1
4	XXV	1	1459947-76, 1459977-1460012, 1460013-45, 1460046-75, 1460076-107	10.5517/ccdc.6bt.s04.xxv.l1
4	XXVI	1	1460108-143, 1460144-78	10.5517/ccdc.6bt.s04.xxvi.l1
5	XXII	1	1461165-97, 1461198-230, 1461231-64	10.5517/ccdc.6bt.s05.xxii.l1
5	XXIII	1	1461267-99, 1461300-32, 1461333-66	10.5517/ccdc.6bt.s05.xxiii.l1
5	XXIV	1	1461370-402, 1461403-35, 1461436-69	10.5517/ccdc.6bt.s05.xxiv.l1
5	XXV	1	1461471-503, 1461504-36, 1461537-70	10.5517/ccdc.6bt.s05.xxv.l1
5	XXVI	1	1461572-604, 1461606-38, 1461639-72	10.5517/ccdc.6bt.s05.xxvi.l1
6	XXII	1	1460179-212, 1460213-42, 1460243-78	10.5517/ccdc.6bt.s06.xxii.l1
6	XXII	2	1460289-320, 1460321-54, 1460355-86	10.5517/ccdc.6bt.s06.xxii.l2
6	XXIII	1	1460387-420, 1460421-53, 1460454-86	10.5517/ccdc.6bt.s06.xxiii.l1
6	XXIII	2	1460487-510, 1460511-35	10.5517/ccdc.6bt.s06.xxiii.l2
6	XXIV	1	1460536-69, 1460570-602, 1460603-35	10.5517/ccdc.6bt.s06.xxiv.l1
6	XXIV	2	1460636-68, 1460669-99, 1460700-33	10.5517/ccdc.6bt.s06.xxiv.l2
6	XXV	1	1460893-926, 1460928-60, 1460961-93	10.5517/ccdc.6bt.s06.xxv.l1
6	XXV	2	1460999-1461025	10.5517/ccdc.6bt.s06.xxv.l2
6	XXVI	1	1461027-60, 1461061-93, 1461094-126	10.5517/ccdc.6bt.s06.xxvi.l1
6	XXVI	2	1461127-44, 1461145-64	10.5517/ccdc.6bt.s06.xxvi.l2
7	XXII	1	1461910-1461943, 1461944-1461983, 1461984-1462009	10.5517/ccdc.6bt.s07.xxii.l1
7	XXII	2	1462110-1462149, 1462150-1462183, 1462184-1462209	10.5517/ccdc.6bt.s07.xxii.l2

Continued on next page...

Table S13: CCDC numbers and digital object identifier of each of the submissions in the blind test, broken down by submission, target and list. Although the CCDC numbers for a given list are not necessarily a complete block, they do match the order of the submitted structures. Note that at most two submitted lists were considered in the blind test, but a third list for Submission 12 is included for reference (see SI document for more details).

Submission	Target	List	CCDC numbers	DOI
7	XXIII	1	1462010-1462035, 1462036-1462075, 1462076-1462109	10.5517/ccdc.6bt.s07.xxiii.l1
7	XXIII	2	1462210-1462243, 1462244-1462283, 1462284-1462309	10.5517/ccdc.6bt.s07.xxiii.l2
7	XXV	1	1462310-1462343, 1462344-1462369, 1462370-1462409	10.5517/ccdc.6bt.s07.xxv.l1
7	XXV	2	1462410-1462449, 1462450-1462483, 1462484-1462509	10.5517/ccdc.6bt.s07.xxv.l2
8	XXII	1	1462588-1462618, 1462619-1462656, 1462657-1462687	10.5517/ccdc.6bt.s08.xxii.l1
8	XXII	2	1462511-1462537, 1462538-1462562, 1462563-1462587	10.5517/ccdc.6bt.s08.xxii.l2
8	XXIII	1	1462688-1462728, 1462729-1462763, 1462764-1462787	10.5517/ccdc.6bt.s08.xxiii.l1
8	XXIV	1	1465815-1465855, 1465924-1465953, 1465988-1466016	10.5517/ccdc.6bt.s08.xxiv.l1
8	XXV	1	1466085-1466111, 1466186-1466227, 1466287-1466317	10.5517/ccdc.6bt.s08.xxv.l1
9	XXII	1	1466413-1466446, 1466527-1466552, 1466557-1466596	10.5517/ccdc.6bt.s09.xxii.l1
9	XXII	2	1466656-1466689, 1466690-1466729, 1466777-1466802	10.5517/ccdc.6bt.s09.xxii.l2
9	XXIII	1	1466837-1466870, 1466871-1466896, 1466923-1466962	10.5517/ccdc.6bt.s09.xxiii.l1
9	XXIII	2	1466963-1467002, 1467006-1467039, 1467040-1467065	10.5517/ccdc.6bt.s09.xxiii.l2
9	XXV	1	1467066-1467099, 1467100-1467125, 1467166-1467205	10.5517/ccdc.6bt.s09.xxv.l1
9	XXV	2	1467206-1467239, 1467240-1467279, 1467281-1467306	10.5517/ccdc.6bt.s09.xxv.l2
10	XXII	1	1462894-936, 1462937-67, 1462968-93	10.5517/ccdc.6bt.s10.xxii.l1
10	XXIII	1	1463011-37, 1463038-61, 1463134-58, 1463159-86	10.5517/ccdc.6bt.s10.xxiii.l1
10	XXIV	1	1463283-313, 1463314-49, 1463350-82	10.5517/ccdc.6bt.s10.xxiv.l1
10	XXV	1	1463383-429, 1463430-58, 1463460-83	10.5517/ccdc.6bt.s10.xxv.l1
10	XXVI	1	1463484-525, 1463526-52, 1463553-83	10.5517/ccdc.6bt.s10.xxvi.l1
11	XXII	1	1467321-1467349, 1467350-1467394, 1467395-1467420	10.5517/ccdc.6bt.s11.xxii.l1
11	XXII	2	1467421-1467463, 1467464-1467493, 1467494-1467520	10.5517/ccdc.6bt.s11.xxii.l2
12	XXII	1	1467521-1467560, 1467561-1467595, 1467596-1467620	10.5517/ccdc.6bt.s12.xxii.l1
12	XXII	2	1467621-1467658, 1467659-1467690, 1467691-1467720	10.5517/ccdc.6bt.s12.xxii.l2
12	XXII	3	1464784-1464828, 1467721-1467750, 1467751-1467775	10.5517/ccdc.6bt.s12.xxii.l3
13	XXII	1	1467776-1467816, 1467817-1467845, 1467846-1467875	10.5517/ccdc.6bt.s13.xxii.l1
13	XXIII	1	1467876-1467913, 1467914-1467948, 1467949-1467975	10.5517/ccdc.6bt.s13.xxiii.l1
13	XXV	1	1468187-1468222, 1468229-1468265, 1468266-1468292	10.5517/ccdc.6bt.s13.xxv.l1
13	XXVI	1	1467976-1468015, 1468016-1468046, 1486047-1468075	10.5517/ccdc.6bt.s13.xxvi.l1
14	XXII	1	1463584-617, 1463618-57, 1463658-83	10.5517/ccdc.6bt.s14.xxii.l1
14	XXIII	1	1465387-418, 1465421-62, 1465463-88	10.5517/ccdc.6bt.s14.xxiii.l1
14	XXIII	2	1465681-722, 1465756-81, 1465783-814	10.5517/ccdc.6bt.s14.xxiii.l2
14	XXIV	1	1465890-923, 1466146-85, 1466261-86	10.5517/ccdc.6bt.s14.xxiv.l1
14	XXV	1	1466386-411, 1466450-90, 1466494-526	10.5517/ccdc.6bt.s14.xxv.l1
14	XXVI	1	1466598-629, 1466630-55, 1466730-71	10.5517/ccdc.6bt.s14.xxvi.l1
14	XXVI	2	1466803-36, 1466897-922, 1467126-65	10.5517/ccdc.6bt.s14.xxvi.l2
15	XXII	1	1468295-1468334, 1468335-1468360, 1468368-1468401	10.5517/ccdc.6bt.s15.xxii.l1
15	XXIII	1	1468402-1468426, 1468427-1468456, 1468458-1468502	10.5517/ccdc.6bt.s15.xxiii.l1

Continued on next page...

Table S13: CCDC numbers and digital object identifier of each of the submissions in the blind test, broken down by submission, target and list. Although the CCDC numbers for a given list are not necessarily a complete block, they do match the order of the submitted structures. Note that at most two submitted lists were considered in the blind test, but a third list for Submission 12 is included for reference (see SI document for more details).

Submission	Target	List	CCDC numbers	DOI
15	XXV	1	1468506-1468557, 1468558-1468586, 1468587-1468605	10.5517/ccdc.6bt.s15.xxv.l1
15	XXVI	1	1468606-1468635, 1468640-1468660, 1468661-1468709	10.5517/ccdc.6bt.s15.xxvi.l1
16	XXII	1	1468711-1468716	10.5517/ccdc.6bt.s16.xxii.l1
16	XXII	2	1468717-1468743	10.5517/ccdc.6bt.s16.xxii.l2
17	XXII	1	1463187-1463219, 1463924-1463989	10.5517/ccdc.6bt.s17.xxii.l1
18	XXII	1	1464706-1464772, 1464829-1464861	10.5517/ccdc.6bt.s18.xxii.l1
18	XXII	2	1464862-1464927, 1464961-1464994	10.5517/ccdc.6bt.s18.xxii.l2
18	XXIII	1	1465547-1465646	10.5517/ccdc.6bt.s18.xxiii.l1
18	XXIII	2	1464928-1464960, 1464998-1465030, 1465504-1465537	10.5517/ccdc.6bt.s18.xxiii.l2
18	XXIV	1	1465723-1465755, 1465856-1465889, 1465954-1465986	10.5517/ccdc.6bt.s18.xxiv.l1
18	XXIV	2	1465647-1465679, 1466228-1466260, 1466351-1466384	10.5517/ccdc.6bt.s18.xxiv.l2
18	XXV	1	1466017-1466049, 1466051-1466084, 1466112-1466144	10.5517/ccdc.6bt.s18.xxv.l1
18	XXV	2	1463990-1464022, 1464057-1464090, 1466318-1466350	10.5517/ccdc.6bt.s18.xxv.l2
18	XXVI	1	1464126-1464191, 1464091-1464124	10.5517/ccdc.6bt.s18.xxvi.l1
18	XXVI	2	1464192-1464258, 1464265-1464297	10.5517/ccdc.6bt.s18.xxvi.l2
19	XXII	1	1464602-1464634, 1464638-1464703	10.5517/ccdc.6bt.s19.xxii.l1
20	XXII	1	1464548-1464601	10.5517/ccdc.6bt.s20.xxii.l1
21	XXII	1	1464298-1464247	10.5517/ccdc.6bt.s21.xxii.l1
21	XXII	2	1464348-1464377	10.5517/ccdc.6bt.s21.xxii.l2
21	XXIII	1	1464436-1464465, 1464491-1464520	10.5517/ccdc.6bt.s21.xxiii.l1
21	XXIV	1	1464466-1464490, 1464523-1464547	10.5517/ccdc.6bt.s21.xxiv.l1
21	XXV	1	1464409-1464433	10.5517/ccdc.6bt.s21.xxv.l1
21	XXVI	1	1464379-1464408	10.5517/ccdc.6bt.s21.xxvi.l1
22	XXII	1	1461680-1461710	10.5517/ccdc.6bt.s22.xxii.l1
22	XXIII	1	1461720-1461737	10.5517/ccdc.6bt.s22.xxiii.l1
22	XXIV	1	1461738-1461752	10.5517/ccdc.6bt.s22.xxiv.l1
22	XXV	1	1461753-1461762	10.5517/ccdc.6bt.s22.xxv.l1
22	XXVI	1	1461765-1461779	10.5517/ccdc.6bt.s22.xxvi.l1
23	XXII	1	1456102-1456201	10.5517/ccdc.6bt.s23.xxii.l1
23	XXII	2	1456202-1456220	10.5517/ccdc.6bt.s23.xxii.l2
23	XXIII	1	1458859-1458925, 1458927-1458959	10.5517/ccdc.6bt.s23.xxiii.l1
23	XXIII	2	1456077-1456101	10.5517/ccdc.6bt.s23.xxiii.l2
23	XXIV	1	1459099-1459132, 1459135-1459167, 1459169-1459201	10.5517/ccdc.6bt.s23.xxiv.l1
23	XXIV	2	1456221-1456239	10.5517/ccdc.6bt.s23.xxiv.l2
23	XXV	1	1456251-1456316, 1458825-1458858	10.5517/ccdc.6bt.s23.xxv.l1
23	XXVI	1	1459202-1459293	10.5517/ccdc.6bt.s23.xxvi.l1
23	XXVI	2	1456240-1456250	10.5517/ccdc.6bt.s23.xxvi.l2
24	XXIV	1	1454481-1454514, 1454516-1454581	10.5517/ccdc.6bt.s24.xxiv.l1

Continued on next page...

Table S13: CCDC numbers and digital object identifier of each of the submissions in the blind test, broken down by submission, target and list. Although the CCDC numbers for a given list are not necessarily a complete block, they do match the order of the submitted structures. Note that at most two submitted lists were considered in the blind test, but a third list for Submission 12 is included for reference (see SI document for more details).

Submission	Target	List	CCDC numbers	DOI
25	XXII	1	1455430-1455495, 1455498-1455531	10.5517/ccdc.6bt.s25.xxii.l1
25	XXII	2	1454271-1454290	10.5517/ccdc.6bt.s25.xxii.l2
25	XXIII	1	1454327-1454357	10.5517/ccdc.6bt.s25.xxiii.l1
25	XXIII	2	1455533-1455551	10.5517/ccdc.6bt.s25.xxiii.l2
25	XXIV	1	1454415-1454439, 1454448-1454472	10.5517/ccdc.6bt.s25.xxiv.l1
25	XXV	1	1454395-1454414	10.5517/ccdc.6bt.s25.xxv.l1

References

Ambrosetti, A., Reilly, A. M., DiStasio Jr., R. A. and Tkatchenko, A. (2014), *J. Chem. Phys.* **140**, 18A508.

Apostolakis, J., Hofmann, D. W. M. and Lengauer, T. (2001), *Acta. Cryst. A* **57**, 442–450.

Becke, A. D. (1988), *Phys. Rev. A* **38**, 3098–3100.

Blum, V., Gehrke, R., Hanke, F., Havu, P., Havu, V., Ren, X., Reuter, K. and Scheffler, M. (2009), *Comput. Phys. Commun.* **180**, 2175–2196.

Case, D. H., Campbell, J. E., Bygrave, P. J. and Day, G. M. (2016), *J. Chem. Theory Comput.* **12**, 910–924.

Clark, S. J., Segall, M. D., Pickard, C. J., Hasnip, P. J., Probert, M. I. J., Refson, K. and Payne, M. C. (2005), *Z. Kristallogr.* **220**, 567–570.

Deij, M. A., ter Horst, J. H., Meekes, H., Jansens, P. and Vlieg, E. (2007), *J. Phys. Chem. B* **111**, 1523–1530.

Dovesi, R., Orlando, R., Erba, A., Zicovich-Wilson, C. M., Civalleri, B., Casassa, S., Maschio, L., Ferrabone, M., De La Pierre, M., D'Arco, P., Noël, Y., Causà, M., Rérat, M. and Kirtman, B. (2014), *Int. J. Quantum Chem.* **114**, 1287–1317.

Dzyabchenko, A. (2008), *Russ. J. Phys. Chem. A* **82**, 1663–1671.

Giannozzi, P., Baroni, S., Bonini, N., Calandra, M., Car, R., Cavazzoni, C., Ceresoli, D., Chiarotti, G. L., Cococcioni, M., Dabo, I., Corso, A. D., de Gironcoli, S., Fabris, S., Fratesi, G., Gebauer, R., Gerstmann, U., Gougoussis, C., Kokalj, A., Lazzeri, M., Martin-Samos, L., Marzari, N., Mauri, F., Mazzarello, R., Paolini, S., Pasquarello, A., Paulatto, L., Sbraccia, C., Scandolo, S., Sclauzero, G., Seitsonen, A. P., Smogunov, A., Umari, P. and Wentzcovitch, R. M. (2009), *J. Phys.: Condens. Matter* **21**, 395502.

Goto, H. and Osawa, E. (1989), *J. Am. Chem. Soc.* **111**, 8950–8951.

Goto, H. and Osawa, E. (1993), *J. Chem. Soc., Perkin Trans. 2* pp. 187–198.

Grimme, S., Antony, J., Ehrlich, S. and Krieg, H. (2010), *J. Chem. Phys.* **132**, 154104.

Habgood, M., Sugden, I., Kazantsev, A. V., Adjiman, C. S. and Pantelides, C. C. (2015), *J. Chem. Theory Comput.* **11**, 1957–1969.

Hofmann, D. W. (2010), Data Mining in Organic Crystallography, *in* D. W. M. Hofmann and L. N. Kuleshova, eds, 'Data Mining in Crystallography', Vol. 134 of *Structure and Bonding*, Springer Berlin Heidelberg, pp. 37–58.

Holden, J., Ammon, H., Du, Z., Prasad, S., Wells, E. and Albu, N. (2014), 'Structure predictions with MOLPAK and PMIN or DMACRYS'. University of Maryand.

Karfunkel, H. and Gdanitz, R. (1992), *J. Comput. Chem.* **13**, 1171–1183.

Kazantsev, A., Karamertzanis, P., Pantelides, C. and Adjiman, C. (2010), Ab Initio Crystal Structure Prediction for Flexible Molecules, *in* '20th European Symposium of Computer Aided Process Engineering', Elsevier Science BV, pp. 817–822.

Kazantsev, A. V., Karamertzanis, P. G., Adjiman, C. S. and Pantelides, C. C. (2011), *J. Chem. Theory Comput.* **7**, 1998–2016.

Kendrick, J., Leusen, F. J. J. and Neumann, M. A. (2012), *J. Comput. Chem.* **33**, 1615–1622.

Kim, S., Orendt, A. M., Ferraro, M. B. and Facelli, J. C. (2009), *J. Comput. Chem.* **30**, 1973–1985.

de Klerk, N. J. J., van den Ende, J., Bylsma, R., Grančič, P., de Wijs, G. A., Cuppen, H. M. and Meekes, H. (2016), *Cryst. Growth Des.* **16**, 662–671.

Kresse, G. and Furthmüller, J. (1996), *Phys. Rev. B* **54**, 11169–11186.

Lee, C., Yang, W. and Parr, R. G. (1988), *Phys. Rev. B* **37**, 785–789.

Lyakhov, A. O., Oganov, A. R., Stokes, H. T. and Zhu, Q. (2013), *Comp. Phys. Comm.* **184**, 1172–1182.

Misquitta, A. J., Podeszwa, R., Jeziorski, B. and Szalewicz, K. (2005), *J. Chem. Phys.* **123**, 214103.

Neumann, M. A. and Perrin, M.-A. (2005), *J. Phys. Chem. B* **109**, 15531–15541.

Nyman, J. and Day, G. M. (2015), *CrystEngComm* **17**, 5154–5165.

Perdew, J. P., Burke, K. and Ernzerhof, M. (1996), *Phys. Rev. Lett.* **77**, 3865–3868.

Price, S. L., Leslie, M., Welch, G. W. A., Habgood, M., Price, L. S., Karamertzanis, P. G. and Day, G. M. (2010), *Phys. Chem. Chem. Phys.* **12**, 8478–8490.

Spek, A. L. (2009), *Acta Cryst. D* **65**, 148–155.

Tao, J., Perdew, J. P., Staroverov, V. N. and Scuseria, G. E. (2003), *Phys. Rev. Lett.* **91**, 146401.

Tkatchenko, A., DiStasio Jr., R. A., Car, R. and Scheffler, M. (2012), *Phys. Rev. Lett.* **108**, 236402.

Tkatchenko, A. and Scheffler, M. (2009), *Phys. Rev. Lett.* **102**, 073005.

Todorov, I. T., Smith, W., Trachenko, K. and Dove, M. T. (2006), *J. Mater. Chem.* **16**, 1911–1918.

Tuckerman, M. E., Yarne, D., Samuelson, S. O., Hughes, A. L. and Martyna, G. J. (2000), *Comp. Phys. Comm.* **128**, 333–376.

van Eijck, B. P. (2015), `http://www.crystal.chem.uu.nl/~vaneyck/upack.html`.

van Eijck, B. P. and Kroon, J. (1999), *J. Comput. Chem.* **20**, 799–812.

van Eijck, B. P. and Kroon, J. (2000), *Acta. Cryst. B* **56**, 535–542.

Wang, Y., Lv, J., Zhu, L. and Ma, Y. (2012), *Comput. Phys. Commun.* **183**, 2063–2070.

Zhu, Q., Oganov, A. R., Glass, C. W. and Stokes, H. T. (2012), *Acta Cryst. B* **68**, 215–226.
