# Supplementary material for: Report on the sixth blind test of organic crystal structure prediction methods
Source: Acta Crystallogr B Struct Sci Cryst Eng Mater. 2016 Aug 1;72(Pt 4):439–59. doi: 10.1107/S2052520616007447 (PMC4971545; doi:10.1107/S2052520616007447)
Supplement: Supplementary file 2 [file b-72-00439-sup2.zip › Group_12_Marom/Note.pdf]

The “edited_XXII…” cifs have the same geometry as those originally submitted in the blind test but have been corrected to remove distortions that resulted from imposing symmetry with pymatgen.

The cif file “PBE0_MBD_Marom.cif” (and the corresponding edited file “edited_PBE0_MBD_Marom.cif”) is an extra set of structures calculated with PBE0+MBD. These were not submitted in the blind test but are included here for reference.

Anthony Reilly, June 2016
