# Supplementary material for: Report on the sixth blind test of organic crystal structure prediction methods
Source: Acta Crystallogr B Struct Sci Cryst Eng Mater. 2016 Aug 1;72(Pt 4):439–59. doi: 10.1107/S2052520616007447 (PMC4971545; doi:10.1107/S2052520616007447)
Supplement: Supplementary file 3 [file b-72-00439-sup3.zip › Group_01_Chadha_Singh.pdf]

Sixth Blind Test of Organic Crystal –Structure Prediction Methods

Renu Chadha*, Pawanpreet Singh
University Institute of Pharmaceutical Sciences,
Panjab University, Chandigarh, India.
Corresponding author E-mail ID: renukchadha@rediffmail.com

Supporting Information

Computational Resources:

All calculations of Crystal structure prediction and energy minimization were performed on Intel® Xenon® (3.20 GHz) (Quadra core) 64 bit HP Z230 SFF workstation using Material Studio 7.0 (Biovia San Diego, CA 92121 USA). The total time used for computational searches for different molecules were given in **Table 1** although parts of the calculations were performed on CPUs with lower performance.

Description of the methodologies used:

1. Geometry optimization:

For all the molecules under study, initial input geometry was done using Smart algorithm of Forcite module at Ultrafine settings with convergence tolerance energy of 2×10^{-5} kcal/mol, force of 0.001 kcal/mol/Å, and displacement of 1×10^{-5} Å with maximum number of iterations of 500 for an independent optimization. The force field used was COMPASS. The optimized low energy conformations were reoptimized by a molecular dynamics simulation using the same module (simulation length of 5 ps with a 1 fs time step at a temperature of 298 K, taking conformations every 5000 steps). These optimized conformations of blind test molecules were used as the starting point for crystal structure prediction calculation using the Materials Studio Polymorph module.

Energy parameters involved:

Forcefield	: COMPASS (Version 2.8)
Charges	: Forcefield assigned

Electrostatic terms:

Summation method : Atom based

Truncation method : Cubic spline

Cutoff distance : 18.5 Å

Spline width : 1 Å

Buffer width : 0.5 Å

van der Waals terms:

Summation method : Atom based

Truncation method : Cubic spline

Cutoff distance : 18.5 Å

Spline width : 1 Å

Buffer width : 0.5 Å

Table 1: Brief description of forcefield applied and the space groups searched for CSP.

Target	Forcefield used in CSP	Number of Space groups studied	Total computational cost, (CPU hours)
XXII	COMPASS	230	350
XXIII	COMPASS	230	450
XXIV	Not attempted		
XXV	Not attempted		
XXVI	COMPASS	230	600

2. Generation of putative crystal structures:

This was carried out using the polymorph module in Materials Studio. Sampling was performed in all of the 230 space groups (as mentioned in **Table 1**). The polymorph predictor was set to its default fine setting (this sets the simulated annealing algorithm to a temperature range of 300.0-150000.0 K with a heating factor of 0.025, requiring 14 consecutive steps to be accepted before cooling and a maximum of 10000 steps) with the force field COMPASS. The polymorph predictions were repeated four times for each starting conformation to ensure adequate sampling of the crystal configurations.

CSP Methodology used during computational studies:

Prediction protocol: (1) Packing (2) Clustering (3) Geometry Optimization and (4) Clustering

(1) ---- Packing parameters ----

Search algorithm	: MC Simulated Annealing
Maximum number of steps	: 10000
Explore torsions	: No
Pre-optimize structures	: No
Steps to accept before cooling	: 14
Minimum move factor	: 0.1000E-09
Heating factor	: 0.02500
Maximum temperature	: 150000.0 K
Minimum temperature	: 300.0 K

(2) ---- Cluster analysis parameters ----

Cluster grouping	: Forcefield type
Cutoff	: 7.000
Number of bins	: 140
Tolerance	: 0.1100
Maximum number of clusters	: 2000

(3) ---- Geometry optimization parameters ----

Algorithm	: Smart

Convergence tolerance:

Energy	: 2e-005 kcal/mol
Force	: 0.001 kcal/mol/ Å
Stress	: 0.001 GPa
Displacement	: 1e-005 Å
Maximum number of iterations	: 500
External pressure	: 0 GPa
Motion groups rigid	: NO
Optimize cell	: YES

Energy parameters ----

Forcefield	: COMPASS (Version 2.8)
Charges	: Forcefield assigned

Electrostatic terms:

Summation method	: Ewald
Accuracy	: 1e-005 kcal/mol
Buffer width	: 0.5 Å

van der Waals terms:

Summation method	: Atom based
Truncation method	: Cubic spline
Cutoff distance	: 18.5 Å
Spline width	: 1 Å
Long range correction	: YES
Buffer width	: 0.5 Å

3. Ranking of the generated structures:

Minimizations and subsequent clustering were further performed using the "Ultrafine" settings. The predicted structures were ranked and sorted based on their energies.

.
