# Supplementary material for: Report on the sixth blind test of organic crystal structure prediction methods
Source: Acta Crystallogr B Struct Sci Cryst Eng Mater. 2016 Aug 1;72(Pt 4):439–59. doi: 10.1107/S2052520616007447 (PMC4971545; doi:10.1107/S2052520616007447)
Supplement: Supplementary file 3 [file b-72-00439-sup3.zip › Group_02_Cole_Post_Analysis.pdf]

Crystal Structure Prediction Blind Test 2015

Jason C. Cole, Patrick McCabe, Murray Read, Anthony M. Reilly and Gregory P. Shields

The Cambridge Crystallographic Data Centre, 12 Union Road, Cambridge, CB2 1EZ, United Kingdom

Crystal Structure Generation

Molecular model

The geometries of the input molecules were generated from SMILES strings (Daylight, 2008) using the chemical structure generator CORINA (Sadowski & Gasteiger, 1993; Sadowski *et al*., 1994).

The molecular structure of **XXII** was optimised at the MP2 level with the 6-311+G* basis set in the Spartan14 package (Wavefunction, 2014) in order to give a more accurate geometry around the sulphur atoms in the 6-membered ring.

Molecules **XXII** and **XXV** were further optimised using the CSD knowledge-based force-field (CSD-KBF) (*McCabe et al*., 2015). This force field uses the data in the CSD Mogul knowledge base (Bruno *et al*., 2004), modified to include rotamer distributions (Taylor *et al.*, 2014), represented using kernel density estimation (McCabe *et al.*, 2014). For the acid molecule in **XXV** (Figure 2), conformers were generated using the CSD conformer generator (Korb *et al.*, 2015) from the CSD-KBF starting geometry. The conformer probabilities show a very strong preference for the molecule to adopt a planar geometry. In addition, a further 12 lower-probability conformers with the carboxylic acid and nitro groups twisted less than 35° from the plane of the aromatic ring were included in the shortlist of 13 conformers using the filters listed in Table 2.

An initial list of 200 conformers for molecule **XXIII** and **XXVI** were generated using the CSD Conformer Generator (CCDC, 2014) from the Corina starting geometry. The geometry of the conformers was subsequently minimised using the CSD-KBF. Conformers of **XXIII** were filtered according to the criteria listed in Table 1, derived from manual inspection of Mogul torsion histograms and intramolecular hydrogen bond propensity scores (Galek *et al*., 2009), to produce a shortlist of 52 conformers. Similarly, conformers of **XXVI** were filtered according to the criteria listed in Table 3 to produce a shortlist of 43 conformers.

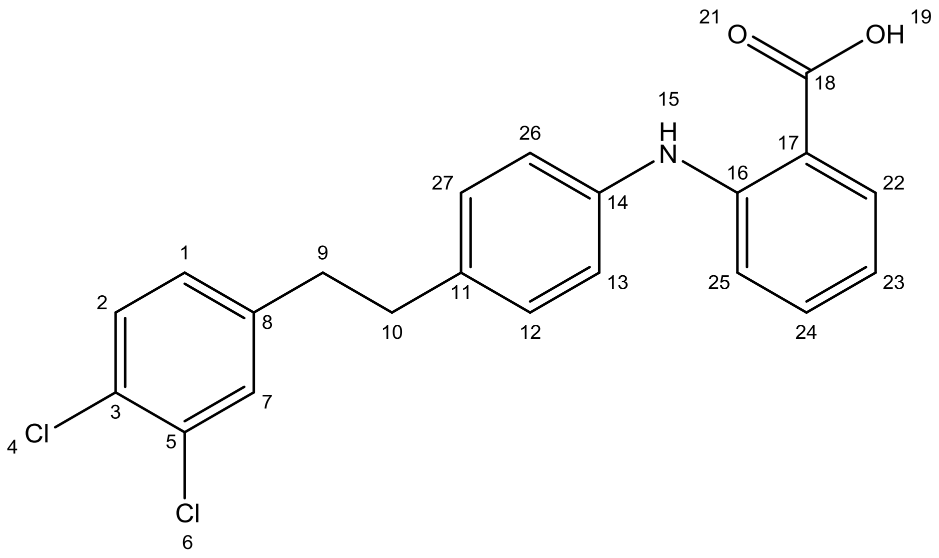

Figure 1. Atom numbering scheme for **XXIII**.

Table 1. Conformer filters for **XXIII**.

Atom labels	Allowed torsion angle range(s) / °
O21 C18 C17 C16	-70.0 70.0
C11 C10 C9 C8	-180.0 -150.0; 150.0 180.0
C27 C11 C10 C9	-130.0 -50.0; 50.0 130.0
C10 C9 C8 C1	-130.0 -50.0; 50.0 130.0

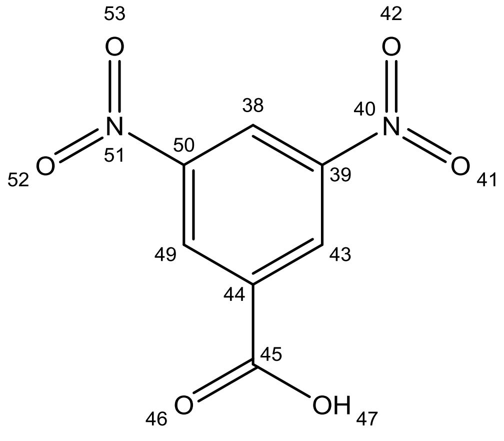

Figure 2. Atom numbering scheme for the acid molecule in **XXV**.

Table 2. Conformer filters for **XXV**.

Atom labels	Allowed torsion angle range(s) / °
O41 N40 C39 C43	-35 35; 145 180; -180 -145
O42 N40 C39 C38	-35 35; 145 180; -180 -145
O52 N51 C50 C49	-35 35; 145 180; -180 -145
O53 N51 C50 C38	-35 35; 145 180; -180 -145
O47 C45 C44 C43	-35 35; 145 180; -180 -145
O46 C45 C44 C49	-35 35; 145 180; -180 -145

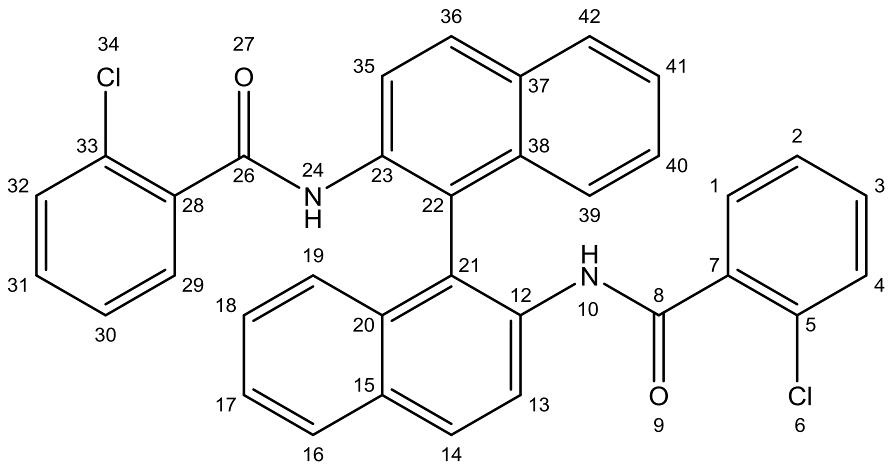

Figure 3. Atom numbering scheme for **XXVI**.

Table 3. Conformer filters for **XXVI**.

Atom labels	Allowed torsion angle range(s) / °
C5 C7 C8 N10	-150 -30; 30 150
C33 C28 C26 N24	-150 -30; 30 150
C12 N10 C8 C7	-180 -170; 170 180
C23 N24 C26 C28	-180 -170; 170 180

Structure model

The structure model is composed of cell lengths and angles (6 variables), molecule position and orientation (6 variables per independent molecule). Cell angles are fixed if constrained by space-group symmetry. No restrictions are placed on the allowed range of variables in local optimisation.

Given the molecules that form the asymmetric unit, and a starting cell with lengths, angles and a space group, a central unit cell is constructed from the asymmetric unit molecules by applying the space-group symmetry operators.

Surrounding unit cells are added by a breadth-first search of neighbouring unit cells until there are no more unit cells that contain atoms within 12 Å of the asymmetric unit atoms. During local optimisation, if more unit cells are found to contain atoms within 12 Å of the asymmetric unit atoms, they are also included and the optimisation is restarted from its current position.

Potentials

The intermolecular score is calculated on a per-molecule basis using intermolecular atom-atom interaction Buckingham potentials:

$$V_B(r) = Ae^{-kr} - \frac{C}{r^6} \tag{1}$$

Various modifications were made to improve computation time and stability.

To keep the potential finite ranged we calculate the approximate long range r value (r_d) where $V(r_d) = 10^{-3}$ and smoothly switch off V beyond this value (*e.g.* 11.6 Å for carbon-carbon interactions), over a range of 1 Å so that

$$\begin{aligned} V(r) &= V_B(r)\frac{1}{2}\big(1+\cos\big((r-r_d)\pi\big)\big) & r_d \le r \le r_d + 1 \\ &= 0 & r > r_d + 1 \end{aligned} \tag{2}$$

To prevent the well-known divergence of $V_B(r)$ at short range, we linearly extrapolate from the point of inflection (r_i) on the repulsive wall, located between the local maximum at short range and the well minimum, where V_B has slope $V_B'(r_i)$ so that

$$V(r) = V_B(r_i) + V_B'(r_i)(r - r_i) \qquad r \le r_i \tag{3}$$

The parameters *A*, *k* and *C* are based on the Unimol intermolecular force-field given in Filippini & Gavezzotti (1993) and Gavezzotti & Filippini (1994). The parameters were tuned to improve their ability to replicate CSD crystal structures. The performance of a set of parameters was evaluated by minimising 100-200 CSD crystals that used only those parameters, using a drift index (Gavezzotti, 2011) after minimisation as a badness score. These sets of parameters were then optimised with LBFGS (Liu & Nocedal, 1989) to minimise this badness score. Parameter tuning used approximately 25 hours of 15 CPU cores, 375 CPU hours in total.

Candidate cell selection

A number of candidate cells were generated for each input molecule, using the CSD as a source of candidate cells. A CSD entry was only considered as a candidate cell when it contained the same number of molecule types as the number of input molecule types. In addition, where a CSD entry has a component molecule on a special position, it is only considered for input molecule conformers that have the required molecular symmetry.

The conformers of the input molecule were ultrafast shape recognition (USR) (Ballester & Richards, 2007) shape matched against molecules in the CSD. The USR match score was converted to a shape-match log probability of finding a better shape match P_{shape} by interpolating in a table of USR match score log probabilities specific to the molecule's atom count. This was combined with the conformer torsion log probability P_{torsion} to give a score for the CSD molecule as a shape analogue for the conformer: $score = P_{\text{torsion}} - P_{\text{shape}}$. The best conformer for each type of component molecule in the CSD entry was selected by score. The CSD entry was then given a score as the sum of the component type scores. The highest scoring CSD entries' cells were then scaled, so that the crystal

packing ratio of the entry is preserved with the new molecules, to create candidate cells for optimisation, and the conformer matched was recorded.

Molecule **XXII** was assumed to be a *Z*' = 1 system with one molecule in the asymmetric unit.

Molecule **XXIII** was assumed to be a *Z*' = 1 or 2 system with one or two molecules in the asymmetric unit.

Molecule **XXV** was assumed to be a *Z*' = 1 system with one molecule of each component in the asymmetric unit. Candidate cells with more than one of either component were filtered out. It was assumed that the protonation state of the molecules is as shown, although CSD analysis of structures of the base molecule and analogues show that proton transfer of an acid proton to the ternary nitrogen could occur.

Molecule **XXVI** was assumed to be a *Z*' = 1 or 2 system with one or two molecules in the asymmetric unit.

Conformational flexibility

Rotatable bonds are defined using SMARTS strings (Daylight, 2008) in a configuration file. The CSD knowledge-based force field (CSD-KBF, *McCabe et al*., 2015) is used to model the torsional degrees of freedom in conjunction with a clash term (*McCabe et al*., 2015). In local optimisation, no constraints are placed on the torsion angles and they are allowed to optimise freely subject to the CSD-KBF function. The CSD-KBF and clash terms were only calculated for rotatable bonds, with a weight relative to the intermolecular contribution to the score of 10.

Molecule **XXII** was treated as a completely rigid molecule.

Molecule **XXIII** was treated with 6 rotatable bonds.

Molecule **XXV** the acid molecule was treated with 3 rotatable bonds; the hydroxyl conformation was fixed. The base molecule was treated as rigid.

Molecule **XXVI** was treated with 7 rotatable bonds.

Local optimisation

Local gradient-based minimisation was carried out using an implementation of the Limited Memory BFGS method (Liu & Nocedal, 1989). For implementation efficiency, gradients are calculated using the CppAD automatic differentiation library (Bell, 2015).

Global optimisation

Global searching was performed by using CSD entries, which were the source of the generated candidate cell list, as structural analogues for the generation of crystal structures, then locally optimising the crystal structures generated from those structural analogues. To generate crystal structures, the CSD entry structural analogues were scaled to match the volume of the input molecules. Then the input molecules were overlaid using the CCDC ligand overlay tool (Taylor *et al.*, 2012) on the CSD molecules to position the input molecules, as rigid molecules, in the correct place in the structural analogue. This was repeated with an inverted copy of the CSD entry. The inverted structural analogue was then used only if the molecule overlay gave a better fit. To complete

construction of the crystal structure from the structural analogue, the CSD molecules in the structural analogue were replaced with the overlaid input molecules.

Table 4. Computational resources for structure generation and optimisation on an Intel® Core™ i7-2700 3.5 GHz PC.

Molecule	Number of structures	Total CPU hours
**XXII**	1876	6
**XXIII***	8976	538
**XXV**	2330	46
**XXVI**	4920	246

*We note that the distribution of time spent is currently highly skewed. The average time per structural analogue is 3.6 minutes, while the median time is just 1.7 minutes; almost half the elapsed time was spent generating 500 of the 8,976 putative structures.

Structure Clustering

Structures were sorted initially on score. Simulated powder patterns (Macrae *et al.*, 2006) were compared for candidate structures with a score difference of 10 or less. Structures with powder pattern similarity of 90% or greater (de Gelder *et al.*, 2001) were compared using COMPACK packing similarity (Chisholm & Motherwell, 2005) with distance tolerance of 20%, an angle tolerance of 20°, a cluster size of 15 molecules and RMSD tolerance of 0.5 Å. Candidate structures equivalent within these criteria were considered to be duplicates.

Crystal Structure Filtering

Numeric descriptor definitions

Formal definitions

A "vdW normalised distance" between two atoms a1 and a2 is defined by equation (4) below:

$$nd(a_1 \cdots a_2) = d(a_1 \cdots a_2) - r_{vdw}(a_1) - r_{vdw}(a_2) \qquad (4)$$

where d(a1…a2) is the distance between atoms a1 and a2, and r_{vdw} is the Van der Waals radius of the respective atom.

Intermolecular void volume calculations

Void volumes were calculated using the void implementation as presented in Mercury (Macrae *et al.,* 2006) *via* the CSD Python API. Two different calculations are performed with differing probe radii (1.2 Å and 0.6 Å). A grid spacing of 0.2 Å is used to achieve a more precise estimate of the amount of void space in a given structure.

Hydrogen bond scoring

Hydrogen bonds were scored using a linear interpolation function as defined below. For each possible combination of a donor atom 'D', connected hydrogen atom 'H' and acceptor atom 'A', a score S was calculated defined by:

$$S(D - H \cdots A) = b(D - H \cdots A) \times a(D - H \cdots A) \qquad (5)$$

where the angular term a(D-H···A) is defined as

$$a(D-H\cdots A)=\begin{cases}\left(\frac{\widehat{DHA}}{90}\right)-1 & \widehat{DHA}\geq 90\\ 0 & \widehat{DHA}<90\end{cases} \quad (6)$$

and the distance term is defined as

$$b(D-H\cdots A)=\begin{cases}1 & nd(H\cdots A)<0.2\\ \frac{0.5-nd(H\cdots A)}{0.3} & 0.2\leq nd(H\cdots A)\leq 0.5\\ 0 & nd(H\cdots A)>0.5\end{cases} \quad (7)$$

nd(H···A) is the vdW normalised distance (4) between the hydrogen, H and acceptor atom A.

Contact assessment

For intramolecular contact assessment, only atoms pairs separated by at least three bonds were scored.

For each pair of atoms, contacts were scored as below:

$$C(a_1\cdots a_2)=nd(a_1\cdots a_2)+T(a_1\cdots a_2) \quad (8)$$

where *T* is an atom-pair specific tolerance. For donor–acceptor pairs *T* = 0.3 Å. For acceptor, hydrogen pairs *T* = 0.4 Å in cases where the hydrogen was bound to a carbon. When the hydrogen was bound to a stronger donor atom (N, O, S) the tolerance *T* = 0.9 Å was used. For all other atom pairs T took the value of 0.0 Å.

Two composite scores were then calculated:

1. The number of pairs of atoms that had $C(a_1\cdots a_2)<-0.3$
2. $Clash=\sum^{pairs(a_1,a_2)}(min(0.0,C(a_1\cdots a_2)))^2$

Filter evaluation

Evaluation set

A random subset of 1254 CSD entries (defined as the CSD Native Set, see CSD_Native_Set_Refcodes.gcd) was used to evaluate the expectation ranges of given filters. These structures all fulfil the requirements given below in Table 5.

Table 5. Requirements for the structures used to evaluate structure filters.

Criterion	Requirement
Allowed elements	C, H, N, O, F, P, S, Cl, Br, I
Z'	1
Disorder	None
Recorded R-factor (%)	In range 0, 5
Number of components	1
Rotatable bond count	In range 0, 15
Molecular weight	In range 100.0, 1000.0
Atom count	In range 20, 100
Acceptor count	In range 0, 8
Donor count	In range 0, 7

All the Native Set entries were locally optimised using the as-published Unimol (6-exp) molecular force field (Filippini & Gavezzotti, 1993 and Gavezzotti & Filippini, 1994) to give a second set of structures defined as the CSD Unimol Set. The distributions of descriptor values were analysed and compared between sets to establish cut-off values that could be used for filtering solutions generated by CSP methodologies.

A third set was generated by locally optimising the Native Set entries with newly tuned Unimol (6-exp) parameters, which were optimised to reproduce CSD structures.

The rationale for using the optimised structures is that the best result we can expect from a global-search technique would lie at the local minimum of the force field used that is closest to the observed crystal structure. Improvements to the scoring used should lead to convergence towards the values seen in CSD structures in future.

Void volumes

Distributions of void volumes (probe radius 0.6 Å) across structures in the CSD Native Set, the CSD Unimol Set and the CSD Unimol Tuned Parameter Set are shown in Figure 4.

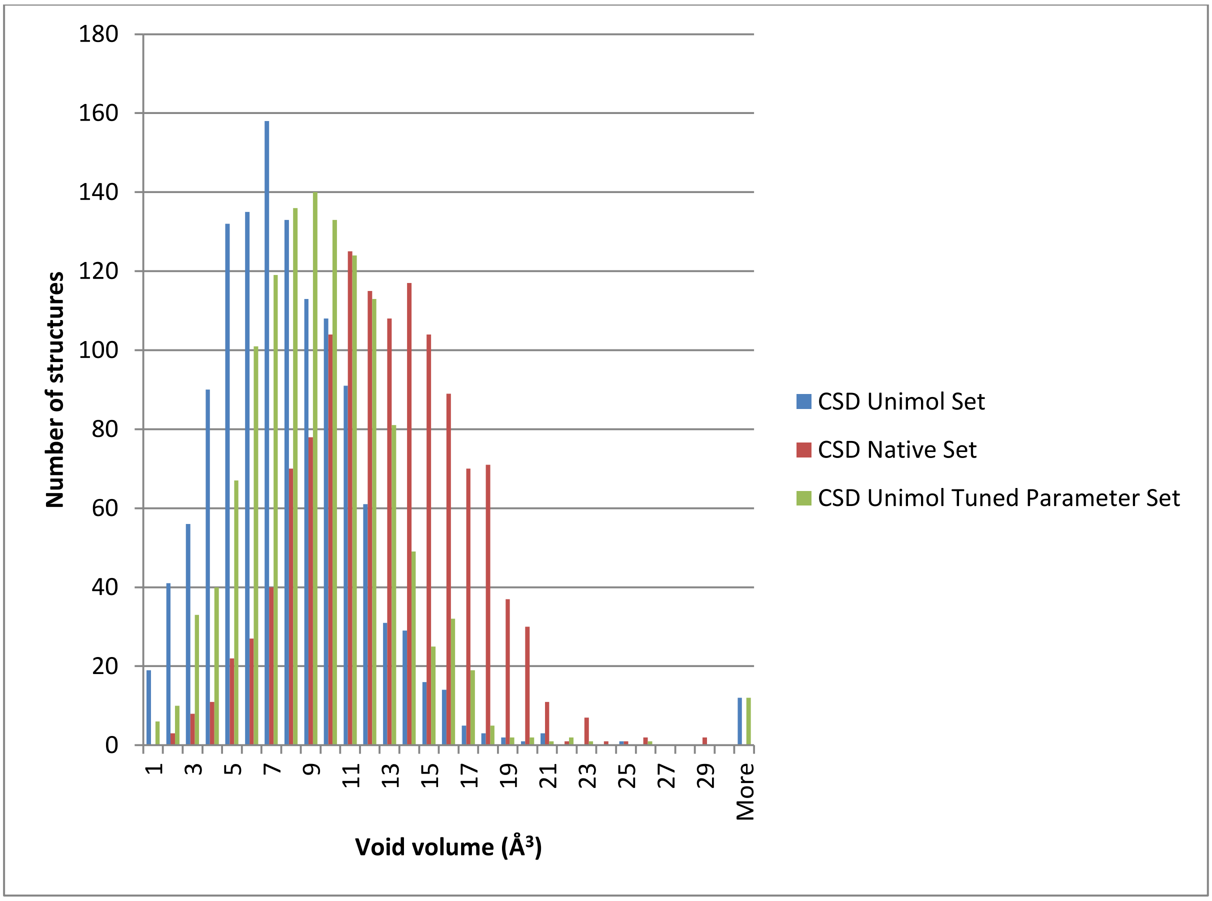

Figure 4. Distributions of void volumes.

As is apparent, local optimisation forces voids to minimise away from the observed CSD distribution; an expected effect given that CSD structures are habitually solved at temperatures significantly higher than 0 K. The 95th percentile for the CSD Native Set is 17.5 Å^3 and the 95th percentile for the locally optimised set using Unimol is 13.8 Å^3. Thus a reasonable void volume filter would be to remove all structures with a void volume (0.6 Å probe radius) greater than 13.8 Å^3 if using the original Unimol parameters.

The tuned parameters shift back towards the CSD distribution. The 95th percentile for that distribution lies at 15.3 Å^3, and therefore if using the tuned parameters in generation this should be set as the cut-off threshold.

Intermolecular contact distributions

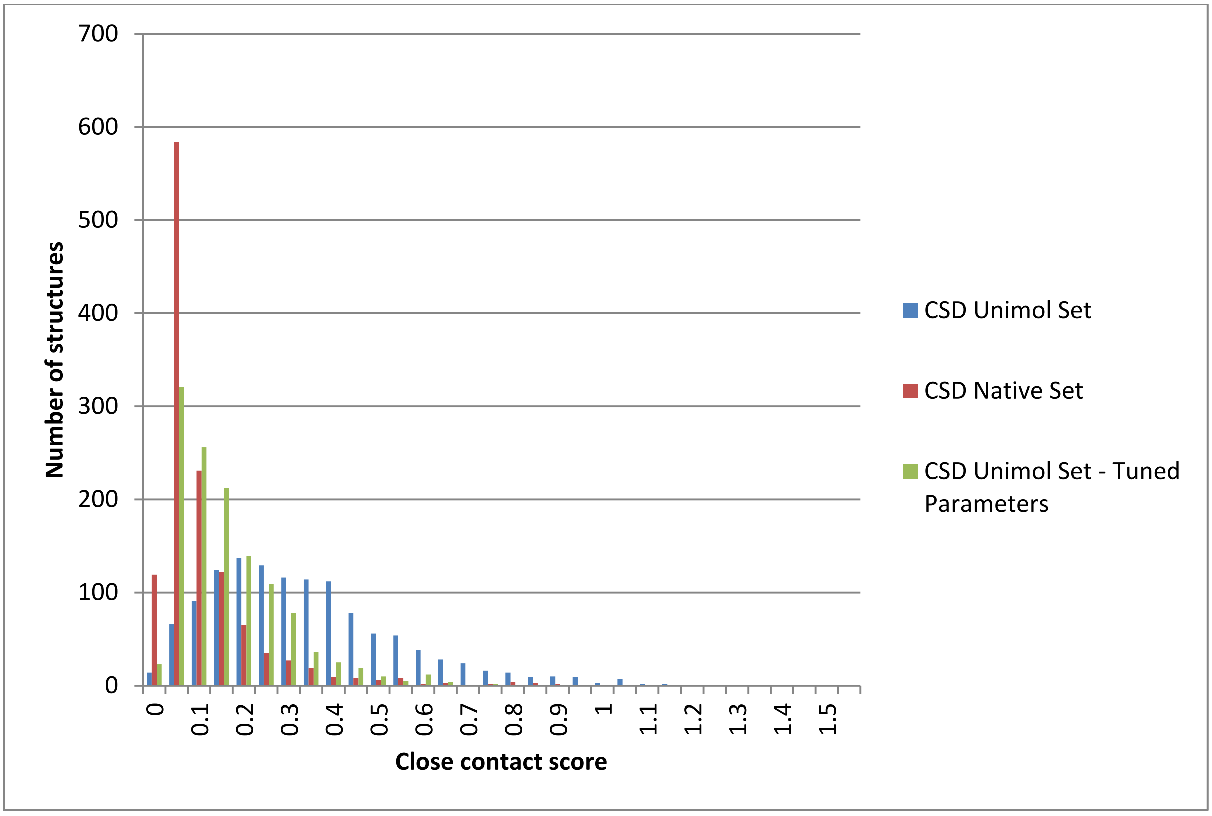

Figure 5. Close contact scores.

The difference between contact scores in the CSD Native and in the Unimol set is marked (Figure 5), and again reflects the tendency for the local optimiser to minimise void volume. The 95th percentile for the native set lies at 0.32 whereas for the Unimol set it lies at 0.72. For the tuned parameter set, the 95th percentile lies at 0.38.

The alternative view in Figure 6 shows the numbers of structures that have *N* contacts that fulfil $C(a1 \cdots a2) < -0.3$, i.e. genuine *individual* close contacts rather than a composite score.

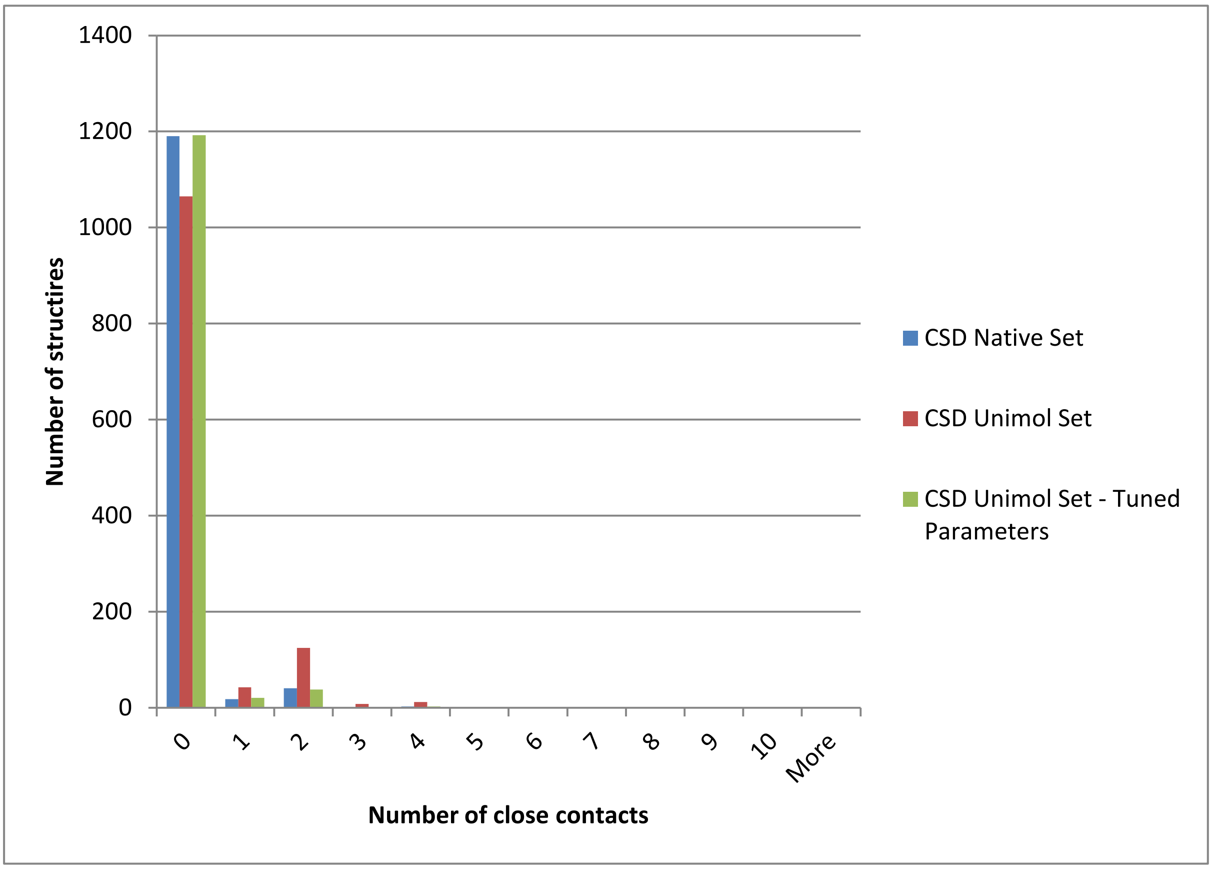

Figure 6. Number of close contacts.

99.6% of entries in the Native set contain no more than 2 close contacts, 96.3% contain no more than 1 close contact, and 94.8% contain no close contacts). For the Unimol set, the distribution is broader: 98.3% contain no more than 2 close contacts, but only 88.4% contain no more than 1 close contact. The close contact distribution for the tuned parameter set matches the CSD Native Set very closely. We note that, while our definition of 'C' attempts to correct for some allowable short contacts due to hydrogen bonding, we make no attempt to further correct for other possible close contacts in crystal lattices (for example; electrostatic attractions between ions and counter-ions).

The data above would suggest that a threshold of no more than 2 close contacts in the minimised structures would be a reasonable filter to use. Combining with a filter for close contact scores most structures with infeasible contacts should be eliminated. The close contact filter is set to the 95th percentile of either the Unimol distribution (0.72) or 0.38 if using the tuned parameter sets.

Hydrogen Bond Propensities

XXIII

For Molecule **XXIII**, the hydrogen bond propensity tool (Galek *et* al., 2009) suggests that the intra-molecular H-bond is highly likely (Table 7), and that dimerization of the COOH fragment is also the most likely intermolecular motif (Table 6).

Table 6. Intermolecular hydrogen bond propensities for molecule **XXIII** (atom numbering scheme shown in Figure 1).

Donor	Acceptor	Propensity	Lower bound	Upper bound
**O19**	O21	**0.50**	0.28	0.72
**N15**	O21	**0.24**	0.11	0.44
**O19**	O19	**0.07**	0.03	0.18
**O19**	N15	**0.03**	0.01	0.09
**N15**	O19	**0.02**	0.01	0.06
**N15**	N15	**0.01**	0.00	0.03

Table 7. Intramolecular hydrogen bond propensities for molecule **XXIII**.

Donor	Acceptor	Donor count	Propensity	Lower bound	Upper bound
**N15**	O21	2	**0.91**	0.91	0.91
**N15**	O19	2	**0.87**	0.87	0.87
**O19**	N15	2	**0.47**	0.47	0.47

XXV

For structure **XXV**, the propensity tool suggests that the tertiary nitrogen atoms in the larger fragment are not good acceptors for hydrogen bonds (Table 8).

Table 8. Intermolecular hydrogen bond propensities for structure **XXV** (atom numbering scheme shown in Figure 2).

Donor	Acceptor	Propensity	Lower bound	Upper bound
**O47**	O46	**0.39**	0.33	0.46
**O47**	O41	**0.20**	0.15	0.25
**O47**	O42	**0.20**	0.15	0.25
**O47**	O52	**0.20**	0.15	0.25
**O47**	O53	**0.20**	0.15	0.25
**O47**	O47	**0.06**	0.04	0.08
**O47**	N15	**0.02**	0.01	0.02
**O47**	N6	**0.02**	0.01	0.02

The propensity analysis would suggest that the most likely interaction is the formation of a carboxylic acid dimer. Alternatively, the carboxylic OH donor could hydrogen bond to a nitro oxygen acceptor.

XXVI

Propensity analysis for Molecule **XXVI** suggests that possibly 2 hydrogen bonds are likely in the observed structure (Table 9). The analysis does not distinguish between the various hydrogen bonds as the underlying molecule has topological symmetry.

Intramolecular analysis suggests that the NH is somewhat likely to form an NH···Cl interaction. This is represented in the torsional preferences of the molecule and so is already implicit in the selection of conformers for this molecule.

Table 9. Intermolecular hydrogen bond propensities for Molecule **XXVI** (atom numbering scheme shown in Figure 3).

Donor	Acceptor	Propensity	Lower bound	Upper bound
**N10**	O27	**0.37**	0.15	0.67
**N10**	O9	**0.37**	0.15	0.67
**N24**	O27	**0.37**	0.15	0.67
**N24**	O9	**0.37**	0.15	0.67
**N10**	Cl34	**0.01**	0.00	0.03
**N10**	Cl6	**0.01**	0.00	0.03
**N24**	Cl34	**0.01**	0.00	0.03
**N24**	Cl6	**0.01**	0.00	0.03

Table 10. Intramolecular hydrogen bond propensities for Molecule **XXVI**.

Donor	Acceptor	Donor count	Propensity	Lower bound	Upper bound
**N10**	Cl6	2	**0.71**	0.71	0.71
**N24**	Cl34	2	**0.71**	0.71	0.71

System-specific substructure-driven filters

To allow for customized filtering of solutions, a 3D substructure searching filtering system was written. The system allows for 3D SMARTS codes to express desired substructures alongside the definition of geometric features between substructures. By identifying where there are one or more hits for a given search, it is then possible to select or remove structures based on the presence or absence of a given feature. Specific searches were defined for each test system and used to filter the generated structures.

Crystal Structure Selection

XXII

Selection filters were calculated for structure **XXII**. One specific substructure filter was developed to identify possible stacking motifs of the butenedinitrile fragment, though in practice this filter was not used in selecting a subset. The initial set of structures generated was reduced from 1875 to 1334 structures by clustering to remove duplicates.

The clustered structures were filtered down to the set of structures with a void-volume fraction of less than 20% (determined using a probe radius of 0.6 Å), no more than 2 close contacts and a close contact score of less than 0.5. The top 100 structures by score were submitted.

XXIII

8,976 structures were generated initially from 10,000 shape-selected structural analogues in the CSD. The high number used was to ensure a reasonable sampling across conformational space. *Z'*=1 and *Z'*=2 structural analogues were included.

Due to the large number of putative structures, selection filtering was performed first. The structures were filtered down to the set of structures with a void-volume fraction of less than 20% (determined using a probe radius of 0.6 Å) no more than 2 close contacts per molecule and a close contact score of less than 0.5 per molecule.

Structures were further filtered based on the quality of the hydrogen bonds in the structure. Only structures that contained at least one OH···O=C 'good quality' hydrogen bond per molecule were included, as the propensity calculation for this system suggested that this hydrogen bond would predominate in molecules of this type. This yielded 354 structures, which were then clustered for structural similarity, reducing the set to 298 structures. The top 100 structures by score were submitted.

XXV

Structural clustering had limited effect on Molecule **XXV**, reducing down the set of structures from 2,330 to 2,294. Only 4 structures in the top 100 ranked by score alone were duplicates of other high scoring structures.

Propensity analysis indicated that the carboxylate group was twice as likely to form a hydrogen bond to itself over one of the nitro groups. Consequently, the clustered structures were filtered down to remove structures where no carboxylate dimer was observed. This reduced the set from 2292 to 233 structures.

Of these structures, only structures with a void-volume fraction of less than 20% (determined using a probe radius of 0.6 Å), no more than 2 close contacts and a close contact score of less than 0.5 were included. This yielded 101 structures. The top 100 by score were then included in the final set.

XXVI

Structures were generated from 5,000 CSD based structural analogues, yielding 4,930 structures. Clustering reduced the number of structures to 4595.

The filter thresholds applied were somewhat softer than in the other systems due to the lower packing coefficients of the solutions generated; only structures with a void volume of less than 30% (determined using a probe radius of 0.6 Å), a maximum of 2 close contacts per molecule and a close contact score per molecule of less than 0.5 were included. While the hydrogen bond propensity analysis for this molecule suggests a tendency for an intermolecular H-bond between amide fragments, the nature of the conformations of this molecule make this challenging. We note that hydrogen bonding is not present in the closest analogue molecule in the CSD (KIRGEX) and so elected to not focus solely on hydrogen-bonded structures for this system.

To focus on the best conformations, final structures that had an intramolecular close contact score greater than 1.5 were removed. This yielded 1058 structures. The top 100 by score were then included in the final set.

Post analysis

Analysis of the experimentally-observed crystal structures for **XXII**, **XXIII**, **XXV** and **XXV** showed that we were not successful in predicting any of the molecules in our top 100 ranked lists according to the blind test criteria. The reasons for this are summarised below.

Conformer prediction

The experimentally observed conformations were compared with the input conformations used in our predictions and the results are summarised in Table 11. In all cases except for one molecule in polymorph (c) of **XXIII** and for molecule **XXVI**, one of the top four ranked conformers had a heavy-atom RMSD within 0.5Å of the experimentally observed conformation.

Table 11. Comparison of generated and experimentally observed conformers by rank and heavy-atom RMSD.

	RMSD of best conformer/Å	Rank of best conformer	RMSD of first within 0.5Å	Rank of first conformer with RMSD within 0.5Å
**XXII**	0.08	1	0.08	1
**XXIII** (a)	0.28	51	0.30	4
**XXIII** (b)	0.28	7	0.42	2
**XXIII** (c) molecule 1	0.50	52	-	-
**XXIII** (c) molecule 2	0.38	7	0.49	2
**XXIII** (d)	0.26	7	0.40	2
**XXIII** (e) molecule 1	0.30	52	0.47	2
**XXIII** (e) molecule 2	0.23	31	0.43	1
**XXV** molecule 1	0.05	1	0.05	1
**XXV** molecule 2	0.11	1	0.11	1
**XXVI**	0.55	27	-	-

Crystal Structure Prediction

Table 12 summarises the best predictions from the method for the full lists of predicted structures prior to filtering, as determined by crystal structure packing similarity. For polymorph (b) of structure **XXIII**, filters removed an experimentally observed structure (it was 0.02Å over our clash threshold of 0.5Å). Later analysis of **XXII** showed that we had not run sufficient structures in the CSD, and structures with a matched cluster size of 20 are found with a larger number of structural analogues (see below).

Table 12. Largest predicted crystal structure packing similarity matched clusters with RMSD < 1.0Å for each experimentally observed structure.

	Cluster size	RMSD/Å	Rank
**XXII**	14	0.70	63
**XXIII** (a)	11	0.47	832
**XXIII** (b)	20	0.44	23
**XXIII** (c)	5	0.81	1273
**XXIII** (d)	7	0.91	5744
**XXIII** (e)	6	0.99	998

**XXV**	11	0.45	433
**XXVI**	7	0.55	4514

Retrieval performance with the experimental and generated conformations

Program runs were repeated using both the experimentally observed conformation and generated conformations as the input, to determine whether using structural analogues could have found the observed crystal structure in either case if up to 100,000 structural analogues were used. The results are summarised in Table 13.

Table 13. Number of crystal structures generated in order to find one with a crystal packing similarity match (RMSD < 0.5Å for a matched cluster of 15 molecules) to the observed crystal structure. The results here are not filtered using expected structural criteria.

Structure	Observed conformer	Generated conformers
**XXII**	480	9199
**XXIII** (a)	1500	-
**XXIII** (b)	126	683
**XXIII** (c)	-	-
**XXIII** (d)	1342	-
**XXIII** (e)	-	-
**XXV**	-	-
**XXVI**	234	-

This showed that when starting with experimentally observed conformation, structures with more than one molecule in the asymmetric unit (**XXIII**(c), **XXIII**(e) and **XXV**) were not found in the first 100,000 generated structures. However, molecule **XXVI** was found in only 234 generated structures, suggesting that the main reason for not predicting its structure with our method was the failure to generate a conformation sufficiently close to the experimentally-observed one, as shown in Table 11.

The large difference in the results for observed and generated conformers of **XXII** is an artefact of the local minimisation protocol, which is very sensitive to the precise starting point and the variables in the model.

Conclusions

The results show that there are a number of challenges to in using structural analogues for crystal structure generation, in particular modelling multi-component systems (e.g. molecule **XXV**) and selecting which conformations(s) to use with a given structural analogue. These will be the focus of future development efforts, along with improvements to the scoring function used for ranking the results.

References

Ballester, P.J. & Richards, W.G. (2007). Ultrafast shape recognition to search compound databases for similar molecular shapes. *J. Comput. Chem.,* **28**, 1711-1723.

Bell, B. M. (2015). *CppAD. A C++ Algorithmic Differentiation Package,* http://www.coin-or.org/CppAD/

Bruno, I. J., Cole, J. C., Kessler, M., Luo, J., Motherwell, W. D. S., Purkis, L. H., Smith, B. R., Taylor, R., Cooper, R. I., Harris, S. E. & Orpen, A. G. (2004). Retrieval of Crystallographically-Derived Molecular Geometry Information. *J. Chem. Inf. Comput. Sci.* **44,** 2133–2144.

CCDC (2014). *Conformer Generator 0.9.3.* Cambridge Crystallographic Data Centre, Cambridge, CB2 1EZ, UK.

Chisholm, J. A. & Motherwell, W. D. S. (2005). COMPACK: a Program for Identifying Crystal Structure Similarity Using Distances., *J. Appl. Cryst.*, **38**, 228-231.

Daylight (2008). *SMARTS. A Language for Describing Molecular Patterns.* Daylight Chemical Information Systems, Laguna Niguel, CA 92677, USA.

Filippini, G. & Gavezzotti, A. (1993). Empirical intermolecular potentials for organic crystals: the `6-exp' approximation revisited. *Acta Cryst.* B**49**, 868-880.

Galek, P. T. A., Allen, F. H. Fábián, L. & Feeder, N. (2009). Knowledge-Based H-Bond Prediction to aid experimental polymorph screening. *CrystEngComm*, **11**, 2634-2639.

Gavezzotti, A. & Filippini, G. (1994). Geometry of the Intermolecular X-H...Y (X, Y = N, O) Hydrogen Bond and the Calibration of Empirical Hydrogen-Bond Potentials. *J. Phys. Chem*., **98**, 4831–4837.

Gavezzotti, A. (2011). Efficient computer modelling of organic materials. The atom-atom, Coulomb-London-Pauli (AA-CLP) model for intermolecular electrostatic-polarisation, dispersion and repulsion energies. *New J. Chem.,* **35**, 1360-1368.

de Gelder, R., Wehrens, R. & Hageman, J. A. (2001) A generalized expression for the similarity of spectra: application to powder diffraction pattern classification. *J. Comput. Chem.*, **22**, 273-289

Liu, D. C. & Nocedal, J. (1989) On the Limited Memory BFGS Method for Large Scale Optimization. *Math. Program.* **45**, 503–528.

Macrae, C. F., Edgington, P. R., McCabe, P., Pidcock, E., Shields, G. P., Taylor, R., Towler, M. & van de Streek, J. (2006). Mercury: visualization and analysis of crystal structures. *J. Appl. Cryst.*, **39**, 453-457.

McCabe, P., Korb, O. & Cole, J. (2014). Kernel Density Estimation Applied to Bond Length, Bond Angle, and Torsion Angle Distributions. *J. Chem. Inf. Model.* **54**, 1284–1288.

McCabe, P., Korb, O. & Cole, J. (2015). Knowledge-Based Optimization of Molecular Geometries using the CSD. Manuscript in preparation.

Sadowski, J. & Gasteiger, J. (1993). *Chemical Reviews* **93**, 2567-2581.

Sadowski, J., Gasteiger, J. & Klebe, G. (1994). *J. Chem. Inf. Comput. Sci.* **34**, 1000-1008.

Taylor, R., Cole, J. C., Cosgrove, D., Gardiner, E., Gillet, V. & Korb, O. (2012). Development and validation of an improved algorithm for overlaying flexible molecules. *J. Comput. Aid. Mol. Des.*, **26**, 451-472.

Taylor, R.; Cole, J.; Korb, O. & McCabe, P. (2014). Knowledge-Based Libraries for Predicting the Geometric Preferences of Druglike Molecules. *J. Chem. Inf. Model.* **54**, 2500-2514

Wavefunction (2014). *Spartan '14 Parallel Suite.* Wavefunction, Inc. Irvine, CA 92612, USA.
