# Supplementary material for: Report on the sixth blind test of organic crystal structure prediction methods
Source: Acta Crystallogr B Struct Sci Cryst Eng Mater. 2016 Aug 1;72(Pt 4):439–59. doi: 10.1107/S2052520616007447 (PMC4971545; doi:10.1107/S2052520616007447)
Supplement: Supplementary file 3 [file b-72-00439-sup3.zip › Group_03_Day_Post_Analysis.pdf]

Day group submission to the 6th blind test of crystal structure prediction

Graeme M. Day, Peter J. Bygrave, Joshua Campbell, David H. Case, Thomas Gee, David P. McMahon, Jonas Nyman, Angeles Pulido, Christopher R. Taylor, Jack Yang

December 2, 2015

School of Chemistry, University of Southampton, g.m.day@soton.ac.uk

1 General description of methods

We first provide a general description of the approach used for crystal structure prediction, which involved the following stages: conformational analysis; crystal structure generation; lattice energy minimisation and clustering to remove duplicate crystal structures at one or more stages for each target.

Prediction was attempted for all five blind test targets. Methodological details that are specific to each target are given in the section describing calculations on that target.

1.1 Summary of submissions

Predictions are submitted for the five targets (XXII – XXVI). Two sets of 100 crystal structures are submitted for each target. All are ranked using energies calculated from an anisotropic intermolecular force field, combined with a density functional theory intramolecular energy model.

- XXII: Rigid molecule search followed by flexible molecule lattice energy minimisation. One list ranked by calculated lattice energy (XXII_List1_latt_Day.cif) and a second list ranked by Helmholtz free energy (XXII_List2_free_Day.cif), calculated from harmonic lattice dynamics.
- XXIII: Both lists are based on calculated lattice energies, but differ in the treatment of flexibility throughout the calculations. One method used a flexible search and flexible lattice energy minimisation (XXIII_List1_flexsearch_Day.cif). The second employed a rigid molecule search, followed by flexible molecule lattice energy minimisation (XXIII_List2_rigidsearch_Day.cif).
- XXIV: Rigid molecule search followed by flexible molecule lattice energy minimisation. Both lists are based on calculated lattice energies, differing in the treatment of polarisation (XXIV_List1_eps3_Day.cif and XXIV_List2_eps7_Day.cif).
- XXV: Rigid molecule search followed by flexible molecule lattice energy minimisation. One list ranked on calculated lattice energy (XXV_List1_latt_Day.cif), a second list ranked by Helmholtz free energy (XXV_List2_free_Day.cif), calculated from harmonic lattice dynamics.
- XXVI: Rigid molecule search, flexible molecule lattice energy minimisation. Both lists are based on calculated lattice energies, differing in the treatment of polarisation (XXIV_List1_eps3_Day.cif and XXIV_List2_eps7_Day.cif).

The data field in the cif files gives the rank, relative energy (kJ/mol), target number, space group number and Sobol seed number for each structure.

1.2 Molecular geometries and conformer searches

For each molecule investigated, and unless stated otherwise, molecular conformers were generated using a low-mode conformational search (LMCS) method [1, 2], as implemented in MacroModel [3], to ensure conformer sets were as complete and unbiased as possible. In the LMCS algorithm, a starting molecular geometry is initially optimized, subsequently perturbed along a random combination of its calculated normal modes and re-optimised. Newly generated molecular conformers are clustered on the fly. Due to the large number of energy evaluations and minimisations required for an exhaustive search, the initial conformer search was performed using a force field based methodology. Our chosen force field, OPLS2005, is a version developed by Schrödinger [4] of the OPLSAA all-atom potential [5] with updated torsion parameters [6]. The OPLS2005 force has recently been used by members of our group in a theoretical study of the conformational distortion in crystal structures of a series of flexible organic molecules [7], providing us with valuable information and guidance about: (i) the relative accuracy between OPLS2005 and DFT methodologies in the study of molecular conformations and (ii) the limits on molecular strain that can be induced due to crystallization. Conformation searches were terminated after 50000 molecular conformations were generated using minimum and maximum move distances of 3 and 6 Å. Each conformational search was started from a 3D molecular geometry drawn in the MacroModel visualizer, Maestro. Energy minimisation was performed using the PRCG algorithm (Polak-Ribiere conjugate gradient) [8] and were considered converged when gradients were below 0.05 kJ/mol/Å. Duplicate molecular geometries were removed if all-atom RMS deviation of atomic positions, as implemented in MacroModel, was lower than 0.02 Å.

In our experience, significant inaccuracies in the description of the possible conformers can result from the use of a force field based methodology. Therefore, a more accurate description was obtained by DFT-D re-optimisation of all unique conformers within a 50 kJ/mol window of the OPLS global minimum. DFT-D geometry optimisation was performed with the Gaussian 09 code [9] using the B3LYP [10, 11] functional and 6-311G(d,p) basis set. Dispersion energy corrections were included using Grimme's D3 scheme with Becke and Johnson (BJ) damping [12, 13]. Ultrafine integration grid and tight SCF convergence criteria and were selected for wavefunction evaluation and as convergence criteria, whereas Gaussian 09 default thresholds were selected for assessing energy minimisation convergence.

For each given target molecule, the relative energy of a given conformer, E_{gas}^{rel}, was defined (see Eq. 1) as the difference between its total gas phase DFT-D energy, E_{gas}^{total}, and the lowest total gas phase DFT-D energy of that molecule conformers, E_{gas}^{lowest}.

$$E_{gas}^{rel} = E_{gas}^{total} - E_{gas}^{lowest}. \tag{1}$$

To select the final set of molecular conformers relevant to crystal packing, the DFT-D optimised conformers were clustered to remove duplicates, using in-house software, with thresholds on all-atom RMS deviations of atomic positions of 0.4, 0.2, 0.5 and 0.5 Å for molecules XXII, XXIII, XXIV and XXVI, respectively.

The molecules in target XXV have very limited conformational freedom, so no systematic conformational search was performed. Details about generation and selection of molecular conformations for crystal packing of XXV are given below.

Unless stated otherwise, for each of the blind test molecules a series of rigid body crystal searches were initially performed using a range of molecular conformers. Thompson and Day [7] showed that conformers up to 26 kJ/mol are seen in crystal structures of flexible molecules. Thus, for each molecule an energy cut-off of 30 kJ/mol above the DFT-D global minimum was imposed for the selection of final molecular conformers to be used in the crystal structure searches. Additional information for each molecule can be found below.

1.3 Crystal structure generation

Upon determining the molecular conformations to be sampled, we proceeded with each CSP study by generating structures with our in-house code, Global Lattice Energy Explorer (G.L.E.E.). For each structure, the degrees of freedom to be sampled are determined by an algorithm which maps a quasi-random vector, drawn from a Sobol sequence [14], onto the relevant parameters, as shall

be described in a forthcoming paper [15]. The positions of the molecules in the asymmetric unit are simply random linear translations, their orientations are sampled according to Shoemake's method [16], and internal lattice angles are also simply mapped from the random variate. In order to sample unit cell lengths, we first assign a nominal "volume" to each molecule by projecting each atomic position onto the eigenvectors of the moments-of-inertia matrix, and determining a cuboid from the maxima and minima along each axis. The volume of the sum of the cuboids of each molecule in the asymmetric unit is multiplied by the number of symmetry elements to yield a target volume for the unit cell. The mapping of the random variate onto lengths of the unit cell vectors can be achieved with bounds relating to the orientation of the molecules, and the number of unique fractional coordinates of each axis in each space group. In order to generate structures whose molecules do not overlap, we calculate the convex hull of each molecule, and allow the cell to expand to relieve molecular clashes up to a maximum volume of 2.5 times the initial target volume. Overlaps of the molecular convex hulls were checked using the separating axis theorem, which also provides the direction for expansion of the unit cell. Crystal structures were subsequently lattice energy minimised and the quasi-random structure generation was repeated until a target number of successful lattice energy minimisations was achieved. One list of predictions for XXIII used a modified approach, where low energy intramolecular distortions were included in the variables sampled during structure generation - more details are provided below.

1.4 Lattice energy minimisation

Crystal structures were initially lattice energy minimised in the rigid molecule approximation using Neighcrys 2.0.4 and DMACRYS 2.0.4 [17, 18]. Unless stated otherwise, atom-centred distributed multipoles up to rank 4 (hexadecapoles) were calculated from the B3LYP-D3(BJ)/6-311G(d,p) charge density using the original implementation of the distributed multipole algorithm [19], as implemented in GDMA 2.2 [20, 21]. DFT calculations were performed with Gaussian 09 [9].

Intermolecular repulsion-dispersion interactions were calculated with empirically parameterised model potentials of the exp-6 form. The revised Williams99 [22, 7, 23] force field was used, supplemented with parameters for S [24], Cl [25], water and Cl^- as needed.

Charge-charge, charge-dipole and dipole-dipole interactions were summed using Ewald summation. Unless otherwise stated, a 15 Å cutoff on van der Waals and higher order electrostatics was used.

After clustering, the lowest energy structures were re-optimised with intramolecular flexibility, using the CrystalOptimizer [26] software, which combines the atom-atom potential description of intermolecular interactions with a quantum chemical description of the intramolecular energy. A set of flexible torsions and angles are specified, which are optimised in response to intermolecular packing forces in the crystal.

After CrystalOptimizer, a new rigid body geometry optimization was performed using polarized multipoles [27], derived from a DFT charge density calculated in a polarizable continuum model [28, 29]. A modified force field [23], in which all hydrogen bonding interactions are parameterized specifically to be used with PCM multipoles was used.

1.5 Clustering

To remove duplicate structures, we used both in-house tools and the program COMPACK [30]. The in-house code calculates the RMSD of the distributions of atomic positions for each molecule, around the centroid of each molecule in the asymmetric unit. For computational efficiency, it only includes the atoms which determine the convex hull of each molecule, and further details are described in [15]. This method has not yet been implemented for multiple molecules in the asymmetric unit. Therefore, for certain systems, the COMPACK algorithm, which calculates the similarity of crystal structures as a root mean square distance between atomic coordinates for a cluster of overlayed molecules was used, and use of this algorithm is noted in the text. For COMPACK clustering, molecular clusters of 25 molecules with a 20% distance tolerance were used. Hydrogen atoms were not used in the calculations based on COMPACK, unless otherwise stated.

1.6 Free energy calculations

For targets XXII and XXV Helmholtz lattice vibrational free energies were calculated from phonon frequencies calculated in the rigid molecule, harmonic approximation. The theory of rigid-body lattice dynamics has been described elsewhere [31, 32, 33, 34]. No intramolecular vibrations or phonon-phonon couplings are considered. Our methods for free energy calculations have been described previously [35, 36].

Phonons at $\mathbf{k} = \mathbf{0}$ can be calculated with DMACRYS. Other **k**-points are sampled by making supercells of the crystal unit cell. The supercell method is most efficient for unit cells with isotropic dimensions, so structures with very acute or obtuse lattice angles were first converted to reduced unit cells [37, 38, 39] with PLATON [40].

Supercells were then constructed from the unit cell by making expansions in **a**, **b** and **c** only, producing linear supercells. The length of the linear supercells is determined by a target **k**-point distance. We expand the unit cell until the distance between adjacent **k**-points is less than 0.1 Å^{-1}.

If the supercell expansion coefficient $s < 6$, the lattice dynamics calculation is performed on the supercell as is. For $s \geqslant 6$ the supercell (1x1xs) is split into n smaller supercells (1x1xk, 1x1xℓ, 1x1xm...) such that $k, \ell, m...$ are all mutually co-prime and $k + \ell + m - n \geqslant s$. This ensures that at least s **unique k**-points are sampled. The phonons for $\mathbf{k} = \mathbf{0}$ will be calculated in each supercell, and we only include these phonons from one of the split supercells. The long linear supercells are split into 2, 3 or 4 co-prime supercells according to the scheme in Table 1.

2	→	2
3	→	3
4	→	4
5	→	5
6	→	3, 4
7	→	3, 5
8	→	4, 5
9	→	3, 4, 5
10	→	3, 4, 5
11	→	3, 4, 7
12	→	3, 4, 7
13	→	3, 5, 7
14	→	4, 5, 7
15	→	5, 6, 7
16	→	3, 4, 5, 7
17	→	5, 7, 8
18	→	5, 7, 8
19	→	3, 5, 7, 8
20	→	3, 5, 7, 8

Table 1: Linear supercells were split into 2, 3 or 4 smaller supercells with mutually co-prime expansion coefficients in this way.

To improve the convergence of the calculated free energy with respect to the number of sampled **k**-points, we used the Debye approximation of linear dispersion out to the nearest explicitly sampled **k**-points from the Brillouin zone centre, using an average phonon frequency on a spheroid around the Γ-point. The Debye frequency is calculated from the elastic stiffness tensor of each crystal structure, also calculated with DMACRYS, as described elsewhere [41, 42].

The Helmholtz energy is the sum of the static lattice energy and the lattice vibrational contribution to the free energy F_{vib}. For six degrees of freedom, N_k sampled **k**-points and N_m molecules per unit cell, the vibrational energy per unit cell is

$$\begin{aligned} F_{vib}(T) = {} & \frac{1}{2}\Big(\frac{6N_mN_k - 3}{6N_mN_k}\Big)\sum_{i,\mathbf{k}} \hbar\omega_{i,\mathbf{k}} \\ & + k_BT\Big(\frac{6N_mN_k - 3}{6N_mN_k}\Big)\sum_{i,\mathbf{k}} \ln\left(1 - \exp\left(\frac{-\hbar\omega_{i,\mathbf{k}}}{k_BT}\right)\right) \\ & + \frac{9\hbar\omega_D}{8N_k} \\ & + \frac{3k_BT}{N_k}\ln\left(1 - \exp\left(\frac{-\hbar\omega_D}{k_BT}\right)\right) \\ & - \frac{k_BT}{N_k}\mathrm{D}\Big(\frac{\hbar\omega_D}{k_BT}\Big) \end{aligned} \tag{2}$$

where $\mathrm{D}(x)$ is the Debye function

$$\mathrm{D}(x) = \frac{3}{x^3}\int_0^x \frac{t^3}{\exp(t) - 1}dt, \tag{3}$$

which is integrated numerically.

2 Molecule XXII

2.1 Conformational analysis

For target molecule XXII, we find two symmetry-related, buckled molecular conformers during the OPLS2005 conformational search, within an energy window of 50 kJ/mol above the OPLS2005 global minimum. A planar molecular conformation was also identified as a first order saddle point between the two minima. After DFT-D (B3LYP-D3(BJ)/6-311G(d,p)) geometry optimisation, the saddle point is found to have a relative energy, E_{gas}^{rel}, of only 5.7 kJ/mol (see Table 2 for conformer energies of all target systems). To sample the low energy pathway from buckled to planar conformation, crystal structures were generated with both the minimum and saddle point geometries. The molecular geometry is given sufficient flexibility to allow buckling from the planar conformation during lattice energy minimisation. The respective sets of crystal structures are denoted XXII-C1 and XXII-C2.

XXII conformational search accounts for 1.2 CPU-hours. Conformational search CPU breaks down by methodology is as follows: (i) initial OPLS2005 conformational search was performed on the i7-3770 personal computer accounting for 0.2 CPU-hours, and (ii) DFT-D geometry optimisations were performed on Iridis4 cluster accounting for 1.0 CPU-hours. See Sec. 7 below for detailed hardware description.

XXII		XXIII		XXIV		XXVI	
Conformer	E_{gas}^{rel}	Conformer	E_{gas}^{rel}	Conformer	E_{gas}^{rel}	Conformer	E_{gas}^{rel}
C1	0.0	C1	0.0	1	0.0	C1	0.0
C2(TS)	5.7	C2	2.7	2	9.0	C2	4.0
		C3	5.3	3	9.4	C3	4.6
		C4	6.8	4	28.5	C4	5.3
		C5	7.1			C5	6.7
		C6	7.4			C6	7.1
		C7	7.4			C7	9.9
		C8	7.6			C8	11.1
		C9	7.6			C9	11.2
		C10	7.7			C10	11.2
		C11	14.3			C11	12.4
		C12	14.8			C12	12.7
		C13	14.9			C13	13.1
		C14	14.9			C14	13.7
		C15	14.9			C15	14.2
		C16	15.0			C16	14.2
		C17	15.0			C17	15.6
		C18	17.2			C18	16.3
		C19	18.9			C19	16.5
		C20	18.9			C20	17.4
		C21	19.2			C21	17.5
		C22	20.9			C22	18.3
		C23	21.0			C23	20.9
		C24	21.7			C24	21.1
		C25	21.8			C25	21.3
		C26	21.9			C26	21.8
		C27	21.9			C27	22.3
		C28	22.1			C28	22.4
		C29	22.2			C29	22.9
		C30	22.2			C30	24.2
		C31	23.9			C31	24.9
		C32	24.3			C32	25.2
		C33	25.1			C33	26.2
		C34	25.8			C34	26.4
		C35	27.5			C35	28.0
		C36	27.5			C36	28.0
		C37	28.0			C37	28.0
		C38	28.7			C38	28.1
		C39	28.9			C39	29.3
		C40	29.0			C40	29.5
		C41	29.2				
		C42	29.2				
		C43	29.3				
		C44	29.3				
		C45	29.3				
		C46	29.4				
		C47	29.5				

Table 2: Relative DFT-D energies, E_{rel} (in kJ/mol), of the selected unique conformers for molecules XXII, XXIII, XXIV and XXVI. Total B3LYP-D3(BJ)/6-311G(d,p) electronic energies of the lowest energy gas phase conformers, E_{gas}^{lowest}, for molecules XXII, XXIII, XXIV and XXVI are −1718.5574, −1936.5203, −814.7257 and −2489.8896 E_h, respectively. Conformers followed by (TS) are saddle point geometries.

2.2 Details of structure generation

For molecule XXII rigid body searches containing molecular geometries of conformers C1 and C2 were performed. Conformer C1 is chiral so 94 space groups were sampled overall (including both space groups from enantiomorphic space groups). The 25 most common space groups had 5000 valid structures generated in each, with 2000 structures in the remaining 69. All of the additional space groups that gave crystal structures within 25 kJ/mol of the global minimum during this initial sampling were restarted to take them up to 5000 total structures (46 space groups in total required this additional sampling). No change in the lowest energy structures was observed during this extended sampling and only 5 crystal structures from the additional space groups made it within 15 kJ/mol of the global minimum. The planar C2 geometry is non-chiral, so 87 space groups were sampled (including only one from each enantiomorphic pair) using the same process as for C1. After an initial 5000 structures the lattice energy of the global minimum was −120.9 kJ/mol. 34 uncommon space groups were sampled further, using the same criteria as above. 4 structures from those additional calculations were within 15 kJ/mol of the minimum. Structure generation and initial minimisations took 26497 CPU-hours.

The original Williams99 potential was used for C and N. Parameters for sulfur [24] were added using combining rules for S···N and S···C interactions.

2.3 Details of energy minimisation

After rigid body lattice energy minimisation, flexible molecule lattice energy minimisation was performed, using CrystalOptimizer, on a subset of crystal structures within an energy window of 15 kJ/mol above the minimum energy structure for each conformer. During flexible molecule lattice energy minimisation, all bond lengths and angles (except the angles in the cyano groups) were allowed to relax to their DFT equilibrium values; whereas the torsions shown in Fig. 1 were allowed to flex in response to intermolecular packing forces. The cyano groups were constrained to an angle of 179.99°.

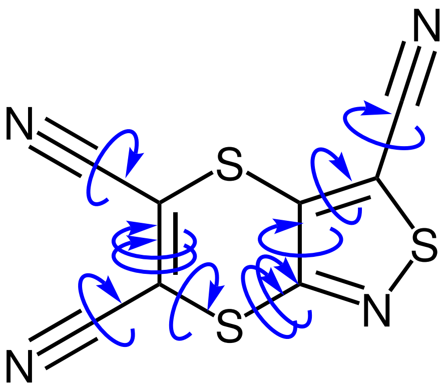

Figure 1: XXII CrystalOptimizer flexible torsions. Two arrows around the same bond indicate two separate degrees of freedom. (Rotation about the linear cyano groups leaves the geometry unchanged, but these torsions were included during the calculations in error.)

After CrystalOptimizer flexible molecule lattice energy minimisation, duplicate crystal structures were removed and a total of 753 unique XXII polymorphs identified.

A total of 853.7 CPU-hours were used during CrystalOptimizer energy minimisation calculations of XXII crystal structures; and an additional 7.8 CPU-hours were used for clustering.

2.4 Free energy calculations

The Helmholtz free energy was calculated at 300 K for all predicted structures of molecule XXII.

Atom-centred distributed multipoles [43, 44, 45] were calculated from the B3LYP-D3(BJ)/6-311G(d,p) charge density, calculated in a polarisable continuum model [28, 29] with a relative permittivity of 3.0, using Gaussian 09 [9] and GDMA [21].

Crystal structures with highly anisotropic dimensions were converted to reduced cells with PLATON's ADDSYMM routine [40, 37, 38, 39].

The **k**-point sampling method described above was used with a target **k**-point distance of 0.1 Å^{-1} resulting in an average of 21 unique **k**-points being sampled. The minimum number of **k**-points sampled in any one structure was 12, maximum 33.

Many structures were initially found to be located at saddle points on the potential energy surface, yielding imaginary phonon frequencies. Such structures had their symmetry reduced to $P1$. If the $P1$ structure was also unstable, the unit cell was doubled and calculations were performed on the 1x1x2, 1x2x1, and 2x1x1 supercells. The space group in which the structure was first generated can therefore differ from the space group of the final optimised structure. The identification and exclusion of unstable structures based on imaginary phonon frequencies has been advocated earlier [46, 47].

The free energy calculations took typically 45 CPU-minutes per structure. The minimum time for any structure was 17 minutes, maximum 12 h. In total 1724 CPU-hours were spent on free energy calculations for this target molecule.

2.5 Final ranking of structures

We submit two lists of 100 structures, ranked by lattice and free energy respectively. The lattice vibrational energy can sometimes cause important differences in the ranking of polymorphs [48, 36].

As well as the vibrational contribution to free energies, our two sets o predictions also differ in that we leave the broken symmetry structures (see above) in the list that is ranked by free energy, but not in the lattice energy ranked list. As this blind test category is stated to be $Z' = 1$, the resulting high Z' structures that result from symmetry breaking are not expected to match the observed structure, whereas they do constitute true structures on the lattice energy surface.

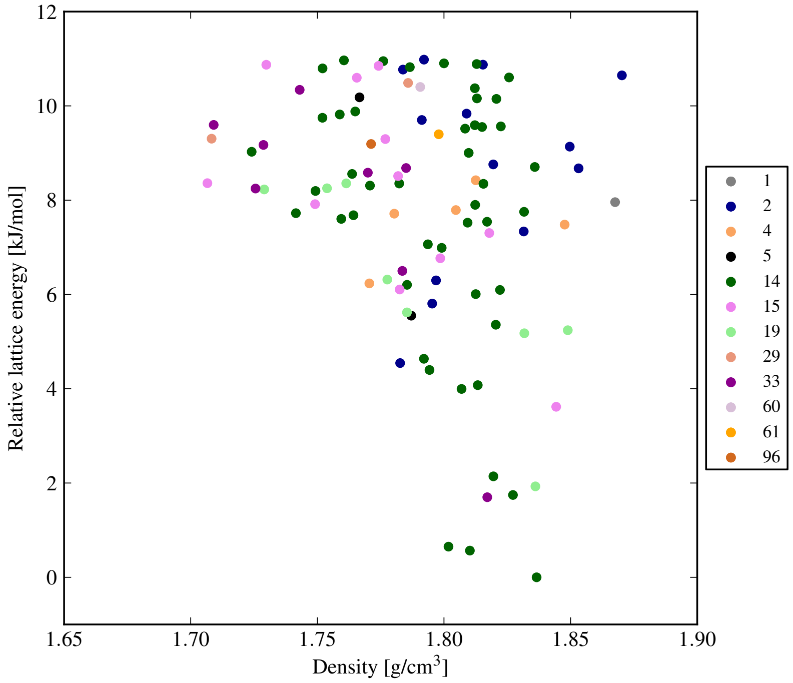

Figure 2: Lattice energy landscape of target XXII. Only the lowest 100 structures are included. Data labels denote space group number.

The Kendall's τ_b rank correlation [49] between the lists ranked by lattice energy and free energy is 0.83 for the structures below 20 kJ/mol lattice energy. The correlation between the two lists is shown in Figure 4.

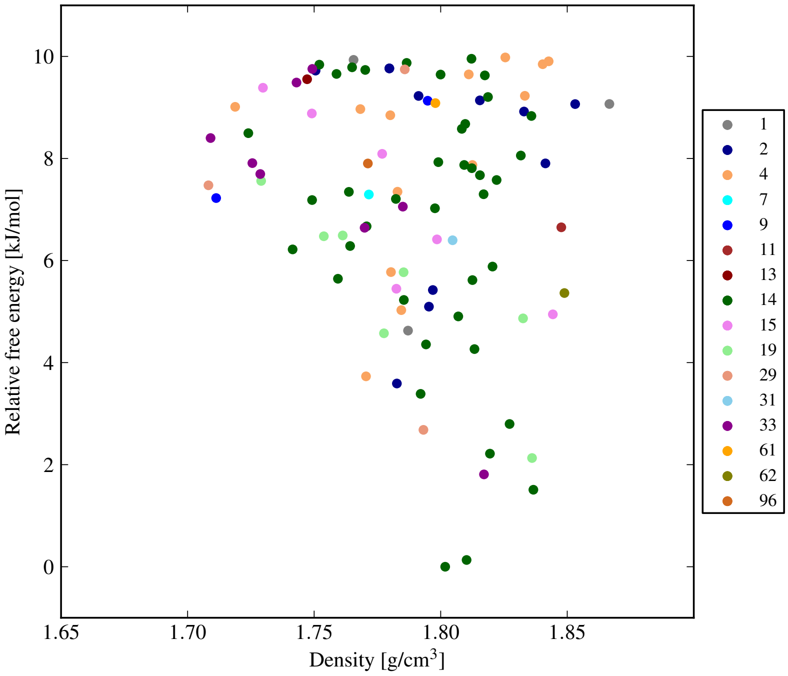

Figure 3: Free energy landscape of target XXII at 300 K. Only the 100 lowest ranked structures included. Labels denote space group number.

The lattice energy global minimum structure is ranked third by free energy.

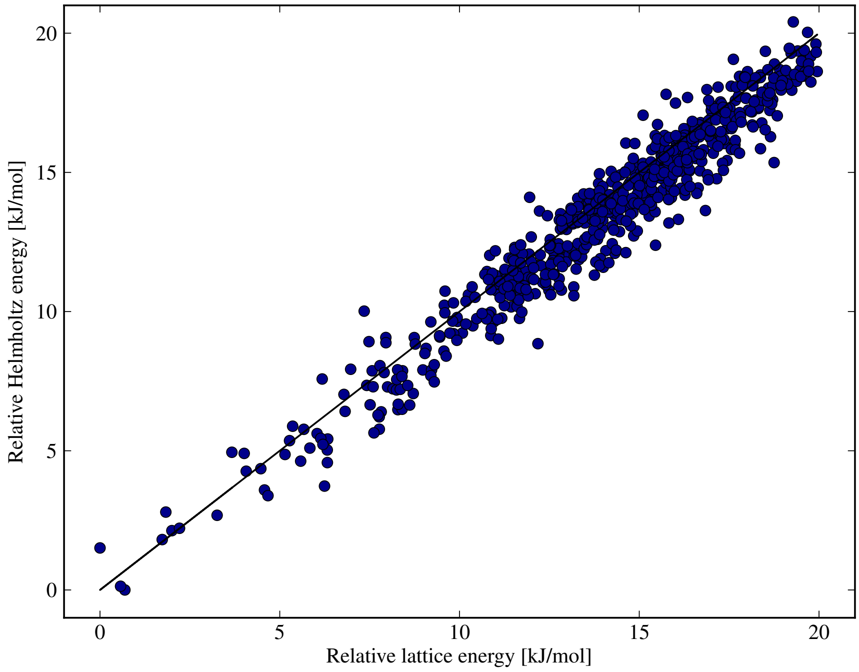

Figure 4: The free energy and the lattice energy are strongly correlated for target XXII, as expected. The rank correlation is 0.85.

3 Molecule XXIII

3.1 Conformational analysis

For target molecule XXIII, 154 molecular conformers were identified during the OPLS2005 conformational search. After DFT-D (B3LYP-D3(BJ)/6-311G(d,p)) geometry optimisation and clustering, 47 molecular conformers were identified with relative energies, E_{gas}^{rel}, smaller than 30 kJ/mol, see Table 2. Trial crystal structures were generated containing molecular geometries of each of the 47 conformers C1, C2, ..., C47.

The XXIII conformational search accounts for 1882.6 CPU-hours. Conformational search CPU breaks down by methodology as follows: (i) 3.4 CPU-hours to perform the initial OPLS2005, and (ii) 1879.2 CPU-hours to perform the DFT-D geometry optimisations.

Two types of crystal structure search were performed. All predictions were restricted to one molecule in the asymmetric unit.

3.2 Details of rigid molecule structure generation

For all 47 molecular conformers we initially performed rigid-molecule crystal structure generation and rigid-molecule lattice energy minimisation in the six most common space groups (space group numbers 14, 19, 2, 4, 61, 15), stopping the search after generating 2000 successfully optimised structures in each space group.

A further 1000 optimised crystal structures were generated in each of the next 19 most common space groups (33, 9, 29, 5, 1, 60, 7, 18, 43, 56, 13, 88, 148, 76, 78, 92, 96, 144, 145) for conformers where the total (inter- + intra-molecular) energy of any one of the structures were within 15 kJ/mol of the global minimum from the initial search of 6 space groups. Intramolecular energies were taken from the B3LYP-D3(BJ)/6-311G(d,p) calculations performed in the molecular conformation search.

For conformer/space group combinations where poor sampling was observed, a further 6000 optimised structures were generated. Poor sampling was identified as there being crystal structures within 15 kJ/mol of the global minimum that had only been located once in the search.

In total 132016 CPU-hours were spent on structure generation and rigid molecule energy minimisation.

After removing duplicate structures, 553 structures were found within 15 kJ/mol and 1626 structures within 20 kJ/mol of the global minimum in total energy.

Structures within the 20 kJ/mol window were re-optimised in CrystalOptimizer to take molecular flexibility into account. The degrees of freedom chosen to be optimised in response to packing forces are shown in figure 5.

CrystalOptimizer calculations took a total of 131179 CPU-hours.

Finally, the structures were optimised with atomic multipoles derived from a PCM calculation with a relative permittivity of 3.0 under a rigid-molecule approximation (at the final molecular geometry after CrystalOptimizer). This final optimisation took 2152 CPU-hours. A final clustering was then used to remove any duplicate structures.

In all DMACRYS calculations a van der Waals and non-Ewald electrostatic cutoff of 25 Å was used.

3.3 Flexible-molecule crystal structure generation

In addition to the standard rigid molecule structure generation, we also performed crystal structure prediction calculations in which the molecular conformation is allowed to distort from the local gas phase minimum during structure generation.

Traditionally this approach uses internal degrees-of-freedom, such as the dihedral angles included in the post-generation minimisation. In this study we use our recently developed method [50] that uses the normal modes of vibration of the gas phase conformers as the degrees-of-freedom. These, by definition, are low energy collective motions that can be ordered by energy and offer chemically sensible molecular motions. Due to time constraints, we limited flexibility of each

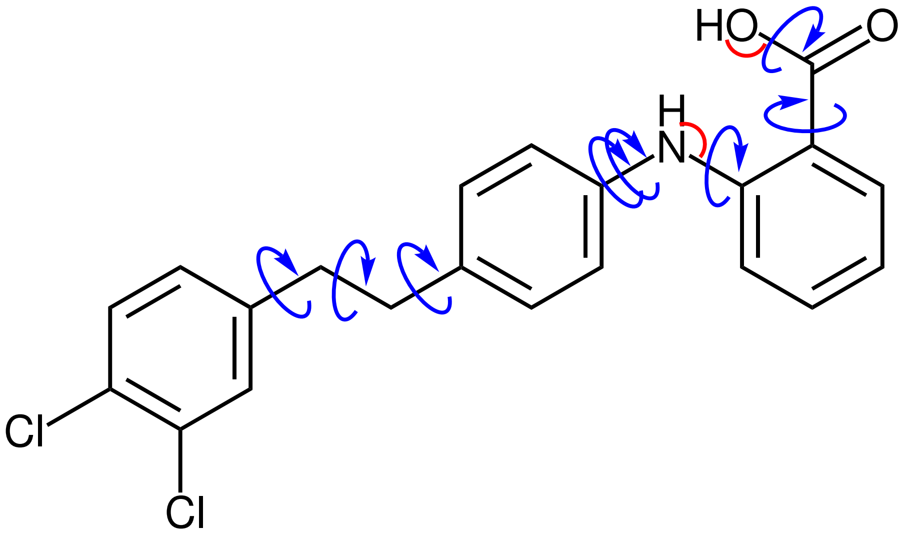

Figure 5: XXIII CrystalOptimizer flexible angles (red) and torsions (blue). Two arrows around the same bond indicate two separate degrees of freedom.

conformer to its two lowest energy modes. These modes were taken as the eigenvectors of the non-mass weighted molecular Hessian at the B3LYP-D3(BJ)/6-311G(d,p) level of theory (calculated in Gaussian09 by setting the mass of each atom to 1.0 during a frequency calculation).

The structure generation technique was extended to include these two degrees of freedom by extending the Sobol vector. These two quasi-random numbers are used to sample the molecular potential energy surface up to 22 kJ/mol from the gas phase geometry, in a scheme that balances even sampling with minimising rejected based on high energy molecular conformations. The boundary energy of 22 kJ/mol is chosen from studies of the molecular strain energy of similar sized molecules found in experimental structures [7]. Finally, we energy minimise the sum of the intra- and inter-molecular lattice energies with respect to the normal-mode displacements. This numerical minimisation technique optimises the displacements along the normal mode coordinates whilst recomputing the lattice energy at each step under a rigid molecule treatment with the newly displaced molecule. To perform lattice energy minimisation, we use a fixed charge model (CHELPG charges) from the DFT calculation at the local gas phase conformation and a Buckingham intermolecular force-field with parameters from the original Williams99 potential [22] supplemented with Cl [25]. In all DMACRYS calculations a van der Waals and non-Ewald electrostatic cutoff of 25 Å was used.

Intramolecular energies are determined from a highly accurate energy model fitted to molecular DFT calculations, all of which transforms this into a tractable procedure. Here, we explicitly calculated 1000 DFT calculations for each conformer at displaced geometries along the two normal modes. The first 150 points with an energy < 22 kJ/mol are used as a test set and the subsequent points with an energy < 35 kJ/mol are used as a training set, giving roughly 500 training points. We then use the Gaussian Processes for Machine Learning module from the scikit-learn package [51] to train a model for this data. For all conformers studied, the maximum absolute errors within the test set are < 0.2 kJ/mol with a mean absolute error of < 0.05 kJ/mol. This now provides us with an energy model at the DFT quality with gradients, that is much quicker to evaluate than the DMACRYS calculation. The fast evaluations are also required for the structure generator to be efficient.

For all conformer and space group combinations that generate a rigid-molecule structure within the 15 kJ/mol energy window, we perform a flexible search and optimisation with normal modes. Space groups 2 and 14 were included for all conformers included in the flexible search. We generate 10000 structures in each space group and optimise each through our normal mode optimiser. In each run between 70 and 85% of all generated structures successfully optimise. After clustering, each unique structure is optimised using the same PCM treatment described above.

To investigate our method further, we also perform the normal mode flexible molecule optimisation and PCM treatment for the crystal structures generated in the rigid search, up to 20 kJ/mol from the global minimum. This allows us to show which extra structures are found using molecular flexibility at the structure generation stage.

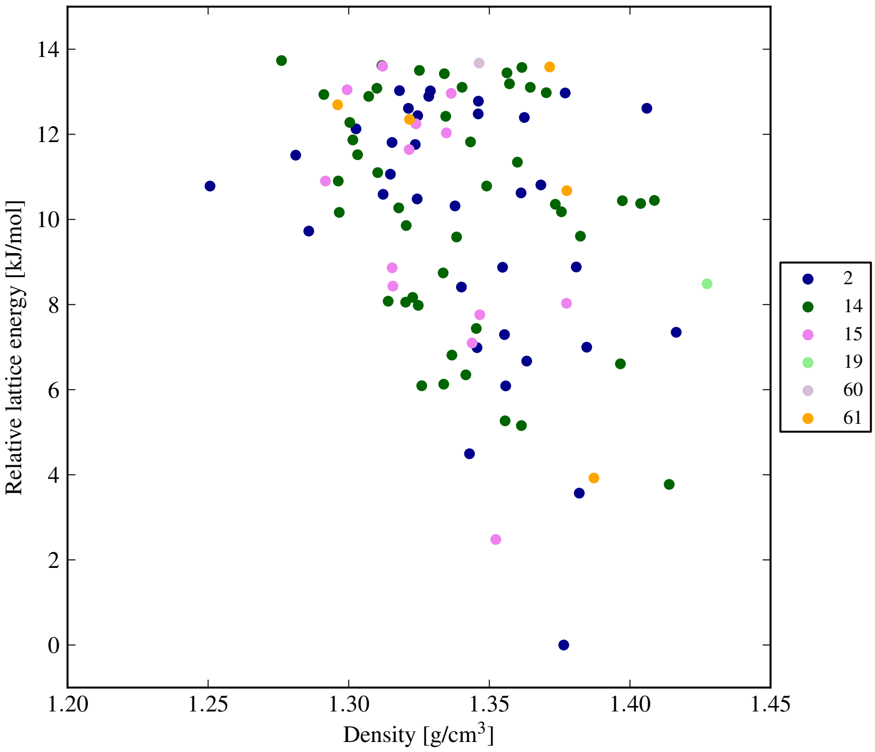

Figure 6: Lattice energy landscape of target XXIII for the lowest 100 structures from the CrystalOptimizer set. The labels are space group numbers.

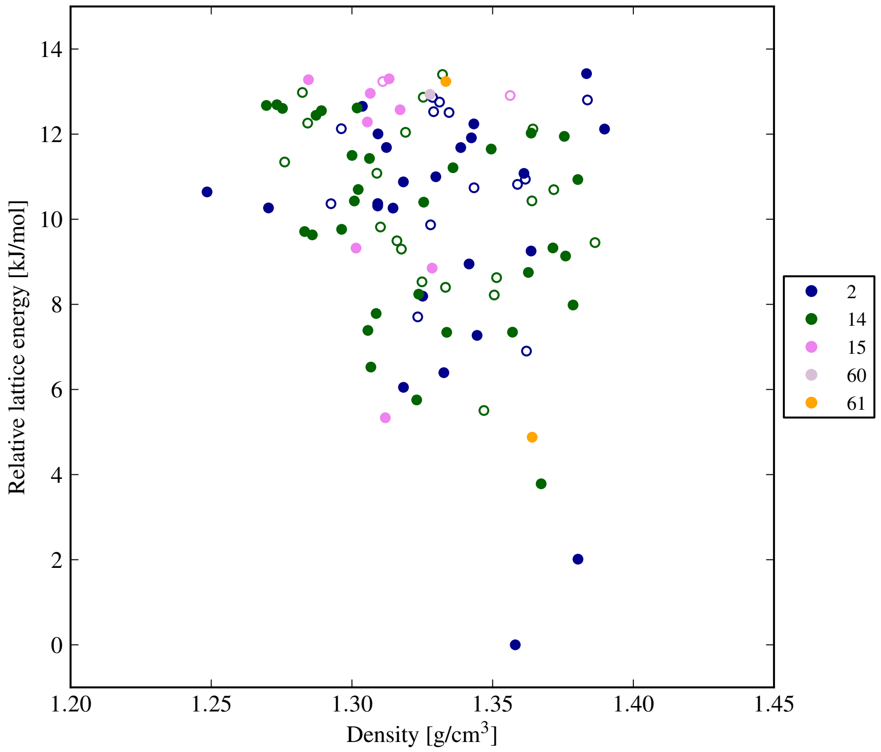

Figure 7: Lattice energy landscape of target XXIII for the lowest 100 structures coming from the normal mode search. Unfilled circles represent those structures found only through flexible structure generation. Filled circles represent structures found after normal mode minimisation of either a rigid or flexibly generated structure. The labels are space group numbers.

4 Molecule XXIV

4.1 Conformational analysis

For target molecule XXIV, 8 molecular conformers were identified during the OPLS2005 conformational search of the cation. After DFT-D (B3LYP-D3(BJ)/6-311G(d,p)) geometry optimisation and clustering, 4 molecular conformers were identified with relative energies, E^{rel}_{gas}, smaller than 30 kJ/mol, see Table 2.

The XXIV conformational search accounts for 6.3 CPU-hours.

4.2 Details of structure generation

Rigid structure generation was carried out using the three lowest-energy conformers (C1, C2, and C3) of the cationic component of target XXIV, using a single fixed water conformation in each case.

For each of the three lowest-energy gas phase conformers of the cationic component of target XXIV, 10000 crystal structures were generated in each of the six most common space groups (14, 19, 2, 4, 61, 15). 2000 structures were generated for each conformer of the cation in each of the next 19 most common space groups.

4.3 Details of energy minimisation

The intermolecular model potential used in these calculations combined our reparametrised version of Williams99 (for use with polarized multipoles) with specific models fitted for interactions involving chloride and water. The chloride potential was parametrised against the crystal structures of a set of chloride salts of organic molecules [52] and the energies of these structures calculated using Gavezzotti's PIXEL method [53]. The water potential was parametrised against the structures of a large set of hydrate crystal structures of organic molecules [54]. All cross terms were calculated using standard combining rules.

The crystal structures resulting from rigid-molecule structure generation were clustered using the COMPACK algorithm, with the carboxylic acid and water hydrogen atoms included in the geometrical comparison used for clustering.

All crystal structures within 25 kJ/mol from the global minimum in total (inter- + intramolecular) energy (1964 crystal structures) were re-minimised using CrystalOptimizer. Degrees of freedom indicated in Fig. 8 were allowed to flex in response to intermolecular packing forces.

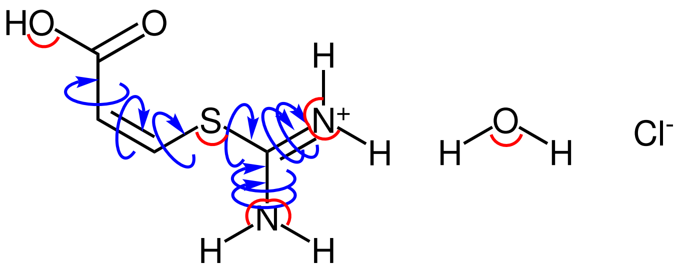

Figure 8: XXIV CrystalOptimizer flexible angles (red) and torsions (blue).

4.4 Final ranking of structures

Finally, all crystal structures from the CrystalOptimizer calculations were re-minimised with their molecular geometries fixed rigid at the CrystalOptimizer geometry, now with multipoles on the cation and water calculated from a PCM calculation.

One set of predictions is based on using a relative permittivity of 3.0 in PCM calculation for these final calculations. This value is typical of organic molecular crystals.

Our experience is that, for crystal structures with net charged groups (salts or zwitterions), the final ranking can be improved by increasing the dielectric constant in the PCM calculations, to

further polarise the multipoles used in the lattice energy minimisation. A second set of predictions was obtained by performing the final calculations using atomic multipoles from PCM calculations with a dielectric constant of 7.0.

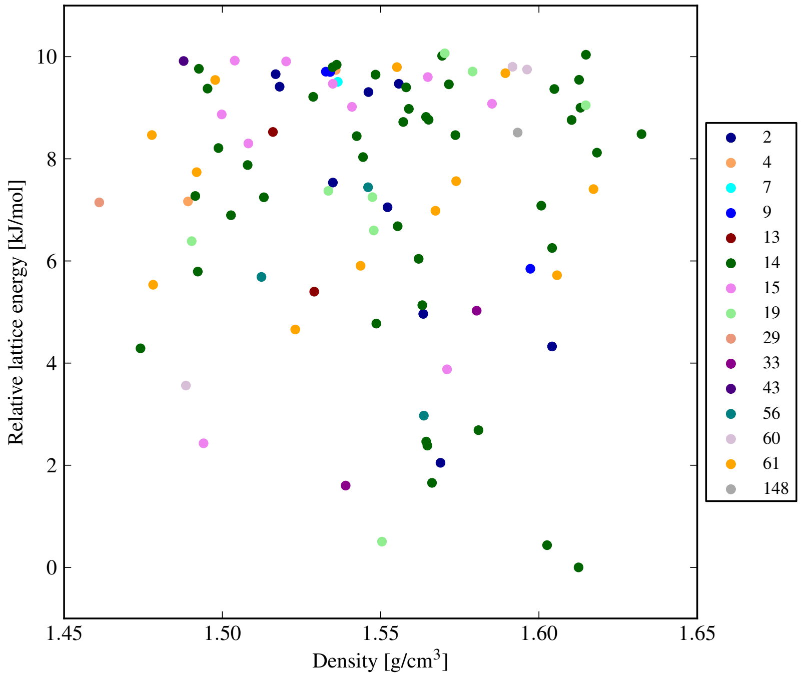

Figure 9: Lattice energy landscape of target XXIV for the lowest 100 structures after calculations with multipoles from PCM with $\epsilon = 3.0$. The labels give space group numbers.

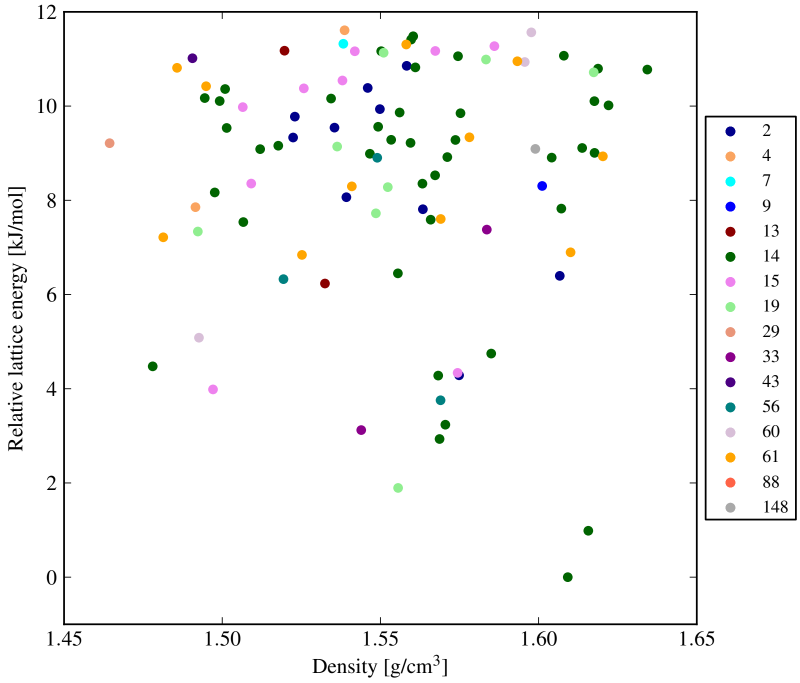

Figure 10: Lattice energy landscape of target XXIV for the lowest 100 structures after calculations with multipoles from PCM with $\epsilon = 7.0$. The labels give space group numbers.

5 Molecule XXV

5.1 Conformational analysis

Three conformers of the 3,5-dinitrobenzoic acid (Figure 11) with differing orientations of the carboxyl OH group and a single conformer of 2,8-dimethyl6H,12H-5,11-methanodibenzo[b,f][1,5]diazocine were optimised with Gaussian 09 according to the general procedure. Conformer A of the acid was found to be the global energy minimum, B a transition state and C a local minimum with relative energies of B and C of 35.12 kJ/mol and 34.03 kJ/mol respectively to conformer A. A total of 22 CPU-hours were used during geometry optimisation.

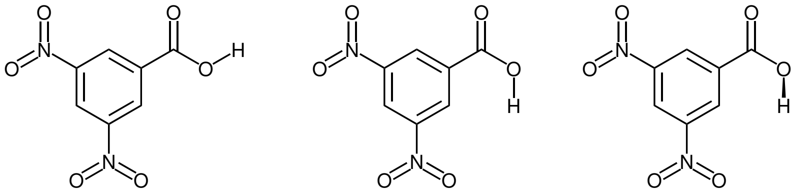

Figure 11: 3,5-dinitrobenzoic acid conformations A, B and C.

5.2 Details of structure generation

Rigid molecule crystal structure generation was carried out using the three specified conformers of the acid component of target XXV in combination with the single conformer of the base component. All calculations were performed in a 1:1 stoichiometry, as given in the specification of this target system.

For the lowest energy gas-phase conformer of the acid component (labelled A), an initial 10000 structures were generated for the co-crystal in the 25 most common space groups. A further 10000 structures were subsequently generated for space groups with structures within 15 kJ/mol of the global lattice energy minimum (2, 7, 9, 14, 15, 19, 29, 33, 43, 60, 61).

For each of the higher-energy gas-phase conformers of the acid (B and C), 10000 structures were generated for the co-crystal in the 25 most common space groups. The chirality of conformer C, combined with the chirality of the base, necessitated generating structures in the subset of those 25 space groups which are enantiomorphic (19, 4, 5, 1, 18, 76, 96, 92, 78, 144, 145) using both possible enantiomers of C, giving 10000 structures for each pairing of enantiomer and enantiomorphic space group.

Structure generation took a total of 113825 CPU-hours split between cocrystals formed from the base and conformer A of the acid (39195 CPU-hours), conformer B (28987 CPU-hours), conformer C (32639 CPU-hours) and the additional sampling of the enantiomorphic spacegroups with the other enantiomer of conformer C (13004 CPU-hours).

Clustering of the structures within 30 kJ/mol of the lattice energy minimum in each of the cocrystal searches was performed using the COMPACK algorithm, with the carboxylic acid hydrogen atom included in the geometrical comparison used during clustering. Clustering took a total of 529 CPU-hours, split between conformer A (70 CPU-hours), conformer B (177 CPU-hours), conformer C (228 CPU-hours) and the other enantiomer of conformer C (54 CPU-hours).

5.3 Details of energy minimisation

Structures within the lowest 30 kJ/mol above the lattice energy minimum structure with each acid conformer were clustered with COMPACK [30]. For structures with conformer A of the 3,5-dinitrobenzoic acid, the lowest 20 kJ/mol were run through CrystalOptimizer with the degrees of freedom as shown in Figure 12. For structures containing conformers B and C of the 3,5-dinitrobenzoic acid only the lowest 10 kJ/mol of structures were run through CrystalOptimizer.

A total of 5981 CPU-hours were used during CrystalOptimizer optimisations of the cocrystals containing the 3 conformers and the additional enantimorphic calculations. Calculations on the cocrystal containing conformer A of the acid accounted for 4259 CPU-hours, B 752 CPU-hours, C

817 hours and an additional 152 CPU-hours for the enantiomorphic space groups arising from the charility of C.

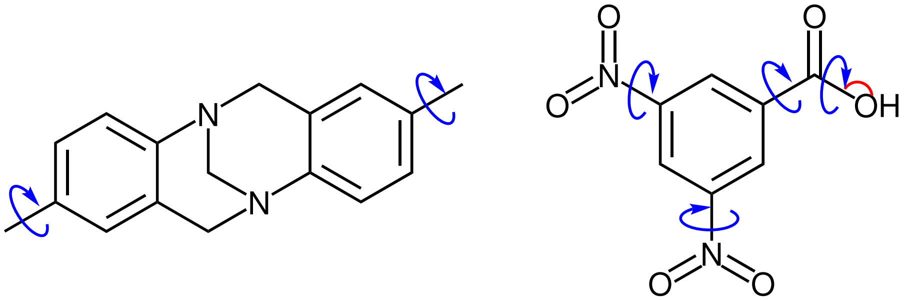

Figure 12: XXV CrystalOptimizer flexible angles (red) and torsions (blue).

5.4 Free energy calculations

The Helmholtz free energy was calculated at 300 K for all predicted structures of molecule XXV, using the revised Williams99 potential for PCM-derived multipoles. Multipoles were calculated from the B3LYP-D3(BJ)/6-311G(d,p) charge density, calculated in a polarizable continuum model with a relative permittivity of 3.0.

The **k**-point sampling method described above resulted in an average of 16.2 unique **k**-points being sampled, a minimum of 10 and a maximum of 24 **k**-points.

Common minimisation algorithms, like the BFGS-method implemented in DMACRYS, can converge to saddle points on the potential energy surface, resulting in structures that are unstable with respect to symmetry breaking. Crystal structures with imaginary phonon frequencies had their symmetry reduced. First, all internal symmetries were removed and a new calculation was performed on the $P1$ cell. If any of the supercells had imaginary phonons, the original unit cell was doubled, and calculations were performed using this doubled cell as a unit cell in $P1$ symmetry. The identification, and exclusion, of unstable structures has been recommended after such structures were submitted to previous blind tests [47].

The final list of predicted structures contains 239 structures, 208 of which are below 20 kJ/mol lattice energy and 225 are below 20 kJ/mol free energy.

A total of 1344 CPU-hours were used for free energy calculations, corresponding to an average of 5.5 CPU-hours per crystal structure. Minimum cost for any one crystal was 1.4 CPU-hours, maximum was 11.4 CPU-hours.

5.5 Final ranking of structures

We submit two sets of crystal structures for target system XXV, ranked either by lattice or Helmholtz free energy.

The two lowest ranked structures are the same in both lists. All low-energy structures have the 3,5-dinitrobenzoic acid in the A conformation.

The lattice vibrational energy causes some re-ranking among the structures. The structures below 20 kJ/mol in lattice energy were ranked by lattice energy and free energy. The Kendall's τ_b rank correlation [49] between these lists is 0.8, indicating that one fifth of all polymorph pairs are ranked discordantly when ranked by free energy instead of lattice energy.

Energy landscape figures are presented in Figures 13 and 14.

It should be noted that the free energy landscape, Figure 14 is "compressed" in energy so that there are more low free energy structures than there are low lattice energy structures, in accordance with previous observations [55, 36].

In conclusion, we predict that target XXV is most likely to crystallise in space group $P\bar{1}$, with the 3,5-dinitrobenzoic acid's carboxylic group in a planar conformation, forming a hydrogen bond to a nitrogen atom in the 2,8-dimethyl-6H,12H-5,11-methanodibenzo[b,f][1,5]diazocine molecule.

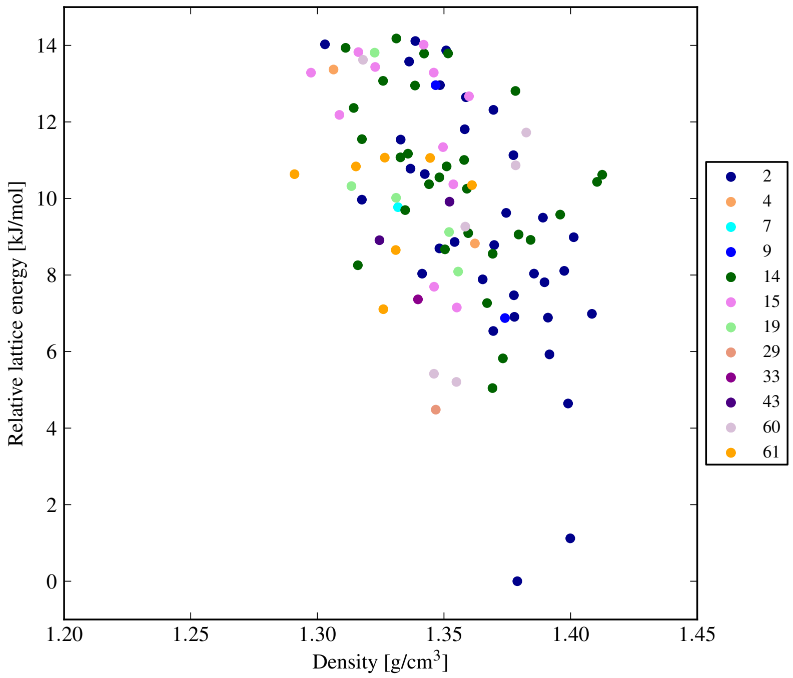

Figure 13: Lattice energy landscape of target XXV. Only the lowest 100 structures are shown. The labels are space group numbers.

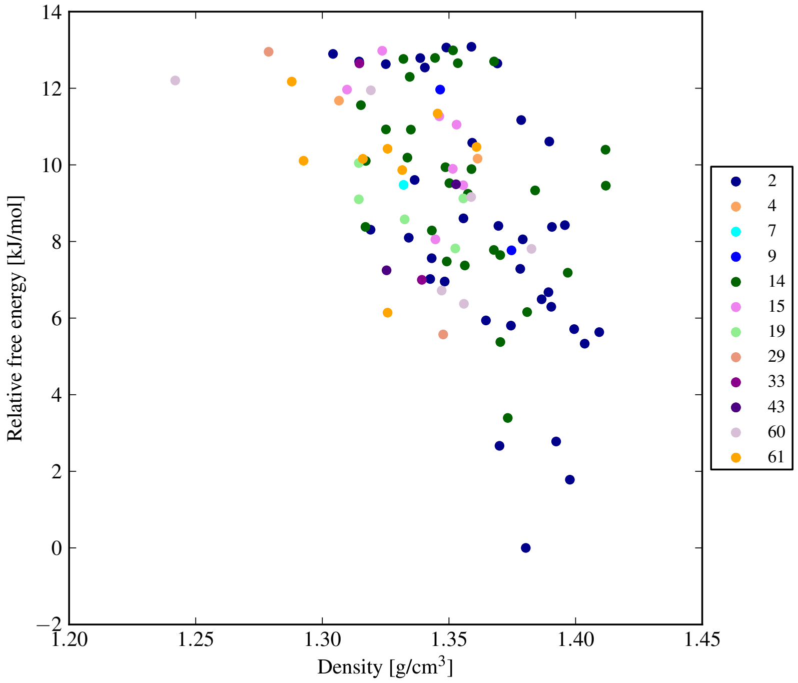

Figure 14: Free energy landscape of target XXV. Only the 100 lowest energy structure shown. Labels denote space group number.

6 Molecule XXVI

6.1 Conformational analysis

For target molecule XXVI, 96 molecular conformers were identified during the OPLS2005 conformational search. After DFT-D (B3LYP-D3(BJ)/6-311G(d,p)) a total of 40 molecular conformers exhibit relative energies, E^{rel}_{gas}, smaller than 30 kJ mol^{-1}, see Table 2. Trial crystal structure generation for molecule XXVI were generated containing molecular geometries of conformers C1, C2, ..., C40. The 41st lowest energy conformer was also included, despite its energy falling above our 30 kJ/mol cutoff. This was because this conformer was the lowest in which both amide groups were found in a cis conformation, which we thought might offer a good opportunity for hydrogen bonding. A set of distributed multipoles were subsequently generated for each conformer using our DFT-D methodology.
The XXVI conformational search and rank 4 multipole generation accounts for 3745 CPU-hours that is broken down thus: (i) initial OPLS2005 conformational search accounts for 5 CPU-hours, (ii) DFT-D geometry optimizations accounts for 3709 CPU-hours and (iii) rank 4 multipole generation accounts for 31 CPU-hours.

6.2 Details of structure generation

Rigid-molecule crystal structure generation was conducted on the 41 conformers. An initial 2000 structures were generated for each conformer in 6 common space groups (2, 4, 14, 15, 19, 61). An additional 2000 crystal structures were generated for any combination of conformer and space group that yielded crystal structures within 15 kJ/mol of the global total energy minimum (relative conformer energy + lattice energy). A further 500 crystal structures were subsequently generated for conformers that gave crystal structures within 15 kJ/mol of the global lattice energy minimum for the next 18 most common space groups (1, 5, 7, 9, 13, 18, 29, 33, 43, 56, 60, 76, 78, 88, 92, 96, 144, 145). Crystal structures within the lowest 30 kJ/mol above the global total energy minimum were clustered within each space group using our own code. This structure generation + rigid molecular lattice energy minimisation process took 150419 CPU-hours.

6.3 Details of energy minimisation

Crystal structures within 25 kJ/mol of the global total energy minimum were re-optimised with CrystalOptimizer to allow for molecular flexibility. This employed our standard DFT-D methodology (B3LYP-D3(BJ)/6-311G(d,p)) for intermolecular electrostatic interactions and PBEPBE-D3(BJ)/6-31G(d,p) for the intramolecular energy model. This lower level of theory was used for the intramolecular energy model due to the size of the molecule and requirement for Hessian calculations in CrystalOptimizer. The flexible degrees of freedom allowed in CrystalOptimizer are illustrated in Figure 15. CrystalOptimizer calculations took 22665 CPU-hours.
In addition, all final crystal structures from CrystalOptimizer were re-minimised with rigid molecular geometries, now with electrostatic models calculated from molecular calculations in a PCM. Two sets of predictions were generated, one with the dielectric constant in the PCM calculation set to $3\,\epsilon_0$ and $7\,\epsilon_0$, with ϵ_0 denoting the permittivity of free space. In this case, the higher dielectric constant cannot be justified as a realistic dielectric constant in the crystal, but allows us to examine the dependence of the predictions on the degree of polarisation of the atomic multipoles.

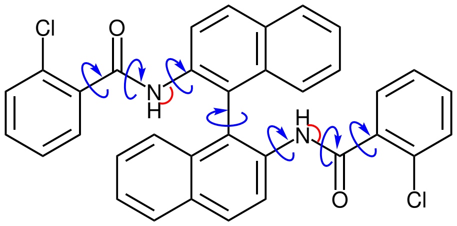

Figure 15: XXVI CrystalOptimizer flexible angles (red) and torsions (blue).

6.4 Final ranking of structures

For the two sets of structures, each of which is minimised under the PCM conditions described above, the final energy of each structure relative to the minimum in each set, is displayed in Figures 16 and 17 respectively. The low energy landscape looks similar in both cases, and an energy gap of almost 5 kJ/mol separates the lowest structure, in space group 14, from the rest of the list. This energy gap is maintained on changing the dielectric constant in the PCM calculation.

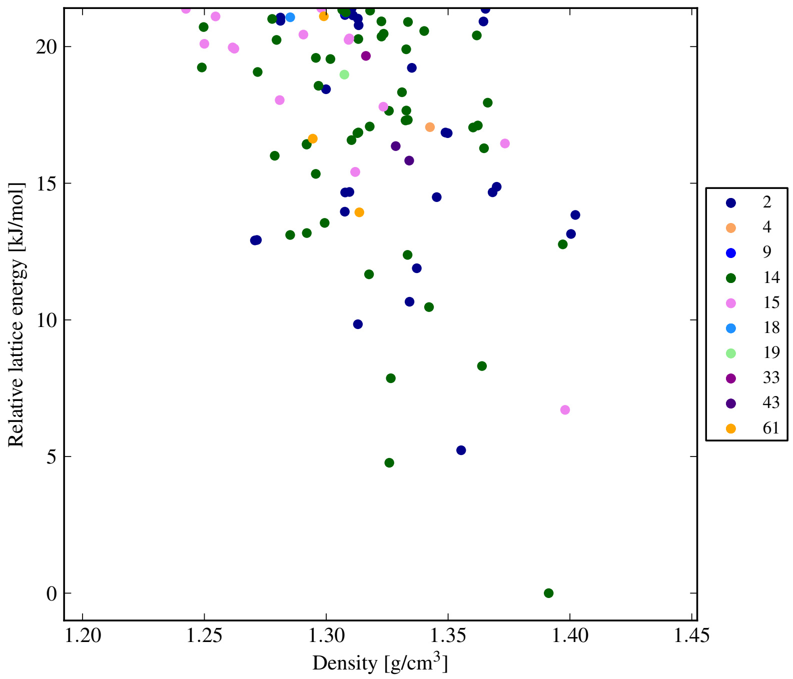

Figure 16: XXVI final structures, calculated with an electrostatic potential from a polarisable continuum model with a permittivity of $3\,\epsilon_0$

.

7 Computational resources and hardware

Force field molecular conformational searches were performed on a single workstation equipped with an Intel core i7-3770k 3.5 GHz quad-core processor.

All DFT-D re-optimization of molecular conformations, structure generation, lattice energy minimisations and free energy calculations were performed on the Iridis4 supercomputer at the University of Southampton. The computer is a Red Hat Enterprise GNU/Linux Beowulf cluster with 750 compute nodes, each with two 8-core Intel Xeon E5-2670 2.6 GHz processors. The nodes share a common global IBM GPFS file system over an FDR InfiniBand interconnect.

All flexible molecule structure generation and normal mode optimisations were performed on the Iridis3 supercomputer at the University of Southampton. The computer is a Red Hat Enterprise GNU/Linux Beowulf cluster with 980 compute nodes, each with two 6-core 2.4 GHz Intel Xeon E5645 processors. The nodes share a common global IBM GPFS file system over an FDR InfiniBand interconnect.

The computational cost of the different methods for each system is tabulated in Table 3.

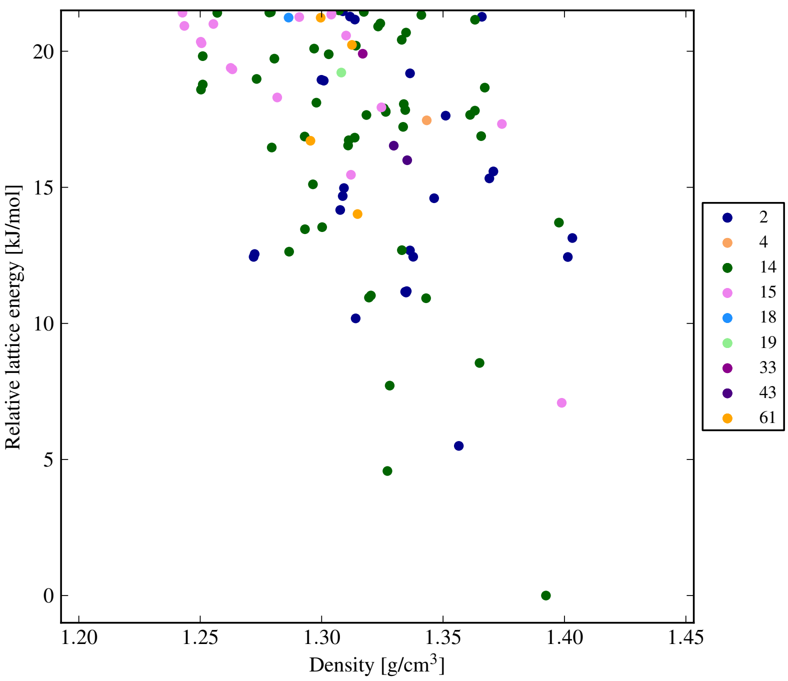

Figure 17: XXVI final structures, calculated with an electrostatic potential from a polarisable continuum model with a permittivity of $7\,\epsilon_0$

.

8 Post-analysis

Results in this section relate to analysis performed after the target crystal structures of the blind test were made available. We summarise our findings target-by-target.

8.1 XXII

The observed structure was located in both our lists, as third lowest energy structure when ranked by lattice energy (+0.65 kJ/mol above the global minimum). Upon re-ranking by free energy, the observed structure is the lowest predicted structure overall.

8.2 XXIII

Two approaches were tested for molecule XXIII. In both methods, searches were only performed with one molecule in the asymmetric unit. Our list 1 of predictions used a new flexible-molecule crystal structure generation method, followed by flexible-molecule lattice energy minimisation. Our second set of predictions (list 2) used rigid-molecule searches with a set of conformers, followed by flexible-molecule lattice energy minimisation.

All three observed Z'=1 crystal structures (forms A, B and D) were located with the flexible search (list 1). Two of these were within our 100 submitted structures: form A as 23rd in the list, 8.53 kJ/mol above the global minimum, and form D as 75th in the submitted list, 12.26 kJ/mol above the global minumum. Form B was outside the 100 submitted structures, but had been located in our search with rank 153, 15.05 kJ/mol above the global minimum. These results show that the flexible-molecule search method is very successful, but that ranking with our energy model has failed.

With the rigid-molecule search, followed by flexible-molecule lattice energy minimisation, only one of the observed Z'=1 polymorphs was located (form B as 75th in the list, 12.6 kJ/mol above

	XXII	XXIII	XXIV	XXV	XXVI
Conformational Search	1	1883	6	22	3714
Generation + rigid Opt	10127	132016	11328	113825	150419
CrystalOptimizer	854	131179	3393	5981	22665
Clustering	8	99	398	529	39
PCM + Free energy	1724	–	–	1344	–
PCM	–	2152	216	–	3060
Flexible generation + opt	–	127619	–	–	–
Total	12714	394948	15341	121701	179897

Table 3: Cost of the CSP for each target molecule broken down for each stage. All computational costs were given in CPU-hours. See Computational resources and hardware section for specific break down of the supercomputing facilities used for the blind test.

the global minimum). Both forms A and D were missed, showing the importance of including molecular flexibility at the search stage, not only during the lattice energy minimisation stage of crystal structure prediction.

For comparison of energies, lattice energy minimisation was performed on the polymorphs that were not located, using the flexibility method used for list 2 (using CrystalOptmizer, described above). These calculations also included the two observed Z'=2 polymorphs.

The final energies of the five observed polymorph of XXIII, relative to the global minumum of the List2 search, were:

A: +8.44 kJ/mol; B: +12.61 kJ/mol; C: +7.16 kJ/mol; D: +9.61 kJ/mol; E: +7.64 kJ/mol

This gives our predicted order of stability, based on lattice energies, as C <E <A <D <B.

8.3 XXIV

The observed structure was not within our submitted 100 structures in either list. However, the observed structure was located in our search, at rank 722 in our list 1 and rank 578 in list 2, with relative energies of +22.16 kJ/mol (list 1) and +21.65 kJ/mol (list 2) above the predicted global minimum. These results show that the search was successful, but our energy model fails to satisfactorily rank the structures in this case.

8.4 XXV

The observed structure was not within the structures submitted.

In post-analysis, we re-ran longer crystal structure generation calculations within the observed space group (space group 14) to examine the source of the failure. During the blind test, we have generated and successfully lattice energy minimised 20000 crystal structures in space group 14 with the relevant conformation of the acid. None of these lattice energy minimisations resulted in the observed structure. In post-analysis, 50000 crystal structures were generated and successfully lattice energy minimised in space group 14. Of these lattice energy minimisations, the observed crystal structure was reached 4 times. Our conclusion is that we terminated our search too early in preparing predictions for this target and could have predicted the observed structure if we had extended the search further. Current work is also looking at improving the efficiency of the search, to locate low energy crystal structures more frequently for this type of system.

After lattice energy minimisation using the same level of theory as used in the predictions (including molecular flexibility), the observed crystal structure had an energy 5.50 kJ/mol above the global minimum from our search, which would have ranked as 8th in our list. After free energy calculations (as used for our list 2 predictions), the observed structure is 6.15 kJ/mol above the global minimum, and would have ranked 15th.

8.5 XXVI

The observed crystal structure of XXVI was not located in our search. We believe that this is due to our crystal structure search being limited to using rigid-molecules in the structure generation

stage. We did not have time during the blind test to apply our flexible search method to XXVI. The molecular conformation in the observed crystal structure corresponds to one of the conformers that was included in the search, but is significantly distorted away from the gas phase molecular geometry.

To see how well this structure would have ranked by energy, we lattice energy minimised the observed crystal structure with the same model that was used in the blind predictions (for our list 1 predictions). The lattice energy minimised observed structure has a lattice energy 6.49 kJ/mol above the predicted global minimum and would have ranked as structure 4 in our list.

9 Acknowledgments

The Day group acknowledge the use of the IRIDIS High Performance Computing Facility, and associated support services at the University of Southampton, in the completion of this work.

We acknowledge funding from the EPSRC (grants EP/J01110X/1 and EP/K018132/1) and the European Research Council under the European Union's Seventh Framework Programme (FP/2007-2013)/ERC through grant agreements n. 307358 (ERC-stG-2012-ANGLE) and n. 321156 (ERC-AG-PE5-ROBOT).

References

[1] István Kolossváry and Wayne C. Guida. *J. Am. Chem. Soc.*, 118(21):5011–5019, 1996.

[2] István Kolossváry and Wayne C. Guida. *J. Comp. Chem.*, 20:1671–5019, 1999.

[3] Fariborz Mohamadi, Nigel GJ Richards, Wayne C Guida, Rob Liskamp, Mark Lipton, Craig Caufield, George Chang, Thomas Hendrickson, and W Clark Still. Macromodel—an integrated software system for modeling organic and bioorganic molecules using molecular mechanics. *Journal of Computational Chemistry*, 11(4):440–467, 1990.

[4] Schrodinger LLC, New York, NY, *MacroModel, V9.9.013*, 2014.

[5] William L. Jorgensen, Maxwell David S., and Julian Tirado-Rives. Development and testing of the opls all-atom force field on conformational energetics and properties of organic liquids. *J. Am. Chem. Soc.*, 118:11225–11236, 1996.

[6] George A Kaminski, Richard A Friesner, Julian Tirado-Rives, and William L Jorgensen. Evaluation and reparametrization of the opls-aa force field for proteins via comparison with accurate quantum chemical calculations on peptides. *The Journal of Physical Chemistry B*, 105(28):6474–6487, 2001.

[7] Hugh P. G. Thompson and Graeme M. Day. Which conformations make stable crystal structures? Mapping crystalline molecular geometries to the conformational energy landscape. *Chemical Science*, 5:3173–3182, 2014.

[8] Avinoam Perry. *Operations Research*, 26(6):1073–1078, 1978.

[9] M. J. Frisch, G. W. Trucks, H. B. Schlegel, G. E. Scuseria, M. A. Robb, J. R. Cheeseman, G. Scalmani, V. Barone, B. Mennucci, G. A. Petersson, H. Nakatsuji, M. Caricato, X. Li, H. P. Hratchian, A. F. Izmaylov, J. Bloino, G. Zheng, J. L. Sonnenberg, M. Hada, M. Ehara, K. Toyota, R. Fukuda, J. Hasegawa, M. Ishida, T. Nakajima, Y. Honda, O. Kitao, H. Nakai, T. Vreven, J. A. Montgomery, Jr., J. E. Peralta, F. Ogliaro, M. Bearpark, J. J. Heyd, E. Brothers, K. N. Kudin, V. N. Staroverov, R. Kobayashi, J. Normand, K. Raghavachari, A. Rendell, J. C. Burant, S. S. Iyengar, J. Tomasi, M. Cossi, N. Rega, J. M. Millam, M. Klene, J. E. Knox, J. B. Cross, V. Bakken, C. Adamo, J. Jaramillo, R. Gomperts, R. E. Stratmann, O. Yazyev, A. J. Austin, R. Cammi, C. Pomelli, J. W. Ochterski, R. L. Martin, K. Morokuma, V. G. Zakrzewski, G. A. Voth, P. Salvador, J. J. Dannenberg, S. Dapprich, A. D. Daniels, Ö. Farkas, J. B. Foresman, J. V. Ortiz, J. Cioslowski, and D. J. Fox. Gaussian 09 Revision D.01. Gaussian Inc. Wallingford CT 2009.

[10] Axel D. Becke. Density-functional thermochemistry. III. The role of exact exchange. *J. Chem. Phys.*, 20:5648–5652, 1993.

[11] Chengteh Lee, Weitao Yang, and Robert G Parr. Development of the colle-salvetti correlation-energy formula into a functional of the electron density. *Physical review B*, 37(2):785, 1988.

[12] Stefan Grimme, Jens Antony, Stephan Ehrlich, and Helge Krieg. A consistent and accurate ab initio parametrization of density functional dispersion correction (DFT-D) for the 94 elements H-Pu. *J. Chem. Phys.*, 132:154104–154104–19, 2010.

[13] Stefan Grimme, Stephan Ehrlich, and Lars Goerigk. Effect of the damping function in dispersion corrected density functional theory. *Journal of computational chemistry*, 32(7):1456–1465, 2011.

[14] I. Sobol. *USSR Computational Mathematics and Mathematical Physics*, 16:236–242.

[15] David H. Case, Josh E. Campbell, and Graeme M. Day. *In preparation.*

[16] Ken Shoemake. Graphics gems iii. chapter Uniform Random Rotations, pages 124–132. Academic Press Professional, Inc., San Diego, CA, USA, 1992.

[17] Sarah L. Price, Maurice Leslie, Gareth W. A. Welch, Matthew Habgood, Louise S. Price, Panagiotis G. Karamertzanis, and Graeme M. Day. Modelling organic crystal structures using distributed multipole and polarizability-based model intermolecular potentials. *Phys. Chem. Chem. Phys.*, 12:8478–8490, 2010.

[18] Maurice Leslie. *Advanced Users DMACRYS manual. Manual release July 2013, referring to Program release DMACRYS 2.0.8.* University College London, 2013. http://www.chem.ucl.ac.uk/cposs/dmacrys/manuals/dmacrys.pdf.

[19] A.J. Stone. Distributed multipole analysis, or how to describe a molecular charge distribution. *Chemical Physics Letters*, 83(2):233 – 239, 1981.

[20] Anthony Stone. *The Theory of Intermolecular Forces.* Oxford University Press, 2 edition, 2013.

[21] Anthony J. Stone. Distributed multipole analysis of gaussian wavefunctions. GMDA version 2.2.02.

[22] Donald E Williams. Improved intermolecular force field for crystalline oxohydrocarbons including O-H$\cdots$O hydrogen bonding. *Journal of Computational Chemistry*, 22(1):1–20, 2001.

[23] Edward O. Pyzer-Knapp. *Exploring the Crystal Energy Landscapes of Porous Molecular Crystals.* PhD thesis, University of Cambridge, 2014.

[24] NL Abraham and MIJ Probert. A periodic genetic algorithm with real-space representation for crystal structure and polymorph prediction. *Physical Review B*, 73(22):224104, 2006.

[25] Graeme M. Day and Sarah L. Price. A nonempirical anisotropic atom–atom model potential for chlorobenzene crystals. *Journal of the American Chemical Society*, 125(52):16434–16443, 2003. PMID: 14692787.

[26] AV Kazantsev, PG Karamertzanis, CC Pantelides, and CS Adjiman. CrystalOptimizer: An efficient algorithm for lattice energy minimization of organic crystals using isolated-molecule quantum mechanical calculations, 2010.

[27] Timothy G. Cooper, Katarzyna E. Hejczyk, William Jones, and Graeme M. Day. Molecular polarization effects on the relative energies of the real and putative crystal structures of valine. *Journal of Chemical Theory and Computation*, 4(10):1795–1805, 2008.

[28] B. Mennucci, J. Tomasi, R. Cammi, J. R. Cheeseman, M. J. Frisch, F. J. Devlin, S. Gabriel, and P. J. Stephens. Polarizable continuum model (PCM) calculations of solvent effects on optical rotations of chiral molecules. *The Journal of Physical Chemistry A*, 106(25):6102–6113, 2002.

[29] Maurizio Cossi, Giovanni Scalmani, Nadia Rega, and Vincenzo Barone. New developments in the polarizable continuum model for quantum mechanical and classical calculations on molecules in solution. *The Journal of Chemical Physics*, 117(1):43–54, 2002.

[30] James Alexander Chisholm and Sam Motherwell. COMPACK: a program for identifying crystal structure similarity using distances. *Journal of applied crystallography*, 38(1):228–231, 2005.

[31] Max Born and Kun Huang. *Dynamical theory of crystal lattices*, volume 188. Clarendon Press Oxford, 1954.

[32] Salvatore Califano. *Lattice dynamics and intermolecular forces*, volume 55. Academic Press, 1975.

[33] N Neto, R Righini, S Califano, and SH Walmsley. Lattice dynamics of molecular crystals using atom—atom and multipole—multipole potentials. *Chemical Physics*, 29(1):167–179, 1978.

[34] Salvatore Califano, Vincenzo Schettino, and Natale Neto. *Lattice dynamics of molecular crystals*, volume 26. Springer-Verlag Berlin, 1981.

[35] Graeme M. Day, Sarah L. Price, and Maurice Leslie. Atomistic calculations of phonon frequencies and thermodynamic quantities for crystals of rigid organic molecules. *The Journal of Physical Chemistry B*, 107(39):10919–10933, 2003.

[36] Jonas Nyman and Graeme M. Day. Static and lattice vibrational energy differences between polymorphs. *CrystEngComm*, 17:5154–5165, 2015.

[37] I. Křivý and B. Gruber. A unified algorithm for determining the reduced (Niggli) cell. *Acta Crystallographica Section A*, 32(2):297–298, Mar 1976.

[38] B. Gruber. The relationship between reduced cells in a general Bravais lattice. *Acta Crystallographica Section A*, 29(4):433–440, Jul 1973.

[39] R. W. Grosse-Kunstleve, N. K. Sauter, and P. D. Adams. Numerically stable algorithms for the computation of reduced unit cells. *Acta Crystallographica Section A*, 60(1):1–6, Jan 2004.

[40] A.L. Spek. Platon, an integrated tool for the analysis of the results of a single crystal structure determination. *Acta Crystallographica Section A: Foundations of Crystallography*, 46:c34–c34, 1990.

[41] Graeme M Day, Sarah L Price, and Maurice Leslie. Elastic constant calculations for molecular organic crystals. *Crystal Growth & Design*, 1(1):13–27, 2001.

[42] Graeme M. Day. *Lattice Dynamical Studies of Molecular Crystals with Application to Polymorphism and Structure Prediction.* PhD thesis, University College London, 2003.

[43] A. J. Stone and S. L. Price. Some new ideas in the theory of intermolecular forces: anisotropic atom-atom potentials. *The Journal of Physical Chemistry*, 92(12):3325–3335, 1988.

[44] Graeme M. Day, W. D. Sam Motherwell, and William Jones. Beyond the isotropic atom model in crystal structure prediction of rigid molecules: Atomic multipoles versus point charges. *Crystal Growth & Design*, 5(3):1023–1033, 2005.

[45] Wijnand TM Mooij and Frank JJ Leusen. Multipoles versus charges in the 1999 crystal structure prediction test. *Physical Chemistry Chemical Physics*, 3(22):5063–5066, 2001.

[46] Graeme M. Day, James Chisholm, Ning Shan, W. D. Sam Motherwell, and William Jones. An assessment of lattice energy minimization for the prediction of molecular organic crystal structures. *Crystal Growth & Design*, 4(6):1327–1340, 2004.

[47] Bouke P van Eijck. Comparing hypothetical structures generated in the third cambridge blind test of crystal structure prediction. *Acta Crystallographica Section B: Structural Science*, 61(5):528–535, 2005.

[48] Bouke P. van Eijck. Ab initio crystal structure predictions for flexible hydrogen-bonded molecules. Part III. Effect of lattice vibrations. *Journal of Computational Chemistry*, 22(8):816–826, 2001.

[49] M. G. Kendall. A new measure of rank correlation. *Biometrika*, 30(1-2):81–93, 1938.

[50] Peter J. Bygrave, David H. Case, Thomas S. Gee, and Graeme M. Day. *In preparation.*

[51] F. Pedregosa, G. Varoquaux, A. Gramfort, V. Michel, B. Thirion, O. Grisel, M. Blondel, P. Prettenhofer, R. Weiss, V. Dubourg, J. Vanderplas, A. Passos, D. Cournapeau, M. Brucher, M. Perrot, and E. Duchesnay. Scikit-learn: Machine learning in Python. *Journal of Machine Learning Research*, 12:2825–2830, 2011.

[52] Katarzyna Hejczyk. *Crystal structure modelling and predictive tools for organic molecular salt structures.* PhD thesis, University of Cambridge, 2010.

[53] A. Gavezzotti and Graeme M. Day. *J. Phys. Chem. B*, 107:2344–2354, 2003.

[54] Eloisa Angeles. *Predicting hydrate formation and stability.* PhD thesis, University of Cambridge, 2014.

[55] Manolis Vasileiadis. *Calculation of the free energy of crystalline solids.* PhD thesis, Imperial College London, 2013.
