# Supplementary material for: Report on the sixth blind test of organic crystal structure prediction methods
Source: Acta Crystallogr B Struct Sci Cryst Eng Mater. 2016 Aug 1;72(Pt 4):439–59. doi: 10.1107/S2052520616007447 (PMC4971545; doi:10.1107/S2052520616007447)
Supplement: Supplementary file 3 [file b-72-00439-sup3.zip › Group_04_Dzyabchenko_Post_Analysis.pdf]

Supporting Information Document

Section 1. List of members

Author: Alexander Dzyabchenko
Affiliation: Karpov Institute of Physical Chemistry, 10 Vorontsovo pole, 105064, Moscow, Russia

Section 2. Methodology

The target structures (XXII, XXV and XXVI) were generated with program PMC [1] for global energy minimization of general organic molecular crystal. The semi empirical intermolecular potential functions involved 6-12 Lennard-Jones atom-atom potentials for van der Waals energy and electrostatic interactions of point charges placed on atoms or auxiliary sites. The intra-molecular energy, in addition to the van der Waals and electrostatic terms, involved harmonic potential functions to connect bonding atoms and auxiliary force centers of constituting rigid fragments into the conformationally flexible molecule. Another component of the intramolecular energy was empirical torsion potential of the form $v(\tau) = v_0 \sin^2\tau$ to respect for π-electron conjugation effect of aromatic fragments.

An essential note is that both intra- and intermolecular contributions to the total energy from the van der Waals and electrostatic terms were calculated with the same set of atom-atom potential parameters and electrostatic charges.

The electrostatic point charges were obtained by approximation of quantum mechanical electrostatic potential of the molecule (as obtained from ab initio MO LCAO calculation [2]) with analytical potential of point charges on program FitMEP [3]. The fit charges were not constrained to occur on atomic positions but were allowed in the least square procedure to shift away in the three dimensions, giving rise to significant improvement in the fit quality. Thus, the best off-atomic charge model found for all target molecules of his test were at least several times as good in RMSD as compared to the models with charges fixed on atoms. (Rather, with regard to XXV and XXVI, we still used plain atomic charge models due to fewer numbers of interaction centers characteristic to them, thus making it possible gain computational effort in considerable amount).

The intramolecular structure of molecule XXII was found non-planar, with the fold angle of 41 deg, on the basis of geometry optimization on Gaussian 98 [2] by the Hartry-Fock method with 6-31G(d,p) basis functions. This geometry was used unchanged throughout the crystal packing calculations. (An alternative planar model, although unstable in gas state, may be probably stabilized due to the crystal packing, since the calculated energy difference of planar and bent shapes was found only 1.7 kcal/mol. However, no effort to check this possibility was attempted here).

In the molecular complex XXV, the larger component (Troger's base, TB) was modeled by three rigid fragments: one central tetracycle and two methyl substituent. Each fragment was supplied with auxiliary force centers (X) in place of carbon of another fragment bonded to it [4]. The X-center of each fragment was linked to the carbon of another fragment participating in the chemical bonding via the harmonic potential (HP) with zero equilibrium distance. The two other kinds of harmonic potentials respect for the C-C bonding distance and the next-neighboring C...C separation, controlling the C-C-C valence angle. Both X-CH_3 groups were thus free to rotate about the C-C bonds under the action of intra- and intermolecular forces.

As to the second component of XXV, dinirobenzoic acid (DNBA), was also modeled with three rigid fragments of planar shape: the fragment of benzoic acid (BA) and two nitro substituents. Like with the TB molecule, the BA and nitro fragments were supplied with auxiliary X force centers to make flexible connection of the three fragments. Furthermore, both nitro connections were also supplied with the torsion potential to control their rotation amplitude.

As to molecule XXVI, consisting of two symmetrical parts of identical chemical structure, we assumed its electrostatic model under the suggestion that the QM-optimized bond lengths and angles and electrostatic charges, elaborated for one half of the molecule (with the methyl substituent in place of the other half), is a good approximation for both. Each half was modeled as three rigid fragments: the naphthyl, amide and chloropenzene groups having planar geometries and connected by virtue of harmonic potentials involving X-force centers.

The staring conformation of the dimer molecule was taken as both halves perpendicular to each other, each half planar in itself, NH *cis* to binaphthyl link and to the chlorine atom.

The global energy minimization was confined to the consideration of the several most likely organic crystal structural classes (determined by space group and the number of molecule in the unit cell, *Z*). In particular, the space groups P-1, $P2_1$, Pc, $P2_1/c$, $C2$, Cc, $P2_12_12_1$, $Pca2_1$, $Pna2_1$, $Pbca$, $C2/c$ were tried for XXII and XXVI. Additionally, the four monoclinic groups containing glide plane *c* were completed with the *same* groups but referred to an *alternative choice* of the crystal axes: Pn, $P2_1/n$, Cn and $C2/n$, in which the diagonal *n* plane replaces *c* plane. Furthermore, the two *C*-centered monoclinic groups are completed with the same ones in one more alternative setting: *I*2. *Ic* and *I*2/*c*. The reason for use of such different settings of the monoclinic groups in the same study is that allows one to avoid lattice geometries too far from rectangular.

As concerns to XXV, the centrosymmetric *P*-1 and $P2_1/c$ groups were only considered here as most likely ones consistent with the idea of the occurrence in the crystal packing of intermolecular pairs of carboxyl groups, cyclically closed by hydrogen bonding about the crystallographic inversion centers. (Moreover, all starting configurations of this system were built in such a way that the carboxyl group was placed enough close the inversion centre of the crystal to form such cyclic H-bonded configurations on the very first steps of structure optimization.

The starting sets of the molecular center-of-mass coordinates were selected from the following list: 0.0,0; 1/4,0,0; 0,1/4,0; 0,0,1/4; 0,1/4,1/4; 1/4,0,1/4; 1/4,1/4,0; 1/4,1/4,1/4, depending on the postulated space group. Thus, positions coincident with the inversion center or twofold axis are evidently prohibited; in the event of the crystal origin not fixed by the space group in one ($P2_1$, $C2$, $I2$, $Pca2_1$, $Pna2_1$) or two dimensions (Pc, Pn, Cc, Cn, Ic) the sets containing non-zero components in the non-fixed-origin directions should be omitted as well.

The starting unit cell parameters were selected in such a way that the crystal density would not exceed approximately 50% of the final density anticipated for best-energy structures. Typically, the following orthogonal cell shapes were generated in turn: the first one is cubic with period *a*, the second is tetragonal and flat, with the parameters $a' = a/2$, $b'=c'=2^{1/2}a$, while the third one is of tetragonal elongated shape: $a' = 2a$, $b'=c'=2^{-1/2}a$. The other four cells have of the same two shapes as before but oriented perpendicular (or along) to other two dimensions.

The starting molecular orientations were selected from the list of 1080 matrices providing a kind of regular 'grid' of the full rotation space in the three dimensions (with the separation angle ω = 27.78° between any two nearest-neighbor orientations). On account of the crystal and approximate molecular symmetry the number of starting orientations for global minimization is effectively reduced [5]. With regard to XXV, the number of starting orientations of one component (TB) was restricted to twelve.

The starting models were optimized by energy with regard to the six rigid body parameters per each molecular fragment and up to twelve cell dimensions depending on the selected space group.

The energy-minimized structures were sorted by energy within each space group and treated with program CRYCOM of crystal structure comparison [6] in terms of rigid body parameters for molecule (XXII) or the several independent fragments (in XXV and XXVI) to select the lists of unique structures. The three lists of the up to 100 lowest-energy structures found in different space

groups, with top structures first, have been submitted as our predictions for targets XXII, XXV and XXVI.

Section 3. CPU resources and hardware used

Most calculations have been performed on the computer 'mvs100k' of the Joint Supercomputer Center of the Russian Academy of Sciences (JSCC RAS) [7]. The numbers of parallel processors, N_p, of Intel Xeon 5450 type (operating on GHz) used in these computations varied from 48 to 96. The CPU times (as rescaled to N_p = 96) have amounted approximately 1.5, 38 and 35 hours for XXII, XXV and XXVI target structures, respectively.

References

1. Dzyabchenko, A. V. (2008). From molecule to solid: the prediction of organic crystal structures. *Russ. J. Phys. Chem. A*, **82**(10), 1663–1671.
2. Frisch, M. J., Trucks, G. W., Schlegel, H. B., Scuseria, G. E., Robb, M. A., Cheeseman, J. R., Zakrzewski, V. G., Montgomery Jr., J. A., Stratmann, R. E., Burant, J. C., Dapprich, S., Millam, J. M., Daniels A. D., Kudin K. N., Strain M. C., Farkas O., Tomasi J., Barone V., Cossi M., Cammi R., Mennucci, B., Pomelli, C., Adamo, C., Clifford, S., Ochterski, J., Petersson, G. A., Ayala, P. Y., Cui, Q., Morokuma, K., Rega, N., Salvador, P., Dannenberg, J. J., Malick, D. K., Rabuck, A. D., Raghavachari, K., Foresman, J. B., Cioslowski, J., Ortiz, J. V., Baboul, A. G., Stefanov, B. B., Liu G., Liashenko, A., Piskorz, P., Komaromi, I., Gomperts, R., Martin, R. L., Fox, D. J., Keith, T., Al-Laham, M. A., Peng, C. Y., Nanayakkara, A., Challacombe, M., Gill, P. M. W., Johnson, B., Chen W., Wong, M. W., Andres, J. L., Gonzalez, C., Head-Gordon, M., Replogle, E. S., & Pople, J. A. (2002). Gaussian 98 Revision A.11.3. Gaussian Inc. Pittsburgh PA.
3. Dzyabchenko, A. V. (2008). A multipole approximation of the electrostatic potential of molecules. *Russ. J. Phys. Chem. A*. **82**(5), 758–766.
4. Dzyabchenko, A , Scheraga, H.A. (2004). Model for the crystal packing and conformational changes of biphenyl in incommensurate phase transitions. *Acta Cryst. B*, **60**(2), 228-237.
5. Dzyabchenko, A.V. (1983). Symmetry of the lattice-energy functional of a molecular crystal. *Acta Cryst. A*, **39**(6), 941-946.
6. Dzyabchenko, A.V. (1994). Method of crystal-structure similarity searching. *Acta Cryst. B*, **50**(4), 414-425.
7. http://www.jscc.ru/hard/mvs100k.shtml

Post-analysis

Overall results. The structure submission of target XXII at rank 1 is nearly identical to the observed structure, whereas neither entry in the submissions of other two attempted targets, XXV and XXVI, shows any similarity with experimental structures.

XXII

Program CRYCOM [6] identifies the rank-1 structure as nearly identical to the experimental one and provided deviations in respective structural parameters (Table 1)

Table 1. Deviations of rank-1 structure from experiment

$\Delta a/a$	$\Delta b/b$	$\Delta c/c$	$\Delta\beta/\beta$	$\Delta V/V$	T, Å	Ω, deg	S_{crys}, Å	S_{mol}, Å
0.4	1.6	-0.4	-1.8	2.7	0.097	1.3	0.117	0.060

$\Delta p/p = 100\%(p_{obs} - p_{pred})/p_{obs}$ are relative deviations in the unit-cell constants and volume; T and Ω are rigid-body translation and rotation angle that is necessary to obtain the best fit of the two sets of atomic coordinates in orthogonal space.

S_{crys} is the root-mean-square deviation in atomic positions calculated as

$$S = (S_x^2 + S_y^2 + S_z^2)^{1/2} \text{ , where}$$

$$S_x = a_{obs} \sin\beta_{obs} [N^{-1}\Sigma(x_{obs} - x_{pred})^2]^{1/2};$$

$$S_y = b_{obs} [N^{-1} \Sigma(y_{obs} - y_{pred})^2]^{1/2};$$

$$S_z = c_{obs} [N^{-1}\Sigma(z_{obs} - z_{pred})^2]^{1/2};$$

x, y, z are fractional coordinates; N is the number of atoms in the asymmetric unit.

S_{mol} characterizes deviations in the molecular structure that are mainly due to conformational difference: the dihedral angle between two aromatic planes in the predicted structure is 3.5° degrees smaller than the experimental value 44.4°.

Note that S_{mol} has the same sense as $RMSD_1$ in the Crystal Packing Similarity Tool (Chisholm & Motherwell, 2005). This is not the case with S_{cryst} which is necessarily smaller than $RMSD_n$ since the latter is calculated over n molecules separated by fraction(s) of lattice translation(s) or even integer translation(s): consequently, deviations in lattice constants contribute to the differences in atomic positions calculated in orthogonal space.

XXV and XXVI

The post analysis of these two attempted target molecules is currently in work. Its detailed analysis will be discussed elsewhere.

Acknowledgement

I am grateful to Mikhail Kuzminskii for calculations of molecular structures on Gaussian 98 program in the Institute of Organic Chemistry RAS. The Russian Foundation for Basic Research is acknowledged for financial support (14-03-01091).

Appendix

Here we provide more details on the intermolecular potentials we used in structure generation and ranking of target XXII.

The point charge model for calculations of electrostatic energy was elaborated with a procedure (FitMEP [3]) in which the sum of weighted squares of deviations from the ab initio potential of analytical potential of point charges was minimized with respect to charges magnitudes and charge-site coordinates as independent variables. Initial positions of charge sites were just atomic positions, while initial charge magnitudes were best charges found with regular atomic charge model (BAC, RRMSD = 13.5%). On final steps of optimization three extra charge sites, were introduces to give some additional gain in the accuracy approximation. (On the other hand, site XC6 originated from atom C6 was finally eliminated from the coordinate list since its charge magnitude converged to zero. Eventually the 18-center off-atomic point charge model (OAC) of RRMSD = 1.48 % was regarded enough accurate to use it in the calculations of electrostatic energy. One can get an idea on how the off-atomic charges are arranged about the atomic molecular cage from Fig. 1.

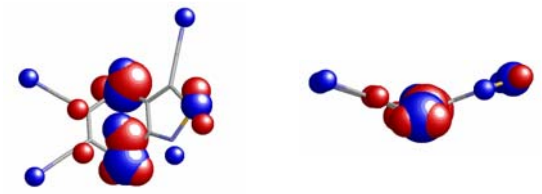

Figure 1. Graphical representations of the off-atomic charge model (OAC) used in the structure prediction of XXII. The volumes of spheres are proportional to charge magnitudes (Table A1).

For calculation of van der Waals contribution to the total energy, we used an updated set of Lennard-Jones 6-12 (C,N,S) potential parameters (Table A2). This set was elaborated in a preliminary step in which energy-minimized crystal-structure parameters and lattice energies calculated with trial sets of potential parameters (and most precise point charges based on MEP approximation) were required to correspond as close as possible to the experimentally observed quantities. The training set of structures consisted of a few C, N, S - compounds: cyclo-octasulfur, tetracyanoethylene, tetratiafulvalene, 1,4-dithiine-2,3,5,6-tetracarbonitrile and the fullerene-C_{60}:$2S_8$ clathrate. Moreover, experimental hits of sublimation were available from literature for the first three compounds of this set. Table A2 reports the parameters of the Lennard-Jones 6-12 potential in terms of equilibrium distance r_m and potential well depth ε. (Full detail of this work will be given in a separate paper which is currently under work).

.

Table A1. Orthogonal coordinates (Å) and point charges (*e*) of atoms and the auxiliary charge sites in the off-atomic point charge model (OAC) found best to approximate the ab initio MEP

	x	*y*	*z*	Charge
Atoms:				
C1	1.96577	-0.47373	0.09059	0
C2	1.62984	0.81391	0.15672	0
C3	-0.64207	-1.21940	0.17912	0
C4	-0.99229	0.17039	0.24256	0
N5	-1.56029	-2.02826	-0.18886	0
C6	-2.28447	0.31587	-0.12569	0
S7	0.95641	-1.83763	0.61636	0
S8	0.09427	1.43627	0.79825	0
S9	-2.96674	-1.22565	-0.48479	0
C10	2.54014	1.84582	-0.26717	0
N11	3.23472	2.68762	-0.57908	0
C12	3.25517	-0.87554	-0.41116	0
N13	4.26572	-1.22944	-0.78760	0
C14	-3.03643	1.52942	-0.20801	0
N15	-3.64829	2.48319	-0.27574	0
Charge sites:				
XC1	0.43773	-0.93594	0.38993	2.08158
XC2	0.58705	-0.50346	0.18499	-0.98181
XC3	-3.19437	-1.11764	-0.54050	-0.78581
XC3'	0.58023	-2.29116	0.81870	1.01084
XC4	-0.44840	1.41898	0.57826	2.39021
XC4	-0.41084	1.87045	0.36441	-0.79953
XN5	-1.45034	-2.77376	-0.24566	-0.18189
XS7	0.88224	-1.86681	0.71640	-2.64005
XS7'	1.57194	-1.92445	0.83300	0.70736
XS8	0.73250	1.68400	0.76364	0.55697
XS8'	-0.07241	1.22131	0.75284	-2.15261
XS9	-3.19848	-2.07261	-0.64427	0.29226
XC10	1.97569	0.98491	-0.11352	0.24708
XN11	3.48257	3.01266	-0.69358	-0.21813
XC12	2.39866	-0.98074	-0.14961	0.25003
XN13	4.62704	-1.37761	-0.92652	-0.22147
XC14	-3.37077	-0.52927	-0.52809	0.64626
XN15	-3.92267	2.90112	-0.29568	-0.20129

TableA2: Parameters of the 6-12 Lennard-Jones atom-atom potentials used with XXII

Atoms		r_m, Å	ε, kcal/mol
C	C	3.700	-0.0722
C	N	3.650	-0.100
C	S	3.910	-0.189
N	N	3.950	-0.110
N	S	3.975	-0.183
S	S	4.000	-0.306
