# Supplementary material for: Report on the sixth blind test of organic crystal structure prediction methods
Source: Acta Crystallogr B Struct Sci Cryst Eng Mater. 2016 Aug 1;72(Pt 4):439–59. doi: 10.1107/S2052520616007447 (PMC4971545; doi:10.1107/S2052520616007447)
Supplement: Supplementary file 3 [file b-72-00439-sup3.zip › Group_05_vanEijck_Post_Analysis.pdf]

Blind test 2015, van Eijck (UPACK)

1 Introduction

This contribution describes the results of using the UPACK program package [1]. It was designed to generate a list of possible crystal structures by a random search method, using an empirical force field. Soon it became obvious that the ranking in such a list is generally inadequate. Therefore, the crystal structure generation was followed (if possible) by more sophisticated calculations. An anisotropic intermolecular potential was implemented by Mooij [2] into the TINKER package, using ideas developed by Price and Stone. For flexible molecules, ab-initio intramolecular energies [3] were sometimes used. Later, blind tests have suggested that an entirely different approach, based on DFT-D calculations [4], performs at least as well.

It thus appears that for general use the UPACK program, as available to the scientific community [5], is now obsolete with regard to sufficiently accurate energy calculations. The follow-up using the programs XTINKER, GAMESS and MOLDEN is not well documented and unavailable to other researchers for copyright reasons. Still, a sufficiently reliable list of potential structures can always be used as starting point for more sophisticated calculations, as preferred by the user.

Therefore, it was decided to concentrate for this blind test on the first stage, the crystal structure generation. Even that may be difficult enough. Already in the fifth blind test UPACK failed to find the structure of the 'monster' compound XX (55 atoms). It will be seen that for the large molecules in the present test similar difficulties occur.

This more limited goal is compatible with the change of the rules for the blind test. Previously there was a sharp definition of successful crystal structure prediction: the experimental structure should be among the first three submitted structures. Of course, that is still highly desirable, but each participant is now only asked to provide a list of potential structures with estimated ranking.

2 Intermolecular force field

To assess the quality of several possible force fields, the results of the previous blind tests were reexamined. For the nonbonded parameters either Jorgensen's OPLS Lennard-Jones (LJ) potential [6-9] or the Price-Williams (PW) Buckingham potential [9-13] was used. Details of the latter force field, which is the one used for the final calculations in this work, are given in the Appendix.

In the simplest approach fixed point charges were taken for all structures. At the other extreme, the most sophisticated calculations used the XTINKER package with individual multipoles, ab-initio calculations of intramolecular energies and forces [2], and the PW potential for intermolecular van der Waals interactions (MP/AI). In cases where that procedure was too time-consuming, the intramolecular nonbonded energy was calculated by the force field (MP/PW) or the multipole approach was abandoned in favour of individual point charges, with intramolecular PW forces and ab-initio energies (PC/PW).

The latter approach has the advantage of saving considerable computing time, without the need for a complex multipole algorithm. The charges can be either calculated "on the fly" as a

subroutine in UPACK or separately before and after a cycle of energy minimization. The latter approach allows other researchers to use UPACK without including external program packages. It is, however, not a genuine energy minimization, as the intramolecular forces are not derivatives of the corresponding 6-31G** energy. It was hoped that this approach would still converge to an acceptable minimum and diminish errors in the torsional potentials, albeit at the cost of neglecting intramolecular dispersion.

To verify this idea, all sets of structures from the previous blind tests were recalculated in this PC/PW approach, as well as simply in the PW force field. The bonded parameters were taken without change from the original calculations. Table 1 shows the results, together with the original submissions for the blind tests. As expected, they get better when going from PW through PC/PW to "submitted". Especially the MP/AI results form a significant improvement over the other two, but the PC/PW results also provide acceptable rankings. The exceptions are compounds IV and XVII. Thus PC/PW was taken as the final force field for this blind test. As previously, no corrections to free energies [14,15] were attempted.

Table 1. Reconsideration of previous results. For explanation of the symbols in the last column, see text. The data under headings PW and PC/PW were recalculated in this work.

Compound	PW		PC/PW		As published for blind tests 1-5		
	R	ΔE	R	ΔE	R	ΔE	Details for the submissions
I-form 1	11	0.7	1		3	0.0	LJ
I-form 2	134	5.0	113	6.1	57		LJ
II	Not attempted						
III	2	1.0			1		LJ
IV	28	3.3	64	9.7	6	2.0	MP/AI
V	4	1.6			1		LJ; free energy; distorted structure
VI	171	17.9	107	16.8	358	20.1	LJ; free energy
VII	12	0.5	8	0.4	5	0.2	LJ
					1		MP/AI (calculation by Mooij)
VIII	11	1.3	9	1.9	1		MP/AI; not really blind result
IX	Not found						
X	137	6.8	68	35.9	135	7.1	PW
XI	48	1.5	16	0.6	30	1.0	MP/AI; $Z'' = 2$
XII	7	1.3	6	1.2	6	0.6	MP/AI
XIII	16	2.5			16	2.5	PW
XIV	11	4.6	3	0.3	1		MP/PW
XV	3	4.8	2	2.4	3	1.4	MP/PW
XVI	1		1		2	0.1	MP/AI
XVII	73	9.4	38	7.8	6	3.7	MP/AI
XVIII	Not found						
XIX	40	8.3	2	1.2	2	1.2	PC/PW
XX	Not found						
XXI	20	10.8	6	3.5	1		MP/AI; hydrogen positions doubtful

3 Procedure and results

For each compound parameters for bond lengths, bond angles and torsional angles were estimated. To this end, molecular models were constructed by geometry optimization of the free molecules, or suitable fragments, at the 6-31G* level. Generally the results were comparable to average geometrical parameters from the CSD database [16]. Force field parameters for bond lengths and bond angles were adjusted to reproduce these geometries, and reasonable values were guessed for the corresponding force constants.

All torsional angles involving sp^2 atoms were restrained to planarity with aid of a harmonic or a twofold torsional potential. Further conclusions and assumptions for less obvious torsional parameters are given in Table 2. Torsional parameters were chosen accordingly.

Table 2. Definitions and assumed properties of dihedral angles.

Compound	Definition	Properties
XXII	Ring C – S – C – C	$\pm$ 30-40°
XXIII	ω_1 = C(Ph) – C – O – H	Planar *trans*
	ω_2 = C(Ph) – C(Ph) – C – O	Planar
	ω_3 = C(Ph) – N – C(Ph) – C(Ph)	Weakly planar
	ω_4 = C(Ph) – C(Ph) – C – C	Free
	ω_5 = C(Ph) – C – C – C(Ph)	Some *trans* preference
XXIV	ω_1 = C – C – O – H	Planar *trans*
	ω_2 = C = C – C – O	Planar *trans*
	ω_3 = C = C – S – C	Free
	ω_4 = C – S – C – N	Free
	ω_5 = S – C – N – H	Planar
XXV	Central 6-membered ring	No torsional parameters
	ω_1 = H(Me) – C – C(Ph) – C(Ph)	Free
	ω_2 = C(Ph) – C – O – H	Planar *trans*
	ω_3 = C(Ph) – C(Ph) – C – O	Weakly planar
	ω_4 = C(Ph) – C(Ph) – N – O	Weakly planar
XXVI	ω_1 = C(Ph) – C(Ph) – C = O	Free
	ω_2 = C(Ph) – N – C = O	Planar
	ω_3 = C(Ph) – C(Ph) – N – C	Free
	ω_4 = Central C(Ph) – C(Ph) – C(Ph) – C(Ph)	Free

The random search technique implemented in the UPACK program package [1,5] was used. Here the Lennard-Jones force field was used to ensure that short distances remained always repulsive. Atomic point charges were calculated once and used throughout in this preliminary stage. In the final stage the program GAMESS-UK[17] was used for the PC/PW calculations. Point charges were calculated from ESP fittings, using MOLDEN [18] on 6-31G** wave functions for each individual structure. All work was done with fully flexible molecules.

In compound XXIV a chlorine ion is present. The Jorgensen group [19] has published Lennard-Jones parameters that differ from those for a bonded Cl atom. This parameter set was used in the structure generation. Afterwards the ranking of structures was recalculated with the bonded parameters, and found to be not dramatically different. So in the end the standard force field was used for this compound too.

The first stage of the work is generation of possible crystal structures. For each compound 5000 structures with one independent molecule (or set of molecules) in the asymmetric unit were created in the thirteen space groups $P2_1/c$, $P\overline{1}$, $P2_12_12_1$, $P2_1$, $Pbca$, $C2/c$, $Pna2_1$, Cc, $Pca2_1$, $C2$, $P1$, $Pbcn$, and Pc. If necessary and possible, this number was increased to 10000 in subsequent runs. The generated structures were subjected to a preliminary energy minimization. For the most promising structures the optimization was continued with the Buckingham PW potential, which generally appears to perform somewhat better. After each minimization equivalent structures were removed by clustering.

All calculations were done for $Z' = 1$, so no attempt was made to find the two $Z' = 2$ polymorphs of compound XXIII.

In each stage one should inspect the resulting structures to be reasonably sure that the list is complete. All low-energy structures should have been found several times. This was only the case for compound XXII. For the other compounds the calculations were rather tedious, especially for the space groups with eight equivalent positions in the unit cell. For compounds XXV and XXVI not even the target of 5000 structures could be obtained in these three space groups (and a few other ones) within an acceptable time. Thus the preferred approach, where all torsional degrees of freedom are set to random values in each starting structure, had to be abandoned. Considering the conformations encountered in the preliminary efforts, combined with the information from Table 2, several starting dihedrals were preset to probable values (and then allowed to optimize, as always).

Even then, the resulting lists were obviously incomplete as most low-energy structures were found only once. Nevertheless, some indication of the space group frequencies was now available for each compound. So a second cycle of structure generation was performed with more structures, but limited to the most probable space groups. Generally the numbers and/or values of the starting dihedral angles were adjusted too.

Many new structures were found in this way, and it turned out to be desirable to continue the calculations in a third cycle. In each cycle, the most promising structures were taken over to the final stage where PC/PW calculations were performed as explained above. Ideally, in the end no new structures with low ranking should have been produced, but see below. Details of the calculations are given in Table 3.

Table 3. Summary of computational details.

	XXII	XXIII	XXIV	XXV	XXVI	Total
Cycle 1						
G	120000	310000	75000	107000	47000	
CPU	50	1200	540	3520	2400	7710
Cycle 2						
G	10000	40000	235000	46000	45000	
Best new rankings	>145	6,7...	1,3...	1,2...	4,5...	
CPU	10	570	360	1780	2580	5300
Cycle 3						
G		80000	125000	96000	33000	
Best new rankings		4,6...	11,12...	2,3,...	6,16...	
CPU		380	170	2110	950	3610
N	1189	4135	3243	5814	1300	
Total CPU stage 1	60	2150	1070	7410	5930	16620
PC/PW calculations						
N	252	614	3067	693	470	
Total CPU stage 2	70	660	330	650	1700	3410
Grand total CPU	130	2810	1400	8060	7630	20030

G is the number of structures generated in each of the three cycles of stage 1. *N* is the final number of structures in each stage of the calculations. Various computers were used; CPU is the computing time (hours) standardized for one 2.66 GHz Intel Quad 9400 processor at Utrecht university.

4 Discussion

As stated in the Introduction, the search problem was central in this work. Only for compound XXII appears the probability of finding the experimental structure to be good. The other compounds present a search problem in many dimensions, as shown in Table 4. In earlier work [1] we have noted that searches started to fail when *D*, the number of variables, is around 20. Of course, computer power has increased enormously since the year 2000, but compound XXIV is still a formidable challenge.

Table 4. Degrees of freedom (*D*) of the search problems.

	XXII	XXIII	XXIV	XXV	XXVI
Cell	4	4	4	4	4
Position and orientation	6	6	15	12	6
Relevant dihedral angles	0	7	4	4	7
Total dimensionality *D*	10	17	23	20	17

The crystallographic parameters are given for $P2_1/c$, but they may be different for other space groups. For instance, $P1$ has 6 cell parameters but 3 position parameters can be chosen freely.

The problem is compounded by the transition from the PW structures, as generated, to the PC/PW structures for the final lists. Except for compound XXII, the RMS differences between these data sets are rather large. This is caused predominantly by the intramolecular energies. An interesting case is compound XXVI, where intramolecular hydrogen bonds are possible. In the PC/PW list the first 100 structures all contain N–H...O, N–H...Cl or C–H...O links, whereas in the PW list structures without such bonds appear already at rank 5. In compounds XXIII – XXVI high-energy structures that were almost discarded from the search occasionally produced quite favourable PC/PW energies. As seen in Table 3, new cycles of structure generation continued to present new low-energy structures, showing that failure is a real possibility.

5 Acknowledgements

Toine Schreurs provided computer facilities and assistance. I am grateful to Matthew Habgood at AWE company for providing a travel grant.

6 References

[1] B. P. van Eijck and J. Kroon, Acta Cryst. **B56** 535-542 (2000).
[2] W. T. M. Mooij, B. P. van Eijck and J. Kroon, J. Phys. Chem. **A103**, 9883-9890 (1999).
[3] B. P. van Eijck, W. T. M. Mooij and J. Kroon, J. Comput. Chem. **22** 805-815 (2001).
[4] M. A. Neumann, F. J. J. Leusen and J. Kendrick, Angew. Chemie Int. Ed. **47**, 2427-2430 (2008).
[5] B.P. van Eijck, http://www.crystal.chem.uu.nl/∼vaneyck/upack.html
[6] W. L. Jorgensen and D. L. Severance, J. Am. Chem. Soc. **112**, 4768-4774 (1990).
[7] W. L. Jorgensen, D. S. Maxwell, and J. Tirado-Rives, J. Am. Chem. Soc. **118**, 11225-11236 (1996).
[8] E. K. Watkins and W. L. Jorgensen, J. Phys. Chem. **A105**, 4118-4125 (2001).
[9] B. P. van Eijck, Phys. Chem. Chem. Phys. **4**, 4789-4794 (2002).
[10] D. E. Williams and D. J. Houpt, Acta Cryst. **B42**, 286-295 (1986)
[11] S. L. Mayo, B. D. Olafson, and W. A. Goddard III, J. Phys. Chem. **94**, 8897-8909 (1990).
[12] D. S. Coombes, S. L. Price, D. J. Willock, and M. Leslie, J. Phys. Chem. **100**, 7352-7360 (1996).
[13] T. Beyer and S. L. Price, J. Phys. Chem. **B104**, 2647-2655 (2000).
[14] B. P. van Eijck, J. Comput. Chem. **22** 816-826 (2001).
[15] B. P. van Eijck, W. T. M. Mooij and J. Kroon, J. Phys. Chem. **B105**, 10573-10578 (2001)
[16] F. H. Allen, Acta Cryst. **B58**, 380-388 (2002).
[17] M. F. Guest et al., Mol. Phys **103**, 719-747 (2005).
[18] G. Schaftenaar and J. H. Noordik, J. Comput.-Aided Mol. Design, **14**, 123-134 (2000).
[19] J. Chandrasekhar, D. C. Spellmayer and W. L. Jorgensen, J. Am. Chem. Soc. **106**, 903-910 (1984).

7 Appendix

For completeness sake, the details of the PW force field are given here. The Buckingham potential has the form

$$U_{Buck} = A\exp(-Br) - \frac{C}{r^6} \quad (1)$$

and the parameters are:

	A (kcal mol^{-1})	B (Å^{-1})	C (kcal mol^{-1} Å^6)	Reference
H (on N)	1202	4.66	5.14	12
H (on O)	541	4.66	5.14	13
H (on C)	2861	3.74	32.6	10
C	88371	3.60	583.1	10
N	60834	3.78	329.4	10
O	54987	3.96	268.5	10
F	86932	4.16	201.7	10
S	55988	2.98	2947.3	11
Cl	227200	3.25	3128	9
Br	287800	3.25	3962	9
I	287800	3.14	4878	9

8 Correspondence

The author is retired from Utrecht University, The Netherlands.
Correspondence address: b.p.vaneijck@chem.uu.nl

9 Post-analysis

After the submissions discussed above, full details of the observed structures became available. The experimental structures were energy-minimized to find the most closely corresponding candidates in the lists of predicted structures. The rankings and energy differences with respect to the global minima are given in Table 5. The predicted structures were subjected to appropriate symmetry transformations in order to compare the calculated and observed structures in detail. The results are also given in Table 5.

Table 5. Results of the crystal structure predictions.

Compound	XXII	XXIII-a	XXIII-b	XXIII-d	XXIV	XXV	XXVI
Space group	$P2_1/n$	$P2_1/c$	$P\bar{1}$	$P2_1/n$	$P2_1/c$	$P2_1/c$	$P\bar{1}$
Intermediate ΔE (PW)	3.5	12.4	5.4	11.9	18.6	7.8	33.6
Final ΔE (PC/PW)	2.4	8.6	4.4	10.0	34.7	0.0	-0.9
Intermediate R (PW)	4	197	17	178	—	14	—
Final R (PC/PW)	4	83	20	112	—	1	—
Cycle	1	2	1	3	post-an.	2	post-an.
Δa (%)	2.4	9.6	2.9	5.9	1.6	5.3	1.4
Δb (%)	-1.6	-2.5	3.9	2.5	-3.6	1.5	0.4
Δc (%)	-1.9	-4.2	-3.8	0.0	-2.0	-4.5	1.3
$\Delta\alpha$ (°)			4.1				-1.2
$\Delta\beta$ (°)	-1.3	-3.5	2.5	4.8	3.4	1.6	-0.2
$\Delta\gamma$ (°)			-1.2				-0.2
ΔV (%)	-0.4	2.7	2.3	4.2	-5.0	1.1	2.5
ΔX (Å)	0.16	0.12	0.09	0.06	0.48	0.42	0.06
$\Delta\tau$ (°)	3.2	5.1	4.6	3.0	17.5	8.1	5.1
$\Delta\omega$ (°)	0.6	13.8	21.1	22.4	10.6	8.4	13.7

Energy differences ΔE (kJ/mol) and rankings R refer to the energy-minimized experimental structure with respect to the lowest predicted energy. The cycle (see Table 3) in which the structure was found is also indicated. Further entries represent differences (calculated minus observed) in cell parameters, cell volume, and the largest differences in center-of-mass coordinates (ΔX), the orientation of the molecule(s) ($\Delta\tau$), and the dihedral angles (methyl groups in XXV excluded). For XXIV and XXVI these differences were calculated from the structures found in post-analysis.

Compound XXII was found at rank 4. Upon recalculation with the MP/AI method (ab-initio energies and individual multipoles in the XTINKER package [2]), the rank would have improved to $R = 2$ and the energy difference to $\Delta E = 0.2$ kJ/mol.

Compound XXIII. The three polymorphs with $Z' = 1$ were identified, with the important remark that XXIII-d was found at rank 112 which is outside the allowed range of 100 predictions. The rankings of XXIII-a and XXIII-b were also disappointing. In these three structures the geometry was adequately reproduced, with largest deviations in dihedral angles of around 20° for ω_3 and ω_4. The two $Z'' = 2$ structures (not attempted) were optimized in the PC/PW force field, producing ΔE = 10.0 kJ/mol for XXIII-c and 9.0 kJ/mol for XXIII-e. Considering all five polymorphs, there is no relation between the calculated energies and the experimental evidence that either XXIII-a or XXIII-d is the most stable. Similar problems were encountered in the 2000 blind test for compound XXI, where no participant obtained convincing rankings for the three known polymorphs.

Compound XXIV was not found at all. One explanation was found in a serious mistake in the force field: the experimental structure is essentially planar, whereas all calculated structures have the $C(NH_2)_2$ group roughly perpendicular to the plane of the rest of the molecule. Indeed, the assumption (Table 2) of free rotation about ω_4 and ω_3 was based on a sloppy inspection of insufficient data. After correction and structure generation of 20000 structures in $P2_1/c$, the experimental structure was found with $\Delta E \approx 18$ kJ/mol. This is a considerable improvement over the original 35 kJ/mol, but still insufficient: for just this one space group the PC/PW rank already exceeds 200.

Charge transfer from the chlorine ion may be important, but this cannot be modeled in the final force field. Therefore, the complete structure generation was repeated with the two ions combined into a dimer without restrictions on the relative chlorine position. Often the chlorine ion moved some distance away, and the dimers had to be reconstructed by combining the cation with the chlorine ion nearest to the amino group. The experimental structure was found again, now with $R = 70$ and $\Delta E = 19.5$ kJ/mol in the PC/PW force field; the improvement in ranking is due to a charge transfer of about $0.15e$.

Compound XXV was found at rank 1. Only the methyl groups were rotated by 27° and 32° from the experimental positions. It is interesting to note that this successful prediction was obtained despite a typing error in the topology file which set one C–N bond to value of 1.34 Å rather than 1.44 Å. Thus the internal symmetry of the molecule was lost, and the clustering procedure failed to remove many equivalent structures. For instance, a recalculation showed that the first three submitted structures should be, in fact, equivalent.

Compound XXVI was not found at all. This is evidently due to an incomplete generation process, as the energy of the experimental structure is lower than the lowest one in the predictions. A possible explanation is that ΔE for the PW force field in the search is about 34 kJ/mole, corresponding to a ranking over 1000. As remarked above, the PW force field does not describe the internal hydrogen bonds adequately. Had the structure been found, it would have had a very good ranking in the final PC/PW approach.

A fourth cycle of structure generation was done, with emphasis on the four most promising space groups from the previous cycles. This time the experimental structure was found, again with a ranking over 1000. What is required is obviously a better force field for the search or, lacking that, a faster procedure to improve the selection for structures in the final calculation.

It was found that a possible solution for the latter requirement is to calculate energies and charges with the simple STO-3G basis set. These calculations are about 20 times faster than for 6-31G**, while the results are promising: the experimental structure came out as one of the best. This preselection introduces a second set of structures for the final PC/PW calculations. That treatment is still going on, and the final ranking (now $R = 7$, $\Delta E \approx 2$ kJ/mol after 39 structures) is expected to remain below 10. Nevertheless, without knowledge of the experimental structure one should still have to continue that calculation up to at least 1000 structures.

All in all, with more perseverance and this intermediate cycle the experimental structure could have been found by expending roughly 4000 additional CPU hours.

10 Summary and conclusions

The goal of the present study was to investigate the capability of UPACK to find the blind test structures at a reasonably good ranking, in order to allow further calculations with more sophisticated methods like DFT-D. Features and additions that are not readily available to other workers were not used; only an ab-initio routine is needed to calculate point charges and energies. For the four large molecules the structure generation proceeded in cycles with iterative adapting of the search parameters. The procedure was successful for XXII and XXV ($R < 10$), moderately successful for XXIII ($R < 150$), and failed for XXIV and XXVI.

For XXIV an error was made in developing the force field. However, even after correction all force fields used were inadequate. For such structures it appears preferable to construct neutral dimers rather than treat the ions as separate “molecules”.

For XXVI the lesson was learned that longer structure generation, followed by an intermediate STO-3G calculation, would probably have been successful.
