# Supplementary material for: Report on the sixth blind test of organic crystal structure prediction methods
Source: Acta Crystallogr B Struct Sci Cryst Eng Mater. 2016 Aug 1;72(Pt 4):439–59. doi: 10.1107/S2052520616007447 (PMC4971545; doi:10.1107/S2052520616007447)
Supplement: Supplementary file 3 [file b-72-00439-sup3.zip › Group_06_Elking_FustiMolnar.pdf]

Supporting Information for the Sixth Blind Test of Organic Crystal-Structure Prediction Methods

Dennis Elking (PI) denny@eyesopen.com
Laszlo Fusti-Molnar (PI) laszlo@eyesopen.com

The crystal structures in the accompanying .cif files were produced by geometry optimization using both force fields and partial re-optimization with plane wave density functional theory (dft). The crystal structures are ranked based on potential energy. A brief methodological description of both the force field and plane wave dft calculations are provided below.

Force Field Description

Initial starting crystal structures are randomly generated, and then optimized using an atomic multipole based force field. The force field inter-molecular energy is composed of an electrostatic contribution and a vdW contribution. The electrostatic contribution is calculated with atomic quadrupoles derived from the Distributed Multipole Analysis[1] (DMA) method with the GAMESS[2] quantum chemistry code. The vdW contribution is calculated from a damped 8-6 Lennard Jones potential of the following form

$$U_{vdW}(r) = \frac{A_1 A_2}{r^8}\exp(-0.5r^2) - \frac{C_1 C_2}{r^6}$$

The C_i dispersion parameters are taken from Grimmes[3], while the A_i repulsion parameters are fit to reproduce experimental crystal structures after rigid body geometry optimization. The Ewald method is used to calculate both the long range electrostatic and dispersion energies. The intra-molecular energy is calculated with the Merck Molecular Force Field[4] (MMFF).

For flexible molecules, initial conformations are generated in gas phase. Separate multipole sets are calculated for each conformation. The top 32 space groups as populated by a statistical analysis of the Cambridge Structure Database[5] are considered. For molecule XXII, two geometries with identical energies are used as starting structures followed by rigid body crystal optimization. For molecules XXIII, XXIV, XXV, and XXVI, several gas phase initial conformations are used as starting structures followed by flexible molecule crystal optimization. For each molecule, the crystals are ranked on total inter-molecular + intra-molecular energy. For each molecule, the top 50 structures from the initial crystal prediction run, as well as additional supplementary structures, are used as input for plane wave dft calculations described as described below.

After an initial complete crystal prediction run, atomic multipoles for the top 2000 crystal structures are re-calculated for molecules XXIII, XXIV, XXV, and XXVI. The crystal structures with re-calculated multipoles are re-optimized using flexible molecule local optimization and re-ranked based on force field total energy.

Plane Wave Density Functional Theory Description

The plane wave density function code Quantum Espresso[6] (QE) was used to refine the initial guess force field structures. Projected Augmented Wave (PAW) method, PBE functional, XDM dispersion corrections, K=2 sampling and 80 Rydberg energy cutoff were used in all calculation. The final force field list described above with the re-calculated multipoles and re-ordered final structures were not available when the QM refinements were started. Approximately one month of wall time were used for all QM refinements using Amazon AWS with Starcluster environment. We provide additional structures that were used as starting point of the qm calculations labeled as "qminputs" in order to be able to analyze the positive or negative impact of qm refinements starting from those force field structures. Some additional limitations had to be applied in quantum calculations due to large computational expenses. The majority of crystal optimizations were not converged tight enough and were stopped due to time limitations. Only the most frequent space groups were

used and with additional restriction to Z<=2 for XXV and XXVI and Z<=4 for XXII, XXIII, XXIV. The total CPU cost will be analyzed and published later.

References

1. A.J.Stone, M.Alderton Molecular Physics 56, 5 (1985)
2. M.W.Schmidt, K.K.Baldridge, J.A.Boatz, S.T.Elbert, M.S.Gordon, J.H.Jensen, S.Koseki, N.Matsunaga, K.A.Nguyen, S.J.Su, T.L.Windus, M.Dupuis, J.A.Montgomery J. Comput. Chem. 14, 1347-1363 (1993)
3. S. Grimme. J. Comp. Chem. 27, 1787 (2006).
4. T. A. Halgren, J. Comp. Chem. 17, 490-519 (1996)
5. F. H. Allen, Acta Cryst. B58, 380-388 (2002)
6. P. Giannozzi, S. Baroni, N. Bonini, M. Calandra, R. Car, C. Cavazzoni, D. Ceresoli, G. L. Chiarotti, M. Cococcioni, I. Dabo, A. Dal Corso, S. Fabris, G. Fratesi, S. de Gironcoli, R. Gebauer, U. Gerstmann, C. Gougoussis, A. Kokalj, M. Lazzeri, L. Martin-Samos, N. Marzari, F. Mauri, R. Mazzarello, S. Paolini, A. Pasquarello, L. Paulatto, C. Sbraccia, S. Scandolo, G. Sclauzero, A. P. Seitsonen, A. Smogunov, P. Umari, R. M. Wentzcovitch, J.Phys.:Condens.Matter, 21, 395502 (2009)
