# Supplementary material for: Report on the sixth blind test of organic crystal structure prediction methods
Source: Acta Crystallogr B Struct Sci Cryst Eng Mater. 2016 Aug 1;72(Pt 4):439–59. doi: 10.1107/S2052520616007447 (PMC4971545; doi:10.1107/S2052520616007447)
Supplement: Supplementary file 3 [file b-72-00439-sup3.zip › Group_07_Ende_Cuppen_compressed.pdf]

Methodology for the Van den Ende group

Daniël T. de Jong, Joost A. van den Ende, René de Gelder, Niek J. J. de Klerk, Rita Bylsma, Gilles A. de Wijs, Hugo Meekes, and Herma M. Cuppen *

Radboud University, Institute for Molecules and Materials, Heyendaalseweg 135, 6525 AJ Nijmegen, The Netherlands. E-mail: h.cuppen@science.ru.nl

Our primary goal for the participation in the sixth blind test of organic crystal structure prediction (CSP) methods was to focus on the ranking of possible structures rather than on the structure generation. Our forte is understanding the impact of anisotropic interactions on crystal growth and we aimed at including this aspect in the ranking rather than basing it purely on thermodynamic energies. A limitation of these energies is the unavoidable self energy component which is a complication when comparing the results of crystal structures obtained with different stoichiometries of the constituting molecules and also when comparing to experimental sublimation enthalpies. To surpass this limitation, we have exploited a newly developed method which calculates lattice energies as a sum of intermolecular interaction energies on the basis of the electron density within the crystal structure itself: q-GRID(de Klerk *et al.*, 2015).

Another approach to rank the generated crystal structures is to take the kinetic pathway of formation of the crystal into account. Information about this kinetic pathway can be obtained from interactions between pairs of molecules. These interactions are

extracted from the generated crystal structures and form the input of a Kinetic Monte Carlo procedure(Boerrigter *et al.*, 2004; Deij *et al.*, 2007) that predicts which crystal structures are the most likely to form.

During the past year, we have experienced the challenging aspects of CSP in its first step: structure generation. Due to our limited experience and the time constraints corresponding to the test, we are unfortunately not able to perform the final analysis (q-GRID and Kinetic Monte Carlo procedure) at the level that we initially aimed and we have not included targets XXIV and XXVI in our submission, because of issues with charge that our q-GRID method cannot treat at the moment (target XXIV) and issues of size and flexibility (target XXVI).

1. Procedure

The isolated molecular structures were optimized with Density Functional Theory using Molpro(Werner *et al.*, 2012) in a two-step fashion: first at a B3LYP/6-31+G(d) level and secondly at a B3LYP/6-311+G(d,p) level of theory. The resulting output file was subsequently used to calculate the electrostatic potential (ESP) on the Van-der-Waals surfaces, to generate ESP atomic point charges using Molden (Schaftenaar & Noordik, 2000).

Crystal structures were generated randomly by an adapted version of Upack(van Eijck & Kroon, 1999). The adaptations are outlined in Section 3. For each compound 5000 structures are created with $Z' = 1$ for each of the following nineteen space groups: C2, C2/c, C2/m, Cc, P1, P$\bar{1}$, P2_1, P2_12_12, P$2_12_12_1$, P2_1/c, P2_1/m, P2/c, Pbca, Pbcn, Pc, Pca2_1, Pccn, Pna2_1, and Pnma. Additionally, for target XXV we created 9999 $Z' = 2$ structures in each of the five space groups P2_1/c, P1, P$\bar{1}$, P$2_12_12_1$, and P2_1. All generated crystal structures were then optimized in four steps with respect to their lattice energies

determined by different force fields, first using a simple Lennard Jones force field, which is present in UPACK, followed by the more accurate Generalized Amber Force Field (GAFF) (Wang *et al.*, 2004; Cornell *et al.*, 1995) with a cut-off 25 Å. For each force field, the molecules were flexible throughout the optimization procedure. First a steepest descent algorithm was applied with as convergence criterion an energy change of less than 0.01 kJ/mol and secondly a conjugate gradients algorithm which was considered to be converged when the root mean square value of the energy derivatives was less than 0.0005 kJ/mol. The conjugate gradients optimization was followed by a clustering step as implemented in UPACK.

The resulting structures from UPACK were then imported into PLATON(Spek, 2009) to obtain their actual space group and to generate more conventional CIF files. Finally, a clustering procedure was applied on the basis of computed powder X-ray diffraction patterns using the program ISOQUEST (de Gelder, 2006) which is based on cross-correlation functions (de Gelder *et al.*, 2001). The 2θ range was set to 30.00 degrees and the width weighting, l, was set to 2.00 for all target structures. The initial list of unique structures was then ranked according to lattice energy. This list forms the basis of our primary ranking and serves as input for the two final ranking approaches mentioned in the introduction. A detailed flow chart of the procedures used is shown in Fig.1.

1.1. q-GRID ranking

The structures with the strongest binding lattice energies were re-ranked by a better description of their charge distribution within the crystalline environment. For this reranking we used the newly developed method q-GRID(de Klerk *et al.*, 2015). In q-GRID the electron density within the crystalline environment is partitioned over grid points which are assigned to the different molecules in the crystal. The electron density within

the crystalline environment is calculated using DFT-D2(Grimme, 2006) geometry optimizations with VASP(Kresse & Hafner, 1993), using the PBE-functional. Intermolecular interaction energies of pairs of molecules are then calculated as a Coulomb sum between grid points and nuclei and analytic dispersion and repulsion contributions are added to this. Lattice energies can be obtained by summation over the interaction energies. For more details concerning the method we refer to de Klerk *et al.*(2015). With the help of Mercury(Macrae *et al.*, 2008) we compared several structures before and after the optimization and this lead to the conclusion that this procedure is indeed a form of re-ranking rather than an adaptation of the crystal structure, since the overlap between the crystal structures was 15 out of 15 for standard settings of the crystal packing similarity tool. The number of structures on the top of the original list taken for this procedure, differed per target and will be mentioned in the corresponding sections.

1.2. Monty ranking

As mentioned in the introduction, a different perspective on the CSP challenge is the study of the kinetic pathway of formation of hypothetical structures. In other words, which structures are expected to show up in a crystallisation essay? To obtain an alternative ranking with this perspective in mind, we performed kinetic Monte Carlo (kMC) calculations on our primary list of structures in order to determine critical cluster sizes for nucleation as a function of driving force. The program used for this procedure is Monty(Boerrigter *et al.*, 2004; Deij *et al.*, 2007) which has recently been adapted to incorporate more general criteria to determine critical cluster sizes. The input for the kMC simulations is formed by a crystal graph which describes the interactions between pairs of molecules in the proposed crystal structure up to a certain cut-off. In principle, these graphs could have been obtained with q-GRID which we expect to give a better

description of the intermolecular interactions than force-fields. However, due to time constraints we have generated the graphs from the applied force field in the crystal structure generation step by the usage of the Hartman-Perdok module in Cerius(Cerius, 1997).

Different crystal structures experience different driving forces in the same crystallisation condition, because of their different chemical potentials, as is illustrated in Fig. 1 of the paper by Deij *et al.* (2007). To compare structures within the same crystallisation condition, a shift of the driving force corresponding to the difference in chemical potentials can be applied, which for example can be approximated by taking the difference of the heat of dissolution. Since this is an experimental property, we cannot take the same approach in this CSP test and we have chosen the difference in lattice energies as calculated by the obtained crystal graphs as the applied shift. However, since we are working with classical force fields this approximation is quite crude and moreover the experimental driving force is unknown.

We have chosen to rank only the first 10 structures of the re-ranked list on the basis of the lowest critical nucleus size in a shifted critical nucleus size vs. driving force plot and the rest of the structures on the basis of an unshifted critical nucleus size vs. driving force plot. For this unshifted part of the list we combined the rankings at $\Delta\mu/kT = 1$ (R_1) and $\Delta\mu/kT = 3$ (R_3), which are driving force values at which reasonable critical nucleus sizes occur. The combined list contains a re-ranking of $2R_1$ and $2R_3$+1. For each structure in this list, appearing twice, the best scoring ranking is maintained. In this way, there is a slight and arbitrary preference for the calculations at $\Delta\mu/kT = 1$. Finally, original structures from which the graph could not successfully be calculated are added to the bottom part (high rankings) of the list.

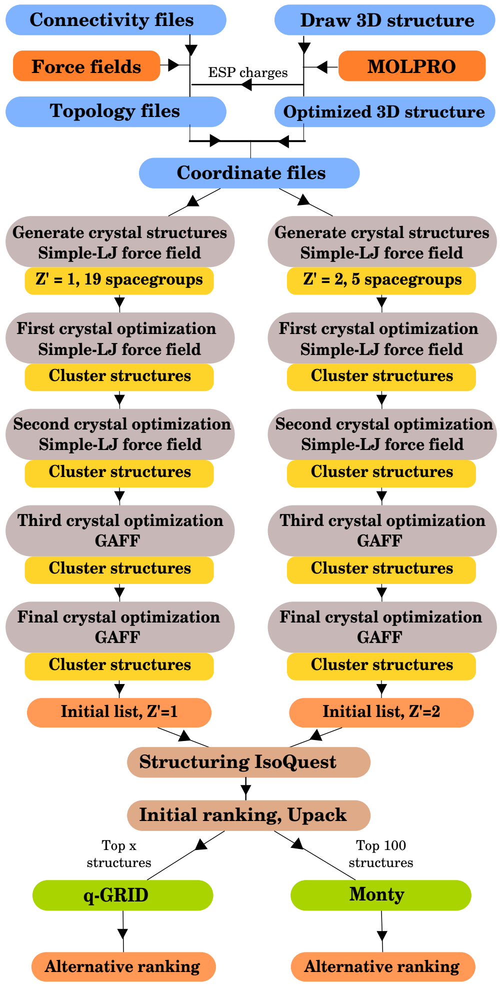

Fig. 1. Flowchart of the used procedures. After several optimizations in a classical force field, structures were re-ranked through a detailed analysis of the electron density within the generated crystal structures (q-GRID) or kinetic Monte Carlo calculations (Monty) on the pathway of formation of these structures.

2. Results

2.1. Target XXII

The GAFF parameters were found to be not completely suitable to accurately describe the five-membered ring of this compound, which resulted in a large self energy. We therefore manually changed the equilibrium angles within this ring to those obtained by

the *ab initio* optimized molecule. The six-membered ring forms a 'tent'-like structure, the angle of which was set to be variable by setting the torsional energy terms concerning this 'tent' to zero. The similarity limit for the IsoQuest procedure was set to 97%. The primary top 100 structures are found within an energy range of 8 kJ/mol. The top 25 of this primary list was reranked by the q-GRID method. Fig. 2 shows the result of this reranking. Evidently, there is not a clear correlation between the two rankings which makes the choice for the top 25 merely a choice governed by feasibility limitations and time constraints instead of by chemical considerations concerning accuracy. The difference in absolute values of the energies between the two methods, is the self-energy of the molecule. This is incorporated in the UPACK-energy, but naturally avoided in the q-GRID energy which therefore calculates the lattice energy in a direct way.

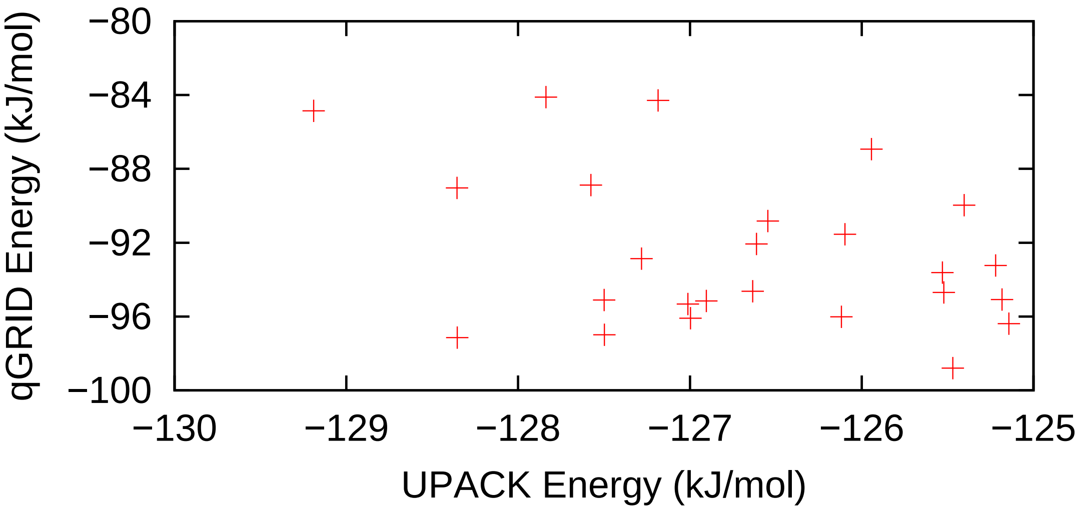

Fig. 2. q-GRID reranking results for the energies of the top 25 structures of target XXII. There is no clear correlation between the UPACK and the q-GRID energies

2.2. Target XXIII

We have chosen to limit ourselves to the $Z' = 1$ structures for this target molecule. Three rotatable bonds were identified (blue bonds in Fig. 3). For each rotatable bond, the corresponding dihedral was randomly assigned and its force constant is set to zero, such that more flexibility is introduced. Structures generated in the space groups Pnma and C2/m were constantly rejected. We have therefore chosen to remove these two space groups for the CSP of this target structure. The similarity limit for the IsoQuest procedure was set to 95%. The primary top 100 structures are found within an energy range of 25 kJ/mol. From the top 25 of this list we have performed a q-GRID analysis for 10 of the structures. We have decided to put these 10 structures ranked on their q-GRID energy on the top of list, which is merely an arbitrary choice.

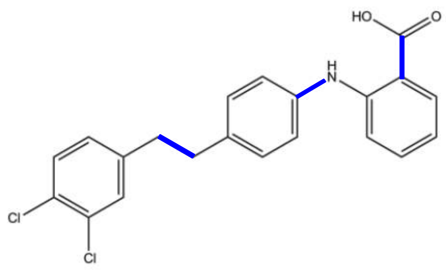

Fig. 3. Target XXIII. The three blue colored bonds indicate that the corresponding dihedral angles were randomly assigned. The compound can rotate about these bonds.

2.3. Target XXV

In the crystal structure generation stage, we have encountered many failed attempts when both independent molecules of the co-crystal were randomly placed in the crystal structure, because symmetry operations may cause the molecules to overlap which results in unreasonable structures which are then rejected. We therefore defined a non-existent bond between the two molecules to keep the two molecules together during the generation

stage. During optimization, this bond was removed again and the molecules were found to move with respect to each other in some cases. In this way, the structure generation is more efficient, but we obviously might miss plausible structures. We manually generated three different asymmetric units for the generation, as can be seen in Fig. 4. The complete procedure was performed in GAFF and three rotatable bonds were defined (blue bonds in the figure). For this compound, $Z' > 1$ structures could also exist. The similarity limit for the IsoQuest procedure was set to 99%. The primary top 100 structures were found within an energy range of 15 kJ/mol. $Z' = 1$ structures with space group $P\bar{1}$ were found most in the primary top 100, which is in agreement with similar structures from the CSD. The top 100 contains only $Z' = 1$ structures.

Because the ratio of the molecules in the crystal is unknown, we also modeled 3000 $Z' = 1$ structures with a 1:2 ratio in the same nineteen space groups as before, starting from two different asymmetric units, shown in Fig 5. The similarity limit for the IsoQuest procedure was set to 99%. The primary top 100 structures are found within an energy range of 20 kJ/mol with higher energies than structures with a 1:1 ratio. In this case, also P$\bar{1}$ structures are found most in the top 100.

Because the asymmetric unit for 1:2 structures has a different self energy than the 1:1 structures, the energies cannot be straightforwardly compared. However, within the q-GRID methods this comparison is possible because it determines the lattice energy while naturally avoiding the calculation of the self energy. Unfortunately, due to time constraints we were not able to perform q-GRID calculations on Target XXV.

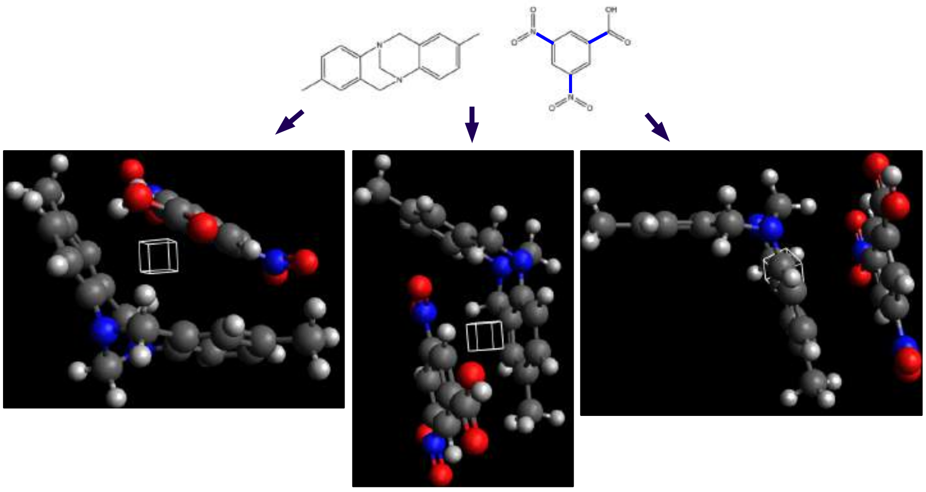

Fig. 4. Target XXV. The three blue colored bonds indicate that the corresponding dihedral angles were randomly assigned. Three different asymmetric units were constructed for the generation stage.

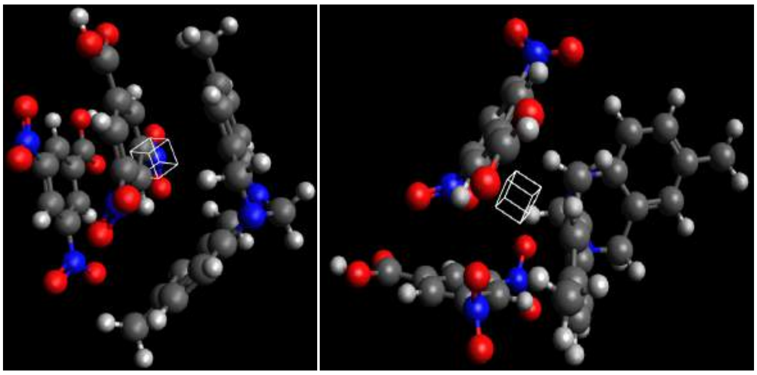

Fig. 5. Target XXV. Two constructed asymmetric units (1:2 ratio) for the generation stage.

2.4. Computing times

Tab. 1 shows the cpu-hours spent for the different parts of the calculation. The hardware used are computer clusters with either Intel™ Xeon™ or AMD Opteron™ processors and with clock speeds ranging from 2.2 to 2.6 GHz. Please note that the compu-

tational times spent on q-GRID and Monty could significantly decrease when technical difficulties connected to the early status of the development of these methods are resolved.

Table 1. *The CPU time used in hours for the CSP.*

	XXII	XXIII	XXV
Ab initio	12	50	38
UPACK	330	3825	5750
VASP	8381	3098	-
q-GRID	418	204	-
MONTY	600	600	600
Total	9741	7777	6388

3. Upack adjustments

In version 10 (2012) of UPACK(van Eijck & Kroon, 1999) the use of pair lists for non-bonded interactions is essential for stable convergence. These lists are only updated when a significant change of any geometric parameter is observed. In our adjusted version, this pair list is changed upon every step of the energy minimizations (steepest descent and conjugate gradients). With this conservative approach, the risk at diverging optimizations is smaller. For the generation of coordinate files (i.e. isolated molecules in UPACK) one has to define certain dihedrals. In the original version of UPACK one has to define dihedrals with H-atoms or without H-atoms. Combinations are not possible. We adjusted the program such that these combinations are possible. In this way, coordinate files can be more easily constructed.

Minimizations in the original version of UPACK were continued for structures within an energy window of 30 kJ/mol. In our methodology we continued the minimizations for all structures. In this way, we do not accidentally lose feasible crystal structures.

References

Boerrigter, S. X. M., Josten, G. P. H., van de Streek, J., Hollander, F. F. A., Los, J., Cuppen, H. M., Bennema, P. & Meekes, H. (2004). *J. Phys. Chem. A*, **108**(27), 5894–5902.

Cerius, (1997). *Cerius^2 User Guide.* Accelrys Inc., 9685 Scranton Road, San Diego, CA, USA.

Cornell, W. D., Cieplak, P., Bayly, C. I., Gould, I. R., Merz, K. M., Ferguson, D. M., Spellmeyer, D. C., Fox, T., Caldwell, J. W. & Kollman, P. A. (1995). *J. Am. Chem. Soc.* **117**(19), 5179–5197.

Deij, M. A., ter Horst, J. H., Meekes, H., Jansens, P. & Vlieg, E. (2007). *J. Phys. Chem. B*, **111**(7), 1523–1530.

van Eijck, B. P. & Kroon, J. (1999). *J. Comput. Chem.* **20**(8), 799–812.

de Gelder, R. (2006). *IUCr CompComm Newsletter*, **7**, 59–69.

de Gelder, R., Wehrens, R. & Hageman, J. A. (2001). *J. Comput. Chem.* **22**(3), 273–289.

Grimme, S. (2006). *J. Comput. Chem.* **27**(15), 17871799.

de Klerk, N. J. J., van den Ende, J., Bylsma, R., Grancic, P., de Wijs, G. A., Cuppen, H. M. & Meekes, H. (2015). *Article submitted.*

Kresse, G. & Hafner, J. (1993). *Phys. Rev. B*, **47**(1), 558–561.

Macrae, C. F., Bruno, I. J., Chisholm, J. A., Edgington, P. R., McCabe, P., Pidcock, E., Rodriguez-Monge, L., Taylor, R., van de Streek, J. & Wood, P. A. (2008). *J. Appl. Crystallogr.* **41**(2), 466–470.

Schaftenaar, G. & Noordik, J. (2000). *J Comput. Aid. Mol. Des.* **14**(2), 123–134.

Spek, A. L. (2009). *Acta Cryst. D*, **65**(2), 148–155.

Wang, J., Wolf, R., Caldwell, J., Kollman, P. & Case, D. (2004). *J Comput Chem*, **25**, 1157–1174.

Werner, H.-J., Knowles, P. J., Knizia, G., Manby, F. R. & Schtz, M. (2012). *WIREs Comput. Mol. Science*, **2**(2), 242–253.
