# Supplementary material for: Report on the sixth blind test of organic crystal structure prediction methods
Source: Acta Crystallogr B Struct Sci Cryst Eng Mater. 2016 Aug 1;72(Pt 4):439–59. doi: 10.1107/S2052520616007447 (PMC4971545; doi:10.1107/S2052520616007447)
Supplement: Supplementary file 3 [file b-72-00439-sup3.zip › Group_08_Facelli.pdf]

Crystal Structure Prediction Using the MGAC Approach

Albert M. Lund,[a,b] *Gabriel I. Pagola,*[d] *Anita M. Orendt,*[b] *Marta B. Ferraro,*[d]

and

Julio C. Facelli [b,c,*]

[a] *Department of Chemistry,* [b] *Center for High Performance Computing, and* [c] *Department of Biomedical Informatics, University of Utah, 155 South 1452 East Room 405, Salt Lake City, UT 84112-0190, US.* [d] *Departamento de Física and Ifiba (CONICET) Facultad de Ciencias Exactas y Naturales, Universidad de Buenos Aires, Ciudad Universitaria, Pab. I (1428), Buenos Aires, Argentina.*

***** Corresponding author at julio.facelli@utah.edu

Introduction

We are submitting two set of predictions, the first one, MGAC_CHARMM_QE, uses our previously reported MGAC_CHARMM (Bazterra *et al.*, 2002b, a, Bazterra *et al.*, 2004, Kim *et al.*, 2009) approach followed by re-optimization and re-ranking of the top 110 structures using Quantum Espresso, QE, (Giannozzi *et al.*, 2009), to choose the 100 best structures; we have applied this procedure to Molecules XXII, XXIII, XIV and XXV. A second approach was used for Molecule XII using a modified version of our recently published MGAC2_QE approach (Lund *et al.*, 2015). Details of the two methods are given in the following sections.

MGAC basic information

In the MGAG approach crystal structures are encoded in a *genome* that allows for both the manipulation of the structures by the genetic operators and for the calculation of the energy of the crystal structure. The *genome* is the representation of **Z** molecules, or any arbitrary number of molecules, per unit cell in the crystal (Bazterra *et al.*, 2004). The *genome* for rigid molecules is given by the crystallographic parameters (α, β, γ), the position of the center of mass of each molecule in the cell (r_1, r_2, r_3, …, r_z), and the orientation of the molecular axes with respect to the unit cell (Φ_1, Φ_2, Φ_3, …, Φ_z). For flexible molecules, the *genome* also includes the values of the dihedral angles that can be significantly affected by the intermolecular interactions during the global optimization.

Note that the MGAC program only considers the lattice angles (α, β, γ) as independent parameters whereas the lattice lengths (a, b, c) are dependent parameters in the GA optimization (Bazterra *et al.*, 2007). The lattice lengths are determined from the molecular coordinates in the unit cell. A minimal intermolecular distance, by default 3 Å, is used to minimize the chance of producing very short intermolecular distances between molecules and their neighbors when the initial guesses of lattice lengths are chosen (Bazterra *et al.*, 2007). This parameter will not affect the final structures since all inter- and intra- molecular parameters are locally optimized in every GA generations (Bazterra *et al.*, 2007).

Several GA operators, including the *one-point-crossover, two-point-crossover, n-point-crossover, uniform-crossover, arithmetic-crossover, inversion-crossover, geometric-crossover*, and *gaussian mutation* which have been proposed by Niesse *et al.* (Niesse & Mayne, 1997, White *et al.*, 1998), are implemented in MGAC. The initial generation or population is started

from a set of randomly selected crystal structures, and then the GA operators are used to create a new set of crystal structures for the next generation. At each GA evolution, all the crystal structures are relaxed to their local minima of the potential energy surface using the local optimization routines in CHARMM (MGAC_CHARMM) or QE (MGAC2_QE). This evolution is repeated until either a predefined number of generations is reached or the data convergence process is achieved when the population becomes stagnant.

MGAC can search for solutions in any of the 230 space groups, however for this project the search was restricted to the 14 most common space groups, namely P1, P-1, $P2_1$ 1, C2, P*c*, C*c*, $P2_1/c$, C2/*c*, $P2_12_12_1$, $Pca2_1$, $Pna2_1$, P*bcn*, P*bca*, and P*nma*, (Gdanitz, 1997) to produce a representative sampling of possible packing arrangements. The global parallelization scheme for GA was implemented in MGAC to reach the high sampling power for these searches (Bazterra *et al.*, 2007).

Routines to remove structures that exhibit interatomic distances and or angles not physically possible based on molecular volume and bad atom−atom contacts have been added to MGAC as these unphysical structures typically gave very low energies and sometimes dominated the final populations.

MGAC_CHARMM_QE searches

For all of the crystal structures considered by us in this blind test the preliminary energy calculation and local optimization were performed using CHARMM (Brooks *et al.*, 1983, MacKerell *et al.*, 1998) with the GAFF(Wang *et al.*, 2004) parameters and RESP charges. (Bayly *et al.*, 1993, Cornell *et al.*, 1993) These charges were calculated using the optimized HF/6-31G* molecular geometries obtained from arbitrary local optimizations. But our previous work shows

that do not significantly depend on the molecular conformation. A cutoff of 14 Å was used to compute short range non-bonded interactions and the Ewald technique was then applied to calculate the electrostatic interactions including at least two unit cells in the simulation box in every direction.

The automatic force field generator, *charmmgen*, was implemented based on the *antechamber* program (Wang *et al.*, 2006, Case *et al.*, 2004, Wang *et al.*, 2004). This software package calculates the molecular parameters using GAFF (Case *et al.*, 2004, Wang *et al.*, 2004) which has parameters suitable for most organic and pharmaceutical molecules composed of H, C, N, O, S, P, and halogens. The potential energy function (*U(R)*) is shown below (Case *et al.*, 2004):

$$\begin{aligned} U(R) = & \sum_{bonds} K_r (r - r_{eq})^2 && \textit{bonds} \\ & + \sum_{angles} K_\theta (\theta - \theta_{eq})^2 && \textit{angles} \\ & + \sum_{dihedrals} \frac{V_n}{2} (1 + \cos[n\phi - \gamma]) && \textit{dihedrals} \\ & + \sum_{i<j}^{atoms} \frac{A_{ij}}{R_{ij}^{12}} - \frac{B_{ij}}{R_{ij}^{6}} && \textit{van der Waals} \\ & + \sum_{i<j}^{atoms} \frac{q_i q_j}{\varepsilon R_{ij}} && \textit{electrostatic} \end{aligned}$$

where r_{eq} and θ_{eq} are equilibrium structural parameters. K_r, K_θ, and V_n are force constants, *n* is multiplicity, and γ the phase angle for the torsional angle parameters. In addition, *A*, *B*, and *q* are parameters related to the non-bonded potentials. For the non-bonded part, the electrostatic parameters (q_i, q_j) are calibrated using the restrained electrostatic potential fit (RESP) model (Bayly *et al.*, 1993, Cornell *et al.*, 1993).

A series of three MGAC_CHARMM runs for each of the 14 most common space groups in organic molecules were completed for each of the targets considered here and assuming one molecule per asymmetric unit in all cases. The parameter values describing the initial population are randomly selected, including the values for the dihedral angles included in the global optimization of flexible molecules. Each GA run produced 150 generations with 30 crystal structures each, using a crossover probability of 1.0 and a mutation probability of 0.1. This process generates approximately 20,000. To generate these structures requires running approximately 42 independent computer jobs, each one taking between 0.5 to 24 hrs on 12 or 20 processors; this translates in a total of 210 to 1,100 processor hours per molecule. For example, 643 hrs were used for mol-XXIV and 270 hrs for mol-XXII. After these MGAC_CHARMM runs have been finished, the results were filtered using a series of utilities developed and/or integrated into our analysis environment in order to obtain a set (ranging in size from 300 to 2000 of unique lowest energy structures (Bazterra *et al.*, 2007). These utilities first detect and remove duplicate crystal structures from the final set and then collected the lowest energy structures for further analysis.

To complete the MGAC_CHARM_QE analysis, the best unique 110 structures from this procedure were selected for further optimization and re-ranking using QE (Giannozzi *et al.*, 2009). The optimization parameters for the QE optimization were identical to those used by Lund *et. al*. (Lund *et al.*, 2013). The DFT functional used was the Perdew-Burke-Ernzerhof generalized gradient approximation (Perdew *et al.*, 1996). The dispersion correction method selected was the semi-empirical D2 method proposed by Grimme as implemented in QE (Grimme, 2006). The self-consistency threshold was set to 10^{-7} Ry and the plane wave cutoff

energy was set to 55 Ry per the recommendation of the pseudopotentials authors. The pseudopotentials used were the Rappe-Rabe-Kaxiras-Joannopoulos-Ultrasoft pseudopotentials provided at the QE website, http://www.quantum-espresso.org/ .

Calculations were performed on a LINUX cluster using 16-core or 20-core nodes (2x8-core Intel Xeon E5-2670 processors clocked at 2.60 GHz), with 64 GB memory per node and Mellanox FDR Infiniband for node interconnectivity. The total number of core hours for each system is given in Table 1.

Table 1: Times in core hours for the 110 re-optimized structures using QE

system	QE
mol-XXII	7,600
mol-XXIII	38,500
mol-XXIV	11,500
mol-XXV	39,000

The .cif and energy files submitted are sorted by the final QE optimization energy (first it is the best structure) but the numbering of the structures corresponds to (line data_Crystal_ in the submitted .cif files) the original CHARMM ordering sequence.

MGAC2_QE searches

For the calculations using the MGAC2_QE approach we are using a new version of MGAC under development (MGAC2), which incorporates a number of changes to our approach to the CSP problem. In particular it uses an improved version of the fitcell algorithm, which reduces bias issues in generating the three dimensional structure for a given schema, which led to very positive results in predicting three polymorphs of Glycine. In addition, a different crossing

method, described below, was employed (Lund, 2015) in order to better cover the search space.

Due to time constraints and sheer computational effort required, we selected to perform calculations on mol-XXII only. We searched the 14 most common space groups (with the modification that P2/*c* was used instead of P*c* as this space group has recently replaced P*c* in the list of the 14 most common groups), doing one MGAC run per space group. For each run, an initial population is constructed of 50 valid structures, where a valid structure has a volume +/- 30% of the estimated volume using the substance density and molecular weight. Once 50 valid structures are obtained, those structures plus an additional 50 random structures are crossed using a full crossing, where every structure is crossed with every other structure. Therefore, at each crossing, a total of 10,000 structures are generated. The addition of random structures contributes to the diversity of the population. Mutation of structures was set to a rate of 0.0001. Since full crossing was used, no scaling of structures was applied. For the P*nma* space group, not enough valid structures could be generated in the time constraints to create an initial population. At the end of each generation, the best 50 structures were propagated to the next generation and the process was repeated.

The optimization parameters for the QE optimization were again identical to those used by Lund *et. al*. (Lund *et al.*, 2013). The DFT functional used was the Perdew-Burke-Ernzerhof generalized gradient approximation (Perdew *et al.*, 1996). The dispersion correction method selected was the semi-empirical D2 method proposed by Grimme as implemented in QE (Grimme, 2006). The self-consistency threshold was set to 10^{-7} Ry and the plane wave cutoff energy was set to 55 Ry per the recommendation of the pseudopotentials authors. The pseudopotentials used were the Rappe-Rabe-Kaxiras-Joannopoulos-Ultrasoft pseudopotentials provided at the QE website, http://www.quantum-espresso.org/. MGAC2 runs were performed

on Stampede at the Texas Advanced Computing Center, a LINUX cluster using 16-core nodes (2x8-coreIntel Xeon E5-2680 processors clocked at 2.70 GHz), with 64 GB memory per node and Mellanox FDR Infiniband for node interconnectivity. The final 100 structures were further re-optimized using a single 16 core node (2x8-coreIntel Xeon E5-2670 processors clocked at 2.60 GHz) at the Center for High Performance Computing at the University of Utah.

Each MGAC2 run was limited to a span of 24 hours across 48 nodes, totaling 18,432 core hours per job, or 258,048 core hours for all 14 space groups. During these runs the number of total candidates generated and evaluated by fitcell was 4.36 million. Of these, approximately 6,000 candidate structures were generated that passed the volume filtering step which was used to remove unphysical structures. Each structure was allotted 20 minutes and a number of nodes proportional to the symmetry elements in the space group (i.e., the number of molecules in the unit cell) to complete as many optimization steps as possible. A final population was generated by combining the results of these optimizations. Structures up to the best 600 were evaluated for duplicates by the grouping algorithm contained in the Mercury Crystal Packing Similarity feature. A final optimization of the 100 unique lowest energy structures was performed using the same optimization parameters as before, but allowing the optimizations to come to completion by the QE default convergence parameters. The total cores hours for this step was 2,364, bringing the total core hours to 260,412. A second check for duplicate structures was completed with Mercury, resulting in the submitted set of 77 structures. The numbering of the structures in the final output file provided is numbered according to the ranking after the final QE optimization.

Acknowledgements: Computer resources were provided by the Center for High Performance Computing at the University of Utah and the Extreme Science and Engineering Discovery Environment (XSEDE), supported by NSF grant number ACI-1053575. MBF and GIP acknowledge the support from the University of Buenos Aires and the Argentinean Research Council.

References

Bayly, C. I., Cieplak, P., Cornell, W. & Kollman, P. A. (1993). *J. Phys. Chem.* **97**, 10269-10280.

Bazterra, V. E., Ferraro, M. B. & Facelli, J. C. (2002a). *J. Chem. Phys.* **116**, 5984-5991.

Bazterra, V. E., Ferraro, M. B. & Facelli, J. C. (2002b). *J. Chem. Phys.* **116**, 5992-5995.

Bazterra, V. E., Ferraro, M. B. & Facelli, J. C. (2004). *Int. J. Quantum Chem.* **96**, 312-320.

Bazterra, V. E., Thorley, M., Ferraro, M. B. & Facelli, J. C. (2007). *J. Chem. Theory Comp.* **3**, 201-209.

Brooks, B. R., Bruccoleri, R. E., Olafson, B. D., States, D. J., Swaminathan, S. & Karplus, M. (1983). *J. Comp. Chem.* **4**, 187-217.

Case, D. A., Darden, T. A., Cheatham, T. E., Simmerling, C. L., Wang, J., Duke, R. E., Luo, R., Merz, K. M., Wang, B., Pearlman, D. A., Crowley, M., Brozell, S., Tsui, V., Gohlke, H., Mongan, J., Hornak, V., Cui, G., Beroza, P., Schafmeister, C., Caldwell, J. W., Ross, W. S. & Kollman, P. A. (2004).

Cornell, W. D., Cieplak, P., Bayly, C. I. & Kollman, P. A. (1993). *J. Am. Chem. Soc.* **115**, 9620-9631.

Gdanitz, R. J. (1997). *Theoretical Aspects and Computer Modeling of the Molecular Solid State*, edited by A. Gavezzotti, p. 185. USA: John Wiley and Sons.

Giannozzi, P., Baroni, S., Bonini, N., Calandra, M., Car, R., Cavazzoni, C., Ceresoli, D., Chiarotti, G. L., Cococcioni, M., Dabo, I., Corso, A. D., Gironcoli, S. d., Fabris, S., Fratesi, G., Gebauer, R., Gerstmann, U., Gougoussis, C., Kokalj, A., Lazzeri, M., Martin-Samos, L., Marzari, N., Mauri, F., Mazzarello, R., Paolini, S., Pasquarello, A., Paulatto, L., Sbraccia, C., Scandolo, S., Sclauzero, G., Seitsonen, A. P., Smogunov, A., Umari, P.

& Wentzcovitc, R. M. (2009). *Journal of Physics: Condensed Matter* **21**, 395502-395519.

Grimme, S. (2006). *J. Comput. Chem.* **27**, 1787-1799.

International Tables for Crystallography, 2007). International Union of Crystallography.

Kim, S., Orendt, A. M., Ferraro, M. B. & Facelli, J. C. (2009). *J. Comp. Chem.* **30**, 1973-1985.

Lund, A. M. (2015). thesis, University of Utah, Salt Lake City.

Lund, A. M., Orendt, A. M., Pagola, G. I., Ferraro, M. B. & Facelli, J. C. (2013). *Crystal Growth & Design* **13**, 2181-2189.

Lund, A. M., Pagola, G. I., Orendt, A. M., Ferraro, M. B. & Facelli, J. C. (2015). *Chemical Physics Letters* **626**, 20-24.

MacKerell, A. D., Brooks, J., B. , Brooks III, C. L., Nilsson, L., Roux, B., Won, Y. & Karplus, M. (1998). *The Encyclopedia of Computational Chemistry*, edited by P. v. R. S. e. al., pp. 271-277. Chichester: John Wiley & Sons.

Niesse, J. A. & Mayne, H. R. (1997). *J.Comput. Chem.* **18**, 1233.

Perdew, J. P., Ernzerhof, M. & Burke, K. (1996). *The Journal of Chemical Physics* **105**, 9982-9985.

Wang, J., Wang, W., Kollman, P. A. & Case, D. A. (2006). *Journal of Molecular Graphics and Modelling* **25**, 247-260.

Wang, J., Wolf, R. M., Caldwell, J. W., Kollman, P. A. & Case, D. A. (2004). *J. Comput. Chem.* **25**, 1157.

White, R. P., Niesse, J. A. & Mayne, H. R. (1998). *Journal of Chemical Physics* **108**, 2208-2218.
