# Supplementary material for: Report on the sixth blind test of organic crystal structure prediction methods
Source: Acta Crystallogr B Struct Sci Cryst Eng Mater. 2016 Aug 1;72(Pt 4):439–59. doi: 10.1107/S2052520616007447 (PMC4971545; doi:10.1107/S2052520616007447)
Supplement: Supplementary file 3 [file b-72-00439-sup3.zip › Group_09_Goto_Obata_Post_Analysis.pdf]

Supporting Information

Shigeaki Obata[1] and Hitoshi Goto[1,2]

[1]Educational Programs on Advanced Simulation Engineering, Toyohashi University of Technology, 1-1 Hibarigaoka, Tempaku-cho, Toyohashi, Aichi 441-8580, Japan

[2]Department of Computer Science and Engineering, Graduate School of Engineering, Toyohashi University of Technology, 1-1 Hibarigaoka, Tempaku-cho, Toyohashi, Aichi 441-8580, Japan

E-mail: obata@adsim.tut.ac.jp, gotoh@tut.jp

Introduction

We tried to predict crystal structures of three targets: XXII, XXIII, and XXV (Figure 1). Calculations for searching crystal structures were performed by using our original method [1-4] implemented in a molecular mechanics package CONFLEX [5]. In order to rank the predicted crystal structures based on their crystal energies evaluated by our calculation model of the finite-sized spherical crystal and the MMFF94s potential [6] implemented to CONFLEX, some of the favorable crystal structures found were re-estimated by CASTEP in Materials Studio 8.0 [7] using the GGA-PBE exchange-correlation functional, Tkatchenko-Scheffler scheme [8] for evaluating dispersion interactions, and ultrasoft pseudopotentials.

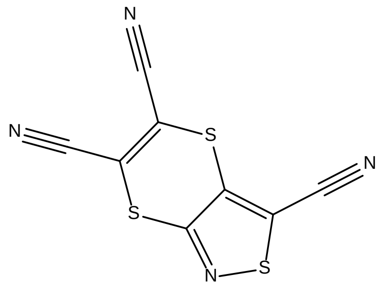

XXII

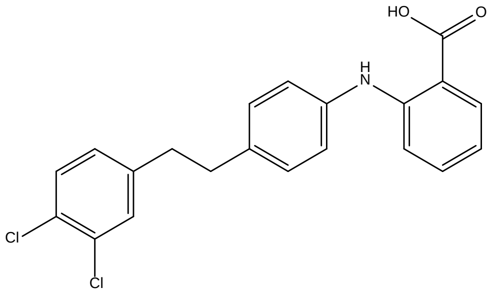

XXIII

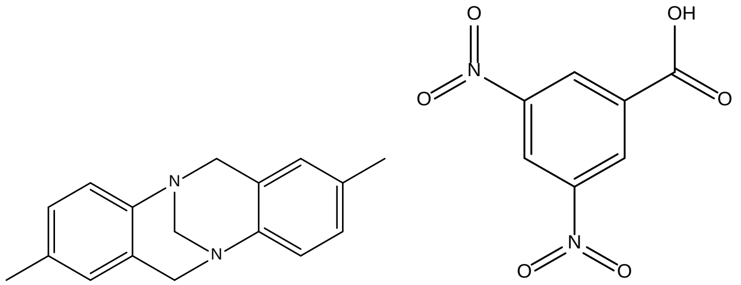

XXV

Figure 1. Targets for blind test.

Overview of Prediction Methods

Our crystal structure prediction employs a typical grid-systematic search algorithm, and four variables required for packing molecular crystal (molecular conformation, molecular orientation, molecular spatial position, and symmetry) were considered. In this work, the prediction procedure have been divided into four main stages: (a) determination of initial molecular geometry and conformations, (b) systematic generation of trial crystal structures, (c) optimization of trial crystal structures, and (d) estimation of predicted crystal structures.

(a) Determination of initial molecular geometry and conformations

In order to generate initial geometries from the chemical structure formulae (Figure 1), we used a molecular design supporting package ChemBioOffice 2012 [9]. The initial geometries were subjected to geometry optimization and conformational space search [10, 11] in gas phase by means of CONFLEX [5]. In the case of XXV, we also explored stable configurations and orientations of two molecules for determining initial arrangements by stepwise rotating each molecule by rotational step of 45 degrees.

(b) Systematic generation of trial crystal structures

In order to consider a number of possible molecular packing arrangements on a specified space group symmetry, each initial conformation of target molecules was rotated around *x*, *y*, and *z* axes. Trial crystal structures were constructed systematically by using the molecules having each unique orientation and by applying symmetry operations of various specified space groups. We have used fourteen space groups of $P1$, $P\overline{1}$, $P2_1$, $C2$, Pc, Cc, $P2_1/c$, $C2/c$, $P2_12_12_1$, $Pca2_1$, $Pna2_1$, $Pbcn$, $Pbca$, and $Pnma$ in this work. Initial lattice lengths were determined by a size of the target molecule and a type of space group, and initial lattice angles are equal to 90 degrees.

(c) Optimization of trial crystal structures

In our crystal calculation model, we construct a finite-sized spherical crystal structure with a given effective crystal radius R_{crystal}. Crystal energy (E_{crystal}) of the spherical crystal model was calculated by the summation of the intramolecular interaction energies in an asymmetric unit, the intermolecular interaction energies between molecules in the asymmetric unit (that is obviously zero if only one molecule in the asymmetric unit), and the interatomic interaction energies between the molecule(s) in the asymmetric unit and replica molecules generated by symmetry operations. Both intra- and inter-molecular interactions were estimated by means of MMFF94s potential [6] implemented in CONFLEX [5]. Precise description of the finite-sized spherical crystal calculation model is explained in our recent publication [1].

All trial crystal structures were subjected to two crystal structure optimizations under a specified space group symmetry. First, lattice parameters of unit cell and spatial positions of molecules in the spherical crystal were optimized while each molecular geometry and orientation were unchanged. In the case of XXV, that is, there are two molecules (Tröger's base and 3,5-dinitrobenzoic acid) in the asymmetric unit, lattice parameters of unit cell and molecular orientations and spatial positions of Tröger's bases in the spherical crystal were optimized, and molecular geometries and molecular orientations and spatial positions of 3,5-dinitrobenzoic acid molecules were unchanged. Second, lattice parameters of unit cell and atomic positions of molecules in the spherical crystal were optimized. Therefore, all degrees of freedom for representing the crystal structure under the specified space group symmetry were fully

optimized and, furthermore, the optimized crystal structures were confirmed whether they have no imaginary frequencies by performing a normal mode analysis in the atomic coordinates under the specified space group symmetry. The all optimizations were carried out using a conjugate gradient technique.

(d) Estimation of predicted crystal structures

After the optimizations, adequate space groups and lattice parameters of the predicted crystal structures were determined by using PLATON program [12]. The unique predicted crystal structures within about 3.5 kcal/mol of the global energy minimum in E_{crystal} based on the MMFF94s potential were re-estimated by CASTEP in Materials Studio 8.0 [7] using the GGA-PBE exchange-correlation functional, Tkatchenko-Scheffler scheme [8] for evaluating dispersion interactions, and the ultrasoft pseudopotential under the periodic boundary condition within the space group symmetry. In the PBE-TS calculations, we determined only crystal energies of the MMFF94s optimized structures for ranking the predicted crystal structures based on their energies on the calculation level.

Molecule XXII

As the result of conformational space search, we found two conformations of XXII (Figure 2). One is a bended conformation of an energy minimum (Figure 2 (a)). Another one is a planar conformation (Figure 2 (b)) that is a transition state between the bended conformers. In the generation of trial crystal structures, each conformation was rotated by the rotational step of 20 degrees around *x*, *y*, and *z* axis, and the trial crystal structures were constructed by employing the conformations having each unique orientation and fourteen space group symmetries. Total number of trial crystal structures are 291,600. All trials were subjected to crystal structure optimization based on the MMFF94s potential. The 200 unique structures within 3.684 kcal/mol of the global energy minimum in E_{crystal} based on the classical force field were re-estimated by DFT-based PBE-TS calculations and ranked again by their PBE-TS crystal energies. Table 1 and Table S1 summaries top 10 and top 200 predicted crystal structures of molecule XXII based on the PBE-TS calculations, respectively.

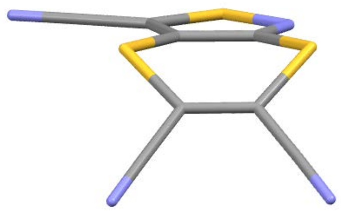

(a)

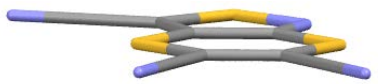

(b)

Figure 2. Conformations of XXII based on the MMFF94s potential. (a) Bended conformation (energy minimum) (b) Planar conformation (transition state).

Table 1. Top 10 predicted crystal structures of molecule XXII.

Rank	$\Delta E_{\mathrm{PBE\text{-}TS}}$ /kcal·mol^{-1}	Space Group	Density /g·cm^{-3}	*a* /Å	*b* /Å	*c* /Å	*β* /°
**1**	**0.000**	$\boldsymbol{P2_1/c}$	**1.499**	**11.771**	**7.258**	**12.926**	**94.94**
**2**	**0.002**	$\boldsymbol{P2_1/c}$	**1.505**	**12.681**	**6.597**	**15.840**	**124.21**
**3**	**0.305**	$\boldsymbol{Pna2_1}$	**1.527**	**13.337**	**10.532**	**7.689**	**90.00**
4	0.334	$P2_1/c$	1.507	10.415	6.538	16.103	93.25
5	0.364	$Pna2_1$	1.506	12.355	12.753	6.949	90.00
6	0.757	$P2_12_12_1$	1.503	6.986	12.308	12.763	90.00
7	0.902	$Pna2_1$	1.483	8.903	19.939	6.268	90.00
8	1.057	$P2_12_12_1$	1.493	6.571	8.055	20.878	90.00
9	1.071	$P2_1/c$	1.533	5.963	9.224	19.878	100.27
10	1.145	$P2_1/c$	1.530	8.643	7.856	16.231	101.98

We suggests top 3 predicted crystal structures (Table 1) as the most likely crystal structures of XXII (Figure 3). The 1st predicted crystal structure, which is the lowest energy crystal structure, shows the interactions between carbon and nitrogen atoms in cyano groups (Figure 3 (a)). On the other hand, the 2nd predicted crystal structure has the interactions between cyano group and 2,3-dihydro-1,4-dithiine (Figure 3 (b)). The 3rd predicted crystal structure has the interactions between sulfur atoms and between cyano nitrogen and sulfur atoms (Figure 3 (c)). Energy difference between the 1st and 2nd structures is only 0.002 kcal/mol (Table 1) on the PBE-TS calculations, and crystal structure difference is small. Molecules in those crystals are characterized by A, B, C, and D: A is identity, B is related to A with inversion symmetry, C is related to A with glide plane symmetry, and D is related to A with 2-fold screw axis symmetry (Figure 4). If anticlockwise molecular rotations of A and B and clockwise molecular rotations of the C and D around the *a* axis occurs in the 2nd structure, the 2nd structure seems to be translate into the 1st one.

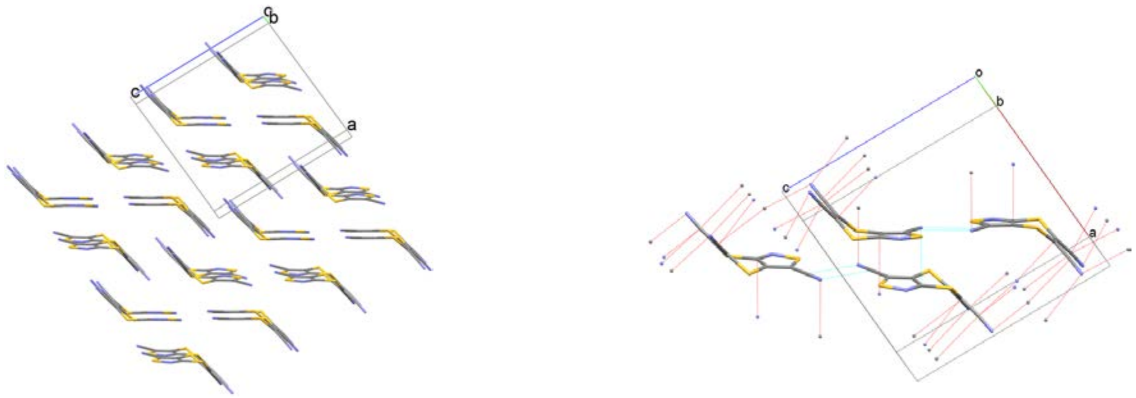

(a) 1st

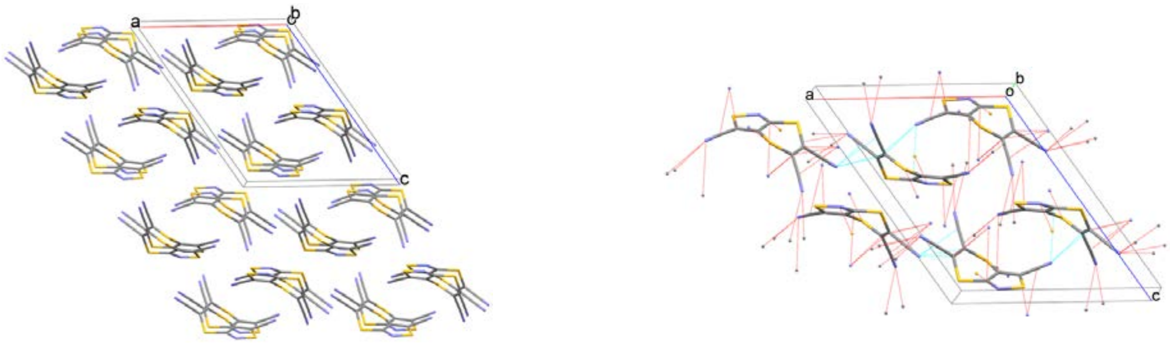

(b) 2nd

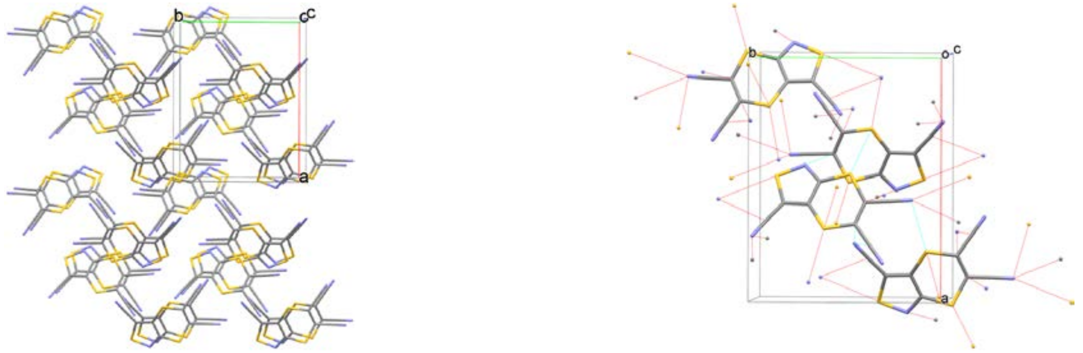

(c) 3rd

Figure 3. Top 3 predicted crystal structures of molecule XXII. Red and light blue dotted lines in right drawings show shorter contacts than the sum of the vdW radius plus 0.2 Å created by using the Materials Module of Mercury [13].

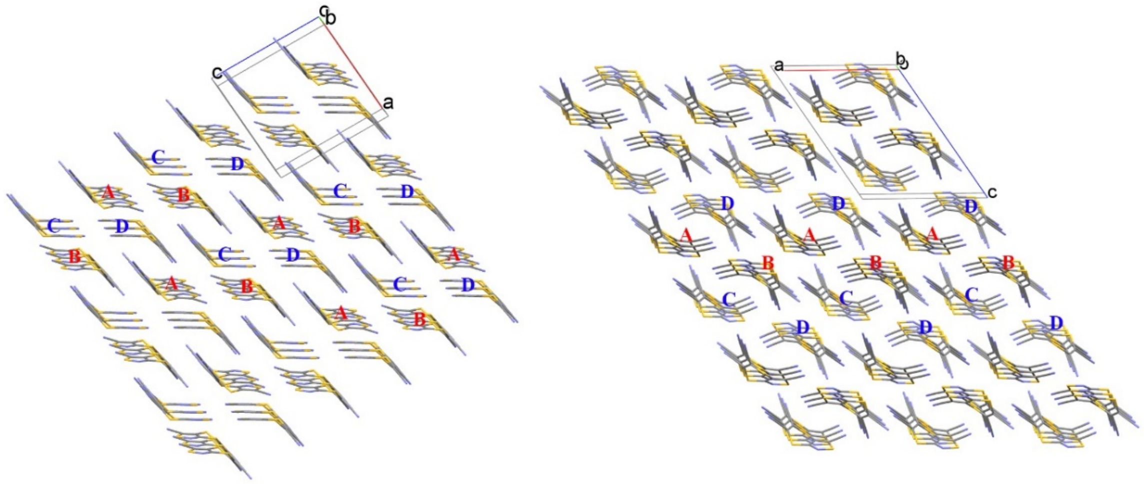

Figure 4. Comparison of the 1st and the 2nd predicted crystal structures. Both structures have the $P2_1/c$ symmetry. Therefore, if the molecules denoted by A are identities, B molecules are related to A molecules with inversion symmetry, C molecules are related to A molecules with glide plane symmetry, and D molecules are related to A molecules with 2-fold screw axis symmetry.

Molecule XXIII

As the result of conformational space search, we found 114 conformers of XXIII, and 31 conformers within 7.5 kcal/mol of the global energy minimum (Figure S1) were employed as the initial geometries for generating trial crystal structures. In order to explore unique crystal structures, each conformer was rotated around *x*, *y*, and *z* axis by the rotational steps of 30 degrees for the three common space groups of $P\bar{1}$, $P2_1/c$, and $C2/c$ and 60 degrees for eleven space groups. 423,708 trial crystal structures generated were subjected to crystal structure optimization. Finally, the 240 unique structures were found under 3.2 kcal/mol of the global energy minimum in E_{crystal} based on the MMFF94s potential, and they were re-estimated by the PBE-TS calculations. Ranking of favorable crystal structures in the crystal energies of PBE-TS calculations were shown in Table 2 (top 10) and also Table S2 (200 lower energy crystal structures).

Table 2. Top 10 predicted crystal structures of molecule XXIII

Rank	$\Delta E_{\text{PBE-TS}}$ /kcal·mol^{-1}	Space Group	Density /g·cm^{-3}	a /Å	b /Å	c /Å	α /°	β /°	γ /°
**1**	**0.000**	$\boldsymbol{P2_1/c}$	**1.306**	**4.632**	**24.672**	**17.210**	**90.00**	**93.08**	**90.00**
**2**	**0.163**	$\boldsymbol{P2_1/c}$	**1.310**	**7.823**	**27.637**	**9.811**	**90.00**	**112.54**	**90.00**
**3**	**0.199**	$\boldsymbol{P2_1/c}$	**1.289**	**4.569**	**15.658**	**27.822**	**90.00**	**90.42**	**90.00**
**4**	**0.244**	$\boldsymbol{P\bar{1}}$	**1.291**	**4.652**	**12.324**	**17.697**	**78.42**	**88.05**	**88.85**
**5**	**0.290**	$\boldsymbol{P\bar{1}}$	**1.311**	**7.820**	**9.729**	**14.285**	**78.45**	**80.45**	**67.46**

6	0.794	$P\bar{1}$	1.297	6.867	9.261	16.147	85.90	80.37	77.82
7	1.126	$P\bar{1}$	1.283	8.044	12.087	12.154	61.26	88.41	75.94
8	1.162	$P2_1/c$	1.290	4.624	29.714	14.495	90.00	92.59	90.00
9	1.234	$P2_1/c$	1.281	7.894	34.846	7.579	90.00	106.05	90.00
10	1.236	$P\bar{1}$	1.269	7.250	10.208	14.668	83.62	76.20	73.80

We suggests top 5 predicted crystal structures as the most likely polymorphs of XXIII (Figure 5), since the pre-information of sixth blind test showed that XXIII has three polymorphs of *Z'*=1 (The other two known polymorphs have *Z'*=2). These top 5 structures are built by the OH···O hydrogen bonding interactions of carboxylic acid dimers (Figure 5). Conformations in the 1st, 3rd, and 4th predicted crystal structures are similar to the 9th conformer in gas phase (Figure 6 (a)). On the other hand, conformations in the 2nd and 5th predicted crystal structures are similar to the 8th conformer in gas phase (Figure 6 (b)). Conformational changes between gas and crystalline phases have been observed (Figure 6 and Table 3). The maximum difference is 21 degrees of the torsional angle of C4-C5-C7-C8 in the 2nd predicted crystal structure that can determine the direction of the dichlorophenyl group (Figure 6 (b) and Table 3).

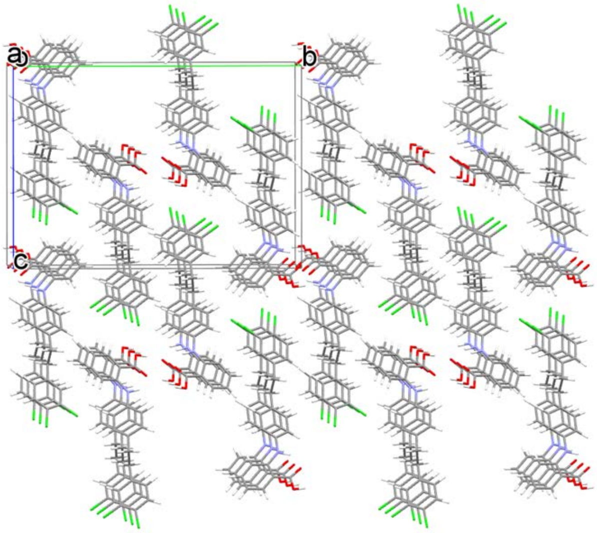

(a) 1st

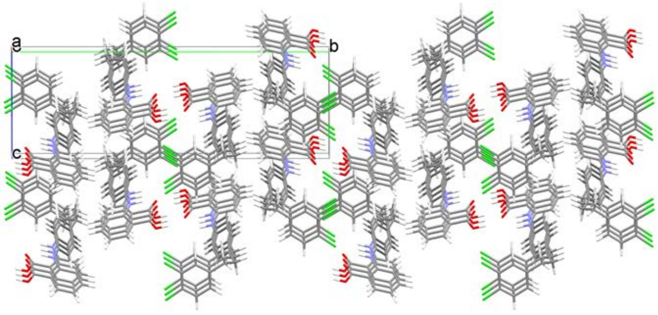

(b) 2nd

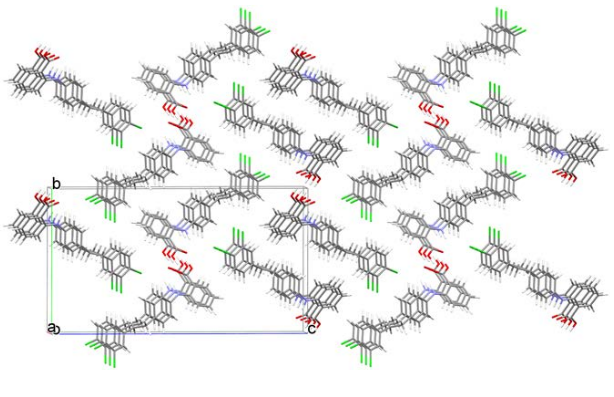

(c) 3rd

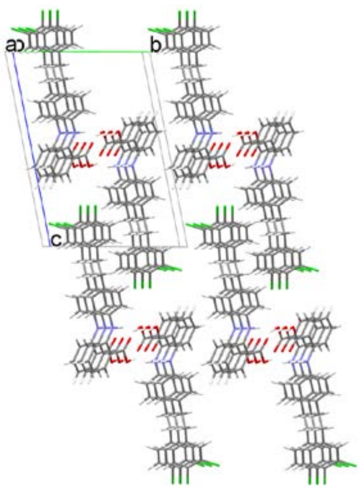

(d) 4th

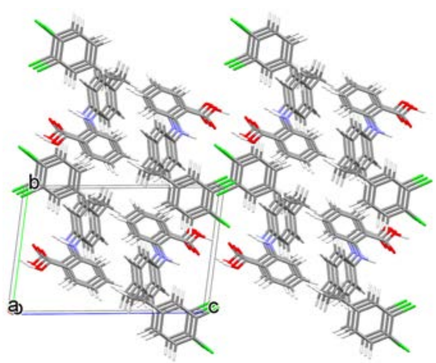

(e) 5th

Figure 5. Top 5 predicted crystal structures of molecule XXIII.

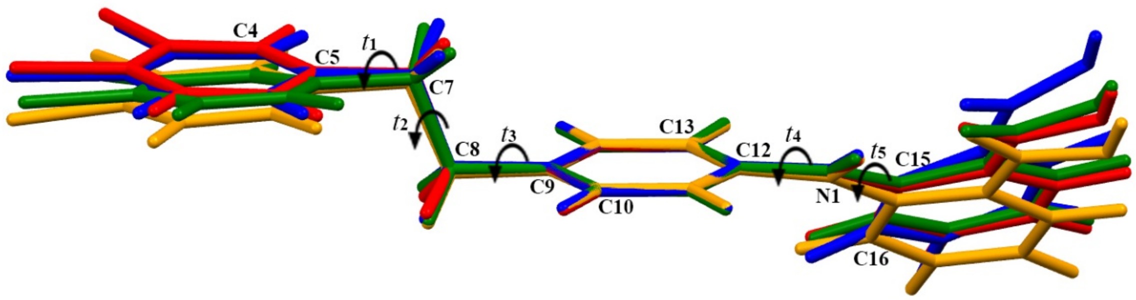

(a)

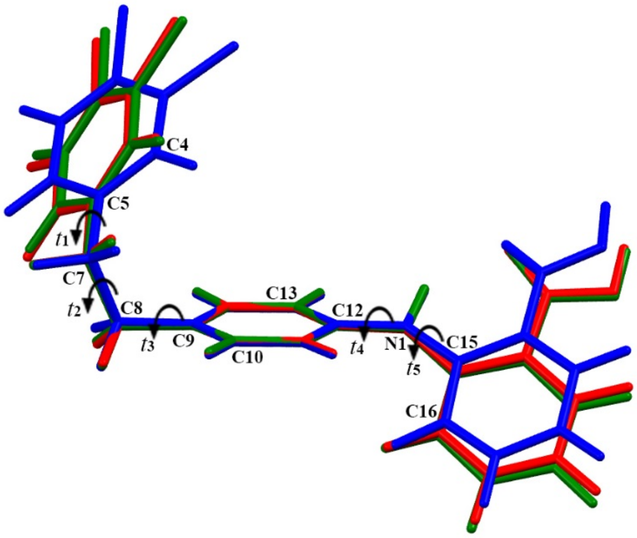

(b)

Figure 6. Characteristic conformations in the predicted crystal structures of XXIII. (a) Superposition of the 9th conformer in the gas phase (blue) and the conformations in the 1st (red), 3rd (green), and 4th (orange) predicted crystal structures. (b) Superposition of the 8th conformer in gas phase (blue) and, the conformations in the 2nd (red) and 5th (green) predicted crystal structures.

Table 3. Torsional angles of conformers in gas phase and conformations in the predicted crystal structures of XXIII.

Structure	Torsional angle [a] / degrees				
	t_1: C4-C5-C7-C8	t_2: C5-C7-C8-C9	t_3: C7-C8-C9-C10	t_4: C13-C12-N1-C15	t_5: C12-N1-C15-C16
CID-9 [b]	-88.82	179.95	89.30	-24.19	-23.44
XXIII_R001	-101.03	-174.35	97.36	-23.97	-14.10
XXIII_R003	-84.47	-167.39	87.98	-23.50	-15.82
XXIII_R004	-94.30	-173.18	92.70	-30.52	-9.54
CID-8 [b]	-95.90	70.44	78.32	-154.24	23.79
XXIII_R002	-117.18	71.54	91.34	-143.02	11.24
XXIII_R005	-116.41	69.94	88.26	-138.94	8.64

[a] Definitions of torsional angles (atom numbers) are denoted in Figure 6. [b] Conformers found by CONFLEX conformational space search in gas phase are denoted by "CID" numbers and ranked based on their conformational energies (energy difference from the global energy minimum) in the MMFF94s potential. Conformation energies of CID-8 and -9 are 0.468 and 0.886 kcal/mol, respectively.

Molecule XXV

The XXV consists of Tröger's base (TGB) and 3,5-dinitrobenzoic acid (DNBA). Geometry optimizations and conformational space searches of TGB and DNBA in gas phase were performed by using CONFLEX and MMFF94s potential, and the existence of one stable conformation on each molecule was confirmed. Exploring stable configurations and orientations of two molecules were also performed, and then, total 16 unique structures of the molecular complex were determined under 0.5 Å of root mean square difference between the center of mass of DNBAs in complexes superimposed on each other by TGBs. For exhaustive generation of trial crystal structures, DNBAs of each complex structure were rotated by the rotational step of 45 degrees around *x*, *y*, and *z* axis, and the resultant complex structures were applied by fourteen space group symmetries. Finally, total number of trial crystal structures is 166,400. All trials were subjected to crystal structure optimization, and 200 unique predicted crystal structures within 3.48 kcal/mol of the global energy minimum in E_{crystal} based on the MMFF94s potential were re-estimated by the PBE-TS calculations. Top 10 and top 200 predicted crystal structures based on their energies in the PBE-TS calculations were listed in Table 4 and Table S3, respectively.

Table 4. Top 10 predicted crystal structures of XXV complex.

Rank	$\Delta E_{\mathrm{PBE\text{-}TS}}$ /kcal·mol^{-1}	Space Group	Density /g·cm^{-3}	*a* /Å	*b* /Å	*c* /Å	*α* /°	*β* /°	*γ* /°
**1**	**0.000**	***P*1̄**	**1.291**	**6.484**	**13.985**	**14.979**	**112.25**	**97.27**	**103.19**
**2**	**0.097**	***Pbcn***	**1.296**	**27.616**	**10.514**	**16.321**	**90.00**	**90.00**	**90.00**
**3**	**0.269**	***P*1̄**	**1.297**	**6.785**	**11.675**	**16.272**	**105.40**	**93.68**	**105.60**
4	1.621	$P2_1/c$	1.267	16.729	6.773	22.392	90.00	107.12	90.00
5	1.789	$P2_1/c$	1.299	8.388	20.466	13.772	90.00	90.22	90.00
6	1.797	*P*1̄	1.275	6.450	14.070	15.062	112.79	98.27	100.36
7	2.068	*Cc*	1.264	11.578	10.389	20.219	90.00	92.07	90.00
8	2.161	$P2_1/c$	1.301	7.313	19.052	17.276	90.00	101.19	90.00
9	2.243	$P2_1/c$	1.244	16.287	6.863	22.885	90.00	105.09	90.00
10	2.336	$Pna2_1$	1.291	10.211	14.142	16.482	90.00	90.00	90.00

We suggests top 3 predicted crystal structures as the most likely crystal structures of XXV (Figure 7). The 1st and 3rd predicted crystal structures are characterized by alternatively-stacking of DNBA and TGB layers in the direction of (0 0 1), and those layers are stacked by mainly OH···N interactions (Figures 7 (a) and (c)). On the other hand, the 2nd predicted crystal structure show a layer constructed by alternatively-stacking of two DNBAs and two TGBs (Figure 7 (b)).

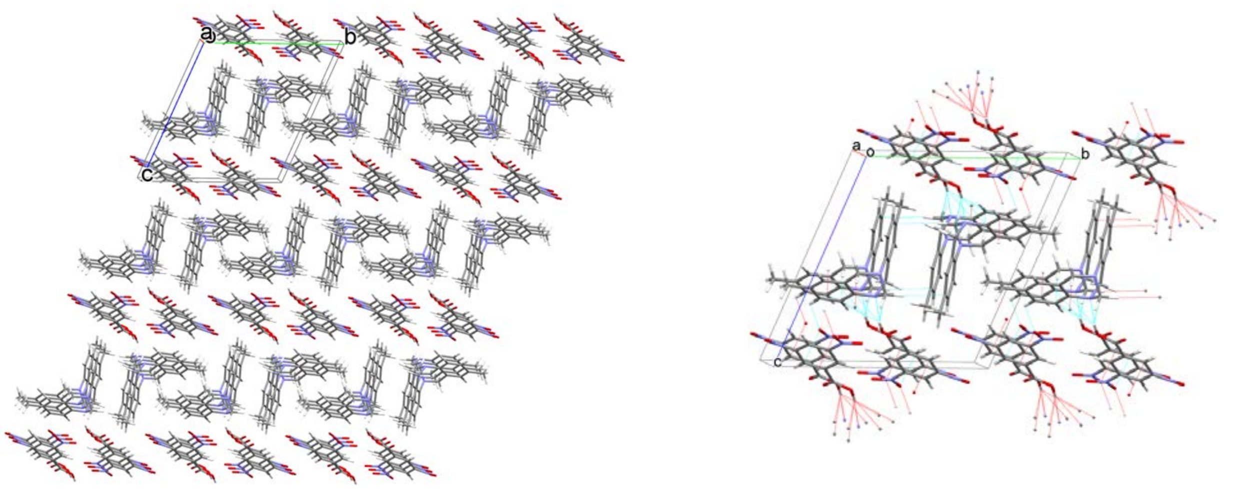

(a) 1st

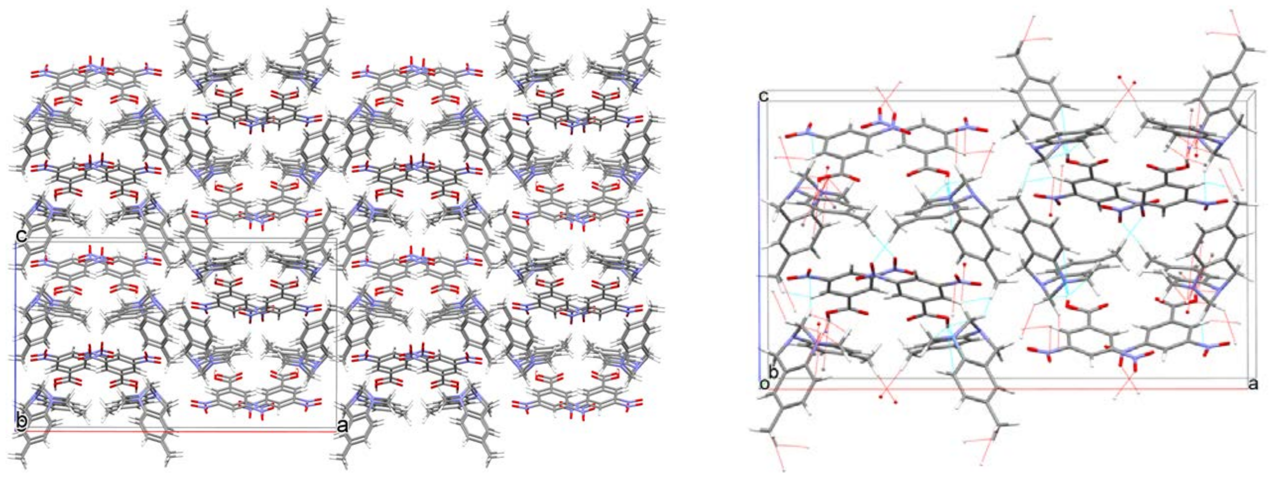

(b) 2nd

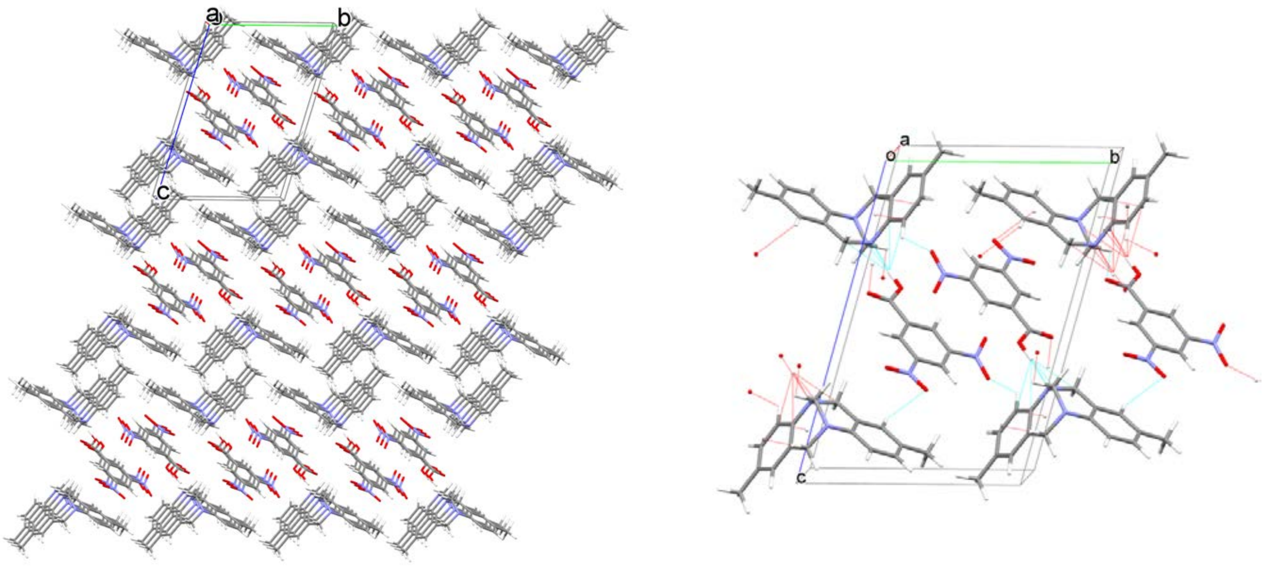

(c) 3rd

Figure 7. Top 3 predicted crystal structures of XXV. Red and light blue dotted lines in right drawings show shorter contacts than the sum of the vdW radius created by using the Materials Module of Mercury [13].

Computational Cost

All calculations were almost performed by using MPI-based parallel machines on TUT supercomputer systems which consists of Intel Xeon CPU E5-2680 2.70 GHz or Intel Xeon CPU E5-2680 v2 2.80 GHz, and partly performed by using Kyoto university supercomputer system (Intel Xeon CPU E5-2670 2.60 GHz) and our laboratory computational clusters (Intel Xeon CPU E5-2690 2.90 GHz). The wall times of the calculations, which were normalized into a computation times of the serial processing corresponding to one core of Intel Xeon CPU 2.7 GHz, were listed as follows. The values in parenthesis show the original computation time.

Molecule XXII:

Crystal Structure Search: 19,000 hours (199 hours)

PBE-TS calculation: 200 hours (53 hours)

Total: CPU hours: 19,200 hours (252 hours)

Molecule XXIII:

Crystal Structure Search: 345,000 hours (4,119 hours)

PBE-TS calculation: 1,000 hours (131 hours)

Total: 346,000 hours (4,250 hours)

Molecule XXV:

Crystal Structure Search: 323,000 hours (4,632 hours)

PBE-TS calculation: 2,000 hours (211 hours)

Total: 325,000 hours (4,843 hours)

Post Analysis

After the submissions of prediction results, information of the observed crystal structures was fully opened to the participants. We have compared our prediction results with them. The preliminary findings are tentatively described below.

Molecule XXIII

In our predicted structure of the ranking 119 (XXIII_R119), we confirmed that the heavy-atom positions are identical with those of the observed structure of XXIII form A, but the position of the hydrogen atom of the carboxylic acid is missed unfortunately.

Molecule XXV

In our predicted structure of the ranking 20 (XXV_R020), we confirmed that the molecular crystal packing is quite similar to the observed one as shown in Figure 8(a), but the DNBA moiety in the asymmetric unit is tilted at about 24 degrees to the observed spatial position in Figure 8(b). Therefore, the XXV_R020 structure does not match the observed one within the criteria of the packing similarity in the blind test.

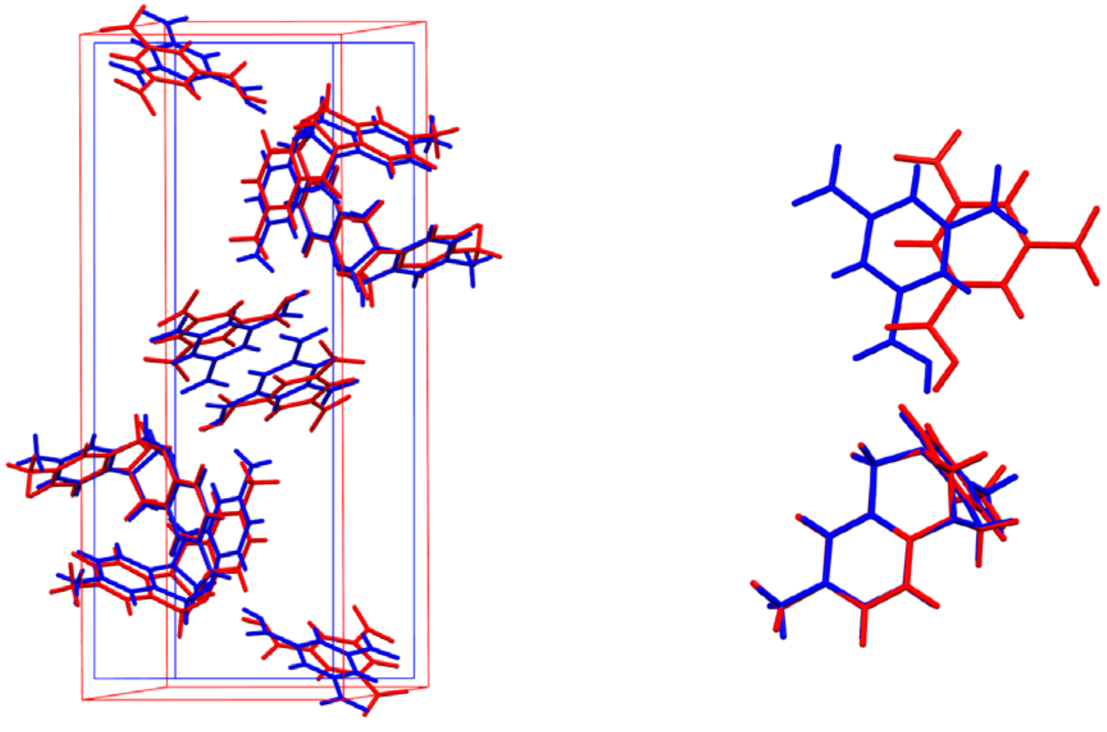

(a) (b)

Figure 8 Superposition of XXV_R020 (red) and the observed structure (blue) is presented by (a) the unit cell and (b) the asymmetric unit. In these figures, the lattice and the asymmetric unit of XXV_R020 were redefined for distinct comparison between them.

Acknowledgements

We thank Dr. Bouke van Eijck for his valuable advice on our predicted structure of XXV. We thank the promotion office for TUT programs on advanced simulation engineering (ADSIM), the leading program for training brain information architects (BRAIN), and the information and media center (IMC) at Toyohashi University of Technology for the use of the TUT supercomputer systems and application software. We also thank the ACCMS at Kyoto University for the use of their supercomputer. In addition, we wish to thank financial supports from Conflex Corp. and Ministry of Education, Culture, Sports, Science and Technology.

References

[1] Obata, S., Goto, H, Manuscript in preparation.

[2] Obata, S., Goto, H. (2008). *J. Comput. Aided Chem.*, **9**, 8-16.

[3] Obata, S., Goto, H. (2008). *J. Comput. Chem. Jpn.*, **7**, 151-164.

[4] Obata, S., Goto, H. (2015). *AIP Conf. Proc.*, **1649**, 130-134.

[5] Goto, H., Obata, S., Nakayama, N., Ohta, K., CONFLEX7, Conflex, Tokyo, Japan, 2012.

[6] Halgren, T. A. (1996), *J. Comput. Chem.*, **17**, 490-519.

[7] Materials Studio 8.0, Dassault Systemes, 2014.

[8] Tkatchenko, A., Scheffler, M. (2009), *Phys. Rev. Lett.*, **102**, 073005.

[9] ChemBioOffice, PerkinElmer, Waltham, MA, USA, 2012.

[10] Goto, H., Osawa, E. (1989), *J. Am. Chem. Soc.*, **111**, 8950-8951.

[11] Goto, H., Osawa, E. (1993), *J. Chem. Soc., Perkin Trans.*, **2**, 187-198.

[12] Spek, A. L. (2009), *Acta Cryst.*, D**65**, 148-155.

[13] Macrae, C. F., Bruno, I. J., Chisholm, J. A., Edgington, P. R., McCabe, P., Pidcock, E., Rodriguez-Monge, L., Taylor, R., van de Streek, J., Wood, P. A. (2008), *J Appl Crystallogr*, **41**, 466-470.

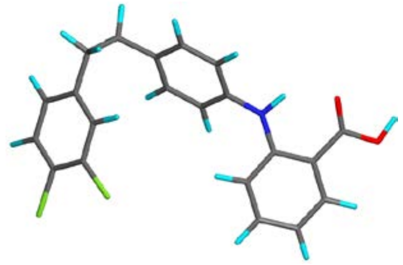

(i)

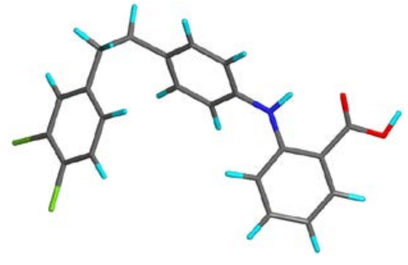

(ii)

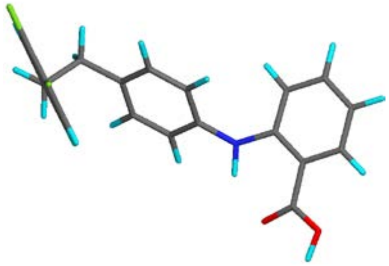

(iii)

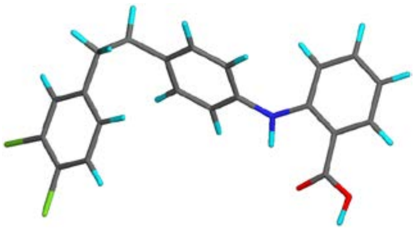

(iv)

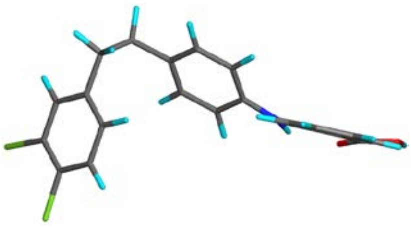

(v)

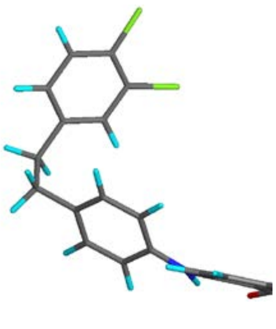

(vi)

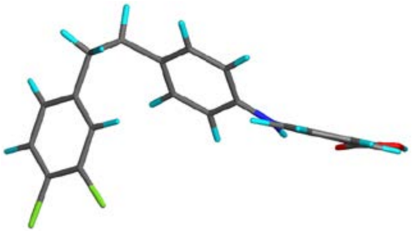

(vii)

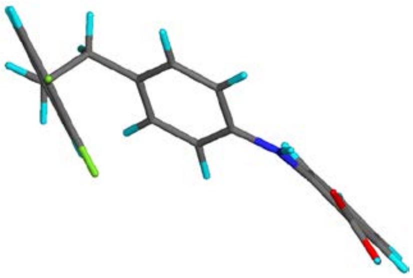

(viii)

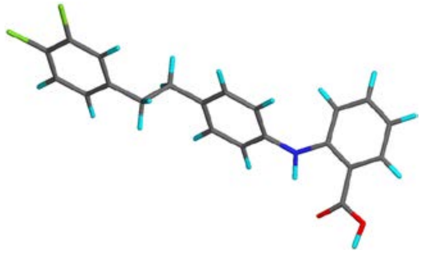

(ix)

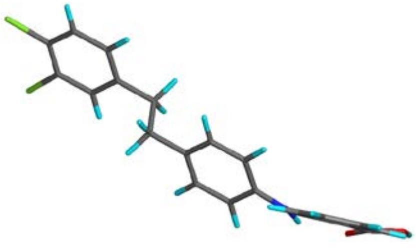

(x)

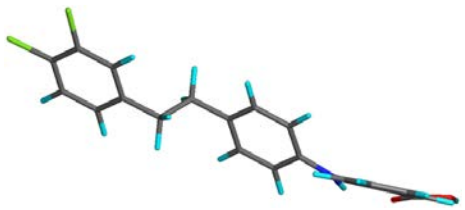

(xi)

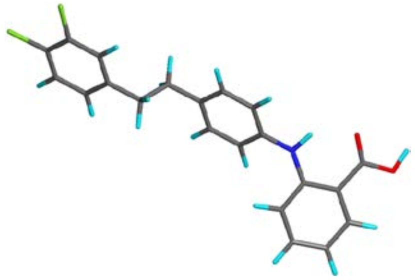

(xii)

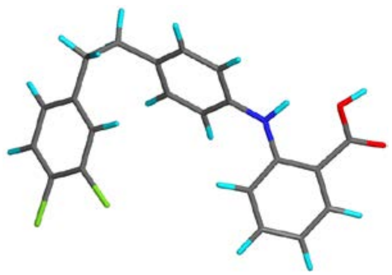

(xiii)

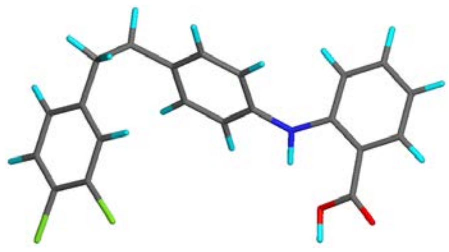

(xiv)

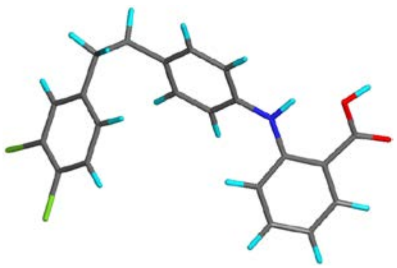

(xv)

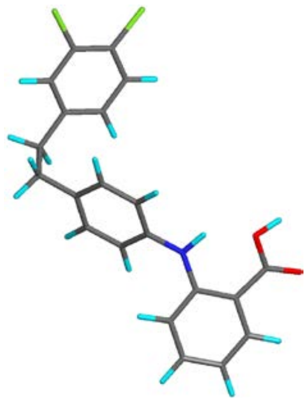

(xvi)

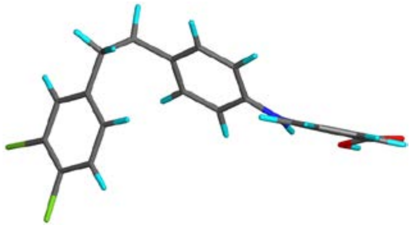

(xvii)

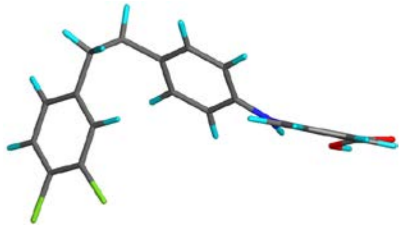

(xviii)

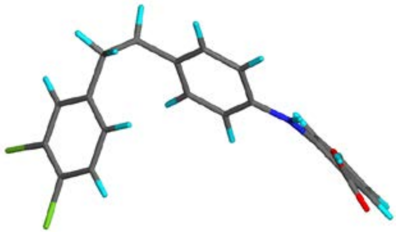

(xix)

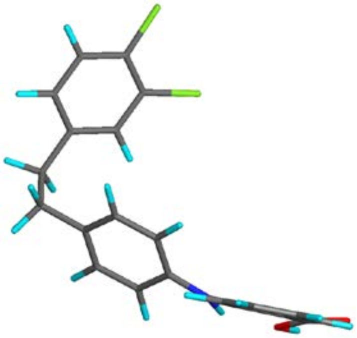

(xx)

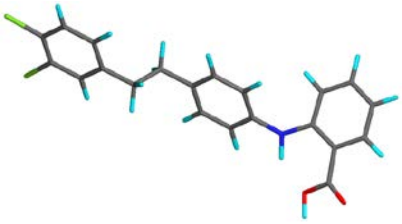

(xxi)

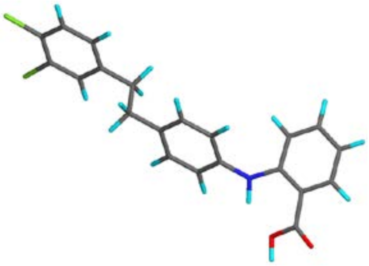

(xxii)

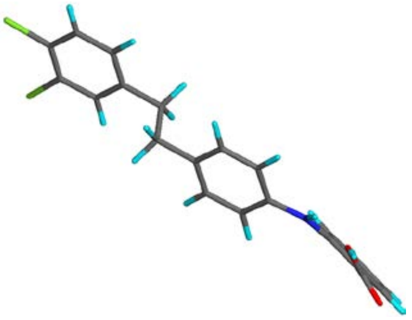

(xxiii)

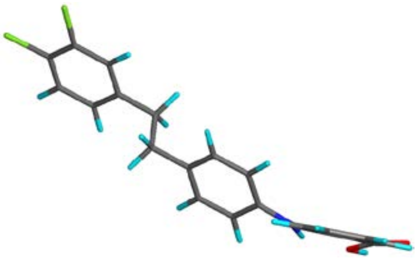

(xxiv)

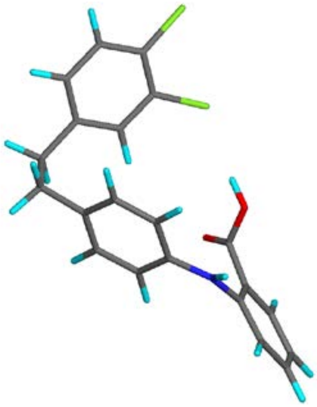

(xxv)

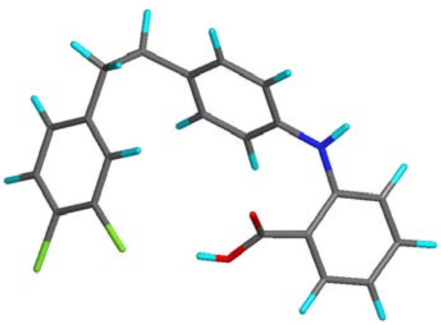

(xxvi)

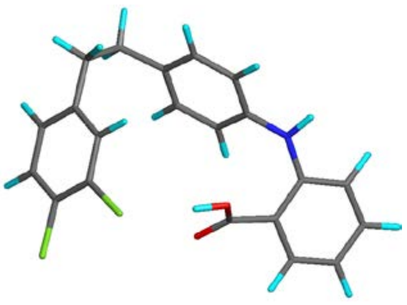

(xxvii)

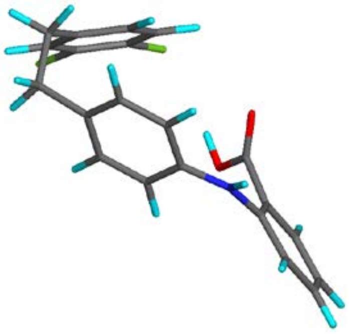

(xxviii)

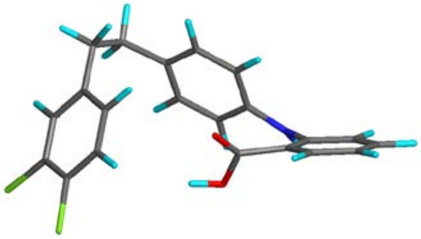

(xxix)

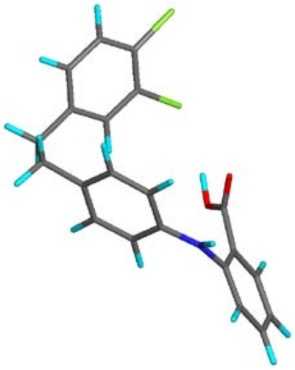

(xxx)

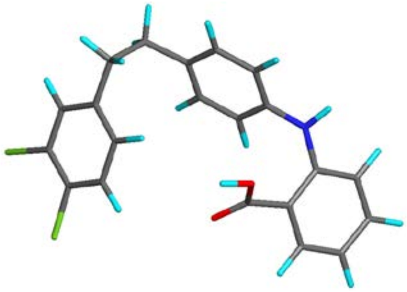

(xxxi)

Figure S1. 31 lowest energy conformers of XXIII in gas phase. The conformers are ordered based on their conformational energies. The conformer (i) show the lowest energy in gas phase.

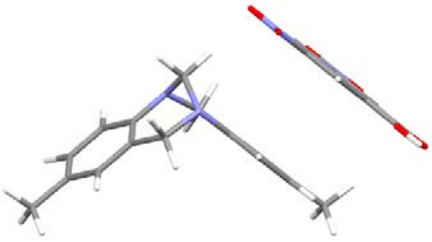

(i)

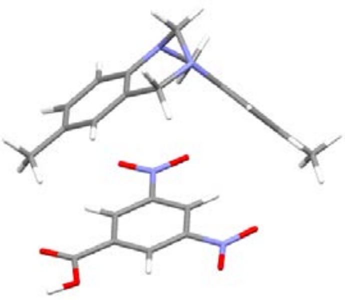

(ii)

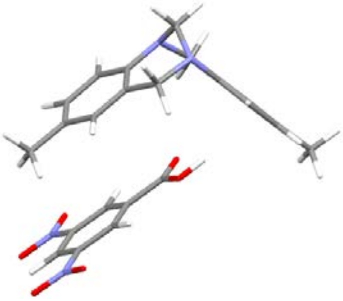

(iii)

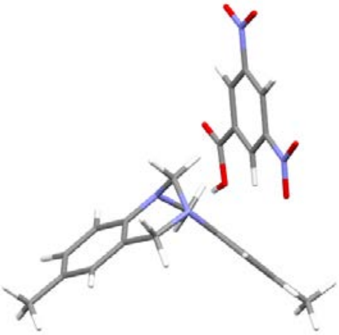

(iv)

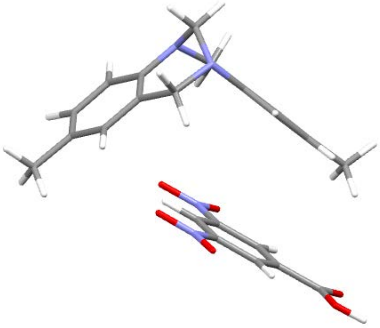

(v)

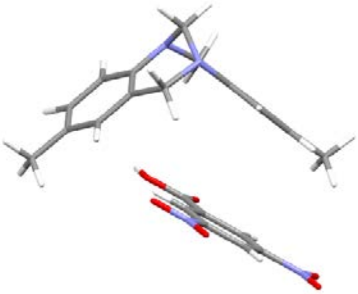

(vi)

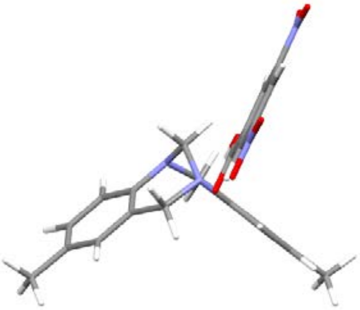

(vii)

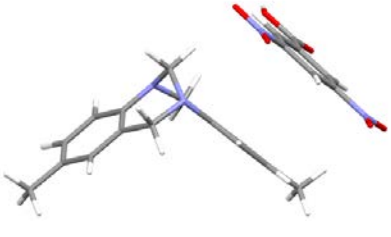

(viii)

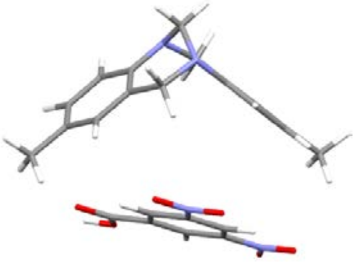

(ix)

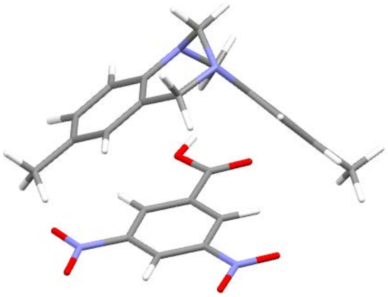

(x)

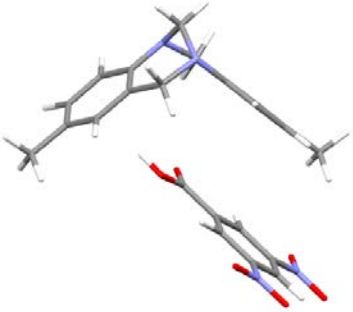

(xi)

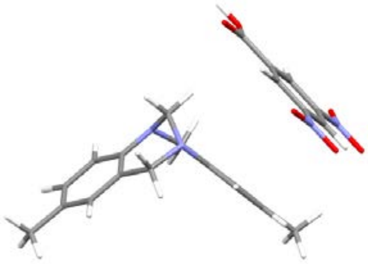

(xii)

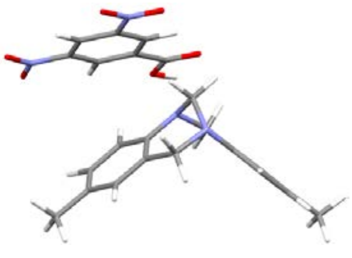

(xiii)

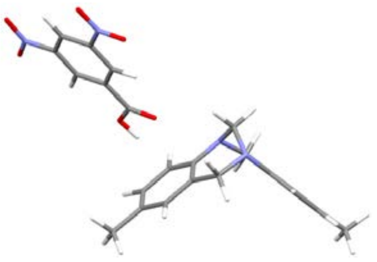

(xiv)

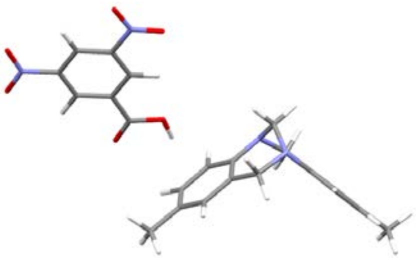

(xv)

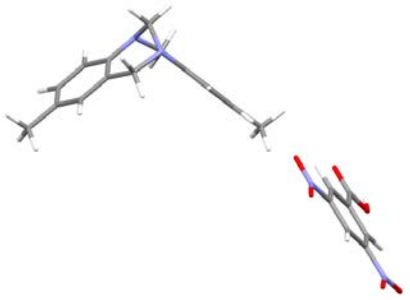

(xvi)

Figure S2. 16 unique structures of the molecular complex XXV. The complex structures are ordered based on their sum of intermolecular interaction energies.

Table S1. List of predicted crystal structures of XXII

Label	Rank	$\Delta E_{\text{PBE-TS}}$ /kcal·mol^{-1}	Space Group	Density /g·cm^{-3}	a /Å	b /Å	c /Å	α /°	β /°	γ /°
XXII_R001	1	0.000	P21/c	1.499	11.771	7.258	12.926	90.00	94.94	90.00
XXII_R002	2	0.002	P21/c	1.505	12.681	6.597	15.840	90.00	124.21	90.00
XXII_R003	3	0.305	Pna21	1.527	13.337	10.532	7.689	90.00	90.00	90.00
XXII_R004	4	0.334	P21/c	1.507	10.415	6.538	16.103	90.00	93.25	90.00
XXII_R005	5	0.364	Pna21	1.506	12.355	12.753	6.949	90.00	90.00	90.00
XXII_R006	6	0.757	P212121	1.503	6.986	12.308	12.763	90.00	90.00	90.00
XXII_R007	7	0.902	Pna21	1.483	8.903	19.939	6.268	90.00	90.00	90.00
XXII_R008	8	1.057	P212121	1.493	6.571	8.055	20.878	90.00	90.00	90.00
XXII_R009	9	1.071	P21/c	1.533	5.963	9.224	19.878	90.00	100.27	90.00
XXII_R010	10	1.145	P21/c	1.530	8.643	7.856	16.231	90.00	101.98	90.00
XXII_R011	11	1.153	P212121	1.501	6.171	9.414	18.913	90.00	90.00	90.00
XXII_R012	12	1.227	Pna21	1.516	20.391	7.582	7.040	90.00	90.00	90.00
XXII_R013	13	1.257	P21/c	1.488	6.515	8.174	21.045	90.00	98.50	90.00
XXII_R014	14	1.258	P21/c	1.465	9.787	7.295	15.834	90.00	95.03	90.00
XXII_R015	15	1.327	P21/c	1.522	10.224	7.504	14.296	90.00	98.76	90.00
XXII_R016	16	1.349	Pbca	1.527	14.215	7.691	19.767	90.00	90.00	90.00
XXII_R017	17	1.374	Pna21	1.496	17.505	9.553	6.595	90.00	90.00	90.00
XXII_R018	18	1.502	P21/c	1.504	8.646	9.327	14.208	90.00	106.79	90.00
XXII_R019	19	1.578	P21	1.502	7.114	7.644	10.251	90.00	99.94	90.00
XXII_R020	20	1.626	C2/c	1.509	19.498	7.883	14.387	90.00	98.73	90.00
XXII_R021	21	1.641	P21	1.479	8.649	6.431	10.084	90.00	96.11	90.00
XXII_R022	22	1.654	P212121	1.466	6.380	8.690	20.290	90.00	90.00	90.00
XXII_R023	23	1.677	Pbca	1.540	12.302	8.861	19.646	90.00	90.00	90.00
XXII_R024	24	1.684	Pca21	1.514	14.127	10.030	7.690	90.00	90.00	90.00
XXII_R025	25	1.700	P212121	1.455	7.987	9.837	14.430	90.00	90.00	90.00
XXII_R026	26	1.703	P21	1.512	7.774	6.518	10.767	90.00	91.03	90.00
XXII_R027	27	1.742	P21/c	1.493	7.087	20.520	10.327	90.00	132.62	90.00
XXII_R028	28	1.797	P21/c	1.447	7.030	22.250	8.711	90.00	123.18	90.00
XXII_R029	29	1.812	P21/c	1.478	6.887	8.355	20.168	90.00	105.95	90.00
XXII_R030	30	1.832	P21	1.494	7.434	6.356	11.688	90.00	90.26	90.00
XXII_R031	31	1.851	P212121	1.482	6.308	7.462	23.650	90.00	90.00	90.00
XXII_R032	32	1.871	P212121	1.448	6.427	12.815	13.831	90.00	90.00	90.00
XXII_R033	33	1.927	P21/c	1.536	6.121	16.838	11.034	90.00	109.21	90.00
XXII_R034	34	1.947	P21/c	1.473	8.610	6.293	20.819	90.00	96.98	90.00
XXII_R035	35	1.950	Pna21	1.543	22.954	7.529	6.186	90.00	90.00	90.00
XXII_R036	36	2.003	Pca21	1.460	20.134	6.486	8.652	90.00	90.00	90.00
XXII_R037	37	2.007	P21/c	1.496	7.200	7.553	20.444	90.00	97.40	90.00
XXII_R038	38	2.033	Pna21	1.439	22.820	7.131	7.043	90.00	90.00	90.00
XXII_R039	39	2.086	Pc	1.504	7.084	10.299	9.446	90.00	127.30	90.00
XXII_R040	40	2.140	Pbcn	1.513	8.939	25.587	9.533	90.00	90.00	90.00
XXII_R041	41	2.247	Pbca	1.477	12.207	9.395	19.470	90.00	90.00	90.00
XXII_R042	42	2.274	C2/c	1.475	19.288	9.945	11.699	90.00	94.73	90.00
XXII_R043	43	2.291	Pna21	1.542	12.574	10.365	8.210	90.00	90.00	90.00
XXII_R044	44	2.321	Pbca	1.451	13.292	8.112	21.090	90.00	90.00	90.00
XXII_R045	45	2.329	P21/c	1.522	7.200	14.296	12.262	90.00	120.83	90.00
XXII_R046	46	2.332	C2/c	1.406	19.264	9.743	14.148	90.00	117.92	90.00
XXII_R047	47	2.337	P21/c	1.528	7.933	8.823	15.893	90.00	103.98	90.00
XXII_R048	48	2.349	Pca21	1.434	11.911	10.342	9.339	90.00	90.00	90.00
XXII_R049	49	2.371	P21/c	1.522	10.906	7.196	13.975	90.00	98.90	90.00
XXII_R050	50	2.402	P21/c	1.408	7.359	18.804	8.705	90.00	103.45	90.00

XXII_R051	51	2.451	Pna21	1.463	20.610	8.433	6.487	90.00	90.00	90.00
XXII_R052	52	2.455	Pbca	1.514	12.118	8.489	21.184	90.00	90.00	90.00
XXII_R053	53	2.459	P21/c	1.550	14.156	7.580	10.356	90.00	106.78	90.00
XXII_R054	54	2.475	Pbca	1.535	14.245	8.180	18.445	90.00	90.00	90.00
XXII_R055	55	2.503	P21	1.455	6.499	8.400	10.398	90.00	92.96	90.00
XXII_R056	56	2.528	Pbca	1.555	7.570	10.140	27.636	90.00	90.00	90.00
XXII_R057	57	2.550	P21/c	1.457	6.153	20.132	10.897	90.00	122.99	90.00
XXII_R058	58	2.554	Pna21	1.471	13.093	10.901	7.855	90.00	90.00	90.00
XXII_R059	59	2.558	P21/c	1.535	9.536	10.620	10.611	90.00	91.25	90.00
XXII_R060	60	2.558	P21/c	1.494	11.089	9.320	12.434	90.00	120.80	90.00
XXII_R061	61	2.564	Pc	1.490	8.356	6.513	11.210	90.00	114.88	90.00
XXII_R062	62	2.568	Pbca	1.456	9.811	11.678	19.776	90.00	90.00	90.00
XXII_R063	63	2.574	Pna21	1.502	22.220	6.840	7.225	90.00	90.00	90.00
XXII_R064	64	2.592	P21	1.459	6.885	8.168	10.390	90.00	104.64	90.00
XXII_R065	65	2.596	Pbca	1.459	11.025	10.690	19.190	90.00	90.00	90.00
XXII_R066	66	2.615	P212121	1.474	6.788	8.306	19.844	90.00	90.00	90.00
XXII_R067	67	2.648	P21/c	1.483	7.188	18.894	9.568	90.00	121.13	90.00
XXII_R068	68	2.655	Pca21	1.504	10.932	10.517	9.541	90.00	90.00	90.00
XXII_R069	69	2.662	P21/c	1.510	6.814	21.493	9.976	90.00	131.60	90.00
XXII_R070	70	2.667	Pna21	1.457	19.271	9.291	6.322	90.00	90.00	90.00
XXII_R071	71	2.683	Pna21	1.449	9.984	17.379	6.559	90.00	90.00	90.00
XXII_R072	72	2.707	P21/c	1.475	13.795	7.476	10.849	90.00	91.08	90.00
XXII_R073	73	2.711	P21/c	1.478	10.908	7.001	14.724	90.00	96.98	90.00
XXII_R074	74	2.730	Pbca	1.515	9.873	10.691	20.631	90.00	90.00	90.00
XXII_R075	75	2.738	P21/c	1.478	11.058	9.844	11.687	90.00	118.72	90.00
XXII_R076	76	2.742	C2/c	1.472	21.901	7.100	14.426	90.00	92.11	90.00
XXII_R077	77	2.765	P21/c	1.505	6.825	7.486	21.646	90.00	97.59	90.00
XXII_R078	78	2.767	P21/c	1.479	7.272	18.536	9.676	90.00	121.24	90.00
XXII_R079	79	2.774	P21/c	1.452	10.044	14.223	8.015	90.00	97.26	90.00
XXII_R080	80	2.779	Pbca	1.529	13.371	10.385	15.539	90.00	90.00	90.00
XXII_R081	81	2.785	Pna21	1.501	24.041	6.099	7.493	90.00	90.00	90.00
XXII_R082	82	2.834	Pca21	1.488	9.220	12.758	9.421	90.00	90.00	90.00
XXII_R083	83	2.852	P212121	1.478	6.974	9.340	17.135	90.00	90.00	90.00
XXII_R084	84	2.855	Pbcn	1.481	8.108	21.224	12.945	90.00	90.00	90.00
XXII_R085	85	2.922	Pbcn	1.421	20.034	12.354	9.382	90.00	90.00	90.00
XXII_R086	86	2.939	Pna21	1.473	20.881	7.155	7.497	90.00	90.00	90.00
XXII_R087	87	2.944	P212121	1.458	6.003	10.028	18.799	90.00	90.00	90.00
XXII_R088	88	2.971	P21/c	1.476	8.413	6.505	20.460	90.00	93.75	90.00
XXII_R089	89	2.975	Pc	1.463	6.177	8.901	11.097	90.00	112.50	90.00
XXII_R090	90	3.017	P21/c	1.505	9.311	9.087	13.739	90.00	109.42	90.00
XXII_R091	91	3.037	C2/c	1.527	12.699	26.742	8.647	90.00	132.62	90.00
XXII_R092	92	3.063	P21/c	1.450	9.688	9.752	12.037	90.00	90.26	90.00
XXII_R093	93	3.064	P21/c	1.447	9.688	6.071	19.477	90.00	95.56	90.00
XXII_R094	94	3.066	P21/c	1.423	13.407	6.495	13.380	90.00	95.56	90.00
XXII_R095	95	3.068	Pca21	1.484	11.743	9.917	9.546	90.00	90.00	90.00
XXII_R096	96	3.089	P212121	1.426	6.903	7.153	23.433	90.00	90.00	90.00
XXII_R097	97	3.109	P21/c	1.425	9.679	7.149	16.791	90.00	94.87	90.00
XXII_R098	98	3.140	P212121	1.529	6.093	7.606	23.271	90.00	90.00	90.00
XXII_R099	99	3.173	P21/c	1.421	7.127	19.000	9.040	90.00	108.56	90.00
XXII_R100	100	3.176	Pbca	1.458	12.294	10.551	17.440	90.00	90.00	90.00
XXII_R101	101	3.187	P21/c	1.395	12.733	7.395	15.442	90.00	125.58	90.00
XXII_R102	102	3.203	P21/c	1.459	12.708	7.525	14.198	90.00	123.64	90.00
XXII_R103	103	3.213	P212121	1.445	6.837	8.359	19.975	90.00	90.00	90.00
XXII_R104	104	3.214	C2/c	1.465	13.162	9.948	18.035	90.00	107.54	90.00

XXII_R105	105	3.214	P212121	1.470	7.051	10.896	14.607	90.00	90.00	90.00
XXII_R106	106	3.216	Pna21	1.438	17.428	10.347	6.360	90.00	90.00	90.00
XXII_R107	107	3.217	Pbca	1.461	8.609	11.735	22.344	90.00	90.00	90.00
XXII_R108	108	3.218	P21/c	1.436	15.019	7.152	11.148	90.00	106.45	90.00
XXII_R109	109	3.258	Pca21	1.393	11.554	9.281	11.040	90.00	90.00	90.00
XXII_R110	110	3.299	Pbca	1.458	9.052	13.164	18.985	90.00	90.00	90.00
XXII_R111	111	3.303	Pbca	1.493	9.277	9.210	25.853	90.00	90.00	90.00
XXII_R112	112	3.317	P21/c	1.430	13.935	6.742	13.581	90.00	115.28	90.00
XXII_R113	113	3.326	P212121	1.405	7.911	8.957	16.570	90.00	90.00	90.00
XXII_R114	114	3.329	P21/c	1.446	9.046	13.601	9.275	90.00	91.90	90.00
XXII_R115	115	3.363	P21/c	1.457	7.514	8.411	18.145	90.00	99.10	90.00
XXII_R116	116	3.382	P21/c	1.435	11.054	8.730	13.871	90.00	120.86	90.00
XXII_R117	117	3.383	P212121	1.393	6.344	11.867	15.731	90.00	90.00	90.00
XXII_R118	118	3.389	P21/c	1.494	16.253	6.363	11.015	90.00	104.27	90.00
XXII_R119	119	3.423	P21/c	1.470	7.468	6.233	24.102	90.00	90.64	90.00
XXII_R120	120	3.424	P21/c	1.416	6.414	7.348	24.722	90.00	90.36	90.00
XXII_R121	121	3.427	C2/c	1.416	21.289	9.902	11.932	90.00	112.16	90.00
XXII_R122	122	3.450	Pna21	1.442	6.872	19.561	8.507	90.00	90.00	90.00
XXII_R123	123	3.450	P212121	1.515	7.066	8.555	18.011	90.00	90.00	90.00
XXII_R124	124	3.481	P21	1.400	6.727	7.196	12.204	90.00	94.34	90.00
XXII_R125	125	3.485	P21/c	1.465	11.515	6.516	15.035	90.00	93.84	90.00
XXII_R126	126	3.504	Pna21	1.416	12.327	10.693	8.838	90.00	90.00	90.00
XXII_R127	127	3.510	Pna21	1.417	14.026	12.750	6.507	90.00	90.00	90.00
XXII_R128	128	3.514	P21/c	1.465	6.130	7.496	24.988	90.00	101.28	90.00
XXII_R129	129	3.527	P21/c	1.435	9.040	8.607	16.549	90.00	116.78	90.00
XXII_R130	130	3.546	P21/c	1.485	8.241	8.437	16.323	90.00	101.84	90.00
XXII_R131	131	3.558	P21/c	1.480	11.059	7.015	15.701	90.00	113.78	90.00
XXII_R132	132	3.589	P212121	1.473	6.433	10.424	16.699	90.00	90.00	90.00
XXII_R133	133	3.592	Pbca	1.479	8.841	10.558	23.899	90.00	90.00	90.00
XXII_R134	134	3.604	Pbcn	1.354	12.134	15.138	13.268	90.00	90.00	90.00
XXII_R135	135	3.626	P21/c	1.423	12.797	6.909	15.562	90.00	122.62	90.00
XXII_R136	136	3.627	Pca21	1.468	11.379	6.618	14.918	90.00	90.00	90.00
XXII_R137	137	3.675	Pna21	1.484	13.003	10.081	8.480	90.00	90.00	90.00
XXII_R138	138	3.692	Pna21	1.448	6.852	17.023	9.767	90.00	90.00	90.00
XXII_R139	139	3.717	P21/c	1.482	13.978	9.639	8.686	90.00	108.00	90.00
XXII_R140	140	3.781	Pna21	1.472	7.626	24.163	6.081	90.00	90.00	90.00
XXII_R141	141	3.786	P21/c	1.472	16.583	8.729	7.924	90.00	102.28	90.00
XXII_R142	142	3.789	P21/c	1.495	7.183	18.484	9.703	90.00	121.05	90.00
XXII_R143	143	3.792	Pna21	1.462	8.783	13.705	9.371	90.00	90.00	90.00
XXII_R144	144	3.828	Pbca	1.485	8.656	8.226	31.200	90.00	90.00	90.00
XXII_R145	145	3.860	P21/c	1.459	13.794	6.509	14.600	90.00	120.41	90.00
XXII_R146	146	3.879	Pbcn	1.479	31.258	8.638	8.262	90.00	90.00	90.00
XXII_R147	147	3.880	Pbcn	1.396	14.132	13.669	12.231	90.00	90.00	90.00
XXII_R148	148	3.894	P21/c	1.472	6.546	20.939	9.045	90.00	115.33	90.00
XXII_R149	149	3.923	P21/c	1.452	13.711	6.466	14.870	90.00	120.51	90.00
XXII_R150	150	3.975	C2/c	1.485	19.671	7.056	16.153	90.00	97.85	90.00
XXII_R151	151	4.004	P21/c	1.443	7.083	19.094	9.211	90.00	113.40	90.00
XXII_R152	152	4.005	Pna21	1.459	16.861	10.673	6.284	90.00	90.00	90.00
XXII_R153	153	4.009	Pbcn	1.458	19.338	8.747	13.376	90.00	90.00	90.00
XXII_R154	154	4.046	P21	1.490	7.477	8.531	8.723	90.00	95.90	90.00
XXII_R155	155	4.051	P212121	1.389	6.840	7.856	22.105	90.00	90.00	90.00
XXII_R156	156	4.059	Pbcn	1.480	10.024	20.750	10.719	90.00	90.00	90.00
XXII_R157	157	4.080	P21/c	1.417	8.091	13.555	10.629	90.00	92.95	90.00
XXII_R158	158	4.100	P21	1.494	5.913	7.581	12.370	90.00	95.41	90.00

XXII_R159	159	4.107	Pbcn	1.438	19.568	8.154	14.374	90.00	90.00	90.00
XXII_R160	160	4.115	C2/c	1.476	31.881	8.648	8.107	90.00	90.44	90.00
XXII_R161	161	4.157	P21/c	1.436	6.898	8.515	19.761	90.00	98.15	90.00
XXII_R162	162	4.196	Pca21	1.397	10.945	9.457	11.403	90.00	90.00	90.00
XXII_R163	163	4.198	P212121	1.487	6.566	9.189	18.388	90.00	90.00	90.00
XXII_R164	164	4.222	Pbcn	1.477	31.840	7.379	9.509	90.00	90.00	90.00
XXII_R165	165	4.228	P21/c	1.439	8.016	10.603	13.602	90.00	97.43	90.00
XXII_R166	166	4.232	Pbca	1.390	7.512	12.310	25.674	90.00	90.00	90.00
XXII_R167	167	4.269	Pbca	1.460	8.913	13.330	19.022	90.00	90.00	90.00
XXII_R168	168	4.291	P212121	1.473	6.485	9.573	18.035	90.00	90.00	90.00
XXII_R169	169	4.304	P212121	1.472	7.215	9.087	17.089	90.00	90.00	90.00
XXII_R170	170	4.347	P21/c	1.429	11.479	6.401	16.043	90.00	101.66	90.00
XXII_R171	171	4.387	Pbca	1.401	8.095	16.364	17.778	90.00	90.00	90.00
XXII_R172	172	4.391	Pbca	1.473	8.499	8.261	31.903	90.00	90.00	90.00
XXII_R173	173	4.395	Pna21	1.416	13.384	10.943	7.955	90.00	90.00	90.00
XXII_R174	174	4.418	Pbcn	1.355	19.940	9.876	12.361	90.00	90.00	90.00
XXII_R175	175	4.419	P212121	1.408	7.292	11.262	14.264	90.00	90.00	90.00
XXII_R176	176	4.423	Pna21	1.460	16.481	10.308	6.648	90.00	90.00	90.00
XXII_R177	177	4.456	Pbca	1.380	9.641	12.459	19.901	90.00	90.00	90.00
XXII_R178	178	4.463	Pbca	1.477	7.030	16.046	19.803	90.00	90.00	90.00
XXII_R179	179	4.492	P21/c	1.489	13.435	8.813	9.881	90.00	108.76	90.00
XXII_R180	180	4.576	P21/c	1.397	7.463	9.562	16.769	90.00	99.50	90.00
XXII_R181	181	4.592	P21/c	1.466	11.792	8.161	13.503	90.00	120.01	90.00
XXII_R182	182	4.611	Pbca	1.423	7.371	9.382	33.529	90.00	90.00	90.00
XXII_R183	183	4.642	C2/c	1.440	19.843	7.929	14.563	90.00	90.48	90.00
XXII_R184	184	4.678	C2/c	1.484	22.436	7.017	15.855	90.00	117.09	90.00
XXII_R185	185	4.759	Pbcn	1.432	24.349	8.489	11.145	90.00	90.00	90.00
XXII_R186	186	4.860	Pbca	1.391	11.710	8.638	23.440	90.00	90.00	90.00
XXII_R187	187	4.911	Pbcn	1.440	19.125	8.574	13.967	90.00	90.00	90.00
XXII_R188	188	5.020	Pc	1.463	6.583	10.506	9.178	90.00	117.34	90.00
XXII_R189	189	5.050	P21/c	1.428	12.207	7.899	14.741	90.00	125.63	90.00
XXII_R190	190	5.070	Pbca	1.438	8.534	14.047	19.143	90.00	90.00	90.00
XXII_R191	191	5.122	Cc	1.413	8.622	21.075	6.925	90.00	111.96	90.00
XXII_R192	192	5.124	P21/c	1.493	10.802	12.368	8.566	90.00	105.20	90.00
XXII_R193	193	5.124	Pbca	1.391	6.504	16.703	21.825	90.00	90.00	90.00
XXII_R194	194	5.193	Pna21	1.424	8.451	20.436	6.709	90.00	90.00	90.00
XXII_R195	195	5.200	P21/c	1.433	9.758	7.978	14.791	90.00	91.30	90.00
XXII_R196	196	5.201	C2/c	1.432	23.954	8.040	14.624	90.00	125.11	90.00
XXII_R197	197	5.861	P21/c	1.397	13.203	6.328	16.157	90.00	118.96	90.00
XXII_R198	198	5.976	C2/c	1.415	24.946	8.192	11.406	90.00	90.48	90.00
XXII_R199	199	6.021	Pbca	1.425	7.927	14.886	19.622	90.00	90.00	90.00
XXII_R200	200	6.769	P21/c	1.411	10.455	7.492	15.006	90.00	95.88	90.00

Table S2. List of predicted crystal structures of XXIII

Label	Rank	$\Delta E_{\text{PBE-TS}}$ /kcal·mol^{-1}	Space Group	Density /g·cm^{-3}	a /Å	b /Å	c /Å	α /°	β /°	γ /°
XXIII_R001	1	0.000	P21/c	1.306	4.632	24.672	17.210	90.00	93.08	90.00
XXIII_R002	2	0.163	P21/c	1.310	7.823	27.637	9.811	90.00	112.54	90.00
XXIII_R003	3	0.199	P21/c	1.289	4.569	15.658	27.822	90.00	90.42	90.00
XXIII_R004	4	0.244	P-1	1.291	4.652	12.324	17.697	78.42	88.05	88.85
XXIII_R005	5	0.290	P-1	1.311	7.820	9.729	14.285	78.45	80.45	67.46
XXIII_R006	6	0.794	P-1	1.297	6.867	9.261	16.147	85.90	80.37	77.82
XXIII_R007	7	1.126	P-1	1.283	8.044	12.087	12.154	61.26	88.41	75.94
XXIII_R008	8	1.162	P21/c	1.290	4.624	29.714	14.495	90.00	92.59	90.00
XXIII_R009	9	1.234	P21/c	1.281	7.894	34.846	7.579	90.00	106.05	90.00
XXIII_R010	10	1.236	P-1	1.269	7.250	10.208	14.668	83.62	76.20	73.80
XXIII_R011	11	1.267	P21/c	1.285	4.661	12.457	34.400	90.00	91.11	90.00
XXIII_R012	12	1.305	P21/c	1.266	17.616	4.632	25.062	90.00	97.83	90.00
XXIII_R013	13	1.307	P-1	1.286	7.209	8.210	18.664	87.41	84.76	65.04
XXIII_R014	14	1.328	P21/c	1.293	12.254	4.797	33.900	90.00	95.60	90.00
XXIII_R015	15	1.368	P212121	1.301	4.783	12.255	33.643	90.00	90.00	90.00
XXIII_R016	16	1.526	P-1	1.281	8.645	10.707	11.715	87.48	83.49	68.38
XXIII_R017	17	1.652	P-1	1.295	7.483	10.487	13.675	78.09	83.36	70.79
XXIII_R018	18	1.687	P-1	1.264	7.603	7.810	18.604	81.90	84.24	68.30
XXIII_R019	19	1.807	P21/c	1.292	12.902	10.909	16.540	90.00	121.48	90.00
XXIII_R020	20	1.840	P-1	1.280	8.633	10.723	10.989	91.88	99.66	90.77
XXIII_R021	21	1.897	P-1	1.267	7.810	12.149	12.169	63.48	86.78	78.69
XXIII_R022	22	2.075	P21/c	1.291	4.803	42.843	10.134	90.00	107.69	90.00
XXIII_R023	23	2.083	P-1	1.278	6.944	8.732	17.980	89.48	82.98	68.14
XXIII_R024	24	2.138	P21/c	1.262	19.595	9.786	10.609	90.00	92.59	90.00
XXIII_R025	25	2.235	P21/c	1.286	19.689	4.640	25.151	90.00	119.74	90.00
XXIII_R026	26	2.257	P-1	1.289	6.985	9.143	16.009	86.40	83.16	78.78
XXIII_R027	27	2.354	P21/c	1.269	14.648	9.378	17.040	90.00	120.28	90.00
XXIII_R028	28	2.391	P21/c	1.304	7.711	27.848	9.948	90.00	112.94	90.00
XXIII_R029	29	2.418	P-1	1.259	8.193	10.912	12.905	109.80	105.39	96.78
XXIII_R030	30	2.422	P21/c	1.297	4.698	15.727	26.787	90.00	91.43	90.00
XXIII_R031	31	2.448	P-1	1.307	7.689	9.868	14.528	94.12	101.88	112.63
XXIII_R032	32	2.530	P-1	1.267	7.882	8.027	17.866	82.98	81.35	65.26
XXIII_R033	33	2.541	P21/c	1.258	7.809	12.251	21.579	90.00	98.96	90.00
XXIII_R034	34	2.571	P-1	1.280	4.781	12.351	17.436	77.88	84.51	88.83
XXIII_R035	35	2.606	P21/c	1.246	12.821	9.473	18.027	90.00	109.83	90.00
XXIII_R036	36	2.612	P21/c	1.260	7.017	38.698	8.322	90.00	115.75	90.00
XXIII_R037	37	2.639	P-1	1.271	7.541	7.744	19.173	83.70	82.89	65.55
XXIII_R038	38	2.648	P-1	1.291	4.958	9.758	21.298	76.86	85.59	82.44
XXIII_R039	39	2.651	P-1	1.258	9.314	9.337	14.224	71.17	84.71	60.86
XXIII_R040	40	2.711	P-1	1.259	7.998	11.115	11.570	85.44	87.13	84.09
XXIII_R041	41	2.743	P21/c	1.298	4.706	24.624	17.127	90.00	95.33	90.00
XXIII_R042	42	2.756	P-1	1.276	8.796	9.717	12.361	91.16	106.99	94.88
XXIII_R043	43	2.838	P21/c	1.300	12.594	13.864	11.475	90.00	100.00	90.00
XXIII_R044	44	2.950	P-1	1.274	8.871	10.738	11.855	109.32	91.69	107.32
XXIII_R045	45	2.965	P-1	1.251	8.607	9.631	12.750	80.24	85.54	80.38
XXIII_R046	46	2.991	P-1	1.284	7.180	8.167	18.926	87.77	83.53	64.93
XXIII_R047	47	3.027	C2/c	1.232	14.874	7.787	35.960	90.00	91.51	90.00
XXIII_R048	48	3.078	P-1	1.258	8.307	9.984	12.586	82.36	81.08	84.79
XXIII_R049	49	3.142	P-1	1.292	8.024	10.405	12.117	93.85	95.74	98.14
XXIII_R050	50	3.166	P-1	1.275	7.373	10.200	14.167	85.35	78.16	74.80

XXIII_R051	51	3.200	C2/c	1.250	25.676	13.396	13.040	90.00	113.75	90.00
XXIII_R052	52	3.222	P21/c	1.281	7.712	11.755	22.319	90.00	98.10	90.00
XXIII_R053	53	3.243	P21/c	1.248	7.797	39.452	7.241	90.00	112.66	90.00
XXIII_R054	54	3.267	P-1	1.264	9.233	11.123	11.149	100.40	104.62	107.52
XXIII_R055	55	3.268	P-1	1.271	7.287	11.719	12.868	106.55	104.18	94.17
XXIII_R056	56	3.278	P-1	1.320	8.751	11.144	11.405	66.19	89.54	74.10
XXIII_R057	57	3.299	P-1	1.219	7.311	7.588	20.690	83.58	87.68	67.27
XXIII_R058	58	3.316	P21/c	1.263	9.014	18.896	12.716	90.00	110.25	90.00
XXIII_R059	59	3.343	P-1	1.292	7.724	7.806	17.852	92.58	95.71	111.41
XXIII_R060	60	3.373	P21/c	1.237	13.014	12.753	12.579	90.00	96.63	90.00
XXIII_R061	61	3.376	P21/c	1.257	7.163	37.369	9.829	90.00	129.11	90.00
XXIII_R062	62	3.388	P-1	1.268	9.039	9.102	13.435	102.02	98.09	106.65
XXIII_R063	63	3.427	P-1	1.305	8.189	10.412	11.652	91.94	90.83	98.03
XXIII_R064	64	3.435	P-1	1.264	6.912	8.295	18.897	86.79	87.85	69.83
XXIII_R065	65	3.453	P-1	1.271	7.876	7.897	17.915	84.29	83.77	65.93
XXIII_R066	66	3.462	P21/c	1.248	14.087	11.337	14.705	90.00	118.91	90.00
XXIII_R067	67	3.470	P-1	1.274	8.113	10.077	12.973	79.67	74.94	85.09
XXIII_R068	68	3.471	P-1	1.247	9.202	9.938	13.418	107.07	95.95	114.75
XXIII_R069	69	3.488	P21/c	1.275	16.788	15.365	7.879	90.00	98.04	90.00
XXIII_R070	70	3.493	P-1	1.270	9.159	10.821	11.547	97.35	101.09	112.64
XXIII_R071	71	3.493	P-1	1.250	9.258	9.947	11.772	89.51	89.68	71.22
XXIII_R072	72	3.542	P-1	1.270	7.793	11.836	12.278	64.46	81.60	88.83
XXIII_R073	73	3.597	P-1	1.276	6.983	8.012	19.283	80.94	87.49	70.63
XXIII_R074	74	3.640	P-1	1.260	7.566	10.985	12.773	91.56	98.48	103.68
XXIII_R075	75	3.662	P21/c	1.277	4.819	12.522	33.334	90.00	93.26	90.00
XXIII_R076	76	3.677	P21/c	1.283	8.695	11.272	22.165	90.00	112.97	90.00
XXIII_R077	77	3.709	C2/c	1.283	21.890	14.340	12.785	90.00	94.54	90.00
XXIII_R078	78	3.716	C2/c	1.233	7.277	14.635	39.157	90.00	93.69	90.00
XXIII_R079	79	3.728	P-1	1.281	8.427	10.851	11.058	90.37	91.29	97.83
XXIII_R080	80	3.820	P-1	1.265	6.647	11.281	13.972	83.88	85.87	77.13
XXIII_R081	81	3.822	P-1	1.271	4.842	12.990	16.496	101.55	94.34	94.21
XXIII_R082	82	3.848	P21/c	1.276	15.537	16.311	8.214	90.00	104.93	90.00
XXIII_R083	83	3.850	P-1	1.260	8.337	10.098	13.174	86.54	86.42	66.99
XXIII_R084	84	3.853	P-1	1.266	7.780	10.622	12.950	81.13	80.22	75.33
XXIII_R085	85	3.853	C2/c	1.252	7.930	33.518	15.584	90.00	98.38	90.00
XXIII_R086	86	3.888	P-1	1.264	8.937	10.271	11.718	88.43	71.09	86.19
XXIII_R087	87	3.901	C2/c	1.299	30.935	6.856	21.523	90.00	120.08	90.00
XXIII_R088	88	3.915	P-1	1.219	7.272	9.275	16.370	84.87	81.95	74.61
XXIII_R089	89	3.977	P21/c	1.243	15.943	15.369	8.557	90.00	100.20	90.00
XXIII_R090	90	3.983	P-1	1.287	8.607	10.640	11.917	104.22	92.93	107.90
XXIII_R091	91	3.991	P-1	1.298	7.551	9.765	14.680	92.85	102.00	109.66
XXIII_R092	92	4.015	P-1	1.267	8.069	10.734	12.874	66.51	81.95	87.00
XXIII_R093	93	4.058	P-1	1.274	6.094	12.312	14.115	98.33	100.98	99.79
XXIII_R094	94	4.093	P21/c	1.255	12.126	9.639	18.690	90.00	110.65	90.00
XXIII_R095	95	4.114	P-1	1.297	6.691	10.466	14.439	94.56	94.34	99.79
XXIII_R096	96	4.138	P-1	1.211	7.631	7.828	19.319	85.57	81.99	68.07
XXIII_R097	97	4.161	P21/c	1.258	7.066	38.866	8.147	90.00	114.23	90.00
XXIII_R098	98	4.243	C2/c	1.179	23.008	13.455	14.067	90.00	92.11	90.00
XXIII_R099	99	4.325	C2/c	1.239	31.241	8.577	15.591	90.00	97.42	90.00
XXIII_R100	100	4.326	P-1	1.272	8.852	9.756	12.744	84.37	87.14	67.04
XXIII_R101	101	4.343	P21/c	1.247	8.049	33.720	7.888	90.00	106.02	90.00
XXIII_R102	102	4.351	P-1	1.270	7.232	9.493	15.639	94.35	99.34	106.01
XXIII_R103	103	4.407	P21/c	1.261	11.653	14.355	12.756	90.00	107.51	90.00
XXIII_R104	104	4.438	C2/c	1.251	14.484	7.852	36.301	90.00	96.37	90.00

XXIII_R105	105	4.461	P21/c	1.289	10.094	27.899	7.601	90.00	111.58	90.00
XXIII_R106	106	4.462	P21/c	1.211	7.565	38.462	7.789	90.00	110.76	90.00
XXIII_R107	107	4.484	P-1	1.257	8.841	9.368	13.090	79.72	73.07	86.69
XXIII_R108	108	4.505	P-1	1.211	7.670	7.797	19.437	100.01	92.44	111.31
XXIII_R109	109	4.510	P-1	1.272	7.728	7.835	18.947	82.90	82.92	62.66
XXIII_R110	110	4.521	P-1	1.249	7.818	7.942	18.980	89.86	78.24	63.52
XXIII_R111	111	4.537	P21/c	1.295	18.000	13.531	8.357	90.00	103.18	90.00
XXIII_R112	112	4.550	P-1	1.257	9.109	9.515	14.155	76.02	72.12	61.63
XXIII_R113	113	4.551	C2/c	1.214	8.223	13.464	38.201	90.00	91.24	90.00
XXIII_R114	114	4.558	P21/c	1.212	7.745	38.786	7.502	90.00	110.08	90.00
XXIII_R115	115	4.562	P-1	1.251	8.692	8.992	14.886	81.62	78.93	64.18
XXIII_R116	116	4.591	P-1	1.275	6.928	7.681	19.399	78.85	89.85	83.42
XXIII_R117	117	4.607	P21/c	1.262	9.790	22.313	9.443	90.00	99.78	90.00
XXIII_R118	118	4.609	P-1	1.265	8.756	9.131	13.983	108.53	96.17	102.87
XXIII_R119	119	4.626	P21/c	1.243	10.954	11.486	16.449	90.00	94.31	90.00
XXIII_R120	120	4.627	P-1	1.269	4.678	13.419	17.228	110.01	90.34	95.30
XXIII_R121	121	4.658	P-1	1.277	8.257	10.268	12.188	87.95	84.84	77.46
XXIII_R122	122	4.707	P-1	1.260	6.997	8.549	18.156	90.91	97.55	108.69
XXIII_R123	123	4.709	P-1	1.279	5.458	12.253	15.414	98.61	93.29	99.16
XXIII_R124	124	4.718	P21/c	1.246	16.426	15.570	8.155	90.00	99.15	90.00
XXIII_R125	125	4.725	P-1	1.271	7.823	7.829	18.058	83.11	83.41	67.18
XXIII_R126	126	4.736	P21/c	1.248	7.771	34.425	9.692	90.00	127.53	90.00
XXIII_R127	127	4.737	P21/c	1.266	7.818	37.983	7.709	90.00	117.75	90.00
XXIII_R128	128	4.748	C2/c	1.197	8.631	13.035	38.109	90.00	92.00	90.00
XXIII_R129	129	4.794	C2/c	1.279	26.016	6.135	25.137	90.00	91.48	90.00
XXIII_R130	130	4.807	P21/c	1.286	9.593	19.702	11.124	90.00	108.34	90.00
XXIII_R131	131	4.822	P-1	1.247	9.407	9.799	12.160	112.24	96.37	91.43
XXIII_R132	132	4.843	C2/c	1.218	12.507	8.793	38.460	90.00	95.27	90.00
XXIII_R133	133	4.860	P-1	1.238	9.232	10.916	11.254	72.11	81.39	74.30
XXIII_R134	134	4.872	P-1	1.262	8.338	9.788	13.723	81.34	79.59	67.94
XXIII_R135	135	4.908	C2/c	1.207	13.003	8.682	37.882	90.00	96.30	90.00
XXIII_R136	136	4.914	P-1	1.257	9.123	9.881	12.018	85.72	87.93	70.85
XXIII_R137	137	4.922	P-1	1.280	7.939	10.641	12.260	98.60	91.82	101.39
XXIII_R138	138	4.931	C2/c	1.249	13.389	7.989	38.906	90.00	99.24	90.00
XXIII_R139	139	4.947	C2/c	1.282	33.673	14.222	8.468	90.00	99.16	90.00
XXIII_R140	140	4.987	C2/c	1.268	35.632	7.095	16.055	90.00	94.64	90.00
XXIII_R141	141	4.987	P-1	1.237	8.580	9.476	13.242	78.82	85.12	79.41
XXIII_R142	142	4.998	C2/c	1.261	23.149	11.709	16.419	90.00	113.84	90.00
XXIII_R143	143	5.034	P-1	1.277	7.304	11.654	12.603	104.87	101.59	94.95
XXIII_R144	144	5.045	P21/c	1.208	7.383	41.400	7.558	90.00	113.17	90.00
XXIII_R145	145	5.047	P-1	1.263	8.566	9.906	12.538	88.19	73.55	84.69
XXIII_R146	146	5.075	P21/c	1.250	16.706	8.371	15.000	90.00	102.00	90.00
XXIII_R147	147	5.076	P-1	1.273	8.875	10.570	12.153	99.84	100.10	111.72
XXIII_R148	148	5.118	P-1	1.253	8.731	10.715	11.975	105.83	92.08	106.84
XXIII_R149	149	5.121	P21/c	1.233	8.054	9.084	29.452	90.00	105.03	90.00
XXIII_R150	150	5.130	P21/c	1.291	16.193	7.863	19.177	90.00	125.51	90.00
XXIII_R151	151	5.134	P21/c	1.270	10.631	14.793	13.649	90.00	109.76	90.00
XXIII_R152	152	5.151	P-1	1.187	7.653	7.774	19.689	92.39	90.18	112.54
XXIII_R153	153	5.187	P21/c	1.260	7.405	14.790	19.336	90.00	105.89	90.00
XXIII_R154	154	5.255	P21/c	1.242	11.259	11.838	15.497	90.00	90.03	90.00
XXIII_R155	155	5.311	P21/c	1.290	15.331	10.468	13.673	90.00	115.03	90.00
XXIII_R156	156	5.328	P-1	1.300	7.789	7.866	17.815	90.58	97.00	114.07
XXIII_R157	157	5.328	P-1	1.265	8.902	9.455	12.940	69.20	88.10	84.97
XXIII_R158	158	5.349	P-1	1.218	7.313	9.190	16.247	85.53	84.58	75.95

XXIII_R159	159	5.349	C2/c	1.269	33.045	7.777	18.642	90.00	122.41	90.00
XXIII_R160	160	5.414	P-1	1.256	6.944	8.139	19.203	88.14	86.85	70.53
XXIII_R161	161	5.444	P-1	1.295	8.897	10.388	12.013	107.18	92.47	109.00
XXIII_R162	162	5.461	P-1	1.220	7.285	7.706	20.901	82.54	85.77	64.70
XXIII_R163	163	5.471	C2/c	1.233	25.302	13.524	13.520	90.00	115.91	90.00
XXIII_R164	164	5.485	C2/c	1.250	21.132	8.777	23.800	90.00	111.61	90.00
XXIII_R165	165	5.486	P-1	1.272	9.458	10.452	12.252	103.93	111.15	105.54
XXIII_R166	166	5.489	C2/c	1.230	7.291	14.555	39.451	90.00	94.98	90.00
XXIII_R167	167	5.506	P-1	1.282	8.696	9.796	12.306	88.71	80.74	75.30
XXIII_R168	168	5.529	P-1	1.268	7.554	11.663	11.823	79.65	84.79	81.63
XXIII_R169	169	5.530	P-1	1.311	6.810	11.621	12.904	96.15	95.66	103.61
XXIII_R170	170	5.531	P21/c	1.251	13.067	19.022	8.519	90.00	104.45	90.00
XXIII_R171	171	5.537	P21/c	1.256	7.015	37.994	9.650	90.00	127.42	90.00
XXIII_R172	172	5.555	C2/c	1.262	32.108	8.407	15.142	90.00	95.93	90.00
XXIII_R173	173	5.558	C2/c	1.222	22.019	8.441	24.655	90.00	113.61	90.00
XXIII_R174	174	5.572	P-1	1.266	8.964	10.244	11.546	85.54	73.55	87.25
XXIII_R175	175	5.610	P-1	1.233	7.973	8.137	18.453	91.46	97.00	118.38
XXIII_R176	176	5.731	P21/c	1.273	18.223	6.657	16.742	90.00	97.18	90.00
XXIII_R177	177	5.834	C2/c	1.242	17.165	9.641	26.082	90.00	106.77	90.00
XXIII_R178	178	5.838	P21/c	1.262	17.614	7.727	15.416	90.00	104.39	90.00
XXIII_R179	179	5.916	C2/c	1.239	29.348	16.676	8.471	90.00	92.56	90.00
XXIII_R180	180	5.918	P21/c	1.256	7.773	6.785	39.219	90.00	98.92	90.00
XXIII_R181	181	6.009	P21/c	1.216	7.646	39.748	7.944	90.00	119.05	90.00
XXIII_R182	182	6.014	P-1	1.285	7.917	10.335	12.467	90.38	93.57	101.35
XXIII_R183	183	6.016	P-1	1.249	8.683	8.989	15.306	76.40	79.64	62.58
XXIII_R184	184	6.043	P-1	1.244	7.611	10.654	13.261	83.72	80.10	77.54
XXIII_R185	185	6.055	Pbca	1.267	14.226	7.650	37.217	90.00	90.00	90.00
XXIII_R186	186	6.071	P-1	1.230	9.714	9.736	12.848	102.61	105.57	108.95
XXIII_R187	187	6.104	P-1	1.227	7.772	8.124	18.533	84.30	81.54	64.70
XXIII_R188	188	6.106	P21/c	1.279	10.861	10.934	19.575	90.00	120.36	90.00
XXIII_R189	189	6.115	C2/c	1.242	7.773	14.076	37.753	90.00	90.33	90.00
XXIII_R190	190	6.139	P-1	1.276	9.164	9.263	12.392	92.59	101.72	101.39
XXIII_R191	191	6.148	C2/c	1.220	25.183	12.553	14.745	90.00	115.59	90.00
XXIII_R192	192	6.184	C2/c	1.227	7.983	13.949	37.560	90.00	91.25	90.00
XXIII_R193	193	6.187	P21/c	1.217	8.300	36.555	7.893	90.00	118.33	90.00
XXIII_R194	194	6.227	C2/c	1.194	23.254	12.813	14.488	90.00	95.27	90.00
XXIII_R195	195	6.244	P21/c	1.283	8.629	22.758	12.587	90.00	126.00	90.00
XXIII_R196	196	6.296	Pbca	1.250	7.727	15.328	34.672	90.00	90.00	90.00
XXIII_R197	197	6.325	P212121	1.266	6.849	9.329	31.719	90.00	90.00	90.00
XXIII_R198	198	6.365	P-1	1.268	7.788	10.024	13.578	86.38	89.48	73.00
XXIII_R199	199	6.372	P21/c	1.245	15.258	8.592	15.782	90.00	94.91	90.00
XXIII_R200	200	6.491	P-1	1.222	7.575	8.929	16.248	82.75	82.21	75.59

Table S3. List of predicted crystal structures of XXV

Label	Rank	$\Delta E_{\text{PBE-TS}}$ /kcal·mol^{-1}	Space Group	Density /g·cm^{-3}	*a* /Å	*b* /Å	*c* /Å	*α* /°	*β* /°	*γ* /°
XXV_R001	1	0.000	P-1	1.291	6.484	13.985	14.979	112.25	97.27	103.19
XXV_R002	2	0.097	Pbcn	1.296	27.616	10.514	16.321	90.00	90.00	90.00
XXV_R003	3	0.269	P-1	1.297	6.785	11.675	16.272	105.40	93.68	105.60
XXV_R004	4	1.621	P21/c	1.267	16.729	6.773	22.392	90.00	107.12	90.00
XXV_R005	5	1.789	P21/c	1.299	8.388	20.466	13.772	90.00	90.22	90.00
XXV_R006	6	1.797	P-1	1.275	6.450	14.070	15.062	112.79	98.27	100.36
XXV_R007	7	2.068	Cc	1.264	11.578	10.389	20.219	90.00	92.07	90.00
XXV_R008	8	2.161	P21/c	1.301	7.313	19.052	17.276	90.00	101.19	90.00
XXV_R009	9	2.243	P21/c	1.244	16.287	6.863	22.885	90.00	105.09	90.00
XXV_R010	10	2.336	Pna21	1.291	10.211	14.142	16.482	90.00	90.00	90.00
XXV_R011	11	2.481	P21	1.286	7.248	20.239	8.143	90.00	91.05	90.00
XXV_R012	12	2.580	P212121	1.258	10.321	14.575	16.230	90.00	90.00	90.00
XXV_R013	13	2.850	P212121	1.292	7.634	16.323	19.074	90.00	90.00	90.00
XXV_R014	14	3.075	P21	1.288	10.866	10.792	11.062	90.00	113.16	90.00
XXV_R015	15	3.278	P-1	1.292	8.196	11.252	14.207	72.57	88.78	72.51
XXV_R016	16	3.328	P212121	1.281	7.176	16.451	20.307	90.00	90.00	90.00
XXV_R017	17	3.459	C2/c	1.230	34.217	7.172	22.488	90.00	115.17	90.00
XXV_R018	18	3.592	P21	1.274	7.641	19.773	7.979	90.00	90.94	90.00
XXV_R019	19	3.636	Pbca	1.245	10.309	16.409	29.161	90.00	90.00	90.00
XXV_R020	20	3.663	P21/c	1.269	10.845	28.915	8.128	90.00	108.18	90.00
XXV_R021	21	3.663	P212121	1.296	7.572	16.187	19.338	90.00	90.00	90.00
XXV_R022	22	3.806	P21/c	1.287	7.661	19.761	15.856	90.00	96.23	90.00
XXV_R023	23	3.816	P-1	1.214	6.421	10.499	19.747	99.56	95.66	103.29
XXV_R024	24	3.818	P212121	1.279	8.161	10.556	27.871	90.00	90.00	90.00
XXV_R025	25	3.881	Pbcn	1.275	27.646	10.462	16.660	90.00	90.00	90.00
XXV_R026	26	3.894	P-1	1.243	6.725	10.288	19.047	98.02	91.57	108.29
XXV_R027	27	3.895	Cc	1.261	10.638	24.596	9.750	90.00	107.33	90.00
XXV_R028	28	3.915	P21/c	1.271	14.570	8.164	21.827	90.00	111.40	90.00
XXV_R029	29	4.004	Pna21	1.285	7.606	16.042	19.598	90.00	90.00	90.00
XXV_R030	30	4.036	Pbca	1.210	10.388	10.431	46.844	90.00	90.00	90.00
XXV_R031	31	4.117	P21/c	1.283	8.252	20.236	14.340	90.00	90.00	90.00
XXV_R032	32	4.144	Pbca	1.247	10.260	16.766	28.637	90.00	90.00	90.00
XXV_R033	33	4.187	Pbca	1.249	11.032	11.466	38.887	90.00	90.00	90.00
XXV_R034	34	4.221	Pc	1.276	8.078	7.608	20.764	90.00	109.41	90.00
XXV_R035	35	4.233	P21	1.268	9.322	9.714	13.593	90.00	100.32	90.00
XXV_R036	36	4.234	P21/c	1.229	10.461	15.629	15.484	90.00	99.19	90.00
XXV_R037	37	4.273	P21/c	1.283	7.953	28.210	10.775	90.00	97.79	90.00
XXV_R038	38	4.277	P21/c	1.231	9.966	21.019	15.374	90.00	129.18	90.00
XXV_R039	39	4.333	P21/c	1.262	10.496	14.848	16.662	90.00	110.42	90.00
XXV_R040	40	4.358	Cc	1.253	7.137	21.240	16.243	90.00	95.35	90.00
XXV_R041	41	4.375	Cc	1.254	7.130	20.256	16.994	90.00	93.51	90.00
XXV_R042	42	4.425	P-1	1.301	8.382	9.553	16.395	90.96	93.54	115.55
XXV_R043	43	4.526	P-1	1.263	9.374	11.863	12.091	103.52	94.90	109.15
XXV_R044	44	4.553	P21/c	1.246	15.329	7.016	26.419	90.00	119.81	90.00
XXV_R045	45	4.572	Pbca	1.285	14.727	15.703	20.674	90.00	90.00	90.00
XXV_R046	46	4.603	P21/c	1.255	15.033	10.366	15.844	90.00	97.48	90.00
XXV_R047	47	4.679	C2/c	1.234	28.607	11.416	16.587	90.00	113.26	90.00
XXV_R048	48	4.685	P-1	1.225	6.405	10.369	20.341	76.87	84.34	72.43
XXV_R049	49	4.685	P21/c	1.283	10.580	15.229	15.686	90.00	108.75	90.00
XXV_R050	50	4.700	C2/c	1.253	30.331	10.752	15.614	90.00	105.59	90.00

XXV_R051	51	4.761	P212121	1.332	7.214	16.299	19.606	90.00	90.00	90.00
XXV_R052	52	4.776	P-1	1.223	7.124	14.082	14.272	62.05	84.17	84.28
XXV_R053	53	4.802	Cc	1.259	7.053	20.641	16.869	90.00	96.57	90.00
XXV_R054	54	4.844	C2/c	1.246	33.463	8.004	19.393	90.00	108.31	90.00
XXV_R055	55	4.872	P21/c	1.228	10.176	21.416	14.506	90.00	127.69	90.00
XXV_R056	56	4.933	P21/c	1.233	7.812	29.933	10.723	90.00	96.65	90.00
XXV_R057	57	4.948	P21	1.273	7.605	19.930	7.961	90.00	90.38	90.00
XXV_R058	58	5.006	P21/c	1.237	6.884	23.586	16.588	90.00	112.78	90.00
XXV_R059	59	5.015	P21/c	1.270	14.041	8.551	21.829	90.00	112.65	90.00
XXV_R060	60	5.023	P21/c	1.199	6.445	39.211	10.899	90.00	111.52	90.00
XXV_R061	61	5.098	Pbca	1.267	15.043	15.674	20.560	90.00	90.00	90.00
XXV_R062	62	5.104	P21/c	1.289	23.802	6.420	15.726	90.00	97.58	90.00
XXV_R063	63	5.118	C2/c	1.274	25.566	10.789	17.820	90.00	101.23	90.00
XXV_R064	64	5.159	P21/c	1.201	10.360	15.131	16.426	90.00	96.61	90.00
XXV_R065	65	5.160	C2/c	1.243	22.383	8.360	27.808	90.00	108.23	90.00
XXV_R066	66	5.194	P21/c	1.270	6.318	19.599	19.627	90.00	95.71	90.00
XXV_R067	67	5.291	Pbca	1.250	18.984	12.766	20.274	90.00	90.00	90.00
XXV_R068	68	5.298	P-1	1.283	8.273	9.601	16.593	92.67	92.93	114.21
XXV_R069	69	5.324	P-1	1.287	6.519	14.443	14.515	116.54	92.00	100.15
XXV_R070	70	5.362	P212121	1.275	7.448	16.107	20.080	90.00	90.00	90.00
XXV_R071	71	5.396	P21/c	1.272	10.725	8.326	28.404	90.00	107.81	90.00
XXV_R072	72	5.401	C2/c	1.205	19.401	6.622	40.015	90.00	97.48	90.00
XXV_R073	73	5.426	P-1	1.290	6.549	14.436	14.459	116.66	92.17	100.34
XXV_R074	74	5.446	P-1	1.268	8.687	11.337	13.622	101.68	101.81	106.28
XXV_R075	75	5.451	P21	1.259	10.816	8.065	14.673	90.00	107.64	90.00
XXV_R076	76	5.502	C2/c	1.281	17.052	8.335	33.831	90.00	93.97	90.00
XXV_R077	77	5.534	Pna21	1.253	27.985	10.719	8.171	90.00	90.00	90.00
XXV_R078	78	5.546	C2/c	1.281	27.453	8.879	20.094	90.00	101.70	90.00
XXV_R079	79	5.567	P21/c	1.274	12.624	10.805	17.686	90.00	91.36	90.00
XXV_R080	80	5.617	P21/c	1.227	9.393	21.470	15.514	90.00	126.86	90.00
XXV_R081	81	5.774	P-1	1.284	6.488	14.449	14.586	63.62	78.76	89.78
XXV_R082	82	6.004	P-1	1.227	7.879	10.998	15.148	100.49	90.54	103.79
XXV_R083	83	6.020	P21/c	1.218	13.712	7.402	27.372	90.00	114.78	90.00
XXV_R084	84	6.027	P-1	1.285	6.513	14.403	14.606	63.41	78.54	89.89
XXV_R085	85	6.029	P21/c	1.285	24.216	6.295	15.925	90.00	100.03	90.00
XXV_R086	86	6.034	P21/c	1.274	11.509	14.794	15.153	90.00	110.83	90.00
XXV_R087	87	6.042	P21/c	1.243	11.095	15.161	15.203	90.00	104.94	90.00
XXV_R088	88	6.154	P21/c	1.180	8.182	21.348	14.940	90.00	94.17	90.00
XXV_R089	89	6.176	Pbcn	1.253	28.797	10.802	15.758	90.00	90.00	90.00
XXV_R090	90	6.190	P-1	1.269	8.199	10.911	14.272	106.88	96.62	92.22
XXV_R091	91	6.370	P21/c	1.272	15.656	6.318	24.424	90.00	91.95	90.00
XXV_R092	92	6.490	P-1	1.278	6.772	12.569	14.641	95.43	103.24	94.03
XXV_R093	93	6.544	Pbca	1.210	14.562	16.794	20.767	90.00	90.00	90.00
XXV_R094	94	6.616	P21/c	1.283	6.477	8.222	45.081	90.00	94.22	90.00
XXV_R095	95	6.682	C2/c	1.310	22.859	15.252	14.365	90.00	110.54	90.00
XXV_R096	96	6.687	C2/c	1.264	30.362	6.347	29.080	90.00	119.84	90.00
XXV_R097	97	6.769	C2/c	1.251	29.088	10.788	17.682	90.00	117.72	90.00
XXV_R098	98	6.798	C2/c	1.249	30.342	10.655	16.108	90.00	109.13	90.00
XXV_R099	99	6.815	P-1	1.279	8.347	9.972	15.329	103.32	102.96	93.82
XXV_R100	100	6.815	P21/c	1.285	6.370	8.378	44.948	90.00	95.00	90.00
XXV_R101	101	6.839	P21/c	1.231	6.679	20.576	18.158	90.00	90.57	90.00
XXV_R102	102	6.871	Pbca	1.270	8.732	21.598	25.644	90.00	90.00	90.00
XXV_R103	103	6.960	P21/c	1.211	7.827	30.592	10.670	90.00	96.83	90.00
XXV_R104	104	7.069	P-1	1.278	7.963	9.889	16.764	95.03	92.15	113.59

XXV_R105	105	7.073	P21/c	1.263	15.008	11.360	15.975	90.00	116.74	90.00
XXV_R106	106	7.094	P21/c	1.238	10.306	15.422	16.620	90.00	110.06	90.00
XXV_R107	107	7.148	P-1	1.279	7.996	8.634	19.662	81.82	81.39	63.92
XXV_R108	108	7.159	P212121	1.311	7.335	15.196	21.014	90.00	90.00	90.00
XXV_R109	109	7.215	P21/c	1.252	10.819	14.519	15.703	90.00	95.85	90.00
XXV_R110	110	7.268	P-1	1.241	7.004	12.431	14.752	84.02	76.37	84.48
XXV_R111	111	7.272	P21/c	1.240	10.378	6.267	39.401	90.00	104.79	90.00
XXV_R112	112	7.273	P21/c	1.244	7.949	10.561	29.652	90.00	97.27	90.00
XXV_R113	113	7.283	C2/c	1.203	26.685	10.749	17.998	90.00	98.34	90.00
XXV_R114	114	7.354	P212121	1.329	8.267	14.773	18.927	90.00	90.00	90.00
XXV_R115	115	7.364	P-1	1.253	6.652	11.723	16.299	80.08	81.72	80.41
XXV_R116	116	7.388	P-1	1.260	8.692	12.592	13.098	63.75	74.07	74.66
XXV_R117	117	7.419	C2/c	1.206	26.448	10.784	18.085	90.00	99.08	90.00
XXV_R118	118	7.485	P21/c	1.231	14.411	24.595	7.069	90.00	95.27	90.00
XXV_R119	119	7.589	P21	1.309	7.597	14.511	10.649	90.00	91.91	90.00
XXV_R120	120	7.608	P-1	1.245	7.838	10.410	15.685	80.18	78.12	86.71
XXV_R121	121	7.637	P21	1.304	7.620	14.539	10.641	90.00	91.92	90.00
XXV_R122	122	7.638	C2/c	1.268	18.041	8.707	31.237	90.00	99.23	90.00
XXV_R123	123	7.746	Pbca	1.247	10.295	16.910	28.303	90.00	90.00	90.00
XXV_R124	124	7.756	P-1	1.290	8.183	8.742	16.829	90.04	93.20	97.99
XXV_R125	125	7.816	P21/c	1.227	7.554	16.263	20.379	90.00	90.24	90.00
XXV_R126	126	7.882	P21/c	1.265	7.999	16.369	19.984	90.00	111.91	90.00
XXV_R127	127	7.904	P21/c	1.231	8.151	16.792	19.879	90.00	113.50	90.00
XXV_R128	128	8.031	P21/c	1.278	8.112	15.237	19.739	90.00	99.85	90.00
XXV_R129	129	8.042	P-1	1.276	8.100	8.513	19.443	85.65	86.03	64.30
XXV_R130	130	8.131	P21/c	1.273	7.746	17.577	19.181	90.00	112.50	90.00
XXV_R131	131	8.134	P-1	1.244	8.850	10.755	14.480	70.21	75.48	75.61
XXV_R132	132	8.150	P21/c	1.246	7.957	21.573	14.388	90.00	93.54	90.00
XXV_R133	133	8.175	P-1	1.260	7.931	8.711	19.726	84.72	87.13	63.90
XXV_R134	134	8.180	Pbca	1.225	24.921	6.991	28.777	90.00	90.00	90.00
XXV_R135	135	8.208	P21/c	1.243	7.801	19.672	17.724	90.00	114.74	90.00
XXV_R136	136	8.208	Pbca	1.262	15.836	14.482	21.231	90.00	90.00	90.00
XXV_R137	137	8.214	P21/c	1.276	8.207	15.338	19.289	90.00	97.39	90.00
XXV_R138	138	8.227	P-1	1.263	6.429	10.249	19.373	75.43	88.09	79.86
XXV_R139	139	8.232	P21/c	1.271	8.095	38.636	8.588	90.00	115.83	90.00
XXV_R140	140	8.276	P21/c	1.229	7.888	20.018	17.674	90.00	116.44	90.00
XXV_R141	141	8.296	P-1	1.278	10.032	10.637	12.969	90.20	112.48	108.36
XXV_R142	142	8.333	P21/c	1.252	7.576	18.088	18.828	90.00	108.02	90.00
XXV_R143	143	8.355	P-1	1.275	7.809	8.277	20.668	89.99	81.80	65.92
XXV_R144	144	8.355	C2/c	1.293	15.158	7.459	42.033	90.00	91.30	90.00
XXV_R145	145	8.369	P-1	1.244	7.780	10.419	15.688	82.72	78.47	85.94
XXV_R146	146	8.388	Pbca	1.251	10.153	18.641	25.950	90.00	90.00	90.00
XXV_R147	147	8.469	P21	1.227	11.960	6.626	15.910	90.00	97.04	90.00
XXV_R148	148	8.471	P-1	1.275	8.261	8.354	17.736	92.26	93.34	99.16
XXV_R149	149	8.539	P-1	1.267	8.323	9.244	17.370	89.03	83.13	66.10
XXV_R150	150	8.562	P21/c	1.248	7.641	17.845	18.920	90.00	107.38	90.00
XXV_R151	151	8.576	P21/c	1.258	7.801	16.150	19.613	90.00	98.77	90.00
XXV_R152	152	8.578	Pbca	1.262	9.750	21.458	23.261	90.00	90.00	90.00
XXV_R153	153	8.618	P-1	1.291	8.208	8.309	18.598	93.57	94.08	109.27
XXV_R154	154	8.621	Pca21	1.292	8.853	21.625	12.423	90.00	90.00	90.00
XXV_R155	155	8.658	P21/c	1.229	7.536	20.754	16.308	90.00	101.40	90.00
XXV_R156	156	8.673	P21/c	1.231	6.833	21.729	16.820	90.00	91.84	90.00
XXV_R157	157	8.804	C2/c	1.260	30.461	11.070	16.489	90.00	118.72	90.00
XXV_R158	158	8.830	P21/c	1.264	7.855	17.609	19.169	90.00	113.62	90.00

XXV_R159	159	8.836	C2/c	1.220	29.412	7.062	24.264	90.00	91.85	90.00
XXV_R160	160	8.847	P-1	1.229	8.187	10.037	15.916	74.33	83.12	86.15
XXV_R161	161	8.897	P21/c	1.225	6.871	22.005	16.596	90.00	91.67	90.00
XXV_R162	162	8.922	P-1	1.291	7.580	8.135	21.264	86.34	84.75	65.73
XXV_R163	163	8.951	P-1	1.257	7.827	8.206	21.175	81.71	80.18	66.16
XXV_R164	164	8.955	P21/c	1.242	11.876	15.125	15.343	90.00	116.20	90.00
XXV_R165	165	9.050	P-1	1.253	9.452	9.919	13.216	87.59	82.08	88.29
XXV_R166	166	9.167	Pca21	1.283	8.836	21.856	12.402	90.00	90.00	90.00
XXV_R167	167	9.175	P21/c	1.269	7.684	15.277	20.631	90.00	92.29	90.00
XXV_R168	168	9.190	P-1	1.273	8.213	11.040	14.569	69.85	83.90	76.79
XXV_R169	169	9.195	P-1	1.266	8.832	10.132	13.814	93.86	97.36	96.59
XXV_R170	170	9.232	P21/c	1.235	10.458	25.287	9.809	90.00	106.48	90.00
XXV_R171	171	9.252	P21/c	1.234	7.984	21.612	14.447	90.00	93.00	90.00
XXV_R172	172	9.318	P-1	1.263	9.005	9.908	13.873	91.31	98.63	96.24
XXV_R173	173	9.444	Pbcn	1.208	28.461	10.066	17.756	90.00	90.00	90.00
XXV_R174	174	9.458	P21/c	1.266	7.706	41.769	8.787	90.00	120.96	90.00
XXV_R175	175	9.462	P-1	1.262	7.797	10.927	15.038	75.44	79.50	84.02
XXV_R176	176	9.562	P-1	1.221	8.901	10.213	15.516	98.69	104.80	107.72
XXV_R177	177	9.628	P21/c	1.266	8.162	14.708	20.259	90.00	93.82	90.00
XXV_R178	178	9.723	C2/c	1.261	14.254	8.805	39.166	90.00	97.62	90.00
XXV_R179	179	9.862	P212121	1.246	9.060	13.237	20.549	90.00	90.00	90.00
XXV_R180	180	10.060	P212121	1.278	10.704	12.862	17.455	90.00	90.00	90.00
XXV_R181	181	10.128	P21/c	1.256	8.393	8.769	33.233	90.00	90.96	90.00
XXV_R182	182	10.138	P21/c	1.201	19.856	7.125	18.199	90.00	96.61	90.00
XXV_R183	183	10.149	P212121	1.215	6.141	10.735	38.355	90.00	90.00	90.00
XXV_R184	184	10.161	C2/c	1.264	46.262	8.001	13.141	90.00	91.63	90.00
XXV_R185	185	10.464	Pna21	1.279	17.312	7.844	17.687	90.00	90.00	90.00
XXV_R186	186	10.538	P-1	1.245	7.852	8.357	21.068	79.00	86.37	65.36
XXV_R187	187	10.562	P21/c	1.297	14.567	9.080	18.481	90.00	104.37	90.00
XXV_R188	188	10.606	Pbca	1.287	13.379	15.491	23.034	90.00	90.00	90.00
XXV_R189	189	10.628	P-1	1.254	9.062	10.679	13.470	71.95	89.99	81.53
XXV_R190	190	10.783	C2/c	1.259	8.829	13.268	41.771	90.00	94.06	90.00
XXV_R191	191	10.798	P-1	1.296	9.417	9.755	13.164	89.87	78.84	87.19
XXV_R192	192	10.831	Pbca	1.261	8.812	12.621	43.822	90.00	90.00	90.00
XXV_R193	193	10.943	P21/c	1.252	8.153	38.338	8.917	90.00	118.29	90.00
XXV_R194	194	10.958	P-1	1.261	7.910	8.750	18.609	98.46	95.62	105.01
XXV_R195	195	11.031	P-1	1.236	6.315	8.835	22.559	90.82	92.11	98.89
XXV_R196	196	11.129	P21/c	1.258	8.216	37.370	9.129	90.00	119.37	90.00
XXV_R197	197	11.160	P21/c	1.225	8.601	29.973	9.936	90.00	101.84	90.00
XXV_R198	198	11.276	P21/c	1.260	9.003	21.263	15.249	90.00	123.35	90.00
XXV_R199	199	11.464	C2/c	1.241	18.575	9.713	28.592	90.00	106.32	90.00
XXV_R200	200	12.059	P-1	1.217	8.559	10.068	15.640	73.47	85.10	77.65
