# Supplementary material for: Report on the sixth blind test of organic crystal structure prediction methods
Source: Acta Crystallogr B Struct Sci Cryst Eng Mater. 2016 Aug 1;72(Pt 4):439–59. doi: 10.1107/S2052520616007447 (PMC4971545; doi:10.1107/S2052520616007447)
Supplement: Supplementary file 3 [file b-72-00439-sup3.zip › Group_10_Hofmann_Post_Analysis.pdf]

FlexCryst: Further Development of the Program and Methodology

Methodology

The method employed involves six general steps:

(i) building a 3D molecular structure from the supplied 2D chemical diagrams;

(ii) searching for plausible molecular conformations;

(iii) searching for plausible crystal packing arrangements of the molecule;

(iv) ranking the generated crystal structures with the scoring function, obtained with Data Mining;

(v) comparison of the ranked crystal structures and elimination of the manifold structures (List: Hofmann-Kuleshova);

(vi) re-ranking of the obtained crystal structures with DFT in order of likelihood of formation (List Hofmann-Boese)

Method of generating the 3D molecular structures

The 2D chemical diagrams have been build up as 3D molecular structures with a 3D structure generator with Materials Studio of Accelrys.

Method of generating molecular conformations

The 3D molecular structures were analyzed by a systematic grid search for possible conformations with Materials Studio of Accelrys.

Generating trial crystal structures

The unit-cell parameters, the positions, the orientations, and the conformations of the molecules were generated randomly for any molecule of the Sixth Blind Test with FlexCryst. During the generation of the crystal structure the space-group symmetry was used. The search has been performed for the ten most common space groups, which cover around 88 % of the known crystal structures and have been performed separately for Z′=1 and Z′=2.

What is new?

►*FlexCryst allows to choose for the prediction among the 79 most common space groups.*

►*FlexCryst allows to calculate till 40 molecules in the unit cell and respectively 10 molecules in the asymmetric unit for s. group $P2_1/c$ and 20 molecule for s. group P-1.*

Ranking of crystal structures with the scoring function

The ranking of the generated crystal structures is based on the calculated lattice energies with a Data Mining Force Field. The main idea of approach (Apostolakis 2001, Hofmann 2010) is to derive the force field parameters with the Data Mining (Big Data Analysis), using the Cambridge

Structure Database (data set of 686 944 crystal structures at the beginning of 2015) and computational facilities (137 Tflops) of CRS4. The combination of both should allow to predict the crystal structure of molecules and their free energy and as consequence the relative stability of polymorphs and cocrystals, the solubility, the melting point, etc. The typical methods of Data Mining, which were used: Anomaly detection , Association rule learning, Clustering , Classification, Regression, Summation , Sequential pattern mining .

What is new?

► *For the Sixth Blind Test the procedure of deriving scoring function has been modified to allow processing of bigger data sets and elaboration of considerable diversity of atom types. The details of the new procedure will be described in the new edition of International Tables for Crystallography, volume C, which is planed to be published in 2016.*

►*The new procedure did allow to split the standard atom types (coincided with the chemical symbol) to a number of subtypes, taking into account the first coordination sphere and hybrid orbitals of atoms, charged species and hydrogen bonds.*

► *FlexCryst allows to calculate the scoring functions as for full data set of CSD, so for specific sets of current classes of chemical substances, assigning the desired atom types in automatic way.*

Elimination of manifold structures

The multiple structures have been eliminated with *FlexCryst*. The random generation of crystal structures causes the multiple generation of several crystal structures. Since several thousands of crystal structures are generated, it is necessary to have a quick method to compare the structures. For this purpose we use the simulated powder diagram (Hofmann 2005). The number of obtained twins give us the coverage of the energetic landscape (Hofmann 2009). After the elimination of the multiple structures the remaining structures are summarized in our list "Hofmann-Kuleshova" and have been used for further processing.

Re-ranking of the obtained crystal structures with DFT

For the final ranking, we employed the Vienna *ab initio* Simulation Package (VASP) using density functional theory plus dispersion corrections. Based on our experience, PBE plus Tkachenko-Scheffler (TS) dispersion (Bucko 2013, Tkachenko 2009) yields the best error cancellation for organic solids (Boese 2013, Boese 2015/1), whereas for more ionic systems with hydrogen bonds, BLYP with Grimme (D3) dispersion (Grimme 2010) is giving a better performance (Boese 2015/2). Overall, we geometry-optimized the best structures of about six snapshots and looked for the thermodynamically most stable conformations. Here, hard pseudo-potentials have been used with a 900 eV cutoff. The zero-point energies have been calculated using finite differences using the same methodology.

Analysis of Our Results for the 6th Blind Test

An overview of our results is presented in the table. In the green color we highlighted the efficient features of approach, in the red – aspects which is still failed.

Table 1. Overall results of predictions

molecule	*rms* of best conformation	Space group	Z'	E_min	E_100	E_exp	DFT_exp – DFT_min [KJ]
XXII	0.45	P21/c	1	-803	-776	-787	-0.90
XXIII(a)	2.015	P21/c	1	-972	-913	-1041	0.73
XXIII(b)	2.205	P-1	1	-972	-913	-1046	-3.77
XXIII(c)	2.021, ?	P-1	2	-972	-913	-1059	-0.01
XXIII(d)	2.233	P21/c	1	-972	-913	-1016	3.81
XXIII(e)	2.130, ?	P-1	2	-972	-913	-1064	2.21
XXIV	0.086	P21/c	1	under development			
XXV	0.093, 0.094	P21/c	1	-904	-869	-824	-20.55
XXVI	0.843	P-1	1	-1146	-1079	-1225	-16.40

In the past our predictions failed mainly for the space group, Z', and the scoring function. As one can see these problems are solved. The recent innovations, implemented to *FlexCryst*, improve significantly the predictability of approach. Presently the largest problem is the prediction of reasonable molecular conformation: *ab initio* calculations of isolated molecule in gas phase do not give the satisfactory results for flexible molecule in crystal environment. The usage of the wrong initial conformation was the reason of failed prediction for molecules XXII, XXIII, XXVI, even if more accurate scoring function has shown good performance.

Molecule XXII. The conformation analysis for this molecule resulted in a single flat conformation. This conformation differs from the experimental bent structure by 0.45 A in the rms. Nevertheless the scoring function still was able to reproduce several crystal structures with the correct molecular layer. However the distorted conformation didn't allow to predict correctly the stacking of the layers.

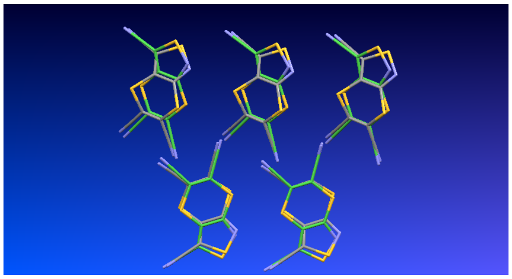

The post-refinement with DFT also was not able to correct this stacking, since the two structures belong to different space groups ($Pna2_1$ vs. $P2_1/c$).

The post-prediction performed with correct conformation resulted in experimental crystal structure on the rank 34.

Molecule XXIII. The used molecular conformation was completely wrong. Calculations in gas phase gave us bending conformation as the most stable, while in all experimentally observed conformations the molecule was extended. So it had no sense further to analyze the results of predictions for relative stability. Nevertheless the post-analysis of the energy function shows that the experimental structures lower in energy and the ranking of the different polymorphs coincide with the ranking of the other groups with the only inverted stability for *b* and *c* polymorphs.

Table 2. The relative stability of XXIII polymorphs

team	member	a	b	c	d	e
14	Neuman-Lleusen-Kendric, 2	5	1	2	3	-
14	Neuman-Lleusen-Kendric, 1	3,5	1	2	-	-
18	Price, 1	-	1	-	3,5	-
18	Price, 2	-	1	-	3,5	-
5	Van Eijck	3,5	1	-		-
3	Day	3	-	-	5	-
10	Hofman-Kuleshova	3	2	1	5	0
22	Boese (Hofmann)	3	1	2	5	4

The details of the analysis of relative stability, obtained with different DFT functionals one can fined in SI by A. Boese.

Molecule XXIV. Materials Studio did indicate two conformations. One of them (*Z*), used for prediction, was nearly identical with the experimental (*rms*=0.086). *FlexCryst* didn't have problems to generate the crystal structures with the three independent units in the asymmetric unit cell. The problems has been detected for the potential function for water molecule, which causes in some cases the generation of too dense crystal structures. We still are working on the problem The inaccurate scoring function for water prevented the prediction of exact crystal structure, even if the basic structural motive and hydrogen bonded system have been reproduced in several ranks of both as predicted, so DFT- optimized crystal structures. As the basic structural fragment we consider the molecular stacks of dimers $R^2_2(16)$, surrounded with the Cl^- ions and H_2O species. On the figure we show the overlay (*rms* 0.362) of hydrogen-bonded motives in experimental structure XXIV (gray) and rank 20 of our prediction (green):

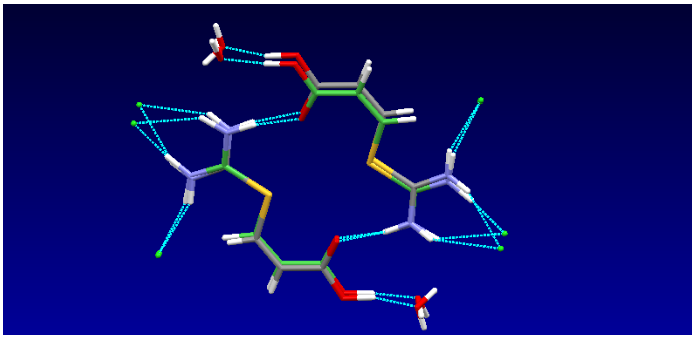

The difference arrives with the packing of this stacks between each other. In particular, we did not reproduce Cl^{-}- H_2O-Cl^{-} chain $C^{1}_{2}(4)$ correctly, which is caused by inaccuracy potential function for water.

The same tendency holds after DFT-optimization; see the comparing of molecular stacks in rank 13 (*rms* 0.476) with experimental structure XXIV:

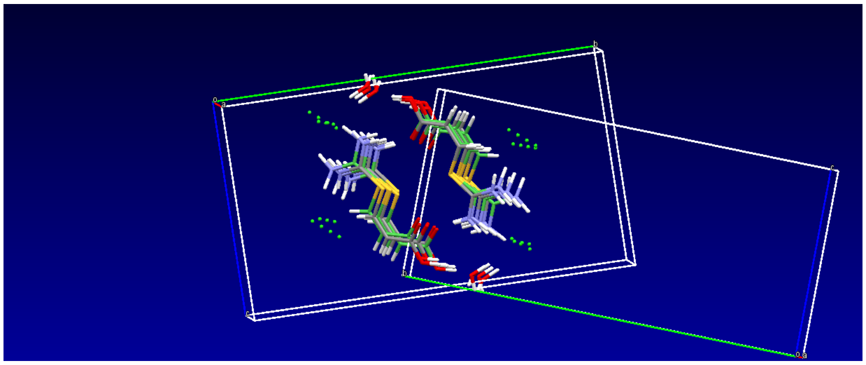

Molecule XXV. The conformation has been taken from the CSD and coincides perfectly with the experiment. However, this was the single case, where we did fail with our potential function (see table 1), and the main motive formed via O-H...N bridge was found only four times in Hofmann & Kuleshova submission (rank: 14, 55, 59, and 62). The DFT calculations recognize the unusual basicity of Trögers Base and rank this motive very high (Boese rank: 2,4, and 7).

During the discussion it did became clear, that in the experimental structure the position of hydrogen atom was disordered between two atoms: N...H...O and the molecule XXV should be considered as salt rather then a cocrystal. For our approach it makes a big difference, since in our force field we assign different atom types: in the salt the interaction is described as N4-HN...OC interaction, in the other case as N3...HO-O2 interaction. As one might see from the picture below the HN...OC interaction (red curve)) would be much stronger according our analysis as the N3...HO (blu curve).

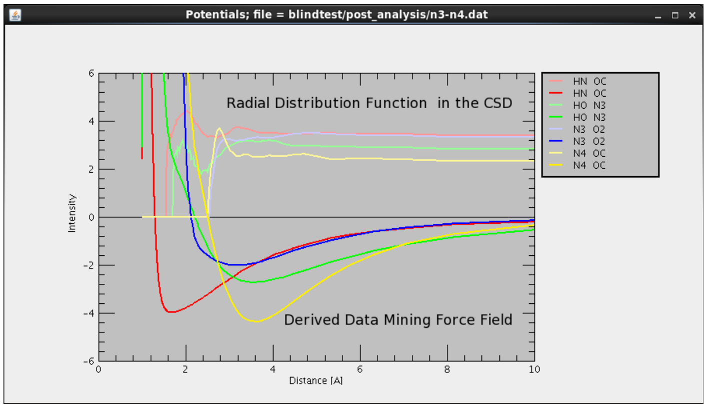

Molecule XXV. The molecule has seven degrees of freedom. We obtained five reasonable conformations within a range of 2 Kcal/mol. The most close to experimental conformation has been conformation rank 4 with a *rms* of 0.843 A. However, the difference is already too large and the first hundred structures do not contain an experimental structure.

The experimental conformation is clearly above our threshold, even if the total energy is below of the energy of the predicted crystal structures.

conformation	Conformational energy	Interaction energy	Total energy
1	77.6402	-1224.43	-1146.79
2	93.4713	-1214.45	-1120.98
3	86.4813	-1182.62	-1096.14
4	74.1744	-1180.22	-1106.04
5	96.7827	-1190.37	-1093.59
experimental	108.154	-1333.87	-1225.71

N.B: The mentioned innovation did not change the performance of the program. The prediction of a crystal structure needs with FlexCryst about 15 hours per structure on a conventional PC (2.0 GHz and one processor). The program has only a slight dependence on the size of the molecule (4 min/atom). For small molecules more neighbor cells have to be taken into account during the energy calculation, large molecules require less.

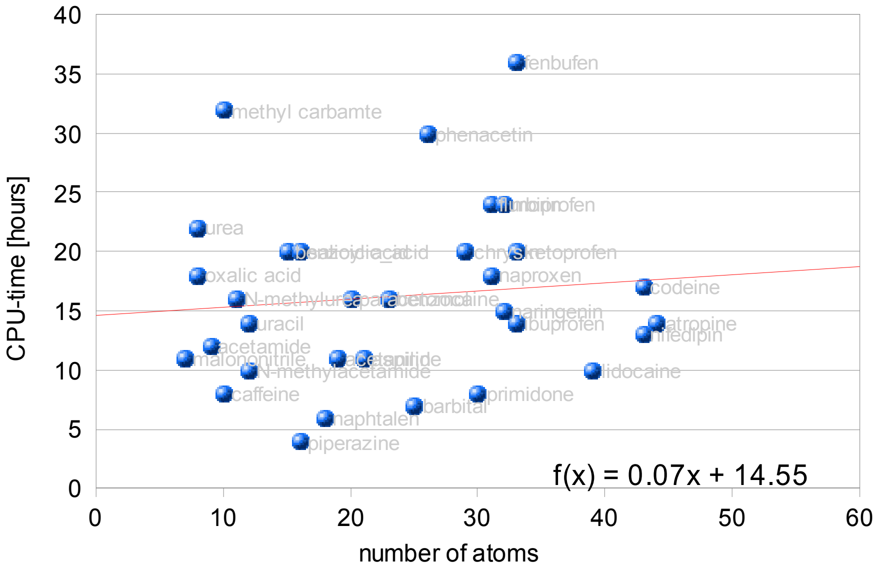

References

Apostolakis 2001: Apostolakis, J., Hofmann, D. W. M., & Lengauer, T. (2001). Derivation of a scoring function for crystal structure prediction. *Acta crystallographica. Section A, Foundations of crystallography*, *57*(Pt 4), 442-450.

Hofmann 2010: Hofmann, D. W. M. (2010). Data Mining in Organic Crystallography. In *Data Mining in Crystallography* (pp. 37-58). Springer Berlin Heidelberg.

Hofmann 2005: Hofmann, D. W. M., & Kuleshova, L. (2005). New similarity index for crystal structure determination from X-ray powder diagrams. *Journal of applied crystallography*, *38*(6), 861-866.

Hofmann 2009: Hofmann, D. W. M., Kuleshova, L. N., Hofmann, F., & D'Aguanno, B. (2009). Cluster analysis and completeness of crystal structure generation. *Chemical Physics Letters*, *475*(1), 149-155.

Bucko 2013: Bucko, T., Lebegue, S., Hafner, J., Angyan, J. G., Tkachenko-Scheffler van der Waals correction with and without self-consistent screening applied to solids. *Phys. Rev. B, 87 (6)*, 064110.

Tkachenko 2009: Tkachenko, A., Scheffler, M., Accurate Molecular van der Waals Interactions from Ground-State Electron Density and Free-Atom Reference Data. *Phys. Rev. Lett., 102 (7)*, 073005.

Boese 2013: Boese, A.D., Kirchner, M., Echeverria, G. A., Boese, R., *ChemPhysChem, 14 (4)*, 799-804.

Boese 2015/1: Boese, A. D., Boese, R., *Cryst. Growth & Design, 15 (3),* 1073-1081.

Grimme 2010: Grimme, S., Anthony, J., Ehrlich, S., Krieg, H., *J. Chem. Phys. 132 (15),* 154104.

Boese 2015/2: Boese, A. D., *ChemPhysChem, 16 (5),* 978-985.
