# Supplementary material for: Report on the sixth blind test of organic crystal structure prediction methods
Source: Acta Crystallogr B Struct Sci Cryst Eng Mater. 2016 Aug 1;72(Pt 4):439–59. doi: 10.1107/S2052520616007447 (PMC4971545; doi:10.1107/S2052520616007447)
Supplement: Supplementary file 3 [file b-72-00439-sup3.zip › Group_11_Lv_Wang_Ma.pdf]

Supporting information: structure prediction of molecular crystal made of molecules XXII through CALYPSO method

Jian Lv, Yanchao Wang and Yanming Ma*

State Key Laboratory of Superhard Materials, Jilin University, Changchun 130012, China.

Email : mym@jlu.edu.cn

We assumed that molecules XXII in the crystalline phase are rigid and the interactions between them are van der Waals. Thus, it is a constrained global optimization problem to search the crystalline structure of molecules XXII, where only intermolecular degrees of freedom need to be optimized.

Here, the constrained structure-searching simulations were performed using the CALYPSO method[1,2], which is based on a particle swarm optimization (PSO) algorithm via a global minimization of free-energy surfaces merging *ab initio* total energy calculations as implemented in the CALYPSO code[1,2]. The details of this method and its applications have been described elsewhere[3]. Currently, it has many attractive features including predictions of the energetically stable/metastable structures at given chemical compositions for isolated nanoparticles/clusters or molecules[4], 2-dimensional (2D) layers (single/multi layers and buckled layers)[5,6], 2D surfaces[7], and 3-dimensional crystals[2]; design of novel functional materials, e.g., superhard materials[8]; structure searching with automatic variation of chemical compositions; structure predictions with fixed unit cell parameters, or fixed spacegroups, or fixed molecules.

The atomic configuration of single molecule XXII, as depicted in Fig. 1, was locally optimized at PBEPBE/aug-cc-pvtz level of theory using Gaussian09[9]. Then, initial crystalline structures were generated through packing of this molecule according to constraints of space group symmetries using the CALYPSO module of structure prediction with fixed molecules. During structure generation, the only given information is the structure of molecule XXII and number of molecules (Z) in a unit cell, while the space group symmetries are selected randomly. Given that molecule XXII possesses a point group of C_1 and the target experimental organic structure contains one molecule in an asymmetric unit (Z'=1), thus only one symmetry-inequivalent molecule XXII was used to generate the initial structures. This means that Z of an initial structure is equal to the number of symmetry operations of the particular space group.

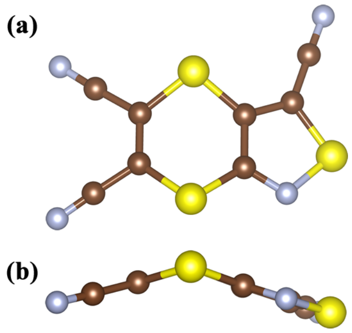

Fig. 1. Two views of molecule XXII optimized at PBEPBE/aug-cc-pvtz level of theory: (a) top view and (b) side view.

In a general case, the initial structures generated by CALYPSO will be subject to local optimizations, from which the resulting structures and energies of corresponding local minima will be

acquired and used to generate new structures through the PSO algorithm[1,2]. Structure evolutions through PSO algorithm will keep Z fixed but change the space group symmetries of parent structures. This typically breaks the asymmetric unit of molecule XXII in the crystal, violating the target experimental structure. Thus structure evolution in CALYPSO is not used. Rather, the strategy used here is similar to random sampling method[10] as implemented in CALYPSO code for generation of initial structures. Note that if Z' is not already known, structure evolution through CALYPSO method must be used.

In our structure searching simulations, structural relaxations and energy calculations were performed in the framework of DFT within the Perdew–Burke–Ernzerhof exchange correlations[11] as implemented in the VASP code[12]. The electron–ion interaction was described by means of a projector augmented wave[13] with s^2p^2, s^2p^3 and s^2p^4 electrons as valence for C, N and S atoms, respectively. Long-range van der Waals interactions have been taken into account via the optB86b-vdW functional[14,15]. Coarse structural relaxations of initially generated structures utilize a plane-wave basis set with an energy cutoff of 400 eV and 0.6 $Å^{-1}$ grid spacing of k-point sampling. Low-energy structures were further re-optimized using a plane-wave basis set with an energy cutoff of 600 eV and 0.4 $Å^{-1}$ grid spacing of k-point sampling. Finally, structures are ranked according to their lattice energies. The top 100 structures with lower energies are given in list 1, and the other 100 structures with higher energies are given in list 2. By the time of deadline, the number of local optimizations and approximate computer resources used are surmised in Table I.

Table I. Summary of structure searches: number of local optimizations and resources used.

Coarse calculation			
	Z=1	Z=2	Z=4
Number of local optimizations	400	3000	3000
Approximately normalized to 3.0 GHz CPU Core hours	7193	98194	269675
Refinement			
Number of local optimizations	200	300	300
Approximately normalized to 3.0 GHz CPU Core hours	8276	20565	55548

References:

[1] Y. Wang, J. Lv, L. Zhu, and Y. Ma, Comput. Phys. Commun. **183**, 2063 (2012).

[2] Y. Wang, J. Lv, L. Zhu, and Y. Ma, Phys. Rev. B **82**, 094116 (2010).

[3] Y. Wang, J. Lv, L. Zhu, S. Lu, K. Yin, Q. Li, H. Wang, L. Zhang, and Y. Ma, J. Phys.: Condens. Matter 1 (2015).

[4] J. Lv, Y. Wang, L. Zhu, and Y. Ma, J. Chem. Phys. **137**, 084104 (2012).

[5] Y. Wang, M. Miao, J. Lv, L. Zhu, K. Yin, H. Liu, and Y. Ma, J. Chem. Phys. **137**, 224108 (2012).

[6] X. Luo, J. Yang, H. Liu, X. Wu, Y. Wang, Y. Ma, S.-H. Wei, X. Gong, and H. Xiang, J. Am. Chem. Soc. **133**, 16285 (2011).

[7] S. Lu, Y. Wang, H. Liu, M.-S. Miao, and Y. Ma, Nat. Commun. **5**, 1 (2014).

[8] X. Zhang, Y. Wang, J. Lv, C. Zhu, Q. Li, M. Zhang, Q. Li, and Y. Ma, J. Chem. Phys. **138**, 114101 (2013).
[9] Frisch, M. J. et al. Gaussian 09, revision C.01; Gaussian, Inc.: Wallingford, CT, 2009.
[10] C. J. Pickard and R. J. Needs, J. Phys.: Condens. Matter **23**, 053201 (2011).
[11] J. P. Perdew, K. Burke, and M. Ernzerhof, Phys. Rev. Lett. **77**, 3865 (1996).
[12] G. Kresse and J. Furthmüller, Phys. Rev. B (1996).
[13] G. Kresse and D. Joubert, Phys. Rev. B **59**, 1758 (1999).
[14] J. Klimeš, D. R. Bowler, and A. Michaelides, J. Phys.: Condens. Matter **22**, 022201 (2009).
[15] M. Dion, H. Rydberg, E. Schröder, D. C. Langreth, and B. I. Lundqvist, Phys. Rev. Lett. **92**, 246401 (2004).
