# Supplementary material for: Report on the sixth blind test of organic crystal structure prediction methods
Source: Acta Crystallogr B Struct Sci Cryst Eng Mater. 2016 Aug 1;72(Pt 4):439–59. doi: 10.1107/S2052520616007447 (PMC4971545; doi:10.1107/S2052520616007447)
Supplement: Supplementary file 3 [file b-72-00439-sup3.zip › Group_12_Marom_Post_Analysis.pdf]

GAtor
Supplementary Information

Farren Curtis[1,2], Xiayue Li[1,5], Christoph Schober[6], Katherine Cosburn[1,7], Sanjaya Lohani[1], Francesca Vacarro[1,8], Harald Oberhofer[6], Karsten Reuter[6], Saswata Bhattacharya[4], Álvaro Vázquez-Mayagoitia[5], Luca M. Ghiringhellli[4], and Noa Marom[*1,3].

[1]*Department of Physics and Engineering Physics, Tulane University, New Orleans, Louisiana 70118, USA.*[2]*Department of Physics, Carnegie Mellon University, Pittsburgh, PA 15213, USA.* [3]*Department of Materials Science and Engineering and Department of Physics, Carnegie Mellon University, Pittsburgh, PA 15213, USA.* [4]*Fritz-Haber-Institut der Max-Planck-Gesellschaft, Faradayweg 4-6, 14195, Berlin, Germany.* [5]*Argonne Leadership Computing Facility, Argonne National Laboratory, Lemont, Illinois 60439, USA.* [6]*Chair for Theoretical Chemistry and Catalysis Research Center, Technische Universität München, Lichtenbergstr. 4, D-85747 Garching, Germany.* [7]*Department of Physics, University of Toronto, Toronto, M5S 1A7, Canada.* [8]*Department of Chemistry, Loyola University, New Orleans, Louisiana 70118, USA.*

1 Overview of Methodology

We developed a first-principles genetic algorithm (GA) for molecular crystal structure prediction, GAtor, which finds the most energetically stable crystal structures for rigid molecules, and applied it to target XXII. GAtor uses principles from evolutionary theory such as survival of the fittest, crossover, and mutation that are implemented as operators acting on individual molecules and/or lattice vectors of the fittest crystal structures selected for mating. Structural relaxations and energy evaluations are performed using dispersion-inclusive density functional theory (DFT). For this purpose, GAtor interfaces with the all-electron electronic structure package FHI-aims [1]. Massive parallelization is achieved by eliminating the concept of GA generations and running several replicas in parallel that read and write to a common pool of structures [2, 3, 4]. Crucial to the success of GAtor is the generation and ranking of an initial pool of structures that maintains diversity and explores the most physically-relevant and promising regions of the potential energy landscape. Below we provide brief descriptions of our initial pool generation as well as the implementation of the genetic algorithm itself. We also explain the methodology we used for post-processing, refinement, and re-ranking of the final structures produced by the genetic algorithm. It should be noted that our approach is a fully quantum mechanical first-principles approach that does not use force fields at any point.

Single Molecule Structure

Since target XXII can adopt different conformations (i.e. the six membered ring can be bent along the S-S axis, such that the CN groups may lie above or below the plane of the 5 membered ring) we analyzed the effect of the bending angle on the energy of the single molecule. First, we performed single-point calculations on a range of angles from 20° to 160° using the Perdew-Burke-Ernzerhof generalized gradient approximation (PBE)[5, 6] with the Tkatchenko-Scheffler (TS)

*Corresponding author: nmarom@andrew.cmu.edu

pairwise dispersion-correction, PBE+TS [7], which employs an inexpensive pairwise approach to account for the van der Waals (vdW) contribution to the energy. We obtained a symmetric double-well potential and fully relaxed the two stable conformations to obtain final bending angles of 155.8° and 204.2°, respectively. We used the rigid, single molecule geometries at these equilibirum angles for the initial pool generation.

Initial Pool Generation

Since target XXII possessed two enantiomers, we generated separate chiral and racemic initial pools to serve as inputs for our genetic algorithm. In total we generated four different pools— each having 2 or 4 molecules per unit cell that contained one or both conformers. For each of these pools, 50,000 structures were generated both in random unit cells (with no enforced symmetry between the molecules) as well as in the most likely space groups. The random, symmetric structures were generated in the $P2_1$ and $P2$ space groups for the Z=2 chiral pool, $P\bar{1}$, Pc, and Pm for the Z=2 racemic pool, $P2_12_12_1$, $P2_12_12$, and $C2$ for the Z=4 chiral pool, and $P2_1/c$, $Pca2_1$, and $Pna2_1$ for the Z=4 racemic pool. The volume range we used for generating the structures was 160-300 $Å^3$/molecule.

The four initial pools were independently ranked in energy using an implementation of the Harris approximation integrated with FHI-aims. Within the Harris approximation, the total density of a system is constructed by a superposition of fragment densities [8]. In this scenario, the DFT total energy can be evaluated for the Harris density without performing a self-consistent cycle, allowing almost instantaneous energy evaluations.

The Harris density of a molecular crystal is constructed by replicating, translating, and rotating a single molecule's density, which is calculated only once. The numerical atom-centered orbital (NAO) basis functions of FHI-aims are based on real-valued linear combinations of spherical harmonics [1]. Since the spherical harmonics are fixed with respect to the *xyz*-coordinate system, rotation of a molecule produces a new linear combination of basis functions. Modified Wigner matrices [9] are employed to obtain the rotated coefficients of each basis function.

A binding energy curve computed with PBE+TS and PBE+TS@Harris for a molecule similar to target XXII, tetracyano-1,4-dithiin, is shown in Fig.1. The curve was calculated along the direction of the closest C· · ·N contacts in the crystal [10] which are similar to the close contacts of target XXII. When the molecules are non-interacting at large distances the Harris approximation and the fully self-consistent method converge to the same result. As the molecules come closer together, the Harris approximation fails to account for the change in density due to the electrostatic interactions between the molecules and polarization effects. This produces a weaker binding energy than the fully self-consistent result. The difference between the self-consistent and Harris densities for the tetracyano-1,4-dithiin dimer is also shown in Fig. 1. The red regions around the N atoms indicate areas where the Harris density overestimates the self-consistent density while the blue regions around the S atoms indicate where the Harris approximation underestimates the self-consistent density. The self-consistent density is concentrated closer to the molecular framework because of Coulomb repulsion between nitrogen lone-pairs.

The Harris approximation allows GAtor to perform fast screening of initial structures using an unbiased first-principles approach without resorting to force fields, which can be difficult to parameterize for non-standard molecules. We used PBE+TS for performing the Harris approximation for target XXII. The parameters of the TS correction (i.e. the C_6 coefficients and the van der Waals radii) are calculated on the fly based on the DFT (or Harris) density. This makes the TS method more accurate than semi-empirical pairwise methods [11].

After the initial structures in each of the four pools were ranked with the Harris approximation, local geometry optimization was performed for the best 6%, using PBE+TS with *lower-level*

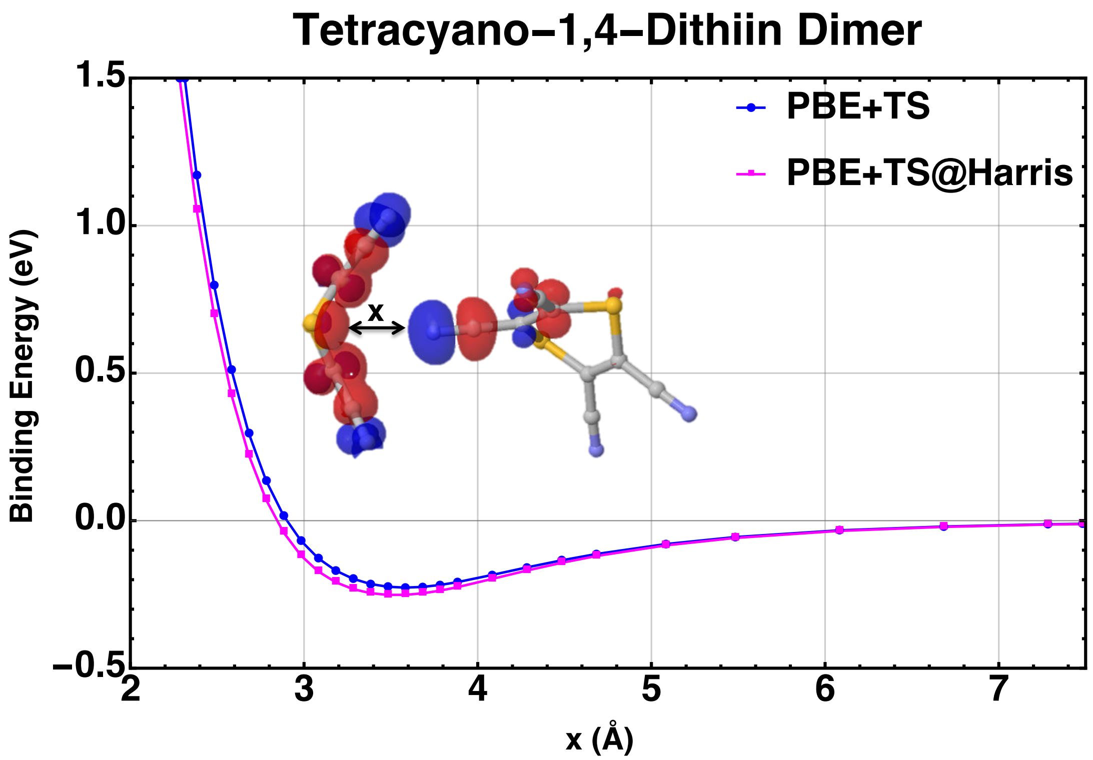

Figure 1: A binding energy curve for tetracyano-1,4-dithiin computed with PBE+TS and PBE+TS@Harris. An isosurface of the density difference between the self-consistent and Harris densities is shown for the equilibrium distance of $x = 3.48$ Å. The red regions indicate areas of a positive density difference while the blue areas indicate regions with a negetive density difference.

numerical settings which correspond to the light/tier1 settings of FHI-aims [1]. For the best 50% of this subset, full unit cell relaxations were performed using PBE+TS and *lower-level* numerical settings. During relaxation no constraints were imposed on the unit cell symmetry or the structure of the molecule (i.e. the central bending angle was allowed to vary for different structures). The fully relaxed structures served as the initial pool for the genetic algorithm.

The GAtor Genetic Algorithm

Similar to the genetic algorithms reported in [2, 3, 4, 12], the fitness f_i of each structure depends on its normalized relative energy given by:

$$f_i = \frac{\epsilon_i}{\sum_i \epsilon_i} \tag{1}$$

where ϵ_i is the relative energy of the ith structure and is given by:

$$\epsilon_i = \frac{E_{max} - E_i}{E_{max} - E_{min}}. \tag{2}$$

In Eq. 2, E_{max} is the current maximum energy in the pool and E_{min} is the minimum. Hence, the fitness for each individual is dynamically updated with each new addition to the common pool. Using a "roulette-wheel" selection criterion [13], structures with higher fitness values have a higher probability of selection. A small fitness reversal probability similar to [3] allows for the occasional selection of an unfit structure to avoid biasing the search towards pre-converging to local minima.

Crossover randomly combines the lattice vectors of each parent structure and randomly selects molecules from one or both parents. After crossover, child structures have a 15% chance of un-

dergoing mutation, which applies random displacements or rotations to the molecules in the child structure or random symmetric or asymmetric strains to the unit cell.

After the child structure has been formed, its single-point energy is computed using PBE+TS with *lower-level settings*. If the single-point energy of the trial structure is outside of a user-specified range from the current global minimum, it is immediately rejected. The structure is also rejected if it is found to be a duplicate of any other structure in the common pool. If the structure is found to be unique then it is passed on to full unit cell relaxation with *lower-level* settings and added to the common pool. GAtor stops when it can no longer find new low-energy structures. The top 10% of the most stable structures found by the GA are then selected for post-processing.

Post-Processing

After combining the top structures found in each of our searches, approximately 500 structures were re-relaxed and re-ranked using PBE+TS and *higher-level* numerical settings, which correspond to the tight/tier 2 settings of FHI-aims [1]. We then performed single-point energy evaluations using PBE with the many-body dispersion (MBD)[14, 15] method for 200 of the best structures as ranked by PBE+TS. The MBD method accounts for long-range electrostatic screening effects and for non-pairwise-additive many-body contributions to the dispersion energy. It has been shown to accurately rank the stability of molecular crystal polymorphs in cases where the pairwise TS approach is not sufficiently accurate [16, 17].

We also performed single-point energy evaluations for 150 of the best structures as ranked by PBE+MBD using the hybrid functional PBE0 [18, 19] with the MBD correction. The inclusion of 25% exact exchange in PBE0 mitigates the self-interaction error, leading to a more accurate description of electron densities and multipoles [17, 20, 21]. For some molecular crystals, such as glycine and oxalic acid, the correct polymorph ranking is reproduced only when using PBE0+MBD [16]. We therefore consider the ranking of PBE0+MBD to be the most reliable of the methods used here.

2 Results and Analysis

Our blind test submission included one list of the top 100 structures as ranked by PBE+TS and another as ranked by PBE+MBD. Since we performed single-point calculations on more than 100 structures from the GA, the two lists did not consist of a simple re-ranking of the exact same structures. The PBE0+MBD calculations were not completed in time for the submission deadline and the full PBE0+MBD list was appended after the submission.

The final top 10 structures as ranked by PBE+MBD and re-ranked by PBE+TS and PBE0+MBD are shown in Table 1 along with the experimental structure which was not found in our searches. The experimental structure would have been ranked in the top three by all three methods and as #1 by PBE0+MBD. Further analysis of the effect of the choice of DFT functional and dispersion method on the ranking of structures is provided below.

Target XXII crystallized in $P2_1/n$, a nonstandard spacegroup used for orthogonal representations of oblique $P2_1/c$ unit cells. We did not explicitly generate structures in $P2_1/n$ because the structures generated in $P2_1/c$ for the initial pool were constrained to have angles between 60 to 120 degrees. Although the GA in principle still could have found the target by various strain and/or rotation mutations, it was biased for selecting, crossing over, and propagating the traits (including the orientation of the structural motifs) of the best structures in the initial pool. Furthermore, our post-analysis revealed that the duplicate check tolerance throughout the GA was set too tight. This allowed some duplicate structures into the pool, leading to an artificial increase in the representation of orthogonal cells. Overall, generating intial pool structures explicitly in $P2_1/n$ would have greatly increased the likelihood of finding the experimental structure.

Name	PBE+MBD		PBE+TS		PBE0+MBD		Z	Space Group	a	b	c	α	β	γ
	Rank	ΔE(eV)	Rank	ΔE(eV)	Rank	ΔE(eV)								
7471226271	1	0.000	1	0.000	2	0.007	4	$Pna2_1$	13.4	10.2	7.1	90	90	90
9f774c9e27	2	0.005	2	0.018	1	0.000	4	$P2_1/c$	14.4	10.3	6.7	90	90	94
dab6897b90	3	0.021	3	0.028	3	0.033	4	$P2_12_12_1$	10.2	7.0	13.7	90	90	90
52cdef12ff	4	0.037	4	0.032	30	0.055	4	$P\bar{1}$	14.0	7.1	10.3	90	90	70
42a9600b47	5	0.038	79	0.077	4	0.036	4	$Pna2_1$	20.5	7.4	6.7	90	90	90
197ac7c454	6	0.040	94	0.081	11	0.045	4	$P2_1/c$	9.7	11.3	9.4	90	85	90
f191f2a68b	7	0.040	6	0.044	10	0.044	4	$Pnma$	20.7	7.1	6.6	90	90	90
585d18ed08	8	0.041	9	0.051	18	0.051	2	$P\bar{1}$	9.0	9.8	6.0	110	93	98
71fe1a6200	9	0.043	20	0.059	31	0.056	4	$P2_1$	10.2	7.3	13.5	90	90	83
a206286cd3	10	0.044	67	0.075	6	0.039	2	$P2_1$	10.3	7.5	6.6	90	90	88
Experimental	(3)	0.006	(2)	0.017	(1)	-0.005	4	$P2_1/n$	12.0	6.7	12.6	90	109	90

Table 1: The top 10 structures as ranked by PBE+MBD with the re-ranking of PBE+TS and PBE0+MBD. The experimental structure which was not found in our search is shown with its hypothetical ranking and relative energy to the respective global minimum of the submitted structures.

We did, however, generate several structures with similar binding motifs to the blind test target. Structure 9f774c9e27, in space group $P2_1/c$, is compared to the experimental structure of target XXII in Fig. 2. These structures are stabilized by similar intramolecular C$\cdots$N interactions.

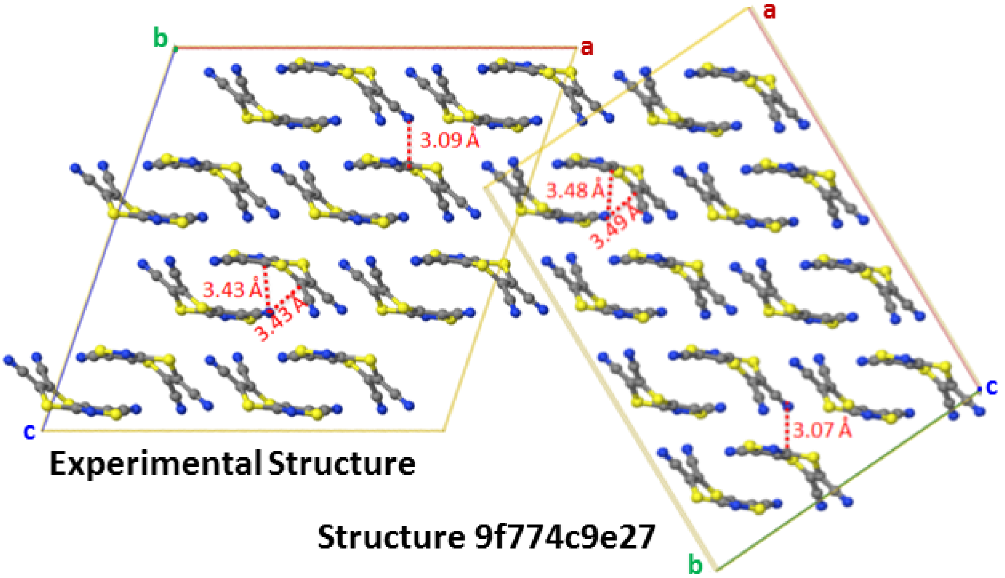

Figure 2: Motif comparisons between target XXII and our PBE+TS and PBE+MBD #2 structure. Distances of the closest intermolecular C$\cdots$N interactions are shown.

The energy differences between target XXII and 9f774c9e27 are extremely small, ranging from 1 meV to 5 meV depending on the method, as shown in Table 1. Furthermore, several other groups submitted this structure within their top 10 structures. Another structure, 7471226271, which was also in the top 2 for all ranking methods, was listed among the top structures of several other groups as well. Since structures 9f774c9e27, 7471226271, and the experimental structure are all extremely close in energy and were found by several other groups, we believe these three structures may be polymorphs.

The effect of the choice of DFT functional and dispersion method on the potential energy landscape and the ranking of structures is illustrated in Figs. 3 and 4. Graphs of the volume per

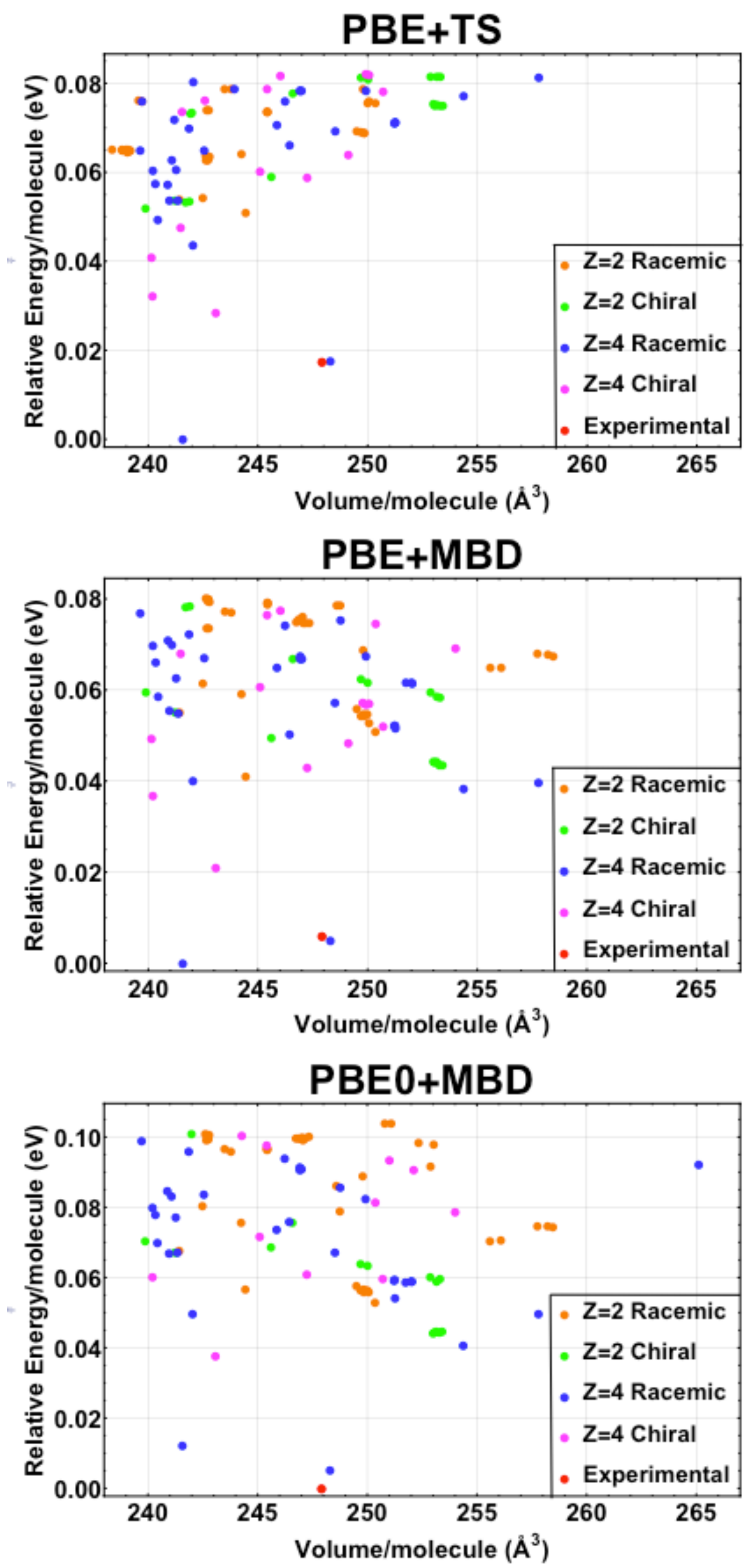

Figure 3: Volume per molecule versus energy plots for the top 100 structures as ranked by PBE+TS, PBE+MBD, and PBE0+MBD. The experimental structure is shown for reference.

molecule versus the energy per molecule for the best 100 structures from PBE+TS, PBE+MBD, and PBE0+MBD are shown in Fig. 3 along with the experimental structure. All of the top structures are extremely close in energy and fall within an interval of about 0.1 eV. The distribution of structures changes significantly depending on the method used. The TS method, which tends to overbind, favors structures with smaller specific volumes than the MBD method. The PBE0 functional increases the energy differences between structures and further stabilizes structures with lower densities as compared to PBE.

Fig. 4 shows the ranking of the top five PBE0+MBD structures for each method used. There is significant rearrangement between methods for most of the structures except for the top 4. Some structures, such as 42a9600b47 and d037fff743, have higher relative energies with PBE+TS but are stabilized dramatically by PBE+MBD and PBE0+MBD. The two best structures as ranked by PBE+TS (9f774c9e27 and 7471226271) become even closer in energy when computed with PBE+MBD but swap rankings with PBE0+MBD. The experimental structure is stabilized by both MBD and PBE0 and would have been ranked as number one with PBE0+MBD.

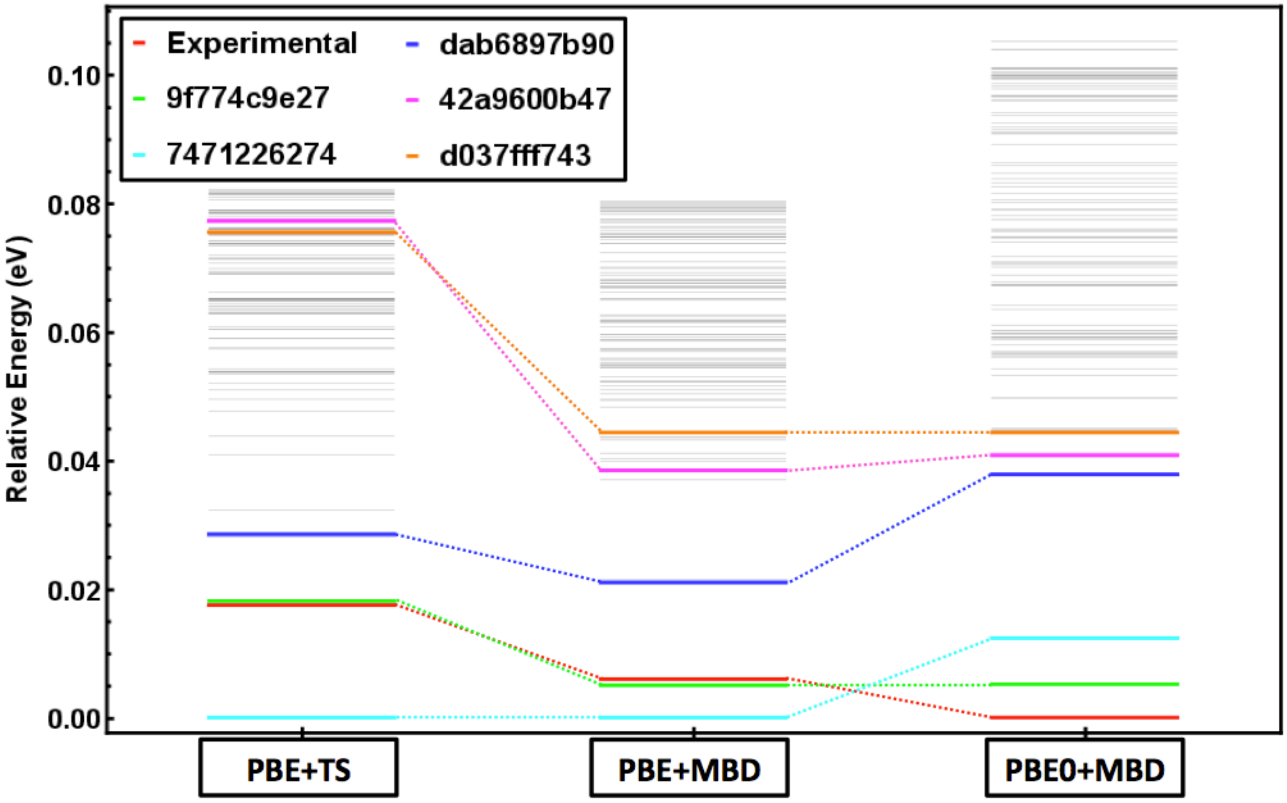

Figure 4: The top 5 PBE0+MBD structures along with the experimental structure as ranked by the different methods. All other structures in the top 100 are shown in gray.

Computational Time

We used approximately 30M CPU hours. Most calculations were done on Mira at the Argonne Leadership Computing Facility (ALCF) which is an IBM Blue Gene/Q, with 16-core 1.6 GHz PowerPC processors. Some additional calculations were performed on Tulane University's Intel Xeon E5-2680 cluster, Cypress, which has dual 10-core 2.8 GHz processors. Most of our CPU time was spent doing full unit cell relaxations with a fully quantum mechanical first-principles approach for approximately 10,000 structures within the GA itself. To the best of our knowledge, we were

the only group in the blind test to use an entirely DFT-based approach which also contributed to a much higher computational cost than if we had used force fields or other semi-empirical methods.

Acknowledgments

We thank Leslie Leiserowitz from the Weizmann Institute of Science and Geoffrey Hutchinson from the University of Pittsburgh for helpful discussions. We thank Adam Scovel at the Argonne Leadership Computing Facility (ALCF) for technical support. Work at Tulane University was funded by the Louisiana Board of Regents Award # LEQSF(2014-17)-RD-A-10 "Toward Crystal Engineering from First Principles", by the NSF award #EPS-1003897 "The Louisiana Alliance for Simulation-Guided Materials Applications (LA-SiGMA)", and by the Tulane Committee on Research Summer Fellowship. Work at the Technical University of Munich was supported by the Solar Technologies Go Hybrid initiative of the State of Bavaria, Germany. Computer time was provided by the Argonne Leadership Computing Facility (ALCF), which is supported by the Office of Science of the U.S. Department of Energy under contract DE-AC02-06CH11357.

References

[1] V. Blum, R. Gehrke, F. Hanke, P. Havu, V. Havu, X. Ren, K. Reuter, and M. Scheffler, Comput. Phys. Commun. **180**, 2175 (2009).

[2] S. Bhattacharya, S. V. Levchenko, L. M. Ghiringhelli, and M. Scheffler, Phys. Rev. Lett. **111**, 135501 (2013).

[3] S. Bhattacharya, S. V Levchenko, L. M. Ghiringhelli, and M. Scheffler, New J. Phys. **16**, 123016 (2014).

[4] S. Bhattacharya, B. H. Sonin, C. J. Jumonville, L. M. Ghiringhelli, and N. Marom, Phys. Rev. B **91**, 241115 (2015).

[5] J. P. Perdew, K. Burke, M. Ernzerhof, Phys. Rev. Lett. **77**, 3865 (1996).

[6] J. P. Perdew, K. Burke, M. Ernzerhof, Phys. Rev. Lett. **78**, 1396 (1997).

[7] A. Tkatchenko and M. Scheffler, Phys. Rev. Lett. **102**, 073005 (2009).

[8] K. Berland, E. Londero, E. Schröder, and P. Hyldgaard, Phys. Rev. B. **88**, 045431 (2013).

[9] M. A. Blanco , M. Flórez, M. Bermejo. J. Mol. Struct. **419**, 1927 (1997).

[10] W. A. Dollase. J. Am. Chem. Soc. **87**, 5 (1965).

[11] N. Marom, A. Tkatchenko, M. Rossi, V. V. Gobre, O. Hod, M. Scheffler, and L. Kronik, J. Chem. Theory Comput. **7**, 3944 (2011).

[12] D. C. Lonie and E. Zurek, Comput. Phys. Commun. **182**, 372 (2011).

[13] D. E. Goldberg, *Genetic Algorithms in Search, Optimization and Machine Learning* (Addison-Wesley, MA, 1989).

[14] A. Tkatchenko, R. A. Distasio, R. Car, and M. Scheffler, Phys. Rev. Lett. **108**, 236402 (2012).

[15] A. Ambrosetti, A. M. Reilly, R. A. Distasio, and A. Tkatchenko, J. Chem. Phys. **140**, 18A508 (2014).

[16] N. Marom, R. A. Distasio, V. Atalla, S. Levchenko, A. M. Reilly, J. R. Chelikowsky, L. Leiserowitz, and A. Tkatchenko, Angew. Chemie - Int. Ed. **52**, 6629 (2013).

[17] A. M. Reilly and A. Tkatchenko, J. Phys. Chem. Lett. **4**, 1028 (2013).

[18] J. P. Perdew, M. Ernzerhof, and K. Burke, J. Chem. Phys. **105**, 9982 (1996).

[19] C. Adamo and V. Barone, J. Chem. Phys. **110**, 6158 (1999).

[20] B. Santra, J. Klimeš, D. Alfé, A. Tkatchenko, B. Slater, A. Michaelides, R. Car, and M. Scheffler, Phys. Rev. Lett. **107**, 185701 (2011).

[21] A. M. Reilly and A. Tkatchenko, J. Chem. Phys. **139**, 024705 (2013).
