# Supplementary material for: Report on the sixth blind test of organic crystal structure prediction methods
Source: Acta Crystallogr B Struct Sci Cryst Eng Mater. 2016 Aug 1;72(Pt 4):439–59. doi: 10.1107/S2052520616007447 (PMC4971545; doi:10.1107/S2052520616007447)
Supplement: Supplementary file 3 [file b-72-00439-sup3.zip › Group_13_Mohamed_Post_Analysis.pdf]

Supplementary Material

Dr. Sharmarke Mohamed

Department of Applied Mathematics and Sciences

Khalifa University, Abu Dhabi, UAE

sharmarke.mohamed@kustar.ac.ae

1 Computational Methodology

1.1 Overview

For all systems attempted in this study, hypothetical crystal structures were generated using the Polymorph Predictor module of *Materials Studio 7*.0 (Accelrys, 2013), which uses a Monte Carlo simulated annealing method (Karfunkel & Gdanitz, 1992; Akkermans *et al.*, 2013) to generate crystal structures. The Polymorph Predictor module requires the definition of motion groups on all molecular fragments for crystal structure prediction (CSP). These motion groups were defined for all molecular systems prior to initiating the CSP protocol. A motion group is effectively a collection of atoms (such as those found in a molecule) that can be translated and rotated such that the relative coordinates of each atom remains fixed during structure optimization. However, the definition of motion groups does not implicitly impose rigid body constraints although it can facilitate this and the user does need to specify whether motion groups should be kept rigid prior to initiating the search. In this work, motion groups were defined for each molecular fragment in the asymmetric unit and these motion groups were kept rigid during structure optimization. Flexible torsion angles were not optimized during the cell refinement stage of the CSP protocol. The starting point for the geometry of the molecule(s) for each system was the gas phase optimized geometry calculated at various levels of theory. For some systems, more than one rigid body search was performed depending on the number of minima found on the potential energy surface during geometry optimization – for reasons of computational expense, the potential energy surface was mapped using semi-empirical methods but final input geometries for CSP searches were calculated from the *ab initio* wavefunctions of the molecules at various levels of theory. In the case of **XXIII**, the choice of input geometries for the rigid body searches was also informed by information obtained from the Cambridge Structural

Database (CSD). Specific information regarding the methods used for obtaining different input conformations for each molecule can be found below under the appropriate section for each system. All systems with the exception of **XXIV** were attempted in this study and all searches were limited to structures with Z'=1 or Z''=2 (Mohamed *et al.*, 2009) in the case of **XXV**. In the search for plausible packing arrangements, a maximum of 7000 consecutive steps was permitted during the Monte Carlo simulation. The minimum temperature that the simulation could reach was set at 300 K and the maximum temperature was set at 1×10^5 K. For rigid body searches, an initial ranking of the lattice energy for the predicted structures was made using the Dreiding (Mayo *et al.*, 1990) force field with atomic charges derived from fitting to the molecular electrostatic potential of the *ab initio* wavefunction of the optimized conformation for the molecule. For **XXIII** and **XXVI** this is the B3LYP/6-31G(d,p) wavefunction and for all other systems, this is the MP2/6-31G(d,p) wavefunction. The fitting of the atomic charges was performed using the ChelpG (Breneman & Wiberg, 1990) scheme as implemented in GAUSSIAN09. The coverage of space groups in the rigid body searches varied depending on the complexity of the system. For all systems with the exception of **XXII**, the search for hypothetical crystal structures was limited to the following 12 space groups: $P1$, $P\overline{1}$, $P2_1$, $P2_1/c$, $P2_12_12_1$, $P2_12_12$, $Pbca$, $Pna2_1$, $Pca2_1$, $C2/c$, Cc, $C2$. For **XXII**, a wider search was performed in the following space groups, reflecting its possible "chiral-like" character: $P1, P\overline{1}, P2_1, P2_1/c, P2_12_12_1, P2_12_12, P2_1/m, P2/c, P2_13, P4_1, P4/n, P4_2/n, P4_12_12, P\overline{4}2_1c,$

$P3_1, P3_121, P\overline{3}, P6_1, P6_3/m, Pna2_1, Pca2_1, Pbca, Pbcn, Pmn2_1, Pnna, Pccn, Pbcm, Pnnm$

$Pmmn, Pnma, Pc, Pa\overline{3}, C2/c, Cc, C2, Cm, C2/m, C222_1, Cmc2_1, Cmcm, Cmca, R\overline{3}, R3, R3c$

$R\overline{3}c, Aba2, Fdd2, Fddd, Iba2, Ibam, I\overline{4}, I4/m, I4_1/a, I\overline{4}2d$. Given the large number of space groups considered in the **XXII** search, a clustering step was added between the packing and cell optimization steps. This has the disadvantage of potentially discarding the experimental structure early in the search, but was felt necessary in this case in order to manage the complexity of the search space. For all other systems, this additional clustering step was removed. For all blind test molecules, the final step in the Polymorph Predictor sequence involved clustering to determine the distinct lattice energy minima. For all systems, the criterion for this final clustering step (same settings were used for both clustering steps in **XXII**) were a tolerance of 0.13, a cut-off of 7.0 Å for the distance-distribution analysis using 140 bins and a maximum cluster size of 500.

For all rigid body searches, once the hypothetical crystal structures were generated, the 2000 lowest energy structures from the search (as ranked by Polymorph Predictor) were selected and passed to DMACRYS (Price *et al.*, 2010) for lattice minimization using a more accurate distributed multipole model (Stone, 1981; Stone & Alderton, 1985) for the electrostatic contribution towards the lattice energy, which was used as the basis for the final energetic ranking of all predicted structures in this work. This was done by performing a distributed multipole analysis of the *ab initio* charge density using GDMA2.2 (Stone, 2005). Multipoles were calculated up to rank 4 (hexadecapole) for all atoms. The dispersion-repulsion contributions towards the lattice energy were modelled using a Buckingham *exp*-6 potential using the FIT potential parameters (Williams & Cox, 1984; Cox *et al.*, 1981; Williams & Houpt, 1986; Hsu & Williams, 1980; Beyer & Price, 2000; Coombes *et al.*, 1996; Day *et al.*, 2009; Motherwell *et al.*, 2002).

1.2 XXII

The molecular structure of **XXII** was optimized at the MP2/6-31G(d,p) level using GAUSSIAN09. The gas phase optimized structure was passed to Polymorph Predictor for the generation of hypothetical crystal structures (refer to Section 1.1 for details). Subsequent multipole analysis for DMACRYS refinement assumed the MP2/6-31G(d,p) wavefunction of the optimized geometry.

1.3 XXIII

The molecular geometry of **XXIII** was optimized using the gradient corrected PBE functional with double numerical basis set with polarization (DNP) using the $DMol^3$ code (Delley, 1990, 2000) as implemented in Materials Studio. The overall *Quality* setting for the geometry optimization was set to *Fine* leading to maximum SCF cycles of 50, multipolar expansion up to Octupole and an SCF tolerance of 1.0×10^{-6}. The convergence parameters for the energy, maximum force and maximum displacement as determined by the Quality setting for the optimization were 1.0×10^{-5} Ha, 0.002 Ha $Å^{-1}$ and 0.005 Å. The optimized molecular geometry for **XXIII** was then passed onto GAUSSIAN09 for an assessment of the energy barrier for varying selected torsion angles of **XXIII.** A list of the flexible torsion angles considered is given in Figure 1.

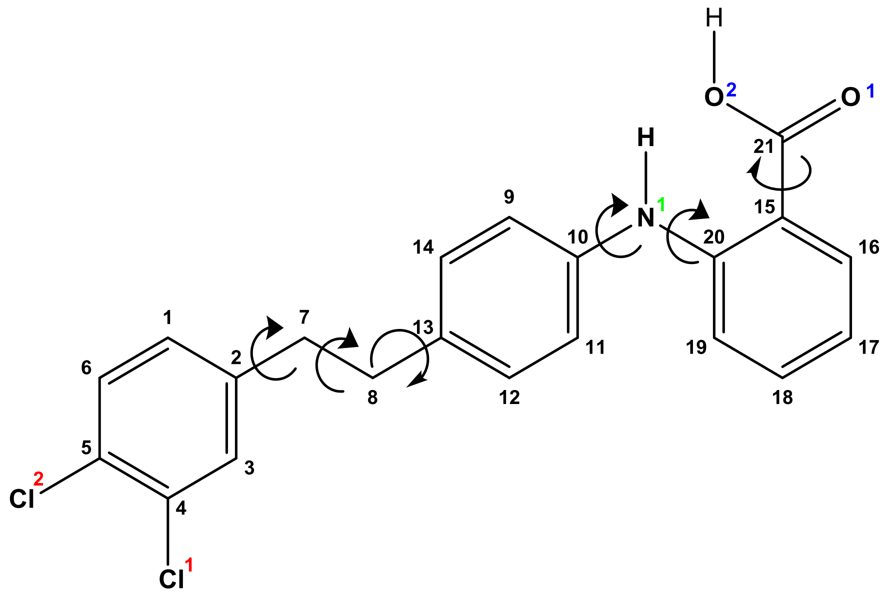

Figure 1: Molecular structure of **XXIII** with atom numbering shown. Oxygen, nitrogen and chlorine atom numbering are colored blue, green and red respectively to distinguish them from the same numbering types for carbon shown in black. The above arrows represent the torsion angles considered as part of the modelling of **XXIII**. Torsion 1 = C1-C2-C7-C8, Torsion 2 = C2-C7-C8-C13, Torsion 3 = C7-C8-C13-C12, Torsion 4 = C9-C10-N1-C20, Torsion 5 = C15-C20-N1-C10 and Torsion 6 = C16-C15-C21-O1.

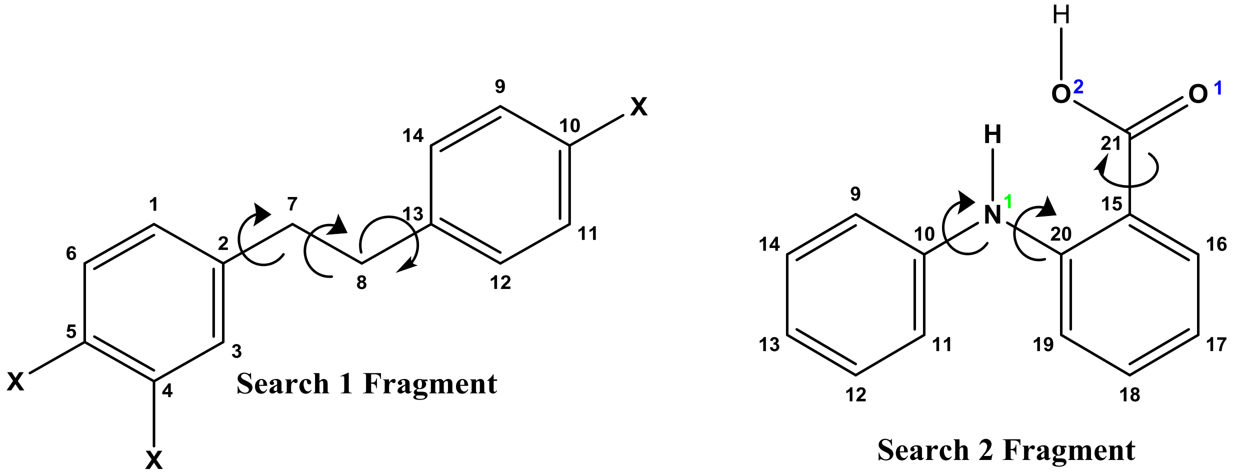

Figure 2: Molecular fragments used in CSD Searches 1 and 2. In Search 1, X denotes any atom/group. Comparing the molecular structure of **XXIII** in Figure 1 to the two search fragments used, it is obvious that Search 1 samples typical CSD values for Torsions 1, 2 and 3 whilst Search 2 samples typical CSD values for Torsions 4, 5 and 6.

The CSD was searched to get an idea of the spread of torsion angles for fragments that were similar in their atomic connectivity to the structure of **XXIII**. Two separate CSD searches employing distinct molecular fragments (Figure 2) were performed to explore the typical values of Torsion angles 1, 2 and 3 (Search 1) as well as the typical Torsion angle values for Torsions 4, 5 and 6 (Search 2). For both CSD searches, CSD Version 5.36 (November 2014) was used with the latest available update. No additional filters were used in the CSD searches.

For search 1, a total of 47 structures were retrieved and analyzed, whilst for search 2, a total of 88 structures from the search were retrieved and analyzed. The distribution of CSD values for Torsions 2 and 6 suggested that the most likely values are very close to a ±180 °. For Torsion 2, 46 out of 47 structures (98%) sampled displayed an absolute torsion angle in the range 170° to 180°. For Torsion 6, 80 out of the 88 structures (91%) sampled displayed an absolute torsion angle in the range 170° to 180°. Moreover, the vast majority of structures from Search 2 suggested that the value of Torsion 6 is such that the C=O group is very likely to be found closer to the N-H group. Torsion 1 can take a range of values but 51% of structures have absolute torsion angles in the range 80-100° with the range 90-100° having the highest frequency (14 structures). With the above CSD statistics in-hand, two separate relaxed 1-dimensional torsion scans were performed for Torsions 1 and 3 respectively starting from the optimized PBE geometry for **XXIII** as generated by $DMol^3$. The relaxed scans were performed in GAUSSIAN09 using the semi-empirical AM1 method using 36 steps of 10° each. An overlay of the potential energy surface scans for Torsions 1 and 3, showed that there were minima in the potential energy surface for each scan at approximately 107° and -73°. Given that the substitution of chlorine atom 1 (Cl1) on C4 can be on either side of the benzene ring as a function of the Torsion 1 angle, it is important to sample the two minima (labelled *Conf1* and *Conf2* in Figure 3) in the potential energy surface of Torsion 1 where C4-Cl1 is on either side of the benzene ring. Thus, the geometry of the two minima indicated in Figure 3 as *Conf1* and *Conf2* in the potential energy surface scan of Torsion 1 were extracted and used as starting points for geometry optimization at the B3LYP level of theory using the 6-31G (d,p) basis set within GAUSSIAN09. The two optimized geometries of **XXIII** at the B3LYP/6-31G(d,p) level of theory were used in separate searches for hypothetical crystal structures assuming the rigid body approximation.

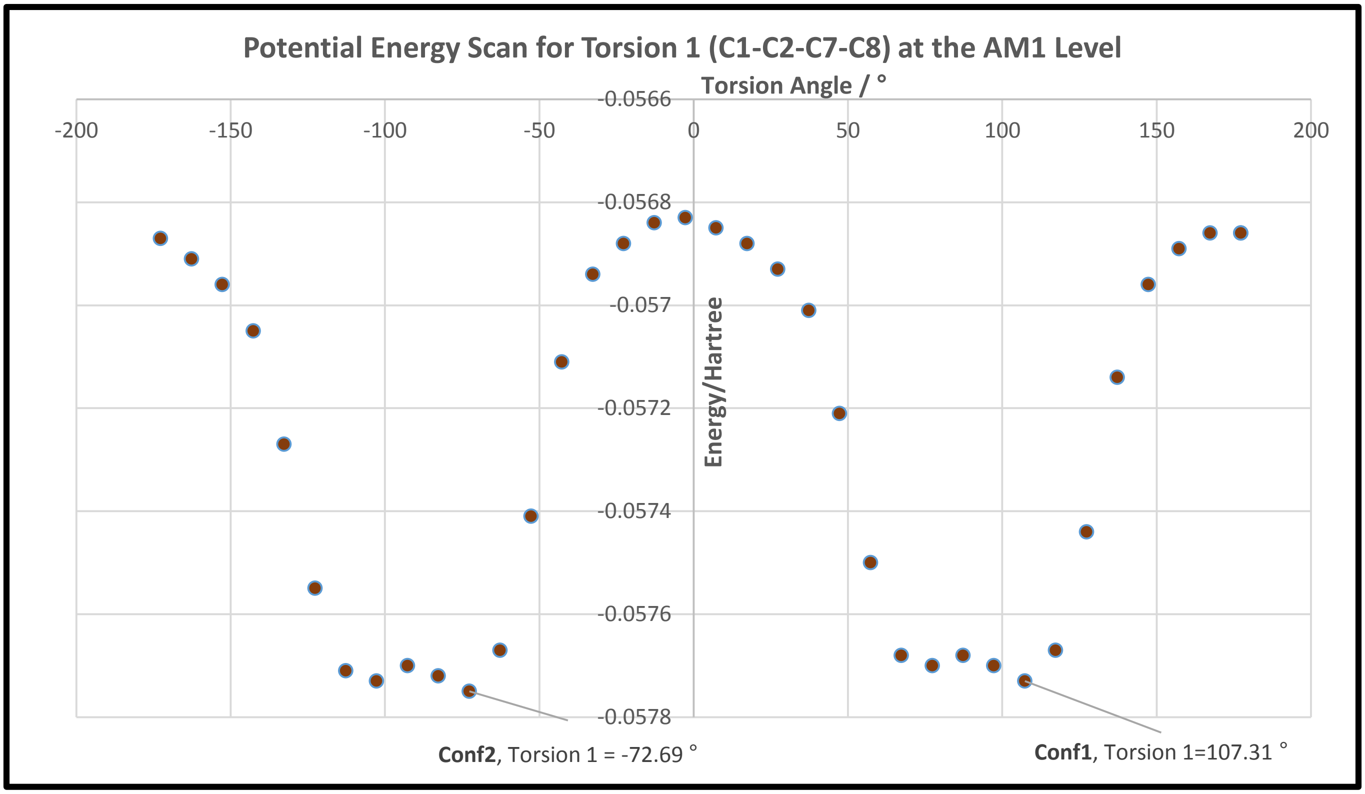

Figure 3: Potential Energy Scan for Torsion 1 (C1-C2-C7-C8) at the AM1 Level.

Following the search for hypothetical crystal structures of **XXIII** in each conformation (*Conf1* and *Conf2*), the predicted structures were passed to DMACRYS for lattice energy minimization using a distributed multipole model for the electrostatic contribution towards the lattice energy (see Section 1.1 for further details). Predicted crystal structures from searches in both conformations were collated and ranked according to the total lattice energy, which for **XXIII** consisted of the sum of the intramolecular and intermolecular energies according to the equation

$$E_{latt} = \Delta E_{intra} + E_{inter}$$

where E_{latt} is the total lattice energy, E_{inter} (estimated using DMACRYS) is the intermolecular contribution towards the crystal lattice energy and ΔE_{intra} refers to the intramolecular energy of a particular conformation relative to the intramolecular energy of the most stable conformation of

XXIII. The ΔE_{intra} term was estimated using the B3LYP/6-31G(d,p) conformational energies for *Conf1* and *Conf2* as obtained from the GAUSSIAN09 calculations.

1.4 XXV

Sketches of the molecular structures of the acid and base were used as inputs for GAUSSIAN09 geometry optimization at the MP2/6-31G(d,p) level of theory. The optimized gas phase geometries for the two constituent molecules of the cocrystal were then used to define the crystallographic asymmetric unit. Using rigid body constraints, the search for hypothetical crystal structures was performed using the Polymorph Predictor module of Materials Studio. No attempts were made to do separate rigid body searches of this cocrystal assuming different input conformations despite the expected flexibility in the torsion angles defining the CO_2H and NO_2 groups of the acid coformer.

1.5 XXVI

The molecular geometry of **XXVI** was optimized using the gradient corrected PBE functional with double numerical basis set with polarization (DNP) using the $DMol^3$ code (Delley, 1990, 2000) as implemented in Materials Studio. The overall *Quality* setting for the geometry optimization was set to *Fine* leading to maximum SCF cycles of 50, multipolar expansion up to Octupole and an SCF tolerance of 1.0×10^{-6}. The convergence parameters for the energy, maximum force and maximum displacement as determined by the Quality setting for the optimization were 1.0×10^{-5} Ha, 0.002 Ha Å^{-1} and 0.005 Å. The optimized molecular geometry for **XXVI** was then passed onto GAUSSIAN09 as a starting point for further geometry optimization at the B3LYP/6-31G(d,p) level. The optimized B3LYP/6-31G(d,p) geometry was used as input for the search for hypothetical crystal structures assuming rigid body constraints.

1.6 Computer resources used for modelling

Materials Studio (comprising of the relevant Polymorph Predictor and $DMol^3$ codes used in this work) and GAUSSIAN09 jobs were run on a dedicated Windows Server 2008 R2 Standard machine (Service Pack 1) with 64-bit operating system, 64 GB RAM and 4 processors with operating frequency of 2.0 GHz. DMACRYS was compiled and executed on an IBM x3755 M3 system with AMD Opteron 6174 architecture comprising 12 Cores and with operating frequency of 2.2 GHz. For all systems, the biggest consumption of computer resources was made during the crystal structure prediction step using the Polymorph Predictor module. The final lattice energy minimization of the predicted structures using DMACRYS was the second biggest drain on resources. With the exception of system **XXII**, the geometry optimization step - using either GAUSSIAN09 or a combination of GAUSSIAN09 and $DMol^3$ - was the least time consuming step. For **XXII**, we attribute the unusually long time taken for geometry optimization to convergence issues. Approximate CPU Hours used for all associated CSP protocols for each system are given below:

System	Approximate Computer Usage/CPU Hours
**XXII**	26
**XXIII (*Conf1* & *Conf2*)**	106
**XXV**	81
**XXVI**	61
**Total**	274

1.7 Discussion in light of experimental structures

Our group was successful in predicting at least one experimental structure for two (**XXII** and **XXIII**) out of the four systems attempted in this blind test. For **XXII**, the only known experimental structure was correctly predicted as the global minimum in lattice energy. Despite the seemingly simple chemical structure of **XXII**, there are significant challenges in the choice of the input conformation and the accuracy of the structure ranking methodology given the variety of functional groups and atom types displayed. Despite these challenges, an extensive rigid body search with a single conformation was successful in finding the experimental structure as the global minimum in lattice energy.

For **XXIII**, only polymorph b was correctly predicted from the search and this was ranked 88 in lattice energy out of a total 100 structures submitted. This polymorph adopts the conformation labelled "Conf2" in the methodology section and the success in finding this polymorph in the search illustrates that careful mapping of the conformational search space can lead to success in predicting experimental structures for flexible molecules even with limited resources in dealing with molecular flexibility. The other polymorphs for this system proved to be intractable given that we limited our search to only two rigid body searches and that $Z'>1$ structures were not considered during the structure generation step.

For **XXV** and **XXVI**, the failure to predict the experimental structures was primarily a result of the incorrect starting points assumed for the molecular conformations in the rigid body searches and the lack of available resources for optimizing the lattice energy as a function of selected torsion angles. Comparing the predicted structures with the known experimental structures for these systems showed that reasonable hydrogen bonding motifs were being predicted from the search.

1.8 Acknowledgments

This work would not have been possible without funding from Khalifa University's College of Engineering. I would like to acknowledge Prof. Robert Bennell and Prof. Bayan Sharif for supporting me acquire the resources needed to carry out this research. Dr. Louise Price is thanked for her guidance on the use of DMACRYS and NEIGHCRYS during the course of this research. She is also thanked for useful discussions and numerous e-mail exchanges concerning the blind test. Prof. Sally Price is acknowledged for her support and guidance over many years and for providing access to DMACRYS and NEIGHCRYS.

1.9 Bibliography

Accelrys (2013). *Materials Studio v7.0.200.* Version Materials Studio 7.0.200.

Akkermans, R. L., Spenley, N. A. & Robertson, S. H. (2013). *Molecular Simulation* **39**, 1153-1164.

Beyer, T. & Price, S. L. (2000). *The Journal of Physical Chemistry B* **104**, 2647-2655.

Breneman, C. M. & Wiberg, K. B. (1990). *Journal of Computational Chemistry* **11**, 361-373.

Coombes, D. S., Price, S. L., Willock, D. J. & Leslie, M. (1996). *The Journal of Physical Chemistry* **100**, 7352-7360.

Cox, S. R., Hsu, L.-Y. & Williams, D. E. (1981). *Acta Crystallographica Section A* **37**, 293-301.

Day, G. M., Cooper, T. G., Cruz-Cabeza, A. J., Hejczyk, K. E., Ammon, H. L., Boerrigter, S. X. M., Tan, J. S., Della Valle, R. G., Venuti, E., Jose, J., Gadre, S. R., Desiraju, G. R., Thakur, T. S., van Eijck, B. P., Facelli, J. C., Bazterra, V. E., Ferraro, M. B., Hofmann, D. W. M., Neumann, M. A., Leusen, F. J. J., Kendrick, J., Price, S. L., Misquitta, A. J., Karamertzanis, P. G., Welch, G. W. A., Scheraga, H. A., Arnautova, Y. A., Schmidt, M. U., van de Streek, J., Wolf, A. K. & Schweizer, B. (2009). *Acta Crystallographica Section B* **65**, 107-125.

Delley, B. (1990). *The Journal of Chemical Physics* **92**, 508-517.

Delley, B. (2000). *The Journal of Chemical Physics* **113**, 7756-7764.

Hsu, L.-Y. & Williams, D. E. (1980). *Acta Crystallographica Section A* **36**, 277-281.

Karfunkel, H. R. & Gdanitz, R. J. (1992). *Journal of Computational Chemistry* **13**, 1171-1183.

Mayo, S. L., Olafson, B. D. & Goddard, W. A. (1990). *Journal of Physical Chemistry* **94**, 8897-8909.

Mohamed, S., Tocher, D. A., Vickers, M., Karamertzanis, P. G. & Price, S. L. (2009). *Crystal Growth & Design* **9**, 2881-2889.

Motherwell, W. D. S., Ammon, H. L., Dunitz, J. D., Dzyabchenko, A., Erk, P., Gavezzotti, A., Hofmann, D. W. M., Leusen, F. J. J., Lommerse, J. P. M., Mooij, W. T. M., Price, S. L., Scheraga, H., Schweizer, B., Schmidt, M. U., van Eijck, B. P., Verwer, P. & Williams, D. E. (2002). *Acta Crystallographica Section B* **58**, 647-661.

Price, S. L., Leslie, M., Welch, G. W. A., Habgood, M., Price, L. S., Karamertzanis, P. G. & Day, G. M. (2010). *Physical Chemistry Chemical Physics* **12**, 8478-8490.

Stone, A. J. (1981). *Chemical Physics Letters* **83**, 233-239.

Stone, A. J. (2005). *Journal of Chemical Theory and Computation* **1**, 1128-1132.

Stone, A. J. & Alderton, M. (1985). *Molecular Physics* **56**, 1047-1064.

Williams, D. E. & Cox, S. R. (1984). *Acta Crystallographica Section B* **40**, 404-417.

Williams, D. E. & Houpt, D. J. (1986). *Acta Crystallographica Section B* **42**, 286-295.
