# Supplementary material for: Report on the sixth blind test of organic crystal structure prediction methods
Source: Acta Crystallogr B Struct Sci Cryst Eng Mater. 2016 Aug 1;72(Pt 4):439–59. doi: 10.1107/S2052520616007447 (PMC4971545; doi:10.1107/S2052520616007447)
Supplement: Supplementary file 3 [file b-72-00439-sup3.zip › Group_14_Neumann_Kendrick_Leusen_Post_Analysis.pdf]

Supplementary information for the blind test 2014 submission of Neumann/Kendrick/Leusen

Method

All crystal structure predictions were carried out with the GRACE 2.4 software package. Each crystal structure prediction with GRACE consists of two parts. Firstly, a tailor-made force field [1] is parameterized from scratch with the Force Field Factory module, and then the actual crystal structure prediction is carried out with the CSP Factory module (see Fig. 1). The workflows of both modules are fully automated. A feedback loop between CSP Factory and Force Field Factory allows the use of preliminary crystal structure prediction results to improve the force field accuracy.

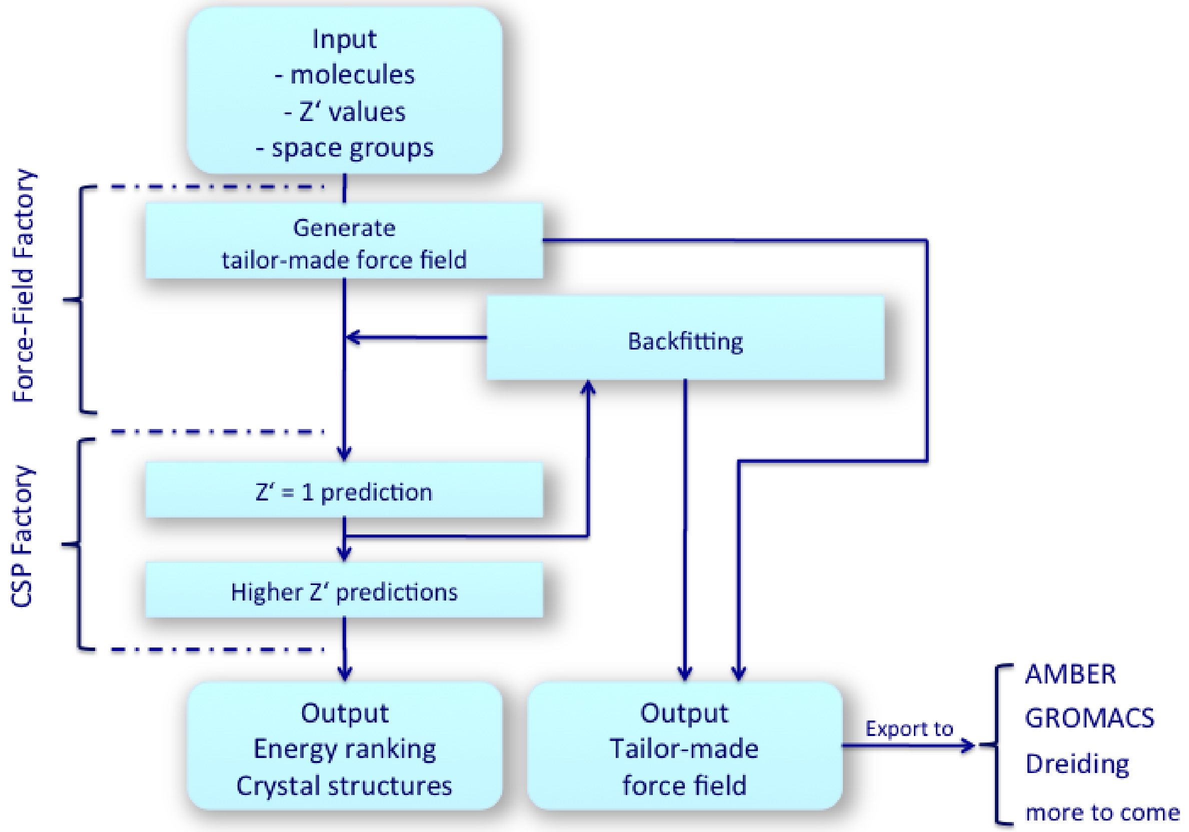

Figure 1: Force Field Factory and CSP Factory in GRACE

The tailor-made force field uses fixed atomic point charges calculated from bond increments and atomic charges (for atoms with a formal charge), isotropic exp-6 van der Waals interactions, harmonic bond stretch and angle bend terms, overall torsions and overall inversions. All force field parameters are fitted simultaneously to ab initio reference data generated at the DFT-D level of theory. The empirical dispersion correction was developed in-house [2]. The DFT part of the energy calculations is performed either with VASP [3-5] or Turbomole [6]. Reference data for ionic species are generated in the COSMO approximation. Larger molecules are automatically cut into chemically reasonable fragments, which are then parameterized. In such a case, no reference data are generated for the complete molecule in the initial force field

parameterization procedure. Subsequently, additional data for the complete molecule from a preliminary crystal structure prediction can improve the force field accuracy by 10-20%.

Crystal structure prediction with the CSP Factory uses a three-step procedure for each Z' value considered (Z' is the number of molecules in the asymmetric unit). Firstly crystal structures are generated with the tailor-made force field by a Monte Carlo parallel tempering algorithm. Then promising candidates are subjected to a DFT-D lattice energy optimization with loose convergence settings and finally the most promising candidates from step 2 undergo a second DFT-D lattice energy optimization with tighter convergence settings.

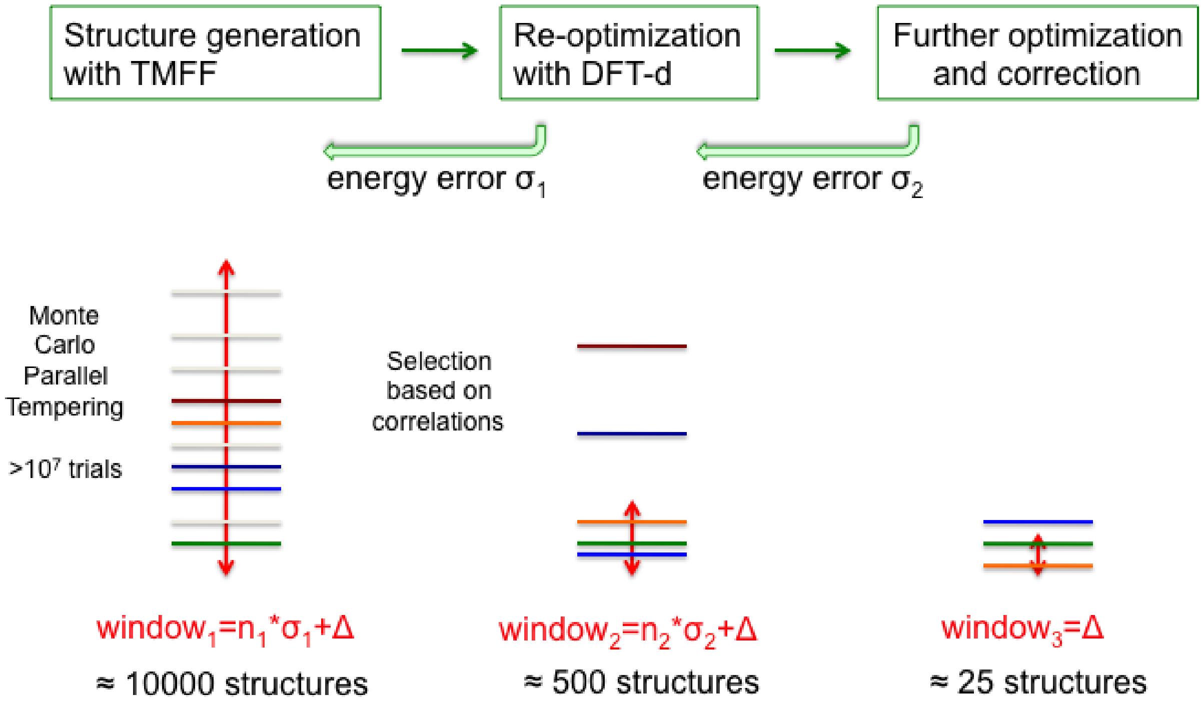

Figure 2: Three-step crystal structure prediction.

Statistical methods are used to control the completeness of the three-step procedure. The energy accuracy at each step is assessed by comparison with the energies obtained at the next step. This way, standard deviations σ_1 and σ_2 are obtained for the force field energies of the first step and the coarse DFT-D energies of the second step, respectively. The user typically specifies an energy window, Δ, for the last step, in which the crystal structure generation should be quasi-complete. In the intermediate step, a larger window of $\Delta + n_2\sigma_2$ is used, and for the initial structure generation step with the tailor-made force field the energy window $\Delta + n_1\sigma_1$ is even larger. For Z'=1 (Z'=2), the standard choice is n_2=3 (2) and n_1=4 (3). In the first and the second step, statistical considerations are used to estimate the number of lattice energy minima and a step is considered complete for Z'=1 (Z'=2) when 99% (95%) of all structures have been generated. All structures found in the energy window of step 2 are further processed in step 3.

To assess the completeness of the first step, statistically independent generations of each lattice energy minimum are counted and two completeness estimates are

calculated, one based on the average number of hits and the other one based on the number of structures that are hit only once. In the second step, it would be too CPU time consuming to optimize the lattice energies of all crystal structures in the target window of the first step. Instead, statistical correlations are built up between structural similarity and the behaviour upon optimization, and between the DFT-D force calculated for each force-field optimized structure and the behaviour upon optimization. These considerations result in various energy estimates with standard deviations that are used to estimate the number of lattice energy minima in the target energy window of the second step.

DFT calculations use a plane wave cutoff energy of 520eV and a k-point spacing of roughly 0.07 $Å^{-1}$. All lattice energy minimizations of the final step have been converged to within at least 0.003 Å for atomic displacements, 0.00025 kcal/mol/atom for energy changes, 0.7 kcal/mol/Å for atomic forces and 1.0 kbar for cell stress. In the second step, lattice energies are converged to within at least 0.02 Å for atomic displacements, 0.001 kcal/mol/atom for energy changes, 7.0 kcal/mol/Å for atomic forces and 15.0 kbar for cell stress. The lists of structures submitted in the 2014 blind test contain structures both from the second and the third steps, because typically less than 100 structures are processed in the third step.

Search space

Grace offers several standard collections of space groups:

Choice	Coverage
Safe	All space groups.
Quasi-safe	For Z'=1, the 38 most frequent space groups according to CSD statistics covering 99.9% of all Z'=1 entries.
Pragmatic	For Z'=2, the 11 most frequent space groups according to CSD statistics covering 95% of all Z'=2 entries (P1, P-1, $P2_1$, C2, Pc, Cc, $P2_1/c$, C2/c, $P2_12_12_1$, $Pca2_1$, $Pna2_1$).

For the five target compounds of the 2014 blind test, the following choices were made.

Compound	Z'=1 space groups	Z'=2 space groups	Step-3 target window [kcal/mol]
xxii	Safe	none	1.0
xxiii	Quasi-safe	Pragmatic	1.0
xxiv	Quasi-safe	none	1.0
xxv	Quasi-safe	none	1.0
xxvi	Quasi-safe	Pragmatic	1.5

Deviations from the standard procedure

In some cases manual changes to the standard workflows were made:

Compound	Change
xxiii	The Z'=1 step-2 reranking was manually continued to reach higher completeness with an energy window of 2.0 kcal/mol.
xxxvi	The Z'=1 step-2 reranking was manually continued to reach higher completeness with an energy window of 3.0 kcal/mol. The Z'=2 step-1 structure generation was interrupted at 80% completeness because the blind test deadline was approaching. The Z'=2 step-3 final optimization was not carried out.

Hardware

The calculations were run on two different computing clusters.

Cluster 1: Purchased in 2009. 28 node Beowulf cluster. Each node of this cluster comprises two quad-core 2.8 GHz Intel® Xeon™ X5462 processors and each node has 16GB of memory. All nodes communicate via an InfiniBand network. In total the cluster has 224 cores.

Cluster 2: Purchased in 2013. 24 node Linux cluster. Each node of this cluster comprises two 8-core 2.6 GHz Intel® Xeon™ E5-2670 processors and has 32GB of memory. All nodes communicate via an InfiniBand network. In total, the cluster has 384 cores.

CPU time consumption on cluster 1 is converted to CPU time consumption on cluster 2 using a conversion factor of 3.

CPU time consumption

CPU time is specified in core time hours. For calculations run on cluster 1, the CPU time consumption before conversion to cluster 2 is given in brackets. Cluster 2 was used to generate all force fields and for the CSP of target xxvi. Cluster 1 was used for the CSPs of targets xxii, xxiii, xxiv and xxv.

Compound	Force field generation [core hours]	CSP [core hours]	Total [core hours]
xxii	20400	11760 (35280)	32160
xxiii	30200	115920 (347760)	146120
xxiv	27500	67200 (201600)	103700
xxv	15800	68880 (206640)	84680
xxvi	43500	313344	356844

At a load of 100%, the wall time on Cluster 2 (384 cores) for all 5 crystal structure prediction studies of the 2014 blind test would have been 75 days.

Submitted lists

Compound	List name	Description
xxii	xxii_nkl.cif	Lowest energy structures from steps 2 and 3. Duplicates removed.
xxiii	xxiii_nkl_zp1.cif	Z'=1 lowest energy structures from steps 2 and 3. Duplicates removed.
	xxiii_nkl_zp1and2.cif	Z'=1 and Z'=2 lowest energy structures from steps 2 and 3. Duplicates removed.
xxiv	xxiv_nkl.cif	Lowest energy structures from steps 2 and 3. Duplicates removed.
xxv	xxv_nkl.cif	Lowest energy structures from steps 2 and 3. Duplicates removed.
xxvi	xxvi_nkl_zp1.cif	Z'=1 lowest energy structures from steps 2 and 3. Duplicates removed.
	xxvi_nkl_zp1and2.cif	Z'=1 and Z'=2 lowest energy structures from steps 2 and 3. Duplicates removed.

Post analysis

Tables 1 and 2 show a comparison of the experimental and calculated unit cells. Table 1 shows the absolute values of the unit cell parameters, while Table 2 shows the percentage change from the experimental values after optimisation. The optimisations were performed starting from the experimental structures. The force fields used were the final force fields determined during the crystal structure predictions. The DFT(D) settings were the same as those used during the CSP. The ranks are those in the most complete list available. If Z'=1 and Z'=2 searches were performed then the ranks refer to the positions in that list.

There is one result which sticks out, namely the force field optimised energy and rank for compound XXIV. The energy of this structure appears very high and is ranked at 2220. Of course despite this there may have been other lower energy force field structures which optimised to the rank 2 DFT(D) structure. Still the result indicates that there may be a problem with an energy term in the force field.
The root mean squared percentage errors in the unit cell parameters are 2.9% and 1.6% for the force field and the DFT(D) optimised results respectively. The force field percentage errors varied between -9.0 and 8.1%, whilst the DTF(D) percentage errors varied between -4.6 and 2.7%.

Table 1: Comparison of Experimental and Calculated Unit Cells

		**Rank**[a]	**ΔE**[b]	**a/Å**	**b/Å**	**c/Å**	**α/°**	**β/°**	**γ/°**
XXII	Exptl			11.947	6.696	12.598	90	108.60	90
	Force field	6	0.0327	12.352	6.442	12.562	90	110.90	90
	DFT(D)	2	0.0140	12.160	6.751	12.924	90	109.97	90
XXIIIa	Exptl			11.164	10.530	16.236	90	95.75	90
	Force field	3979	0.0697	12.066	9.786	15.335	90	94.13	90
	DFT(D)	85	0.0251	10.897	10.579	16.233	90	95.09	90
XXIIIb	Exptl			7.006	7.805	18.893	85.28	80.75	65.77
	Force field	278	0.0383	7.007	7.876	18.179	87.33	80.55	63.76
	DFT(D)	4	0.0032	7.088	7.788	18.278	87.56	80.59	64.38
XXIIIc	Exptl			7.638	12.039	20.443	84.79	85.38	80.09
	Force field	153	0.0341	7.618	11.744	20.190	85.79	86.36	80.28
	DFT(D)	6	0.0038	7.532	11.827	20.322	86.48	86.45	80.34
XXIIId	Exptl			13.886	10.728	14.078	90	113.63	90
	Force field	3837	0.0651	14.510	10.549	13.802	90	119.40	90
	DFT(D)	39	0.0181	14.200	10.847	13.547	90	116.53	90
XXIIIe	Exptl			6.687	12.180	24.390	102.88	96.69	97.22
	Force field		0.0599	6.570	11.876	24.402	105.88	95.64	97.24
	DFT(D)		0.0143	6.569	11.994	23.758	98.14	97.73	96.71
XXIV	Exptl			4.026	21.304	10.125	90	97.81	90
	Force field	2220	0.2758	4.017	21.927	9.214	90	103.22	90
	DFT(D)	2	0.0067	4.098	20.915	9.956	90	98.61	90
XXV	Exptl			10.408	27.547	8.112	90	109.58	90
	Force field	1	0.0000	10.391	27.913	7.829	90	110.81	90
	DFT(D)	6	0.0129	10.443	27.262	8.030	90	109.93	90
XXVI	Exptl			10.402	11.030	14.179	76.83	73.33	63.47
	Force field	88	0.0284	10.313	11.199	14.397	75.57	72.87	61.61
	DFT(D)	1	-0.0001	10.286	11.073	13.964	78.52	73.46	63.64

a) Energies are in kcal/mol/atom. In the cases where Z’=1 and Z’=2 searches were performed the rank is that in the combined list.
b) The energies are relative to the global minimum found for that compound.

Table 2: Comparison of Experimental and Calculated Unit Cells[a]

		Rank[b]	ΔE[c]	a/Å	b/Å	c/Å	α/⁰	β/⁰	γ/⁰
XXII	Exptl			11.947	6.696	12.598	90	108.60	90
	Force field	6	0.0327	3.4	-3.8	-0.3	0.0	2.1	0.0
	DFT(D)	2	0.0140	1.8	0.8	2.6	0.0	1.3	0.0
XXIIIa	Exptl			11.164	10.530	16.236	90	95.75	90
	Force field	3979	0.0697	8.1	-7.1	-5.5	0.0	-1.7	0.0
	DFT(D)	85	0.0251	-2.4	0.5	0.0	0.0	-0.7	0.0
XXIIIb	Exptl			7.006	7.805	18.893	85.28	80.75	65.77
	Force field	278	0.0383	0.0	0.9	-3.8	2.4	-0.3	-3.1
	DFT(D)	4	0.0032	1.2	-0.2	-3.3	2.7	-0.2	-2.1
XXIIIc	Exptl			7.638	12.039	20.443	84.79	85.38	80.09
	Force field	153	0.0341	-0.3	-2.5	-1.2	1.2	1.1	0.2
	DFT(D)	6	0.0038	-1.4	-1.8	-0.6	2.0	1.3	0.3
XXIIId	Exptl			13.886	10.728	14.078	90	113.63	90
	Force field	3837	0.0651	4.5	-1.7	-2.0	0.0	5.1	0.0
	DFT(D)	39	0.0181	2.3	1.1	-3.8	0.0	2.5	0.0
XXIIIe	Exptl			6.687	12.180	24.390	102.88	96.69	97.22
	Force field		0.0599	-1.8	-2.5	0.0	2.9	-1.1	0.0
	DFT(D)		0.0143	-1.8	-1.5	-2.6	-4.6	1.1	-0.5
XXIV	Exptl			4.026	21.304	10.125	90	97.81	90
	Force field	2220	0.2758	-0.2	2.9	-9.0	0.0	5.5	0.0
	DFT(D)	2	0.0067	1.8	-1.8	-1.7	0.0	0.8	0.0
XXV	Exptl			10.408	27.547	8.112	90	109.58	90
	Force field	1	0.0000	-0.2	1.3	-3.5	0.0	1.1	0.0
	DFT(D)	6	0.0129	0.3	-1.0	-1.0	0.0	0.3	0.0
XXVI	Exptl			10.402	11.030	14.179	76.83	73.33	63.47
	Force field	88	0.0284	-0.9	2.0	1.8	-1.9	-1.3	-3.6
	DFT(D)	1	-0.0001	-1.1	0.4	-1.5	2.2	0.2	0.3

a) Calculated unit cells are given as a percentage change to the experimental value. A positive value indicates an increase.

b) In the cases where Z'=1 and Z'=2 searches were performed the rank is that in the combined list.

c) Energies are in kcal/mol/atom. The energies are relative to the global minimum found for that compound.

CSP calculations for the co-formers of co-crystal XXV

As part of the CSP for co-crystal XXV, independent CSPs were performed for the two co-formers, shown in Figure 3. The CSPs considered all possible space groups for a single molecule in the unit cell. Molecule XXV$_1$ is commonly known as Troger's base and has an entry AXAGEL in the CSD with space group symmetry $P2_12_12_1$. There is also an entry DILLEP which is a racemate of the same molecule with space group symmetry *Pccn* and 1.5 molecules in the asymmetric unit. Molecule XXV$_2$ has several entries in the database and comparison will be made between the CSP structures and the polymorphs CUKCAM13 and CUKCAM021, which crystallise in the $P2_1/c$ and $C2/c$ space groups respectively.

XXV$_1$	XXV$_2$
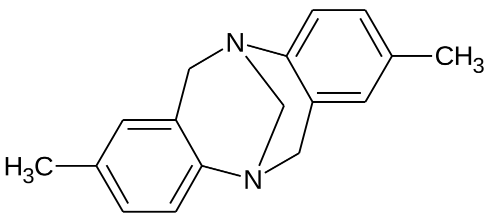	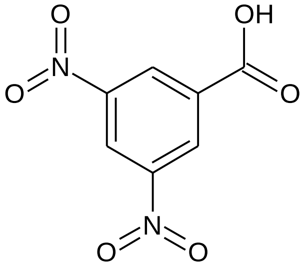

Figure 3: Structures of the co-formers of co-crystal XXV.

Table 3 summarises the experimental structures of the co-formers of the co-crystal XXV available in the CSD.

Table 3: Experimental Unit Cell Parameters for XXV$_1$ and XXV$_2$.

CSD Code	Space Group	a/Å	b/Å	c/Å	$\alpha/^{0}$	$\beta/^{0}$	$\gamma/^{0}$	volume/Å^3
DILLEP	*Pccn*	12.774	30.29	10.386	90	90	90	4018.6
AXAGEL	$P2_12_12_1$	6.110	7.934	28.328	90	90	90	1373.25
CUKCAM13	$P2_1/c$	9.784	8.944	9.450	90	97.6	90	819.727
CUKCAM21	$C2/c$	20.200	8.745	9.645	90	109.8	90	1603.05

Table 4 shows the 10 lowest energy structures found by the CSP on molecule XXV$_1$. Any predicted structure which matches an experimental structure is shaded in pink. As all space groups were considered in the CSP both chiral and racemic crystals were found. The lowest energy structure predicted to be chiral was a $P2_12_12_1$ structure at rank 3, 0.805 kcal/mol above the lowest energy structure, which corresponds well with the experimental structure of AXAGEL. Two racemic structures were found at lower energies with $C2/c$ space group symmetry. The racemic structure corresponding to DILLEP was not found as it has more than one molecule in the asymmetric unit.
Table 5 shows the 10 lowest energy structures found by the CSP on molecule XXV$_2$. The lowest energy structure at rank 1 corresponds to the experimental structure found in CUCKAM21. The other known polymorph of this molecule is predicted at rank 5, 0.517 kcal/mol above the lowest energy structure. The methods we are using appear to be reliable for these molecules.

Table 4: CSP results for molecule XXV_1.

Rank	Space Group	a/Å	b/Å	c/Å	α/°	β/°	γ/°	volume /Å^3	ΔE kcal/mol
1	$C2/c$	22.457	5.212	18.927	90	143.11	90	1329.7	0
2	$C2/c$	14.322	9.225	10.893	90	113.56	90	1319.2	0.579
3	$P2_12_12_1$	6.068	7.961	27.309	90	90	90	1319.2	0.805
4	$P2_1/c$	11.220	13.960	8.481	90	90.19	90	1328.4	0.919
5	$P2_1/c$	14.220	7.819	12.174	90	76.90	90	1318.5	0.941
6	$C2/c$	39.421	5.215	13.629	90	74.04	90	2693.8	1.239
7	$P2_1/c$	14.529	7.712	12.063	90	100.80	90	1327.8	1.254
8	$P2_1/c$	5.507	12.088	20.412	90	78.76	90	1332.7	1.269
9	$Pna2_1$	27.813	6.046	7.936	90	90	90	1334.7	1.336
10	$P\bar{1}$	6.283	10.430	10.846	98.94	74.45	97.38	673.6	1.505

Table 5: CSP results for molecule XXV_2.

Rank	Space Group	a/Å	b/Å	c/Å	α/°	β/°	γ/°	volume /Å^3	ΔE kcal/mol
1	$C2/c$	20.727	8.688	9.801	90	110.50	90	1653.2	0
2	$C2/c$	21.157	8.671	9.728	90	68.72	90	1663.0	0.367
3	$Pbca$	7.852	8.611	24.203	90	90	90	1636.5	0.382
4	$P2_1/c$	9.467	6.372	14.037	90	97.44	90	839.7	0.493
5	$P2_1/c$	10.191	8.728	9.440	90	96.95	90	833.5	0.517
6	$P2_1/c$	9.505	6.361	14.006	90	82.64	90	839.9	0.556
7	$P2_1/c$	11.652	8.129	8.896	90	87.56	90	841.9	0.609
8	$Pbca$	7.869	8.815	23.811	90	90.00	90	1651.4	0.717
9	$Pccn$	8.870	19.049	9.923	90	90	90	1676.6	0.911
10	$C2/c$	26.025	6.075	11.072	90.00	104.74	90.00	1692.9	0.960

Why was polymorph XXIIIe missed?

Polymorph XXIIIe has two molecules per asymmetric unit (Z'=2). The target size of the Z'=2 structure generation window was three-times the force field accuracy plus the user-defined window size ($3\sigma_1 + \Delta$). The force field accuracy used for Z'=2 is determined from force field and DFT-D calculations for Z'=1, and it may happen that the actual force field accuracy for Z'=2 is less because the additional degrees of freedom allow for interactions not observed for Z'=1. In the case of XXIII, this happened and the actual structure generation window size for Z'=2 was only about 2.4 σ_1. By optimising the experimental XXIIIe structure with the force field it was found that the energy of the structure falls just outside the 2.4 σ_1 energy window covered in the Z'=2 structure generation. In order to make sure that XXIIIe would not be missed again under similar circumstances, the target value for the Z'=2 structure generation window in GRACE has now been increased from 3.0 σ_1 to 3.5 σ_1. It has not been set to 4.0 (default for Z' =1) because the window size strongly affects the CPU-time requirements of the structure generation step.

References

[1] Neumann, M. A. Tailor-made force fields for crystal-structure prediction. *J. Phys. Chem. B* **112,** 9810-9829 (2008).

[2] Neumann, M. A. & Perrin, M.-A. Energy ranking of molecular crystals using density functional theory calculations and an empirical van der Waals correction. *J. Phys. Chem. B* **109,** 15531-15541 (2005).

[3] Kresse, G. & Furthmüller, J. Efficiency of ab-initio total energy calcualtions for metals and semiconducturs using a plane-wave basis set. *Comput. Mat. Sci.* **6,** 15-50 (1996).

[4] Kresse, G. & Furthmüller, J. Efficient iterative schemes for ab initio total-energy calculations using a plane-wave basis set, *Phys. Rev. B* **54,** 11169-11186 (1996).

[5] Kresse, G. & Joubert, D. From ultrasoft pseudopotentials to the projector augmented-wave method. *Phy. Rev. B* **59,** 1758-1775 (1999).

[6] TURBOMOLE V6.2.2010, a development of University of Karsruhe and ForschungszentrumKarlsruhe GmbH, 1989-2007. Turbomole GmbH since 2007.
