# Supplementary material for: Report on the sixth blind test of organic crystal structure prediction methods
Source: Acta Crystallogr B Struct Sci Cryst Eng Mater. 2016 Aug 1;72(Pt 4):439–59. doi: 10.1107/S2052520616007447 (PMC4971545; doi:10.1107/S2052520616007447)
Supplement: Supplementary file 3 [file b-72-00439-sup3.zip › Group_15_Pantelides_Adjiman_Post_Analysis.pdf]

Sixth Blind Test of Organic Crystal Structure Prediction Methods

Supporting Information

Isaac J. Sugden, Christina-Anna Gatsiou, Manolis Vasileiadis, Claire S. Adjiman, Constantinos C. Pantelides

Department of Chemical Engineering, Centre for Process Systems Engineering, Imperial College London, London SW7 2AZ

c.adjiman@imperial.ac.uk; c.pantelides@imperial.ac.uk

1. Methodology

1.1 General methodology

The general methodology used for Crystal Structure Prediction in this submission follows a three-stage process (Pantelides *et al.*, 2014).

Step 1: identification of flexibility within the molecule, and setting up of input files that describe the flexibility. Relevant data from the Crystal Structure Database (CSD) are used to choose an appropriate level of theory.

Step 2 – Global search: generation of putative crystal structures from an extensive search space using an approximation of the lattice energy based on a set of isolated-molecule quantum mechanical calculations, a simple representation of electrostatics (point charges), and an empirical term to model intramolecular repulsion/dispersion. The approximation is based either on restricted Hermite interpolants (CrystalPredictor I (Karamertzanis & Pantelides, 2005, 2007)) or quadratic local approximate models (LAMs, CrystalPredictor II (Habgood *et al.*, 2015)). Only Z'=1 structures were generated for all searches undertaken during the blind test. Structures were generated within the 59 most common space groups.

Step 3 - Refinement: refinement of the most promising structures using a local optimisation algorithm to identify the 100 structures with the lowest lattice energy:

- for rigid molecules, DMACRYS (Price et al., 2010) is used. Relative to Step 2, this entails a more detailed modelling of electrostatics (via multipoles) and, if appropriate, a higher level of theory. The repulsion/dispersion term can also be changed from that used in Step 2.
- for flexible molecules, CrystalOptimizer (Kazantsev et al., 2011) is used. Relative to Step 2, this entails a more detailed modelling of electrostatics (via multipoles) and flexibility, and, if appropriate, a higher level of theory. CrystalOptimizer is based on a two-level optimisation, with the flexible degrees of freedom as variables at the outer level, and the lattice parameters and molecular position/orientation as variables at the

inner level. The inner optimisation is performed by DMACRYS (Price et al., 2010). At the outer level, quantum mechanical calculations on isolated molecules are carried out on the fly and LAMs are used to approximate the lattice energy based on these calculations.

Step 3 can be split into two steps, 3a and 3b, to reduce the number of structures that are fully minimised with the more detailed model. In Step 3a, a re-ranking of the structures generated in Step 2 is carried out with a single iteration of CrystalOptimizer, as shown for the axitinib molecule in Vasileiadis et al. (2015). Step 3b then consists of the full local minimisation of the most promising re-ranked structures with the final model.

Molecules XXII, XXIII, XXV and XXVI were investigated and specific aspects for each molecule are described in the remainder of this document. Molecule XXIV was not attempted.

1.2 Molecule XXII (Gatsiou, Vasileiadis, Sugden, Adjiman, Pantelides)

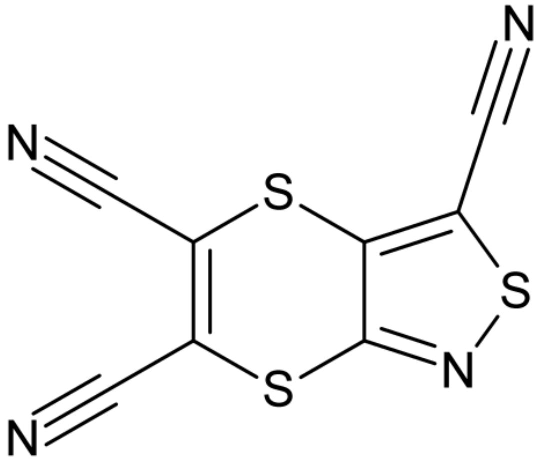

Figure 1 - Molecule XXII

A key challenge for this small rigid molecule (Figure 1) is the choice of model. In our methodology, this includes the level of theory and the choice of repulsion-dispersion potential. The FIT potential (Williams, 1984; Cox et al., 1981; Williams & Starr, 1977), augmented with the S parameter derived in a previous blind test (Lommerse et al., 2000), would typically be used for repulsion-dispersion interactions. While we undertake part of the search with these parameters, which we refer to as "FIT+S", we finalise the list of structures using a new set of parameters for all atoms involved, developed through extensive fitting to geometry and energy data for a wide range of compounds containing the relevant atoms. The Buckingham potential is used:

$$U(r_{ij}) = A_{ij} \exp\left(-\frac{r_{ij}}{B_{ij}}\right) - C_{ij}/{r_{ij}}^6 \qquad (1)$$

The parameters are reported in Table 1. They were developed using the M06/6-31(d,p) level of theory, and the method adopted to derive them will be reported elsewhere.

Table 1 – Repulsion dispersion parameters developed in this work and used in refinement Step 3.

Interaction	A_{ij}(eV)	B_{ij}(Å)	C_{ij}(eVÅ^6)
C...C	2419.415	0.2778	18.535
N…N	4484.539	0.2646	19.883
S...S	4447.155	0.3030	110.731
C...N	3718.398	0.2710	25.563
N…S	4375.588	0.2825	46.850
C...S	4481.922	0.2898	56.890

Key information for each step of the CSP approach as applied to Molecule XXII is reported in Table 2. A summary of the number of structures identified is presented in Table 3 and the energy landscape with the 100 lowest energy structures is shown in Figure 2.

Step 1. The molecule was identified as rigid through the study of flexibility of similar molecules in the CSD (NAMSUR, ISTZCN10). The conformation was therefore fixed to the gas phase conformation for the relevant level of theory in every subsequent step. Local minimisations of similar molecules (NAMSUR, CBITAZ, ISTZCN10) were carried out at HF/6-31(d,p), M06/6-31(d,p), PBE0/6-31G(d,p), B3LYP/6-31G(d,p) levels of theory and HF/6-31G(d,p) was found to reproduce the geometry of the known crystal structures comparatively well and was therefore chosen for initial investigation.

Step 2. CrystalPredictor I was used to generate 100,000 structures (cf Table 2) and 1416 structures were found within 20 kJ/mol of the global minimum.

Step 3. The 1416 minima were refined with DMACRYS (Price et al., 2010) using the M06/6-31G(d,p) level of theory for the calculation of multipoles.

Table 2- Key information for each step of the CSP methodology for Molecule XXII

**Step**	1	2	3
**Method**	CSD local minimisations with DMACRYS	CrystalPredictor I	DMACRYS
**Flexibility**	None	None	None
**Level of theory**	Several See text	HF/6-31G(d,p)	M06/6-31G(d,p)
**Electrostatics**	DMA	RESP	DMA
**Dispersion/repulsion**	FIT+S	FIT + S	Table 1
**No. of structures minimised**	3	100,000	1,416

Table 3. Number of structures found for Molecule XXII, as a function of energy difference relative to the global minimum

	**Cutoff from global minimum (kJ/mol)**		
	**5**	**10**	**20**
**Step 2**	18	118	1,416
**Step 3**	7	48	440

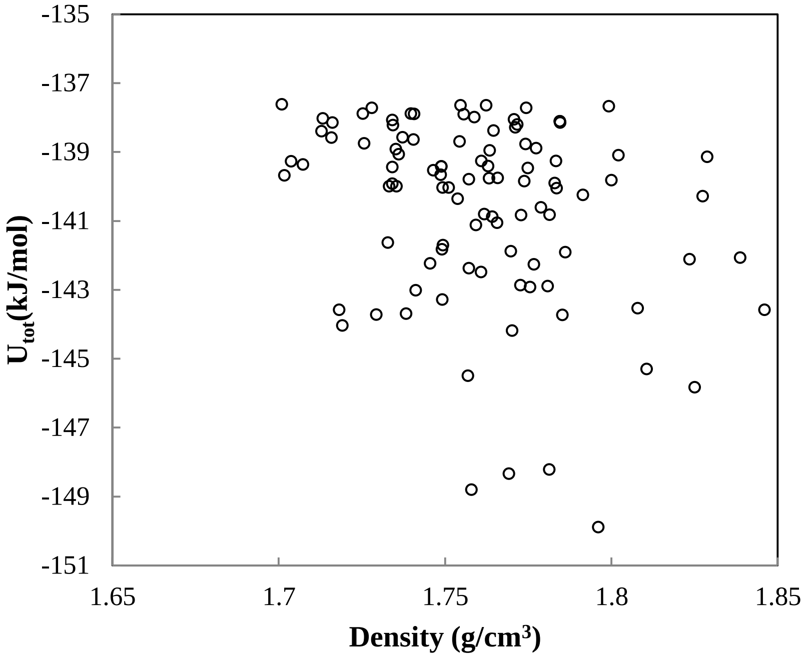

Figure 2 - Lattice energy (U_{tot}) vs density for 100 most stable structures for Molecule XXII

***A posteriori* analysis**

The experimental form of XXII was found as the global minimum structure in Step 2. After refinement of the lowest energy structures in Step 3, the experimental structure ranked 6th, 4.4 kJ/mol above the global minimum.

A posteriori, an analysis of the impact of flexibility, based on fully-atomistic minimisation in Step 3, reveals that the flexibility has an impact on structure, with the $rmsd_1$ going from 0.066 Å (rigid) to 0.026 Å (flexible). Most importantly, it has a significant impact on energy, with a stabilisation of approximately 20 kJ/mol for the experimental structure. The experimental structure is then found to be 11th, 7.26 kJ/mol above the global minimum.

1.3 Molecule XXIII (Sugden, Adjiman, Pantelides)

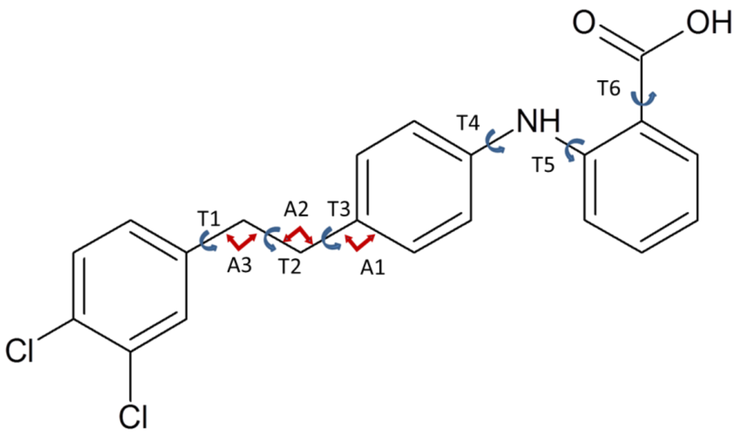

Figure 3. Flexible torsions and angles considered in Molecule XXIII

Key information on the application of the CSP approach to Molecule XXIII is reported in Figure 3, Table 4 and Table 5. A summary of the number of structures identified is presented in Table 6 and the energy landscape with the 100 lowest energy structures is shown in Figure 4.

Step 1. Gaussian single point minimisations were used to investigate flexibility within the molecule, and two torsional groups were identified. The three torsions around carbon-carbon single bonds (T1, T2, T3 in Figure 3) and the three torsions around carbon to nitrogen single bonds and the benzene to acid group in the fenamate group (T4, T5, T6) were found to be most important in capturing the impact of flexibility on lattice energy.

Step 2. Two torsion groups were defined for CrystalPredictor I (Karamertzanis & Pantelides, 2007): T1, T2, T3 as the first group and T4, T5, T6 as the second. The flexibility was taken into account by generating a grid based on constrained minimisations at fixed values of the flexible torsions, for each torsional group. The fixed values were chosen at regular intervals as described in Table 4.

The search space was sampled using CrystalPredictor I (Karamertzanis & Pantelides, 2007), since the use of torsional groups is under development in CrystalPredictor II (Habgood *et al.*, 2015). The search was made in the 59 most common space groups with Z'=1. Although it is known that at least one polymorph with Z'>1 has been identified experimentally, a search of structures with Z'>1 was not attempted.

Step 3. All minima with lattice energies within 20 kJ/mol of the global minimum as predicted in Step 2 (≈1500 structures) were further refined by CrystalOptimizer (Kazantsev et al., 2011), with additional flexibility in the C-C-C single bond angles (A1, A2 and A3 in Figure 3 and Table 4).

Table 4. Flexible torsions and angles in Molecule XXIII. Angles in italics are treated as flexible in Step 3 only

Identification	Gas phase value (°)	CrystalPredictor information			
		Spacing (°)	Grid Points	Torsion group	Domain (°)
T1	87.709	30	13	2	[0,360]
T2	179.559	30	13	2	[0,360]
T3	87.164	30	13	2	[0,360]
T4	-37.505	30	13	1	[0,360]
T5	167.479	30	8	1	[70,280]
T6	4.707	30	5	1	[-60,60]
A1	*111.968*	-	-	-	-
A2	*111.961*	-	-	-	-
A3	*120.45*	-	-	-	-

Table 5. Key information for each step of the CSP methodology for Molecule XXIII

**Step**	1	2	3
**Method**	Scans and CSD searches CSP	CrystalPredictor I	CrystalOptimizer
**Flexibility**	full	T1-6	T1-6, A1-3
**Level of theory**	M06/6-31G(d,p)	M06/6-31G(d,p)	M06/6-31G(d,p)
**Electrostatics**	N/A	RESP	DMA
**Dispersion/repulsion**	N/A	FIT	FIT
**No. of structures minimised**	N/A	450,000	1600

Table 6 - Number of structures found for Molecule XXIII, as a function of energy difference relative to the global minimum

	**Cutoff from global minimum (kJ/mol)**			
	**5**	**10**	**20**	**30**
**Step 2**	25	122	1,600	13,300
**Step 3**	47	172	570	758

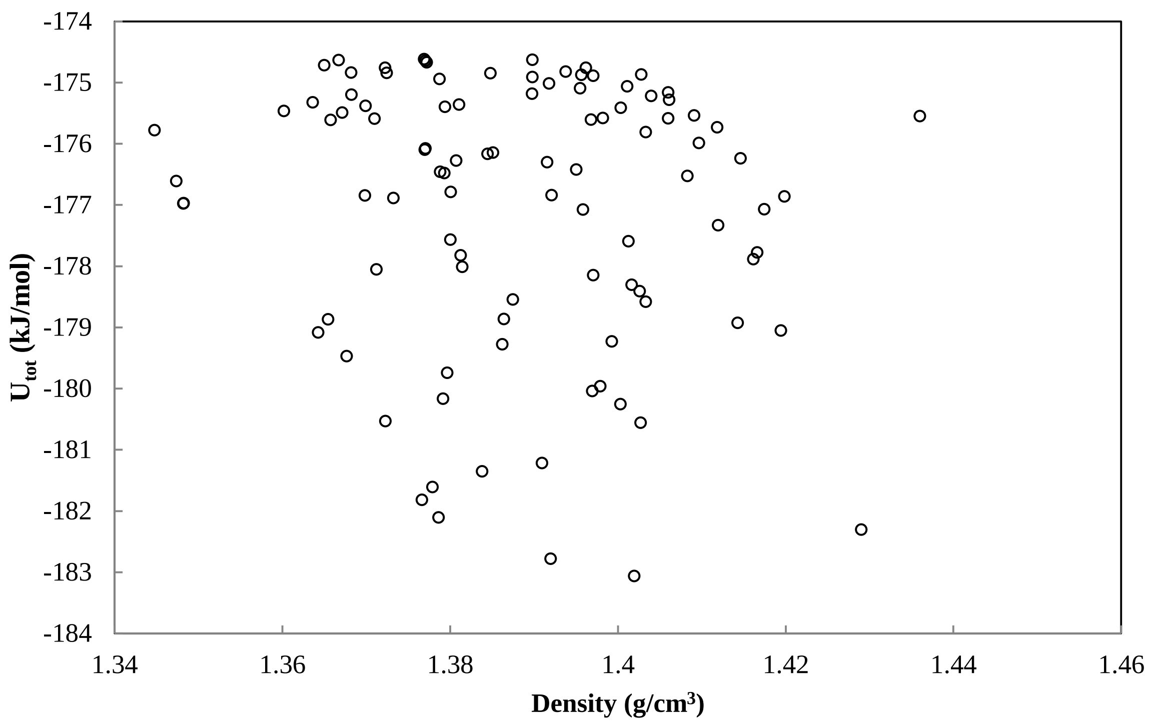

Figure 4. Lattice energy (U_{tot}) vs density for 100 most stable structures for Molecule XXIII.

A posteriori analysis

Forms *a* and *b* were found as 70th and 13th, respectively.

Form *d* was found as the structure with the 372nd lowest energy, with an $rmsd_{20}$ of 0.461. However, this energy is within 15 kJ/mol of the global minimum, with the polymorphic landscape in this instance being very dense. Several structures have been found multiple times (e.g., form *b* is a match to structures 13 and 25). With the application of a more aggressive clustering algorithm to remove duplicates, form *d* moves down to structure 167.

The two Z'=2 structures, forms *c* and *e*, have also been minimised, starting from the experimental structures and using the same model as in Step 3. Both are found to have low energies in the landscape. Form *c* is found to have an energy of -175.20 kJ/mol, -7.86 kJ/mol above the global minimum, and an $rmsd_{20}$ of 0.504, whilst form *e* is found to have an energy of -180.48 kJ/mol, -2.58 kJ/mol above the global minimum, and an $rmsd_{20}$ of 0.613.

1.4 Molecule XXV (Gatsiou, Sugden, Adjiman, Pantelides)

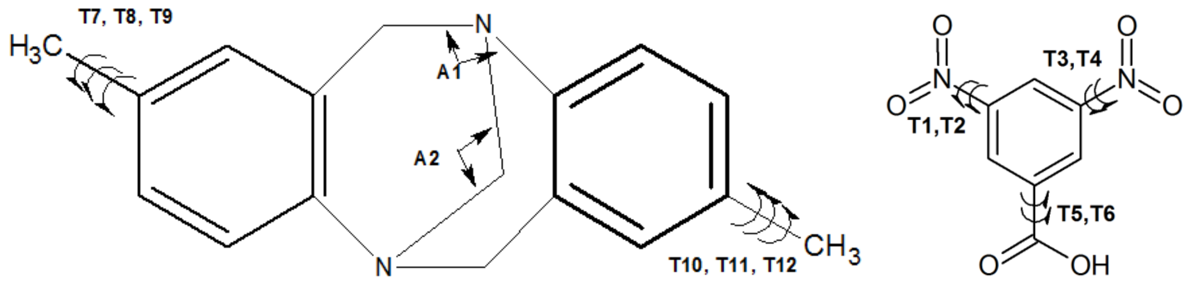

Figure 5. Flexible torsions and angles for co-crystal XXV

Key information on the application of the CSP approach to Molecule XXV is reported in Figure 5, Table 7 and Table 8. A summary of the number of structures identified is presented in Table 10 and the energy landscape with the 100 lowest energy structures is shown in Figure 6.

Step 1. A preliminary CrystalPredictor I search for the pure crystal of Troger's base showed that the experimental crystal is found within 15 kJ/mol of the global minimum. Troger's base was treated as rigid with its conformation fixed to the gas phase conformation with the relevant level of theory. Local minimizations of the experimental crystal of Troger's base (AXAGEL) with CrystalOptimizer, allowing different degrees of flexibility to the molecule each time led to minima very close in energy and geometry. Comparison of the minima showed small deviations in hydrogen positions of the methyl and two angles. The energetic impact of all these variations was less than 1 kJ/mol, allowing the molecule to be treated as rigid in the more computationally expensive global search stage. For dinitrobenzoic acid, the three torsions were treated as flexible (T1, T3, T5 in Figure 6).

Step 2. Crystal Predictor I was used. The flexibility of dinitrobenzoic acid was described by splitting the molecule into 3 groups, each with one torsion, so that the variation of the three torsions could be captured by computing three independent 1-dimensional grids of constrained minimisations (Table 7), as input to CrystalPredictor I.

Table 7. Flexible torsions and angles for co-crystal XXV. Angles and torsions in italics are treated as flexible in Step 3 only.

Identification	Gas phase value (°)	CrystalPredictor information			
		Spacing (°)	Grid Points	Torsion group	Domain (°)
T1	179.77	30	5	1	[-60,60]
T3	179.90	30	5	2	[-60,60]
T5	-0.048	30	5	3	[-60,60]
T2	*-0.24*	-	-	-	-
T4	*-0.09*	-	-	-	-
T6	*179.96*	-	-	-	-
T7	*10.18*	-	-	-	-
T8	*-130.47*	-	-	-	-
T8	*-9.89*	-	-	-	-
T10	*121.98*	-	-	-	-
T11	*-118.73*	-	-	-	-
T12	*1.60*	-	-	-	-
A1	*113.23*	-	-	-	-
A2	*111.75*	-	-	-	-

An unusually large number of low-energy structures were generated, with 27,389 structures within 20 kJ/mol of the global minimum and nearly 4,000 within 15 kJ/mol.

Step 3: Flexibility was introduced for Troger's base in the refinement stage, allowing rotation of the two methyl groups and bending of two angles Table 7. Given the large number of structures, a re-ranking based on a single iteration of CrystalOptimizer was carried out in Step 3a, for just over 3000 structures, following the approach of Vasileiadis *et al.* (2015). The lowest 500 structures were then fully minimized with CrystalOptimizer.

Table 8 - Key information for each step of the CSP methodology for co-crystal XXV.

**Step**	1	2	3a	3b
**Method**	Scans and CSD searches CSP for Troger's base	CrystalPredictor I	CrystalOptimizer One iteration only	CrystalOptimizer
**Flexibility**	CSD local minimisations with Crystal Optimizer	T1, T3, T4	T1, T2, T3, T4, T5, T6, T7, T8, T9, T10, T11, T12, A1, A2	T1, T2, T3, T4, T5, T6, T7, T8, T9, T10, T11, T12, A1, A2
**Level of theory**	None	M06/6-31G(d,p)	M06/6-31G(d,p)	M06/6-31G(d,p)
**Electrostatics**	Several See text	RESP	DMA	DMA
**Dispersion/repulsion**	Multipoles	FIT	FIT	FIT
**No. of structures minimised**	FIT	500,000	3,079	500

Table 9 - Number of structures found for Molecule XXV, as a function of energy difference relative to the global minimum.

	**Cutoff from global minimum (kJ/mol)**			
	**5**	**10**	**15**	**20**
**Step 2**	18	337	3,978	27,389
**Step 3a**	2	2	9	41
**Step 3b**	1	2	17	71

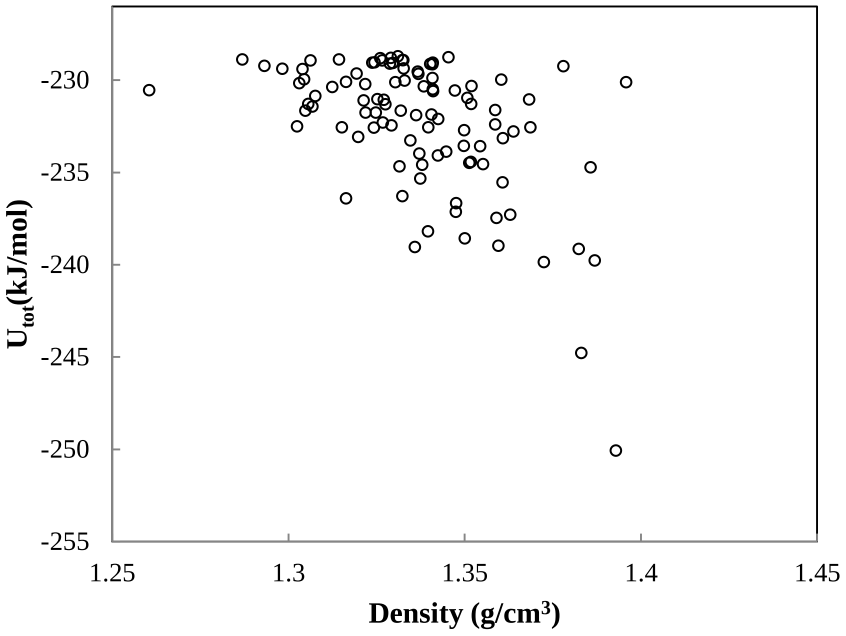

Figure 6 - Lattice energy (U_{tot}) vs density for the 100 most stable structures for co-crystal XXV

***A posteriori* analysis**

The experimental form of XXV was predicted as the lowest energy structure both in Step 2 and Step 3. In Step 2 the experimental form was found to be 4 kJ/mol lower in energy than the second lowest and it was found 6 kJ/mol below the second lowest after Step 3. The use of different lattice energy models between Step 2 and Step 3 did not affect the rank of the experimental structure.

1.5 Molecule XXVI (Sugden, Adjiman, Pantelides)

Key information on the application of the CSP approach to Molecule XXVI is reported in Figure 7 and Table 10 and Table 11. A summary of the number of structures identified is presented in Table 12 and the energy landscape with the 100 lowest energy structures is shown in Figure 8.

Step 1. The first stage for analysis of this molecule was to identify 7 flexible torsions (Figure 7 and Table 10).

Step 2. Since the flexible torsions could not be separated into torsional groups, CrystalPredictor II (Habgood et al., 2015) was used. A uniform grid of LAMs with constrained minimisations at fixed values of the flexible torsions, at the HF/6-31G(d,p) level of theory was generated. Only one region (relative to the conformation of ketone groups) was sampled, as other conformations appeared to lead to highly strained structures due to the rigid

benzene groups. Due to computational limitations, only a relatively small (150,000 points) search was completed.

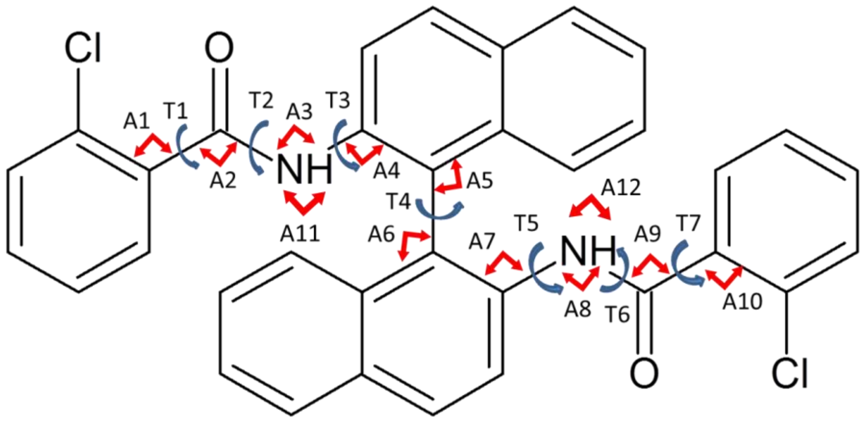

Figure 7. Flexible torsions and angles in molecule XXVI

Table 10 - Flexible torsions and angles in Molecule XXVI. Angles in italics are treated as flexible in Step 3 only

Identification	Gas phase value (º)	CrystalPredictor information		
		Spacing (º)	Grid Points	Domain(º)
T1	72.66	30	12	[-180,180]
T2	177.5	30	1	[165,195]
T3	-128.2	30	3	[-185,-95]
T4	-94.32	30	2	[-115,-55]
T5	-128.60	30	3	[-185,-95]
T6	177.61	30	1	[165,195]
T7	72.87	30	12	[-180,180]
A1	*119.7*	-	-	-
A2	*113.0*	-	-	-
A3	*128.6*	-	-	-
A4	*117.8*	-	-	-
A5	*119.4*	-	-	-
A6	*121.2*	-	-	-
A7	*117.7*	-	-	-
A8	*128.6*	-	-	-
A9	*113.0*	-	-	-
A10	*119.7*	-	-	-
A11	*116.5*	-	-	-
A12	*114.8*	-	-	-

The search was made in the 59 most common space groups with Z’=1 (axial chirality was not treated, due to computational limitations).

Step 3. The 713 lowest energy structures were further optimised using CrystalOptimizer with intramolecular energy and conformational multipoles calculated at the PBE1PBE 6-31G(d,p) level of theory.

Table 11. Key information for each step of the CSP methodology for Molecule XXVI

**Step**	1	2	3
**Method**	Scans and CSD searches CSP	CrystalPredictor I	CrystalOptimizer
**Flexibility**	Full	T1-7	T1-7, A1-12
**Level of theory**	M06/6-31G(d,p)	HF/6-31G(d,p)	PBE1PBE/6-31G(d,p)
**Electrostatics**	N/A	RESP	DMA
**Dispersion/repulsion**	N/A	FIT	FIT
**No. of structures minimised**	N/A	150,0000	713

Table 12. Number of structures found for Molecule XXVI, as a function of energy difference relative to the global minimum

	**Cutoff from global minimum (kJ/mol)**			
	**5**	**10**	**20**	**30**
**Step 2**	18	40	152	998
**Step 3**	4	10	94	376

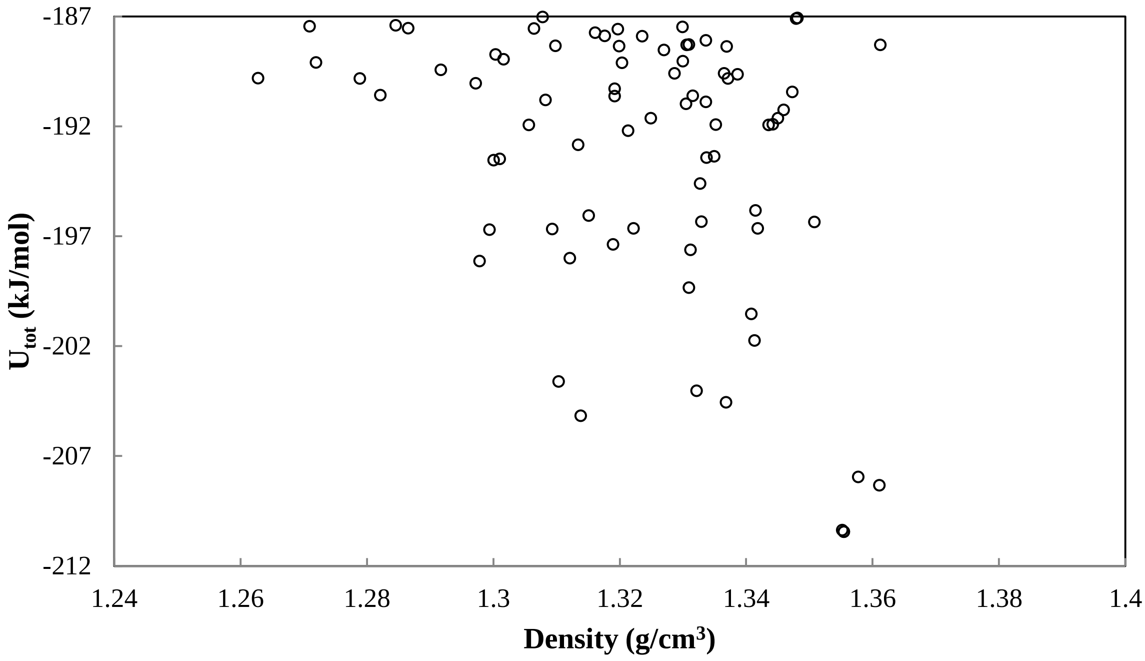

Figure 8. Lattice energy (U_{tot}) vs density for 100 most stable structures for Molecule XXVI

***A posteriori* analysis**

Molecule XXVI was not found in our search, for the simple reason that the search was insufficiently broad; as noted in our submission to the blind test, we left insufficient time to complete a full search. A new feature of the CrystalPredictor II program is to evaluate the intramolecular energy at the edge of the search space, and output a warning message if this energy is below 10 kJ/mol, indicating to the user that the search space should be expanded to account reliably for flexibility. This issue was identified during the Molecule XXVI run, but in order to meet the deadline, a reduced search space was considered. After the blind test, QM calculations were completed to extend the search space (symmetrically) to include the experimental conformation, necessitating a further ≈350k CPU hours expenditure. Although the edges of the search space are still not uniformly above 10kJ/mol, a search of 100,000 structures finds the experimental form predicted to be 18th lowest energy structure at Step 2, and the global minimum at Step 3. This suggests that the methods are sound, but efficiency could be improved.

1.6 Computational resources used

Table 13. Estimation of CPU hours expended in each investigation. (Most typically on Intel(R) Xeon(R) CPU E5-2660 v2 running at 2.20GHz)

**Time (CPU hours)**	**MOLECULE XXII**	**MOLECULE XXIII**	**MOLECULE XXV**	**MOLECULE XXVI**
**Step 1**	270	Relatively negligible	212	Relatively negligible
**Step 2**				
QM calculations	18	~30000	30	200,000
Global search	44	5,500	228	12,500
**Step 3**	1	51,079	3a: 5,199<br>3b: 31,866	60,000
**Total for CSP**	**333**	**87,000**	**37,535**	**272,500**

Bibliography

Cox, S. R., Hsu, L. Y. & Williams, D. E. (1981). *Acta Crystallogr A* **37**, 293-301.

Habgood, M., Sugden, I. J., Kazantsev, A. V., Adjiman, C. S. & Pantelides, C. C. (2015). *J Chem Theory Comput* **11**, 1957-1969.

Karamertzanis, P. G. & Pantelides, C. C. (2005) *J. Comp. Chem.* **26**, 304–324.

Karamertzanis, P. G. & Pantelides, C. C. (2007). *Mol Phys* **105**, 273-291.

Kazantsev, A. V., Karamertzanis, P. G., Adjiman, C. S. & Pantelides, C. C. (2011). *J Chem Theory Comput* **7**, 1998-2016.

Lommerse, J. P. M., Motherwell, W. D. S., Ammon, H. L., Dunitz, J. D., Gavezzotti, A., Hofmann, Detlef W. M., Leusen, Frank J. J., Mooij, W. T. M., Price, S. L., Schweizer, B. and Schmidt, M. U. (2000). *Acta Crystallographica Section B* **56**, 697--714.

Pantelides, C. C., Adjiman, C. S. & Kazantsev, A. V. (2014). *Top Curr Chem* **345**, 25-58.

Price, S. L. a. L., Maurice and Welch, Gareth W. A. and Habgood, Matthew and Price, Louise S. and Karamertzanis, Panagiotis G. and Day, Graeme M. (2010). *Phys. Chem. Chem. Phys.* **12**, 8478-8490.

Vasileiadis M., Pantelides. C. C, Adjiman C. S.. (2015). *Chem. Eng. Sci.* **121**, 60 - 76.

Williams, D. E. (1984). *Acta Crystallogr A* **40**, C95-C95.

Williams, D. E. & Starr, T. L. (1977). *Comput Chem* **1**, 173-177.
