# Supplementary material for: Report on the sixth blind test of organic crystal structure prediction methods
Source: Acta Crystallogr B Struct Sci Cryst Eng Mater. 2016 Aug 1;72(Pt 4):439–59. doi: 10.1107/S2052520616007447 (PMC4971545; doi:10.1107/S2052520616007447)
Supplement: Supplementary file 3 [file b-72-00439-sup3.zip › Group_16_Pickard_et_al.pdf]

Crystal structure of molecule XXII: The 2015 CCDC Blind Test of Organic Crystal Structure Prediction

Chris J. Pickard,[1,2] Bartomeu Monserrat,[3,4] Alston J. Misquitta,[5] and Richard J. Needs[3]

[1]*Department of Materials Science & Metallurgy, University of Cambridge, 27 Charles Babbage Road, Cambridge CB3 0FS, United Kingdom*
[2]*Department of Physics and Astronomy, University College London, Gower St., London WC1E 6BT, United Kingdom*
[3]*Cavendish Laboratory, 19, J J Thomson Avenue Cambridge CB3 0HE, United Kingdom*
[4]*Department of Physics and Astronomy, Rutgers University, Piscataway, New Jersey 08854-8019, USA*
[5]*School of Physics and Astronomy, Queen Mary University of London, London E1 4NS, United Kingdom*

(Dated: August 31, 2015)

I. INTRODUCTION

We have used a range of state-of-the-art search techniques and energy and free-energy methods to predict the crystal structure of molecule XXII. As we have learned from past Blind Tests, in order to successfully predict a crystal structure for a molecular system, not only do we need to have a good search method, but we also need to use a sufficiently accurate energy model. Our structure searches are based on first principles methods. Furthermore, as molecular crystals are generally grown at room temperature, vibrational contributions should be included. In a polymorphic system, kinetic effects could play a role in determining which polymorph is obtained, but these effects are as yet difficult to account for and we have made no attempt to do so here.

II. METHOD 1: AIRSS

Ab initio random structure searching, or AIRSS [1], is a powerful searching method that method that has led to the discovery of new structures subsequently verified by experiment in materials ranging from ammonia [2, 3], ammonia hydrates [4, 5], aluminium hydride [6], silane [7], hydrogen [8, 9] to xenon oxides [10]. Within the AIRSS method a cell shape and volume are selected at random, atoms are added at random positions to provide the desired stoichiometric composition, and the cell shape and atomic positions are relaxed until the atomic forces and pressure take the required value, which is zero in this case. This procedure is repeated many times, giving a relatively sparse sampling of the "structure space". We also introduce other biases such as using starting structures with simple bonding motifs. We constrain the minimum separations between atom pairs (C–C, N–N, S–S, C–N, C–S, N–S) in the initial structures, which helps to space out the atoms appropriately, while retaining a high degree of randomness. The minimum distances are obtained from preliminary short AIRSS runs. In almost all of the subsequent searches we impose symmetry constraints on the structures which are maintained throughout the relaxation. Using symmetry constraints greatly reduces the size of the structure space that must be searched, although they also break up the space into disconnected regions which can prevent some structures from relaxing fully. An additional useful bias is the choice of space group symmetries imposed which can be selected, for example, at random with a probability proportional to the frequencies of occurrence [11] in different system types.

Under extreme conditions of high pressure, for example, there is little to guide theoretical methods and random structure searching has proved to be an excellent means of exploring the potential energy landscape. In this sense AIRSS can be used as an *extrapolative* method for investigating structures under conditions in which the chemistry is unknown. The exploratory nature of AIRSS was manifest in the fifth Blind Test [12] when we found out that molecule XVI was able to undergo a conformational change into a considerably more stable from.

III. METHOD 2: HARMONIC AND ANHARMONIC CORRECTIONS

The vibrational energy was evaluated at both the harmonic and anharmonic levels. In both cases, the energy $E_{\mathbf{S}}$ associated with the state $|\Phi_{\mathbf{S}}\rangle$ was used to evaluate the zero-point energy, corresponding to the vibrational ground state, and the free energy at temperature T:

$$\mathcal{F} = -k_{\mathrm{B}}T \ln \mathcal{Z}, \tag{1}$$

where $\mathcal{Z} = \sum_{\mathbf{S}} e^{-E_{\mathbf{S}}/k_{\mathrm{B}}T}$ is the partition function, and k_{B} is Boltzmann's constant.

At the harmonic level, the finite displacement method was used to construct the matrix of force constants, which was subsequently Fourier transformed to obtain the dynamical matrices over the vibrational Brillouin zone. We obtained the harmonic frequencies by diagonalising the dynamical matrices.

At the anharmonic level, we mapped the Born-Oppenheimer energy surface beyond the local quadratic region using the principal axes approximation as described in Ref. [13]. The resulting anharmonic vibrational equations of motion were solved within a mean-field ansatz, the so-called vibrational self-consistent field method. This approach has accurately described anharmonic vibrational energies in molecular systems such as hydrogen [14] and ice [15].

The mapping of the Born-Oppenheimer energy surface (at the harmonic and anharmonic levels) is carried out using the plane-wave density functional theory code CASTEP [16]. The exchange-correlation functional was described at the PBE level, including dispersion corrections using the TS scheme [17] and MBD scheme [18]. Plane-wave energy cut-off energies of 800 eV and Monkhorst-Pack Brillouin zone grids of spacing $2\pi \times 0.03$ Å^{-1} were used. The default "on-the-fly" pseudopotentials were used.

For the anharmonic calculations, 15 points were sampled along each normal mode direction, and cubic splines were fitted to the first-principles data. The VSCF equations were solved by expanding the vibrational anharmonic wave function in a basis of simple harmonic oscillator eigenstates, with 20 states per degree of freedom.

TABLE I. SAPT-DFT interaction energy components for a dimer extracted from the lowest-energy Pna21 crystal structure. SAPT-DFT calculations have been performed using the CamCASP program. All energies are reported in eV.

Component	Energy / eV
$E^{(1)}_{\mathrm{elst}}$	−0.321
$E^{(1)}_{\mathrm{exch}}$	0.754
$E^{(2)}_{\mathrm{POL}}$	−0.054
$E^{(2)}_{\mathrm{CT}}$	−0.006
$E^{(2)}_{\mathrm{DISP}}$	−0.682
$E^{(2)}_{\mathrm{tot}}$	−0.308

IV. CHOICE OF FUNCTIONALS

By design, the PBE functional is probably one of the least biased density functionals: the parameters in this functional have been obtained theoretically, without the use of any empirical fitting. It will however suffer from the shortcomings of the GGA class of functionals, in particular, PBE is unable to correctly model dispersion interactions and can result in sizable errors for systems with a significant degree of charge transfer. By applying an appropriate dispersion correction it is possible to significantly reduce the error made in systems such as XXII that are significantly dispersion-bound. Nevertheless, the dispersion correction is not unique and via the damping function and choice of dispersion model, we introduce a degree of empiricism to the method. At present there is little to be done about this. Instead, we will attempt to develop an idea of the uncertainty associated with the choice of dispersion correction by using a variety of types of dispersion models.

To gain insight into the nature of the interaction in this system we performed a SAPT-DFT (symmetry adapted perturbation theory) [19, 20] interaction energy analysis of a selection of dimers from the crystal. These calculations were performed using the CamCASP program [21] with orbitals and eigenvalues evaluated using the PBE/AC (PBE with asymptotic correction) functional [22] with the NWChem program [23]. We used a Sadlej-pVTZ basis in the MC+ format with a 3s2p1d mid-bond set. The ALDA (adiabatic LDA) response kernel [19] was used for the second-order energies. From the interaction energies reported in Table I we can see that the dominant source of binding (at the two-body level) is the second-order dispersion interaction. The electrostatic and polarization contributions are smaller in magnitude than the exchange-repulsion and, with the absence of hydrogen atoms, the second-order charge-transfer component evaluated using regularised SAPT-DFT [24] is sufficiently small to be neglected. The small charge-transfer suggests that we should not expect significant errors associated with the self-interaction seen in standard GGAs, and the PBE functional should suffice. The importance of the dispersion interaction for this system clearly indicates that we should expect to see a strong dependence on the choice of dispersion model. As indicated by a basis-space version of the iterated stockholder atoms (ISA) procedure [25, 26], there is significant charge movement in molecule XXII, particularly around the nitrogen atoms. This is perhaps not a surprise, but it does indicate that the static dispersion coefficients used in the Grimme D2 method may not be suitable. A better choice would be the TS scheme [17] which uses Hirshfeld partitioning to capture some of the *local* effects of the charge delocalisation. However the TS dispersion model does not capture the through-space polarization that leads to the polarizability anisotropy, which is substantial in this molecule. The static average dipole–dipole polarizability of molecule XXII is 191 a.u. and the polarizability anisotropy is 157 a.u. This anisotropy is entirely missing in the TS (or Grimme) scheme, but it can be recovered using methods that account for through-space polarization such as those that derive the dispersion coefficients from the frequency-dependent density response function [27], or through models such as the MBD scheme of Tkatchenko *et al.* [18]. There are additional effects associated with plasmon-like modes [28, 29] to the dispersion, but these are expected to be negligible in this system. We have used all three dispersion models and expect that the most accurate results should be those from PBE+MBD. However, it should be appreciated that any dispersion correction is only as good as the splicing (damping) scheme used in the overlap region. There will always be an uncertainty associated with the splicing model used, but we have not investigated this aspect of the problem.

As mentioned earlier, the choice of molecular conformation and close-packing parameters can bias the set of randomly generated structures that are subsequently relaxed using DFT. We reduce this bias as follows:

- $Z = 1$: Place the unoptimized molecular unit randomly in the cell. Relax all atomic positions.
- Extract molecular conformation and intermolecular distance matrix.
- Repeat steps to generate a statistically significant range of conformations and intermolecular distance matrices.
- $Z = 2$: Generate the initial random cell using molecular conformations and separations from the previous step. Relax.

TABLE II. Relative lattice energies for a selection of stable crystal structures from the 0K AIRSS searches with PBE and various dispersion corrections. Relative lattice energies, $\Delta E_{\rm latt}^{\rm static}$, are reported in meV/f.u. (formula unit) and cell volumes, V, are reported in Å^3/f.u.

Structure name	Space Group	Z	PBE+G06			PBE+TS			PBE+MBD		
			Rank	V	$\Delta E_{\rm latt}^{\rm static}$	Rank	V	$\Delta E_{\rm latt}^{\rm static}$	Rank	V	$\Delta E_{\rm latt}^{\rm static}$
7153-3605-15	Pna21	4	1	235.55	0	1	240.90	0	3	240.40	0
30795-5511-6	P212121	4	2	234.59	25	2	242.35	38	5	241.25	19
9368-9830-4	P21/c	4	4	233.03	33	3	241.37	45	4	240.17	12
1408-4781-4	Pmn21	2	8	237.33	42	13	245.62	58	18	249.09	103
9314-8768-3	P21	2	17	232.29	60	9	240.08	50	2	240.16	-6
9037-4-8	P21	2	18	232.83	61	10	240.03	51	1	240.22	-8

TABLE III. Zero-point energies for some of the lower energy crystal structures evaluated at 0K and 300K. Harmonic ZPE corrections have been calculated as described in the text. Harmonic vibrational energies at temperature T K, $E_{\rm vib}^{\rm har}(T)$, are reported in eV/f.u.

Structure name	Space Group	Z	PBE+TS		PBE+MBD	
			$E_{\rm vib}^{\rm har}(0)$	$E_{\rm vib}^{\rm har}(300)$	$E_{\rm vib}^{\rm har}(0)$	$E_{\rm vib}^{\rm har}(300)$
7153-3605-15	Pna21	4	1.3494	1.7590	1.7606	1.3311
30795-5511-6	P212121	4	1.3308	1.7581	1.7617	1.3471
9368-9830-4	P21/c	4	1.3654	1.7575	1.7621	—
1408-4781-4	Pmn21	2	1.3642	1.7555	—	—
9314-8768-3	P21	2	—	—	1.7635	—
9037-4-8	P21	2	—	—	1.7641	—

- Update the list of molecular conformations and separations using data from the $Z = 2$ searches.
- $Z > 2$: Proceed as above.

In this manner, we have eliminated, or at least significantly reduced any bias associated with the randomly generated cells. This procedure also ensures that we use molecular conformations appropriate for the condensed phase. Further, it should be apparent that the above algorithm naturally adapts if, say, pressure is applied to the cell. This method is expected to be superior to the standard approach of using a gas-phase optimized molecular conformation which may not always be appropriate for the condensed phase, particularly for conformationally flexible molecules, or for systems under pressure.

Our searches were performed using the CASTEP [16] code for the lattice energy minimisation. This was done using the Perdew-Burke-Ernzerhof (PBE) generalised gradient approximation density functional [30] with Vanderbilt ultrasoft PBE pseudopotentials for C, N, and S, and a plane wave cutoff of 350 eV and a Monkhorst-Pack Brillouin zone sampling grid spacing of 0.1 Å^{-1}. The Grimme D2 [31] dispersion correction was used.

The numerical results reported here were obtained using the default "on-the-fly" pseudopotentials, plane-wave energy cut-off energies of 800 eV and Brillouin zone grids of spacing $2\pi \times 0.03$ Å^{-1} were used. These calculational parameters provide significantly better converged results than obtained in the searching. We performed calculations using the PBE+G06, PBE+TS [17] and PBE+MBD [18] methods.

We were able to perform a complete search of space groups with one and two molecules in the unit cell ($Z = 1, 2$). For $Z = 2$ around 1000 structures were optimized. For $Z > 2$, rather than doing a full relaxation for all randomly generated initial structures, we evaluated single point lattice energies (unrelaxed) for these structures, and relaxed only those structures in the lowest 10% of the energy range. This step was repeated several times, after which both the list of molecular conformations and intermolecular distance matrices were updated. Note that although the space groups were chosen randomly, the packing subject to distance constraints naturally picks out the common space groups. In all cases we searched for crystals with $Z' = 1$.

V. RESULTS

Harmonic vibrational energies for selected crystal structures calculated at 0K and 300K with both PBE+TS and PBE+MBD are reported in Table III. In Table IV we report *relative* free energies for these structures. In both cases (PBE+TS and PBE+MBD), including the vibrational contribution does not change the ordering of the structures presented in these tables. Due to the numerical difficulties encountered in optimising these structures we expect the 300K vibrational contributions to be uncertain by as much as 20 meV/f.u. consequently these energies must be

TABLE IV. Same as Table III but with ZPE/free energies displayed relative to those from structure 7153-3605-15. The numerical uncertainties on the ZPE energies are expected to be around 1meV/f.u. while that on the vibrational energy at 300K energies is likely larger, particularly for the PBE+MBD functional as these were not quite as well relaxed.

Structure name	Space Group	Z	PBE+TS				PBE+MBD			
			$\Delta E_{\text{vib}}^{\text{har}}(0)$	$\Delta F(0)$	$\Delta E_{\text{vib}}^{\text{har}}(300)$	$\Delta F(300)$	$\Delta E_{\text{vib}}^{\text{har}}(0)$	$\Delta F(0)$	$\Delta E_{\text{vib}}^{\text{har}}(300)$	$\Delta F(300)$
7153-3605-15	Pna21	4	0	0	0	0	0	0	0	0
30795-5511-6	P212121	4	-19	19	-1	37	1	20	16	35
9368-9830-4	P21/c	4	16	61	-2	43	2	14	—	—
1408-4781-4	Pmn21	2	15	73	-4	54	—	—	—	—
9314-8768-3	P21	2	—	—	—	—	3	-3	—	—
9037-4-8	P21	2	—	—	—	—	4	-4	—	—

considered with caution. We have also evaluated the anharmonic vibrational contribution for the Pna21 structure. At −6 meV/f.u. it is smaller, but comparable to the harmonic free-energy differences between the structures, and consequently may alter the energetic ordering of crystal structures. It is likely that the anharmonic corrections are not very large as molecule XXII does not contain any light atoms. By far, the largest effect arises from the use of the MBD dispersion correction. From Table II we see that the MBD correction significantly changes the energetic ranking of the crystal structures compared with PBE+TS. In particular, two of the high ranking structures with PBE+TS (9314-8768-3 at rank 9 and 9037-4-8 at rank 10) are found to be the most stable with PBE+MBD, with the MBD correction resulting in a change of nearly 60 meV/f.u. As with PBE+TS, including the harmonic vibrational contribution does not lead to a further change in the ordering of these structures.

We have found that molecule XXII is not rigid. There is a very small conformational energy penalty paid to flatten out molecule XXII. The C–C–S–C dihedral angle in the central ring of the molecule is 23° in structure 7153-3605-15, but it is only 1.8° in structure 9314-8768-3 and is less that one degree in structure 9037-4-8. The conformational energy difference between these two structures is only 3 meV/f.u. when evaluated using the PBE functional and a Dunning aug-cc-pVTZ basis set. Including range-separation to minimise the effects of the self-interaction error does not seem to alter these results. However, there is a substantial increase in the molecular dipole–dipole polarizability in the flat structure. The average polarizability increases by 4 a.u. to 195 a.u. and the polarizability anisotropy increases by 14 a.u. to 171 a.u. The dispersion coefficient will show an even larger increase. This increase in polarizability will, at least in part, be captured by the MBD model and is probably the reason for the increase in stability of structures 9314-8768-3 and 9037-4-8, both of which are formed from molecules in the flat conformation.

Given the uncertainties in our search method (in particular, the uncertainty introduced by not using the MBD model in the initial searches), and uncertainties in the static energy (while we regard MBD as the most convincing dispersion model from the ones we used, it includes dipole–dipole interactions only and higher-order terms are missing, further, there will always be uncertainties in the splicing scheme used, but this should not affect the relative order of the structures) and free energy calculations, we conclude that structures 9314-8768-3, 9037-4-8, and 7153-3605-15 must all be considered as candidate structures on an equal footing. Structures 9314-8768-3 and 9037-4-8 are very similar, and may well be identical. We note that the small energy differences between the three lowest energy structures may also suggest that the XXII molecule may crystallise in more than one form, depending on the C–C–S–C dihedral angle in the central ring, that is, this system may be polymorphic.

VI. COMPUTATIONAL RESOURCES USED

The following computational resources were used for this project (all times are reported in core-hours):

- AIRSS: 240,000
- ZPE, harmonic (4 structures): 40,000
- ZPE, anharmonic (1 structure): 100,000
- SAPT-DFT & polarizability calculations: 250
- Geometry optimizations and DFT energy evaluations for the monomers: 150

We estimate that, in all, we have used a total of around 380,000 core-hours. The bulk of this was spent on the AIRSS searches and the anharmonic ZPE correction.

[1] C. J. Pickard and R. J. Needs, J. Phys.: Condens. Matter **23**, 053201 (2011).

[2] S. Ninet, F. Datchi, P. Dumas, M. Mezouar, G. Garbarino, A. Mafety, C. J. Pickard, R. J. Needs, and A. M. Saitta, Phys. Rev. B **89**, 174103 (2014).

[3] C. J. Pickard and R. J. Needs, Nature Materials **7**, 775 (2008).

[4] A. D. Fortes, E. Suard, M. Lemee-Cailleau, C. J. Pickard, and R. J. Needs, J. Am. Chem. Soc. **131**, 13508 (2009).

[5] G. I. G. Griffiths, A. D. Fortes, C. J. Pickard, and R. J. Needs, The Journal of Chemical Physics **136**, 174512 (2012).

[6] C. J. Pickard and R. J. Needs, Phys. Rev. B **76**, 144114 (2007).

[7] C. J. Pickard and R. J. Needs, Phys. Rev. Lett. **97**, 045504 (2006).

[8] C. J. Pickard and R. J. Needs, Nature Physics **3**, 473 (2007).

[9] C. J. Pickard, M. Martinez-Canales, and R. J. Needs, Phys. Rev. B **85**, 214114 (2012).

[10] A. Dewaele, (2015), unpublished.

[11] B. P. van Eijck, A. L. Spek, W. Z. M. Mooij, and J. Kroon, Acta Cryst. B **54**, 291 (1998).

[12] D. A. Bardwell, C. S. Adjiman, Y. A. Arnautova, E. Bartashevich, S. X. M. Boerrigter, D. E. Braun, A. J. Cruz-Cabeza, G. M. Day, R. G. D. Valle, G. R. Desiraju, B. P. van Eijck, J. C. Facelli, M. B. Ferraro, D. Grillo, M. Habgood, D. W. M. Hofmann, F. Hofmann, K. V. J. Jose, P. G. Karamertzanis, A. V. Kazantsev, J. Kendrick, L. N. Kuleshova, F. J. J. Leusen, A. V. Maleev, A. J. Misquitta, S. Mohamed, R. J. Needs, M. A. Neumann, D. Nikylov, A. M. Orendt, R. Pal, C. C. Pantelides, C. J. Pickard, L. S. Price, S. L. Price, H. A. Scheraga, J. van de Streek, T. S. Thakur, S. Tiwari, E. Venuti, and I. K. Zhitkov, Acta Cryst. B **67**, 535 (2011).

[13] B. Monserrat, N. D. Drummond, and R. J. Needs, Phys. Rev. B **87**, 144302 (2013).

[14] N. D. Drummond, B. Monserrat, J. H. Lloyd-Williams, P. L. Ríos, C. J. Pickard, and R. J. Needs, Nat. Commun. **6**, 7794 (2015).

[15] E. A. Engel, B. Monserrat, and R. J. Needs, Phys. Rev. X **5**, 021033 (2015).

[16] S. J. Clark, M. D. Segall, C. J. Pickard, P. J. Hasnip, M. J. Probert, K. Refson, and M. C. Payne, Zeitschrift fuer Kristallographie **220**, 567 (2005).

[17] A. Tkatchenko and M. Scheffler, Phys. Rev. Lett. **102**, 073005 (2009).

[18] A. Tkatchenko, R. A. DiStasio, R. Car, and M. Scheffler, Phys. Rev. Lett. **108**, 236402 (2012).

[19] A. J. Misquitta, R. Podeszwa, B. Jeziorski, and K. Szalewicz, J. Chem. Phys. **123**, 214103 (2005).

[20] A. J. Misquitta, B. Jeziorski, and K. Szalewicz, Phys. Rev. Lett. **91**, 33201 (2003).

[21] A. J. Misquitta and A. J. Stone, "CamCASP: a program for studying intermolecular interactions and for the calculation of molecular properties in distributed form," University of Cambridge (2013), accessed: July 2014.

[22] M. E. Casida and D. R. Salahub, J. Chem. Phys. **113**, 8918 (2000).

[23] E. J. Bylaska, W. A. de Jong, K. Kowalski, T. P. Straatsma, M. Valiev, D. Wang, E. Apra, T. L. Windus, S. Hirata, M. T. Hackler, Y. Zhao, P.-D. Fan, R. J. Harrison, M. Dupuis, D. M. A. Smith, J. Nieplocha, V. Tipparaju, M. Krishnan, A. A. Auer, M. Nooijen, E. Brown, G. Cisneros, G. I. Fann, H. Fruchtl, J. Garza, K. Hirao, R. Kendall, J. A. Nichols, K. Tsemekhman, K. Wolinski, J. Anchell, D. Bernholdt, P. Borowski, T. Clark, D. Clerc, H. Dachsel, M. Deegan, K. Dyall, D. Elwood, E. Glendening, M. Gutowski, A. Hess, J. Jaffe, B. Johnson, J. Ju, R. Kobayashi, R. Kutteh, Z. Lin, R. Littlefield, X. Long, B. Meng, T. Nakajima, S. Niu, L. Pollack, M. Rosing, G. Sandrone, M. Stave, H. Taylor, G. Thomas, J. van Lenthe, A. Wong, and Z. Zhang, "NWChem, a computational chemistry package for parallel computers, version 5.0," Pacific Northwest National Laboratory, Richland, Washington 99352-0999, USA (2006).

[24] A. J. Misquitta, J. Chem. Theory Comput. **9**, 5313 (2013).

[25] T. C. Lillestolen and R. J. Wheatley, Chem. Commun. **2008**, 5909 (2008).

[26] A. J. Misquitta, A. J. Stone, and F. Fazeli, J. Chem. Theory Comput. **10**, 5405 (2014).

[27] A. J. Misquitta and A. J. Stone, Mol. Phys. **106**, 1631 (2008).

[28] A. J. Misquitta, J. Spencer, A. J. Stone, and A. Alavi, Phys. Rev. B **82**, 075312 (2010).

[29] A. J. Misquitta, R. Maezono, N. D. Drummond, A. J. Stone, and R. J. Needs, Physical Review B **89**, 045140 (2014).

[30] J. Perdew, K. Burke, and M. Ernzerhof, Phys. Rev. Lett. **77**, 3865 (1996), also see erratum, Phys. Rev. Lett. **78**, 1396 (1996).

[31] S. Grimme, J. Comp. Chem. **27**, 1787 (2006).
