# Supplementary material for: Report on the sixth blind test of organic crystal structure prediction methods
Source: Acta Crystallogr B Struct Sci Cryst Eng Mater. 2016 Aug 1;72(Pt 4):439–59. doi: 10.1107/S2052520616007447 (PMC4971545; doi:10.1107/S2052520616007447)
Supplement: Supplementary file 3 [file b-72-00439-sup3.zip › Group_17_Podeszwa_Post_Analysis.pdf]

Supplementary information for predictions of structure of the crystal built of molecule XXII

Wojciech Jankiewicz,[1] Michael P. Metz,[2] Rafał Podeszwa,[1] and Krzysztof Szalewicz[2]

[1]*Institute of Chemistry, University of Silesia, Szkolna 9, 40-006 Katowice, Poland*

[2]*Department of Physics and Astronomy, University of Delaware, Newark, Delaware 19716*

(Dated: July 7, 2016)

I. METHODOLOGY

The procedure consists of monomer optimization, potential (force-field) generation, molecular packing, energy minimization, and molecular dynamics simulation.

The intermolecular potentials were constructed using symmetry-adapted perturbation theory (SAPT)[1,2] based on Kohn-Sham density-functional theory (DFT) description of monomers, SAPT(DFT)[3,4], implemented in the SAPT2012[5] package, using the ORCA set of codes[6] for DFT calculations on monomers. The monomer geometry was obtained by an unconstrained PBE0+D3[7–9] minimization using Orca[6] and the aug-cc-pVTZ basis set[10]. It resulted in the bending angle around the S-S line in the six-membered ring of about 140.3° where 180° indicates an approximately flat monomer. The monomer energy was lowered by about 5.4 kcal/mol in this optimization. We have checked these findings by calculating the interaction energies with several other methods on the path along the bending angle (keeping all other geometric parameters the same as in the PBE0+D3 minimum). The results are shown in Fig. I. The highest-level theory, the coupled-cluster method with single, double,

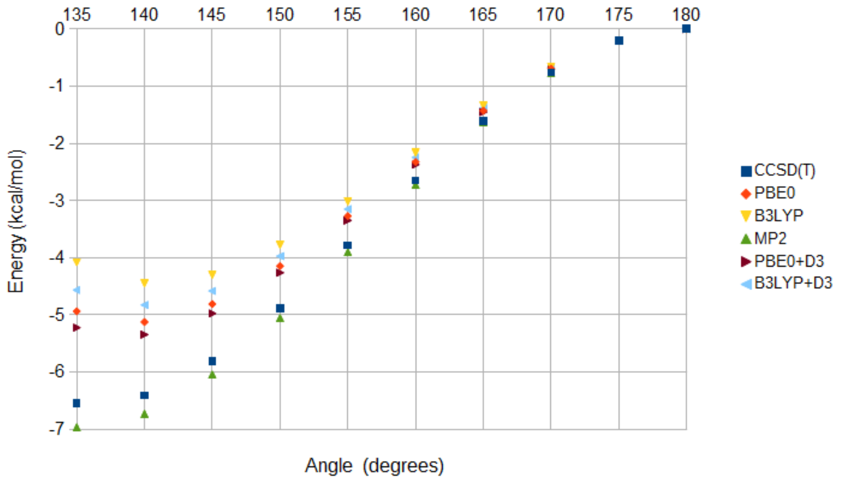

FIG. 1. Comparison of the dependence of monomer energy on the S-S bending angle γ relative to the energy at 180°. After an unconstrained monomer optimization was performed using PBE0+D3, the angle defined by midpoints of the S-S and two C-C bonds in the six-member ring was found to be equal to $\gamma_0 = 140.3°$. Other geometries were obtained from the reference geometry by rotation of all atoms about the S-S axis by the angle $\pm(\gamma - \gamma_0)/2$. Note that since the fragments of the molecule in each side of the S-S bond are not ideally flat, the angle γ is not exactly the same angle as the one given by the definition of γ_0 and the 180° monomer is not ideally flat.

and noniterative triple excitations, CCSD(T), gave the minimum about 1 kcal/mol deeper than the PBE0+D3 one and located at about 135°. The use of the D3 dispersion-energy correction resulted in only a minor improvement as the PBE0 method predicted essentially the same angle and the energy about 0.3 kcal/mol higher than PBE0+D3. In all calculations of interaction energies, the monomers were held rigid at the PBE0+D3-optimized geometry. In some SAPT(DFT) calculations, we used the mirror reflections of such monomer. We will use symbols L and R for the two enantiomers. In crystal structure predictions, the mirror symmetry was applied to monomers, so each monomer could become an L or R enantiomer.

The intermolecular potentials had the same parameters for the LL, RR, and LR dimers.

Four potentials were constructed, each using the functional form

$$V = \sum_{a \in A} \sum_{b \in B} u_{ab}(r_{ab}) \tag{1}$$

where a and b are atoms in monomers A and B, respectively, and r_{ab} is the distance between them. The form of the isotropic atom-atom function u_{ab} varies between the different potentials.

The first and second potentials, denoted `LL+LR_exp-6_com0` and `LL+LR+crys_exp-6_com0`, were constructed for use with the PMIN lattice energy minimization program[11] and therefore had to have the following simple form

$$u^1_{ab}(r_{ab}) = e^{\alpha_{ab} - \beta_{ab} r_{ab}} + \frac{q_a q_b}{r_{ab}} - \frac{\sqrt{C_a C_b}}{(r_{ab})^6} \tag{2}$$

where α_{ab}, β_{ab}, q_i, and C_i are optimized free parameters. The third and fourth potentials, denoted `LL+LR_poly1_com1` and `LL+LR+crys_poly1_com1`, were constructed for use with a molecular dynamics (MD) program and therefore could be of a more complex form

$$u^2_{ab}(r_{ab}) = [1 + a_{ab} r_{ab}]\, e^{\frac{1}{2}(\alpha_a + \alpha_b) - \frac{1}{2}(\beta_a + \beta_b) r_{ab}} + \frac{q_a q_b}{r_{ab}} - f_6(\sqrt{\delta_a \delta_b}, r_{ab}) \frac{\sqrt{C_a C_b}}{(r_{ab})^6} \tag{3}$$

where α_i, β_i, q_i, δ_i, C_i, and a_{ab} are free parameters and f_6 denotes the Tang-Toennies type damping function[12]. The DL_POLY MD program[13,14] that we used does not require combination rules, the reasons for using them will be explained later.

A set of 1381 dimer configurations was used as the primary training data. Of these, 981 are configurations where both monomers are of the same chirality, and the remaining 400 contain monomers of opposite chirality. These configurations were generated using a Monte Carlo type algorithm with importance sampling based on the OPLS-AA[15] guiding potential. A large grid of dimer configurations was first randomly generated, and then each configuration was accepted or rejected with probability proportional to $\exp(-\frac{E_{\mathrm{guide}}}{12.0\,\mathrm{kcal/mol}})$, where E_{guide} is the interaction energy given by the guiding potential. This resulted in 871 configurations. These points were then fit with an initial potential, which was iteratively augmented with additional points until the total set of 1381 grid points was reached. The details of this iterative procedure will be described in Ref. 16.

The potentials `LL+LR+crys_exp-6_com0` and `LL+LR+crys_poly1_com1` were fit to a data set containing, in addition to the 1381 configurations described above, 42 dimers taken from the 6 top ranked crystals obtained in an early phase of this project utilizing only molecular packing and lattice energy minimizations with preliminary versions of our potentials. From each crystal, we selected about 7 dimers based on the criterion of the smallness of closest-contact atoms distances (the two atoms from different monomers closest to each other). In this way we have selected all dimers in the 6 crystals whose closest-contact atoms are within 8 Å. This resulted in a total a total of 1423 data points. SAPT(DFT) interaction energies with the δ_{HF} correction[17] were computed at each of these dimer configurations using the aug-cc-pVDZ basis[10] and the PBE functional[7] for DFT calculations.

Parameterization was performed using a procedure similar to that used in an earlier work on the cyclotrimethylene trinitramine (RDX) dimer, described in Ref. 18. This parameterization is divided broadly into asymptotic and short-range stages. In the asymptotic fitting

stage, the multipole moments and static and dynamic polarizabilities of the monomer are used to generate multipole expansions about the monomer's center of mass. These expansions are then used to compute electrostatic, induction, and dispersion interaction energies on a grid of 10,000 dimer configurations, with monomer's closest-contact atoms separated by at least 15 Å. The C_i and q_i parameters are then optimized using this grid of asymptotic interaction energies. This stage is described in detail in Ref. 18. With such large number of grid points in the asymptotic region, we could have used the C_6^{ab} coefficients instead of those obtained from the combination rules, however, this change was resulting in only small improvements and the applications of the combination rules allowed us to use the same asymptotic expansion as in the `LL+LR_exp-6_com0` and `LL+LR+crys_exp-6_com0` potentials.

In the short-range fitting stage, all remaining free parameters are optimized using the set of SAPT(DFT) interaction energies. The δ_i parameters are first optimized to reproduce the sum of induction and dispersion SAPT components, and then finally the α_i, β_i, and a_{ab} parameters are optimized to reproduce the total SAPT interaction energy while keeping all other parameters fixed. This final optimization is performed in multiple stages, and under a set of constraints designed to produce physically meaningful parameter values. This procedure will be described in detail in Ref. 16. On the 881 training dimer configurations with attractive interactions, the root-mean-square errors (RMSEs) of the potentials `LL+LR_exp-6_com0`, `LL+LR+crys_exp-6_com0`, `LL+LR_poly1_com1`, and `LL+LR+crys_poly1_com1` are 0.65, 0.59, 0.41, and 0.42 kcal/mol, respectively.

The reasons we used combination rules for the parameters α, β, and δ are as follows. For the damping parameters we did not have to use combination rules, but damping is not very sensitive to precise values of the parameters, so the use of δ_{ab} would not make any difference. For the exponential parameters, the reason was that if we had used parameters α_{ab}, β_{ab}, and a_{ab}, this would have resulted in a total of 360 free parameters, a number large compared to the total number of grid points (871) in the first iteration of the potential development. We have later optimized a potential without use of any combination rules fitted to all 1423 data points, and it did reduce the RMSE given above by about 10 percent. However, this potential had too many holes (areas of unphysically low energies at very small intermonomer separations) and since there not enough time to eliminate this behaviour by computing additional interaction energies in these areas, we have not used this potential.

The crystal predictions follow the procedure of Ref. 19. The first step consists of molecular packing by MOLPAK (molecular packing) software suite[20]. Crystals of different symmetries are created using only the monomer's structure on input. MOLPAK produces crystals corresponding to molecular coordination geometries observed in the most common space groups of molecular crystals; descriptions of coordination geometries are given in Refs. 11 and 20. The version of MOLPAK used in this study[11] samples 51 coordination geometries corresponding to the triclinic ($P1$, $P\bar{1}$), monoclinic ($P2_1$, $P2_1/c$, Cc, $C2$, $C2/c$, Pc, $P2/c$, $P2_1/m$, $P2/m$, $P2$, Pm, $P2/m$), and orthorhombic ($Z = 4$, $P2_12_12$, $P2_12_12_1$, $Pca21$, $Pna21$, $Pnn2$, $Pba2$, $Pnc2$, $P222_1$, $Pmn21$, $Pma2$, $Z = 8$, $Pbcn$, $Pbca$) space groups. For each hypothetical crystal generated, an initial arrangement of the atoms within the unit cell is obtained by first orienting the monomer in a Cartesian coordinate system whose origin is located at the centroid of the monomer. The remaining symmetry-equivalent entities are then generated using the appropriate space group symmetry operations and coordinate geometry definition as described in Ref. 20. Sampling of possible orientations of the molecules within each coordination geometry is performed by a systematic angular sweep of the range ±90° in 10° steps. This sampling results in generation of 6,859 (19^3) hypothetical crystal structures per

coordination geometry. The intermonomer separations were then varied for each hypothetical structure until a minimum-volume packing based on a repulsion criterion is reached as described in Ref. 20 and Appendix 1 of Ref. 11. In this way, the MOLPAK step generated 6,859×51 hypothetical crystals which could be rank-ordered by density. Rigid-molecule lattice energy minimizations were next performed on the 500 highest density candidates per coordination geometry using the PMIN lattice energy optimization codes[11]. In this procedure, the cell parameters, location, and orientations of the molecules are optimized within space group symmetry constraints using the potential function described hereafter. The starting values of these parameters were those generated by the MOLPAK step.

The PMIN program[11] can accommodate only the very simple form of the potential given by Eq. (2). Of the two potentials of this form, only the `LL+LR_exp-6_com0` potential was used in PMIN. When its improved version, `LL+LR+crys_exp-6_com0`, was developed, the minimizations were not repeated since we decided that the two potentials are not sufficiently different to make a difference at this stage. The resulting global minimum of symmetry Pna21 had lattice energy of -32.98 kcal/mol per molecule.

The molecular dynamics simulations consisted of five consecutive simulations with the number of crystal structures in each stage generally decreasing. In the first stage, the set of 924 crystal structures with energies below -28 kcal/mol produced by PMIN was minimized with the potential `LL+LR_poly1_com1` which has the more elaborate functional form of Eq. (3). The minimization was achieved by performing a short NsT rigid-monomer molecular dynamics (MD) simulation with the DL_POLY Version 2 (DL_POLY Classic) suite of molecular dynamics simulation software[13,14] at 0.1 K for each of the 924 structures. No symmetry constraints were imposed during the simulation. Such low-temperature MD is practically equivalent to a search for the minimum on the potential energy surface. The supercells used in the simulations were composed of blocks of unit cells, with the contents initially arranged in the configuration corresponding to that of the structure produced by the PMIN/`LL+LR_exp-6_com0` optimization. The sizes of supercells were selected to ensure that the perpendicular widths between opposing faces of the simulation cell were at least 28 Å, more than twice the potential cutoff distance (10.0 Å). In a few cases, the link-cell algorithm in DL_POLY failed and then the size of the simulation cell was increased to 40 Å. Coulombic interactions beyond the cutoff were handled using Ewald summations. Long-range beyond-the-cutoff dispersion/induction interactions were added with an isotropic model of Ref. 21.

In stage 2, the 250 structures of the lowest energies (ranging from -28.2 to -31.6 kcal/mol) from 0.1 K MD simulations were selected for NsT-MD simulations at 298 K using the same potential and the same type of MD as in 0.1 K simulations. Again, the symmetry constraints were not imposed. The starting structure for each simulation was the final structure from one of the MD minimizations of stage one. Velocities corresponding to T = 298 K were assigned to atoms and a trajectory of 30,000 steps (1 time step = 1 fs) was followed to equilibrate the system. The atomic velocities were scaled at every 5th step during this equilibration trajectory to more rapidly drive the system to the desired temperature. After the equilibration, each simulation was continued for another 100,000 steps without velocity scaling. During this stage, atomic configurations were recorded at every 500th step. For 155 simulations which ended up with the lowest energies, the recorded configurations were used to generate time-averaged information about the locations and orientations of the molecules within the unit cells. These coordinates were transformed into fractional coordinates that together with symmetry operators and cell vectors provide a complete information about crystal structure. Cell vectors within 0.1° of ideal 90° angles for the crystallographic system

were rounded to 90°.

In three cases, the deviation from 90° was was larger than 0.1°. For these structures, the angles were manually set to 90° and the MD simulation was performed one more time, constituting stage 3 of the simulations. After this stage, the angles deviated less than 0.1°.

The procedure described above failed for 18 structures as the MD simulation deformed the unit cell and crystal configuration so dramatically that the time-averaged structure did not preserve the translation symmetry and/or the initial symmetry operators. Visual inspection for the lowest-energy such a structure showed that this structure is periodic but the averaging software is not capable of searching of unknown symmetries and or structures with $Z' > 1$. Ten of these structures with the lowest energies are included at the end of the list of predicted crystals with a label "`geometries_not_relaxed`". Their energies corresponds to the 298 K MD relaxed energies, but the configurations are equivalent to `LL+LR_exp-6_com0` 0 K MOLPAK/PMIN structures.

An inspection of the structures suggested that some of them were not fully converged. Therefore, we repeated stage 2, including equilibration, for the crystal structures still considered. This stage 4 used the 137 (= 155 - 18) time-averaged crystal structures from stage 2 as as starting geometries. The use of this approach, rather than continuations of stage 2 simulations from the final configurations, was important for ensuring that the averaged geometries, in particular the shapes of the cells, are fully relaxed and also showed that that the time-averaging was done correctly. The 137 time-averaged crystals from stage 4 were inspected for duplicates and 21 duplicates were removed. This stage determined the submitted crystal geometries for 90 of our crystals. However, the lattice energies and the ranking of these crystals was taken from the next stage.

In stage 5, the 116 structures remaining after stage 4 were used as starting points in one more MD 298 K simulations using the `LL+LR+crys_poly1_com1` potential. As stated before, the training set of this potential included 42 dimers from the crystals from preliminary rankings and therefore this potential is different from the `LL+LR_poly1_com1` potential used in the previous stages. The geometries were changed insignificantly in this stage, so we did not average them again. However, the lattice energies from stage 5 provided the final ranking of the predicted structures. The 90 lowest-energies structures are included in the final ranking. The global minimum of *Pbca* space group has the lattice energy of −30.12 kcal/mol. There are two close-lying $P2_1/c$ structures with the lattice energies of −30.01 and −29.72 kcal/mol, respectively. An alternative submitted ranking shows energies of the `LL+LR_poly1_com1` 298 K simulations, i.e., from stage 4. These results were obtained with a less accurate potential, but the energies are fully consistent with the geometries.

II. COMPUTER RESOURCES

A SAPT(DFT) calculation including the δ_{HF} correction took about 23 wall time hours per grid point on two 2.5 GHz Intel Ivy Bridge cores. The entire set of 1423 dimer interaction energies required about 66000 CPU hours. Other steps of potential generation were negligible in comparison.

The MOLPAK/PMIN step was performed on an Opteron 2.6 GHz and took 0.25–0.75 hours per coordination geometry, i.e., about 20 hours total. The MD steps were performed on a 2.6 and 2.2 GHz Opteron on 1 or 4 cores. The timings of the parallel jobs are given as a multiple of wall time and number of cores used. The 0.1 K MD calculations for 924 structures took about 700 hours and the 298 K MD step for the total set of about 550

crystals was 5500 hours.

III. POST-SUBMISSION ANALYSIS

The experimental crystal, the `ai_71` structure in our notation, turned out to be number 3 on our alternate list, but became number 8 on our main list. We found that the reason for this drop in rankings was an error in SAPT(DFT) calculations at the 42 additional grid points which were used to fit the `LL+LR+crys_poly1_com1` potential. This potential was used only in stage 5 to rerank the structures from the alternate list (this stage produced the main list). These 42 configurations mistakenly did not include the exchange-dispersion contribution $E^{(2)}_{\text{exch-disp}}$. We corrected this mistake and RMSE of the refitted the potential on all points with negative interaction energies was reduced from 0.42 to 0.39 kcal/mol. This reduction is not large, but since the 42 points are in important regions, the erroneous deformation of the potential in these regions was apparently important. The size of the error is well seen from the fact that RMSE of the correct interaction energies at these 42 grid points is 0.69 kcal/mol with `LL+LR+crys_poly1_com1` and only 0.26 kcal/mol with its corrected version.

We recalculated the ranking from stage 5 with the corrected potential, performing again 298 K MD simulations, and the experimental crystal became the structure of rank 4 with lattice energy of -28.36 kcal/mol, only 0.16 kcal/mol above the rank 1 structure, whereas this separation was 0.88 kcal/mol on our alternate list. The separation from the rank 3 structure is only 0.01 kcal/mol. Thus, despite the slight worsening of the rank, the experimental crystal is now very close in energy to all higher-ranked crystals. One possible reason why the ranking has not improved is that the set of 6 preliminary crystals does not contain the experimental crystal.

We have computed before the submission date a set of energies for additional 104 dimers extracted from the 25 lowest-energy structures originating from the same early phase of this project as the 42 dimers discussed above, but ranked after performing the 0.1 K MD simulations. These 25 crystals do not include the experimental structure. Due to time restrictions, we were not able to use these points prior to the submission date, but we used them now to fit a new potential to the total of 1527 grid points. In all other respects, the new potential, denoted as `LL+LR+crys'_poly1_com1`, is identical to `LL+LR+crys_poly1_com1`. The new potential was used in step 5, i.e., we performed MD simulations at 298 K, resulting in the experimental crystal ranked as number 2. It is 0.15 kcal/mol above the rank 1 crystal (denoted as `ai_151` in our set of structures).

The improvement obtained with the `LL+LR+crys'_poly1_com1` potential suggests that the additions of dimers from crystal structures results in a convergence to the correct result. We should have, however, utilized crystal structures from later stages of our process since these would have included the experimental crystal. Clearly the next step that can be performed without using the knowledge of the experimental structure (i.e., retaining the blind test conditions) is to extract dimers from the 25 lowest energy crystals of the last step. With the increased number of grid points available now, we can also stop using the combination rules which will increase the quality of the fit.

ACKNOWLEDGMENTS

The work at the University of Delaware was supported by the Army Research Office under Grant W911NF-13-1-0387 and by the National Science Foundation Grant CHE-1152899. The work at the University of Silesia was supported by the Polish National Science Centre Grant No. DEC-2012/05/B/ST4/00086.

---

[1] B. Jeziorski, R. Moszyński, and K. Szalewicz, Chem. Rev. **94**, 1887 (1994).

[2] K. Szalewicz, Wiley Interdisc. Rev.–Comp. Mol. Sci. **2**, 254 (2012).

[3] A. J. Misquitta, B. Jeziorski, and K. Szalewicz, Phys. Rev. Lett. **91**, 033201 (2003).

[4] A. J. Misquitta, R. Podeszwa, B. Jeziorski, and K. Szalewicz, J. Chem. Phys. **123**, 214103 (2005).

[5] R. Bukowski, W. Cencek, P. Jankowski, M. Jeziorska, B. Jeziorski, S. A. Kucharski, V. F. Lotrich, A. J. Misquitta, R. Moszyński, K. Patkowski, R. Podeszwa, F. Rob, S. Rybak, K. Szalewicz, H. L. Williams, R. J. Wheatley, P. E. S. Wormer, and P. S. Żuchowski, "SAPT2012: An *ab initio* program for many-body symmetry-adapted perturbation theory calculations of intermolecular interaction energies," University of Delaware and University of Warsaw (2012).

[6] F. Neese, "*ORCA: An Ab Initio, DFT, and Semiempirical electronic structure package*," with contributions from U. Becker, D. Ganyushin, A. Hansen, D. Liakos, C. Kollmar, S. Kossmann, T. Petrenko, C. Reimann, C. Riplinger, K. Sivalingam, B. Wezisla, and F. Wennmohs.

[7] J. P. Perdew, K. Burke, and M. Ernzerhof, Phys. Rev. Lett. **77**, 3865 (1996).

[8] C. Adamo and V. Barone, J. Chem. Phys. **110**, 6158 (1999).

[9] S. Grimme, J. Antony, S. Ehrlich, and S. Krieg, J. Chem. Phys. **132**, 154104 (2010).

[10] R. A. Kendall, T. H. Dunning, Jr., and R. J. Harrison, J. Chem. Phys. **96**, 6796 (1992).

[11] J. Holden, H. Ammon, Z. Du, S. Prasad, E. Wells, and N. Albu, "Structure predictions with MOLPAK and PMIN or DMACRYS," (March 2014), University of Maryland.

[12] K. T. Tang and J. P. Toennies, J. Chem. Phys. **80**, 3726 (1984).

[13] I. Todorov, W. Smith, K. Trachenko, and M. Dove, J. Mater. Chem. **16**, 1911 (2006).

[14] DL_POLY Classic: `http://www.ccp5.ac.uk/DL_POLY_CLASSIC/`.

[15] W. L. Jorgensen, D. S. Maxwell, and J. Tirado-Rives, J. Am. Chem. Soc. **118**, 11225 (1996).

[16] M. P. Metz, K. Piszczatowski, and K. Szalewicz, (2015), manuscript in preparation.

[17] K. Szalewicz, K. Patkowski, and B. Jeziorski, *Intermolecular Forces and Clusters*, Structure and Bonding **116**, 43 (2005).

[18] R. Podeszwa, R. Bukowski, B. M. Rice, and K. Szalewicz, Phys. Chem. Chem. Phys. **9**, 5561 (2007).

[19] R. Podeszwa, B. M. Rice, and K. Szalewicz, Phys. Chem. Chem. Phys. **11**, 5512 (2009).

[20] J. R. Holden, Z. Du, and H. L. Ammon, J. Comput. Chem. **14**, 422 (1993).

[21] M. P. Allen and D. J. Tidesley, *Computer Simulation of Liquids* (Oxford, 1987).
