# Supplementary material for: Report on the sixth blind test of organic crystal structure prediction methods
Source: Acta Crystallogr B Struct Sci Cryst Eng Mater. 2016 Aug 1;72(Pt 4):439–59. doi: 10.1107/S2052520616007447 (PMC4971545; doi:10.1107/S2052520616007447)
Supplement: Supplementary file 3 [file b-72-00439-sup3.zip › Group_18_Price_Post_Analysis.pdf]

Price group 18 Supporting Information for 6th Blind Test submission

Sarah L. Price, Rebecca K. Hylton (XXII), Louise S. Price(XXIII), Rui Guo (XXIV), Rona E. Watson (XXV) and Luca Iuzzolino (XXVI)

Department of Chemistry, University College London, 20 Gordon St., London WC1H 0AJ, U.K.

General approach and methodology

Our strategy was built upon the knowledge that we are not yet able to evaluate the lattice, let alone the free, energies, of the organic crystal structures that we will generate to sufficient accuracy. The degree of cancellation of errors in the relative energies and rankings is very dependent on not only the functional groups and conformational flexibility of the molecule (which is assessed prior to generating the structures) but also the range of energies, densities and variety of conformations, hydrogen bonding and stacking motifs etc. in the low energy structures. This is not apparent until after the structures have been generated. Many-body dispersion is expected to be important in the relative lattice energies of all of the target systems (with the possible exception of XXIV), and progress in evaluating this for benzene has only recently (Yang *et al.*, 2014) reached an absolute accuracy of 1 kJ mol^{-1}. Hence we do not have the resources available to use the best available energy evaluation methods in our submission, let alone confidence that thermodynamic stability is a reliable criterion for predicting the crystal structures of all molecules (Hylton *et al.*, 2015). We have collaborated with groups who are developing more accurate energy evaluation methods, by providing them with 1000 putative structures for each system. We have also varied the approach used for each molecule to be the best we could afford, depending on the system, and tested the sensitivity of our ranking to the method used. The details of the CSP study for each target are summarized and compared in Table 1.

The overall approach is based on the lattice energies of infinite perfect crystals, divided into the intermolecular contribution, U_{inter}, and the energy penalty paid for changing the conformation from the most stable conformation for the isolated molecule, ΔE_{intra}, using $E_{latt} = U_{inter} + \Delta E_{intra}$. In all cases a preliminary assessment of the flexibility of the molecule was carried out, using ab initio conformational energy scans on the isolated molecule, and checked against analysis of the CSD for conformer distributions. The degrees of freedom were classified into those that would not change significantly under the influence of crystal packing forces (all bondlengths and most bond angles), those that would only vary slightly or where the variation would not change the overall shape of the molecule (such as torsions involving hydrogen atoms), and those which had such a major effect on the shape of the molecule that they would have to be included as a search variable in the structure generation stage. The sensitivity of the most widely-varying, lowest-barrier torsion angles to ab initio method was used, along with affordability considerations, to determine the type of electronic structure method used to evaluate ΔE_{intra} and the molecular structure in Gaussian calculations. The corresponding charge density was analyzed to provide the potential derived (CHELPG) atomic charges used in the search and subjected to a Distributed Multipole Analysis (Stone, 2005) using GDMA2.1 (Stone, 2010) to provide atomic multipoles up to hexadecapole (DMA) to model the electrostatic contribution to U_{inter} in the refinement stages. The other contributions to U_{inter} were estimated by an isotropic atom-atom *exp-6* potential using the empirically fitted FIT potential parameters (Coombes *et al.*, 1996, Price *et al.*, 2010) unless otherwise stated.

The crystal structures were generated using CrystalPredictor (Kazantsev, Karamertzanis, Adjiman, Pantelides*, et al.*, 2011, Karamertzanis & Pantelides, 2007, 2005, Pantelides *et al.*, 2014), using the version most suited to the number and interdependence of the search conformational degrees of freedom (Table 1). Thus, this varied from XXII and XXV using the ab initio optimized molecules as rigid molecules in the search, to setting up grids of ab initio estimated energies, and doing multiple searches for distinct regions.

The searches were all conducted for Z'=1 and covered 59 space groups, once it had been established that none of the target molecules could be assumed to contain only a single enantiomer. The space groups covered were P1, P-1, P21, P21/c, P21212, P212121, Pna21, Pca21, Pbca, Pbcn, C2/c, Cc, C2, Pc, Cm, P21/m, C2/m, P2/c, C2221, Pmn21, Cmc21, Aba2, Fdd2, Iba2, Pnna, Pccn, Pbcm, Pnnm, Pmmn, Pnma, Fddd, Ibam, P41, P43, I-4, P4/n, P42/n, I4/m, I41/a, P41212, P43212, P-421c, I-42d, P31, P32, R3, P-3, R-3, P3121, P3221, R3c, R-3c, P61, P63, P63/m, P213, Pa-3, Cmcm and Cmca.

CrystalPredictor uses Sobol sequences in the search variables and optimizes the lattice energies of the structures using the FIT *exp-6* atom-atom potential (Price *et al.*, 2010) and the CHELPG atomic charges. Typically, 1,000,000 structures would be generated in each search, and the completeness evaluated by the extent to which the low energy structures were found multiple times. The structures were then clustered to remove duplicates using CrystalPredictor's internal method, based on closest intermolecular atom-atom distances. The structure labels used are the ranking of the structures at this stage, preceded by the region if multiple searches were run.

The next stage was an improvement in the energy rankings by replacing the atomic charges with the distributed multipoles of the corresponding conformation, and lattice energy minimizing with DMACRYS (Price *et al.*, 2010). If the molecule was treated as flexible in the search, then the conformations in each structure are different, and need their own ab initio calculation for ΔE_{intra} and the DMA. For XXVI, one iteration of CrystalOptimizer was used to both apply the multipoles, give a correct rather than estimated value of ΔE_{intra}, and ensure that all the degrees of freedom that were not being explicitly varied in the search correspond to the constrained optimized conformation for the torsion angles determined in the search.

Full CrystalOptimizer (Kazantsev *et al.*, 2010, Kazantsev, Karamertzanis, Adjiman & Pantelides, 2011) runs were performed on the most stable crystal structures following this intermediate stage, varying all the angles that were expected to be affected by the packing forces. This was usually confirmed by running a few dozen structures with the maximum possible degrees of freedom to see which varied the most. CrystalOptimizer produces a database of the energies, energy gradients, and multipoles obtained from the isolated molecule ab initio calculations, and uses local approximate models (LAMs) to interpolate between points, and so the optimization of structures with a similar conformation to ones that have already been processed is considerably quicker.

The clustering to remove duplicates after DMACRYS and CrystalOptimizer was done on the basis of energy and density differences, and within this window by comparing the powder pattern similarity, and then finally by considering the overlay of the coordination shell within the crystal structure, using the CCDC method of determining the best overlay of a coordination shell of n molecules, $rmsd_n$ (Chisholm & Motherwell, 2005). The variations in the size of the molecules and number of molecules within the formula unit meant that the parameters for clustering had to be varied with system.

The converged CrystalOptimizer energies correspond to lattice energy minima which have been checked to be true minima within the space group constraints by consideration of the Hessian matrix (this generated some Z'=2 structures). The 100 lowest energy structures are used for the first list.

The second list aimed to be the 100 most likely structures. The structures included differ significantly from those in the first list when the likely energy range of polymorphism, allowing for the likely error in our estimate of the relative thermodynamic stability, included more than 100 structures. This energy cutoff for defining the crystal energy landscape can only be estimated, since it is the energy barrier to transformation to more stable forms, rather than the energy difference, that dictates whether a structure is a long-lived metastable polymorph, and it is difficult to define a suitable measure of the uncertainty in relative energy rankings. Work on larger flexible molecules suggests that polymorphic energy differences can be larger than those for smaller rigid molecules when there is a substantial conformational difference (Goldstein *et al.*, 2015), with polymorphs produced by solid state routes, such as desolvating solvates, also potentially being highly metastable (Braun *et al.*, 2014).

The uncertainty in the relative energy estimates used in List 1 was crudely estimated by a sensitivity analysis, looking at whether a sample of structures with very different packing motifs changed ranking when reasonable, affordable changes were made to the energy model. In most cases, this included substituting the FIT *exp-6* parameters by the W99 parameters (Williams, 2001), and re-evaluating ΔE_{intra} and the multipoles in a polarizable continuum model (PCM) (Cossi *et al.*, 2002) with a dielectric constant ε of 3 (ε = 11 was used for the salt XXIV). An estimate was made of the Helmholtz free energy at 298 K from the rigid-body k=0 phonon modes and elastic constants for the unit cell (Anghel *et al.*, 2002). This is a crude approximation to the free energy (c.f. the model required for aspirin polymorphs(Reilly & Tkatchenko, 2014) without the rigid body assumption, and should be converged for unit cell size (Nyman & Day, 2015), but such calculations show that this term reranks only 10% of known polymorph pairs (Nyman

& Day, 2015). The submitted second list was ranked using an alternative energy evaluation from List 1, but this was always one produced using DMACRYS. The reasoning behind the estimates of computational error and energy range of plausible polymorphism are given for each individual target in the discussion of the prior to submission confidence section.

The final stage in trying to determine the most likely polymorphs is based on comparing the unique structures within the energy range of possible polymorphism (including computational uncertainty), looking for distinct packing arrangements in terms of hydrogen bonds, common chains and layers. Structures that appeared to be sufficiently similar to lower energy structures that they were unlikely to be distinct free energy minima or nucleate and grow separately (Price, 2013) were eliminated from the higher energy regions. Since the blind test targets are not disordered, we are not interested in structures that are so similar that they might be observed as disorder components. However, we note that we would consider our second list successful if it contained a structure that was closely related to the experimental structure, such as an alternative stacking of the same layers, a Z'=1 approximation to a Z'=2 structure, or other cases where there was an overlay with 5 or more molecules in the coordination unit, but not the same crystallographic unit cell and space group. However, the experience of examining the blind test crystal energy landscapes continued to show the challenges in defining criteria for "effectively the same structure" that are not a matter for human judgement based on experience in comparing the crystal energy landscapes for similar molecules that have undergone polymorph screening.

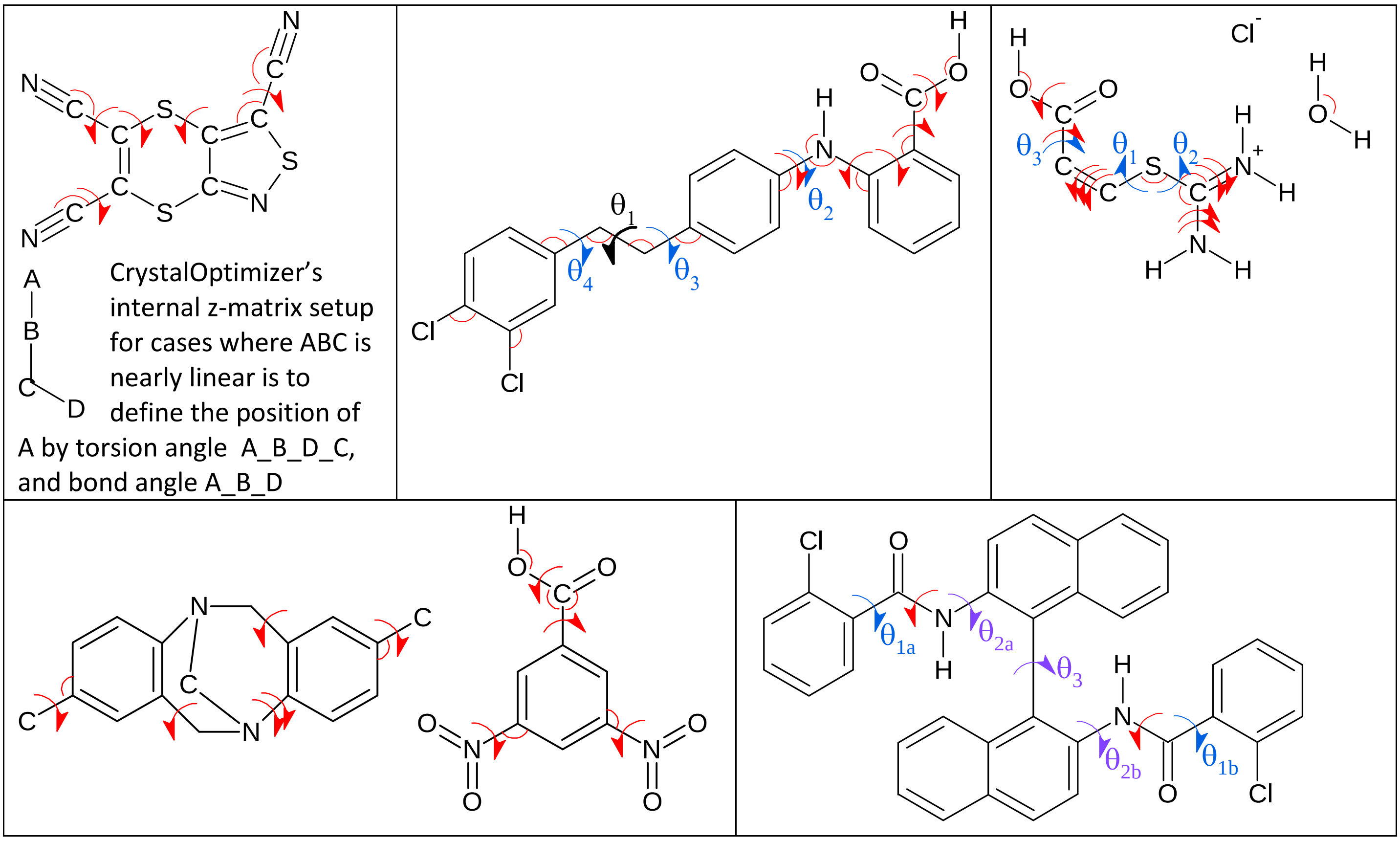

Figure 1. Flexible degrees of freedom considered at different stages. Purple and blue arrows denote degrees of freedom considered flexible during the search, and all arrows denote degrees of freedom refined in the CrystalOptimizer step.

Table 1. Specific details of methods used for each molecule.

	XXII Rebecca Hylton	XXIII Louise Price	XXIV Rui Guo	XXV Rona Watson	XXVI Luca Iuzzolino
CrystalPredictor search type * and no. of structures generated and minimized	Rigid v1.6<br>1,000,000 minimizations<br>(-S- parameters for S)	Flexible v2.1<br>3 regions with fixed θ_1 and θ_2 = 0-360°, θ_3 = 0-180°, θ_4 = 0-360°<br>Region A: θ_1 = 179.7°<br>Region B: θ_1 = -66.146°<br>Region C: θ_1 = 64.578°<br>Total 1,869,794 minimizations (534,976 A, 734,738 B, 600,000 C)	Flexible v1.8<br>3 regions<br>Region A: θ_1 = 140-220°; θ_2 = 10-170°, θ_3 = 180°.<br>Region B: θ_1 = 40-120°; θ_2 = 10-170°, θ_3 = 180°.<br>Region C: θ_1 = 40-120°; θ_2 = 10-170°, θ_3 = 0°.<br>Total 1,750,000 minimizations (1,000,000 in A, 500,000 in B, 250,000 in C)	Rigid v1.8<br>2,000,000 minimizations	Flexible v1.6<br>Grids using model molecules<br>Grid a=Grid b: θ_1 = 20-340°<br>Grid c: θ_3=-130 to -70°, θ_{2a} & θ_{2b} = 100-320°<br>1,000,000 minimizations
Molecular Ψ	PBE0/6-31G(d,p)	PBE0/6-31G(d,p)	PBE0/6-31G(d,p)	PBE0/6-31+G(d)	PBE0/6-31G(d,p)
Clustering criteria	CrystalPredictor default	CrystalPredictor default	CrystalPredictor default + CrystOpt clustering with<br>ΔEnergy < 0.1 kJ mol^{-1}<br>PXRD Simi (5-40 2θ) > 0.97<br>$RMSD_{50}$ (5% distance / 5° angle tolerance) < 0.05 Å.	CrystalPredictor default	CrystalPredictor default
Number of unique structures after CrystalPredictor, and energy range	10000 structures within about 29 kJ mol^{-1}	28,068 Region A, 14,200 Region B, 1,705 Region C within 30 kJ mol^{-1}<br>43,973 taken on	Region A: 5,609 within 27.7 kJ mol^{-1} Region B: 9,524 within 31.5 kJ mol^{-1} Region C: 1,319 within 20.6 kJ mol^{-1} of min in that region	~80,000 structures within ~20 kJ mol^{-1}<br>40,000 structures taken on	~95,000 structures within 60 kJ mol^{-1}<br>9,400 structures within 40 kJ mol^{-1} taken on
Next step	Single step DMACRYS + FIT potential, with PBE0/6-31G(d,p) multipoles	Single step DMACRYS + FIT potential, with PBE0/6-31G(d,p) multipoles calculated for each structure	None	Single step DMACRYS + FIT potential, with PBE0/6-31+G(d) multipoles.	Single iteration of Crystal Optimizer. PBE0 6-31G(d,p) +FIT for each structure.
Clustering criteria	ΔEnergy < 0.5 kJ mol^{-1}<br>ΔDensity < 0.01 g cm^{-3}<br>PXRD Sim. (5-40 2θ) > 0.97<br>$RMSD_{20}$ (20% distance /	ΔEnergy < 0.3 kJ mol^{-1}<br>ΔDensity < 0.01 g cm^{-3}<br>PXRD Sim. (5-40 2θ) > 0.97<br>$RMSD_{20}$ (20% distance / 20° angle tolerance) < 0.25 Å.		ΔEnergy < 0.1 kJ mol^{-1}<br>ΔDensity < 0.05 g cm^{-3}<br>PXRD Sim. (5-40 2θ) > 0.97<br>$RMSD_{30}$ (20% distance / 20° angle tolerance) < 0.1 Å.	ΔEnergy < 2.5 kJ mol^{-1}<br>ΔDensity < 0.05 g cm^{-3}<br>PXRD Sim. (5-40 2θ) > 0.97<br>$RMSD_{20}$ (20% distance / 20° angle tolerance) < 0.65 Å.

	20° angle tolerance) < 0.25 Å.				
Number of unique structures and energy range	8306 within 45 kJ mol^{-1} 1000 within 20 kJ mol^{-1} taken on	15,638 within 30 kJ mol^{-1} 2,494 within 20 kJ mol^{-1} taken on	Taken forward: 2000 in Region A (range of 22 kJ mol^{-1}) 1000 in Region B (19 kJ mol^{-1}) 1319 in Region C (21 kJ mol^{-1})	5584 within 40 kJ mol^{-1} 234 within 20 kJ mol^{-1} 26 within 10 kJ mol^{-1}	1322 within 30 kJ mol^{-1} taken on
CrystalOptimizer refinement; see Fig for independently refined angles	5 torsion & 4 bond angles PBE0/6-31G(d,p) +FIT (-S-)	9 torsion & 13 bond angles. PBE0/6-31G(d,p) + FIT	12 torsion & 5 bond angles PBE0/6-31G(d,p) +FIT (-S-, Cl$^-$)	10 torsion & 7 bond angles. PBE0/6-31+G(d) +FIT	7 torsion angles PBE0/6-31G(d,p) +FIT
Clustering criteria	ΔEnergy < 0.3 kJ mol^{-1} ΔDensity < 0.01 g cm^{-3} PXRD Sim. (5-40 2θ) >0.97 $RMSD_{30}$ (30% distance / 30° angle tolerance)< 0.1Å	ΔEnergy < 2.5 kJ mol^{-1} ΔDensity < 0.01 g cm^{-3} PXRD Sim.(5-40 2θ) > 0.97 $RMSD_{30}$ (20%/ 20°) < 0.35 Å.	ΔEnergy < 0.5 kJ mol^{-1} ΔVolume < 10 Å^3 PXRD Sim.(5-40 2θ) > 0.97 $RMSD_{50}$(20% /20°)< 0.10 Å.	ΔEnergy < 0.1 kJ mol^{-1} ΔDensity < 0.05 g cm^{-3} PXRD Sim. (5-40 2θ) > 0.97 $RMSD_{30}$(20%/20°) < 0.1 Å.	ΔEnergy < 2.85 kJ mol^{-1} ΔDensity < 0.05 g cm^{-3} PXRD Sim. (5-40 2θ) > 0.97 $RMSD_{30}$ (20%/20°) < 0.65 Å.
Number of unique structures and energy range	909 within 29 kJ mol^{-1}	1029 within 30 kJ mol^{-1}	1000 within 31.6 kJ mol^{-1}	3019 within 40 kJ mol^{-1} 149 within 20 kJ mol^{-1} 10 within 10 kJ mol^{-1}	902 within 37 kJ mol^{-1} 97 within 15 kJ mol^{-1} 28 within 10 kJ mol^{-1}
Final energy model for List1	PBE0/6-31G(d,p) Ψ for conformation (9 dof) and DMA and FIT (-S-) pot.	PBE0/6-31G(d,p) Ψ for conformation (22 dof) and DMA and FIT potential	PBE0/6-31G(d,p) Ψ for conformation (17 dof) and DMA and FIT (-S-, Cl$^-$ potential	PBE0/6-31+G(d) Ψ for conformation (17 dof) and DMA and FIT potential	PBE0/6-31G(d,p) Ψ for conformation (7 dof) and multipoles, and FIT pot.
Energy range of List1	7.9 kJ mol^{-1}	9.86 kJ mol^{-1}	17.4 kJ mol^{-1}	18.85 kJ mol^{-1}	16.1 kJ mol^{-1}
Final energy model for ranking List2 unless otherwise noted.	ΔE_{intra} and DMA from ε=3 PCM PBE0/6-31G(d,p) Ψ. U_{inter} from FIT (S=SO2) *exp-6* +DMA. Rigid body free energy.	ΔE_{intra} and DMA from ε=3 PCM PBE0/6-31G(d,p) Ψ. U_{inter} from FIT *exp-6* +DMA. Rigid body free energy.	ΔE_{intra} and DMA from ε=11 PCM PBE0/6-31G(d,p) Ψ. U_{inter} from Williams99 exp-6 (-S-, Cl- potential) +DMA.	ΔE_{intra} and DMA from ε=3 PCM PBE0/6-31G(d) Ψ. U_{inter} from FIT *exp-6* +DMA. Rigid body free energy.	ΔE_{intra} and DMA from ε=3 PCM PBE0/6-31G(d,p) Ψ. U_{inter} from FIT *exp-6* +DMA. Rigid body free energy.
Sensitivity Analysis, as max likely changes in energy for alternatives Energies in kJ mol^{-1}	CrystOpt(-S-) vs CrystOpt(SO2) = 1.5 CrystOpt(SO2) vs PCM(SO2) = 1.1 PCM(SO2) vs PCM + FE (SO2) = 1.3	CrystOpt(PBE) vs CrystOpt(B3LYP) Ψ ~ 0.8 CrystOpt(FIT) vs CrystOpt(Will) *exp-6* ~ 1.6 PCM ~1.4 PCM vs PCM+fe ~ 1	W99 potential+PCM > 20	W99 unsuitable for these hydrogen bonds PCM ~ 1 Free energy ~ 1.5	W99 ~ 6 PCM ~ 14 Free energy ~ 3

Structures for List2 using energy model given above	Lowest energy 100 unique structures after manually removing some higher energy structures that also appeared in List1. Energy range of list 2: 11.156 kJ mol^{-1}	Removed similar structures then by energy ranking. Energy range of list 2: 12.36 kJ mol^{-1} is similar to sum of 10 kJ mol^{-1} for energy range of polymorphism + 2.5 for energy uncertainty	100 low-energy unique structures. The use of the PCM model drastically changes the relative ΔE_{intra} Energy range for list 2: 20.25 kJ mol^{-1}	Lowest 100 unique structures.<br><br>Energy range of list 2 = 18.19 kJ mol^{-1}	Removed similar structures then by energy ranking. Energy range of 22.21 kJ mol^{-1} for List2:
† CPU estimate/ khrs	Conformational Anal. <1<br>Search & single point DMACRYS 2<br>CrystalOptimizer 22.5<br>PCM + free energies 1<br>**Total 26**<br><br>Sensitivity etc. 41	Conformational Anal. 2<br>Conformations for search 12<br>Search 7<br>Single point DMACRYS 24<br>CrystalOptimizer 35<br>PCM + free energies 1<br>**Total 84**<br>Sensitivity analysis 3	Conformational Anal <1<br>Search 12<br>CrystalOptimizer 51<br>PCM <1<br><br>**Total 63**	Method choice and conformational Anal ~ 0.25<br>Search 27<br>Single point DMACRYS 0.5<br>CrystalOptimizer 140<br>PCM + free energies 1<br>**Total ~ 169**	Conformational Anal 11<br>Conformations for search 19<br>Search 20<br>Single iteration of CO 170<br>CrystalOptimizer 105<br>PCM + free energies 1.8<br>**Total 327**<br>Sensitivity analysis 1.7

Green highlighting denotes the set of structures from which the lowest 1000 were sent to Johannes Hoja of Alexander Tkatchenko's group at the Fritz Haber Institute of the Max Planck Society Theory Department Berlin-Dahlem, Germany and Gerit Brandenburg at University of Bonn. In the case of XXVI, the first 1000 structures to complete to this stage were sent, due to time taken to get to this stage, and not wanting to leave the Hoja group with insufficient time to investigate them. For XXIV these structures were generated with the FIT organic Cl potential, and we later redid the search with the Cl^- potential.

* CrystalPredictor versions 2.x and 1.x differ in how the intramolecular energy penalty is estimated within the search.
CrystalPredictor2.1 works using a previously calculated database of conformations. At each conformation GAUSSIAN is used to calculate the constrained optimized molecular conformation, with the predetermined values for the independent degrees of freedom. The conformation and its Hessian are stored in the database, along with the corresponding point charges. During a minimization, the point charges for the LAM point are used without modification, but the molecular conformation and ΔE_{intra} are updated as the conformation changes.

CrystalPredictor1.6 works from a grid of ΔE_{intra} values calculated using GAUSSIAN for each combination of torsion angles with all other conformational parameters fixed at the values for the fully optimized molecule. Point charges are those calculated for the gas phase optimized molecule. Separate parts of the molecule can be treated with separate grids derived from model molecules, which was used only for XXVI, as allowing much more conformational space to be explored.

† The CPU timings are from an old cluster, mainly of processors which are a decade old. We acquired a new cluster just before the deadline, which is significantly faster, by a factor of 2-3.

Discussion on specific features of the methodology used for compiling the two lists for each target.

XXII – Rebecca K. Hylton

The information provided defined XXII as being a rigid molecule, with a subsequent amendment stating that XXII can be considered to be pseudo chiral with two conformations resulting from the potential flexibility of the six membered ring. Conformations could be adopted which position the two nitrile groups and connected ring atoms in the six membered ring above or below the plane of the 5 membered ring. A torsion angle scan was performed around the N-C-S-C dihedral in the ring system which showed that it could change between 143 and 153° with a small energy penalty (<1 kJ mol^{-1}) and indeed the barrier to inversion was about 7 kJ mol^{-1}. A rigid search was performed to generate the structures with CrystalPredictor. Full atomistic CrystalOptimizer calculations were performed on 30 of the generated structures which determined that the 5 dihedrals and 4 angles shown in Figure 1 needed optimizing in all CrystalOptimizer calculations.

There is a large uncertainty in the accuracy of the sulphur potential used for calculations and to a lesser degree the cyanide groups. A search through the CCDC for similar molecules to test the sulphur potential on gave no suitable hits and so it was not possible to test the accuracy of the potential prior to performing the search. Two different sulphur potentials were used for the study: the FIT potential S(-S-) and the S(SO2) thiazides potential. It is expected that the S(-S-) potential will be more accurate due to the similarity of the bonding environment, but keeping the repulsion-dispersion potential isotropic is a major simplification for N≡C and -S- atoms.

The search produced a large density of structures within a small energy and density range. The conformations vary from planar to quite bent. There was no dominant dimer motif and many different layer structures which could stack in a multitude of ways. The molecule is sufficiently small and flexible and the interactions appeared so non-directional that it seems likely to be able to rearrange into the most thermodynamically stable structure, which the free energy estimates suggest would vary with temperature. The energy difference between polymorphs is likely to be small. However, accurately determining the relative energies, or providing a cutoff in degree of similarity between structures to distinguish which could remain distinct during crystallization was not possible. Thus List2 was constructed using a very different energy model (different S potential, PCM and rigid body free energy), and re-optimizing with CrystalOptimizer. In most cases the structures remained virtually identical, but in some cases there was a marked difference. The energy uncertainty between the two models, taken as the average difference the exact and predicted values from a regression analysis, was about 5 kJ mol^{-1}, rather larger than plausible polymorphic energy differences for this molecule. Hence, List2 followed the energy ranking for the second model up to 5 kJ mol^{-1} (18 structures), and then only contained those structures which were not virtually identical to those in List1, in order to represent more of the structural diversity. It seems likely that this molecule could have a high temperature dynamically disordered polymorph.

Post-Analysis for XXIII The experimental structure was within the most stable group of structures, coming 6th in list 1 and 2nd by only 0.5 kJ/mol in list 2. Many other more sophisticated methods were applied to this structure, with comparable success. Our results are consistent with those of Tkatchenko and Day in indicating that free energies are significant in improving ranking this structure as the most thermodynamically stable.

XXIII – Louise S. Price

The search for XXIII was carried out in three conformational Regions, as the conformational analysis of the molecule revealed that there were three distinct angles for the C-C-C-C angle joining the two rings. Following the searches, it was seen that the two folded conformations of Regions B and C were mirror images of one another, but the small differences in constraints used in the generation of LAM points led to some differences in output structures, although the majority of structures were duplicated between the two searches. These folded conformations are significantly higher in conformational energy, and yielded higher energy structures. The intramolecular N-H···O=C hydrogen bond was assumed throughout the search but subsequently a few structures were tested and it was seen that the alternative carboxylic acid orientation was significantly higher in energy, and so disorder in the carboxylic acid dimer is unlikely, due to the hydrogen bonded N-H group.

Due to a problem with the computers, the Region A search ended after 534,976 minimizations had been completed. The programme cannot be restarted after such a termination, so it was decided to see if the search was complete enough before restarting the search from scratch. Normally, we would require that the 20 lowest energy structures are found at least 10 times before we would consider a search complete. At this point, half of the 20 lowest energy structures were found more than 10 times, and the others were all found more than once, so it was decided that the search was adequate. Similarly, the Region B search was stopped after 734,738 minimizations, because 14 of the 20 lowest energy structures were found more than 5 times. As a consequence, the Region C search was limited to 600,000 minimizations, which again was deemed adequate. In summary, the search was not 100% complete, but was felt to be the best balance of computer availability and search coverage.

The search was only run in Z'=1. Some of the structures were observed not to be true minima by DMACRYS, and symmetry reduction was carried out to reduce these saddle point structures to lower energy lower symmetry structures. This is why a handful of low energy structures are Z'=2 with similar conformations of the two molecules.

The majority of structures are in an extended conformation. Three quarters of the structures contain the carboxylic acid dimer that is observed in the smaller fenamates (Uzoh *et al.*, 2012). The low energy structures that do not have this $R_2^2(8)$ motif are stabilized by a greater density, and the most stable of these have $C_1^1(4)$ hydrogen bonded chains. There is a huge diversity in crystal packing, consistent with the observed polymorphism. However, there are some common sheets, with both A70 (the global minimum in List1) and A1361 (the global minimum in List2) containing different sheets that are both seen in various other low energy structures. The variety of symmetry relationships between the sheets could indicate different polymorphs, potential polymorphic domains or merely stacking faults (Price *et al.*, 2014). There are also a variety of stacks of the hydrocarbon moieties that reoccur in many structures, which do not necessarily contain the $R_2^2(8)$ hydrogen bonds. A few pairs of structures had a 15 molecule overlay with each other with only a slight difference in conformation; the higher energy of the pair was eliminated as unlikely to be a distinct polymorph. However, overall the diversity of structures would suggest that this molecule would be polymorphic. Indeed the role of kinetics in the crystallizations, with the possibility of desolvated solvates and problems in nucleating or growing some structures, raises the question as to whether the most thermodynamically stable observed structure would correspond to the global minimum in a perfect CSP study (Hylton *et al.*, 2015).

This is not a perfect CSP study, as we have not been able to evaluate the lattice energy, let alone free energy, to the accuracy required. The energy range of possible polymorphism for this type of molecule is large (~ 10 kJ mol^{-1}) given the possibility of desolvated solvates and difficulty of rearranging to a lower energy structure. The range of densities of the computer generated structures means that the energy is sensitive to the treatment of dispersion, the range of conformations is large with flat potential wells and so the conformational energy penalty is poorly estimated by available methods, etc. Although the two lists have many structures in common, we cannot be confident that all possible structures are within the 1029 structures refined by CrystalOptimizer, let alone the submitted lists.

Post-Analysis Result for XXIII

The Crystal Predictor search within Z'=1 was successful in locating all the Z'=1 forms. There was no explicit search in Z'=2, and the Z'=2 structures generated by symmetry lowering were unlikely to have been able to change conformation sufficiently to match forms C and E. The results emphasised our concern about the number of structures generated that are close in structure but differ by more than 2.5 kJ mol^{-1} of the automatic clustering for List1.

Our results had form A outside the lists, as A2607 rank #284 (ΔE_{latt}~ 13.5 kJ mol^{-1}) on List1 (with the experimental structure minimized with the same computational model at ΔE_{latt}~ 10 kJ mol^{-1}) and #167 in List2. Form B was found #1 and #2 on the lists as structure A70, i.e. ΔE_{latt} = 0 and ~ 1.7 kJ mol^{-1}. Form D was found as structure A505, #85 on List1 and #44 on List2 (both ΔE_{latt} ~ 9 kJ mol^{-1}). Hence, the Sheet8 polymorphs, A and D, are only just within the likely energy range for conformational polymorphs, and probably too high in energy considering the small differences in conformations(Cruz-Cabeza *et al.*, 2015). Forms C and an ordered model of E are at reasonable energies. We have got a structural model for E which fails on the second derivatives, which may correlate with the large thermal

ellipsoids, and means that there is no list 2 energy for form E. The experimental information implies that form A is the most stable in lattice energy, in which case the energy ranking is definitely poor. However, it is notable that most successful submissions ranked A and D higher in energy than form B. Further details of the slurrying experiments and other work to determine the relative stability is expected. Since A and D have the same Sheet8, and B and C have the same Sheet2, slurrying would readily interconvert A and D, but may not convert either to forms B, C or E. However, it does seem likely that there is a significant error in our energy model in the relative energies of the two observed sheets. The observation that many structures are based on different stackings of the same sheet, as indeed are seen in observed polymorph families (A and D) and (B,C and E), suggests the possibility of disorder in stacking particularly in microcrystalline samples.

Since Form A XXIII was found in the search, but not in the lists, here are is the corresponding entry for SI Table 5

	Rank	List	Δa / %	Δb /%	Δc /%	Δβ /%	ΔVolume /%	ΔDensity /%	$RMSD_{20}$ /Å	$RMSD_{1}$ /Å
Price	284	1	1.89	2.31	-6.63	-2.75	-2.32	2.39	0.972*	0.3828
	167	2	1.80	2.38	-6.74	-2.71	-2.46	2.53	0.973*	0.3828

* 31% and 31° tolerances used in structure matching

Following release of the experimentally determined structures, each was minimized with the same computational model as used for the submission of the predictions. These minima did not correspond exactly to the structure generated in the search, so both are given.

Table 2. Relative energies of computationally minimized experimental structures and corresponding search structures.

Polymorph	List1 relative energy / kJ mol^{-1}		List2 relative energy / kJ mol^{-1}	
	Minimized experimental	Search structure	Minimized experimental	Search structure
Form A	+10.0	+13.5 (#284)	+9.8	+13.6 (#167)
Form B	+0.6	Global minimum (#1)	+2.4	+1.7 (#2)
Form C	+3.9		+4.2	
Form D	+9.8	+9.2 (#85)	+9.5	+9.3 (#44)
Form E	+5.9		Fails 2nd derivs.	

Table 3. Quality of reproduction of computationally minimized experimental structures of XXIII in Price lists in terms of the relative deviation in lattice parameters, volume and density: ((pred. − expt.)/expt.) × 100%. The root mean squared deviation for the overlay of matching clusters of 20 molecules (RMSD20) and the overlay of the experimental and predicted conformations (RMSD1) are also given in Å. Experimental values for lattice parameters, unit-cell volume and density are reported in Å and, $Å^3$ and g/cm^3, respectively.

	List	a	b	C		β		Volume	Density	$RMSD^{20}$	$RMSD^{1}$
Form A T=300K		11.1637(10)	10.5295(10)	16.2358(15)		95.749(2)		1898.9(3)	1.351		
	1	7.06	-5.77	-2.29		0.13		-1.44	1.48	0.657	0.214
	2	7.01	-5.76	-2.51		0.03		-1.69	1.73	0.659	0.214
Form B T=300K		7.0061(13)	7.8047(15)	18.893(4)	85.277(4)	80.753(4)	65.769(3)	929.7(3)	1.380		
	1	2.52	0.86	-4.33	4.51	1.36	-1.47	-1.66	1.69	0.475	0.631
	2	2.50	0.76	-4.33	4.61	1.45	-1.50	-1.78	1.80	0.479	0.631
Form C T=300K		7.6375(11)	12.0393(17)	20.443(3)	84.790(3)	85.379(3)	80.091(3)	1840.0(5)	1.394		
	1	-0.43	-0.54	-1.40	1.44	4.30	3.55	-1.24	1.29	0.340	-
	2	-0.54	-0.78	-1.36	1.65	4.43	3.72	-1.51	1.56	0.349	-
Form D T=300K		13.886(4)	10.728(3)	14.078(4)		113.632(5)		1921.3(9)	1.335		
	1	3.19	-0.07	-1.13		2.18		-0.07	0.10	0.360	0.121
	2	3.07	-0.22	-1.29		2.16		-0.49	-0.52	0.360	0.121
Form E T=292K		6.687(8)	12.180(18)	24.39(4)	102.88(3)	96.69(2)	97.215(15)	1899(5)	1.351		
	1	0.57	-3.14	0.18	-2.11	1.68	-0.41	-1.87	1.92	0.385	-
	2	0.46	-3.05	0.23	-2.03	1.73	-0.57	-1.83	1.87	0.381	-

XXIV – Rui Guo

This has the challenge of being both a salt and a hydrate, with 3 independent components in the asymmetric unit. Moreover, the cation, with a carboxylic acid and a thiouronium group (-S-C$(NH_2)_2^+$), has many possible hydrogen bonding possibilities. The cation is reasonably flexible, particularly about the two torsion angles θ_1 and θ_2, and the COOH group can adopt two orientations with either the C=O or the C-OH adjacent to the -S- atom, with gas-phase conformational analysis showing the former to be much more stable. The cation can also adopt a conformation with an intra-molecular hydrogen-bond between the –COOH and the thiouronium group, which is the most stable in isolation but has reduced intermolecular hydrogen bonding capability. All these conformational regions were included in the CrystalPredictor search for the sake of completeness. Searches were deemed sufficiently complete as most low energy structures were found hundreds of times.

The standard FIT potential, with the -S- sulphur potential and using H_N and O for water(Hulme & Price, 2007), was augmented by the Cl^- potential kindly provided by GM Day. This led to more realistic distances between chloride ions and its ligands than assuming the same *exp-6* parameters as organic chlorine. Clustering with three independent components in the asymmetric unit could not use the default 15 molecule cluster; even a 30-molecule cluster sometimes failed to differentiate between crystal structures with different stacking of layers. Thus 50-molecule clusters were used to determine unique structures. It was observed in creating List1 that some structures which had a very good $RMSD_{50}$ <0.1 Å corresponded to energy differences greater than the 0.5 kJ mol^{-1} used as the first screen for removal of duplicates, showing the increased sensitivity of the energy to details of the structure.

No intermediate CrystalOptimizer run was performed as test calculations showed that re-ranking in lattice energies through inclusion of higher multipoles was less important than the changes in conformation of the cation induced by the packing forces, and full CrystalOptimizer runs were relatively inexpensive. Many structures beginning in Region B with an intramolecular hydrogen bond changed to break this hydrogen bond to form an intermolecular one (and hence be similar to Region A structures) within CrystalOptimizer optimization. Free energy corrections for lattice energies were attempted, however the current version of DMACRYS failed for such a 3-component system, possibly due to the existence of the chloride ion.

There is a considerable variety in the structures generated, although all have the Cl^- coordinated by the cation CO_2H and NH_2, and water. Many structures form chains of $Cl^-\cdots H_2O\cdots Cl^-$. Some structures are very similar except that the Cl^- and H_2O have swapped places. There are various different layer structures.

The polarization within the salts is only crudely estimated by using a polarizable continuum model PCM, ε=11. However, both this and changing to the W99 potential had a huge effect on the relative rankings, particularly the PCM model made the conformational energies of the two carboxylic acid conformations more equal. This means that there is a huge uncertainty in the relative energies, mainly because of inadequate treatment of polarization, but also uncertainty in the *exp-6* parameters (a few structures optimized to unrealistic $H_2O\cdots$H-OCO distances with W99 and were excluded.) Hence there are relatively few structures in common between the two lists.

Post Analysis Result for XXIV The experimental structure was not found in the search, but this was because one torsion angle range was 5° too small. The number of structures generated was huge, giving rise to clustering issues and complicating the reranking process. However, all the lattice energy models based on isolated ion calculations were far too poor for this salt. The experimental structure was 42 kJ/mol above the global minimum in List 1.

XXV – Rona E. Watson

The initial conformational analysis of both components of the XXV cocrystal confirmed that both components could be considered as rigid in the search which initially generated 1,000,000 structures using CrystalPredictor1.8. This gave inadequate coverage of the additional search space that is required when the relative positions of the two components provide additional variables, judging from the number of times the low energy structures were found. Hence an additional 1,000,000 structures were generated, one of which proved to be the lowest energy structure. We are not confident that the search is complete.

Full atomistic CrystalOptimizer calculations were performed on the crystal structures of the individual components of XXV from the CSD which determined the 17 degrees of freedom, shown in Figure 1, that were optimised in all CrystalOptimiser calculations. We had expected the potential model to be adequate from our experience of this type of molecule, but initially the Troger's base AXAGEL structure was poorly reproduced. However, the redetermination AXAGEL01 which appeared during the study was well reproduced. Although we are well aware of the limitations of the FIT parameters, and were unable to use the W99 potential in conjunction with a DMA for describing the $CO_2H\cdots N$ hydrogen bonds, the energy gaps between the structures suggested that the potential should be reasonable for relative rankings. Adding a polarizable continuum gave some reranking, as did adding the rigid body free energy estimate. (The free energy could not be calculated for 10% of the structures, which were therefore omitted from List2). However, the energy gaps between the lower energy structures were sufficiently large that the reranking among the likely candidates was fairly limited.

All the low energy structures generated contained the expected $CO_2H\cdots N$ hydrogen bond. It was notable that many of the cocrystals had layers of Troger's base linked by layers of the coformer. Similarly, there were layers of the carboxylic acid coformer that were the same in various structures linked by different orientations of the Troger's base. There was a large variety of $\pi\cdots\pi$ stacking geometries between each component and between the components in the crystal structures. There was a large range of different orientations of the molecules relative to the approximately linear O-H···N hydrogen bond. Hence, few structures could be eliminated as being too similar to be able to remain distinct during crystallization. The cocrystal was estimated to be significantly more stable than the components (~ 25 kJ mol^{-1}), and the slow evaporation conditions suggest thermodynamic control of the crystallization. Hence, the two lists are based on the energy rankings with different models. The global minimum $P2_1/c$ structure (with both energy models) seems likely to be the target crystal structure if our search space coverage is sufficient and the crystallization is under thermodynamic control.

Post Analaysis Result for XXV Correct prediction; the experimental structure was the global minimum in both lists. The challenge for XXV was mainly in the extent of the search for the crystal structure generation. The lattice energy model was adequate for discriminating between the possible structures.

Further crystallographic work showed proton disorder at low temperature. Our methodology was restricted to finding cocrystals with the assumed chemical bonding, and so could not show the possibility of it forming a salt or disordered structure. However, even methods that in principle can allow a proton transfer may not do so if there is an energy barrier to transfer. Crystal structure prediction studies on both the salt and cocrystal of some pyridine carboxylic acids(Mohamed *et al.*, 2011), show that there are significant differences between the salt and cocrystal energy landscapes, though the example that was proton disordered had the observed structure as a favourable structure on both landscapes.

XXVI – Luca Iuzzolino

The CSP of XXVI was particularly challenging because of the size and flexibility of the molecule. Five torsional degrees of freedom were considered as independent, which made the search space quite large and meant that the crystal structures were generated with CrystalPredictor1.6, using torsional potentials derived from molecular fragments. Treating the flexibility of XXVI with LAMs for a 5 dimensional grid with CrystalPredictor2 would have been unfeasible. Nevertheless this estimate of intramolecular energy penalty omits steric clashes not within the model molecules, so decreases confidence in the completeness of the search. The search was only carried out in Z'=1 for cost reasons, although after symmetry reduction a few low-energy structures were found to be Z'=2. Although the flexibility and the racemic nature of XXVI decreases the chances of a crystallization with Z'>1 (Steed & Steed, 2015), this possibility cannot be ruled out and this also limits the completeness of this CSP study.

The first re-ranking was carried out with a single iteration of CrystalOptimizer, to allow the relaxation of the dependant degrees of freedom (kept fixed by CrystalPredictor1.6) and to evaluate the intermolecular interactions with more accurate distributed multipoles. Although this method is more accurate than single point DMACRYS energy evaluation, its high computational cost allowed the re-ranking of only 9400 structures, which may have led to missing out some important structures that CrystalPredictor1.6 may have erroneously considered energetically unfeasible.

A key observation was that XXVI appeared to only be able to pack in low density structures (67-70.5% packing coefficient), and only a few contained hydrogen bonds within the traditional length definition. A CSD search for similar molecules showed that most formed solvates, giving greater confidence that the lack of dense structures could be realistic. Most of the structures were unique, but ~30 structures were removed in forming List2 because they had a common layer with similar stacking. The sensitivity analysis showed that the relative energies of the structures were very sensitive to the inclusion of a polarizable continuum, particularly in reranking the hydrogen bonded structures, with a marked effect on both ΔE_{intra} and U_{inter}. The range of densities would make these structures sensitive to both inter and intramolecular dispersion effects. Using rigid body free energies is likely to be unrealistic. Hence there is low confidence in the relative energies of structures that differ in hydrogen bonding motif or major changes in conformation. However, the kinetic effect that XXVI may have lost solvent relatively late in the nucleation process means that thermodynamics may not be determining the crystal structure observed. Structure CP38 which has unconnected voids, just a bit too small to accommodate CCl_2H_2, seems a likely candidate.

Post Analysis for XXVI : The experimental structure was 2nd in list 1 and 1st in list 2., corresponding to the search structure labelled #1600.

The observed conformation has one phenyl group and attached amide group that were sufficiently far away from any isolated molecule conformational minimum, that including flexibility at the search stage was a key to the successful prediction. The energy gap between the #1600=exptal and any other generated structure was much larger after the inclusion of the PCM, implying that accounting for the polarisation of the molecule within the crystal was important in ranking the hydrogen bonded structures relative to the others.

Overall post-analysis conclusion

CrystalPredictor is capable of providing an extensive enough search for all the targets in the 6th Blind Test. The energy model, using ab initio calculations on the molecule and distributed multipole electrostatics, was adequate for the purposes of generating the observed structures within a reasonable energy range for all systems apart from the salt. These structures could be reranked with a more accurate model, such as those used by Tkatchenko or Brandenburg, as part of a hierarchical treatment or for greater confidence in assessing the uncertainty in the relative energies. The energies and rankings of the experimental structures were adequate for a reasonable interpretation of the crystal energy landscapes for most practical purposes, and for a relatively successful submission. It is pleasing that our results in this blind tests were very much in line with the degree of confidence in predicting the structures expressed prior to submission.

Computer resources

All calculations were performed by the group sharing our ancient 230 CPU cluster of mixed type, alongside other projects. The time estimate in Table 1 covers all computer processes required for producing the lists; it excludes the amount of time spent testing sensitivity to other approximations and the amount of human time spent analyzing the resulting structures with Mercury. The same calculations should take half to a third of the time on our new cluster, let alone the time savings from improved algorithms, adaption of approach for different purposes, etc.

Acknowledgements

We would like to thank Costas Pantelides, Claire Adjiman and Isaac Sugden of Imperial College for their support of our use of CrystalPredictor and CrystalOptimizer in this and other applications. The CSP work of the group is supported by EPSRC, though grant ESPRC EP/K039229/1, and Eli Lilly. The PhD students support: RKH by a joint UCL Max-Planck Society Magdeburg Impact studentship, REW by a UCL Impact studentship; LI by Cambridge Crystallographic Data Centre and the M3S Centre for Doctoral Training (EPSRC EP/G036675/1).

References

Anghel, A. T., Day, G. M. & Price, S. L. (2002). *CrystEngComm* **4**, 348-355.

Braun, D. E., McMahon, J. A., Koztecki, L. H., Price, S. L. & Reutzel-Edens, S. M. (2014). *Crystal Growth & Design* **14**, 2056-2072.
Chisholm, J. A. & Motherwell, S. (2005). *Journal of Applied Crystallography* **38**, 228-231.
Coombes, D. S., Price, S. L., Willock, D. J. & Leslie, M. (1996). *Journal of Physical Chemistry* **100**, 7352-7360.
Cossi, M., Scalmani, G., Rega, N. & Barone, V. (2002). *Journal of Chemical Physics* **117**, 43-45.
Cruz-Cabeza, A. J., Reutzel-Edens, S. M. & Bernstein, J. (2015). *Chemical Society Reviews* **ASAP**.
Goldstein, R. I., Guo, R., Hughes, C., Maurer, D. P., Newhouse, T. R., Sisto, T. J., Conry, R. R., Price, S. L. & Thamattoor, D. M. (2015). *CrystEngComm* **17**, 4877-4882.
Hulme, A. T. & Price, S. L. (2007). *Journal of Chemical Theory and Computation* **3**, 1597-1608.
Hylton, R. K., Tizzard, G. J., Threlfall, T. L., Ellis, A. L., Coles, S. J., Seaton, C. C., Schulze, E., Lorenz, H., Seidel-Morgenstern, A., Stein, M. & Price, S. L. (2015). *Journal of the American Chemical Society*.
Karamertzanis, P. G. & Pantelides, C. C. (2005). *Journal of Computational Chemistry* **26**, 304-324.
Karamertzanis, P. G. & Pantelides, C. C. (2007). *Molecular Physics* **105**, 273-291.
Kazantsev, A. V., Karamertzanis, P. G., Adjiman, C. S. & Pantelides, C. C. (2010). *Molecular System Engineering*, edited by C. S. Adjiman & A. Galindo, pp. 1-42. Weinheim: WILEY-VCH Verlag GmbH & Co.
Kazantsev, A. V., Karamertzanis, P. G., Adjiman, C. S. & Pantelides, C. C. (2011). *Journal of Chemical Theory and Computation* **7**, 1998-2016.
Kazantsev, A. V., Karamertzanis, P. G., Adjiman, C. S., Pantelides, C. C., Price, S. L., Galek, P. T., Day, G. M. & Cruz-Cabeza, A. J. (2011). *International Journal of Pharmaceutics* **418**, 168-178.
Mohamed, S., Tocher, D. A. & Price, S. L. (2011). *International Journal of Pharmaceutics* **418**, 187-198.
Nyman, J. & Day, G. (2015). *Crystengcomm* **17**, 5154-5165.
Pantelides, C., Adjiman, C., Kazantsev, A., AtahanEvrenk, S. & AspuruGuzik, A. (2014). *Prediction and Calculation of Crystal Structures: Methods and Applications* **345**, 25-58.
Price, L. S., McMahon, J. A., Lingireddy, S. R., Lau, S.-F., Diseroad, B. A., Price, S. L. & Reutzel-Edens, S. M. (2014). *Journal of Molecular Structure* **1078**, 26-42.
Price, S. L. (2013). *Acta Crystallographica Section B-Structural Science Crystal Engineering and Materials* **69**, 313-328.
Price, S. L., Leslie, M., Welch, G. W. A., Habgood, M., Price, L. S., Karamertzanis, P. G. & Day, G. M. (2010). *Physical Chemistry Chemical Physics* **12**, 8478-8490.
Reilly, A. & Tkatchenko, A. (2014). *Physical Review Letters* **113**.
Steed, K. & Steed, J. (2015). *Chemical Reviews* **115**, 2895-2933.
Stone, A. J. (2005). *Journal of Chemical Theory and Computation* **1**, 1128-1132.
Stone, A. J. (2010). *GDMA: A Program for Performing Distributed Multipole Analysis of Wave Functions Calculated Using the Gaussian Program System.* Version 2.2.
Uzoh, O. G., Cruz-Cabeza, A. J. & Price, S. L. (2012). *Crystal Growth & Design* **12**, 4230-4239.
Williams, D. E. (2001). *Journal of Computational Chemistry* **22**, 1154-1166.
Yang, J., Hu, W., Usvyat, D., Matthews, D., Schutz, M. & Chan, H. (2014). *Science* **345**, 640-643.
