# Supplementary material for: Report on the sixth blind test of organic crystal structure prediction methods
Source: Acta Crystallogr B Struct Sci Cryst Eng Mater. 2016 Aug 1;72(Pt 4):439–59. doi: 10.1107/S2052520616007447 (PMC4971545; doi:10.1107/S2052520616007447)
Supplement: Supplementary file 3 [file b-72-00439-sup3.zip › Group_19_Szalewicz_Post_Analysis.pdf]

Supplementary information for predictions of structure of the crystal built of molecule XXII

Michael P. Metz,[1] Rebecca K. Hylton,[2] Sarah L. Price,[2] and Krzysztof Szalewicz[1]

[1]*Department of Physics and Astronomy, University of Delaware, Newark, Delaware 19716, USA*

[2]*Department of Chemistry, University College London, 20 Gordon Street, London WC1H 0AJ, UK*

(Dated: July 7, 2016)

I. METHODOLOGY

The procedure consists of monomer optimization, potential (force-field) generation, molecular packing, and energy minimization.

The intermolecular potentials were constructed using symmetry-adapted perturbation theory (SAPT)[1,2] based on Kohn-Sham density-functional theory (DFT) description of monomers, SAPT(DFT)[3,4], implemented in the SAPT2012[5] package, using ORCA[6] for monomer DFT. The monomer geometry was obtained by an unconstrained PBE0+D3[7–9] minimization using Orca[6] and the aug-cc-pVTZ basis set[10]. It resulted in the bending angle around the S-S line in the six-membered ring of 140.3° (with the flat monomer corresponding to 180°) and the increase of the magnitude of the total monomer energy by nearly 5.4 kcal/mol. We have checked these findings by calculating the interaction energies with several other methods on the bending path, keeping all other geometric parameters the same as in the PBE0+D3 minimum. The highest-level theory, the coupled-cluster method with single, double, and noniterative triple excitations, CCSD(T), gave the minimum about 1 kcal/mol deeper and located at about 135°. The use of the D3 dispersion-energy correction resulted in only a minor improvement as the PBE0 method predicted essentially the same angle and energy about 0.3 kcal/mol higher than PBE0+D3. In all SAPT(DFT) calculations of interaction energies, the monomers were held rigid at the PBE0+D3-optimized geometry. In some of these calculations, we used the mirror reflections of such monomer. We will use symbols L and R for the two enantiomers. In crystal-structure predictions, the mirror symmetry was applied to monomers, so each monomer could become an L or R enantiomer. The intermolecular potentials had the same parameters for the LL, RR, and LR dimers.

Two potentials were constructed, each using the functional form

$$V = \sum_{a\in A}\sum_{b\in B} u_{ab}(r_{ab}) \tag{1}$$

where a and b are atoms in monomers A and B, respectively, and r_{ab} is the distance between them. The isotropic atom-atom function u_{ab} has the form

$$u_{ab}(r_{ab}) = e^{\alpha_{ab}-\beta_{ab}r_{ab}} + \frac{q_a q_b}{r_{ab}} - \frac{\sqrt{C_a C_b}}{(r_{ab})^6} \tag{2}$$

where α_{ab}, β_{ab}, q_i, and C_i are optimized free parameters.

The first potential, denoted `exp-6_com1`, was constructed for use with the CrystalPredictor program[11–14] and applies arithmetic combination rules for the exponential parameters:

$$\alpha_{ab} = \alpha_a + \alpha_b \tag{3}$$

$$\beta_{ab} = \beta_a + \beta_b. \tag{4}$$

The second potential is denoted `LL+LR+crys` and was constructed for use with the DMACRYS program[15]. It uses a more general form with separate α_{ab} and β_{ab} parameters for each atom pair.

The potential `exp-6_com1` was fitted using a data set of 981 dimer configurations. These configurations were generated using a Monte Carlo type importance sampling algorithm based on an OPLS-AA[16] guiding potential. A large grid of dimer configurations was first randomly generated, and then each configuration was accepted or rejected with probability

proportional to $\exp(-\frac{E_{\mathrm{guide}}}{12.0\,\mathrm{kcal/mol}})$, where E_{guide} is the interaction energy given by the guiding potential. This resulted in 871 configurations. These points were then fitted with an initial potential, which was iteratively improved computing additional points until the total set of 981 grid points was reached. The details of this iterative procedure will be described in Ref. 17. SAPT(DFT) interaction energies with δ_{HF} correction[18] were computed at each of these dimer configurations using the aug-cc-pVDZ basis[10] and the PBE functional[7] for DFT calculations.

The potential `LL+LR+crys` was fitted to a larger data set of 1423 dimer configurations. In addition to the original 981 points, each of which contain two monomers of the same chirality, 400 points were computed in which the monomers have opposite chirality (LR dimers), using the same Monte Carlo method. This set was further augmented with 42 dimer configurations taken from earlier crystal structure predictions for a total of 1423 points.

Parameterization was performed using a procedure similar to that used in an earlier work on the cyclotrimethylene trinitramine (RDX) dimer, described in Ref. 19. The parameterization is divided broadly into asymptotic and short-range fitting. In the asymptotic fitting stage, the electron density and static and dynamic polarizabilities of the monomer are expressed as multipole expansions about the monomer's center of mass. This expansion is then used to compute electrostatic, induction, and dispersion interaction energies on a grid of 10,000 dimer configurations, with monomer's closest-contact atoms separated by at least 15 Å. The C_i and q_i parameters are then optimized using this grid of asymptotic interaction energies. This stage is described in detail in Ref. 19.

In the short-range fitting stage, all remaining free parameters, α_i and β_i or α_{ab} and β_{ab}, are optimized using the set of SAPT(DFT) total interaction energies, while keeping all other parameters fixed. This final optimization is performed in multiple stages, and under a set of constraints designed to produce physically meaningful parameter values. The procedure will be described in detail in Ref. 17. On the 881 training dimer configurations with attractive interactions, the root-mean square errors (RMSEs) of the potentials `exp-6_com1` and `LL+LR+crys` are 1.0 and 0.61 kcal/mol, respectively. These RMSE's are larger than one can achieve with more elaborate functional forms, but the forms used are as elaborate as the CrystalPredictor and DMACRYS allow. The RMSE's are probably of similar size to the errors of *ab initio* calculations resulting from theory level and basis set truncations, whereas one desires the former to be several times smaller than the latter. Since the University of Delaware group has now the capability of generating very accurate potentials of more elaborate forms, and moreover such potentials can be generated in an automated way, with a minimal input from users, it becomes important for the field of crystal structure predictions that programs such as the CrystalPredictor or DMACRYS are extended to accept more elaborate potential forms.

The initial crystal structures were generated using version 1.6 of CrystalPredictor[11–14] and the potential `exp-6_com1`. The monomers were rigid except for the mirror inversion at the geometry optimized as described above. Otherwise the search was done in the same way as in another submission for system XXII by Price and Hylton.

The next stage was an improvement in the energy rankings by using the refined custom potential `LL+LR+crys` and the lattice energy minimization program DMACRYS[15]. The DMACRYS minimization was performed for the 1,000 lowest energy crystals from the CrystalPredictor search. The 100 crystals ranked according to DMACRYS lattice energies are submitted to the blind test. The file numbers on the ranking list reflect the CrystalPredictor rankings.

II. POST-SUBMISSION ANALYSIS

The experimental structure was found ranked 139 in the search with the `exp-6_com1` potential, 11 kJ/mol above the global minimum of the lattice energy. The ranking deteriorated to 162 with `LL+LR+crys` potential, 12.6 kJ/mol above global minimum. Both potentials seem to favor the structures with molecules forming chains rather than molecules clasped into dimers. Since the similar potentials used in submissions 17 and 20, based on the same set of *ab initio* points, were successful in identifying the experimental structure, it appears that the simpler potential form, leading to higher RMSEs in the present case, was responsible for this behavior. In particular, the lack of damping in `exp-6_com1` might lead to relatively large errors on the repulsive wall of the potential. The deterioration of the ranking when moving to the more flexible potential form in `LL+LR+crys` was most likely due to a mistake in calculations of the additional SAPT(DFT) points used to generate `LL+LR+crys`.

III. COMPUTER RESOURCES

A SAPT(DFT) calculation including the $\delta_{\rm HF}$ correction took about 23 wall time hours per grid point on two 2.5 GHz Intel Ivy Bridge cores. The entire set of 1423 dimer interaction energies required about 66000 CPU hours. Other steps of potential generation were negligible in comparison. The predictions with the CrystalPredictor and DMACRYS programs required negligible amounts of computer time compared to that of SAPT(DFT) runs.

ACKNOWLEDGMENTS

RKH and SLP would like to thank Costas Pantelides, Claire Adjiman, and Isaac Sugden of Imperial College for their support of the use of CrystalPredictor. RKH was funded by a joint UCL Max-Planck Society Magdeburg Impact studentship. The potential generation work at the University of Delaware was supported by the Army Research Office under Grant W911NF-13-1-0387 and by the National Science Foundation Grant CHE-1152899.

[1] B. Jeziorski, R. Moszyński, and K. Szalewicz, Chem. Rev. **94**, 1887 (1994).

[2] K. Szalewicz, Wiley Interdisc. Rev.–Comp. Mol. Sci. **2**, 254 (2012).

[3] A. J. Misquitta, B. Jeziorski, and K. Szalewicz, Phys. Rev. Lett. **91**, 033201 (2003).

[4] A. J. Misquitta, R. Podeszwa, B. Jeziorski, and K. Szalewicz, J. Chem. Phys. **123**, 214103 (2005).

[5] R. Bukowski, W. Cencek, P. Jankowski, M. Jeziorska, B. Jeziorski, S. A. Kucharski, V. F. Lotrich, A. J. Misquitta, R. Moszyński, K. Patkowski, R. Podeszwa, F. Rob, S. Rybak, K. Szalewicz, H. L. Williams, R. J. Wheatley, P. E. S. Wormer, and P. S. Żuchowski, "SAPT2012: An *ab initio* program for many-body symmetry-adapted perturbation theory calculations of intermolecular interaction energies," University of Delaware and University of Warsaw (2012).

[6] F. Neese, "*ORCA: An Ab Initio, DFT, and Semiempirical electronic structure package*," with contributions from U. Becker, D. Ganyushin, A. Hansen, D. Liakos, C. Kollmar, S. Kossmann, T. Petrenko, C. Reimann, C. Riplinger, K. Sivalingam, B. Wezisla, and F. Wennmohs.

[7] J. P. Perdew, K. Burke, and M. Ernzerhof, Phys. Rev. Lett. **77**, 3865 (1996).
[8] C. Adamo and V. Barone, J. Chem. Phys. **110**, 6158 (1999).
[9] S. Grimme, J. Antony, S. Ehrlich, and S. Krieg, J. Chem. Phys. **132**, 154104 (2010).
[10] R. A. Kendall, T. H. Dunning, Jr., and R. J. Harrison, J. Chem. Phys. **96**, 6796 (1992).
[11] P. G. Karamertzanis and C. C. Pantelides, J. Comp. Chem. **26**, 304 (2005).
[12] P. G. Karamertzanis and C. C. Pantelides, Mol. Phys. **105**, 273 (2007).
[13] A. V. Kazantsev, P. G. Karamertzanis, C. S. Adjiman, and C. C. Pantelides, J. Chem. Theory Comput. **7**, 1998 (2011).
[14] C. Pantelides, C. Adjiman, and A. Kazantsev, Topics Curr. Chem. **345**, 25 (2014).
[15] S. L. Price, M. Leslie, G. W. A. Welch, M. Habgood, L. S. Price, P. G. Karamertzanis, and G. M. Day, Phys. Chem. Chem. Phys. **12**, 8478 (2010).
[16] W. L. Jorgensen, D. S. Maxwell, and J. Tirado-Rives, J. Am. Chem. Soc. **118**, 11225 (1996).
[17] M. P. Metz, K. Piszczatowski, and K. Szalewicz, (2015), manuscript in preparation.
[18] K. Szalewicz, K. Patkowski, and B. Jeziorski, *Intermolecular Forces and Clusters*, Structure and Bonding **116**, 43 (2005).
[19] R. Podeszwa, R. Bukowski, B. M. Rice, and K. Szalewicz, Phys. Chem. Chem. Phys. **9**, 5561 (2007).
