# Supplementary material for: Report on the sixth blind test of organic crystal structure prediction methods
Source: Acta Crystallogr B Struct Sci Cryst Eng Mater. 2016 Aug 1;72(Pt 4):439–59. doi: 10.1107/S2052520616007447 (PMC4971545; doi:10.1107/S2052520616007447)
Supplement: Supplementary file 3 [file b-72-00439-sup3.zip › Group_20_Tuckerman_Szalewicz_Post_Analysis.pdf]

Crystal Structure Prediction from Molecular Dynamics and Enhanced Sampling using Density Functional Based Symmetry Adapted Perturbation Theory (SAPT(DFT)) Potentials

Leslie Vogt,[1] Elia Schneider,[1] Michael P. Metz,[2]
Mark Tuckerman,[1,3] and Krzysztof Szalewicz[2]

[1]*Department of Chemistry, New York University, New York, New York 10003, USA*

[2]*Department of Physics and Astronomy, University of Delaware, Newark, Delaware 19716, USA*

[3]*Courant Institute of Mathematical Science, New York University, New York, New York 10003, USA*

I. FORCE FIELD PARAMETERS

SAPT(DFT) Potential

Geometry Optimization

The monomer geometry was obtained using density functional theory (DFT) with an unconstrained PBE0+D3[1–3] minimization using Orca[4] and the aug-cc-pVTZ basis set[5]. The optimization resulted in bending around the S-S line in the six-membered ring of about 40°, with the flat monomer higher in energy by about 5.4 kcal/mol. We checked these findings by calculating the energies with several other methods on the path from the flat monomer to the minimum-energy bend structure, keeping all other geometric parameters the same as in the PBE0+D3 minimum. The highest-level theory, the coupled-cluster method with single, double, and noniterative triple excitations, CCSD(T), gave the minimum about 1 kcal/mol deeper and located at about 45°. The use of the D3 dispersion-energy correction resulted in only a minor improvement as the PBE0 method predicted essentially the same angle and energy about 0.2 kcal/mol higher than PBE0+D3. In all SAPT(DFT) calculations of interaction energies, the monomers were held rigid at the PBE0+D3-optimized geometry. In some of these calculations, we used the mirror reflections of such monomer. In crystal-structure predictions, the mirror symmetry was applied to monomers, so each monomer could become either enantiomer.

Dimer Calculations

The intermolecular potentials were constructed using symmetry adapted perturbation theory (SAPT)[6,7] based on Kohn-Sham DFT description of the monomers, SAPT(DFT)[8,9], implemented in the SAPT2012[10] package, using ORCA[4] for monomer DFT.

A set of 1381 dimer configurations was used as the primary training data. Of these, 981 are configurations where both monomers are of the same chirality, and the remaining 400 contain monomers of opposite chirality (note that the intermolecular potentials had the same parameters for the dimers consisting of monomers of both types). These configurations were generated using a Monte Carlo type algorithm with importance sampling based on an OPLS-AA[11] guiding potential. A large grid of dimer configurations was first randomly

generated, and then each configuration was accepted or rejected with probability proportional to $\exp[-E_{\rm guide}/(12.0\,{\rm kcal}\cdot{\rm mol}^{-1})]$, where $E_{\rm guide}$ is the interaction energy given by the guiding potential. This resulted in 871 configurations. These points were then fit with an initial potential, which was iteratively augmented with additional points until the total set of 1381 grid points was reached. The details of this iterative procedure is described in Ref. 12. SAPT(DFT) interaction energies with $\delta_{\rm HF}$ correction[13] were computed at each of these dimer configurations using the aug-cc-pVDZ basis[5] and the PBE functional[1] for DFT calculations.

Parameter Fitting

Two potentials were constructed, both using the functional form

$$V = \sum_{a\in A}\sum_{b\in B} u_{ab}(r_{ab}) \tag{1}$$

where a and b are atoms in monomers A and B, respectively, and r_{ab} is the distance between them. The form of the isotropic atom-atom function u_{ab} varies between the different potentials.

The first potential, denoted `poly0`, was constructed for use with UPACK[14] and has the form

$$u^1_{ab}(r_{ab}) = e^{\frac{1}{2}(\alpha_a+\alpha_b)-\frac{1}{2}(\beta_a+\beta_b)r_{ab}} + \frac{q_a q_b}{r_{ab}} - f_6(\sqrt{\delta_a\delta_b}, r_{ab})\frac{\sqrt{C_a C_b}}{(r_{ab})^6} \tag{2}$$

where α_{ab}, β_{ab}, q_i, δ_i and C_i are optimized free parameters and f_6 denotes the Tang-Toennies type damping function[15]. The second potential is denoted `poly1` and has a more complex form

$$u^2_{ab}(r_{ab}) = [1 + a_{ab}r_{ab}]\, e^{\frac{1}{2}(\alpha_a+\alpha_b)-\frac{1}{2}(\beta_a+\beta_b)r_{ab}} + \frac{q_a q_b}{r_{ab}} - f_6(\sqrt{\delta_a\delta_b}, r_{ab})\frac{\sqrt{C_a C_b}}{(r_{ab})^6} \tag{3}$$

which includes the additional free parameters a_{ab}.

Parameterization was performed using a procedure similar to that used in an earlier work on the cyclotrimethylene trinitramine (RDX) dimer, described in Ref. 16. The parameterization is divided broadly into asymptotic and short-range fitting. In the asymptotic fitting stage, the multipole moments and static and dynamic polarizabilities of the monomer are used to construct multipole expansions about the monomer's center of mass. These expansions are then used to compute electrostatic, induction, and dispersion interaction energies

on a grid of 10,000 dimer configurations, with monomer's closest-contact atoms separated by at least 15 Å. The C_i and q_i parameters are then optimized using this grid of asymptotic interaction energies. This stage is described in detail in Ref. 16.

In the short-range fitting stage, all remaining free parameters are optimized using the set of SAPT(DFT) interaction energies. The δ_i parameters are first optimized to reproduce the sum of induction and dispersion SAPT components, and then finally the α_i, β_i, and a_{ab} parameters are optimized to reproduce the total SAPT interaction energy while keeping all other parameters fixed. This final optimization is performed in multiple stages and under a set of constraints designed to produce physically meaningful parameter values. This procedure is described in detail in Ref. 12. On the 839 training dimer configurations with attractive interactions, the root mean square errors (RMSEs) of the potentials `poly0` and `poly1` are 0.66 and 0.41 kcal/mol, respectively.

OPLS

For initial generation of crystal structures with rigid monomers, we used the SAPT(DFT) charges (Table A.1) and van der Waals (vdW) parameters from the OPLS-AA force field with geometric combination rules (Table A.2)[11,17]. Molecular dynamics simulations with flexible monomers used OPLS-AA parameters for intramolecular potentials, including bond, bend, torsion, and 1-4 interactions (Table A.2). Most of the intramolecular force field parameter have been assigned using acpype software[18], except some bend and torsional potentials, which were updated to reflect the OPLS-AA parameters for aryl nitrile groups (Table A.3)[11,17]. Dihedrals in the 6-membered dithiin ring were parameterized using a power-series potential to reproduce the 5.4 kcal/mol barrier for ring inversion (Table A.4).

II. CRYSTAL STRUCTURE GENERATION

To generate the initial set of structures for target XXII, we modified the UPACK program suite[14] to use the modified Buckingham form of the `poly0` SAPT(DFT) potential. This modification adds a damping term to the real-space evaluation of the Coulomb and dispersion interactions (see Section I), although only the C_6 damping was used in this work.

Randomly generated structures can produce short interatomic distances that are not well-

described by the functional form of the SAPT(DFT) potential. Therefore, we performed initial packing with the OPLS force field, with subsequent optimization using the `poly0` SAPT(DFT) potential, as detailed below.

Random Packing

The `pack12` program in UPACK was used to generate the initial set of crystal structures for analysis. For target XXII, we used rigid monomers with geometry determined as described in Section I. The initial packing was performed using OPLS vdW parameters (Table A.2) and charges from the SAPT(DFT) potential (Table A.1). Structures were generated with an external pressure of 1 bar for the 13 most common space groups ($P2_1/c$, $P\bar{1}$, $P2_12_12_1$, $P2_1$, $Pbca$, $C2/c$, $Pna2_1$, Cc, $Pca2_1$, $C2$, $P1$, $Pbcn$, Pc), plus 7 additional space groups based on preliminary screening ($P2_1/n$, Ia, $I2/a$, $I2/c$, Pn, $Pnma$, $C2/m$).

For each space group, the unit cell volume was estimated from the 1.1 x the smallest volume found for 100 initial structures. Lattice energies were determined using a cutoff of 12 Å, with Ewald damping $\alpha = 3$ nm^{-1} and reciprocal space cutoff of 2 nm^{-1} for both Coulomb and dispersion terms. The initial guesses were minimized using the default UPACK steepest descent (SD) and conjugate gradient (CG) steps. For each space group, 1,000 random unit cells were generated, with structures retained if the energy was within 40 kJ/mol of the current lowest energy. Structures were clustered using the `dist` program in UPACK to compare radial distribution functions for each atom in the monomer using a cutoff of 5 Å and a tolerance of 0.05 Å.

If the lowest energy structure in each space group did not have a cluster size of at least 5, more structures were generated. This resulted in 48,000 total structures in the initial set (2,000 each for $P2_1/c$ and $Pca2_1$; 4,000 for $P2_1/n$; 5,000 each for $C2/c$, Cc, $I2/a$, $I2/c$). After clustering in each space group, the remaining 13,958 unit cells represented 10,664 unique structures in the initial set.

Minimization and Clustering with SAPT(DFT) Potential

Using the `pack3` program in UPACK, structures within 30 kJ/mol of the lowest energy in each space group were further optimized using the `poly0` SAPT(DFT) potential. Each

structure was minimized using the default SD and 10,000 CG steps with a cutoff of 12 Å, with Ewald damping $\alpha = 3$ nm^{-1} and reciprocal space cutoff of 3 nm^{-1} for both Coulomb and dispersion terms. Clustering structures within each space group resulted in 9,837 unit cells that represented 8,362 unique structures in the `poly0` set. There were 131 structures within 10 kJ/mol of the lowest energy crystal structure.

III. MOLECULAR DYNAMICS SIMULATIONS

Following unit cell generation, we ran molecular dynamics (MD) for the 308 lowest energy unit cells from the UPACK ranking. We modified the PINY_MD package[19] to run MD using the SAPT(DFT) potential with first order polynomial terms (see Section I) for the intermolecular interactions. These simulations use flexible monomers with intramolecular terms, including 1-4 vdW interactions, from the OPLS force field (see Section I). Charges on each atom are from the SAPT(DFT) potential (Table A.1).

Crystal Structure Minimization

Supercells of 512 molecules were constructed from the unit cells such that no side was < 30 Å in length. Each system, including cell parameters, was minimized using the `poly0` potential, then minimized using the `poly1` potential. Energies were evaluated with a intermolecular cutoff of 12 Å and Coulomb terms used a Particle Mesh Ewald summation (alpha $= 3.75$ nm^{-1}, kmax $= 0.55$ nm^{-1}). The minimized `poly1` structures were clustered and re-ranked using the UPACK RDF clustering algorithm with a tolerance of 0.025 nm. The relative energies for some of the structures are shown in Table I. Minimization using `poly1` results in 171 unique structures.

Molecular Dynamics: Equilibration and Thermalization

The minimized structures were then evaluated for thermal stability using MD with the `poly1` SAPT(DFT) intermolecular potential. For each structure, a 20 ps constant volume isothermal (NVT) simulation with massive Nosé-Hoover chain (NHC)[20] thermostatting at $T = 300$ K (length $= 4$, $\tau = 20$ fs, Suzuki-Yoshida order $= 7$, MTS $= 4$) was used to

Rank	Space Group	ΔE (kJ/mol)	Min Rank	ΔE_{min} (kJ/mol)
1	$P2_1/n$		1	
2	$Pbca$	+3.46	4	+4.45
3	$P2_1/c$	+5.37	2	+3.97
4	$P2_1/n$	+5.99	5	+4.76
5	$P2_1/c$	+6.31	3	+4.43
			135	+22.55
6	$P2_1/n$	+7.79	9	+10.12
7	$P2_1/n$	+8.02	6	+4.76
8	$P2_1/c$	+9.20	105	+19.44
9	$P2_1/n$	+9.61	8	+9.96
			10	+10.62
			118	+19.86
10	$P\bar{1}$	+10.15	91	+17.73

TABLE I. XXII: Lowest energy structures for the `poly1` SAPT(DFT) potential. Final rankings (left) include thermal fluctuations. The preliminary rank of each structure after minimization is indicated on the right.

equilibrate the atomic velocities. Subsequently, 40 ps flexible-cell isothermal-isobaric (NPT) simulations were run at $T = 300$ K and $P = 1$ bar using the MTK barostat[21–24] ($\tau = 500$ fs) and a NHC barostat thermostat ($\tau = 100$ fs). We employed the r-RESPA multiple time step approach[25] to calculate slowly varying forces every n steps, with a base time step of 1.0 fs (bond/bends $n = 6$, torsions $n = 4$). Systems were equilibrated for 15 ps before further analysis.

Molecular Dynamics: Production Runs

The potential energy per molecule was averaged over 25 ps of NPT simulations for each supercell, and the structures are then re-ranked based on those values. The last snapshot of each supercell simulation was reduced to an averaged unit cell and the space group checked using the PLATON program[26]. After this ranking, we eliminated the first 6 structures

in which unphysical monomer deformations led to energies more than twice as low any previously observed values. MD simulations run with the `poly0` potential were stable, however, and so we report these structures as *not ranked* at the end of the .cif file. There are 165 unique structures within $\sim$ 30 kJ/mol of the lowest energy.

During the 25 ps flexible-cell NPT simulations, we observed small changes in the cell size and shape and in a few cases, changes in the space group. Therefore, additional 100 ps of flexible-cell NPT simulations were run for the top 48 structures (all within 14.7 kJ/mol) to confirm the structural stability and improve the energy evaluation. For these structures, UPACK clustering of snapshots taken every 10 ps (RDF tolerance of 0.25 Å) indicated that each trajectory is for a unique unit cell. The final potential energy for each structure was obtained by averaging the potential energy over the whole trajectory and the submitted structures correspond to the averages of the reduced cell configuration obtained every 10 ps. The final space group is assigned using the PLATON program (see Table I).

Molecular Dynamics: Structural Stability Analysis

Standard NPT MD simulations run for 125 ps indicate that the predicted structures are local minima on the free energy surface. A number of structures observed after minimization proved to be metastable, converting during NPT simulations to a more stable structure. Some of these new structures were not found in the initial packing set (e.g. Min-Rank 105 converts to Rank 8), and some were found to be the same as other local minima (e.g. Min-Rank 8, 10, and 118 all result in the Rank 9 structure).

To gain insight into the relationship between the packing interactions, we also performed temperature-enhanced sampling of the unit cell shape and size. This Crystal-Adiabatic Free Energy Dynamics (Crystal-AFED) technique[27] allowed us to obtain a better qualitative insight into the stability of the predicted structures. Crystal-AFED is a flexible-cell NPT technique in which the cell vectors are targeted for temperature-accelerated enhanced sampling and are adiabatically decoupled from the atomic degrees of freedom. Each diagonal element is subject to its own heat bath, while off-diagonal elements are coupled to a common, independent heat bath. Both of these heat baths are maintained at a temperature $T_{\mathbf{h}} \gg T$, where T is the physical temperature. In addition, the time-scale parameter for the barostat is made sufficiently large to affect the aforementioned adiabatic decoupling. In

this way, the algorithm directly explores the Gibbs free energy surface $G(\mathbf{h}, T)$. By coupling the supercell vectors in the cell matrix $\mathbf{h}$ to heat baths having temperature $T_{\mathbf{h}} = 50,000$ K and large effective mass ($\tau_{\mathbf{h}} = 5$ ps), we promote large, slow fluctuations in the unit cell size and shape but maintain normal dynamics for the atomic degrees of freedom. We have used Crystal-AFED successfully in predicting the polymorphs from the Gibbs free energy surface of benzene at 100 K and 2 GPa pressure[27], and we are currently employing it to study polymorphism in α-resorcinol and several energetic co-crystalline systems.

We performed 100 ps Crystal-AFED simulations for the first 30 ranked structures. While these run lengths are insufficient to generate the Gibbs free energy surface, they did allow us to determine that the lowest energy structure remains stable during the enhanced sampling protocol, revisiting the same clustered geometries over the course of the trajectory and maintaining the initial space group assignment, suggesting that this structure may also be the lowest free energy structure.

COMPUTER RESOURCES

A SAPT(DFT) calculation including the δ_{HF} correction point took about 23 wall time hours per grid point on two 2.5 GHz Intel Ivy Bridge cores. The entire set of 1423 dimer interaction energies took in total about 66,000 CPU hours. Other aspects of potential generation were negligible in comparison. Running UPACK on an AMD Athlon II X4 620 (2.6 GHz) processor required 215 CPU hours for initial structure generation and 15 hours for SAPT(DFT) `poly0` optimization. MD simulations were performed on Intel Xeon 2695v3 (2.3GHz) processors. The total set of about 300 supercell crystals took 15,000 CPU hours.

IV. POST-TEST ANALYSIS

Prediction Results

The comparison of the submitted list of structures with experimental one reveals that we predicted the observed experimental structure ranked 4th with $\mathrm{RMSD}_{20} = 0.187$ Å. In Fig. 1, we show the good agreement between our result and the real structure.

After the submission deadline, we obtained the additional information that the diffraction experiment was performed at 150 K. We repeated the procedure explained in Section III,

T [K]	a [Å]	b [Å]	c [Å]	β	$RMSD_{20}$ [Å]
Exp	11.947	6.696	12.598	108.6	
150	12.018	6.721	12.486	108.7	0.14
300	12.145	6.742	12.652	109.1	0.187

TABLE II. Comparison between predicted and experimental crystal cell parameters.

using the new temperature T = 150 K, for the first 10 ranked structures. The change in temperature improved the crystal structure prediction precision (we obtained a better $RMSD_{20}$ and crystal cell parameters; see Table II), although it does not affect the ranking or the energy difference.

Crystal-AFED Structure Exploration - Revisited

Using the same criteria as described in the main text, comparison of structures submitted by all groups for target XXII shows that most of our ranked structures were also submitted by other participants in the blind test. Of our top 10 structures, the 3rd, 4th (experimental structure), and 5th structures were included by most groups. Therefore, we concentrated our extended analysis on these three structures.

Review of the Crystal-AFED simulations described in Section III indicated that the energy landscape for XXII is quite rough, with low energy structures isolated in deep min-

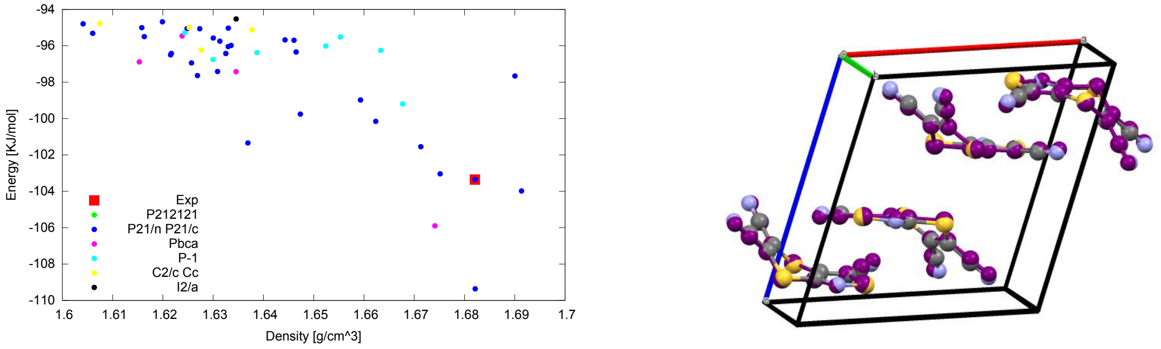

FIG. 1. On the left, density vs. potential energy per molecule for the submitted structures. On the right, the overlay between the predicted (purple) and the experimental structures (C in gray, N in blue, and S in yellow).

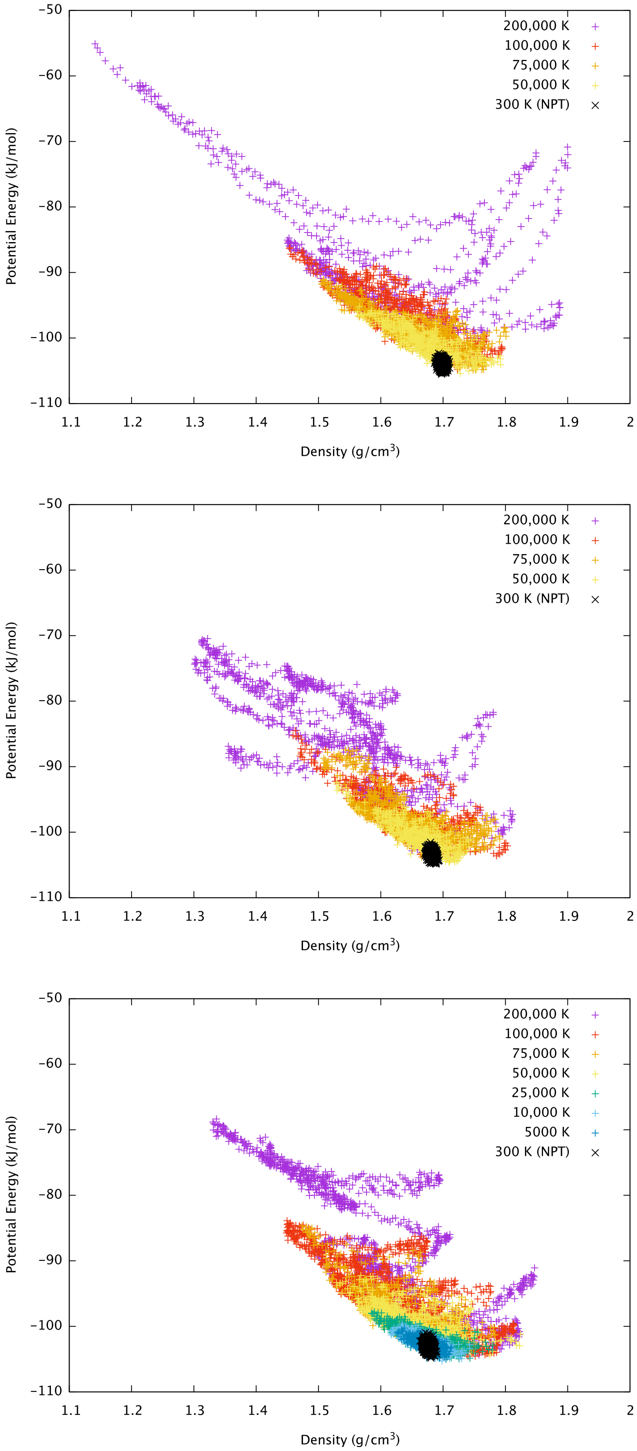

FIG. 2. Density vs. potential energy plots for a set of Crystal-AFED runs at various barostat temperatures ($T_{\mathbf{h}}$) for the structures ranked as 3rd (top plot), 4th (center plot), and 5th (bottom plot). In all three cases, we observed that the simulations can cross barriers higher than 20 kJ/mol only with barostat temperatures larger than 10^6 K.

ima. The initial Crystal-AFED simulations used a cell matrix barostat temperature of $T_{\mathbf{h}}$=50,000 K, which proved to be too low to promote structural transitions for this molecule. Fig. 2 shows that for this $T_{\mathbf{h}}$, the Crystal-AFED simulations only sample structures with potential energy $\approx$ 10 kJ/mol higher than the initial structure, which is too low to escape the minima. During the post-analysis, we ran Crystal-AFED simulations with $T_{\mathbf{h}}$ up to 200,000 K with $\tau_{\mathbf{h}} = 0.5$ ps (purple points in Fig. 2). With this higher value for $T_{\mathbf{h}}$, we improved our sampling of the high energy configurations required to find transitions away from the initial structures.

We note that although the rank 3 and rank 4 structures have similar dimer intermolecular interactions and potential energies separated by only +0.6 kJ/mol, we do not observe a transition between these structures during any of the Crystal-AFED simulations, even when using the largest $T_{\mathbf{h}}$ values. Given the similar ranking for these two structures from all groups that submitted both, the rank 3 structure may be observable as a polymorph that is unlikely to undergo a solid-to-solid phase transition to the observed rank 4 structure.

Characterizing Structural Transitions

Beyond the efficient exploration of the Gibbs free energy surface $G(\mathbf{h}, T)$, the use of Crystal-AFED allows us to find a path along which we can determine the free energy difference between structures. The Crystal-AFED technique is first used to explore the configuration space to find a transition path which connects two crystal structures. We can then reconstruct the free energy difference by calculating the free energy gradient (i.e. mean forces) for each $\mathbf{h}$-matrix visited along the simulation that connects the two different states[28,29]:

$$\begin{aligned} f_{\mu\nu}(\mathbf{h}) &= -\nabla_{\mathbf{h}}\, G(\mathbf{h}, T) \\ &= \det(\mathbf{h}) \sum_{\gamma} \langle h^{-1}_{\mu\gamma} \left(P^{(\mathrm{int})}_{\gamma\nu} - P\delta_{\gamma\nu} \right) \rangle_{\mathbf{h}} \,, \end{aligned} \tag{4}$$

where $P^{(\mathrm{int})}$ is the internal pressure. Then, by integrating the mean forces along the path we calculate the free energy difference between the initial structure and alternate structures during the Crystal-AFED simulation using:

$$\Delta G = -\sum_{\mu\nu} \int_{path} f_{\mu\nu}(\mathbf{h})\; dh_{\mu\nu} \,. \tag{5}$$

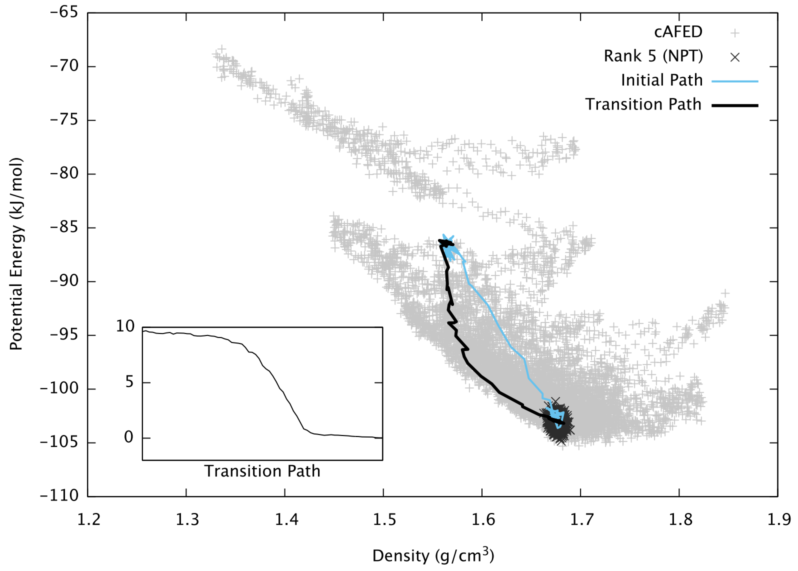

FIG. 3. The transition paths from a high energy structure to the 5th-ranked structure. The blue line corresponds to the original trajectory obtained with a standard flexible-cell NPT simulation, while the black line represents the points after convergence of the free energy gradient. Structures sampled by Crystal-AFED simulations are shown as grey points in the background. The inset shows the free energy difference in kJ/mol along the transition path computed by Eq. (5).

As an example, we identified a high-energy state (rank 135) that converts to the rank 5 structure during a standard flexible-cell NPT simulation, as reported in Table I. We extracted snapshots every 0.2 ps along this trajectory and determined the internal pressure tensor for each **h**-matrix from equilibrated NVT runs in order to converge the free energy gradient with respect to **h**-matrix at each point. Then, we integrate the free energy as a function of the **h**-matrix, following Eqs. (4) and (5). The initial path and the resulting equilibrated path are shown in Fig. 3. The free energy difference between the 5th-ranked structure and the related metastable high-energy structure is +9.6 kJ/mol.

Crystal-AFED Simulations for the Experimental Structure

To better characterize the stability of the experimental structure, we analyzed the first 80 ps of the Crystal-AFED simulation with $T_{\mathbf{h}}$=200,000 K and $\tau_{\mathbf{h}}$=1 ps. For this analysis, we extracted snapshots every 0.1 ps along the Crystal-AFED trajectory (shown as a purple

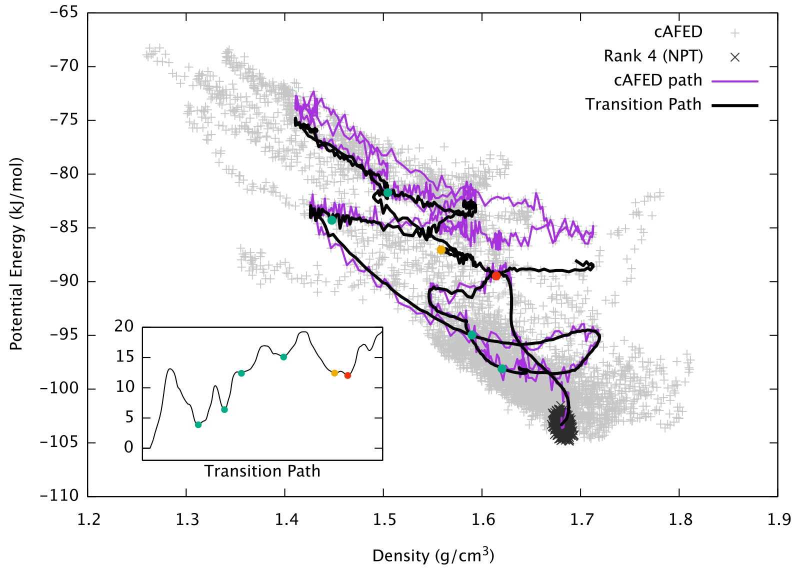

FIG. 4. The Crystal-AFED simulation path for the experimental structure with the structures visited by all Crystal-AFED simulations in grey and the standard flexible-cell NPT simulation for the rank 4 structure in black points. The purple line corresponds to the analyzed Crystal-AFED trajectory, while the black line represents the path after the convergence of the free energy gradient. The inset shows the free energy difference in kJ/mol relative to the experimental structure along the transition path computed by Eq. (5). The dots in the inset and along the path correspond to the local free energy minima; structures visited before the highest barrier relax to the experimental structure (green dots), while after the barrier we found two new structures (orange and red dots).

line in Fig. 4) and follow the same procedure as described above.

In order to check the stability of the visited structures during Crystal-AFED, we performed 40 ps flexible-cell NPT simulations for structures found to be near the free energy minima (dots in Fig. 4) Consistent with the deep energetic minima for this structure, we found that all the local minima visited prior to the largest free energy barrier relaxed to the experimental structure. After passing over a barrier of +18 kJ/mol, structures relax in two different free energy minima (see Fig. 5, Table III and Table A.8), which correspond to two new crystal structures not obtained by the rigid molecule UPACK structure generation step (indicated by orange and red dots in Fig. 4).

The energy differences between the experimental structure and these new local free energy minima are +15.3 kJ/mol (orange dot) and +11.9 kJ/mol (red dot), while the computed free

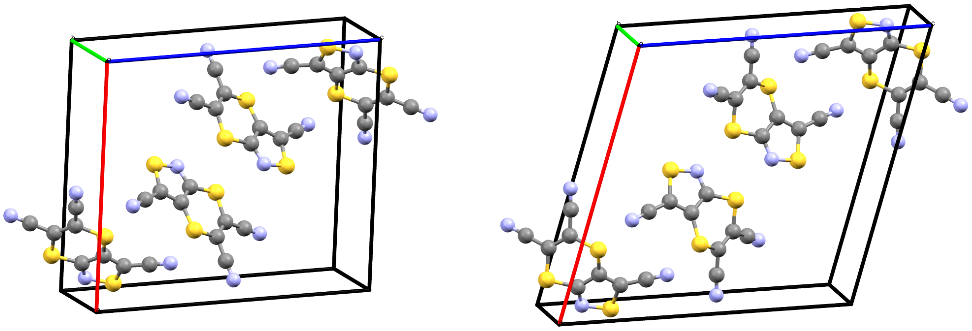

FIG. 5. Cell structures founded in Crystal-AFED simulation.

Space Group	a [Å]	b [Å]	c [Å]	β
P21/n	13.610	4.951	15.591	91.5
P21/n	15.566	4.201	16.080	106.2

TABLE III. Cell parameters for the two new structures. The first line corresponds to the orange dot, and the second line corresponds to the red dot.

energy differences are +11.5 kJ/mol and +10.8 kJ/mol, respectively. We point out that to compute these values, we also included the contribution during the NPTF simulation path, in which the cell relaxed slightly to reach the stable structures. While these high-energy new structures would not have been included in our submitted list, this example shows the ability of the Crystal-AFED method to explore possible crystal structures and compute the free energy between potential polymorphs.

[1] J. P. Perdew, K. Burke, and M. Ernzerhof, Phys. Rev. Lett. **77**, 3865 (1996).

[2] C. Adamo and V. Barone, J. Chem. Phys. **110**, 6158 (1999).

[3] S. Grimme, J. Antony, S. Ehrlich, and S. Krieg, J. Chem. Phys. **132**, 154104 (2010).

[4] F. Neese, "ORCA: An Ab Initio, DFT, and Semiempirical electronic structure package," with contributions from U. Becker, D. Ganyushin, A. Hansen, D. Liakos, C. Kollmar, S. Kossmann, T. Petrenko, C. Reimann, C. Riplinger, K. Sivalingam, B. Wezisla, and F. Wennmohs.

[5] R. A. Kendall, T. H. Dunning, Jr., and R. J. Harrison, J. Chem. Phys. **96**, 6796 (1992).

[6] B. Jeziorski, R. Moszyński, and K. Szalewicz, Chem. Rev. **94**, 1887 (1994).

[7] K. Szalewicz, Wiley Interdisc. Rev.–Comp. Mol. Sci. **2**, 254 (2012).

[8] A. J. Misquitta, B. Jeziorski, and K. Szalewicz, Phys. Rev. Lett. **91**, 033201 (2003).

[9] A. J. Misquitta, R. Podeszwa, B. Jeziorski, and K. Szalewicz, J. Chem. Phys. **123**, 214103 (2005).

[10] R. Bukowski, W. Cencek, P. Jankowski, M. Jeziorska, B. Jeziorski, S. A. Kucharski, V. F. Lotrich, A. J. Misquitta, R. Moszyński, K. Patkowski, R. Podeszwa, F. Rob, S. Rybak, K. Szalewicz, H. L. Williams, R. J. Wheatley, P. E. S. Wormer, and P. S. Żuchowski, "SAPT2012: An ab initio program for many-body symmetry-adapted perturbation theory calculations of intermolecular interaction energies," University of Delaware and University of Warsaw (2012).

[11] W. L. Jorgensen, D. S. Maxwell, and J. Tirado-Rives, J. Am. Chem. Soc. **118**, 11225 (1996).

[12] M. Metz, K. Piszczatowski, and K. Szalewicz, (2015), manuscript in preparation.

[13] K. Szalewicz, K. Patkowski, and B. Jeziorski, Intermolecular Forces and Clusters, Structure and Bonding **116**, 43 (2005).

[14] B.P. van Eijck and J. Kroon, J. Comp. Chem. **20**, 799 (1999).

[15] K. T. Tang and J. P. Toennies, J. Chem. Phys. **80**, 3726 (1984).

[16] R. Podeszwa, R. Bukowski, B. M. Rice, and K. Szalewicz, Phys. Chem. Chem. Phys. **9**, 5561 (2007)

.

[17] W. L. Jorgensen, T.B. Nguyen and J. Tirado-Rives, J. Comp. Chem. **14**, 195 (1993).

[18] A.W.S. da Silva and W. F. Vranken, BMC research notes **5**, 367 (2012).

[19] M. E. Tuckerman, D. A. Yarne, S. O. Samuelson, A. L. Hughes, and G. J. Martyna, Comp. Phys. Comm. **128**, 333 (2000).

[20] G. J. Martyna, M. E. Tuckerman, and M. L. Klein, J. Chem. Phys. **97**, 2635 (1992).

[21] G. J. Martyna, D. J. Tobias, and M. L. Klein, J. Chem. Phys. **101**, 4177 (1994).

[22] G. J. Martyna, M. E. Tuckerman, D. J. Tobias, and M. L. Klein, Mol. Phys. **87**, 1117 (1996).

[23] J. Alejandre, R. López-Rendón, A. L. Jochim, G. J. Martyna, and M. E. Tuckerman, J. Phys. A **39**, 5629 (2006).

[24] T. -Q. Yu, J. Alejandre, R. López-Rendón, G. J. Martyna, and M. E. Tuckerman, Chem. Phys. **370**, 294 (2010).

[25] M. E. Tuckerman, G. J. Martyna, and B. J. Berne, J. Chem. Phys. **97**, 1990 (1992).

[26] A. L. Spek, J. App. Cryst. **36**, 7-13 (2003).

[27] T.-Q. Yu and M. E. Tuckerman, Phys. Rev. Lett. **107**, 015701 (2011).

[28] L. Maragliano and E. Vanden-Eijnden, J. Chem. Phys. **128**, 184110 (2008).

[29] T.-Q. Yu, P.-Y. Chen, M. Chen, A. Samanta E. Vanden-Eijnden, and M. E. Tuckerman, J. Chem. Phys. **140**, 214109 (2014).

Appendix A: Force Fields parameters

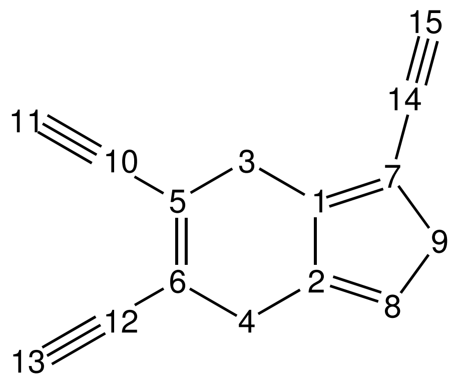

FIG. A.1. Atom numbering for force field specification

1	2	3	4	5	6	7	8	9
-0.2128	0.0721	0.1706	0.1566	-0.2732	-0.1279	0.3331	-0.2880	0.2222

10	11	12	13	14	15
0.5932	-0.4577	0.4410	-0.4017	0.0640	-0.2915

TABLE A.1. Atomic charges in atomic units from SAPT(DFT) fit

Atom #	C_{12} (kcal/mol Å^{-12})	C_6 (kcal/mol Å^{-6})
1	280439	280.22
2	280439	280.22
3	1001568	1000.78
4	1001568	1000.78
5	280439	280.22
6	280439	280.22
7	280439	280.22
8	236075	400.66
9	1001568	1000.78
10	838698	709.38
11	195997	365.07
12	838698	709.38
13	195997	365.07
14	838698	709.38
15	195997	365.07 power-series potential

TABLE A.2. Lennard-Jones parameters from OPLS-AA[11,17] for initial packing and MD intramolecular interactions

Atom	Number		k
1	7	14	1226.2
5	6	12	1226.2
6	5	10	1226.2
3	5	10	1226.2
4	6	12	1226.2
9	7	14	1226.2
5	10	11	2978.0
6	12	13	2978.0
7	14	15	2978.0

TABLE A.3. Cyano group bend parameters taken from OPLS-AA[11,17]

#1	#2	#3	#4	V0	V1	V2	V3
1	2	4	6	2.20	-32.06	2.20	12.80
1	3	5	6	2.20	-26.02	2.20	10.45
1	3	5	10	2.20	34.00	2.20	-13.54
2	1	3	5	2.20	-25.47	2.20	10.24
2	4	6	5	2.20	-27.80	2.20	11.14
2	4	6	12	2.20	36.14	2.20	-14.36
6	4	2	8	2.20	36.14	2.20	-14.36
7	1	3	5	2.20	41.41	2.20	-16.37

TABLE A.4. Modified intramolecular power-series torsional potential parameters for XXII in kcal/mol.

Atom #	β_i (Å^{-1})	α_i	C_i (kcal/mol Å^{-6})	δ_i (Å^{-1})
1	3.59	9.202	4.05	5.66
2	3.59	5.725	6.59	5.66
3	3.33	11.561	1727.96	5.61
4	2.91	11.186	5840.51	5.18
5	3.59	8.676	3.46	5.66
6	3.53	8.792	3.39	5.66
7	2.96	7.586	6.11	5.65
8	2.66	8.267	470.75	3.19
9	2.61	10.331	9087.07	2.90
10	3.58	9.012	4.91	5.67
11	2.42	8.473	4166.92	2.60
12	3.57	9.335	4.63	5.67
13	2.86	9.426	2596.94	2.65
14	2.73	7.598	33.19	5.67
15	3.20	10.343	1896.90	4.26

TABLE A.5. Intermolecular parameters for SAPT(DFT) potential `poly0`

Atom #	β_i (Å^{-1})	α_i	C (kcal/mol Å^{-6})	δ_i (Å^{-1})
1	3.58	13.223	4.05	5.66
2	3.58	11.618	6.59	5.66
3	2.68	10.938	1727.96	5.61
4	2.66	10.859	5840.51	5.18
5	3.58	12.960	3.46	5.66
6	3.43	11.906	3.39	5.66
7	2.86	9.409	6.11	5.65
8	2.90	9.070	470.75	3.19
9	2.23	9.096	9087.07	2.90
10	3.34	11.460	4.91	5.67
11	1.99	7.187	4166.92	2.60
12	3.59	11.452	4.63	5.67
13	2.15	7.786	2596.94	2.65
14	3.21	11.066	33.19	5.67
15	2.50	8.763	1896.90	4.26

TABLE A.6. Intermolecular parameters for SAPT(DFT) potential `poly1`

a	b	a_{ab} (Å^{-1})	a	b	a_{ab} (Å^{-1})	a	b	a_{ab} (Å^{-1})	a	b	a_{ab} (Å^{-1})
1	1	-0.31	8	3	-0.07	11	6	-0.139	13	13	-0.0310
2	1	-0.11	8	4	-0.01	11	7	-0.057	14	1	-0.2341
2	2	-0.13	8	5	-0.22	11	8	-0.057	14	2	0.0453
3	1	-0.13	8	6	-0.04	11	9	0.045	14	3	-0.1203
3	2	-0.21	8	7	-0.11	11	10	-0.091	14	4	-0.1608
3	3	-0.11	8	8	-0.02	11	11	0.020	14	5	-0.1331
4	1	-0.12	9	1	-0.14	12	1	-0.0091	14	6	-0.2191
4	2	-0.15	9	2	-0.16	12	2	0.0853	14	7	0.0647
4	3	-0.10	9	3	-0.07	12	3	-0.1692	14	8	-0.1742
4	4	-0.06	9	4	-0.02	12	4	-0.1402	14	9	-0.1270
5	1	0.01	9	5	-0.14	12	5	-0.1098	14	10	-0.1701
5	2	-0.05	9	6	-0.12	12	6	-0.1249	14	11	-0.2046
5	3	-0.20	9	7	-0.13	12	7	-0.3842	14	12	-0.0906
5	4	-0.15	9	8	-0.00	12	8	-0.1546	14	13	-0.1810
5	5	0.01	9	9	0.00	12	9	-0.1434	14	14	-0.1668
6	1	-0.11	10	1	-0.27	12	10	-0.2289	15	1	-0.1617
6	2	-0.47	10	2	-0.13	12	11	-0.1324	15	2	-0.2270
6	3	-0.10	10	3	-0.16	12	12	-0.1824	15	3	-0.1002
6	4	-0.13	10	4	-0.14	13	1	-0.2304	15	4	-0.0292
6	5	-0.14	10	5	-0.05	13	2	-0.1748	15	5	-0.1743
6	6	-0.10	10	6	-0.25	13	3	-0.0714	15	6	-0.1829
7	1	0.30	10	7	-0.02	13	4	-0.0432	15	7	-0.1912
7	2	-0.12	10	8	-0.06	13	5	-0.1948	15	8	-0.0682
7	3	-0.20	10	9	-0.18	13	6	-0.1779	15	9	0.0097
7	4	-0.15	10	10	-0.350	13	7	-0.0010	15	10	-0.1368
7	5	-0.17	11	1	-0.167	13	8	-0.0669	15	11	0.0418
7	6	0.02	11	2	-0.196	13	9	-0.0322	15	12	-0.1373
7	7	-0.80	11	3	-0.060	13	10	-0.1257	15	13	-0.0278
8	1	-0.09	11	4	-0.014	13	11	-0.0163	15	14	-0.1073
8	2	-0.28	11	5	-0.205	13	12	-0.1482	15	15	-0.0581

TABLE A.7. Polynomial parameter a_{ab} for SAPT(DFT) potential `poly1`

red dot				orange dot			
atom	x	y	z	atom	x	y	z
S	0.22824	0.60840	0.55126	S	0.22447	0.25135	0.55305
S	0.35229	0.24689	0.43552	S	0.32534	-0.19514	0.41927
S	0.47711	0.22536	0.69061	S	0.45709	-0.19124	0.64990
N	0.46150	0.16473	0.59223	N	0.43409	-0.27204	0.55622
N	0.01665	0.34791	0.41721	N	0.02536	0.39744	0.42024
N	0.17074	-0.12486	0.28048	N	0.14891	-0.15630	0.25743
N	0.33505	0.63025	0.81438	N	0.34621	0.31019	0.78997
C	0.33144	0.43325	0.59540	C	0.31947	0.04730	0.57819
C	0.38399	0.28457	0.54733	C	0.36091	-0.13670	0.52367
C	0.18679	0.37864	0.45923	C	0.17992	0.15101	0.45548
C	0.23711	0.23425	0.41184	C	0.21989	-0.02644	0.40271
C	0.37711	0.41991	0.68137	C	0.36853	0.03931	0.65500
C	0.35432	0.53653	0.75513	C	0.35651	0.18853	0.72980
C	0.09240	0.36353	0.43820	C	0.09394	0.28767	0.43683
C	0.20129	0.03835	0.33842	C	0.18068	-0.09942	0.32232

TABLE A.8. Fractional coordinates of the new structures founded using Crystal-AFED simulation
