# Supplementary material for: Report on the sixth blind test of organic crystal structure prediction methods
Source: Acta Crystallogr B Struct Sci Cryst Eng Mater. 2016 Aug 1;72(Pt 4):439–59. doi: 10.1107/S2052520616007447 (PMC4971545; doi:10.1107/S2052520616007447)
Supplement: Supplementary file 3 [file b-72-00439-sup3.zip › Group_21_Zhu_Oganov_Masunov_Post_Analysis.pdf]

# Supporting Information

Qiang Zhu[a], Artem R. Oganov[b,a,c,d], Artëm E. Masunov[e,f,g,h]

[a] *Department of Geosciences,Center for Materials by Design, and Institute for Advanced Computational Science, SUNY Stony Brook, New York 11794-2100, USA*

[b] *Skolkovo Institute of Science and Technology, Skolkovo Innovation Centers, Bldg. 3, Moscow Region, 143026, Russia*

[c] *Moscow Institute of Physics and Technology,9 Institutskiy lane, Dolgoprudny city, Moscow Region, 141700, Russia*

[d] *International Center for Materials Discovery, School of Materials Science and Enginering, Northwestern Polytechnical University, Xi'an, 710072, China*

[e] *NanoScienece Technology Center, University of Central Florida, 12424 Research Parkway, PAV400, Orlando, Florida, 32826, USA,*

[f] *Department of Chemistry, University of Central Florida, 4111 Libra Drive PSB225, Orlando, FL 32816, USA*

[g] *Department of Physics, University of Central Florida, 4111 Libra Drive PSB430, Orlando, FL 32816, USA*

[h] *Department of Condensed Matter Physics, National Research Nuclear University MEPhI, Kashirskoye shosse 31, Moscow, 115409, Russia*

## CSP METHODOLOGY

We firstly performed a systematic conformational analysis on the molecules, and then search for their crystal packings based on the evolutionary algorithms, as implemented in the USPEX code [1,2]. To start, the crystals structures were randomly generated with a given symmetry among all possible space groups (in this study, we constrained them to only triclinic, monoclinic, orthorhombic, and some tetragonal groups). Structures with lower energies are more likely to be selected as parents to generate new child structures by variation operators such as heredity and mutation. In contrast to the previous method [2], all the variations should only act on the asymmetric unit. Therefore, the initial symmetry was preserved (or increased) with the evolution.

For structural relaxations, we used a distributed multipole model to compute the relative lattice energies as implemented in DMACRYS [3]. To construct this model, the distributed multipole analysis of charge density obtained at the Moller-Plesset MP2 levels of theory using the 6-31G(d,p) basis set in Gaussian09 [4]. At the end of crystal structure search, the best 100 structures in energy ranking were selected to be reconsidered at higher level of theory (vdW-DF functional [5,6] in the VASP code [7]).

## HANDLING INTERNAL FLEXIBILITY

The internal degrees of freedom can be also taken into account in the USPEX code. However, the DMACRYS code is based on distributed multipole analysis of charge density on the rigid molecule. In order to find the molecular geometry for using with DMA model, a conformational analysis was performed before crystal packing search. The SCAN procedure from TINKER suite of programs [8] was used for the exhaustive conformational search. This conformational search employed custom polarizable molecule-specific AMOEBA force field parameters, obtained using POLTYPE script [9], which was modified to fit atomic multipoles to charge distribution at CAM-B3LYP theory level. The most important dihedral parameters were manually fit to relaxed potential surface scan with DFT. Conformations obtained were scored according to their DFT energies at CAM-B3LYP+D3 theory level.

Only the lower energy conformers were selected and treated as rigid molecules for the following crystal packing search with the USPEX code. This strategy was used for XXII, XXIII, XXV, and XXVI. Alternatively, one can also directly perform flexible molecular packing search if VASP is used as the local optimizer. Such strategy was applied to XXIV.

## CHOICES OF SPACE GROUPS AND Z'

For Z'=1 search (XXII-XXVI), all triclinic/monoclinic/orthorhombic/tetragonal space groups with less (or equal) than 8 symmetry operations have been considered.

For Z'=2 search (XXII-planar, XXVI), only $P1$, $P\text{-}1$, $P2_1$, $Cc$, $Pc$, $P2_1/c$, $P2_12_12_1$, $Pna2_1$, $Pca2_1$ wre allowed.

## SPECIAL NOTES

**XXII**: There are two possible conformations for XXII. The gas molecule is in flat configuration according to MP2 and pure DFT optimization. However, optimization with the range separated hybrid DFT functionals (wB97XD and CAM-B3LYP+D3) resulted in bending of six-member ring with 2 sulfur atoms, in agreement with majority cases in the database search. Therefore, we performed the structure search by starting from both the planar and bent conformations. The results are shown in **XXII_list1** (bent conformation, 50 structures) and **XXII_list2** (planar conformation, 30 structures).

**XXV**. Some structures in the submitted list underwent proton transfer from OH to N upon relaxation (OH··N → O··HN). The structure without proton transfer can be easily reconstructed by moving H back from N to O.

**XXIII and XXV:** The low-energy structures can be interpreted as the packing of stable dimers. Therefore, we performed additional crystal structure search by starting from those dimers.

# References

[1] Q. Zhu, A. R. Oganov, C. W. Glass, H. T. Stokes, *Acta. Cryst.* **2012,** B68, 215-226

[2] A. O. Lyakhov, A. R. Oganov, H. T. Stokes, Q. Zhu, *Comput. Phys. Comm.* **2013,** 184, 1172-1182

[3] S. L. Price, M. Leslie, G. W. A. Wlech, M. Habgood, L. S. Price, P. G. Karamertzanis, G. M. Day, *Phys. Chem. Chem. Phys.* **2010,** 12, 8478-8490

[4] M. J. Frisch, *et al. GAUSSIAN09,* Version A. 1. Gaussian Inc., **2009**, Wallingford, CT, USA

[5] M. Dion, H. Rydberg, E. Schroder, D. C. Langreth, B. I. Lundqvist, *Phys. Rev. Lett.* **2004,** 92, 246401

[6] J. Klimes, D. R. Bowler, A. Michaelides, *Phys. Rev. B,* **2011,** 83, 195131

[7] G. Kresse, J. Futhmuller, *Phys. Rev. B,* **1996,** 54, 11169-11186

[8] M. J. Dudek, K. Ramnarayan, J. W. Ponder *J Comput Chem* **1998**, 19, 548

[9] J. C. Wu, G. Chattree, P.Y Ren, *THEORETICAL CHEMISTRY ACCOUNTS,* **2012**, 131, 1138
