# Supplementary material for: Report on the sixth blind test of organic crystal structure prediction methods
Source: Acta Crystallogr B Struct Sci Cryst Eng Mater. 2016 Aug 1;72(Pt 4):439–59. doi: 10.1107/S2052520616007447 (PMC4971545; doi:10.1107/S2052520616007447)
Supplement: Supplementary file 3 [file b-72-00439-sup3.zip › Group_R22_Boese_Post_Analysis.pdf]

Methodology

The method employed involves six general steps:

(i) building a 3D molecular structure from the supplied 2D chemical diagrams;

(ii) searching for plausible molecular conformations;

(iii) searching for plausible crystal packing arrangements of the molecule;

(iv) intermediate ranking the generated crystal structures;

(v) comparison of the ranked crystal structures and elimination of the multiple structures (List: Hofmann-Kuleshova);

(vi) final ranking of the crystal structures with DFT in order of likelihood of formation (List Hofmann-Boese)

Method of generating the 3D molecular structures

The 2D chemical diagrams have been build up as 3D molecular structures with a 3D structure generator with Materials Studio of Accelrys.

Method of generating molecular conformations

The 3D molecular structures were analyzed by a systematic grid search for possible conformations with Materials Studio of Accelrys.

Generating trial crystal structures

The unit-cell parameters, the positions, the orientations, and the conformations of the molecules were generated randomly for any molecule with FlexCryst. During the generation of the crystal structure the space-group symmetry is used. The search has been for the nine most common space groups and have been performed separately for *Z'=1 and Z'=2.*

Intermediate ranking of crystal structures

The intermediate ranking of the crystal structures is still almost based on the calculated lattice energies with a force field of the structures generated by the crystal structure search with FlexCryst. The main idea of the force field is to derive the force field parameters from experimental crystal structures by data mining (Apostolakis 2001, Hofmann 2010). Recently the approach has been improved to get parameters for extended numbers of atom types, taking into account hybrid orbitals of atoms and charged species. For Molecule 24 we applied as additional criteria the density and the occurrence of synthons.

Elimination of manifold structures

The multiple structures have been eliminated with FlexCryst. The random generation of crystal structures causes the multiple generation of several crystal structures. Since several thousands of crystal structures are generated, it is necessary to have a quick method to compare the structures. For this purpose we use the simulated powder diagram (Hofmann 2005). The number of obtained twins give us the coverage of the energetic landscape (Hofmann 2009). After the elimination of the multiple structures the remaining structures are summarized in our list "Hofmann-Kuleshova" and have been used for further processing.

Final ranking

For the final ranking, we employed the Vienna ab initio Simulation Package (VASP) using density functional theory plus dispersion corrections. Based on our experience, PBE plus Tkachenko-Scheffler (TS) dispersion (Bucko 2013, Tkachenko 2009) yields the best error cancellation for organic solids (Boese 2013, Boese 2015/1), whereas for more ionic systems with hydrogen bonds, BLYP with Grimme (D3) dispersion (Grimme 2010) is giving a better performance (Boese 2015/2). Overall, we geometry-optimized the best structures of about six snapshots and looked for the thermodynamically most stable conformations. Here, hard pseudopotentials have been used with a 900 eV cutoff. The zero-point energies have been calculated using finite differences using the same methodology.

Acknowledgment

The computational results presented have been achieved in part using the Vienna Scientific Cluster (VSC).

References

Apostolakis 2001: Apostolakis, J., Hofmann, D. W. M., & Lengauer, T. (2001). Derivation of a scoring function for crystal structure prediction. *Acta crystallographica. Section A, Foundations of crystallography*, *57*(Pt 4), 442-450.

Hofmann 2010: Hofmann, D. W. M. (2010). Data Mining in Organic Crystallography. In *Data Mining in Crystallography* (pp. 37-58). Springer Berlin Heidelberg.

Hofmann 2005: Hofmann, D. W. M., & Kuleshova, L. (2005). New similarity index for crystal structure determination from X-ray powder diagrams. *Journal of applied crystallography*, *38*(6), 861-866.

Hofmann 2009: Hofmann, D. W. M., Kuleshova, L. N., Hofmann, F., & D'Aguanno, B. (2009). Cluster analysis and completeness of crystal structure generation. *Chemical Physics Letters*, *475*(1), 149-155.

Bucko 2013: Bucko, T., Lebegue, S., Hafner, J., Angyan, J. G., Tkachenko-Scheffler van der Waals correction with and without self-consistent screening applied to solids. *Phys. Rev. B, 87 (6)*, 064110.

Tkachenko 2009: Tkachenko, A., Scheffler, M., Accurate Molecular van der Waals Interactions from Ground-State Electron Density and Free-Atom Reference Data. *Phys. Rev. Lett., 102 (7)*, 073005.

Boese 2013: Boese, A.D., Kirchner, M., Echeverria, G. A., Boese, R., *ChemPhysChem, 14 (4)*, 799-804.

Boese 2015/1: Boese, A. D., Boese, R., *Cryst. Growth & Design, 15 (3)*, 1073-1081.

Grimme 2010: Grimme, S., Anthony, J., Ehrlich, S., Krieg, H., *J. Chem. Phys. 132 (15)*, 154104.

Boese 2015/2: Boese, A. D., *ChemPhysChem, 16 (5)*, 978-985.

Testing various Density Functionals with VASP, we get the following results with and without taking zero-point energies and free energies into account for the different polymorphs of XXIII.

Overall, we get a very large deviation of 7-10 kJ/mol for different functionals with different van der Waals coefficients. Free energies enlarge the range (with the exception of PBE-TS) and change the ordering of the polymorphs. Given that there are at least tens of other structures within this energy range, this is very discouraging. Within this energy range, we (Hofmann/Boese) obtained five more structures with our submission, and there are many, many more by Neumann and Price.

Table 1: Different relative energies in respect to polymorph c of molecule XIII. All energies are given in kJ/mol. The Zero-point energy contribution (ZPE) and full free energy contribution has been calculated with the PBE-TS functional.

Functional	Polymorph (XXIII)	Energy	Energy (Functional) + ZPE (PBE-TS)	Energy (Functional) + [ZPE + Free Energy contr.] (PBE-TS)
PBE-TS	a	1.7	2.4	1.6
	b	-2.8	-2.2	2.9
	c	0.0	0.0	0.0
	d	4.2	4.1	0.8
	e	3.0	3.3	2.2
	spread Δ	7.0	6.3	2.9
PBE-MBD	a	3.8	4.5	3.7
	b	0.8	1.3	6.5
	c	0.0	0.0	0.0
	d	4.5	4.4	1.2
	e	2.1	2.4	1.4
	spread Δ	4.5	4.5	6.5
optB88-vdW	a	5.5	6.2	5.4
	b	0.2	0.7	5.9
	c	0.0	0.0	0.0
	d	7.6	7.5	4.3
	e	3.8	4.1	3.0
	spread Δ	7.6	7.5	5.9
RPBE+D3	a	0.8	1.5	0.7
	b	0.4	0.9	6.1
	c	0.0	0.0	0.0
	d	1.2	1.1	-2.1
	e	1.3	1.6	0.5
	spread Δ	1.3	1.6	8.2

We have used four functionals with different dispersion coefficients, namely optB88vdW, PBE-TS, PBE-MBD and RPBE+D3.

In Table 1, we look at the sublimation energies differences with the different functionals. For the different forms of the polymorphs a-e, we computed all energies relative to the polymorph c, which is set to zero.

The (free energy) calculations on the last column, have, however, to be taken with grain of salt, as we were only able to calculate these at the Γ-point. Nevertheless, given the accuracy of these methods, for an estimate these values may be enough.

Without zero-point energies, optB88-vdW, PBE-MBD and RPBE+D3 predict c to be the most stable polymorph, whereas PBE-TS predict b to be more stable. Finally, when including free energies (computed with PBE-TS), all functionals except RPBE+D3 (with d being the most stable) predict c to be the polymorph lowest in energy. In general, the spread of energy of the five polymorphs is the lowest for RPBE+D3 with only 1.3 kJ/mol, and the largest for optB88-vdW with 7.6 kJ/mol. This is then changed to 2.9 (for PBE-TS) and 8.2 (RPBE+D3) kJ/mol, respectively, when including zero-point energies and temperature effects at the PBE-TS level of theory.

Within this predicted window of 8 kJ/mol, however, a lot of different polymorphs, including some reported by us, can be found. It still remains a challenge for crystal structure prediction to accurately foretell which ones of the many can be measured experimentally.
