# Supplementary material for: Report on the sixth blind test of organic crystal structure prediction methods
Source: Acta Crystallogr B Struct Sci Cryst Eng Mater. 2016 Aug 1;72(Pt 4):439–59. doi: 10.1107/S2052520616007447 (PMC4971545; doi:10.1107/S2052520616007447)
Supplement: Supplementary file 3 [file b-72-00439-sup3.zip › Group_R23_Brandenburg_Grimme_Post_Analysis.pdf]

The 6th crystal structure prediction blind test:
Contributions from Jan Gerit Brandenburg and Stefan Grimme

Jan Gerit Brandenburg[1, a)] and Stefan Grimme[1, b)]

Mulliken Center for Theoretical Chemistry, Institut für Physikalische und Theoretische Chemie, Rheinische Friedrich-Wilhelms Universität Bonn, Beringstraße 4, 53115 Bonn, Germany

(Dated: 31 December 2015)

[a)]Electronic mail: gerit.brandenburg@thch.uni-bonn.de

[b)]Electronic mail: grimme@thch.uni-bonn.de

Supporting Information

Start structures	Pages S3-S3
General methodology	Pages S3-S6
Methods for set A	Pages S6-S7
Methods for set B	Pages S7-S8
References	Pages S9-S9
Polymorph ranking	Pages S10-S29
Post analysis	Pages S30-S31

S1. START STRUCTURES

The group of Sarah L. Price (together with her coworkers Rebecca K. Hylton, Louise S. Price, Rui Guo, Rona E. Watson, and Luca Iuzzolino) kindly provided us for each target molecule the lowest 1000 crystal structures of their lattice energy predictions. The structures are not given in their final ranking, however, they have been given a generation tag, which we use for easier comparison in our submission.

It should be noted that with our current limited computational resources, the here presented approach is only possible because of the generation and ranking of structures in the Price group. Structures which have not been sufficiently low in the QM/MM treatment, are completely disregarded. A combination of different program packages, specifically the CrystalPredictor,[1] DMACRYST,[2] and CrystalOptimizer[3] codes, was used to successively refine the crystal polymorphs according to their lattice energy. The covered search space was restricted to $Z' = 1$ and the 59 space groups P1, P-1, P21, P21/c, P21212, P212121, Pna21, Pca21, Pbca, Pbcn, C2/c, Cc, C2, Pc, Cm, P21/m, C2/m, P2/c, C2221, Pmn21, Cmc21, Aba2, Fdd2, Iba2, Pnna, Pccn, Pbcm, Pnnm, Pmmn, Pnma, Fddd, Ibam, P41, P43, I-4, P4/n, P42/n, I4/m, I41/a, P41212, P43212, P-421c, I-42d, P31, P32, R3, P-3, R-3, P3121, P3221, R3c, R-3c, P61, P63, P63/m, P213, Pa-3, Cmcm, and Cmca.

For further details on the structure generation, intramolecular quantum chemistry method, and intermolecular force field potentials, we refer to the individual submission of the Price group.

S2. GENERAL METHODOLOGY

Our general strategy to cope with the large sampling space and at the same time yield sufficiently accurate polymorph rankings is a successive reduction of the polymorph space with increasingly accurate method as sketched in Figure S1. Here, it is mandatory that the energy interval for the most stable structures is determined by the largest possible error in the crystal energy method. Otherwise important polymorphs may be neglected. We start with 1000 structures that have to be treated. Therefore, semiempirical methods are applicable. We use two different approaches, namely the dispersion corrected density functional tight-binding DFTB3-D3[4,5] and the threefold corrected minimal basis set Hartree-Fock HF-3c.[6,7]

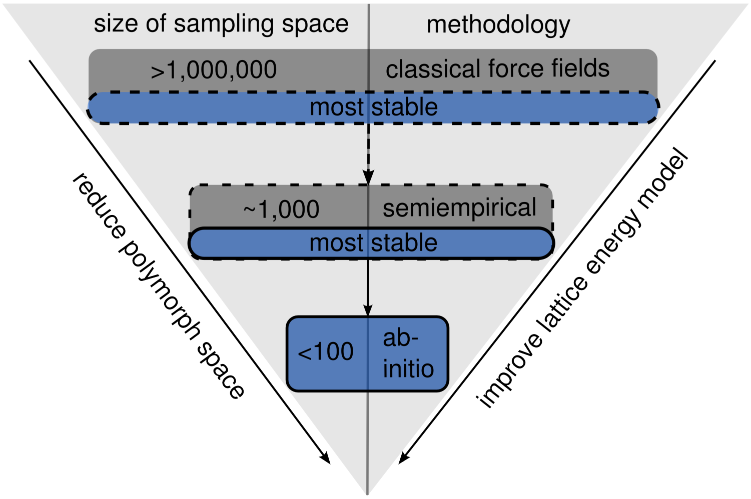

FIG. S1. A sketch of the reduction of the large polymorph sampling space. Millions of possible structures have to be reduced to a few plausible ones, which are then treated with high accuracy.

With DFTB3-D3, we could describe small organic crystals to very good accuracy with mean absolute deviations (MADs) from the references of about 2.5 kcal/mol.[5] Also various ice phases haven been successfully characterized with the tight-binding model.[8] However, we expect the description of more complicated binding situations to suffer from the very approximately treated Pauli exchange repulsion. Further, the Slater-Koster splines are only available for few (about 10) elements.[9] The DFTB3-D3 calculations are conducted with the dftb+ program.[10]

The accuracy of the HF-3c method is due to the exactly treated many-center integrals more robust as tested on the same benchmark sets given above.. Though it is substantially more expansive compare to the tight-binding scheme, is was possible to treat all 1000 structures in an energy single-point mode, i.e. HF-3c energy on the provided QM/MM structures. The HF-3c calculations are conducted with the CRYSTAL14 program suite.[11]

For higher accuracy, the single-particle basis set has to be converged more rigorously. In the projector-augmented plane wave (PAW[12]) approach the meta generalized gradient approximated (mGGA) density functional TPSS and the hybrid functional PBE0 are evaluated as implemented in the VASP package.[13] The estimated relative accuracy and relative computational cost are summarized in Table S1. At all levels, an accurate treatment of London dispersion interactions is mandatory. The three-body dispersion interaction is can be important as it alters the lattice energy by 2–7%. In the above listed methodologies, the three-body dispersion is included via a non-additive Axilrod-Teller-Muto type summation

TABLE S1. Classical and electronic structure methods for lattice energy calculations are classified. The typical accuracy for small organic solids is estimated as absolute deviation from reference lattice energies in kcal/mol and the relative cost is estimated as computational time needed for an energy and gradient calculation of a prototypical crystal.[5,7,14]

description	example	accuracy	rel. cost
semiempirical TB	DFTB3-D3	2.5	10^0
minimal basis HF	HF-3c^{atm}	2.0	10^3
GGA density functional	TPSS-D3^{atm}	1.5	10^4
hybrid density functional	PBE0-D3^{atm}	1.0	10^5

over atom triples within the D3 scheme.[15,16]

In addition to the purely electronic energy described above, zero-point and thermal corrections may be of importance. They can be calculated in a harmonic frequency approach and used additive to the above electronic energies. Because their calculation is substantially more expensive compared to the electronic energy, these contributions will be only considered at the semiempirical level. HF-3c frequencies are scales by 0.86 as suggested in the original methods publication.[6]

In a multi level approach, each of the contributions geometry, electronic energy, zero-point and thermal energy (ZPE) can be combined to a final free energy model. In the following, we use the notation

'electronic energy level' // 'geometry level'

and include ZPE ('frequency level') when ZPE contributions are considered. The geometries provided by the Price group will be abbreviated as force field (FF) structure, though the intramolecular structure is based on a quantum chemical calculation.

We decided to submit two sets of structures. The first set **A** is purely based on semiempirical methods, while the second set **B** uses *ab initio* methods for further refinements. However, the second set contains less than 100 structures, because we did not have the computational resources for a full (or more complete) treatment. The set **B** could be complemented with the semiempirical structure, but we prefer to keep the estimated energies and geometries consistent.

Due to very limited computational resources available for the crystal structure prediction

blind test and additionally only limited (human work) time spend on this project, target number XXV is only available within the set A. However, we want to stress that this is no principal limitation of our presented approach.

S3. METHODS FOR SET A

Step 1: DFTB3-D3//FF

We transform all 1000 supplied crystal structures into the needed CRYSTAL14 input format and calculate a single-point energy and gradient at the DFTB3-D3 level of theory. The structure is considered as reasonable when the calculated lattice energy is negative (net binding) and when the gradient is smaller than 100 a.u.

Step 2: HF-3c^{atm}//FF

On all structures which passed step 1 (about 900 to 1000), we compute a HF-3c^{atm} single-point energy. The polymorphs are ranked according to their calculated HF-3c^{atm}//FF electronic lattice energy.

Step 3: HF-3c^{atm}//HF-3c^{atm}

The lowest 100 to 150 structures are optimized (including call parameters) at the HF-3c^{atm} level with a fully consistent treatment of the space group symmetry. The polymorphs are ranked according to their calculated HF-3c^{atm}//HF-3c^{atm} electronic lattice energy.

The lowest 100 structures from step 3 are submitted as set A.

The computational effort to generate the set A of predicted crystal structures is summarized in Table S3. The timings are given in total CPU hours performed on our local computer cluster with Intel(R) Xeon(R) E5620 processors (about 4 years old). The overall time (excluding crashed or redundant computations) for the whole set sums up to about 51 000 CPU hours.

TABLE S2. Energy selection windows ΔE in kcal/mol for set A, calculations on target systems 22–26. Step 3 corresponds to the energy window of the submitted polymorphs.

	XXII		XXIII		XXIV		XXV		XXVI	
	ΔE	#pol	ΔE	#pol	ΔE	#pol	ΔE	#pol	ΔE	#pol
step 1	28.0	1000	51.6	1000	151.7	1000	53.2	1000	50.0	1000
step 2	22.7	894	40.1	940	185.8	925	53.3	718	40.2	882
step 3	15.3	200	7.4	212	7.1	152	5.8	147	7.5	92
submit	3.1	100	2.3	100	4.1	100	3.9	100	7.5	92

TABLE S3. Timings in CPU h for set A, calculations on target systems 22–26. The individual steps are given per polymorph, the sum includes the factor due to the number of treated systems.

	XXII	XXIII	XIV	XV	XVI
step 1	0.001	0.003	0.001	0.02	0.003
step 2	0.6	0.2	0.4	2	2.2
step 3	43	13	21	207	27.7
sum	7 030	2 071	3 509	32 736	6 099

S4. METHODS FOR SET B

Step 4: TPSS-D3^{atm}/600eV//HF-3c^{atm}

On all structures passing step 3, we calculate TPSS-D3^{atm}/600eV single point energies. The polymorphs are ranked according to their calculated TPSS-D3^{atm}/600eV//HF-3c^{atm} electronic lattice energy.

Step 5a: TPSS-D3^{atm}/800eV//TPSS-D3^{atm}/800eV

The lowest 10 to 30 structures are optimized (including cell parameter) at the TPSS-D3^{atm}/800eV level.

Step 5b: TPSS-D3^{atm}/800eV + ZPE(HF-3c^{atm})//TPSS-D3^{atm}/800eV

For the structures treated in step 5a, we calculate zero-point and thermodynamic corrections

based on HF-3c^{atm} harmonic frequencies. The polymorphs are ranked according to their calculated TPSS-D3^{atm}/800eV + ZPE(HF-3c^{atm})//TPSS-D3^{atm}/800eV free energy.

The 10 to 30 structures treated in step 5 are submitted as set B.

TABLE S4. Energy selection windows ΔE in kcal/mol for set B, calculations on target systems 22–26. Step 3 corresponds to the energy window of the submitted polymorphs.

	XXII		XXIII		XXIV		XXV		XXVI	
	ΔE	#pol	ΔE	#pol	ΔE	#pol	ΔE	#pol	ΔE	#pol
step 4	4.4	150	6.7	100	9.1	125	–	–	6.8	92
step 5a	1.4	19	1.5	29	2.4	19	–	–	4.5	11
step 5b	1.4	19	1.8	29	2.4	19	–	–	–	–
submit	1.4	19	1.8	29	2.4	19	–	–	4.5	11

The computational effort to generate the set B of predicted crystal structures is summarized in Table S5. The timings are given in total CPU hours performed on our local computer cluster with Intel(R) Xeon(R) E5620 processors (about 4 years old). The overall time (excluding crashed or redundant computations but including the generation of set A) for the whole set sums up to about 70 000 CPU hours.

TABLE S5. Timings in CPU h for set B calculations on target systems 22–26. The individual steps are given per polymorph, the sum includes the factor due to the number of treated systems and the sum of step 1-3.

	XXII	XXIII	XIV	XV	XVI
step 4	2	1	2	–	5
step 5a	80	88	37	–	222
step 5b	347	344	x.x	–	–
sum	13 665	8 661	–	34 824	10 135

REFERENCES

[1]M. Vasileiadis, A. V. Kazantsev, P. G. Karamertzanis, C. S. Adjiman, and C. C. Pantelides, Acta Cryst. B **68**, 677 (2012).

[2]S. L. Price, M. Leslie, G. W. A. Welch, M. Habgood, L. S. Price, P. G. Karamertzanis, and G. M. Day, Phys. Chem. Chem. Phys. **12**, 8478 (2010).

[3]A. V. Kazantsev, P. G. Karamertzanis, C. S. Adjiman, and C. C. Pantelides, *CrystalOptimizer. An efficient Algorithm for Lattice Energy Minimisation of Organic Crystal using Isolated-Molecule Quantum Mechanical Calculations*, Vol. 6 (Wiley, Weinheim, 2010).

[4]M. Elstner, Theor. Chem. Acc. **116**, 316 (2006).

[5]J. G. Brandenburg and S. Grimme, J. Phys. Chem. Lett. **5**, 1785 (2014).

[6]R. Sure and S. Grimme, J. Comput. Chem. **34**, 1672 (2013).

[7]J. G. Brandenburg and S. Grimme, Top Curr Chem **345**, 1 (2014).

[8]J. G. Brandenburg, T. Maas, and S. Grimme, J. Chem. Phys. **142**, 124104 (2015).

[9]M. Gaus, A. Goez, and M. Elstner, J. Chem. Theory Comput. **9**, 338 (2013).

[10]B. Aradi, B. Hourahine, and T. Frauenheim, J. Phys. Chem. A **111**, 5678 (2007).

[11]R. Dovesi, R. Orlando, A. Erba, C. M. Zicovich-Wilson, B. Civalleri, S. Casassa, L. Maschio, M. Ferrabone, M. De La Pierre, P. D'Arco, Y. Noël, M. Causà, M. Rérat, and B. Kirtman, Int. J. Quantum Chem. **114**, 1287 (2014).

[12]P. E. Blöchl, Phys. Rev. B **50**, 17953 (1994).

[13]G. Kresse and J. Furthmüller, J. Comp. Mat. Sci. **6**, 15 (1996).

[14]J. G. Brandenburg, M. Hochheim, T. Bredow, and S. Grimme, J. Phys. Chem. Lett. **5**, 4275 (2014).

[15]S. Grimme, J. Antony, S. Ehrlich, and H. Krieg, J. Chem. Phys. **132**, 154104 (2010).

[16]S. Grimme, S. Ehrlich, and L. Goerigk, J. Comput. Chem. **32**, 1456 (2011).

[17]S. Grimme, G. Brandenburg, C. Bannwarth, and A. Hansen, J. Chem. Phys. **143**, 054107 (2015).

S5. POLYMORPH RANKING

22_list1_brandenburg

Rank	Elat/kcal/mol	rho / g/cmˆ3	SG	tag
1	-42.9517	1.6363	R-3	A207
2	-33.2742	1.8243	P-1	A7362
3	-33.2127	1.9379	P-1	A51
4	-33.2065	1.93	P-1	A6444
5	-32.8425	1.9137	P21/n	A8321
6	-32.6283	1.8562	P21/c	A43
7	-32.5507	1.9214	P-1	A481
8	-32.49	1.8296	P21/c	A2056
9	-32.137	1.8519	P21/n	A108
10	-32.046	1.843	P21/n	A98
11	-31.9835	1.9085	P21/n	A25
12	-31.9457	1.833	P-1	A9649
13	-31.926	1.8311	P-1	A1515
14	-31.8046	1.8668	P21/c	A104
15	-31.7856	1.8622	Pbca	A505
16	-31.7821	1.8184	Pbcn	A786
17	-31.7122	1.8594	P21212	A259
18	-31.6682	1.8502	P21/c	A580
19	-31.6453	1.8475	P-1	A123
20	-31.5111	1.8612	P212121	A58
21	-31.4985	1.8808	P21/n	A102
22	-31.4864	1.8181	P21/n	A10
23	-31.4763	1.8585	P212121	A426
24	-31.4744	1.8411	P21/c	A467
25	-31.3967	1.8216	P-1	A389
26	-31.3456	1.8158	P21/c	A41
27	-31.312	1.8978	P1	A981
28	-31.3111	1.8746	Pn	A2004

29	-31.3023	1.8764	P21212	A5044
30	-31.2772	1.8462	P21/n	A1
31	-31.2637	1.8204	P21/c	A5945
32	-31.2346	1.8655	P212121	A1393
33	-31.2292	1.746	P212121	A1440
34	-31.1994	1.8457	P21/n	A5922
35	-31.1444	1.8898	P21/n	A1692
36	-31.122 1.8424	P21/n	A77	
37	-31.0454	1.8463	P21/c	A645
38	-31.0302	1.8577	Pbcn	A728
39	-31.0236	1.8825	P21/n	A464
40	-30.9945	1.8483	P21/n	A471
41	-30.9639	1.886	P-1	A292
42	-30.9631	1.8772	Ia	A3988
43	-30.9496	1.8456	P21	A31
44	-30.9027	1.7968	P21/n	A196
45	-30.8766	1.8723	Pna21	A3518
46	-30.8747	1.8797	P21	A2643
47	-30.8683	1.8621	P21/c	A83
48	-30.842 1.8562	P21/c	A3328	
49	-30.8411	1.7909	P21/c	A782
50	-30.8134	1.8009	Pbcn	A362
51	-30.7954	1.8809	C2/c	A206
52	-30.7876	1.8218	P21/n	A161
53	-30.7827	1.8323	C2/c	A723
54	-30.7781	1.8736	Pca21	A192
55	-30.7505	1.8675	P-1	A381
56	-30.7431	1.7502	C2/c	A4131
57	-30.7367	1.8437	P2/c	A2685
58	-30.7231	1.9204	P21	A3558
59	-30.7009	1.8497	P21	A1663

60	-30.6702	1.843	P21/n	A93
61	-30.653 1.88	Pna21	A224	
62	-30.6481	1.8569	P21/c	A205
63	-30.6376	1.8632	Pbca	A285
64	-30.5964	1.8514	Pna21	A232
65	-30.5841	1.857	Pna21	A2966
66	-30.5771	1.7703	P21/c	A519
67	-30.5765	1.8043	Pna21	A14
68	-30.5715	1.8641	P21/c	A182
69	-30.5693	1.8545	P-1	A1051
70	-30.5649	1.855	P21/n	A3994
71	-30.5628	1.8881	P21/c	A955
72	-30.5132	1.8528	Pbcn	A762
73	-30.5126	1.7831	P21/n	A36
74	-30.5098	1.8758	P21/c	A1227
75	-30.4851	1.7983	Pn	A3624
76	-30.4779	1.8654	P21/c	A1670
77	-30.4753	1.8471	Pccn	A656
78	-30.4597	1.8339	P-1	A164
79	-30.4393	1.7561	P-1	A754
80	-30.408 1.813	P212121 A878		
81	-30.3796	1.787	P212121 A84	
82	-30.3711	1.7751	P-1	A2047
83	-30.3704	1.7979	P212121 A21	
84	-30.3491	1.8376	P21/n	A128
85	-30.3464	1.8304	P21/n	A821
86	-30.339 1.8415	P21/c	A573	
87	-30.3319	1.8145	P21/c	A1249
88	-30.3172	1.7993	P2/c	A884
89	-30.3107	1.7702	Pna21	A8308
90	-30.3031	1.8424	Pna21	A1194

91	-30.3013	1.8321	P212121	A1652
92	-30.3012	1.8315	P212121	A651
93	-30.2973	1.7349	I2/a	A9709
94	-30.2965	1.8111	P21/c	A336
95	-30.23	1.8169	P21/c	A134
96	-30.2103	1.8525	P212121	A398
97	-30.1895	1.7802	P21/n	A298
98	-30.1579	1.8797	P21/c	A13
99	-30.1531	1.7304	P21/c	A56
100	-30.1361	1.79	P21/n	A668

22_list2_brandenburg

Rank	Elat/kcal/mol	rho / g/cm^3	SG	tag
1	-28.3509	1.7595	P21/c	A467
2	-28.1666	1.8157	P21/n	A1
3	-27.8983	1.7956	P21/c	A645
4	-27.8783	1.7546	P21/n	A10
5	-27.8314	1.7813	P-1	A1515
6	-27.75	1.746	P-1	A389
7	-27.6607	1.7012	P21	A32
8	-27.6488	1.7713	P21/n	A98
9	-27.6141	1.8134	Pna21	A224
10	-27.5345	1.7602	P21/c	A580
11	-27.4594	1.7599	P212121	A426
12	-27.4153	1.76	P212121	A58
13	-27.3276	1.7461	P21/n	A161
14	-27.324	1.7424	P21/c	A5945
15	-27.3188	1.6958	P21/c	A519
16	-27.2883	1.7818	P21/n	A108
17	-27.1874	1.7898	P212121	A1393
18	-27.1081	1.734	P21/c	A134

19	-26.911 1.7358	P21/c	A41	

23_list1_brandenburg

Rank	Elat/kcal/mol	rho / g/cmˆ3	SG	tag
1	-45.0465	1.5425	P-1	A2383
2	-44.9913	1.5037	P21/n	B116
3	-44.9877	1.5058	P-1	B29
4	-44.7955	1.5067	P-1	B609
5	-44.7111	1.5059	P-1	B45
6	-44.6492	1.5052	P-1	B68
7	-44.637 1.494	P21/n	A1189	
8	-44.4821	1.4944	P-1	A406
9	-44.3418	1.4947	P-1	A237
10	-44.3178	1.4946	P-1	A1276
11	-44.2367	1.4921	P-1	A797
12	-44.1758	1.4928	P-1	A333
13	-44.1676	1.4897	P-1	A284
14	-44.1661	1.4958	P-1	A2073
15	-44.1622	1.4946	P-1	A453
16	-44.1596	1.4934	P-1	A385
17	-44.1442	1.4956	P-1	A232
18	-44.091 1.5126	P-1	A323	
19	-44.0825	1.4938	P-1	A12658
20	-43.9989	1.5117	P-1	A4330
21	-43.9008	1.5212	P-1	A89
22	-43.8808	1.5216	P-1	A80
23	-43.8385	1.4861	P-1	A283
24	-43.7442	1.4868	P-1	A1697
25	-43.7321	1.4863	P-1	A913
26	-43.7284	1.4902	P-1	A3661
27	-43.6956	1.512	P-1	A157

28	-43.5359	1.4978	P-1	A228
29	-43.5206	1.4979	P-1	A257
30	-43.4881	1.4662	P21/c	A27
31	-43.486	1.4832	P-1	A19416
32	-43.479	1.4863	P-1	A70
33	-43.479	1.4742	P21/c	A929
34	-43.4752	1.4861	P-1	A44
35	-43.4686	1.5368	P-1	A2487
36	-43.4676	1.4857	P-1	A32
37	-43.464	1.486	P-1	A151
38	-43.4627	1.4829	P-1	A2412
39	-43.4539	1.4853	P-1	A4615
40	-43.4453	1.4725	P-1	A1543
41	-43.4431	1.4505	P21/c	B198
42	-43.443	1.4846	P-1	A11367
43	-43.3628	1.4771	P-1	A1698
44	-43.3625	1.4824	P-1	A14947
45	-43.3623	1.4846	P-1	A66
46	-43.3608	1.4838	P-1	A308
47	-43.3496	1.4835	P-1	A4
48	-43.3433	1.4724	P-1	A602
49	-43.3362	1.4716	P-1	A623
50	-43.3341	1.4989	P21/n	A428
51	-43.3311	1.4556	P21/c	A281
52	-43.3133	1.4824	P-1	A142
53	-43.3091	1.515	P-1	A871
54	-43.2985	1.472	P-1	A21933
55	-43.2974	1.4723	P-1	A452
56	-43.2806	1.4829	P-1	A375
57	-43.277	1.483	P-1	A5
58	-43.2399	1.4442	P-1	A3937

59	-43.2339	1.483	P-1	A8
60	-43.2048	1.4395	P-1	A48
61	-43.2035	1.4831	P-1	A394
62	-43.1842	1.4445	P-1	A53
63	-43.1738	1.4467	P21/c	A729
64	-43.1603	1.4716	P-1	A2677
65	-43.1515	1.4521	P-1	A238
66	-43.1484	1.4713	P-1	A2937
67	-43.1376	1.4358	P21/c	A1612
68	-43.1288	1.4529	P-1	A176
69	-43.0866	1.4604	P-1	C76
70	-43.0649	1.4405	P-1	A71
71	-43.06	1.4367	P-1	A523
72	-43.0419	1.4627	P-1	A7573
73	-43.013	1.461	P21/c	A6634
74	-43.0049	1.4598	P-1	B74
75	-42.9992	1.4691	P-1	A2964
76	-42.9831	1.4277	P-1	A209
77	-42.9741	1.4254	P-1	A16440
78	-42.9736	1.4413	P-1	A22
79	-42.9736	1.417	P-1	A2274
80	-42.9631	1.4255	P-1	B15
81	-42.9522	1.4587	P-1	B28
82	-42.951	1.4261	P-1	B7
83	-42.9402	1.44	P-1	A24995
84	-42.9393	1.4277	P-1	B17
85	-42.9378	1.4348	P-1	A165
86	-42.9378	1.5093	P-1	A312
87	-42.9222	1.4265	P-1	B10
88	-42.922	1.4401	P-1	A1149
89	-42.8655	1.4643	P-1	A856

90	-42.8637	1.4429	P-1	A1093
91	-42.8601	1.4429	P-1	A133
92	-42.8601	1.4412	P-1	A490
93	-42.8579	1.4445	P-1	A2181
94	-42.8537	1.437	P-1	A79
95	-42.8337	1.4336	P-1	A1631
96	-42.8119	1.4416	P-1	A1222
97	-42.8047	1.4399	P-1	A2786
98	-42.7569	1.4412	P-1	A2296
99	-42.749	1.4409	P-1	A924
100	-42.74	1.4324	P-1	A51

23_list2_brandenburg

Rank	Elat/kcal/mol	rho / g/cm^3	SG	tag
1	-44.0516	1.462	P-1	A32
2	-44.0508	1.4727	P-1	A4615
3	-44.0475	1.4728	P-1	A70
4	-44.0416	1.4723	P-1	A151
5	-44.0412	1.4622	P-1	A44
6	-44.0159	1.467	P-1	A11367
7	-43.9721	1.4588	P-1	A66
8	-43.9442	1.4722	P-1	A3661
9	-43.6499	1.439	P-1	A3937
10	-43.6139	1.436	P-1	A71
11	-43.5982	1.4317	P-1	A2786
12	-43.5925	1.4297	P-1	A53
13	-43.5714	1.4334	P-1	A48
14	-43.493	1.4333	P-1	A490
15	-43.2272	1.4761	P-1	A797
16	-43.198	1.4658	P-1	B68
17	-43.1943	1.4671	P-1	B45

18	-43.1749	1.4666	P-1	B29
19	-43.1635	1.4715	P-1	A232
20	-43.1585	1.4665	P-1	B609
21	-42.9343	1.4982	P-1	A80
22	-42.8121	1.4969	P-1	A89
23	-42.5382	1.4287	P-1	B20
24	-42.3016	1.4607	P-1	A283
25	-42.2724	1.4579	P-1	A284

24_list1_brandenburg

Rank	Elat/kcal/mol	rho / g/cm^3	SG	tag
1	-262.7058	1.3781	P212121	1331
2	-262.6353	1.4787	P21/n	1703
3	-262.3035	1.5388	P21/c	1522
4	-262.2272	1.5149	P21/c	1192
5	-262.1183	1.3987	P212121	863
6	-262.0923	1.3997	P212121	i445
7	-262.0891	1.6293	P21/c	i806
8	-262.0558	1.532	P21/c	1683
9	-262.0115	1.454	P212121	2189
10	-261.9622	1.6337	P21/c	144
11	-261.9284	1.6733	P21/c	721
12	-261.9043	1.5378	P21/c	1845
13	-261.8789	1.5783	P21/c	633
14	-261.8315	1.5329	P21/c	711
15	-261.8037	1.4664	P212121	2106
16	-261.7783	1.4885	P21/n	1585
17	-261.4158	1.4396	P21/n	2174
18	-261.3913	1.4926	P212121	2343
19	-261.2871	1.6631	P21/c	2239
20	-261.287	1.4393	P212121	1904

21	-261.1188	1.4679	P21/c	755
22	-261.0949	1.5106	P21/c	1547
23	-261.0797	1.5507	P21/c	159
24	-261.0714	1.5483	P21/c	1262
25	-261.054	1.6441	P21/n	464
26	-261.0533	1.657	P21/n	266
27	-261.0427	1.3972	P21/c	596
28	-261.0419	1.4808	P21/n	2031
29	-260.9302	1.4974	P212121	1897
30	-260.8363	1.6431	I2/c	475
31	-260.7614	1.5038	Pbca	1349
32	-260.5204	1.463	P21/c	2167
33	-260.5035	1.4472	P21/c	742
34	-260.4994	1.5176	P212121	261
35	-260.4391	1.5252	Pna21	1805
36	-260.3616	1.398	P21/n	336
37	-260.3436	1.4671	P2/n	1376
38	-260.2984	1.5353	P21/c	809
39	-260.1882	1.6608	P21/c	298
40	-260.0958	1.5158	P21/c	1509
41	-260.0533	1.7091	Pbca	813
42	-260.0526	1.4258	P21/n	1742
43	-260.0506	1.7192	Pbca	1084
44	-259.9759	1.4424	C2/c	1558
45	-259.9713	1.334	Pca21	1528
46	-259.956	1.463	P21/n	1748
47	-259.9543	1.4409	C2/c	i625
48	-259.874	1.4302	P212121	1036
49	-259.8276	1.417	P21/c	168
50	-259.7741	1.4899	P21/c	1616
51	-259.7562	1.4869	P21/c	98

52	-259.7524	1.6984	Pbca	112
53	-259.7487	1.5517	Pbca	123
54	-259.7268	1.4768	P21/c	1124
55	-259.7243	1.6949	P21/n	2365
56	-259.714	1.694	P21/c	559
57	-259.7078	1.464	P21/n	1022
58	-259.674	1.5091	Pbca	i455
59	-259.6359	1.3626	P212121	1928
60	-259.6355	1.7157	P21/n	1000
61	-259.6218	1.622	Pbca	1242
62	-259.5818	1.6827	Pbca	199
63	-259.5312	1.6147	Pbca	793
64	-259.5213	1.4377	Pbca	957
65	-259.4769	1.4762	Pna21	565
66	-259.4746	1.6341	P21/c	1301
67	-259.4581	1.5554	Pbca	139
68	-259.4481	1.5952	P21/c	1046
69	-259.4472	1.5949	P21/c	2141
70	-259.4053	1.5555	Pbca	369
71	-259.3919	1.4052	Pbcn	831
72	-259.3827	1.4009	Pbca	966
73	-259.3766	1.4743	Pbca	165
74	-259.3281	1.4177	P-1	645
75	-259.3131	1.5907	Fdd2	2201
76	-259.2657	1.6512	Pbca	233
77	-259.2405	1.4146	I2/c	200
78	-259.149	1.5384	P212121	169
79	-259.1417	1.538	P212121	517
80	-259.1057	1.3843	Pbcn	211
81	-259.1033	1.5496	P21/c	484
82	-259.0948	1.6685	Pbca	865

83	-259.0333	1.4048	Pbca	1451
84	-259.0181	1.4672	P21/c	1391
85	-258.9867	1.4785	P21/c	296
86	-258.9702	1.4133	Pbca	1396
87	-258.9386	1.4408	P21	54
88	-258.9342	1.6229	P21/n	2107
89	-258.923	1.6158	P2/c	2070
90	-258.9158	1.5865	Fdd2	1082
91	-258.9025	1.4058	Pbca	1888
92	-258.8989	1.4725	P21/c	74
93	-258.8718	1.3856	Pbca	39
94	-258.784	1.6674	Pbca	1214
95	-258.7603	1.4886	Pbca	304
96	-258.7298	1.4103	Cc	311
97	-258.7286	1.585	P212121	945
98	-258.7051	1.4022	P212121	710
99	-258.6841	1.7055	P21/n	254
100	-258.6431	1.4557	P21/c	512

24_list2_brandenburg

1	-449.7496	1.5522	P21/c	1192
2	-449.5828	1.5054	P21/n	2174
3	-449.2655	1.5161	P21/c	2167
4	-448.8815	1.5541	P21/c	1522
5	-448.7981	1.5488	P21/c	1547
6	-448.3962	1.4926	P21/c	1124
7	-448.3318	1.5687	Pna21	1805
8	-448.1363	1.4364	P212121	1928
9	-447.9554	1.5747	P21/c	1262
10	-447.9183	1.5728	P21/c	159
11	-447.9068	1.4392	P212121	1331

12	-447.8525	1.4818	P2/n	1376
13	-447.6988	1.4920	P21/c	1391
14	-447.5926	1.5353	P21/c	98
15	-447.4990	1.5305	P21/c	1683
16	-447.4022	1.5015	P212121	2106
17	-447.1551	1.5399	P21/c	1509
18	-447.0435	1.4633	Pbca	39
19	-447.0	1.4930	P212121	2189

25_list1_brandenburg

Rank	Elat/kcal/mol	rho / g/cm^3	SG	tag
1	-61.1044	1.4549	Pbca	A613
2	-60.8643	1.4889	P21/c	B1
3	-60.5578	1.4542	P21/c	A1954
4	-60.221	1.484	P-1	A1928
5	-60.1886	1.4699	P-1	A1477
6	-60.1165	1.4385	C2/c	A207
7	-59.8104	1.4438	Pbca	A214
8	-59.6795	1.4299	Pbca	A13426
9	-59.5962	1.4575	P2/c	A56
10	-59.4899	1.4357	I2/a	B18669
11	-59.4548	1.4543	P21/n	A118
12	-59.437	1.4481	P21/c	A10
13	-59.4255	1.4775	P21/n	A192
14	-59.364	1.4748	P-1	A12
15	-59.2928	1.4571	P-1	A8936
16	-59.0793	1.3937	P21/c	A1043
17	-59.0463	1.4231	Pbca	B13553
18	-58.9883	1.418	Pbca	A340
19	-58.934	1.4881	P-1	A4
20	-58.9337	1.4414	P-1	A6480

21	-58.8834	1.4349	Pbca	A775
22	-58.8725	1.4676	Pna21	A957
23	-58.803	1.4222	P-1	A4518
24	-58.7742	1.4179	P-1	A9908
25	-58.6337	1.401	Pna21	A1005
26	-58.6299	1.4607	P-1	A15092
27	-58.5911	1.4329	P21/c	A3308
28	-58.5322	1.4112	P21/c	A864
29	-58.5107	1.4143	P-1	A717
30	-58.5084	1.4201	P21/c	A334
31	-58.5026	1.4313	P-1	A4025
32	-58.4873	1.4632	P-1	A134
33	-58.3364	1.4383	P21/c	A3194
34	-58.3299	1.4314	P21/c	A1430
35	-58.3228	1.4195	P212121	A3019
36	-58.3146	1.4623	P-1	A173
37	-58.2851	1.4103	P21/c	A432
38	-58.269	1.2909	R-3	B10981
39	-58.2627	1.4711	P-1	A1238
40	-58.1951	1.4532	P21/c	A3025
41	-58.1916	1.4634	P-1	A14814
42	-58.1841	1.3955	P21/c	A8498
43	-58.1706	1.4298	P21/c	A2023
44	-58.0945	1.4275	P21/c	A877
45	-58.065	1.4622	P21	A539
46	-57.976	1.4522	C2/c	A74
47	-57.9673	1.4682	P21/c	A568
48	-57.955	1.3986	P-1	A3204
49	-57.9508	1.4334	P21/c	B1401
50	-57.9488	1.4423	P-1	A3095
51	-57.9087	1.4705	C2/c	A1167

52	-57.8936	1.4248	P21/n	A3268
53	-57.8839	1.4125	P212121	A1277
54	-57.8809	1.4919	P-1	A696
55	-57.8613	1.4328	Pbcn	A13
56	-57.847	1.468	P-1	A9520
57	-57.8453	1.4485	P21/n	A569
58	-57.8411	1.4095	P21/c	A1068
59	-57.8224	1.4484	P-1	A133
60	-57.8036	1.4239	P21/n	A381
61	-57.7986	1.4655	Pna21	A872
62	-57.7902	1.4054	P21/n	A540
63	-57.7895	1.4204	P21/n	A15548
64	-57.7566	1.4531	Pbca	A86
65	-57.7554	1.4641	P21/c	A508
66	-57.7384	1.398	P21/c	B1108
67	-57.7329	1.4018	P21/n	A672
68	-57.7146	1.4279	P-1	A811
69	-57.6927	1.4024	P21/c	A1369
70	-57.69	1.4415	P21/c	A282
71	-57.6822	1.4407	P21/n	A1565
72	-57.6556	1.4302	P21/c	B1207
73	-57.6306	1.4425	P21/n	A14
74	-57.6172	1.4433	C2/c	B10346
75	-57.6108	1.4177	P-1	A438
76	-57.5947	1.4124	P1	A974
77	-57.5775	1.4645	P21/c	A11431
78	-57.5584	1.467	P-1	A1395
79	-57.5541	1.4072	P212121	A5234
80	-57.5501	1.4359	P-1	A707
81	-57.5249	1.4436	P-1	A75
82	-57.5111	1.4063	P212121	A252

83	-57.494	1.4334	P212121	A232
84	-57.4895	1.4427	P21/c	A2944
85	-57.4825	1.4132	P-1	A71
86	-57.4821	1.4452	P21/c	A139
87	-57.4389	1.4135	C2/c	A520
88	-57.4334	1.4392	C2/c	A13589
89	-57.4192	1.4325	P212121	A104
90	-57.4046	1.4217	P212121	A558
91	-57.3995	1.4057	P21/c	A816
92	-57.3618	1.4397	P21/n	A3517
93	-57.3585	1.437	Pbcn	A3287
94	-57.3544	1.4107	Pbca	A970
95	-57.3391	1.4667	P21/c	A9267
96	-57.3328	1.4711	P-1	A959
97	-57.3026	1.391	P21/c	A1836
98	-57.3012	1.4161	P-1	A2613
99	-57.2759	1.4843	P21/c	A564
100	-57.2459	1.4503	P21/n	B11370

26_list1_brandenburg

1	-52.0195	1.4441	P21/n	314
2	-51.9901	1.4448	P21/n	229
3	-51.9082	1.4443	P21/n	593
4	-51.5434	1.4583	P21/n	361
5	-50.6398	1.4698	P21/n	421
6	-50.6224	1.4672	P21/n	675
7	-50.3282	1.4715	P21/c	239
8	-50.0407	1.5149	P21/c	43
9	-49.7366	1.5147	P21/n	2358
10	-49.5740	1.4690	P-1	337
11	-49.5487	1.4684	P21/c	508

12	-49.5482	1.4675	P21/c	497
13	-49.5471	1.4666	P-1	339
14	-49.4985	1.4643	P-1	338
15	-49.4967	1.4428	P21/n	579
16	-49.4931	1.4427	P21/n	526
17	-49.4817	1.4427	P21/n	379
18	-49.0254	1.4526	P-1	4800
19	-48.9598	1.4501	P21/c	2496
20	-48.9159	1.4493	P21/c	2877
21	-48.8952	1.4056	C2/c	294
22	-48.8697	1.4303	C2/c	280
23	-48.6552	1.2712	P21/c	1480
24	-48.6465	1.4529	P21/n	1275
25	-48.6273	1.4376	P-1	3215
26	-48.6142	1.4376	P-1	3702
27	-48.4331	1.4313	P-1	2390
28	-48.2357	1.4273	P-1	2623
29	-48.1633	1.4540	P-1	166
30	-48.0865	1.4445	P21/c	565
31	-48.0741	1.4408	P21/n	473
32	-48.0489	1.4413	P21/n	615
33	-48.0445	1.4447	P21/c	1249
34	-48.0133	1.4546	P-1	2231
35	-48.0025	1.4521	P-1	2230
36	-47.9917	1.4549	P-1	168
37	-47.9642	1.4010	Pbca	5126
38	-47.9636	1.4237	P21/n	5110
39	-47.9622	1.4234	P21/n	4122
40	-47.9568	1.4550	P-1	167
41	-47.9534	1.4543	P-1	165
42	-47.8489	1.4268	P21/n	3209

43	-47.8396	1.3117	P21/c	2225
44	-47.8299	1.4009	Pbca	3306
45	-47.8235	1.4351	P-1	180
46	-47.7914	1.4919	P21/n	83
47	-47.7848	1.4899	P21/n	138
48	-47.7645	1.4298	P-1	2477
49	-47.7387	1.4353	P-1	179
50	-47.6457	1.4810	P21/n	259
51	-47.4569	1.4539	P-1	955
52	-47.4014	1.4349	P-1	225
53	-47.3761	1.4815	C2/c	551
54	-47.2574	1.4337	P21/c	709
55	-47.1108	1.5053	I2/c	5245
56	-47.0686	1.4374	P21/c	599
57	-47.0427	1.4403	P-1	2137
58	-46.9695	1.4357	P21/n	960
59	-46.9509	1.4285	P-1	1739
60	-46.9450	1.4360	P-1	2002
61	-46.9296	1.4413	P-1	1640
62	-46.9028	1.4247	P-1	4266
63	-46.8818	1.4362	P-1	2003
64	-46.8740	1.4286	P-1	4816
65	-46.8512	1.4259	P-1	4817
66	-46.7533	1.4620	P-1	5201
67	-46.7434	1.4585	P-1	4636
68	-46.7418	1.4879	C2/c	245
69	-46.7114	1.4321	P21/n	1377
70	-46.6620	1.4394	P21/n	871
71	-46.5350	1.3877	C2/c	595
72	-46.3185	1.4505	P21/c	3141
73	-46.2128	1.4346	P21/n	400

74	-46.1858	1.4253	P21/n	576
75	-46.1450	1.4384	P-1	1346
76	-45.9667	1.4238	P212121	810
77	-45.9344	1.4088	R-3	1352
78	-45.8929	1.4243	P212121	1244
79	-45.7444	1.4129	P21/n	2971
80	-45.7291	1.2891	P21/n	1881
81	-45.6806	1.4197	C2/c	686
82	-45.6468	1.4198	C2/c	1083
83	-45.5611	1.4363	C2/c	2004
84	-45.5282	1.4183	P21/c	1920
85	-45.3266	1.4392	P21/n	545
86	-45.2655	1.4404	P21/n	445
87	-45.0623	1.3954	P21/n	1242
88	-45.0324	1.3862	Pbca	1786
89	-44.9597	1.4063	P-1	3420
90	-44.9258	1.4004	P-1	3801
91	-44.6064	1.4160	P21/c	1050
92	-44.4985	1.3945	P21/c	5257

26_list2_brandenburg

1	-48.0214	1.4328	P21/n	361
2	-47.7995	1.4409	P21/n	421
3	-47.7572	1.4425	P21/n	675
4	-47.7122	1.4271	P21/n	229
5	-47.6887	1.4292	P21/n	593
6	-47.6839	1.4281	P21/n	314
7	-47.0487	1.4436	P21/c	239
8	-46.5393	1.4357	P-1	166
9	-46.4312	1.4358	P-1	165
10	-45.5347	1.4589	C2/c	245

11	-43.5514	1.3741	P21/n	1881

S6. POST ANALYSIS

After the experimental structures have been released, we performed some post-analysis. This is helpful to identify the sources, where experimental polymorphs have been lost. Furthermore it can be used to judge the accuracy of the lattice energy models by comparing the energy (and density) of the experimental polymorphs with the computed rank and structure. As we have submitted polymorph lists without removing the redundant polymorphs, we have used in the post analysis a standard mercury structure match to remove those structures. Because each group has generated its own polymorph list, these analysis has to be compared carefully.

TABLE S6. Experimental polymorphs are included in the predicted polymorph landscape. The absolute rank together with the energetic distance to the most stable polymorph and the deviation of the computed mass density with the measured one are given.

	HF-3c			TPSS-D3			TPSS-D3(ΔG)		
	rank	ΔE **	$\Delta\rho$ *	rank	ΔE **	$\Delta\rho$	rank	ΔE **	$\Delta\rho$ *
XXII	8	10.6	1.4%	1	0	1.0%	1	0	-1.0%
XXIIIA	104	3.4	0.2%	4	0.8	4.3%	7	1.0	-0.7%
**B**	10	1.4	2.7%	1	0	6.2%	**1**	**0**	**1.4%**
C	6	0.7	0.8%	15	1.4	4.9%	13	0.9	-1.3%
D	108	3.2	0.7%	14	1.3	5.1%	11	0.7	-0.2%
E	40	2.0	3.1%	10	0.9	5.8%	9	0.4	0.6%
XXIV	1	0	-1.9%	1	0	3.8%	1	0	0.2%
**XXV**	2	0.2	1.2%	2	0.2	1.5%	**2**	**0.0**	**-3.3%**
XXVI	18	2.8	1.6%	1	0	4.3%	1	0	-0.3%
|mean|	55	2.7	1.8%	5.4	0.5	4.1%	5.1	0.3	1.0%

* including quasi-anharmonic ZPE and thermal corrections at DFTB3-D3 level

** difference to lowest polymorph in kcal/mol

In Table S6 we give the new ranking of the methodologies, which are applied as described above. Additionally, the quasi-anharmonic effect of ZPE and thermal corrections to the mass density are estimated at the DFTB3-D3 level. This relative effect is used to correct the computed mass densities accordingly.

The final lattice energy model at the TPSS-D3 level provides excellent rankings. Only some polymorphs of target XXIII are by about 1 kcal/mol less stable compared to the ther-

modynamically most stable one (XXIIIB). The effect of ZPE and thermal energies on the ranking seem to be small, but the results overall improve. Apart from the polymorphs of compound XXIII, HF-3c provides very reasonable rankings considering the computational speed up. Interestingly, the ZPE and thermal effect on the geometry (as analyzed by the mass density) is significant. The corrected TPSS-D3 mass densities agree with a mean absolute error of 1.0% with the experimental measurements. The (corrected) HF-3c mass densities deviate slightly more from the experiment with mean absolute deviation of 1.4%. Both systematic shifts are qualitaticely with the known trends from benchmark calculations.[8,17] TPSS-D3 computes slightly too large noncovalent distances (which corresponds to underestimated mass densities), while HF-3c on average underestimates noncovalent distances leading to too dens crystals.

TABLE S7. Analysis at which prediction stage the experimental polymorph was 'lost'.

XXII	positive E_{lat} on FF geometry
XXIIIA	positive E_{lat} on FF geometry
C	not in FF search space
D	not in DFT(30) space
E	not in FF search space
XXIV	not in FF(1000) space
XXVI	not in HF-3c(100) space

The analysis in Table S7 shows that the critical points are the transitions from one lattice energy model to another. Especially the FF potential energy surface seems to deviate significantly from the HF-3c and TPSS-D3 one. The force field potentials for the heavier elements (S, CL) can probably be improved and more structures can be treated at the full DFT level.
