# Supplementary material for: Report on the sixth blind test of organic crystal structure prediction methods
Source: Acta Crystallogr B Struct Sci Cryst Eng Mater. 2016 Aug 1;72(Pt 4):439–59. doi: 10.1107/S2052520616007447 (PMC4971545; doi:10.1107/S2052520616007447)
Supplement: Supplementary file 3 [file b-72-00439-sup3.zip › Group_R24_Szalewicz_Post_Analysis.pdf]

Supplementary information for crystal structure predictions of cocrystal XXIV

Michael P. Metz,[1] Rui Guo,[2] Sarah L. Price,[2] and Krzysztof Szalewicz[1]

[1]*Department of Physics and Astronomy, University of Delaware, Newark, Delaware 19716, USA*

[2]*Department of Chemistry, University College London, 20 Gordon Street, London WC1H 0AJ, UK*

(Dated: July 7, 2016)

System XXIV clearly represented a major challenge due to strong many-body polarization effects present. Such effects could have been included in potentials developed by the methods described below, but the crystal structure prediction (CSP) programs could not take a polarizable model. Thus, the polarization effects were fully accounted for at the two-body but not at the three- and higher-body levels. Moreover, this entry was only reranking of the structures obtained in the group 18 entry with a set of six dimer potentials fitted to *ab initio* interaction energies. The potentials were of a rather simple form, again due to CSP software restrictions.

I. METHODOLOGY

The intermolecular potentials were constructed using symmetry adapted perturbation theory (SAPT)[1,2] based on Kohn-Sham density-functional theory (DFT) description of monomers, SAPT(DFT)[3,4], implemented in the SAPT2012[5] package, using ORCA[6] for monomer DFT. Six potentials were constructed, one for each pair of monomers in the three-component system. The geometry of the cation was obtained by unconstrained geometry optimization of the cation-chloride pair, with PBE0[7] and the aug-cc-pVTZ basis[8] using ORCA. Details of the six potentials are given in Table I.

The potentials were constructed for use with the DMACRYS program[9] and have the functional form

$$V = \sum_{a\in A}\sum_{b\in B} u_{ab}(r_{ab}) \tag{1}$$

where a and b are atoms in monomers A and B, respectively, and r_{ab} is the distance between them. The isotropic atom-atom function u_{ab} has the form

$$u_{ab}(r_{ab}) = e^{\alpha_{ab}-\beta_{ab}r_{ab}} + \frac{q_a q_b}{r_{ab}} - \frac{\sqrt{C_a C_b}}{(r_{ab})^6} \tag{2}$$

where α_{ab}, β_{ab}, q_i, and C_i are optimized free parameters.

Each potential was fit using an initial data set of 800 configurations for each dimer. For three out of the six dimers with only one or none monomers charged, these configurations were generated using a Monte Carlo (MC) type importance sampling algorithm with an OPLS-AA[10] guiding potential. For the cation, we extended the OPLS parametrization to the case of a charged systems by multiplying all the partial charges of the same sign as the overall charge by the same factor larger than 1, and the opposite sign partial charges by the inverse of this factor to get the appropriate overall charge. The chloride ion is parametrized in OPLS, so no modifications were necessary. For these systems, a large grid of dimer configurations was first randomly generated, and then each configuration was accepted with probability proportional to $\exp\left(-\frac{E_{\mathrm{guide}}-E_{\mathrm{min}}}{E_0}\right)$ or rejected, where E_{guide} is the interaction energy given by the guiding potential, E_{min} is the lowest of such values, and $E_0 = 12$ kcal/mol. We have chosen the same value of E_0 for all systems, which means that a given energetic interval above E_{min} is sampled with the same probability for each system. Another possible choice is to make E_0 proportional to $|E_{\mathrm{min}}|$, which results in a more even sampling of the whole potential well for each system, but we have not tried it. For the potentials involving two charges of the same sign, this importance sampling does not work since there are no minima on the surface. Therefore, we instead used MC without importance sampling (with the initial large grid generated in the region of intermonomer

separations large enough that the closest-contact atom pairs are reasonably well separated). The method with importance sampling should have worked for the cation-chloride dimer, but due to the very deep minimum we were not able to tune it properly and we eventually used MC without importance sampling also for this system. The initial set of 800 configurations for each dimer were iteratively augmented with additional grid points until the total set size listed in Table I was reached. The details of this iterative procedure are described in Ref. 11. SAPT(DFT) interaction energies with δ_{HF} correction[12] were computed at each of these dimer configurations using the aug-cc-pVTZ basis and the PBE functional for DFT calculations. The choice of 800 initial dimer configurations in all cases was due to the default setting of the automatic potential generation software[11], which was designed for large dimers. This choice results in unnecessarily many calculated points for the smaller systems, but since calculations for such systems are very fast relative to the calculations for large ones, this is not an issue.

Parameterization was performed using a procedure similar to that used in an earlier work on the cyclotrimethylene trinitramine (RDX) dimer, described in Ref. 13. Such parameterization is divided broadly into asymptotic and short-range fitting stages. In the asymptotic fitting stage, the multipole moments and static and dynamic polarizabilities of monomers are used to construct multipole expansions about the monomer's center of mass. These expansions are then used to compute electrostatic, induction, and dispersion interaction energies on a long-range grid of 10,000 dimer configurations, with intermonomer closest-contact atoms separated by at least 15 Å. The C_i and q_i parameters are then optimized using this grid of asymptotic interaction energies. This stage is described in detail in Ref. 13.

In the short-range fitting stage, all remaining free parameters are optimized using the set of SAPT(DFT) total interaction energies. The α_{ab} and β_{ab} parameters are optimized by minimizing the least-square functional via a nonlinear search using an implementation of Powell's method. All previously optimized long-range parameters are kept fixed at this stage to preserve asymptotic correctness of the potential. This final optimization is performed in multiple stages, and under a set of constraints designed to produce physically meaningful parameter values. The procedure is described in detail in Ref. 11.

The RMSEs listed in Table I may appear to be large compared to typical RMSEs of the fits to SAPT interaction energies which are of the order of a couple tenths of kcal/mol. There are couple reasons for it. First, due to restrictions imposed by the DMACRYS codes, the potential form of Eq. (2) is simpler than typically used in the fits of SAPT potentials. In particular, to charge-neutral systems the leading power of the asymptotic is $1/R^4$ and such power could not be used due to DMACRYS restrictions. Second, for the three charged-charged dimers, the interaction energies are very large at intermonomer closet-contact atom-atom separations of the order of the sum of van der Waals (vdW) radii. For example, the cation-chloride interaction energy at the dimer minimum is -131 kcal/mol, at least ten times larger than typical interaction energies of neutral systems in the vdW minimum region. In additions, for dimers of same charge monomers, the RMSE has to be calculated for all grid points, rather than those with negative interaction energies only. Such sets include always some points at very short separations with huge interaction energies which have large absolute errors. Thus, the RMSEs for charged-charged dimers about ten times larger than usual are to be expected. For the cation-water dimer, the RMSE of 1 kcal/mol amounts to about 5% of interaction energy at the minimum, which is a reasonable relative accuracy for our very simple form of the potential. For water-chloride and water-water systems the RMSEs are of the order of 10% of the interaction energies at the vdW minima. Here

Dimer	Number of points	RMSE	Minimum Energy
cation-cation	920	3.75	N/A
cation-water	1087	1.05	-20.7
cation-chloride	1226	6.90	-131.3
water-water	996	0.55	-5.14
water-chloride	863	1.59	-14.8
chloride-chloride	800	4.04	N/A

TABLE I. Details of interaction potentials: number of points, root-mean square errors (RMSEs, in kcal/mol), and interaction energies at global minima (in kcal/mol). RMSEs are given for fitting points with negative interaction energy, except for cation-cation and chloride-chloride where all points were used.

the reason for these large errors is that for such small monomers, the restriction of the potential to only atom-atom interactions does not allow accurate enough description of the anisotropy of the potentials. For such systems one has to use off-atomic sites if only isotropic pair potentials are used. For larger monomers, since any rigid-monomer potential is always only six-dimensional and the number of sites becomes large, the description of the dimer anisotropy becomes adequate.

The fitted potentials thus generated for system XXIV dimers were applied to 1580 hypothetical structures generated by the UCL team in a separate submission (see the submission number 18). The cation and water molecules in the structures were kept "as is", i.e., the monomer geometries were the same as in the submission 18 despite the fact that somewhat different geometries were used in the development of SAPT(DFT) potentials. We tried to reoptimize these structures using DMACRYS, but the reoptimizations failed due to "holes" in the potential surface, i.e., regions at short separations where the energy becomes unphysically low due to the competition between the exponential and $1/(r_{ab})^6$ terms. The removal of such holes is a part of the potential development, but there was not enough time to do it thoroughly enough before the submission deadline. This issue resulted in H_2O and/or chloride ions merging into the cation during the optimization, even though the initial lattice energies were reasonable. Therefore, we applied DMACRYS only to compute single-point lattice energies for the input structures and used the results to rerank the structures. The 100 crystal structures with the lowest intermonomer lattice energy form the list of this submission. Since the experimental structure was not on the list of the 1580 structures, this reranking could not succeed.

II. COMPUTER RESOURCES

A SAPT(DFT) calculation for the cation-cation system, including the $\delta_{\rm HF}$ correction points, took about 9 wall time hours per grid point on one 2.5 GHz Intel Ivy Bridge core. The cation-H_2O and cation-Cl systems took about 1.25 hours per point. The entire set of dimer interaction energies took in total about 15000 CPU hours. Other aspects of potential generation were negligible in comparison.

ACKNOWLEDGMENTS

RG and SLP would like to thank Costas Pantelides, Claire Adjiman, and Isaac Sugden of Imperial College for their support of the use of CrystalPredictor. The potential generation work at the University of Delaware was supported by the Army Research Office under Grant W911NF-13-1-0387 and by the National Science Foundation Grant CHE-1152899.

---

[1] B. Jeziorski, R. Moszyński, and K. Szalewicz, Chem. Rev. **94**, 1887 (1994).

[2] K. Szalewicz, Wiley Interdisc. Rev.–Comp. Mol. Sci. **2**, 254 (2012).

[3] A. J. Misquitta, B. Jeziorski, and K. Szalewicz, Phys. Rev. Lett. **91**, 033201 (2003).

[4] A. J. Misquitta, R. Podeszwa, B. Jeziorski, and K. Szalewicz, J. Chem. Phys. **123**, 214103 (2005).

[5] R. Bukowski, W. Cencek, P. Jankowski, M. Jeziorska, B. Jeziorski, S. A. Kucharski, V. F. Lotrich, A. J. Misquitta, R. Moszyński, K. Patkowski, R. Podeszwa, F. Rob, S. Rybak, K. Szalewicz, H. L. Williams, R. J. Wheatley, P. E. S. Wormer, and P. S. Żuchowski, "SAPT2012: An *ab initio* program for many-body symmetry-adapted perturbation theory calculations of intermolecular interaction energies," University of Delaware and University of Warsaw (2012).

[6] F. Neese, "*ORCA: An Ab Initio, DFT, and Semiempirical electronic structure package*," with contributions from U. Becker, D. Ganyushin, A. Hansen, D. Liakos, C. Kollmar, S. Kossmann, T. Petrenko, C. Reimann, C. Riplinger, K. Sivalingam, B. Wezisla, and F. Wennmohs.

[7] J. P. Perdew, K. Burke, and M. Ernzerhof, Phys. Rev. Lett. **77**, 3865 (1996).

[8] R. A. Kendall, T. H. Dunning, Jr., and R. J. Harrison, J. Chem. Phys. **96**, 6796 (1992).

[9] S. L. Price, M. Leslie, G. W. A. Welch, M. Habgood, L. S. Price, P. G. Karamertzanis, and G. M. Day, Phys. Chem. Chem. Phys. **12**, 8478 (2010).

[10] W. L. Jorgensen, D. S. Maxwell, and J. Tirado-Rives, J. Am. Chem. Soc. **118**, 11225 (1996).

[11] M. P. Metz, K. Piszczatowski, and K. Szalewicz, (2015), manuscript in preparation.

[12] K. Szalewicz, K. Patkowski, and B. Jeziorski, *Intermolecular Forces and Clusters*, Structure and Bonding **116**, 43 (2005).

[13] R. Podeszwa, R. Bukowski, B. M. Rice, and K. Szalewicz, Phys. Chem. Chem. Phys. **9**, 5561 (2007).
