# Supplementary material for: Report on the sixth blind test of organic crystal structure prediction methods
Source: Acta Crystallogr B Struct Sci Cryst Eng Mater. 2016 Aug 1;72(Pt 4):439–59. doi: 10.1107/S2052520616007447 (PMC4971545; doi:10.1107/S2052520616007447)
Supplement: Supplementary file 3 [file b-72-00439-sup3.zip › Group_R25_Tkatchenko_Post_Analysis.pdf]

6th Crystal Structure Prediction Blind Test

Johannes Hoja,[1] Hsin-Yu Ko,[2] Roberto Car,[2] Robert A. DiStasio Jr.,[2,3] and Alexandre Tkatchenko[1,*]

[1] *Fritz-Haber-Institut der Max-Planck-Gesellschaft, Faradayweg 4-6, 14195 Berlin, Germany*
[2] *Department of Chemistry, Princeton University, Princeton, NJ 08544, USA*
[3] *Department of Chemistry and Chemical Biology, Cornell University, Ithaca, NY 14853, USA*

GENERAL METHODOLOGY

In this work, we used density-functional theory (DFT) in conjunction with an explicit many-body dispersion (MBD) model for the long-range van der Waals (vdW) interactions to investigate the structures and energetics of the polymorphs pertaining to four systems (XXII, XXIII, XXIV, XXV) in the 6th crystal structure prediction (CSP) blind test. To utilize the DFT+MBD methodology in CSP, we employed a hierarchical procedure which is explained in detail below.

First, we obtained a set of 1000 initial structures for each system from the Price group. For more detailed information on these initial structures the reader is referred to the respective Supporting Information of the Price group submission. For the purposes of our investigation, we used these initial geometries as starting points for further structural refinement.

For the electronic structure calculations and geometry/lattice optimizations we used the PBE [1] density functional with the pairwise Tkatchenko-Scheffler (TS) [2] and many-body dispersion (MBD) [3] methods to accurately account for the long-range vdW interactions. First, we calculated the total energy of all 1000 structures without any geometry or lattice optimization at the PBE+TS [2] level of theory with light default settings within the FHI-aims all-electron code [4–7]. Subsequently, we re-ranked the structures based on the total energy per molecule. This is necessary since the considered unit cells contain between 1 and 32 molecules. Following this re-ranking, we performed geometry optimizations (*i.e.*, with lattice constants kept fixed) at the PBE+TS level of theory (again with light default settings in FHI-aims) for the 200 lowest energy structures on this list and then re-ranked the geometry optimized structures. Next, we performed complete lattice relaxations for the 100 lowest energy structures of the previous ranking. The resulting optimized geometries were symmeterized by using PLATON [8] and now constitute our final structures.

The converged lattice energies for these 100 structures were then calculated using tight numerical settings in FHI-aims at the PBE+MBD level of theory. This final ranking, which can be found for all systems studied in this work is based on relative lattice energetics with respect to the most stable structure. Here, we do not account for the fact that the structures have slightly different densities in general.

By now, it is also well-known that vibrational free energies can have a significant influence on the relative stabilities of molecular crystal structures [9]. Therefore, we provide a second list (for the first two systems only) that contains the rankings of about 20 structures based on the sum of the PBE+MBD energy and the vibrational free energy at 300 K. The phonon calculations required for this free energy correction were carried out with the finite difference method using FHI-aims and phonopy [10]. All force calculations were performed at the PBE+TS level of theory using light default settings. Calculations were performed on 1.6 GHz PowerPC and 2.6 GHz Intel Sandy Bridge-EP cores.

SYSTEM XXII

For System XXII, the initial structures were calculated by Rebecca Hylton from the Price group (see the corresponding Supporting Information) and the ranking procedure was exactly as described in the General Methodology section above. The ranking of the 100 lattice-optimized structures according to PBE+MBD energies is shown in Table I. The structure name always corresponds to the name of the initial structure from the Price group which was used as the starting point for all of our optimizations. E_{rel} is the relative lattice energy per molecule compared to the lowest energy structure and V corresponds to the volume per molecule in the unit cell. We observed 8 structures within an energy window of 1 kcal/mol with the first two essentially degenerate in energy (0.7 kJ/mol/molecule difference). Most of the lowest-energy structures have 4 molecules per unit cell.

TABLE I: XXII stability ranking based on PBE+MBD energies.

Rank	Name	E_{rel} [kJ/mol/molec.]	Z	Symm.	a [Å]	b [Å]	c [Å]	α [deg.]	β [deg.]	γ [deg.]	V [Å^3/molec.]	ρ [g/cm^3]
1	A8	0.00	4	P n a 21	13.5	10.1	7.1	90.0	90.0	90.0	240.4	1.715
2	A467	0.66	4	P 21/c	10.2	6.7	14.4	90.0	93.6	90.0	246.5	1.673
3	A4	2.19	4	P 21/n	12.1	6.8	12.6	89.5	107.7	90.3	246.2	1.675
4	A10	2.38	4	P 21/n	8.0	15.0	8.2	89.9	90.9	89.6	246.2	1.675
5	A5293	3.13	4	P 21 21 21	7.1	10.1	13.5	90.0	90.0	90.0	241.0	1.711
6	A645	3.70	4	P 21/c	8.3	8.1	14.6	89.9	99.5	90.4	241.5	1.707
7	A98	3.77	4	P 21/n	6.9	11.8	12.1	90.0	91.7	90.0	247.0	1.669
8	A232	3.89	4	P n m a	20.7	6.6	7.0	90.0	90.0	90.0	240.9	1.712
9	A127	4.24	4	P 21 21 21	5.7	9.0	19.1	90.0	90.0	90.0	248.3	1.660
10	A389	4.47	2	P -1	6.9	7.2	10.6	86.0	76.3	78.3	251.0	1.643
11	A19	4.59	4	P 21/n	11.8	6.3	13.4	90.0	93.7	90.0	249.2	1.655
12	A2846	4.80	4	P 21/n	9.9	9.3	11.0	90.0	96.2	90.0	250.3	1.647
13	A58	4.94	4	P 21 21 21	6.8	11.6	12.4	90.0	90.0	90.0	247.2	1.668
14	A43	4.98	4	P 21/c	11.0	7.0	12.8	89.7	95.0	89.1	244.2	1.689
15	A108	5.08	4	P 21/n	12.5	6.8	13.0	90.0	117.3	90.0	246.5	1.673
16	A134	5.09	4	P 21/c	7.3	6.7	20.2	90.0	94.3	90.0	248.9	1.656
17	A182	5.29	4	P 21/c	7.0	6.9	20.4	90.0	92.4	90.0	247.2	1.668
18	A161	5.33	4	P 21/n	12.1	6.8	12.9	90.0	110.3	90.0	248.6	1.659
19	A519	5.39	4	P 21/c	9.7	11.1	9.6	90.6	95.2	90.4	257.0	1.604
20	A7	5.48	8	P b c a	13.8	7.5	19.0	90.0	90.0	90.0	244.8	1.684
21	A97	5.49	4	I c	8.7	7.1	16.2	90.0	93.0	90.0	249.6	1.652
22	A1562	5.49	4	P 21/c	10.0	7.2	13.7	90.0	99.3	90.0	244.2	1.688
23	A4355	5.63	8	P b c a	13.4	7.6	20.0	90.0	90.0	90.0	253.2	1.629
24	A2848	5.73	4	P 21/n	9.4	9.9	11.5	89.3	111.4	90.3	248.8	1.657
25	A22	5.76	4	P 21 21 21	8.2	10.0	11.9	90.0	90.0	90.0	245.8	1.677
26	A580	5.95	4	P 21/c	7.2	7.0	19.6	90.0	91.0	90.0	247.5	1.666
27	A1393	5.98	4	P 21 21 21	6.9	10.5	13.4	90.0	90.0	90.0	243.1	1.696
28	A1	5.98	4	P 21/n	6.4	7.7	19.8	90.0	95.9	90.0	242.9	1.697
29	A215	6.03	8	P b c a	13.4	7.1	21.7	90.0	90.0	90.0	256.5	1.607
30	A32	6.05	2	P 21	7.6	6.6	9.9	90.0	90.1	90.0	249.4	1.653
31	A5945	6.28	4	P 21/c	7.2	13.4	10.3	90.0	90.3	90.0	247.8	1.664
32	A1515	6.44	2	P -1	6.8	7.5	10.0	83.7	81.2	76.5	243.9	1.690
33	A464	6.47	4	P 21/n	11.4	7.1	13.0	90.0	112.6	90.0	241.3	1.708
34	A426	6.51	4	P 21 21 21	6.9	7.3	19.6	90.0	90.0	90.0	247.5	1.666
35	A25	6.64	4	P 21/c	10.1	7.0	14.1	90.0	109.0	90.0	237.6	1.735
36	A56	6.73	4	P 21/c	10.7	6.4	14.6	90.0	91.5	90.0	250.0	1.649
37	A3726	6.74	4	P 21/n	7.0	10.4	13.5	90.0	94.7	90.0	242.8	1.698
38	A3645	6.75	4	P 21/c	10.4	13.6	7.6	90.0	109.5	90.0	252.5	1.633
39	A104	6.86	4	P 21/c	6.9	7.2	20.0	89.8	95.3	89.6	246.8	1.671
40	A180	6.87	8	P b c a	9.9	7.9	24.7	90.0	90.0	90.0	242.2	1.703
41	A1905	7.05	4	P 21/c	11.7	11.0	7.8	90.0	95.2	90.0	249.8	1.650
42	A3	7.08	8	C 2/c	18.2	7.8	14.1	90.0	101.2	90.0	246.7	1.672
43	A41	7.13	4	P 21/c	7.0	6.8	21.0	90.0	93.9	90.0	249.3	1.654
44	A5583	7.31	4	P 21/c	11.6	11.2	7.7	90.0	94.4	90.0	248.7	1.658
45	A6490	7.40	4	P 1	7.0	10.5	13.6	89.2	86.3	81.9	247.0	1.670
46	A1663	7.46	2	P 21	6.9	7.3	9.8	89.7	96.8	90.5	243.9	1.691
47	A505	7.55	8	P b c a	7.2	13.6	19.8	90.0	90.0	90.0	242.6	1.700

TABLE I: (continued) XXII stability ranking based on PBE+MBD energies.

Rank	Name	E_{rel} [kJ/mol/molec.]	Z	Symm.	a [Å]	b [Å]	c [Å]	α [deg.]	β [deg.]	γ [deg.]	V [Å^3/molec.]	ρ [g/cm^3]
48	A40	7.57	4	P 21/n	10.2	7.9	12.4	90.0	95.8	90.0	247.1	1.669
49	A9649	7.77	2	P -1	6.6	7.6	10.1	83.7	80.8	81.9	246.1	1.676
50	A741	7.89	8	P b c a	7.9	13.4	18.8	90.0	90.0	90.0	248.9	1.657
51	A3994	7.91	4	P 21/n	11.7	7.1	12.2	90.0	101.3	90.0	248.3	1.661
52	A2028	7.93	4	P 21/c	6.7	11.0	13.5	90.0	102.1	90.0	244.9	1.684
53	A123	8.02	2	P -1	7.0	7.9	9.8	84.9	71.9	71.7	246.3	1.674
54	A224	8.03	4	P c	3.9	25.4	9.7	90.0	90.2	90.0	240.1	1.718
55	A51	8.05	2	P -1	6.9	7.2	10.2	91.4	96.1	103.5	245.7	1.678
56	A7362	8.09	2	P -1	7.8	8.4	9.0	77.9	72.6	62.5	246.6	1.672
57	A243	8.21	8	P b c n	12.8	12.0	12.9	90.0	90.0	90.0	248.1	1.662
58	A762	8.25	8	P 21/c	20.1	7.3	13.6	90.0	90.8	90.0	249.3	1.654
59	A394	8.31	8	P 41 21 2	6.8	6.8	42.3	90.0	90.0	90.0	247.8	1.664
60	A413	8.35	4	P 21/c	3.8	26.9	9.4	90.0	96.3	90.0	241.0	1.711
61	A887	8.59	4	P 21/n	11.0	7.2	12.5	90.0	91.5	90.0	247.7	1.665
62	A201	8.70	4	P 21/c	7.0	13.2	10.7	90.0	99.9	90.0	245.0	1.683
63	A2056	8.99	4	P 21/c	9.4	13.5	8.3	90.0	103.4	90.0	254.7	1.619
64	A362	9.13	8	P b c n	15.3	11.1	11.7	90.0	90.0	90.0	249.5	1.653
65	A723	9.44	8	C 2/c	17.8	8.4	13.5	90.0	101.2	90.0	247.9	1.663
66	A398	9.48	4	P 21	3.9	9.8	25.6	90.0	91.0	90.0	242.0	1.704
67	A1338	9.57	8	P n	3.8	19.0	26.9	90.0	93.3	90.0	243.8	1.692
68	A57	9.73	4	P 21/c	10.1	6.8	14.6	89.1	91.5	90.3	249.4	1.653
69	A279	9.85	4	P 21 21 21	4.0	12.7	19.3	90.0	90.0	90.0	243.3	1.695
70	A509	9.91	4	P 21/c	11.3	12.2	7.3	90.0	92.1	90.0	250.5	1.646
71	A1051	10.01	2	P -1	7.2	8.3	8.8	89.7	76.0	75.4	247.6	1.665
72	A2723	10.07	8	P b c a	7.4	14.1	18.9	90.0	90.0	90.0	245.7	1.678
73	A3230	10.38	4	P 21/n	9.8	10.0	10.2	90.0	102.4	90.0	242.8	1.698
74	A206	10.43	8	C 2/c	13.9	7.1	20.2	90.0	104.8	90.0	241.9	1.705
75	A5506	10.65	4	C c	13.2	3.8	19.2	90.0	99.2	90.0	239.8	1.719
76	A1309	11.00	4	C c	3.8	26.8	9.6	90.0	100.3	90.0	241.1	1.710
77	A899	11.31	4	P 21/c	13.6	9.9	7.4	89.5	101.1	90.1	245.6	1.679
78	A1200	11.36	8	C 2/c	28.0	3.8	18.4	90.0	100.9	90.0	242.8	1.698
79	A587	11.69	8	P b c a	13.8	7.6	18.8	90.0	90.0	90.0	246.0	1.676
80	A1194	11.77	4	P n a 21	19.3	3.8	13.1	90.0	90.0	90.0	242.4	1.701
81	A981	11.80	1	P 1	3.8	6.8	9.8	96.7	99.8	103.0	240.5	1.714
82	A5575	11.87	4	P 21/c	12.8	4.0	19.8	90.0	105.6	90.0	244.1	1.689
83	A282	12.39	4	P 21/n	4.0	19.0	13.1	90.0	95.7	90.0	247.0	1.670
84	A8321	12.61	4	P 21/n	3.8	19.3	13.1	90.0	97.1	90.0	241.6	1.706
85	A827	12.75	4	C 2	19.9	4.0	12.8	90.0	106.0	90.0	244.0	1.690
86	A9983	12.77	4	P -1	3.9	9.9	25.2	89.7	88.1	83.3	241.6	1.707
87	A8915	12.78	4	P n a 21	24.4	10.3	3.9	90.0	90.0	90.0	246.2	1.675
88	A1697	13.00	4	P n a 21	14.2	17.8	4.0	90.0	90.0	90.0	250.2	1.648
89	A1652	13.16	4	P 21 21 21	3.9	10.2	24.6	90.0	90.0	90.0	243.6	1.693
90	A2004	13.30	2	P n	10.7	3.8	12.5	89.6	110.1	89.9	241.7	1.706
91	A3314	13.41	8	C 2/c	28.5	3.8	18.4	90.0	101.3	90.0	243.6	1.692
92	A605	13.64	4	P n a 21	19.3	3.8	13.0	90.0	90.0	90.0	241.1	1.710
93	A879	13.68	4	P c a 21	19.5	3.8	13.0	90.0	90.0	90.0	243.2	1.695
94	A424	13.69	2	P 21	3.8	12.9	9.8	90.0	98.5	90.0	240.7	1.713
95	A1427	13.71	2	P n	3.8	9.8	13.0	90.0	95.3	90.0	242.6	1.700
96	A1414	14.06	4	P 21/c	13.9	9.7	7.4	90.9	92.8	90.0	249.5	1.653
97	A124	14.08	4	P 21/c	6.4	19.3	8.0	90.0	94.8	89.4	245.7	1.678
98	A1050	14.20	4	P 21/c	13.7	9.8	7.6	89.1	97.9	90.3	251.4	1.640
99	A609	14.29	8	C 2/c	20.1	4.0	25.4	90.0	105.3	90.0	244.0	1.690
100	A516	14.37	4	P 21/n	14.2	3.9	18.0	90.0	105.5	90.0	244.3	1.688

The previously shown ranking considers only the PBE+MBD total energies, so there is no account of zero point motion and thermal vibrations. To estimate the energy ranking at room temperature we have added the harmonic vibrational free energy (calculated at the PBE+TS level of theory) at 300 K to the PBE+MBD energies. This ranking considers the top 20 structures of Table I and is given in Table II. All calculations for this system needed roughly 100,000 CPU hours (with 30,000 hours for the phonon calculations).

TABLE II: XXII stability ranking based on PBE+MBD energies and PBE+TS vibrational free energies at 300 K.

Rank	Name	E_{rel} [kJ/mol/molec.]	Z	Symm.	a [Å]	b [Å]	c [Å]	α [deg.]	β [deg.]	γ [deg.]	V [Å^3/molec.]	ρ [g/cm^3]
1	A4	0.00	4	P 21/n	12.1	6.8	12.6	89.5	107.7	90.3	246.2	1.675
2	A467	0.11	4	P 21/c	10.2	6.7	14.4	90.0	93.6	90.0	246.5	1.673
3	A8	1.08	4	P n a 21	13.5	10.1	7.1	90.0	90.0	90.0	240.4	1.715
4	A134	3.23	4	P 21/c	7.3	6.7	20.2	90.0	94.3	90.0	248.9	1.656
5	A5293	3.50	4	P 21 21 21	7.1	10.1	13.5	90.0	90.0	90.0	241.0	1.711
6	A98	3.52	4	P 21/n	6.9	11.8	12.1	90.0	91.7	90.0	247.0	1.669
7	A10	3.63	4	P 21/n	8.0	15.0	8.2	89.9	90.9	89.6	246.2	1.675
8	A58	3.73	4	P 21 21 21	6.8	11.6	12.4	90.0	90.0	90.0	247.2	1.668
9	A389	4.21	2	P -1	6.9	7.2	10.6	86.0	76.3	78.3	251.0	1.643
10	A2846	4.33	4	P 21/n	9.9	9.3	11.0	90.0	96.2	90.0	250.3	1.647
11	A182	4.41	4	P 21/c	7.0	6.9	20.4	90.0	92.4	90.0	247.2	1.668
12	A127	4.53	4	P 21 21 21	5.7	9.0	19.1	90.0	90.0	90.0	248.3	1.660
13	A19	4.63	4	P 21/n	11.8	6.3	13.4	90.0	93.7	90.0	249.2	1.655
14	A161	4.77	4	P 21/n	12.1	6.8	12.9	90.0	110.3	90.0	248.6	1.659
15	A645	4.88	4	P 21/c	8.3	8.1	14.6	89.9	99.5	90.4	241.5	1.707
16	A232	4.90	4	P n m a	20.7	6.6	7.0	90.0	90.0	90.0	240.9	1.712
17	A519	5.00	4	P 21/c	9.7	11.1	9.6	90.6	95.2	90.4	257.0	1.604
18	A43	5.10	4	P 21/c	11.0	7.0	12.8	89.7	95.0	89.1	244.2	1.689
19	A108	5.38	4	P 21/n	12.5	6.8	13.0	90.0	117.3	90.0	246.5	1.673
20	A7	5.77	8	P b c a	13.8	7.5	19.0	90.0	90.0	90.0	244.8	1.684

SYSTEM XXIII

For System XXIII, the initial structures were generated by Louise Price (see Supporting Information from Price group) and the ranking procedure was exactly as described in the General Methodology section above. Since the ranking of the 100 lattice-optimized structures revealed a lot of duplicate structures, we omitted all duplicates in the final energy ranking resulting in a list with 31 structures. The ranking according to PBE+MBD energies is shown in Table III. The structure name always corresponds to the name of the initial structure from the Price group which was used as the starting point for all of our optimizations. $E_{\rm rel}$ is the relative lattice energy per molecule compared to the lowest energy structure, V corresponds to the volume per molecule in the unit cell, and the number of duplicates observed within the top 100 structures is given in the last column. Here, we observed 19 structures within an energy window of 1 kcal/mol with the first five essentially degenerate in energy (the difference is < 1 kJ/mol/molecule).

TABLE III: XXIII stability ranking based on PBE+MBD energies.

Rank	Name	$E_{\rm rel}$ [kJ/mol/molec.]	Z	Symm.	a [Å]	b [Å]	c [Å]	α [deg.]	β [deg.]	γ [deg.]	V [Å^3/molec.]	ρ [g/cm^3]	Dupl.
1	B45	0.00	2	P -1	7.4	9.5	14.4	96.1	102.3	112.6	447.8	1.433	4
2	A3661	0.29	2	P -1	7.1	7.8	18.0	88.9	81.2	64.9	444.0	1.444	24
3	B116	0.44	4	P 21/n	7.5	27.2	9.5	90.0	111.1	90.0	450.6	1.423	2
4	A63	0.46	8	I 2/c	23.5	4.4	34.7	90.0	96.9	90.0	446.2	1.438	1
5	A7573	0.50	2	P -1	4.5	11.9	17.6	77.3	83.9	84.0	449.3	1.428	8
6	A2463	1.24	2	P -1	6.7	8.9	15.6	85.4	84.5	79.6	449.7	1.426	6
7	A191	2.05	8	C 2/c	35.0	4.4	23.5	90.0	97.0	90.0	454.0	1.413	1
8	B20	2.13	2	P -1	7.5	11.8	12.0	63.1	86.2	74.6	454.7	1.410	2
9	A75	2.14	4	P 21/c	4.3	24.4	17.1	90.0	96.6	90.0	440.4	1.456	1
10	A2073	2.30	2	P -1	7.1	7.7	18.2	87.3	80.4	64.8	445.3	1.440	2
11	A3937	2.42	2	P -1	7.3	7.5	18.1	81.9	84.6	66.9	452.6	1.417	4
12	B198	2.81	4	P 21/c	18.7	9.6	10.1	90.0	90.3	90.0	454.7	1.411	1
13	A729	3.27	4	P 21/c	7.5	34.6	7.4	90.0	111.3	90.0	448.3	1.431	5
14	A506	3.28	4	P 21/n	13.4	10.8	14.2	90.0	115.4	90.0	462.9	1.386	2
15	A2487	3.30	2	P -1	3.9	15.0	15.7	71.2	87.8	89.0	432.4	1.483	1
16	A214	3.56	4	P 21/c	7.0	36.8	7.7	90.0	114.4	90.0	454.6	1.411	1
17	A988	3.74	4	P 21/c	11.0	11.2	14.8	90.0	95.5	90.0	455.6	1.408	1
18	A385	4.01	2	P -1	6.6	7.6	19.9	94.2	95.5	114.7	446.7	1.436	3
19	A53	4.13	2	P -1	7.1	7.5	18.8	80.7	83.4	67.3	453.7	1.414	3
20	A656	4.49	2	P -1	6.7	8.8	15.4	92.7	97.9	100.4	444.0	1.445	3
21	A283	5.30	2	P -1	7.4	11.3	11.5	110.5	91.3	90.7	450.6	1.423	2
22	A87	5.43	4	P 21/n	8.2	11.2	19.5	89.7	90.9	89.5	450.8	1.423	1
23	A80	5.59	2	P -1	3.9	8.6	26.5	88.2	87.5	81.5	437.0	1.468	2
24	A55	6.58	2	P -1	6.9	9.6	14.2	102.2	93.0	98.9	452.2	1.419	4
25	A2127	6.79	2	P -1	6.8	8.6	17.1	92.1	99.2	112.4	456.7	1.404	3
26	A157	6.80	2	P -1	3.9	8.6	27.0	92.0	90.7	103.1	439.1	1.461	1
27	A251	6.98	2	P -1	4.4	13.0	17.1	107.0	91.3	96.9	466.5	1.375	1
28	A96	7.78	4	P 21/c	11.7	4.5	33.7	90.0	95.5	90.0	443.1	1.448	1
29	A166	7.99	4	P 21/n	4.5	31.9	13.2	90.0	95.1	90.0	465.9	1.377	1
30	A24	8.02	4	P 21 21 21	4.5	11.6	33.6	90.0	90.0	90.0	440.9	1.455	2
31	A1266	9.18	4	P 21/n	8.2	20.1	11.2	90.0	99.1	90.0	453.4	1.415	1

In order to estimate the energy ranking at room temperature, we have added the harmonic vibrational free energy (calculated at the PBE+TS level) at 300 K to the PBE+MBD energies. This ranking considers the top 19 structures of Table III and is given in Table IV. All calculations for this system needed roughly 2.1 million CPU hours (with 1.3 million CPU hours for phonon calculations).

TABLE IV: XXIII stability ranking based on PBE+MBD energies and PBE+TS vibrational free energies at 300 K.

Rank	Name	$E_{\rm rel}$ [kJ/mol/molec.]	Z	Symm.	a [Å]	b [Å]	c [Å]	α [deg.]	β [deg.]	γ [deg.]	V [Å^3/molec.]	ρ [g/cm^3]	Dupl.
1	A2463	0.00	2	P -1	6.7	8.9	15.6	85.4	84.5	79.6	449.7	1.426	6
2	A506	1.36	4	P 21/n	13.4	10.8	14.2	90.0	115.4	90.0	462.9	1.386	2
3	B45	1.65	2	P -1	7.4	9.5	14.4	96.1	102.3	112.6	447.8	1.433	4
4	A7573	1.73	2	P -1	4.5	11.9	17.6	77.3	83.9	84.0	449.3	1.428	8
5	A3661	2.07	2	P -1	7.1	7.8	18.0	88.9	81.2	64.9	444.0	1.444	24
6	A63	2.08	8	I 2/c	23.5	4.4	34.7	90.0	96.9	90.0	446.2	1.438	1
7	A191	2.64	8	C 2/c	35.0	4.4	23.5	90.0	97.0	90.0	454.0	1.413	1
8	B116	2.94	4	P 21/n	7.5	27.2	9.5	90.0	111.1	90.0	450.6	1.423	2
9	A988	4.90	4	P 21/c	11.0	11.2	14.8	90.0	95.5	90.0	455.6	1.408	1
10	B20	6.07	2	P -1	7.5	11.8	12.0	63.1	86.2	74.6	454.7	1.410	2
11	A75	6.60	4	P 21/c	4.3	24.4	17.1	90.0	96.6	90.0	440.4	1.456	1
12	B198	7.54	4	P 21/c	18.7	9.6	10.1	90.0	90.3	90.0	454.7	1.411	1
13	A2487	7.81	2	P -1	3.9	15.0	15.7	71.2	87.8	89.0	432.4	1.483	1
14	A2073	9.14	2	P -1	7.1	7.7	18.2	87.3	80.4	64.8	445.3	1.440	2
15	A214	10.25	4	P 21/c	7.0	36.8	7.7	90.0	114.4	90.0	454.6	1.411	1
16	A385	10.32	2	P -1	6.6	7.6	19.9	94.2	95.5	114.7	446.7	1.436	3
17	A3937	11.92	2	P -1	7.3	7.5	18.1	81.9	84.6	66.9	452.6	1.417	4
18	A53	13.10	2	P -1	7.1	7.5	18.8	80.7	83.4	67.3	453.7	1.414	3
19	A729	13.47	4	P 21/c	7.5	34.6	7.4	90.0	111.3	90.0	448.3	1.431	5

SYSTEM XXIV

For System XXIV, the initial structures were calculated by Rui Guo from the Price group (see the corresponding Supporting Information). For this system we needed to reduce the computational cost compared to the procedure described above in the General Methodology section. Therefore, we calculated the initial ranking as before, but then we proceeded with PBE+TS lattice optimizations using lighter convergence criteria for the top 100 structures. The final PBE+MBD energy ranking with tight default settings was done for the top 50 structures after lattice optimizations. The result of this ranking is shown in Table V. The structure name always corresponds to the name of the initial structure from the Price group which was used as the starting point of our optimization. E_{rel} is the relative lattice energy per trimer ($C_4H_7N_2O_2S + Cl + H_2O$) compared to the lowest energy structure and V corresponds to the volume per trimer in the unit cell. Here, we observed 7 structures within an energy window of 1 kcal/mol with the first two being essentially degenerate (the difference amounts to 0.76 kJ/mol/molecule). All calculations for this system needed roughly 0.5 million CPU hours.

TABLE V: XXIV stability ranking based on PBE+MBD energies.

Rank	Name	E_{rel} [kJ/mol/trimer]	Z	Symm.	a [Å]	b [Å]	c [Å]	α [deg.]	β [deg.]	γ [deg.]	V [Å^3/trimer]	ρ [g/cm^3]
1	1192	0.00	4	P 21/c	6.3	18.7	7.5	90.0	100.1	90.0	219.4	1.519
2	757	0.76	4	P 21 21 21	3.9	10.3	21.8	90.0	90.0	90.0	218.4	1.526
3	1514	2.94	4	P 21/c	8.9	13.7	7.9	90.0	103.3	90.0	232.8	1.431
4	2167	2.96	4	P 21/c	6.1	15.4	9.8	90.0	103.9	90.0	224.1	1.487
5	420	3.26	4	P 21/c	4.0	10.3	21.0	90.0	90.5	90.0	214.3	1.555
6	483	3.75	3	P 1	8.0	8.1	12.5	89.6	89.2	60.8	236.1	1.411
7	2106	4.10	4	P 21	7.3	11.0	11.0	90.0	92.5	90.0	220.9	1.508
8	1114	5.89	4	P 21/c	9.5	7.3	13.1	90.0	107.8	90.0	215.5	1.546
9	159	6.52	4	P 21/c	10.6	5.5	15.2	89.5	101.4	89.8	218.8	1.523
10	2385	6.54	2	P -1	6.4	7.4	10.0	74.6	87.0	81.3	225.4	1.478
11	266	6.67	4	P 21/n	7.0	13.0	10.2	90.0	110.0	90.0	218.8	1.523
12	i806	6.69	4	P 21/c	5.2	23.6	7.4	89.6	100.0	90.2	223.3	1.492
13	1389	6.69	2	P -1	7.0	7.6	9.6	75.0	84.2	66.7	226.0	1.475
14	1055	6.77	2	P -1	6.3	7.4	10.0	75.1	87.8	82.0	224.4	1.485
15	1928	6.78	4	P 21 21 21	7.1	10.8	12.0	90.0	90.0	90.0	231.7	1.438
16	1010	6.79	4	P 21 21 21	7.1	10.8	12.0	90.0	90.0	90.0	231.6	1.439
17	122	6.84	8	C 2/c	17.0	7.6	13.8	90.0	95.5	90.0	220.6	1.510
18	1599	7.06	4	P 21/c	7.0	10.9	12.3	90.0	105.4	90.0	224.3	1.485
19	1805	7.23	4	P n a 21	6.8	9.8	13.1	90.0	90.0	90.0	218.3	1.526
20	2189	7.28	4	P 21 21 21	7.7	9.7	12.1	90.0	90.0	90.0	226.3	1.472
21	350	7.34	4	P 21/c	9.7	7.1	12.9	90.0	95.0	90.0	222.2	1.499
22	1987	7.37	8	P b c a	10.1	13.1	13.8	90.0	90.0	90.0	229.0	1.455
23	778	7.72	4	P 21 21 21	7.4	8.6	14.3	90.0	90.0	90.0	225.0	1.481
24	2072	7.75	8	C 2/c	13.0	12.5	11.1	90.0	97.3	90.0	223.9	1.488
25	220	7.90	8	P 21/c	13.1	10.1	13.9	89.2	100.3	89.9	226.8	1.469
26	1519	8.49	2	P 1	4.0	9.4	11.9	89.1	88.9	82.7	219.7	1.517
27	1931	8.55	4	P 21/n	8.4	10.3	10.4	90.0	94.2	90.0	222.8	1.495
28	1748	8.59	4	P 21/n	6.8	13.1	10.2	90.0	100.1	90.0	224.8	1.482
29	1082	9.03	16	F d d 2	12.3	18.2	16.2	90.0	90.0	90.0	226.2	1.473
30	1021	9.09	4	I c	8.6	8.9	12.1	90.0	106.1	90.0	221.6	1.503
31	1332	9.10	4	I a	8.7	8.8	12.1	90.0	106.4	90.0	221.6	1.503
32	187	9.30	4	P 21/n	10.6	7.4	11.6	90.0	102.6	90.0	220.3	1.513
33	1331	9.33	4	P 21 21 21	7.3	10.0	12.7	90.0	90.0	90.0	232.5	1.433
34	711	9.44	4	P 21/c	7.8	18.0	6.9	90.0	113.6	90.0	221.8	1.502
35	1845	9.48	4	P 21/c	7.9	17.8	6.9	90.0	113.4	90.0	222.2	1.499
36	721	9.59	4	P 21/c	7.8	18.0	6.8	90.0	113.1	90.0	221.7	1.503
37	1376	9.64	4	P 2/n	7.7	11.8	10.2	90.0	100.3	90.0	227.2	1.466
38	755	10.14	4	P 21/c	6.9	10.0	13.0	90.0	101.3	90.0	217.9	1.529
39	1458	10.32	4	I c	6.8	9.8	13.3	90.0	102.7	90.0	216.2	1.541
40	i445	10.45	4	P 21 21 21	7.4	10.3	11.9	90.0	90.0	90.0	227.1	1.467
41	484	11.17	4	P 21/c	8.9	12.5	8.7	89.7	115.6	90.3	219.2	1.520
42	144	11.20	4	P 21/c	5.4	14.7	11.2	90.0	100.3	90.0	220.3	1.513
43	780	11.26	4	P 21/c	8.7	13.0	8.0	90.0	108.2	90.0	214.4	1.554

TABLE V: (continued) XXIV stability ranking based on PBE+MBD energies.

Rank	Name	E_{rel} [kJ/mol/trimer]	Z	Symm.	a [Å]	b [Å]	c [Å]	α [deg.]	β [deg.]	γ [deg.]	V [Å^3/trimer]	ρ [g/cm^3]
44	1152	11.27	4	P 21/c	8.7	12.9	8.0	90.0	108.3	90.0	214.0	1.557
45	1703	11.36	4	P 21/c	5.5	14.7	11.2	90.0	100.5	90.0	220.8	1.509
46	43	11.63	8	P b c a	6.9	11.4	22.2	90.0	90.0	90.0	217.4	1.533
47	634	12.16	2	P -1	7.0	7.9	8.7	72.6	86.2	70.0	215.9	1.544
48	325	13.13	8	P -1	10.0	13.0	13.5	96.3	90.2	95.4	216.8	1.537
49	390	13.31	8	P b c n	12.6	10.0	13.7	90.0	90.0	90.0	215.2	1.548
50	56	13.57	8	P b c a	13.0	10.0	13.4	90.0	90.0	90.0	220.0	1.514

SYSTEM XXV

For System XXV, the initial structures were calculated by Rona Watson from the Price group (see the corresponding Supporting Information). Due to the size of this system, we could only do very limited calculations. We calculated the initial ranking as above, but then we proceeded with PBE+TS lattice optimizations using lighter convergence criteria for the top 20 structures only. The final PBE+MBD energy ranking was done with light default settings based on these 20 optimized geometries. The result of this ranking is shown in Table VI. The structure name always corresponds to the name of the initial structure from the Price group which was used as the starting point for all of our optimizations. E_{rel} is the relative lattice energy per co-crystal dimer compared to the lowest energy structure and V corresponds to the volume of one co-crystal dimer. The two most stable structures are within an energy window of 1 kcal/mol (2.9 kJ/mol/dimer difference). All calculations for this system needed roughly 0.5 million CPU hours.

TABLE VI: XXV stability ranking based on PBE+MBD energies.

Rank	Name	E_{rel} [kJ/mol/dimer]	Z	Symm.	a [Å]	b [Å]	c [Å]	α [deg.]	β [deg.]	γ [deg.]	V [Å^3/dimer]	ρ [g/cm^3]
1	B1	0.00	4	P 21/c	10.6	27.8	7.8	90.0	111.1	90.0	538.7	1.426
2	A1954	2.93	4	P 21/c	14.2	20.4	7.5	90.0	97.2	90.0	540.3	1.421
3	A1928	4.32	2	P -1	8.2	11.4	12.7	106.2	96.2	107.5	532.8	1.441
4	A8936	4.46	2	P -1	7.6	10.8	13.8	104.8	92.5	97.6	542.7	1.415
5	A17	4.57	4	P 21/n	13.3	8.1	21.0	89.4	106.7	90.2	538.5	1.426
6	A1238	4.84	2	P -1	9.6	10.2	12.5	92.2	98.5	114.9	545.5	1.408
7	A613	5.12	8	P b c a	20.9	7.6	28.9	90.0	90.0	90.0	577.6	1.330
8	A1477	5.16	2	P -1	7.6	10.9	13.6	104.1	92.5	97.5	542.2	1.416
9	A12	5.55	2	P -1	6.3	13.7	14.3	64.1	84.4	76.8	542.1	1.417
10	A13	6.97	8	P b c n	27.4	10.2	15.7	90.0	90.0	90.0	551.3	1.393
11	A5	7.19	2	P -1	6.9	11.1	15.3	103.4	94.2	107.0	536.6	1.431
12	B483	7.27	4	P 21/c	8.3	18.3	14.3	90.0	94.8	90.0	540.6	1.421
13	A3308	7.74	4	P 21/c	10.3	9.7	22.4	90.0	99.8	90.0	552.3	1.391
14	A1	8.13	4	P 21/c	15.4	6.6	22.7	90.0	106.1	90.0	554.2	1.386
15	A47	8.85	8	I 2/a	22.7	7.9	26.0	90.0	110.1	90.0	545.8	1.407
16	A4	9.06	2	P -1	6.9	11.4	15.1	103.8	94.2	106.9	540.1	1.422
17	A50	10.34	8	A b a 2	28.6	20.0	7.8	90.0	90.0	90.0	557.1	1.378
18	B62	10.75	4	P 21/n	12.8	7.9	22.1	90.0	103.8	90.0	540.7	1.420
19	A125	15.93	2	P -1	7.7	11.0	13.9	69.6	80.2	77.6	535.5	1.434
20	A931	17.13	2	P -1	7.8	10.8	13.9	110.9	93.7	99.4	540.1	1.422

POST-ANALYSIS

XXII

The experimentally observed structure was predicted at rank 1 in list 2, which includes vibrational free energy contributions. It can be seen that free energies are already important for this small and quite rigid system since this structure is located at rank 3 by considering only PBE+MBD energies (list 1). Here the difference to rank 1 amounts to 2.2 kJ/mol/molecule.

XXIII

We have correctly predicted form b and d within our top 5 structures of list 2 (including vibrational free energies). We did not search explicitly for $Z' = 2$ structures (form c and e). For this post-analysis we have taken the experimental structures as input and we performed all calculations as described before. Figure 1 shows the two rankings for the initially predicted structures (all structures for which free energies were calculated) and the PBE+TS-optimized experimental structures. For the PBE+MBD energy ranking (list 1) form c is now the most stable structure and all experimentally observed structures are found within an energy interval of about 5 kJ/mol/molecule. By considering vibrational free energies (list 2) the relative ordering of the experimental structures changes significantly and form e becomes the most stable structure. It can be seen that inclusion of vibrational free energies leads to a spreading of the energy interval from about 6 to about 14 kJ/mol/molecule. In contrast, all experimental structures are now within 2.9 kJ/mol/molecule. Lattice parameters and relative energies compared to Table IV are shown in Table VII.

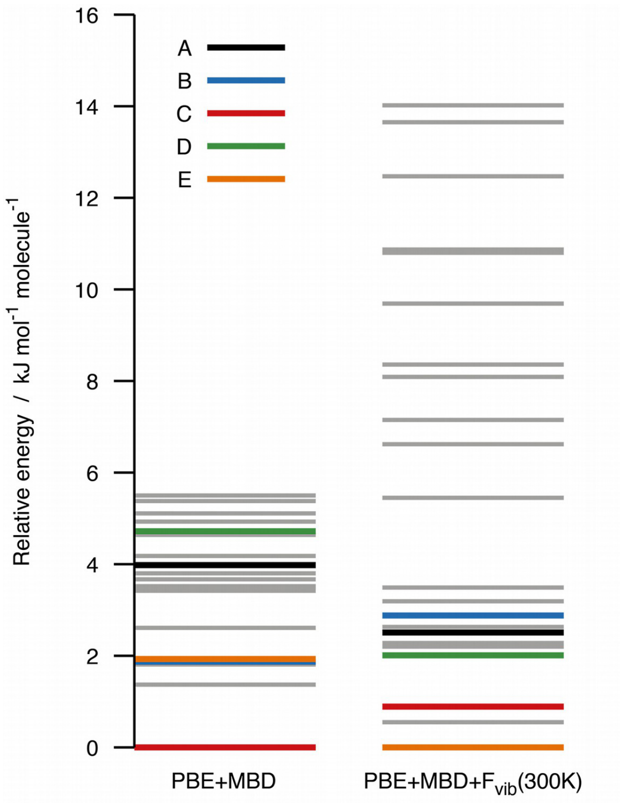

FIG. 1. Relative ranking of PBE+TS-optimized experimental structures (color) and predicted structures (gray).

TABLE VII: Data for PBE+TS-optimized experimental structures of system XXIII. The stability ranking is based on the sum of PBE+MBD energies and PBE+TS vibrational free energies at 300 K. The energy values are relative to the most stable structure in Table IV.

Structure	E_{rel} [kJ/mol/molec.]	Z	Symm.	a [Å]	b [Å]	c [Å]	α [deg.]	β [deg.]	γ [deg.]	V [Å^3/molec.]	ρ [g/cm^3]
A	1.96	4	P 21/c	11.0	10.3	16.2	90.0	94.2	90.0	460.4	1.393
B	2.33	2	P -1	7.1	7.8	18.0	89.2	81.4	64.8	444.3	1.444
C	0.34	4	P 21/c	7.5	11.9	20.2	86.8	87.1	79.9	445.2	1.441
D	1.46	4	P 21/n	13.4	10.8	14.2	90.0	115.3	90.0	463.1	1.385
E	−0.55	4	P -1	6.6	11.7	23.9	97.6	97.7	96.6	452.1	1.419

XXIV

For system xxiv we could not predict the correct crystal structure. The molecular arrangement and hydrogen-bonding pattern of the experimental structure was not present in our set of initial structures. Recalculation based on the experimental structure (see Table VIII) revealed that this structure is by 6.4 kJ/mol/trimer more stable than our most stable predicted structure.

TABLE VIII: Data for the PBE+TS-optimized experimental structure of system XXIV. The energy is relative to the most stable structure in Table V.

Structure	E_{rel} [kJ/mol/trimer]	Z	Symm.	a [Å]	b [Å]	c [Å]	α [deg.]	β [deg.]	γ [deg.]	V [Å^3/trimer]	ρ [g/cm^3]
Exp	−6.40	4	P 21/c	4.1	21.0	10.0	90.0	98.8	90.0	424.4	1.570

XXV

The experimentally observed structure was already predicted at rank 1.

XXVI

Due to time reasons we did not predict structures for system XXVI in the actual blind test. But for the post-analysis we performed all calculations in the same way as for system XXV based on 1000 initial structures generated by Luca Iuzzolino of the Price group. Price *et al.* predicted the experimental structure on rank 1 of their second list. With our procedure we would have predicted the experimental geometry on rank 1 as well. The lattice parameters of this structure are given in Table IX. The energy difference to rank 2 amounts to 0.7 kJ/mol/molecule.

TABLE IX: Data for the most stable structure of system XXVI according to PBE+MBD energies.

Structure	Z	Symm.	a [Å]	b [Å]	c [Å]	α [deg.]	β [deg.]	γ [deg.]	V [Å^3/molec.]	ρ [g/cm^3]	RMSD_{20} [Å]	RMSD_1 [Å]
1600	2	P -1	10.4	10.7	13.9	79.5	74.0	65.4	677.0	1.377	0.358	0.145

ACKNOWLEDGMENT

J.H. and A.T. acknowledge the support from the Deutsche Forschungsgemeinschaft under the program DFG-SPP 1807. H-Y.K., R.A.D., and R.C. acknowledge support from the Department of Energy (DOE) under Grant Nos. DE-SC0008626. This research used resources of the Argonne Leadership Computing Facility at Argonne National Laboratory, which is supported by the Office of Science of the U.S. Department of Energy under Contract No. DE-AC02-06CH11357. This research used resources of the National Energy Research Scientific Computing Center, which is supported by the Office of Science of the U.S. Department of Energy under Contract No. DE-AC02-05CH11231. Additional computational resources were provided by the Terascale Infrastructure for Groundbreaking Research in Science and Engineering (TIGRESS) High Performance Computing Center and Visualization Laboratory at Princeton University.

* E-mail: tkatchen@fhi-berlin.mpg.de

[1] J. P. Perdew, K. Burke, and M. Ernzerhof, Phys. Rev. Lett. **77**, 3865 (1996).
[2] A. Tkatchenko and M. Scheffler, Phys. Rev. Lett. **102**, 073005 (2009).
[3] A. Tkatchenko, R. A. DiStasio Jr., R. Car, and M. Scheffler, Phys. Rev. Lett. **108**, 236402 (2012).
[4] V. Blum, R. Gehrke, F. Hanke, P. Havu, V. Havu, X. Ren, K. Reuter, and M. Scheffler, Comput. Phys. Commun. **180**, 2175 (2009).
[5] A. Marek, V. Blum, R. Johanni, V. Havu, B. Lang, T. Auckenthaler, A. Heinecke, H.-J. Bungartz, and H. Lederer, J. Phys.: Condens. Matter **26**, 213201 (2014).
[6] T. Auckenthaler, V. Blum, H.-J. Bungartz, T. Huckle, R. Johanni, L. Krmer, B. Lang, H. Lederer, and P. Willems, Parallel Computing **37**, 783 (2011), 6th International Workshop on Parallel Matrix Algorithms and Applications (PMAA'10).
[7] V. Havu, V. Blum, P. Havu, and M. Scheffler, J. Comput. Phys. **228**, 8367 (2009).
[8] A. L. Spek, Acta Crystallogr. Sect. D **65**, 148 (2009).
[9] A. M. Reilly and A. Tkatchenko, Phys. Rev. Lett. **113**, 055701 (2014).
[10] A. Togo and I. Tanaka, Scr. Mater. **108**, 1 (2015).
